# Age–sex differences in the global burden of lower respiratory infections and risk factors, 1990–2019: results from the Global Burden of Disease Study 2019

**DOI:** 10.1016/S1473-3099(22)00510-2

**Published:** 2022-11

**Authors:** GBD 2019 LRI Collaborators, Hmwe Hmwe Kyu, Avina Vongpradith, Sarah Brooke Sirota, Amanda Novotney, Christopher E Troeger, Matthew C Doxey, Rose G Bender, Jorge R Ledesma, Molly H Biehl, Samuel B Albertson, Joseph Jon Frostad, Katrin Burkart, Fiona B Bennitt, Jeff T Zhao, William M Gardner, Hailey Hagins, Dana Bryazka, Regina-Mae Villanueva Dominguez, Semagn Mekonnen Abate, Michael Abdelmasseh, Amir Abdoli, Gholamreza Abdoli, Aidin Abedi, Vida Abedi, Tadesse M Abegaz, Hassan Abidi, Richard Gyan Aboagye, Hassan Abolhassani, Yonas Derso Abtew, Hiwa Abubaker Ali, Eman Abu-Gharbieh, Ahmed Abu-Zaid, Kidist Adamu, Isaac Yeboah Addo, Oyelola A Adegboye, Mohammad Adnan, Qorinah Estiningtyas Sakilah Adnani, Muhammad Sohail Afzal, Saira Afzal, Bright Opoku Ahinkorah, Aqeel Ahmad, Araz Ramazan Ahmad, Sajjad Ahmad, Ali Ahmadi, Sepideh Ahmadi, Haroon Ahmed, Jivan Qasim Ahmed, Tarik Ahmed Rashid, Mostafa Akbarzadeh-Khiavi, Hanadi Al Hamad, Luciana Albano, Mamoon A Aldeyab, Bezatu Mengistie Alemu, Kefyalew Addis Alene, Abdelazeem M Algammal, Fadwa Alhalaiqa Naji Alhalaiqa, Robert Kaba Alhassan, Beriwan Abdulqadir Ali, Liaqat Ali, Musa Mohammed Ali, Syed Shujait Ali, Yousef Alimohamadi, Vahid Alipour, Adel Al-Jumaily, Syed Mohamed Aljunid, Sami Almustanyir, Rajaa M Al-Raddadi, Rami H Hani Al-Rifai, Saif Aldeen S AlRyalat, Nelson Alvis-Guzman, Nelson J Alvis-Zakzuk, Edward Kwabena Ameyaw, Javad Javad Aminian Dehkordi, John H Amuasi, Dickson A Amugsi, Etsay Woldu Anbesu, Adnan Ansar, Anayochukwu Edward Anyasodor, Jalal Arabloo, Demelash Areda, Ayele Mamo Argaw, Zeleke Gebru Argaw, Judie Arulappan, Raphael Taiwo Aruleba, Mulusew A Asemahagn, Seyyed Shamsadin Athari, Daniel Atlaw, Engi F Attia, Sameh Attia, Avinash Aujayeb, Tewachew Awoke, Tegegn Mulatu Ayana, Martin Amogre Ayanore, Sina Azadnajafabad, Mohammadreza Azangou-Khyavy, Samad Azari, Amirhossein Azari Jafari, Muhammad Badar, Ashish D Badiye, Nayereh Baghcheghi, Sara Bagherieh, Atif Amin Baig, Maciej Banach, Indrajit Banerjee, Mainak Bardhan, Francesco Barone-Adesi, Hiba Jawdat Barqawi, Amadou Barrow, Azadeh Bashiri, Quique Bassat, Abdul-Monim Mohammad Batiha, Abate Bekele Belachew, Melaku Ashagrie Belete, Uzma Iqbal Belgaumi, Akshaya Srikanth Bhagavathula, Nikha Bhardwaj, Pankaj Bhardwaj, Parth Bhatt, Vijayalakshmi S Bhojaraja, Zulfiqar A Bhutta, Soumitra S Bhuyan, Ali Bijani, Saeid Bitaraf, Belay Boda Abule Bodicha, Nikolay Ivanovich Briko, Danilo Buonsenso, Muhammad Hammad Butt, Jiao Cai, Paulo Camargos, Luis Alberto Cámera, Promit Ananyo Chakraborty, Muluken Genetu Chanie, Jaykaran Charan, Vijay Kumar Chattu, Patrick R Ching, Sungchul Choi, Yuen Yu Chong, Sonali Gajanan Choudhari, Enayet Karim Chowdhury, Devasahayam J Christopher, Dinh-Toi Chu, Natalie L Cobb, Aaron J Cohen, Natália Cruz-Martins, Omid Dadras, Fentaw Teshome Dagnaw, Xiaochen Dai, Lalit Dandona, Rakhi Dandona, An Thi Minh Dao, Sisay Abebe Debela, Biniyam Demisse, Fitsum Wolde Demisse, Solomon Demissie, Diriba Dereje, Hardik Dineshbhai Desai, Abebaw Alemayehu Desta, Belay Desye, Sameer Dhingra, Nancy Diao, Daniel Diaz, Lankamo Ena Digesa, Linh Phuong Doan, Milad Dodangeh, Deepa Dongarwar, Fariba Dorostkar, Wendel Mombaque dos Santos, Haneil Larson Dsouza, Eleonora Dubljanin, Oyewole Christopher Durojaiye, Hisham Atan Edinur, Elham Ehsani-Chimeh, Ebrahim Eini, Michael Ekholuenetale, Temitope Cyrus Ekundayo, Eman D El Desouky, Iman El Sayed, Maysaa El Sayed Zaki, Muhammed Elhadi, Ahmed Mahmoud Rabie Elkhapery, Amir Emami, Luchuo Engelbert Bain, Ryenchindorj Erkhembayar, Farshid Etaee, Mohamad Ezati Asar, Adeniyi Francis Fagbamigbe, Shahab Falahi, Aida Fallahzadeh, Anwar Faraj, Emerito Jose A Faraon, Ali Fatehizadeh, Pietro Ferrara, Allegra Allegra Ferrari, Getahun Fetensa, Florian Fischer, Joanne Flavel, Masoud Foroutan, Peter Andras Gaal, Abhay Motiramji Gaidhane, Santosh Gaihre, Nasrin Galehdar, Alberto L Garcia-Basteiro, Tushar Garg, Mesfin Damtew Gebrehiwot, Mathewos Alemu Gebremichael, Yibeltal Yismaw Gela, Belete Negese Belete Gemeda, Bradford D Gessner, Melaku Getachew, Asmare Getie, Seyyed-Hadi Ghamari, Mohammad Ghasemi Nour, Ahmad Ghashghaee, Ali Gholamrezanezhad, Abdolmajid Gholizadeh, Rakesh Ghosh, Sherief Ghozy, Pouya Goleij, Mohamad Golitaleb, Giuseppe Gorini, Alessandra C Goulart, Girma Garedew Goyomsa, Habtamu Alganeh Guadie, Zewdie Gudisa, Rashid Abdi Guled, Sapna Gupta, Veer Bala Gupta, Vivek Kumar Gupta, Alemu Guta, Parham Habibzadeh, Arvin Haj-Mirzaian, Rabih Halwani, Samer Hamidi, Md Abdul Hannan, Mehdi Harorani, Ahmed I Hasaballah, Hamidreza Hasani, Abbas M Hassan, Shokoufeh Hassani, Hossein Hassanian-Moghaddam, Hadi Hassankhani, Khezar Hayat, Behzad Heibati, Mohammad Heidari, Demisu Zenbaba Heyi, Kamal Hezam, Ramesh Holla, Sung Hwi Hong, Nobuyuki Horita, Mohammad-Salar Hosseini, Mehdi Hosseinzadeh, Mihaela Hostiuc, Mowafa Househ, Soodabeh Hoveidamanesh, Junjie Huang, Nawfal R Hussein, Ivo Iavicoli, Segun Emmanuel Ibitoye, Kevin S Ikuta, Olayinka Stephen Ilesanmi, Irena M Ilic, Milena D Ilic, Mustapha Immurana, Nahlah Elkudssiah Ismail, Masao Iwagami, Jalil Jaafari, Elham Jamshidi, Sung-In Jang, Amirreza Javadi Mamaghani, Tahereh Javaheri, Fatemeh Javanmardi, Javad Javidnia, Sathish Kumar Jayapal, Umesh Jayarajah, Shubha Jayaram, Alelign Tasew Jema, Wonjeong Jeong, Jost B Jonas, Nitin Joseph, Farahnaz Joukar, Jacek Jerzy Jozwiak, Vaishali K, Zubair Kabir, Salah Eddine Oussama Kacimi, Vidya Kadashetti, Laleh R Kalankesh, Rohollah Kalhor, Ashwin Kamath, Bhushan Dattatray Kamble, Himal Kandel, Tesfaye K Kanko, Ibraheem M Karaye, André Karch, Samad Karkhah, Bekalu Getnet Kassa, Patrick DMC Katoto, Harkiran Kaur, Rimple Jeet Kaur, Leila Keikavoosi-Arani, Mohammad Keykhaei, Yousef Saleh Khader, Himanshu Khajuria, Ejaz Ahmad Khan, Gulfaraz Khan, Imteyaz A Khan, Maseer Khan, Md Nuruzzaman Khan, Moien AB Khan, Yusra H Khan, Moawiah Mohammad Khatatbeh, Mina Khosravifar, Jagdish Khubchandani, Min Seo Kim, Ruth W Kimokoti, Adnan Kisa, Sezer Kisa, Niranjan Kissoon, Luke D Knibbs, Sonali Kochhar, Farzad Kompani, Hamid Reza Koohestani, Vladimir Andreevich Korshunov, Soewarta Kosen, Parvaiz A Koul, Ai Koyanagi, Kewal Krishan, Barthelemy Kuate Defo, G Anil Kumar, Om P Kurmi, Ambily Kuttikkattu, Dharmesh Kumar Lal, Judit Lám, Iván Landires, Caterina Ledda, Sang-woong Lee, Miriam Levi, Sonia Lewycka, Gang Liu, Wei Liu, Rakesh Lodha, László Lorenzovici, Mojgan Lotfi, Joana A Loureiro, Farzan Madadizadeh, Ata Mahmoodpoor, Razzagh Mahmoudi, Marzieh Mahmoudimanesh, Jamal Majidpoor, Alaa Makki, Elaheh Malakan Rad, Ahmad Azam Malik, Tauqeer Hussain Mallhi, Yosef Manla, Clara N Matei, Alexander G Mathioudakis, Richard James Maude, Entezar Mehrabi Nasab, Addisu Melese, Ziad A Memish, Oliver Mendoza-Cano, Alexios-Fotios A Mentis, Tuomo J Meretoja, Mehari Woldemariam Merid, Tomislav Mestrovic, Ana Carolina Micheletti Gomide Nogueira de Sá, Gelana Fekadu Worku Mijena, Le Huu Nhat Minh, Shabir Ahmad Mir, Reza Mirfakhraie, Seyyedmohammadsadeq Mirmoeeni, Agha Zeeshan Mirza, Moonis Mirza, Mohammad Mirza-Aghazadeh-Attari, Abay Sisay Misganaw, Awoke Temesgen Misganaw, Esmaeil Mohammadi, Mokhtar Mohammadi, Arif Mohammed, Shafiu Mohammed, Syam Mohan, Mohammad Mohseni, Nagabhishek Moka, Ali H Mokdad, Sara Momtazmanesh, Lorenzo Monasta, Md Moniruzzaman, Fateme Montazeri, Catrin E Moore, Abdolvahab Moradi, Lidia Morawska, Jonathan F Mosser, Ebrahim Mostafavi, Majid Motaghinejad, Haleh Mousavi Isfahani, Seyed Ali Mousavi-Aghdas, Sumaira Mubarik, Efrén Murillo-Zamora, Ghulam Mustafa, Sanjeev Nair, Tapas Sadasivan Nair, Houshang Najafi, Atta Abbas Naqvi, Sreenivas Narasimha Swamy, Zuhair S Natto, Biswa Prakash Nayak, Seyed Aria Nejadghaderi, Huy Van Nguyen Nguyen, Robina Khan Niazi, Antonio Tolentino Nogueira de Sá, Hasti Nouraei, Ali Nowroozi, Virginia Nuñez-Samudio, Chimezie Igwegbe Nzoputam, Ogochukwu Janet Nzoputam, Bogdan Oancea, Chimedsuren Ochir, Oluwakemi Ololade Odukoya, Hassan Okati-Aliabad, Akinkunmi Paul Okekunle, Osaretin Christabel Okonji, Andrew T Olagunju, Isaac Iyinoluwa Olufadewa, Ahmed Omar Bali, Emad Omer, Eyal Oren, Erika Ota, Nikita Otstavnov, Abderrahim Oulhaj, Mahesh P A, Jagadish Rao Padubidri, Keyvan Pakshir, Reza Pakzad, Tamás Palicz, Anamika Pandey, Suman Pant, Shahina Pardhan, Eun-Cheol Park, Eun-Kee Park, Fatemeh Pashazadeh Kan, Rajan Paudel, Shrikant Pawar, Minjin Peng, Gavin Pereira, Simone Perna, Navaraj Perumalsamy, Ionela-Roxana Petcu, David M Pigott, Zahra Zahid Piracha, Vivek Podder, Roman V Polibin, Maarten J Postma, Hamid Pourasghari, Naeimeh Pourtaheri, Mirza Muhammad Fahd Qadir, Mathieu Raad, Mohammad Rabiee, Navid Rabiee, Saber Raeghi, Alireza Rafiei, Fakher Rahim, Mehran Rahimi, Vafa Rahimi-Movaghar, Azizur Rahman, Md Obaidur Rahman, Mosiur Rahman, Muhammad Aziz Rahman, Amir Masoud Rahmani, Vahid Rahmanian, Pradhum Ram, Kiana Ramezanzadeh, Juwel Rana, Priyanga Ranasinghe, Usha Rani, Sowmya J Rao, Sina Rashedi, Mohammad-Mahdi Rashidi, Azad Rasul, Zubair Ahmed Ratan, David Laith Rawaf, Salman Rawaf, Reza Rawassizadeh, Mohammad Sadegh Razeghinia, Elrashdy Moustafa Mohamed Redwan, Marissa B Reitsma, Andre M N Renzaho, Mohsen Rezaeian, Abanoub Riad, Reza Rikhtegar, Jefferson Antonio Buendia Rodriguez, Emma L B Rogowski, Luca Ronfani, Kristina E Rudd, Basema Saddik, Erfan Sadeghi, Umar Saeed, Azam Safary, Sher Zaman Safi, Maryam Sahebazzamani, Amirhossein Sahebkar, Sateesh Sakhamuri, Sana Salehi, Muhammad Salman, Hossein Samadi Kafil, Abdallah M Samy, Milena M Santric-Milicevic, Bruno Piassi Sao Jose, Maryam Sarkhosh, Brijesh Sathian, Monika Sawhney, Ganesh Kumar Saya, Abdul-Aziz Seidu, Allen Seylani, Amira A Shaheen, Masood Ali Shaikh, Elaheh Shaker, Hina Shamshad, Mequannent Melaku Sharew, Asaad Sharhani, Azam Sharifi, Purva Sharma, Ali Sheidaei, Suchitra M Shenoy, Jeevan K Shetty, Damtew Solomon Shiferaw, Mika Shigematsu, Jae Il Shin, Hesamaddin Shirzad-Aski, K M Shivakumar, Siddharudha Shivalli, Parnian Shobeiri, Wudneh Simegn, Colin R Simpson, Harpreet Singh, Jasvinder A Singh, Paramdeep Singh, Samarjeet Singh Siwal, Valentin Yurievich Skryabin, Anna Aleksandrovna Skryabina, Mohammad Sadegh Soltani-Zangbar, Suhang Song, Yimeng Song, Prashant Sood, Chandrashekhar T Sreeramareddy, Paschalis Steiropoulos, Muhammad Suleman, Seyed-Amir Tabatabaeizadeh, Alireza Tahamtan, Majid Taheri, Moslem Taheri Soodejani, Elahe Taki, Iman M Talaat, Mircea Tampa, Sarmila Tandukar, Nathan Y Tat, Vivian Y Tat, Yibekal Manaye Tefera, Gebremaryam Temesgen, Mohamad-Hani Temsah, Azene Tesfaye, Degefa Gomora Tesfaye, Belay Tessema, Rekha Thapar, Jansje Henny Vera Ticoalu, Amir Tiyuri, Imad I Tleyjeh, Munkhsaikhan Togtmol, Marcos Roberto Tovani-Palone, Derara Girma Tufa, Irfan Ullah, Era Upadhyay, Sahel Valadan Tahbaz, Pascual R Valdez, Rohollah Valizadeh, Constantine Vardavas, Tommi Juhani Vasankari, Bay Vo, Linh Gia Vu, Birhanu Wagaye, Yasir Waheed, Yu Wang, Abdul Waris, T Eoin West, Nuwan Darshana Wickramasinghe, Xiaoyue Xu, Sajad Yaghoubi, Gahin Abdulraheem Tayib Yahya, Seyed Hossein Yahyazadeh Jabbari, Dong Keon Yon, Naohiro Yonemoto, Burhan Abdullah Zaman, Alireza Zandifar, Moein Zangiabadian, Heather J Zar, Iman Zare, Zahra Zareshahrabadi, Armin Zarrintan, Mikhail Sergeevich Zastrozhin, Wu Zeng, Mengxi Zhang, Zhi-Jiang Zhang, Chenwen Zhong, Mohammad Zoladl, Alimuddin Zumla, Stephen S Lim, Theo Vos, Mohsen Naghavi, Michael Brauer, Simon I Hay, Christopher J L Murray

## Abstract

**Background:**

The global burden of lower respiratory infections (LRIs) and corresponding risk factors in children older than 5 years and adults has not been studied as comprehensively as it has been in children younger than 5 years. We assessed the burden and trends of LRIs and risk factors across all age groups by sex, for 204 countries and territories.

**Methods:**

In this analysis of data for the Global Burden of Diseases, Injuries, and Risk Factors Study (GBD) 2019, we used clinician-diagnosed pneumonia or bronchiolitis as our case definition for LRIs. We included International Classification of Diseases 9th edition codes 079.6, 466–469, 470.0, 480–482.8, 483.0–483.9, 484.1–484.2, 484.6–484.7, and 487–489 and International Classification of Diseases 10th edition codes A48.1, A70, B97.4–B97.6, J09–J15.8, J16–J16.9, J20–J21.9, J91.0, P23.0–P23.4, and U04–U04.9. We used the Cause of Death Ensemble modelling strategy to analyse 23 109 site-years of vital registration data, 825 site-years of sample vital registration data, 1766 site-years of verbal autopsy data, and 681 site-years of mortality surveillance data. We used DisMod-MR 2.1, a Bayesian meta-regression tool, to analyse age–sex-specific incidence and prevalence data identified via systematic reviews of the literature, population-based survey data, and claims and inpatient data. Additionally, we estimated age–sex-specific LRI mortality that is attributable to the independent effects of 14 risk factors.

**Findings:**

Globally, in 2019, we estimated that there were 257 million (95% uncertainty interval [UI] 240–275) LRI incident episodes in males and 232 million (217–248) in females. In the same year, LRIs accounted for 1·30 million (95% UI 1·18–1·42) male deaths and 1·20 million (1·07–1·33) female deaths. Age-standardised incidence and mortality rates were 1·17 times (95% UI 1·16–1·18) and 1·31 times (95% UI 1·23–1·41) greater in males than in females in 2019. Between 1990 and 2019, LRI incidence and mortality rates declined at different rates across age groups and an increase in LRI episodes and deaths was estimated among all adult age groups, with males aged 70 years and older having the highest increase in LRI episodes (126·0% [95% UI 121·4–131·1]) and deaths (100·0% [83·4–115·9]). During the same period, LRI episodes and deaths in children younger than 15 years were estimated to have decreased, and the greatest decline was observed for LRI deaths in males younger than 5 years (–70·7% [–77·2 to –61·8]). The leading risk factors for LRI mortality varied across age groups and sex. More than half of global LRI deaths in children younger than 5 years were attributable to child wasting (population attributable fraction [PAF] 53·0% [95% UI 37·7–61·8] in males and 56·4% [40·7–65·1] in females), and more than a quarter of LRI deaths among those aged 5–14 years were attributable to household air pollution (PAF 26·0% [95% UI 16·6–35·5] for males and PAF 25·8% [16·3–35·4] for females). PAFs of male LRI deaths attributed to smoking were 20·4% (95% UI 15·4–25·2) in those aged 15–49 years, 30·5% (24·1–36·9) in those aged 50–69 years, and 21·9% (16·8–27·3) in those aged 70 years and older. PAFs of female LRI deaths attributed to household air pollution were 21·1% (95% UI 14·5–27·9) in those aged 15–49 years and 18·2% (12·5–24·5) in those aged 50–69 years. For females aged 70 years and older, the leading risk factor, ambient particulate matter, was responsible for 11·7% (95% UI 8·2–15·8) of LRI deaths.

**Interpretation:**

The patterns and progress in reducing the burden of LRIs and key risk factors for mortality varied across age groups and sexes. The progress seen in children younger than 5 years was clearly a result of targeted interventions, such as vaccination and reduction of exposure to risk factors. Similar interventions for other age groups could contribute to the achievement of multiple Sustainable Development Goals targets, including promoting wellbeing at all ages and reducing health inequalities. Interventions, including addressing risk factors such as child wasting, smoking, ambient particulate matter pollution, and household air pollution, would prevent deaths and reduce health disparities.

**Funding:**

Bill & Melinda Gates Foundation.

**Research in context**

**Evidence before this study**

The burden of lower respiratory infections (LRI) among children younger than 5 years has been studied extensively by several groups, including the WHO Maternal and Child Epidemiology Estimation group and the Global Burden of Diseases, Injuries, and Risk Factors Study (GBD). We searched PubMed for the terms (“lower respiratory infection*”OR “LRI”)AND (“burden” OR “estimates”) AND (“age” OR “sex” OR “gender”) AND (“differenc*” OR “discrepan*” OR “disparit*”), with no language restrictions, for publications from Jan 1, 1980, to July 22, 2022. Our search identified 21 studies that reported population-based LRI morbidity and mortality estimates. Of these studies, 15 focused on either a single location or a subset of countries or regions, and six studies reported the LRI estimates at the global level. None of those studies reported the burden of LRIs attributable to risk factors for people older than 5 years by age and sex. We also did not find any studies reporting risk-deleted LRI mortality estimates. GBD 2017 estimated 2·56 million (95% uncertainty interval [UI] 2·44–2·66) LRI deaths among all ages and 0·80 million (0·75–0·87) LRI deaths in children younger than 5 years in 2017. The GBD 2017 LRI paper evaluated the risk factors and interventions that have affected the burden of LRIs among children younger than 5 years in 195 countries and territories.

**Added value of this study**

GBD 2019 included new data sources on LRI mortality and morbidity and used an enhanced standardised approach to adjust data from different sources (using different case definitions or measurement methods) to improve the comparability of data. We assessed the LRI burden for all age groups by sex for 204 countries and territories. We also assessed, for the first time, the burden of LRIs attributable to risk factors for children aged 5–14 years, as well as different adult age groups. Lastly, for the first time, we provided the risk-deleted mortality estimates that represent the LRI mortality rates that would have been observed if the combined effects of all evaluated risk factors were removed.

**Implications of all the available evidence**

Our study provides a comprehensive assessment of the LRI burden and risk factors across different age groups by sex. We identify the regions, countries, and age–sex groups with the highest LRI incidence and mortality to inform targeted interventions. By analysing the LRI burden by time, and identifying the leading risk factors by age groups separately for males and females, we provide insight into policy planning and resource prioritisation for addressing the uneven progress in reducing the LRI burden.

## Introduction

Lower respiratory infections (LRIs), mainly caused by bacteria such as *Streptococcus pneumoniae* and *Haemophilus influenzae* type b and viruses such as influenza and respiratory syncytial virus, are a leading cause of death globally, killing more than 2 million people every year.^1^ LRIs are also the leading underlying cause of sepsis, which is a major cause of health loss and death worldwide.^2^ Global initiatives to tackle LRIs, such as the Global Action Plan for the Prevention and Control of Pneumonia and Diarrhoea,^3^ the Stop Pneumonia Initiative,^4^ and the Integrated Management of Childhood Illness initiative,^5^ are targeted at children younger than 5 years. Current literature on the burden of LRIs also focuses primarily on children younger than 5 years; less attention is paid to the LRI burden among children older than 5 years and adults. Evidence indicates that males are more susceptible to LRIs than females, possibly due to factors such as differences in immune response to infection and behavioural factors such as smoking.^6^ Understanding the current burden and trends of LRIs across all age groups by sex is essential for identifying areas of intervention.

Although measuring the burden of LRIs is a crucial input in policy decision making, the assessment of modifiable risk factors for LRIs can inform preventive interventions. With the ageing of populations, it is increasingly important to assess LRI risk factors, especially those for which exposure is not declining, such as ambient particulate matter air pollution, and compare them to risk factors for which exposure is decreasing, such as household air pollution.^7^ Understanding the changing LRI burden attributable to various risk factors across the entire age spectrum can assist in identifying priorities for targeted interventions. To our knowledge, the global burden of LRIs attributable to risk factors for age groups other than those younger than 5 years has not been comprehensively studied. The objective of this study is to assess the burden and trends of LRIs and risk factors across all age groups by sex for 204 countries and territories. This manuscript was produced as part of the Global Burden of Diseases, Injuries, and Risk Factors Study (GBD) Collaborator Network and in accordance with the GBD Protocol.

## Methods

### Overview

Detailed methods for GBD 2019 have been published elsewhere.^1^, ^7^ Here, we describe the methods and estimation strategies for LRIs and risk factors. In compliance with the Guidelines for Accurate and Transparent Health Estimates Reporting (GATHER), input data sources and code for each step of the estimation process are available on the Global Health Data Exchange.

### Case definition

We used clinician-diagnosed pneumonia or bronchiolitis as our case definition for LRIs. We included International Classification of Diseases 9th edition codes 079.6, 466–469, 470.0, 480–482.8, 483.0–483.9, 484.1–484.2, 484.6–484.7, and 487–489, and International Classification of Diseases 10th edition codes A48.1, A70, B97.4–B97.6, J09–J15.8, J16–J16.9, J20–J21.9, J91.0, P23.0–P23.4, and U04–U04.9 (appendix 1 pp 81–84).

### LRI mortality

The GBD Cause of Death database collates all available data from vital registration systems, surveillance systems, and verbal autopsy studies. Input data for LRI mortality estimation included 23 109 site-years (the number of years for which data are available for a particular location) of vital registration data, 825 site-years of sample vital registration data (ie, data covering a sample of the population), 1766 site-years of verbal autopsy data, and 681 site-years of mortality surveillance data. Country-specific data sources and citations are available on the Global Health Data Exchange. Vital registration data were adjusted for completeness and garbage coding.^1^, ^8^ Data before and after garbage code redistribution are available in the online data visualisation tool.

We used the Cause of Death Ensemble modelling (CODEm) strategy^1^, ^9^ to generate LRI mortality estimates by location, year, age, and sex. CODEm assesses a vast array of sub-models with varying combinations of predictive covariates (eg, undernutrition and air pollution) that are run through four model categories (ie, mixed-effects regression models and spatiotemporal Gaussian process regression models for cause fractions and mortality rates; appendix 1 pp 13–14). Sub-models are evaluated using out-of-sample predictive validity and combined into an ensemble with the best predictive performance.

### LRI morbidity

To estimate age–sex-specific incidence and prevalence of LRIs, we used data identified via systematic reviews of the literature. Additionally, we used population-based survey data, claims data, and inpatient data to estimate incidence and prevalence (appendix 1 pp 6–10. For GBD 2019, we used an enhanced standardised approach, compared with previous GBD iterations, to adjust definitions in data sources that did not use our reference case definition to be comparable with our reference case definition (ie, clinician-diagnosed pneumonia or bronchiolitis). To do so, we first computed the ratio of the data based on alternative case definitions to the data based on the reference case definition, on the basis of all available data matched by location, year, age, and sex. We then ran a meta-regression to pool the ratios and used the pooled ratio to adjust the data based on alternative case definitions to the level of the data based on the reference case definition (appendix 1 pp 8–10).

Our inclusion criteria for scientific literature included a study duration of at least 1 year to avoid bias in the seasonal timing of LRIs and a sample size of at least 100 people (the sample size threshold was chosen arbitrarily). Survey data were adjusted for seasonality by fitting a generalised additive mixed-effects model with a forced periodicity for each GBD region, accounting for the year of the survey and the case definition used. The percentage difference between the monthly model-fit LRI prevalence and the corresponding regional mean LRI prevalence was computed to adjust survey data by month and geography. The mean duration of LRIs was 7·79 days (uncertainty interval [UI] 6·20–9·64]); this was determined on the basis of a systematic review and meta-analysis,^10^ and was used to convert incidence data to prevalence. We modelled these data together with LRI mortality estimates using DisMod-MR 2.1,^1^, ^11^ a Bayesian meta-regression tool that imposes coherence between data for different parameters, to produce final incidence and prevalence estimates. Details on the preparation of data sources and the modelling in DisMod can be found in appendix 1 (pp 6–11).

### Risk factors

Detailed methods for GBD risk factor estimation have been published elsewhere.^7^ In summary, we first selected risk–outcome pairs (eg, LRIs attributable to smoking) on the basis of evidence of a convincing or probable causal relationship between the risk and the outcome. A full list of LRI risk factors and the mechanism through which each risk factor could cause LRIs can be found in appendix 1 (pp 15–17). The population attributable fractions (PAFs) of risk factors were quantified by estimating the risk factor exposure distributions and the relative risk of the association between each risk factor and the outcome, and determining the theoretical minimum-risk exposure level. The PAF is the fraction of LRI mortality that would have been reduced if the exposure to the risk factor had been at the theoretical minimum-risk exposure level. The attributable burden was computed by multiplying the location–year–age–sex-specific PAFs of risk factors by corresponding LRI deaths. We also calculated risk-deleted mortality rates to represent the LRI mortality rate that would have been observed had the risk factors been set to their corresponding theoretical minimum-risk exposure levels. Full details of the methods used for estimating each of the 14 LRI risk factors are provided in appendix 1 (pp 18–79).

### Uncertainty intervals and age-standardisation

We computed 95% UIs based on 1000 draws from the posterior distribution of each stage in the estimation process using the 2·5th and 97·5th percentiles of the 1000 ordered values.

We used the GBD world population age standard^12^ to calculate age-standardised LRI incidence and mortality rates.

### Role of the funding source

The funder of the study had no role in study design, data collection, data analysis, data interpretation, or writing of the report.

## Results

**Figure 1:**
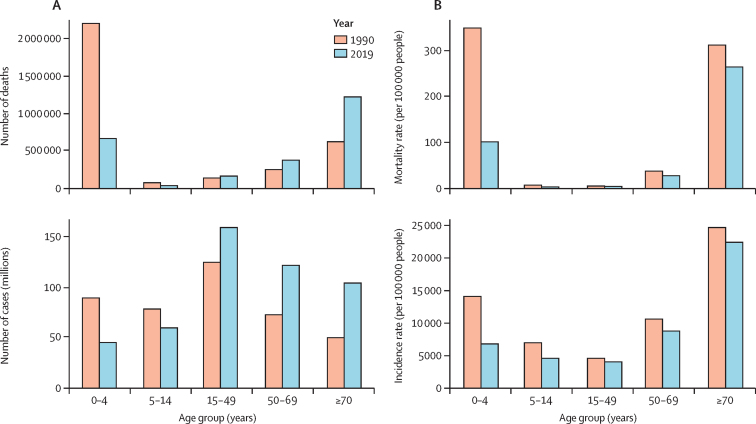
Incidence and mortality counts (A) and rates (B) due to lower respiratory infections for both sexes combined in 1990 and 2019, by age group

On a global scale, in 2019, the total number of LRI incident episodes was 257 million (95% UI 240–275) for males and 232 million (217–248) for females, reflecting an increase of 20·0% (95% UI 15·8–24·5) for males and 15·8% (11·9–19·7) for females since 1990 (appendix 2 pp 3, 5). The age-standardised incidence rate was 1·17 (95% UI 1·16–1·18) times greater in males than in females in 2019. When looking at specific age–sex groups, we estimated that there was a decrease in LRI episodes between 1990 and 2019 in children younger than 15 years and an increase in this period in all adult age groups (figure 1; appendix 2 pp 3, 34). Among children, the decrease varied from 20·8% (95% UI 16·2–25·6) among males aged 5–14 years to 49·9% (48·5–51·6) among females younger than 5 years. Among adult age groups, the increase varied from 26·1% (23·2–29·1) for females aged 15–49 years to 126·0% (121·4–131·1) for males aged 70 years and older during the same period (appendix 2 p 34).

**Figure 2:**
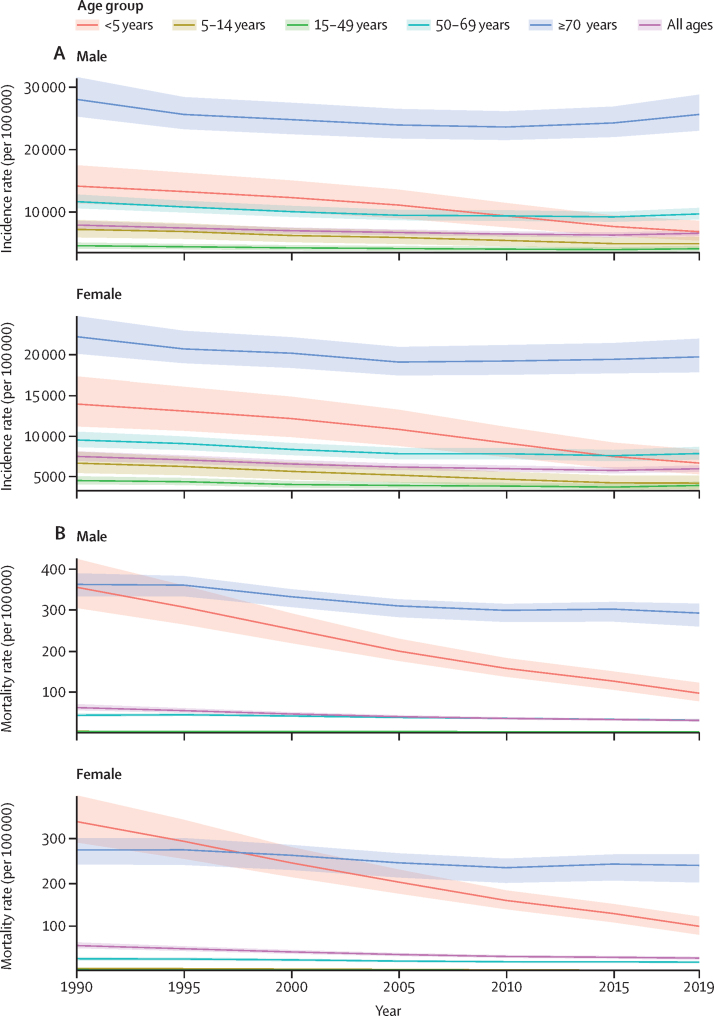
Global time trend of lower respiratory infection incidence rates (A) and mortality rates (B) by age and sex, 1990–2019

Between 1990 and 2019, children younger than 5 years saw the greatest improvement, with a decrease in incidence rate per 100 000 population of 51·7% (95% UI 50·0–53·5) for males and 52·1% (50·7–53·7) for females. Other age groups did not show similar improvements (figure 2; appendix 2 pp 34–37). We estimated only an 8·6% (6·6–10·5) decrease in incidence rate for males aged 70 years and older and only an 11·2% (9·2–13·0) decrease in incidence rate for females aged 70 years and older between 1990 and 2019.

In 2019, we estimated that individuals aged 15–49 years had the lowest global incidence rate of LRI episodes per 100 000 population among all age groups: 4128·1 (95% UI 3726·8–4583·5) for males and 3944·6 episodes (3541·5–4421·4) for females (Figure 1, Figure 2; appendix pp 34–37). Individuals aged 70 years and older, on the other hand, had the highest incidence rate per 100 000 population of all age groups: 25 786·6 (23 182·5–28 975·4) for males and 19 819·9 (17 921·3–22 072·6) for females. Of all super-regions, South Asia had the highest incidence rate per 100 000 population among both males aged 70 years and older (48 185·3 [95% UI 42 327·6–56 191·8]) and females aged 70 years and older (38 852·6 [34 264·3–44 606·1]; appendix 2 pp 34–37).

Globally, in terms of absolute numbers, LRIs accounted for 1·30 million (95% UI 1·18–1·42) deaths in 2019 among males and 1·20 million (1·07–1·33) deaths among females (appendix 2 p 38). The age-standardised mortality rate was 1·31 (95% UI 1·23–1·41) times greater in males than in females in 2019. We estimated an increase in LRI deaths among all adult age groups between 1990 and 2019 (figure 1; appendix 2 p 4), with males aged 70 years and older having the highest increase in deaths (100·0% [95% UI 83·4–115·9]; table). In high-income countries, we estimated a 70·7% (95% UI 58·3–77·9) increase in death counts for males aged 70 years and older and a 54·3% (39·7–63·0) increase for females aged 70 years and older (table). This increase in the number of deaths between 1990 and 2019 is visible regardless of age, with 20 countries showing an increase of more than 100% in death counts attributable to LRIs for males and 19 countries for females (appendix 2 pp 38–67).

**Table:** Lower respiratory infection deaths and mortality rates in 2019 and the percentage change in deaths and mortality rates between 1990 and 2019 by age, sex, and GBD super-region

	**Male**				**Female**			
	2019		Percentage change from 1990 to 2019		2019		Percentage change from 1990 to 2019	
	Number of deaths*	Mortality rate (per 100 000 people)	Number of deaths	Mortality rate (per 100 000 people)	Number of deaths*	Mortality rate (per 100 000 people)	Number of deaths	Mortality rate (per 100 000 people)
**Global**								
0–4 years	341 000 (275 000 to 428 000)	99·7 (80·2 to 124·9)	−70·7% (−77·2 to −61·8)	−72·1% (−78·4 to −63·7)	331 000 (270 000 to 401 000)	103·2 (84·2 to 125·0)	−68·4% (−75·9 to −60·0)	−69·7% (−76·9 to −61·7)
5–14 years	21 800 (18 500 to 25 900)	3·3 (2·8 to 3·9)	−45·0% (−54·2 to −30·9)	−52·8% (−60·6 to −40·6)	20 500 (16 700 to 24 700)	3·3 (2·7 to 3·9)	−50·2% (−59·8 to −39·7)	−56·6% (−65·0 to −47·5)
15–49 years	105 000 (95 000 to 116 000)	5·3 (4·8 to 5·8)	27·5% (14·1 to 43·7)	−11·9% (−21·2 to −0·7)	65 200 (56 500 to 74 400)	3·4 (2·9 to 3·8)	5·7% (−7·3 to 21·1)	−27·3% (−36·3 to −16·7)
50–69 years	228 000 (210 000 to 248 000)	33·8 (31·1 to 36·7)	47·7% (32·7 to 63·9)	−26·5% (−34·0 to −18·4)	153 000 (132 000 to 171 000)	21·8 (18·8 to 24·3)	50·1% (31·7 to 71·4)	−26·2% (−35·2 to −15·7)
≥70 years	600 000 (534 000 to 646 000)	294·8 (262·3 to 317·8)	100·0% (83·4 to 115·9)	−19·1% (−25·8 to −12·7)	628 000 (527 000 to 693 000)	241·2 (202·6 to 266·2)	90·5% (73·3 to 105·8)	−12·7% (−20·6 to −5·6)
**Central Europe, eastern Europe, and central Asia**								
0–4 years	7800 (6300 to 9700)	54·9 (44·3 to 68·1)	−78·1% (−82·8 to −72·4)	−72·0% (−78·0 to −64·8)	6200 (5100 to 7600)	46·5 (38·0 to 57·2)	−78·2% (−82·6 to −72·7)	−71·5% (−77·2 to −64·3)
5–14 years	800 (700 to 900)	3·0 (2·6 to 3·3)	−42·2% (−49·6 to −33·9)	−26·3% (−35·8 to −15·8)	700 (600 to 800)	2·6 (2·3 to 3·0)	−46·5% (−52·9 to −37·4)	−30·1% (−38·4 to −18·2)
15–49 years	13 100 (11 600 to 14 700)	13·1 (11·6 to 14·7)	83·4% (62·8 to 105·7)	87·4% (66·4 to 110·3)	4400 (3900 to 5100)	4·4 (3·9 to 5·1)	49·3% (30·0 to 71·9)	53·6% (33·8 to 76·8)
50–69 years	20 000 (17 700 to 22 400)	44·8 (39·6 to 50·2)	72·2% (52·9 to 92·2)	40·3% (24·5 to 56·6)	6600 (5900 to 7300)	12·0 (10·9 to 13·3)	38·3% (24·4 to 53·3)	19·3% (7·3 to 32·2)
≥70 years	18 700 (16 600 to 20 400)	147·3 (130·4 to 160·7)	69·8% (54·8 to 84·7)	0·8% (−8·1 to 9·7)	21 500 (18 700 to 23 600)	87·8 (76·3 to 96·2)	35·4% (24·6 to 45·9)	−2·5% (−10·2 to 5·1)
**High-income**								
0–4 years	900 (700 to 1000)	3·1 (2·6 to 3·6)	−74·5% (−78·8 to −69·6)	−72·5% (−77·1 to −67·2)	700 (600 to 800)	2·5 (2·1 to 2·8)	−75·3% (−79·2 to −71·4)	−73·4% (−77·6 to −69·2)
5–14 years	200 (200 to 200)	0·3 (0·3 to 0·3)	−64·4% (−67·6 to −58·9)	−63·3% (−66·5 to −57·5)	200 (200 to 200)	0·3 (0·3 to 0·3)	−62·2% (−66·1 to −53·7)	−61·0% (−65·0 to −52·2)
15–49 years	4500 (4300 to 4600)	1·8 (1·7 to 1·9)	−29·6% (−32·6 to −26·5)	−32·1% (−35·0 to −29·1)	2900 (2800 to 3000)	1·2 (1·2 to 1·3)	−15·1% (−19·0 to −11·2)	−17·7% (−21·5 to −13·9)
50–69 years	23 800 (22 900 to 24 700)	17·8 (17·2 to 18·5)	11·0% (6·8 to 15·5)	−30·8% (−33·4 to −27·9)	13 500 (12 900 to 13 900)	9·6 (9·3 to 10·0)	17·7% (13·2 to 22·2)	−22·4% (−25·4 to −19·4)
≥70 years	189 000 (164 000 to 202 000)	302·2 (263·3 to 323·4)	70·7% (58·3 to 77·9)	−21·9% (−27·5 to −18·5)	201 000 (160 000 to 223 000)	240·5 (191·9 to 266·7)	54·3% (39·7 to 63·0)	−12·2% (−20·5 to −7·2)
**Latin America and Caribbean**								
0–4 years	11 000 (8400 to 14 200)	44·9 (34·0 to 57·9)	−79·9% (−85·4 to −73·1)	−79·2% (−84·9 to −72·2)	8700 (6800 to 10900)	37·0 (28·8 to 46·4)	−81·4% (−86·4 to −75·6)	−80·5% (−85·7 to −74·4)
5–14 years	1000 (800 to 1200)	2·1 (1·7 to 2·4)	−60·3% (−67·4 to −53·1)	−61·7% (−68·5 to −54·7)	900 (800 to 1100)	2·0 (1·7 to 2·2)	−62·1% (−67·5 to −56·1)	−62·6% (−67·9 to −56·7)
15–49 years	10 200 (9200 to 11 300)	6·8 (6·1 to 7·5)	19·0% (7·6 to 32·1)	−24·3% (−31·6 to −15·9)	5800 (5100 to 6500)	3·7 (3·3 to 4·2)	5·1% (−7·9 to 19·0)	−32·5% (−40·8 to −23·5)
50–69 years	20 000 (18 100 to 22 100)	42·8 (38·8 to 47·2)	89·3% (72·4 to 110·8)	−25·1% (−31·8 to −16·6)	13 600 (12 400 to 14 900)	26·1 (23·7 to 28·6)	94·5% (75·6 to 114·8)	−27·0% (−34·1 to −19·3)
≥70 years	55 400 (47 700 to 60 800)	385·2 (331·5 to 422·7)	160·9% (139·7 to 183·4)	−8·6% (−16·1 to −0·8)	64 600 (53 500 to 71 600)	354·0 (293·4 to 392·7)	187·6% (161·9 to 210·2)	−7·2% (−15·5 to 0·1)
**North Africa and Middle East**								
0–4 years	15300 (11 800 to 19 500)	50·0 (38·6 to 63·6)	−80·3% (−86·6 to −72·9)	−82·5% (−88·1 to −75·9)	15 400 (12 000 to 19 100)	52·9 (41·3 to 65·9)	−80·1% (−85·6 to −73·6)	−82·2% (−87·1 to −76·3)
5–14 years	1800 (1400 to 2300)	3·0 (2·3 to 3·8)	−48·3% (−64·1 to −32·8)	−60·0% (−72·2 to −47·9)	1600 (1100 to 2100)	2·9 (2·0 to 3·7)	−52·0% (−63·9 to −37·4)	−62·5% (−71·7 to −51·0)
15–49 years	6500 (5400 to 7800)	3·7 (3·1 to 4·5)	68·0% (40·5 to 99·6)	−19·9% (−33·1 to −4·9)	4900 (3800 to 6000)	3·1 (2·4 to 3·8)	37·4% (11·3 to 65·5)	−31·5% (−44·6 to −17·6)
50–69 years	11 600 (9700 to 13 700)	28·2 (23·6 to 33·3)	89·7% (56·4 to 134·0)	−25·6% (−38·6 to −8·2)	7700 (5900 to 9400)	19·9 (15·3 to 24·4)	80·7% (46·1 to 144·3)	−28·4% (−42·1 to −3·2)
≥70 years	23 200 (20 100 to 26 700)	238·7 (206·3 to 274·0)	143·9% (111·4 to 195·5)	−8·2% (−20·4 to 11·2)	19 700 (16 500 to 23 100)	201·5 (168·1 to 236·0)	131·4% (92·0 to 204·4)	−9·7% (−25·1 to 18·8)
**South Asia**								
0–4 years	87 800 (69 700 to 111 000)	102·4 (81·3 to 129·1)	−76·1% (−82·3 to −67·2)	−76·6% (−82·7 to −67·9)	103 000 (82 700 to 126 000)	131·1 (105·1 to 160·7)	−69·9% (−78·2 to −60·3)	−70·2% (−78·4 to −60·6)
5–14 years	6600 (5000 to 8500)	3·6 (2·7 to 4·6)	−46·8% (−58·6 to −28·5)	−58·2% (−67·4 to −43·8)	7400 (5500 to 9400)	4·4 (3·3 to 5·5)	−58·4% (−68·1 to −46·6)	−67·3% (−74·9 to −58·0)
15–49 years	16 900 (13 800 to 20 700)	3·4 (2·8 to 4·2)	21·1% (−4·8 to 51·1)	−33·1% (−47·4 to −16·5)	15 000 (11 600 to 18 900)	3·1 (2·4 to 4·0)	7·1% (−18·5 to 37·4)	−42·8% (−56·5 to −26·7)
50–69 years	48 200 (38 600 to 58 300)	39·2 (31·4 to 47·5)	42·3% (10·4 to 79·0)	−33·9% (−48·7 to −16·8)	49 600 (38 000 to 62 000)	40·5 (31·1 to 50·6)	70·3% (26·4 to 126·0)	−29·9% (−47·9 to −6·9)
≥70 years	102 000 (83 800 to 122 000)	309·2 (253·6 to 368·1)	133·3% (84·1 to 187·3)	−20·9% (−37·6 to −2·6)	111 000 (86 700 to 138 000)	308·4 (240·1 to 382·4)	178·6% (107·1 to 271·0)	−17·4% (−38·6 to 10·0)
**Southeast Asia, east Asia, and Oceania**								
0–4 years	30 600 (25 800 to 35 800)	41·2 (34·7 to 48·2)	−90·2% (−92·5 to −87·6)	−87·6% (−90·4 to −84·3)	23 400 (19 900 to 27 200)	35·4 (30·0 to 41·1)	−91·6% (−93·4 to −89·3)	−89·1% (−91·4 to −86·1)
5–14 years	2200 (1900 to 2600)	1·6 (1·4 to 1·8)	−78·8% (−82·1 to −64·7)	−74·4% (−78·4 to −57·4)	1700 (1500 to 2000)	1·4 (1·2 to 1·6)	−79·1% (−82·6 to −70·0)	−73·4% (−77·8 to −61·7)
15–49 years	16 700 (14 800 to 19 000)	2·9 (2·6 to 3·3)	−20·2% (−33·4 to 4·5)	−33·5% (−44·4 to −12·9)	8400 (7200 to 10000)	1·5 (1·3 to 1·8)	−38·1% (−49·1 to −24·2)	−48·4% (−57·6 to −36·8)
50–69 years	47 900 (42 100 to 54 500)	19·4 (17·1 to 22·1)	42·2% (19·0 to 71·5)	−39·1% (−49·0 to −26·5)	25 000 (20 300 to 28 700)	10·0 (8·1 to 11·5)	10·7% (−6·7 to 32·0)	−54·9% (−62·0 to −46·2)
≥70 years	138 000 (122 000 to 154 000)	221·4 (195·1 to 246·0)	119·6% (89·6 to 162·2)	−23·1% (−33·6 to −8·2)	138 000 (112 000 to 158 000)	178·4 (144·8 to 203·4)	79·2% (55·9 to 110·8)	−33·5% (−42·2 to −21·8)
**Sub-Saharan Africa**								
0–4 years	188 000 (143 000 to 243 000)	224·1 (170·1 to 289·2)	−40·1% (−55·1 to −17·6)	−67·8% (−75·9 to −55·7)	173 000 (135 000 to 220 000)	211·5 (165·3 to 268·8)	−35·3% (−52·9 to −14·3)	−64·8% (−74·3 to −53·3)
5–14 years	9200 (7300 to 11 400)	6·3 (5·0 to 7·8)	2·4% (−21·6 to 42·8)	−52·7% (−63·8 to −34·1)	8000 (6100 to 10 100)	5·6 (4·2 to 7·0)	4·7% (−22·9 to 39·7)	−51·0% (−63·9 to −34·5)
15–49 years	36 800 (31 100 to 43 600)	14·6 (12·3 to 17·3)	73·2% (43·0 to 106·9)	−26·8% (−39·5 to −12·5)	23 700 (18 700 to 29 400)	8·9 (7·0 to 11·1)	27·8% (3·2 to 57·3)	−46·2% (−56·6 to −33·8)
50–69 years	56 700 (48 600 to 65 900)	139·6 (119·8 to 162·4)	52·3% (28·3 to 80·8)	−26·8% (−38·4 to −13·2)	37 500 (29 900 to 45 200)	84·2 (67·3 to 101·5)	62·6% (35·8 to 99·2)	−30·8% (−42·2 to −15·2)
≥70 years	73 100 (65 200 to 80 900)	850·8 (758·1 to 941·4)	79·7% (54·2 to 106·1)	−10·0% (−22·8 to 3·2)	71 500 (58 900 to 81 500)	672·1 (553·9 to 766·2)	101·5% (76·0 to 130·9)	−5·4% (−17·4 to 8·4)

95% uncertainty intervals are shown in parentheses. GBD=Global Burden of Diseases, Injuries, and Risk Factors Study.

* Six-digit numbers are reported to the nearest 1000 deaths and all other numbers are reported to the nearest 100 deaths.

Between 1990 and 2019, children younger than 5 years showed the greatest improvement in death rates for LRIs (Figure 1, Figure 2), with a decrease in mortality rate per 100 000 people of 72·1% (95% UI 63·7–78·4) for males and a decrease of 69·7% (61·7–76·9) for females (table). Despite this finding, in 2019, there were still 672 000 LRI deaths (95% UI 551 000–826 000) in children younger than 5 years for both sexes combined (table; figure 1). Mortality rates for individuals aged 70 years and older decreased at a much slower pace globally over the same period (figure 2B); we estimated a 19·1% (95% UI 12·7–25·8) decrease in mortality rate for males and a 12·7% (5·6–20·6) decrease in mortality rate for females (table).

In 2019, global mortality rates due to LRIs were highest in individuals aged 70 years and older; the mortality rate per 100 000 was 294·8 (95% UI 262·3–317·8) for males and 241·2 (202·6–266·2) for females (table; Figure 1, Figure 2). In contrast, children aged 5–14 years had the lowest mortality rates, with a mortality rate per 100 000 people of only 3·3 (2·8–3·9) for males and 3·3 (2·7–3·9) for females. We estimated that sub-Saharan Africa was the super-region with the highest mortality rate in individuals aged 70 years and older, with a mortality rate per 100 000 people of 850·8 (758·1–941·4) for males and 672·1 (553·9–766·2) for females (table).

Globally, in 2019, we estimated that 876 000 LRI deaths (95% UI 770 000–987 000) among males (PAF 67·6% [95% UI 62·9–72·1]) and 725 000 deaths (95% UI 629 000–826 000) among females (PAF 60·6% (95% UI 55·6–65·6) were attributable to all evaluated LRI risk factors (appendix 2 p 68). Globally, the number of LRI deaths attributable to all risk factors decreased by 41·4% (95% UI 32·0–49·3) for males and 44·5% (34·9–53·9) for females between 1990 and 2019. Children younger than 5 years had the greatest percentage decrease in number of deaths and mortality rate attributable to all risk factors between 1990 and 2019 (appendix 2 p 97). The greatest percentage increase in attributable deaths between 1990 and 2019 was estimated to be in males aged 70 years and older (66·6% [95% UI 50·4–82·8]). Global age-standardised attributable mortality rate per 100 000 population due to all risk factors in 2019 was 26·2 (95% UI 23·1–29·5) for males and 19·4 (16·8–22·2) for females. Between 1990 and 2019, this rate decreased by 56·1% (95% UI 50·0–60·9) for males and 59·1% (52·5–65·6) for females (appendix 2 p 68).

**Figure 3:**
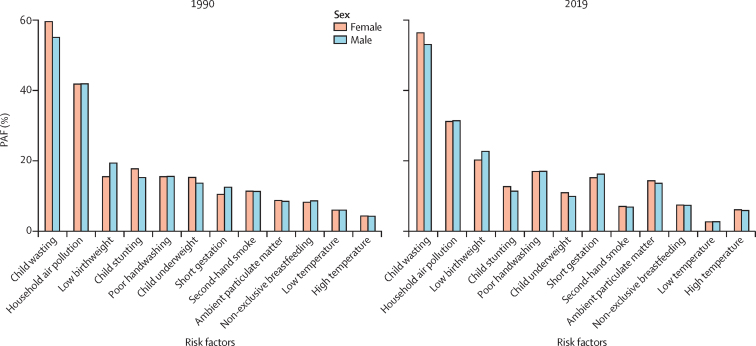
PAFs of lower respiratory infection deaths due to evaluated risk factors among males and females younger than 5 years in 1990 and 2019 Poor handwashing is defined as no access to a handwashing station with available soap and water. Non-exclusive breastfeeding is defined as the proportion of children under 6 months of age who are not exclusively breastfed. PAF=population attributable fraction.

In 2019, the leading risk factor for LRI mortality in children younger than 5 years was child wasting, in both males (PAF 53·0% [95% UI 37·7–61·8]) and females (56·4% [40·7–65·1]; figure 3; appendix p 117). Child wasting was also the largest risk factor in children younger than 5 years in 1990 and had decreased only slightly by 2019 (figure 3). For children younger than 5 years in 2019, the second largest PAF was for household air pollution; male and female children in this age group had near identical PAFs (31·4% [95% UI 21·5–41·5] *vs* 31·2% [21·3–41·5]; appendix p 117). Household air pollution was the second largest PAF in 1990 and had decreased substantially by 2019 (figure 3).

**Figure 4:**
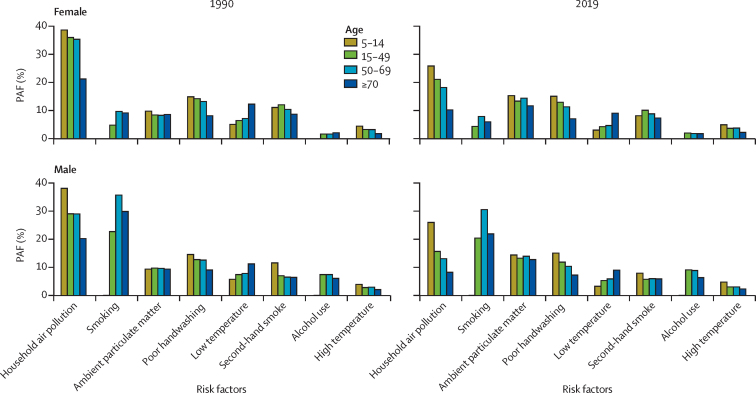
PAFs of lower respiratory infection deaths due to evaluated risk factors among males and females aged 5–14 years, 15–49 years, 50–69 years, and 70 years and older in 1990 and 2019 Poor handwashing is defined as no access to a handwashing station with available soap and water. Non-exclusive breastfeeding is defined as the proportion of children under 6 months of age who are not exclusively breastfed. PAF=population attributable fraction.

In 2019, the largest risk factor for children aged 5–14 years was household air pollution (PAF 26·0% [95% UI 16·6–35·5] for males and 25·8% [16·3–35·4] for females; figure 4; appendix p 134). For males aged 15–49 years, the risk factor with the highest PAF in 2019 was smoking (20·4% [15·4–25·2]); the risk factor with the lowest effect on the same group was high temperature (3·0% [1·3–6·9]; appendix p 147). These findings differ from findings in males aged 15–49 years in 1990, for whom the largest risk factor was household air pollution (29·0% [20·0–37·9]; figure 4). In 2019, females aged 15–49 years had the highest PAF from household air pollution (21·1% [14·5–27·9]), and the lowest PAF from alcohol use (2·0% [0·6–3·4]; figure 4; appendix p 147).

Males aged 50–69 years in 2019 had the highest PAF from smoking (30·5% [95% UI 24·1–36·9]) and the lowest PAF from high temperature (3·0% [1·3–6·4]; figure 4; appendix p 160). Females in the same age group had the highest PAF from household air pollution (18·2% [12·5–24·5]) and the lowest from alcohol use (1·8% [0·5–3·1]; figure 4; appendix p 160). In individuals aged 70 and older, males in 2019 had the highest PAF from smoking (21·9% [16·8–27·3]) and the lowest PAF from high temperature (2·3% [1·1–4·8]; figure 4; appendix p 173). Females in this age group had the highest PAF from ambient particulate matter (11·7% [8·2–15·8]), and the lowest PAF from alcohol use (1·8% [0·5–3·2]; figure 4; appendix p 173). Differing from females in 2019, females in 1990 had the highest PAF from household air pollution (21·2% [15·4–27·6]), and the lowest PAF from high temperature (1·8% [0·7–6·2]; figure 4).

Ambient particulate matter tended to affect males and females similarly across all age ranges (figure 4). PAFs for low temperatures were higher for people aged 70 years and older than for the younger age ranges. In 2019, second-hand smoke produced differing patterns of effect between males and females: males aged 5–14 years had a higher PAF (7·9% [95% UI 4·5–11·5]) than other age categories, whereas females aged 15–49 years were estimated to have a slightly higher PAF from second-hand smoke (10·1% [5·8–14·4]) than other age categories. Alcohol-use PAFs were much higher in males across all age categories than in females. Lastly, high temperature PAFs were consistent across both sexes and tended to show a greater effect on individuals in younger age categories.

**Figure 5:**
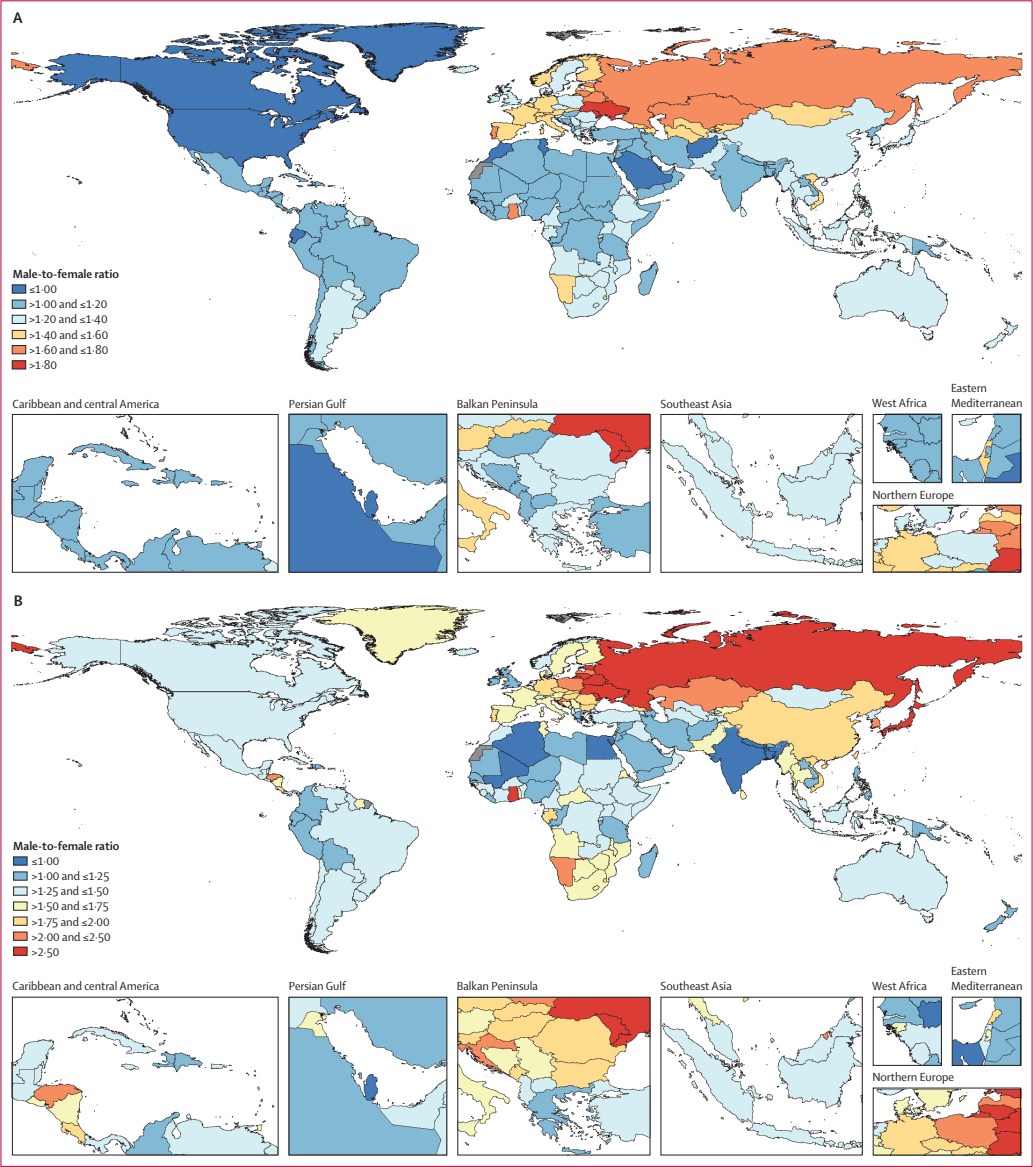
Male-to-female ratio of age-standardised lower respiratory infection incidence rates (A) and mortality rates (B), 2019

Mortality and incidence ratios were calculated between males and females across countries for the year 2019 (figure 5). Male-to-female ratios of age-standardised incidence rates were the highest in Ukraine and Moldova (figure 5A). Male-to-female ratios of age-standardised mortality rates were the highest in Russia, Belarus, Ukraine, Estonia, Lithuania, Japan, Ghana, and Moldova (figure 5B).

In 2019, the male-to-female ratio in global age-standardised mortality rates was 1·31 (95% UI 1·23–1·41). When all risk factors for LRIs were removed, the global age-standardised mortality rate per 100 000 population in 2019 in males was 13·5 (95% UI 11·4–15·7), and in females was 11·1 (9·2–12·7; appendix 2 p 102). Therefore, the ratio of risk-deleted mortality rates between males and females in 2019 was 1·22 (95% UI 1·12–1·36). Comparatively, in 1990, the male-to-female ratio in global age-standardised mortality rates was 1·24 (95% UI 1·15–1·33). The global risk-deleted mortality rates per 100 000 population were 15·6 (95% UI 13·0–18·3) for males and 13·4 (11·2–15·7) for females (appendix p 102). The risk-deleted mortality rate ratio between males and females in 1990 was 1·17 (95% UI 1·07–1·28). In 2019, the central Europe, eastern Europe, and central Asia region had the largest risk-deleted male-to-female ratio of all regions at 1·62 (95% UI 1·43–1·83). South Asia had the lowest male-to-female ratio at 0·88 (95% UI 0·67–1·14), where females had a higher risk-deleted mortality rate than males (appendix p 110).

## Discussion

Between 1990 and 2019, the greatest progress in reducing LRI incidence and mortality rates was observed in children younger than 5 years, indicating the success of initiatives targeting children in this age bracket. Much less progress has been made to reduce LRI incidence and mortality rates in adults, particularly in older age groups. Over the same period, in terms of absolute numbers, an increase in LRI incident episodes and deaths was estimated among all adult age groups due to population growth and aging. Globally, in 2019, age-standardised incidence rates were 1·2 times greater in males than in females and mortality rates were 1·3 times greater. Smoking was the leading risk factor for LRI mortality in adult males, responsible for about one-third of LRI deaths in those aged 50–69 years and one-fifth of LRI deaths in other age groups in 2019. PAFs of LRI deaths attributable to ambient particulate matter pollution have gone up for both males and females of all ages since 1990. On the other hand, PAFs of LRI deaths due to household air pollution have decreased among all age groups since 1990. PAFs of LRI deaths due to child wasting, stunting, and being underweight have also decreased among children younger than 5 years during the same period.

Despite the substantial progress made in children younger than 5 years, there were still 672 000 LRI deaths in this age group in 2019, and 93·5% (95% UI 90·4–95·7) of those deaths were attributable to preventable risk factors. Over the past two decades, global initiatives to combat wasting, the leading LRI risk factor, have focused mainly on treating wasting children, particularly in humanitarian situations.^13^ Although treatment coverage has steadily increased over time, only one-third of severely wasted children received treatment in 2019.^13^ This situation has been compounded by COVID-19 pandemic-related disruptions to nutrition and other fundamental services;^14^ the global prevalence of child wasting was estimated to increase by 14·3% during the first year of the pandemic.^15^ Given that undernutrition is the main risk factor not only for LRIs but also for other leading causes of death in children younger than 5 years such as diarrhoea and measles, long-term adverse implications are foreseeable unless the recommended actions (eg, protecting and facilitating access to healthy, nutritious, and affordable food and reactivation and scaling up of early detection and treatment services for child wasting^14^) are taken promptly.

In 2015, the era of the Millennium Development Goals (MDGs) ended and the global community unanimously adopted the Sustainable Development Goals (SDGs). Although substantial progress had been made towards the MDG goal of reducing under-5 mortality by two-thirds between 1990 and 2015,^16^ achieving the new SDG target of 25 or fewer deaths per 1000 livebirths by 2030 would require promoting child survival by accelerating the decline of the major causes of death in young children. LRIs, which were still the leading infectious cause of death among children younger than 5 years in 2019, are largely preventable through vaccination and addressing key risk factors.^10^, ^17^

In contrast to the progress seen in children younger than five years, little has been achieved in reducing the LRI burden among adults, indicating a need for initiatives that address LRI risk factors in the adult age groups. Ambient particulate matter pollution was a leading risk factor for LRI mortality in all adult age groups in 2019. Studies from 2020 have also suggested associations between elevated exposures to particulate matter with a diameter less than 2·5 μm (PM_2·5_) and higher COVID-19 cases and deaths.^18^, ^19^, ^20^ Contributors to global ambient particulate matter pollution include wildfires, biomass burning, sandstorms, chemical plants, and vehicle combustion sources.^21^, ^22^, ^23^, ^24^, ^25^ A study of how countries have followed the WHO ambient air quality guidelines found there were no air quality standards in 57 (34%) of the 170 countries examined.^26^ The same study also found that air quality standards for some pollutants, including PM_2·5_, were non-compliant with WHO guidelines in many countries.^26^

Our results showed that global LRI deaths attributable to household air pollution decreased among all age groups between 1990 and 2019; however, exposure to household air pollution was responsible for more than a quarter of LRI deaths among children younger than 5 years and children aged 5–14 years, and more than a fifth of LRI deaths among women aged 15–49 years, in 2019. Sub-Saharan Africa had the largest PAFs, and South Asia had the second largest PAFs, for household air pollution across all age groups in 2019. More than 890 million people do not have access to clean cooking fuels in sub-Saharan Africa.^27^ In India, the Pradhan Mantri Ujjwala Yojana, one of India's primary policies to provide households with liquid petroleum gas, a clean cooking fuel, was scheduled to be implemented in 102 cities and towns and related villages in 2019.^28^ An evaluation study done in a rural community in Odisha found that the majority of Pradhan Mantri Ujjwala Yojana recipients did not refill their liquid petroleum gas cylinders (ie, solid fuels were still being used for cooking), indicating the need for interventions to address challenges faced by rural households to ensure a complete transition from polluting to clean fuels.^29^

Consistent with previous studies,^6^ we found higher LRI incidence and mortality among males than females, especially among adults. Potential reasons for this difference include sex differences in the immune response to infection and behavioural factors such as smoking and alcohol use.^6^, ^30^ Females generally have a stronger immune system than males.^31^ Smoking is not only immunosuppressive but also causes changes such as ciliary dysfunction in the respiratory tract, leading to decreased pathogen clearance.^32^ The highest LRI mortality rates attributable to smoking among men were observed in countries in east Asia, southeast Asia, and eastern Europe. Despite a gradual decline in smoking prevalence in most of these countries, the declines were not sufficient to compensate for population growth, leading to a steady or growing number of smokers with time.^33^ In many countries worldwide, progress towards reducing smoking prevalence has stalled in the past decade.^7^, ^33^ The number of countries that have implemented at least one key intervention of the WHO Framework Convention on Tobacco Control has increased over time; however, only 62 countries had a complete ban on smoking in public and workplace settings, and only 23 countries provided comprehensive support for smokers seeking assistance in quitting smoking, as of 2018.^34^

Results showed that PAFs attributable to alcohol use were much higher in males than females across all adult age categories. Alcohol use increases the risk of microbe aspiration and weakens the host immune system.^35^ Although alcohol use is generally higher among men than women, it is increasing among women in different parts of the world, including some countries in sub-Saharan Africa.^36^, ^37^, ^38^ Increased government support and engagement are essential for adopting and enforcing effective alcohol policies in sub-Saharan Africa, which is a target region for alcohol companies to expand their market.^39^

We found that the male-to-female ratio in global age-standardised LRI mortality rates decreased from 1·31 to 1·22 after removing the combined effects of all evaluated risk factors. Despite the smaller ratio, males still had a higher mortality rate than females, suggesting that other factors such as genetics and hormones could have a role in differential regulation of the immune system and the greater risk of mortality among males than females.^31^

Our results suggest that reducing the LRI burden and targeting the key risk factors that are different across age–sex groups will help in achieving multiple SDG targets, including SDG 3 (ensuring healthy lives and promoting wellbeing for all ages), SDG 7 (affordable and clean energy), and SDG-10 (reducing inequalities).^40^ The remarkable progress made in children younger than 5 years was a result of the scale-up of proven interventions, including vaccination and reducing exposure to known risk factors.^10^ Similar interventions for other age groups could contribute to the achievement of the SDG targets. Pneumococcal conjugate vaccines have been shown to have a direct protective effect on young children and an indirect protective effect on unvaccinated adults.^41^ The global pneumococcal conjugate vaccine coverage (third dose) among young children was estimated to be 47·9% (95% UI 47·0–48·9) in 2019.^42^ The gap in childhood immunisation coverage has become wider as the COVID-19 pandemic disrupted routine immunisation services worldwide, indicating an urgent need for catch-up and expansion of immunisation.^43^, ^44^ Studies published since 2019 have shown that direct immunisation of older adults with PCV13 significantly reduced the disease burden.^45^, ^46^, ^47^ Immunisation of older adults, as well as addressing key leading risk factors such as child wasting, smoking, ambient particulate matter pollution, and household air pollution, could help reduce the burden of LRIs across all age groups. Additionally, supportive care, such as oxygen therapy, is a key part of the management of severe LRIs, and interventions to strengthen oxygen systems in low-resource settings could further help reduce LRI mortality.^48^

This study has several limitations. One of the key limitations is the availability of data. In the absence of data for a particular country, estimates were dependent on the regional patterns, covariates, and out-of-sample predictive validity assessment. The absence of data in a given country translated into wide intervals of uncertainty. Even in countries with data, delays in data reporting prevented their timely integration into the GBD estimation. The most recent years for which cause of death data were available were 2016 and 2017. We were able to validate our estimation method by comparing two sets of estimates produced for a particular year with and without using any data for that year. For example, GBD 2016 produced LRI mortality estimates for 2016 using data available up to 2013 and 2014; these estimates were compared with GBD 2019 estimates for the same year that were informed by empirical data for 2016. GBD 2016^49^ estimated a mortality rate per 100 000 population of 37·0 (95% UI 34·1–40·0) and GBD 2019 estimated 37·9 (32·5–40·8) for all ages and both sexes combined for the high-income super-region in 2016. Although the estimates are not identical, they are sufficiently close enough to support the validity of our approach. In this study, we were unable to evaluate the contribution of individual causes to the LRI burden. We plan to do a comprehensive assessment of the burden attributable to various pathogens in our future GBD estimation. Additionally, we have not assessed the LRI burden attributable to some potentially important risk factors such as overcrowding and incomplete immunisation.^50^ Current risk–outcome pairs were included on the basis of the World Cancer Research Fund criteria for convincing or probable evidence. We could evaluate whether additional risk factors are eligible for inclusion in future GBD iterations. Lastly, our current estimates of risk-attributable burden are limited by the quality of the primary data underlying the analysis. For example, data on some risk factors such as smoking and second-hand smoke were self-reported. Studies have indicated that self-reported smoking prevalence data might be prone to underestimation depending on respondents’ perception of the social acceptance of smoking.^51^, ^52^ Second-hand smoke exposure data might also be prone to recall bias.^53^ Biomarker-based exposure assessment such as cotinine could help improve the accuracy of smoking and second-hand smoke data.^52^, ^53^

Although our results represent the LRI burden before the COVID-19 pandemic, the effect of the pandemic on LRIs needs to be investigated further. The pandemic was linked to a reduction of influenza and respiratory syncytial virus infections, probably as a result of mitigation measures, including mask wearing and social distancing.^54^, ^55^ With the relaxation of measures, some countries started to see a rebound in influenza and respiratory syncytial virus infections in late 2020.^56^, ^57^ As the data become more widely available, in future rounds of the GBD, we can quantify the indirect effects of the COVID-19 pandemic on the burden and causes of LRIs.

In conclusion, our results showed that despite an overall global decline in LRI incidence and mortality rates between 1990 and 2019, the pace of decline has been unequal across age groups. The observed progress in children younger than 5 years was clearly a result of targeted interventions, including improving vaccination and reducing exposure to risk factors. Similar interventions for other age groups could contribute to the achievement of multiple SDG targets, including promoting well-being at all ages and reducing health inequalities.

## Data sharing

To download the data used in these analyses, please visit the Global Health Data Exchange GBD 2019 website.

## Declaration of interests

V Abedi reports grants or contracts from Genentech/ROCHE Biotech company and the National Institutes of Health (NIH) (2R56HL116832-04) ending in 2021, outside the submitted work. S Afzal reports leadership or fiduciary roles in board, society, committee, or advocacy groups, paid or unpaid, as a member of the Corona Expert Advisory Group, a member of the Medical Microbiology and Infectious Diseases Society of Pakistan, and as secretary of the task force for integrated management of childhood illnesses, all outside the submitted work. E F Atia report grants or contracts from the NIH and National Heart, Lung, and Blood Institute (K23 HL129888) and participation on a data safety monitoring board for effectiveness of low-dose theophyline for biomass-associated chronic obstructive pulmonary disease study, all outside the submitted work. D Bryazka reports grants or contracts from Bloomberg outside the submitted work. B D Gessner is an employee of Pfizer Vaccines and holds stock options in Pfizer. J Jozwiak reports personal fees for lectures, presentations, speakers bureaus, manuscript writing, or educational events from Teva, Amgen, Synexus, Boehringer Ingelheim, Zentiva, and Sanofi, all outside the submitted work. K Krishan reports non-financial support from the UGC Centre of Advanced Study, CAS II, Department of Anthropology, Panjab University, Chandigarh, India, all outside the submitted work. J A Loureiro reports support for the present manuscript from Scientific Employment Stimulus (CEECINST/00049/2018). A-F A Mentis reports grants or contracts from MilkSafe: a novel pipeline to enrich formula milk using omics technologies, a research co-financed by the European Regional Development Fund of the European Union and Greek national funds through the operational programme competitiveness, entrepreneurship and innovation, under the call research, create, innovate (T2EDK-02222), as well as from ELIDEK (Hellenic Foundation for Research and Innovation, MIMS-860); stock or stock options in a family winery; support from BGI Group as a scientific officer. L Monasta and L Ronfani report support for the present manuscript from the Italian Ministry of Health on project Ricerca Corrente 34/2017 and payments made to Institute for Maternal and Child Health IRCCS Burlo Garofolo. O Odukoya reports support from the present manuscript from the Fogarty International Center of the National Institutes of Health (K43TW020704) for protected time. The content is solely the responsibility of the authors and does not necessarily represent the official views of the National Institutes of Health. M Postma reports stock or stock options from Pharmacoeconomics Advice Groningen and Health-Ecore, outside the submitted work. M Raad reports consulting fees from Fondation Mérieux, support for attending meetings from Fondation Mérieux, and is the CEO of an antibiotic prescription assistance company SMARTBIOTIC, all outside the submitted work. K E Rudd reports grants or contracts from the NIH National Institute of General Medical Sciences (1K23GM141463), outside the submitted work. C R Simpson reports grants or contracts from New Zealand Ministry of Business, Innovation and Employment, Health Research Council of New Zealand, UK Medical Research Council, and UK Chief Scientist Office, as research grants paid to their institution, outside the submitted work. J A Singh reports consulting fees from Crealta Horizon, Medisys, Fidia, PK Med, Two Labs, Adept Field Solutions, Clinical Care Options, Clearview Healthcare Partners, Putnam Associates, Focus Forward, Navigant Consulting, Spherix, MedIQ, Jupiter Life Science, UBM, Trio Health, Medscape, WebMD, and Practice Point Communications, the National Institutes of Health, and the American College of Rheumatology; payment or honoraria for lectures, presentations, speakers’ bureaus, manuscript writing or educational events from Simply Speaking; support for attending meetings or travel from the steering committee of OMERACT; participation on a data safety monitoring board or advisory board with the US Food and Drug Administration Arthritis Advisory Committee; leadership or fiduciary role in board, society, committee or advocacy group, paid or unpaid, with OMERACT as a steering committee member, with the Veterans Affairs Rheumatology Field Advisory Committee as Chair (unpaid), and with the UAB Cochrane Musculoskeletal Group Satellite Center on Network Meta-analysis and editor and director (unpaid); stock or stock options in TPT Global Tech, Vaxart Pharmaceuticals, Atyu Biopharma, Adaptimmune Therapeutics, GeoVax Labs, Pieris Pharmaceuticals, Enzolytics, Seres Therapeutics, Tonix Pharmaceuticals and Charlotte's Web Holdings, and previously owned stock options in Amarin, Viking, and Moderna Pharmaceuticals; all outside the submitted work. E Upadhyay reports patents published for a system and method of reusable filters for anti-pollution mask and a system and method for electricity generation through crop stubble by using microbial fuel cells and filed for a system for disposed personal protection equipment (PPE) into biofuel through pyrolysis and method and a novel herbal pharmaceutical aid for formulation of gel and method thereof and a leadership or fiduciary role as part of the Joint Secretary of Indian Meteorological Society, Jaipur Chapter (India). A Zumla reports grants or contracts from Pan-African Network on Emerging and Re-Emerging Infections (https://www.pandora-id.net/) funded by the European and developing countries clinical trials partnership the EU horizon 2020 framework programme. Acknowledge support from EDCTP-Central Africa and East African Clinical Research Networks (CANTAM-3, EACCR-3) and unpaid membership of the Scientific Advisory Committee of the EC-EDCTP-3 global health programme, Brussels with effect from March, 2022, all outside the submitted work. All other authors declare no competing interests.
