# Supplementary material for: Age–sex differences in the global burden of lower respiratory infections and risk factors, 1990–2019: results from the Global Burden of Disease Study 2019
Source: Lancet Infect Dis. 2022 Nov;22(11):1626–47. doi: 10.1016/S1473-3099(22)00510-2 (PMC9605880; doi:10.1016/S1473-3099(22)00510-2)
Supplement: Supplementary appendix [file mmc1.pdf]

THE LANCET
Infectious Diseases

Supplementary appendix 1

This appendix formed part of the original submission and has been peer reviewed. We post it as supplied by the authors.

Supplement to: GBD 2019 LRI Collaborators. Age–sex differences in the global burden of lower respiratory infections and risk factors, 1990–2019: results from the Global Burden of Disease Study 2019. *Lancet Infect Dis* 2022; published online Aug 11. https://doi.org/10.1016/S1473-3099(22)00510-2.

Appendix 1

“Age-sex differences in the global burden of lower respiratory infections and risk factors: results from the Global Burden of Disease Study 2019”

This appendix provides further methodological details for “Age-sex differences in the global burden of lower respiratory infections and risk factors: results from the Global Burden of Disease Study 2019”.

All the material in the paper itself is novel although it builds off previous GBD work and the parts of the supplemental methods appendix include sections adapted from the GBD Capstones[1,2] published in The Lancet last year.

1. GBD 2019 Diseases and Injuries Collaborators. Global burden of 369 diseases and injuries in 204 countries and territories, 1990-2019: a systematic analysis for the Global Burden of Disease Study 2019. Lancet 2020; 396(10258): 1204-22.
2. GBD 2019 Risk Factors Collaborators. Global burden of 87 risk factors in 204 countries and territories, 1990-2019: a systematic analysis for the Global Burden of Disease Study 2019. Lancet 2020; 396(10258): 1223-49.

TABLE OF CONTENTS

Appendix 1 ... 1

"Age-sex differences in the global burden of lower respiratory infections and risk factors: results from the Global Burden of Disease Study 2019" ... 1

Lower respiratory infections (morbidity) ... 6

Flowchart ... 6

Case definition ... 6

Input data ... 6

Modelling strategy ... 10

Changes from GBD 2017 ... 11

Lower respiratory infections (mortality) ... 12

Input data ... 12

Modelling strategy ... 13

Risk factors included in this study by age group ... 15

Risk factors and potential causal pathways leading to LRI ... 16

Child growth failure ... 18

Flowchart ... 18

Input data and methodological summary ... 18

Exposure ... 18

Modelling strategy *Exposure estimation* ... 20

Theoretical minimum-risk exposure level ... 20

Relative risks ... 21

References ... 21

Smoking ... 22

Flowchart ... 22

Input data and methodological summary ... 22

Definition ... 22

Input data ... 22

Modelling strategy ... 23

Theoretical minimum-risk exposure level ... 25

Relative risk ... 25

Population attributable fraction (PAF) ... 25

References ... 26

Household air pollution 27
Flowchart 27
Input data and methodological summary 27
Exposure 27
Theoretical minimum-risk exposure level 29
Relative risks 29
$PM_{2.5}$ mapping value 30
References 31
Ambient particulate matter pollution 32
Flowchart 32
Input data and modelling strategy 32
Exposure 32
Modelling strategy 34
Theoretical minimum-risk exposure level 36
Relative risks and population attributable fractions 36
Secondhand smoke 43
Flowchart 43
Exposure 43
Case definition 43
Input data 43
Modelling strategy 44
Theoretical minimum-risk exposure level 44
Relative risks 45
Low birthweight and short gestation 46
Flowchart 46
Exposure 46
Case definition 46
Input data and methodological summary 47
Case definition 48
Exposure 49
Input data and data processing 49
Literature review 50
Modelling strategy 53
Relative risks & theoretical minimum-risk exposure level 54

Causes 54
Input data 54
Modelling strategy 55
PAF calculations 55
References 56
Suboptimal breastfeeding 57
Flowchart 57
Input data and methodological summary 57
Exposure definitions 57
Input data 57
Exposure 57
Exposure modelling 58
Modelling strategy 58
Assessment of risk-outcome pairs 58
Theoretical minimum-risk exposure level 59
Relative risks 59
Population attributable fraction 59
References 59
No access to handwashing facility 60
Flowchart 60
Input data and methodological summary 60
Exposure 60
Modelling strategy 60
Theoretical minimum-risk exposure level 61
Relative risks 61
References 64
Alcohol use 65
Flowchart 65
Input data and methodological summary 65
Definition 65
Input data 65
Modelling strategy 68
Theoretical minimum-risk exposure level 70
Relative risks 70

Population attributable fraction 70
References 71
Non-optimal temperature 73
Flowchart 73
Input data and modelling strategy 73
Case definition 73
Exposure 73
Exposure-response modelling 74
Exposure-response modelling (MR-BRT) 74
Cause selection 76
Theoretical minimum risk exposure level (TMREL) 76
Population attributable fractions 76
References 77
Calculating the burden of multiple risk factors 79
Inclusion of a risk–outcome pair in the GBD 80
Lower Respiratory Infection ICD-9 and ICD-10 Codes and Definitions 81
GATHER Compliance 85
Author Contributions 89
Managing the overall research enterprise 89
Writing the first draft of the manuscript 89
Primary responsibility for applying analytical methods to produce estimates 89
Primary responsibility for seeking, cataloguing, extracting, or cleaning data; designing or coding figures and tables 89
Providing data or critical feedback on data sources 89
Developing methods or computational machinery 90
Providing critical feedback on methods or results 91
Drafting the work or revising it critically for important intellectual content 93
Managing the estimation or publications process 94

Lower respiratory infections (morbidity)

Flowchart

Lower respiratory infections

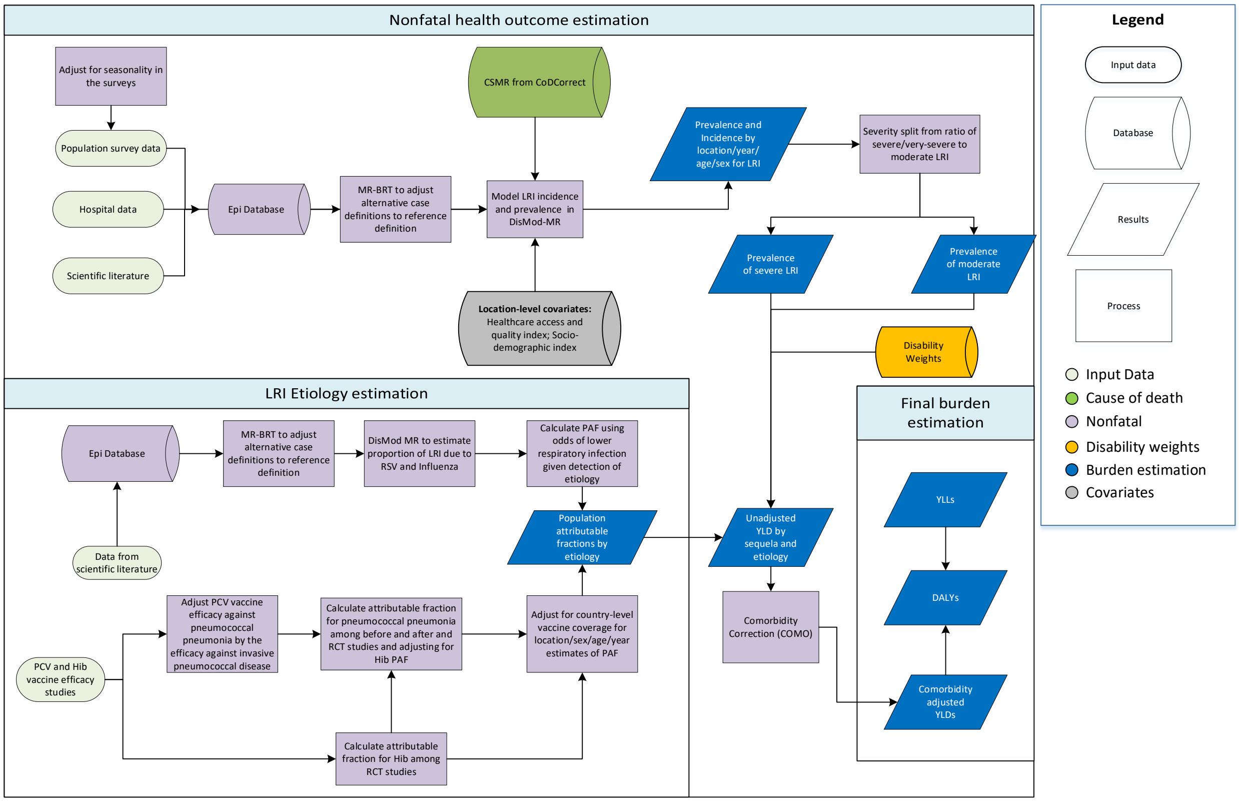

Case definition

We used clinician-diagnosed pneumonia or bronchiolitis as our case definition for lower respiratory infections (LRI). We included ICD9 codes 079.6, 466-469, 470.0, 480-482.8, 483.0-483.9, 484.1-484.2, 484.6-484.7, 487-489 and ICD10 codes A48.1, A70, B97.4-B97.6, J09-J15.8, J16-J16.9, J20-J21.9, J91.0, P23.0-P23.4, U04-U04.9. The definitions for each of the ICD-9 and ICD-10 codes can be found on pages 81–84 in this appendix. LRI etiologies are modeled separately from overall LRI incidence and prevalence.

Input data

Input data included all data used in GBD 2017 and new data identified in our updated systematic review, population-representative surveys, and new claims and inpatient data.

For GBD 2019, we included new survey data from the following countries: Benin, Haiti, Kenya, Mali, Papua New Guinea, Senegal, Uganda, South Africa, Timor-Leste, Guinea, Guinea-Bissau, Mongolia, Mauritania, Sudan, Thailand, Trinidad and Tobago, and Ukraine.

This search string below looks for the incidence and prevalence of LRI cases, and the etiology proportion for influenza and RSV.

(("lower respiratory"[title] OR pneumonia[title]) AND (2018/08/01[PDat] : 2019/2/7[PDat]) AND ((incidence OR prevalence OR epidemiology) OR (etiolog[title/abstract] OR influenza[title/abstract] OR "respiratory syncytial virus"[title/abstract])) AND Humans[MeSH Terms]) NOT(autoimmune[title/abstract] OR COPD [title/abstract] OR "cystic fibrosis"[title/abstract] OR Review[ptyp]) NOT (animals[MeSH] NOT humans[MeSH])*

Our inclusion criteria were studies that had a sample size of at least 100 people (the sample size threshold was chosen arbitrarily), a study duration of at least one year, and included lower respiratory infections, pneumonia, or bronchiolitis in the case definition.

We identified 121 studies, of which 2 met our inclusion criteria and were extracted.

Figure 1. Lower Respiratory Infection updated systematic review (2018/08/01-2019/2/7) PRISMA flow diagram

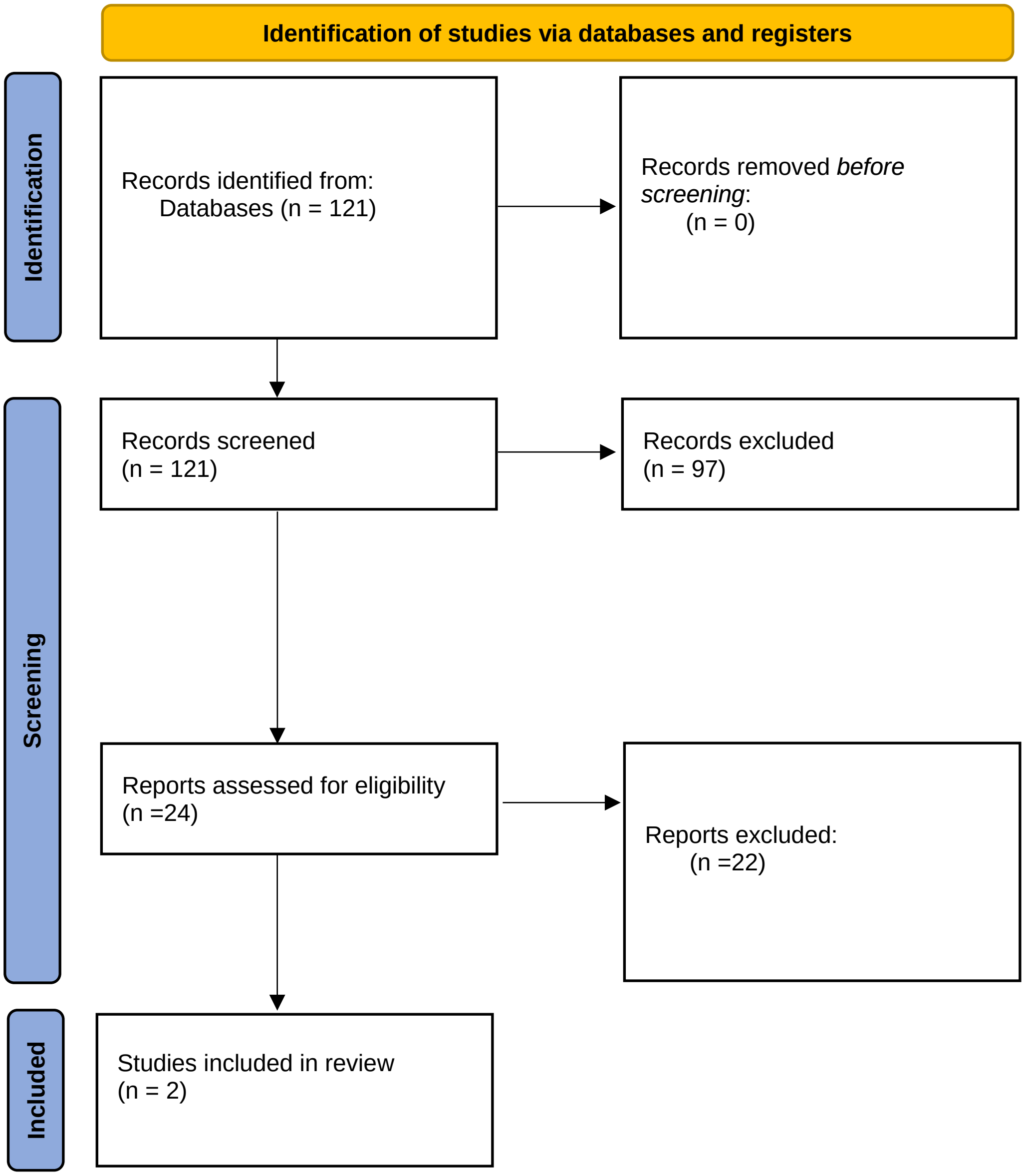

Table 1. Unique source counts for lower respiratory infections by measure

Measure	Total sources	Countries with data
All measures	1152	162
Prevalence	918	154

Figure 1. Total nonfatal data availability (site-years) by location, lower respiratory infections, GBD 2019

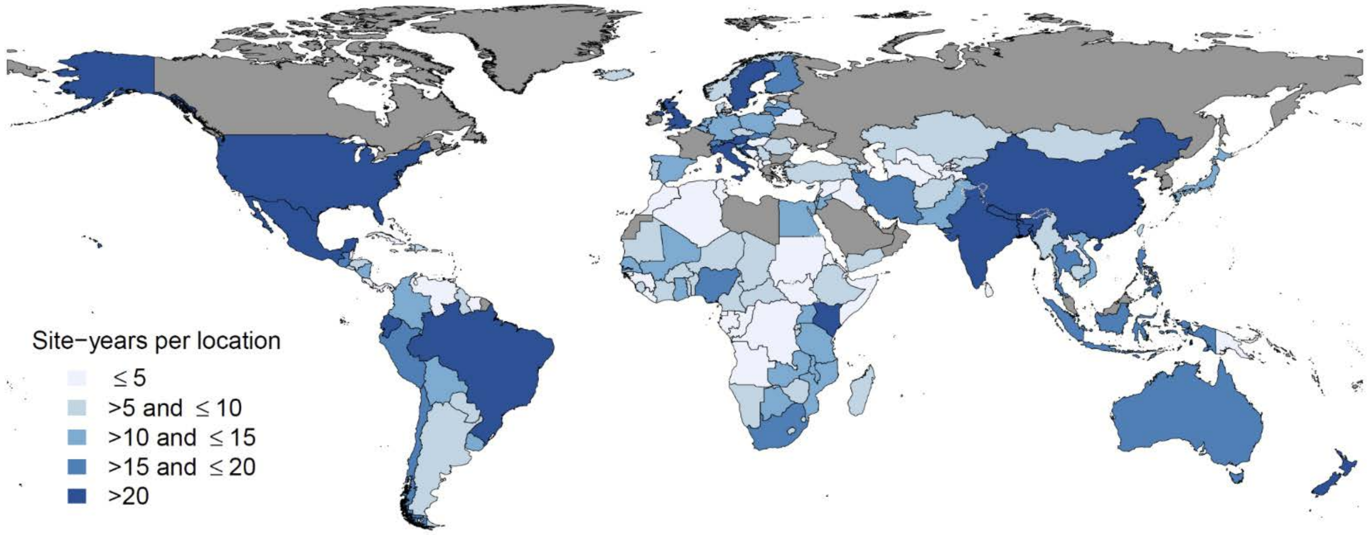

To estimate the non-fatal burden of LRI, we also used self-reported prevalence of LRI symptoms from population-representative surveys, such as the Demographic and Health Survey and the Multiple Indicator Cluster Survey. When possible, we extracted survey data by one-year age group and by sex. We converted these data from two-week period prevalence to point prevalence. The equation for this adjustment is

$$1) \quad Point\ Prevalence = \frac{Period\ Prevalence * Duration}{(Recall\ Period + Duration - 1)}$$

We accepted four survey definitions for the prevalence of symptoms of LRI: 1) Cough with difficulty breathing with the symptoms in the chest with a fever was our gold standard but we also accepted 2) Cough with difficulty breathing with the symptoms in the chest *without* fever, 3) Cough with difficulty breathing with fever, and 4) Cough with difficulty breathing *without* fever. To make these definitions comparable, we identified the surveys that met the best case definition (definition 1). Within these surveys, we calculated the ratio of the prevalence of the best case definition to the prevalence of the alternate definitions. This ratio was used as the dependent variable in a meta-

regression. The results from that meta-regression were used to adjust the prevalence and uncertainty for all the surveys that reported alternate case definitions **(Table 1)**. The survey data adjusted to the best survey definition were then adjusted to the level of our reference case definition of clinician-diagnosed pneumonia or bronchiolitis.

Table 1. Survey crosswalk coefficients

Data Input	Reference or alternative case definition	Gamma	Crosswalk covariate	Beta Coefficient, Logit (95% UI)
Cough, with difficulty breathing and fever	ref	--	--	--
Survey, chest without fever	alt	0.18	intercept	-0.5 (-0.85, -0.15)
Survey, difficulty breath without fever	alt	0.55	intercept	-0.78 (-1.87, 0.31)
Survey, difficulty breathing with fever	alt	0.23	intercept	-0.6 (-1.04, -0.15)

Survey data were adjusted for seasonality. An inclusion criterion for scientific literature is a study duration of at least one year to avoid bias in the seasonal timing of LRI. Surveys are frequently conducted over several months. To account for seasonal variation in LRI symptom prevalence, we fit a generalised additive model with a forced periodicity for each GBD region. The model is mixed-effects with random effects on each country. The model accounts for the year of the survey and the case definition used. The percent difference between the monthly model fit LRI prevalence and the corresponding regional-mean LRI prevalence is a scalar to adjust survey data by month and geography.

In addition to survey data, hospital inpatient, outpatient data, and US claims data were included in the LRI modelling. These data are adjusted prior to modelling for multiple admissions, multiple diagnoses, and for outpatient claims. To make the data more consistent in the modelling process, we converted all incidence data to prevalence. We found the ratio of the prevalence of LRI in hospitalisation records to the prevalence of LRI in our case definition (clinician-diagnosed pneumonia or bronchiolitis) for locations that contained data on both these prevalence values. We then regressed this ratio in a meta-regression to predict the adjustment factor for hospitalisation data to make them compatible with the reference case definition for our modelling. This meta-regression considered the Socio-demographic Index (SDI) as a predictor of this ratio for inpatient data, assuming that location-years with higher values of SDI are more likely to have access to healthcare, making this ratio smaller in those location-years **(Table 2)**. Similarly, age was considered a predictor for hospital-based studies, and data was adjusted accordingly using age midpoint **(Table 3)**.

Table 2. Crosswalk coefficient, clinical inpatient to reference definition

Data Input	Reference or alternative case definition	Gamma	Crosswalk covariate	Beta Coefficient, Logit (95% UI)

clinician-diagnosed pneumonia or bronchiolitis	ref	1.49	--	--
Clinical, inpatient	alt		sdi_0	2.77 (-0.37, 5.92)
Clinical, inpatient	alt		sdi_1	4.82 (3.77, 5.87)
Clinical, inpatient	alt		sdi_2	1.25 (0.22, 2.29)
Clinical, inpatient	alt		sdi_3	0.47 (0.04, 0.9)

Table 3. Crosswalk coefficient, hospital-based studies to reference definition

Data Input	Reference or alternative case definition	Gamma	Covariate	Beta Coefficient, Logit (95% UI)
clinician-diagnosed pneumonia or bronchiolitis	ref	0.3	--	--
Literature, hospital-based	alt		age_mid_0	1.06 (0.03, 2.08)
Literature, hospital-based	alt		age_mid_1	1.98 (-0.16, 4.12)
Literature, hospital-based	alt		age_mid_2	1.31 (0.38, 2.25)
Literature, hospital-based	alt		age_mid_3	0.95 (0.56, 1.34)

Claims data for GBD 2019 include Marketscan (US), and data from Taiwan, Poland, and Russia. Marketscan data are retrieved by IHME's the Clinical Informatics Team. As with inpatient clinical data, these data are converted first to prevalence, then compared to the reference definition for LRI using a meta-regression model **(Table 4)**. Taiwan claims data were dropped as there were no reference data to match with and because the values there were systematically different from those in the United States.

Table 4. Claims to reference crosswalk coefficients

Data Input	Reference or alternative case definition	Gamma	Crosswalk covariate	Beta Coefficient, Logit (95% UI)
Claims, marketscan	Alt	0.39	intercept	-0.87 (-1.67, -0.067)

We performed a systematic review of the duration of symptoms of LRI. We sought consistency with our case definition of LRI and defined our duration as the time between the onset of symptoms to the resolution of increased work of breathing. Although crucial, there were very limited data on spatial, temporal, or age-specific duration, which may vary based on severity, aetiology, and treatment. We identified 485 titles from PubMed and extracted six studies which were used in a meta-analysis (mean duration 7.79 days, 6.2–9.64 days). We used this as the duration of LRI in our conversions from period to point prevalence and for the conversion between incidence and prevalence.

Modelling strategy

The non-fatal lower respiratory infection burden is modelled in DisMod MR 2.1, a Bayesian meta-regression modelling framework. DisMod-MR 2.1 produces estimates of the incidence, prevalence, and remission of LRI for each

age, sex, geographic location, and year. We defined the time to recovery as an average of 10 days (5-15 days), which corresponds with a remission 36.5. The models are informed by country-level covariates (**Table 5**).

Table 5. Model covariates

Study covariate	Type	Parameter	Exponentiated beta (95% Uncertainty Interval)
Socio-demographic Index	Country-level	Prevalence	0.14 (0.14 — 0.14)
Healthcare access and quality index	Country-level	Excess mortality	0.38 (0.15 — 1.00)

Changes from GBD 2017

There is one key methodological change from GBD 2017. All data adjustments in GBD 2019 occur before modeling using a standardized approach. Data adjustments for non-fatal LRI include survey prevalence, inpatient clinical prevalence, and clinical claims prevalence. All of these data sources are adjusted to be comparable with our reference definition. To do so, we first computed the ratio of the alternative data to the reference data based on all available data matched by location, year, age, and sex. We then ran a meta-regression to pool the ratios and used the pooled ratio to crosswalk or adjust alternative data to the level of the reference data. We believe that this represents an improvement in our methodology because it standardizes these adjustments, accounts for between and within study variance, and explicitly creates these ratios using data within studies or location-years.

Lower respiratory infections (mortality)

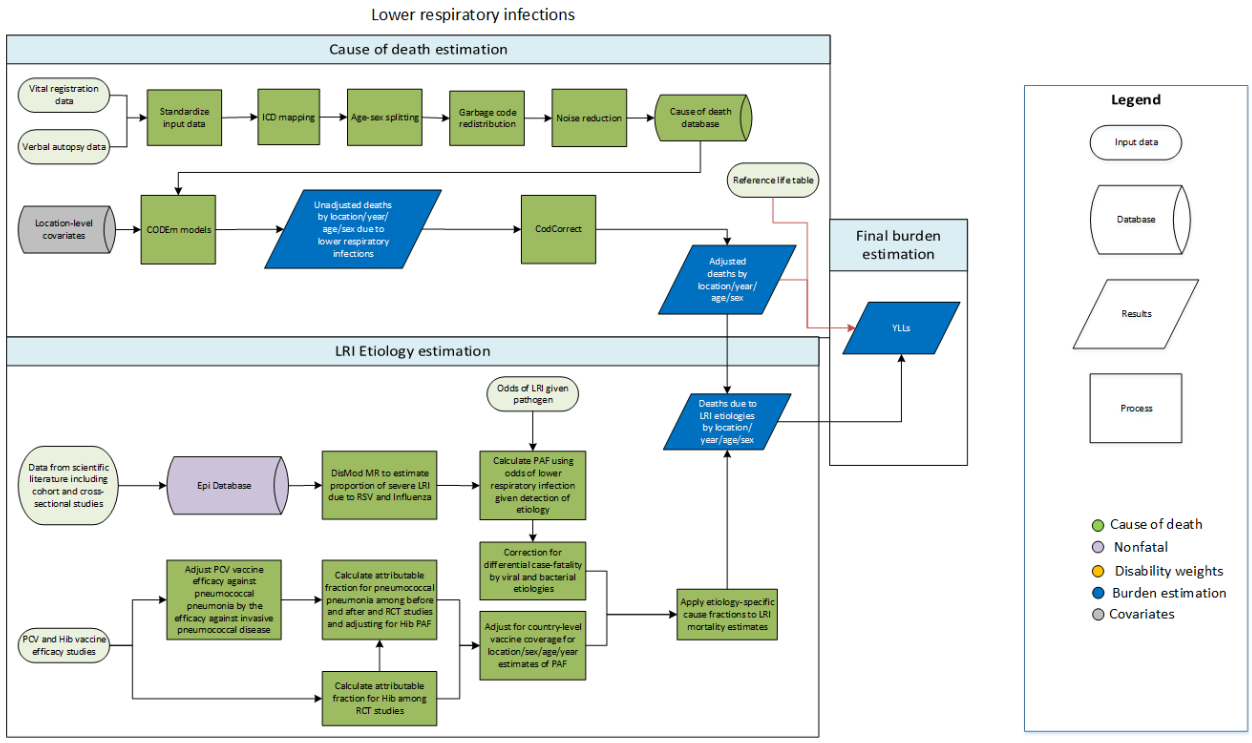

Input data

Lower respiratory infection (LRI) mortality was estimated in CODEm. We estimated LRI mortality separately for males and females and for children under 5 years and older than 5 years. We used all available data from vital registration systems, surveillance systems, and verbal autopsy. We checked for and excluded outliers from our data by country or region. We also excluded ICD9-coded mortality data in Sri Lanka (1982, 1987–1992), ICD9-coded neonatal mortality data in Guatemala (1980, 1981, 1984, 2000–2004), and medically coded cause of death data and Civil Registration System data in many Indian states (1986–2013).

Figure 1. Total cause of death data availability (site-years) by location, lower respiratory infections, GBD 2019

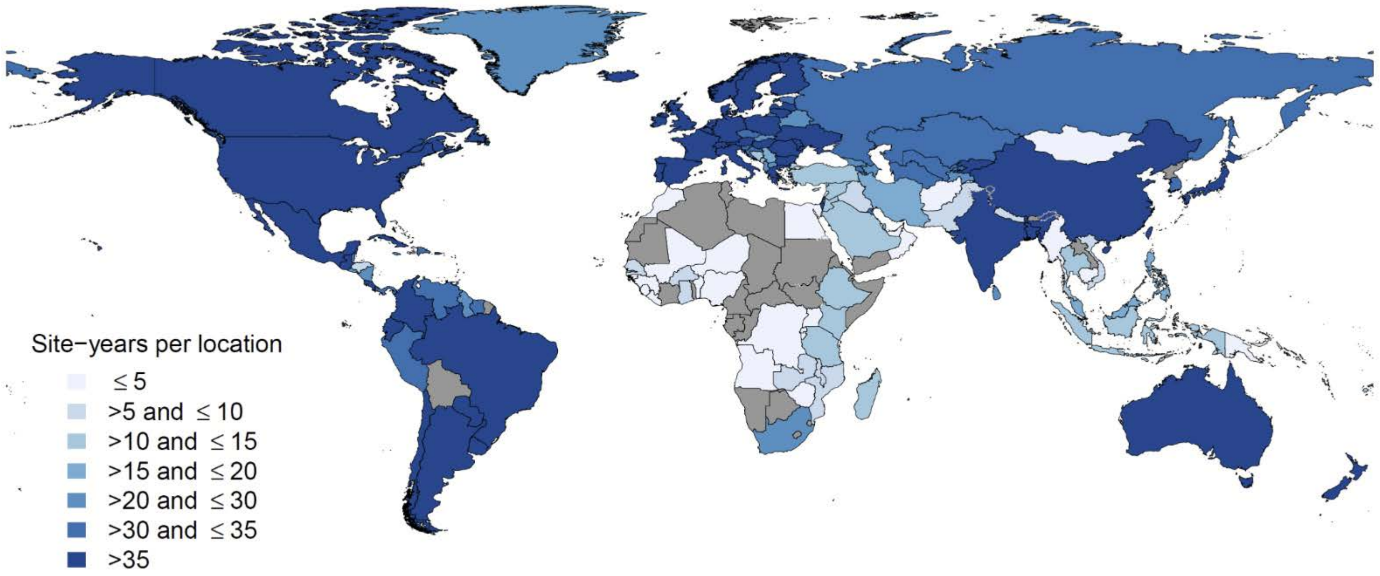

Modelling strategy

LRI fatal modelling occurs using CODEm. Because of starkly different patterns, LRI CODEm models include under-5 years and 5–95+ years. Like all models of mortality in GBD, LRI mortality models are single-cause, requiring in effect that the sum of all mortality models must be equal to the all-cause mortality envelope. We correct LRI mortality estimates, and other causes of mortality, by rescaling them according to the uncertainty around the cause-specific mortality rate. This process is called CoDCorrect and is essential to ensure internal consistency among causes of death.

Table 1. Covariates used in LRI mortality modelling. Table 1A is for children under 5 and Table 1B shows the covariates used for ages 5–95+. The *Level* is the associated strength of relationship between the covariate and LRI mortality, ranked from 1 (proximally related) to 3 (distally related). *Direction* is the direction of the association between the covariate and LRI mortality.

Table 1A. Covariates used in under 5 years model

Level	Covariate	Direction
1	Childhood stunting summary exposure value (SEV)	+
	Childhood underweight SEV	+
	Childhood wasting SEV	+
	Indoor air pollution	+
	LRI SEV	+
	Antibiotics for LRI	-
	Hib vaccine coverage	-
	PCV coverage	-
	Vitamin A deficiency	+

2	Secondhand smoking prevalence	+
	Zinc deficiency	+
	DTP3 vaccine coverage	-
	Healthcare Access and Quality Index	-
	Ambient particulate matter SEV	+
	Household air pollution	+
	Outdoor air pollution ($PM_{2.5}$)	+
	Handwashing SEV	+
3	Sanitation SEV	+
	Population density > 1000/km^2	+
	Population density < 150/km^2	+
	Maternal education	-
	Socio-demographic Index	-

Table 1B. Covariates used in 5-95+ years model

Level	Covariate	Direction
1	Indoor air pollution	+
	LRI SEV	+
	Outdoor air pollution	+
	Secondhand smoking prevalence	+
	Smoking prevalence	+
2	DTP3 vaccine coverage	-
	Adult underweight	+
	Healthcare Access and Quality Index	-
	PCV coverage	-
	Handwashing access	+
3	Education years per capita	-
	Lag distributed income per capita	-
	Socio-demographic Index	-
	Sanitation SEV	+

Risk factors included in this study by age group

**Age group**	**Risk factors**
Under five	Child wasting, child underweight, child stunting, low birth weight, short gestation, non-exclusive breastfeeding, ambient particulate matter, household air pollution, secondhand smoke, handwashing, high temperature, low temperature
5-14	Ambient particulate matter, household air pollution, secondhand smoke, handwashing, high temperature, low temperature
15-49; 50-69; 70+	Ambient particulate matter, household air pollution, smoking, secondhand smoke, alcohol use, handwashing, high temperature, low temperature.

Risk factors and potential causal pathways leading to LRI

Risk factor	Mechanism
Child malnutrition (wasting, underweight, stunting)	Malnutrition weakens immune response; it especially impairs mucosal epithelial barriers in the respiratory tract and also decreases leukocyte microbicidal capacity.[1]
Household air pollution	Adversely affects airway defense against infection such as inducing epithelial inflammation, surfactant dysfunction, and cilial function impairment.[2]
Ambient particulate matter	Ambient particulate matter can serve as bacterial carriers, impair antimicrobial activity in the respiratory tract, reduce mucociliary transportation, and hinder the phagocytic capacity against pathogens.[3]
Short gestation, low birth weight	Immature immune system with impaired innate and adaptive immunity.[4-7]
No access to handwashing facility	Hands carry respiratory pathogens to the nasal mucosa and conjunctiva.[8]
Non-exclusive breastfeeding	Breast milk involves immune cells (for defense against pathogens), antibodies, and immune system modulators that protect the infant against respiratory infections.[9]
Smoking	Smoking interferes with the structural, functional, and immunologic defense mechanisms.[10]
Secondhand smoke	Induces inflammatory response and weakens the immune system.[11,12]
Alcohol use	Elevates the risk of microbial aspiration and impairs the host's immunity.[13]
Non-optimal temperature	Decreases thermoregulation capability and increases vulnerability to infection.[14]

References

1. Rodríguez L, Cervantes E, Ortiz R. Malnutrition and gastrointestinal and respiratory infections in children: a public health problem. *Int J Environ Res Public Health* 2011; **8**(4): 1174-205.
2. Gordon SB, Bruce NG, Grigg J, et al. Respiratory risks from household air pollution in low and middle income countries. *Lancet Respir Med* 2014; **2**(10): 823-60.
3. Vargas Buonfiglio LG, Comellas AP. Mechanism of ambient particulate matter and respiratory infections. *J Thorac Dis* 2020; **12**(3): 134-6.
4. Levy O. Innate immunity of the newborn: basic mechanisms and clinical correlates. *Nature Reviews Immunology* 2007; **7**(5): 379-90.

5. Miller JE, Hammond GC, Strunk T, et al. Association of gestational age and growth measures at birth with infection-related admissions to hospital throughout childhood: a population-based, data-linkage study from Western Australia. *Lancet Infect Dis* 2016; **16**(8): 952-61.
6. van den Berg JP, Westerbeek EA, van der Klis FR, Berbers GA, van Elburg RM. Transplacental transport of IgG antibodies to preterm infants: a review of the literature. *Early Hum Dev* 2011; **87**(2): 67-72.
7. Melville JM, Moss TJM. The immune consequences of preterm birth. *Front Neurosci* 2013; **7**: 79-.
8. Rabie T, Curtis V. Handwashing and risk of respiratory infections: a quantitative systematic review. *Trop Med Int Health* 2006; **11**(3): 258-67.
9. Horta BL, Victora CG, World Health O. Short-term effects of breastfeeding: a systematic review on the benefits of breastfeeding on diarrhoea and pneumonia mortality. Geneva: World Health Organization; 2013.
10. Jiang C, Chen Q, Xie M. Smoking increases the risk of infectious diseases: A narrative review. *Tob Induc Dis* 2020; **18**: 60-.
11. Flouris AD, Vardavas CI, Metsios GS, Tsatsakis AM, Koutedakis Y. Biological evidence for the acute health effects of secondhand smoke exposure. *Am J Physiol Lung Cell Mol Physiol* 2010; **298**(1): L3-l12.
12. Bhat TA, Kalathil SG, Bogner PN, et al. Secondhand Smoke Induces Inflammation and Impairs Immunity to Respiratory Infections. *The Journal of Immunology* 2018; **200**(8): 2927-40.
13. Mehta AJ, Guidot DM. Alcohol abuse, the alveolar macrophage and pneumonia. *The American journal of the medical sciences* 2012; **343**(3): 244-7.
14. Qiu H, Sun S, Tang R, Chan K-P, Tian L. Pneumonia Hospitalization Risk in the Elderly Attributable to Cold and Hot Temperatures in Hong Kong, China. *Am J Epidemiol* 2016; **184**(8): 570-8.

Child growth failure

Flowchart

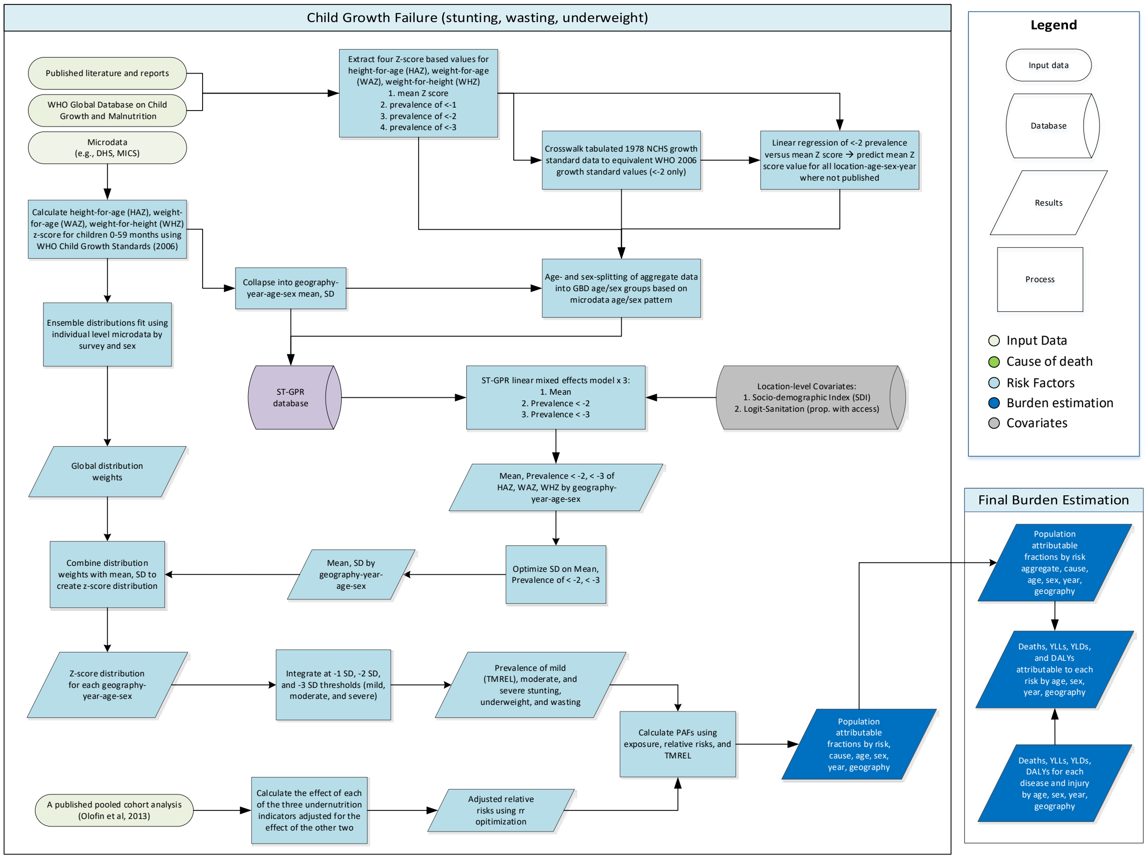

Input data and methodological summary

Exposure

Case definition

Child growth failure is estimated using three indicators (stunting, wasting, and underweight), all of which are based on categorical definitions using the WHO 2006 growth standards for children 0-59 months. Definitions are based on Z scores from the growth standards, which were derived from an international reference population. Mild (<-1 to -2 Z score), moderate (<-2 to -3 Z score), and severe (<3 Z score) categorical prevalences were estimated for each of the three indicators.

Input data

There are three main inputs for the GBD child growth failure models: microdata from population surveys, tabulated data from reports and published literature, and the WHO Global Database on Child Growth and Malnutrition.[1] The primary data additions in GBD 2019 for child growth failure were from population surveys that include anthropometry. Population surveys include a variety of multi-country and country-specific survey series such as Multiple Indicator Cluster Surveys (MICS), Demographic and Health Surveys (DHS), Living Standards Measurement Surveys (LSMS), and the China Health and Nutrition Survey (CHNS), as well as other one-time country-specific surveys

such as the Indonesia Family Life Survey and the Brazil National Demographic and Health Survey of Children and Women. These microdata contain information about each individual child's age (from which age in weeks and age in months are calculated), as well as height and/or weight. From that information, a height-for-age z-score (HAZ), weight-for-age z-score (WAZ), and weight-for-height z-score (WHZ) are calculated using the WHO 2006 Child Growth Standards and the LMS method.[2] In GBD 2019, several new data-cleaning criteria were applied to increase the quality of the microdata set. Data that did not meet the following criteria were dropped: 1) non-sex-specific data, 2) data with invalid Z-scores (HAZ, WAZ, WHZ, or BMI above 6 SD or below -6 SD), and 3) data with impossible values (negative height, weight, or age).

All available data from the WHO Global Database on Child Growth and Malnutrition were extracted in GBD 2016 – much of which are from published studies. Exclusions included examination date prior to 1985, non-population-representative studies, and those based on self-report. A systematic literature review was last completed in GBD 2010. We looked for four metrics from all sources with tabulated data: mean Z score, prevalence <-1 Z score, prevalence <-2 Z score, and prevalence <-3 Z score. All data for each metric were extracted for each of stunting (height-for-age Z score; HAZ), wasting (weight-for-height Z score; WHZ), and underweight (weight-for-age Z score; WAZ).

Table 1: Input data counts for Child wasting exposure models

Input data	Exposure
Source count (total)	1240
Number of countries with data	151

Table 2: Input data counts for Child underweight exposure models

Input data	Exposure
Source count (total)	1270
Number of countries with data	150

Table 3: Input data counts for Child stunting exposure models

Input data	Exposure
Source count (total)	1262
Number of countries with data	151

Data processing

To maximise internal consistency and comprehensiveness of the modelling dataset, we performed three data transformations. First, any data that were reported using the National Center for Health Statistics (NCHS) 1978 growth standards were crosswalked to corresponding values on the WHO 2006 Growth Standards curves based on a study that evaluated growth standard concordance.[3] Crosswalks from 1978 to 2006 growth standards were

performed using OLS linear regression only on <-2 (ie, moderate) prevalence data, as that is where the concordance was most consistent. Second, for any study that lacked a measure of mean Z score for any of stunting, wasting, or underweight, we predicted a mean value for that study based on an ordinary-least-squares regression of mean Z score versus <-2 prevalence for that metric from all sources where both were available. Third, for any data that were presented as both sexes combined or for 0-59 months combined, we used the age and sex pattern from all data sources that included that detail to split into corresponding and age- and sex-specific data.

Modelling strategy

Exposure estimation

The following four-step modelling process was applied in parallel to each of stunting, wasting, and underweight.

First, all microdata were fit using an ensemble modelling process, a modelling framework developed for GBD 2019 that is described elsewhere in this appendix. A series of 12 individual distributions (normal, log-normal, log-logistic, exponential, gamma, mirror gamma, inverse gamma, gumbel, mirror gumbel, Weibull, inverse Weibull, and beta) were fit to the entire set of microdata (approximately 2.5 million individual z-scores) at the individual survey level. A weighting algorithm combined each distribution to find the optimal combination of these distributions for each survey, minimising the absolute prediction error across the entire distribution. Ensemble weights for each survey were then averaged across all surveys to produce a single set of global weights of the ensemble distributions. Weights were different for each sex, but invariant across geography, time, and age group. All component distributions that were used to derive weights were parameterised using “method of moments,” meaning that each corresponding probability density function (PDF) could be described as a function of the mean and variance of the quantity of interest.

Second, models were developed for mean Z scores and prevalence of moderate and severe growth failure. Individual-level microdata were collapsed to calculate three metrics: mean z-score, moderate prevalence, and severe prevalence. These data were combined with those derived from literature, GHDx review, and the WHO Global Database on Child Growth and Malnutrition. Each of the three metrics was then modelled using spatiotemporal Gaussian process regression (ST-GPR), a common modelling framework used across GBD, generating estimates for each age group, sex, year, and location. Location-level covariates used in all models included Socio-demographic Index (SDI) and logit-transformed proportion of households with improved sanitation.

Third, we combined estimates of mean, prevalence (moderate and severe) with ensemble weights in an optimisation framework in order to derive the variance that would best correspond to the predicted mean and prevalence. This variance was then paired with the mean and, using the method of moments equation for each of the component distributions of the ensemble, PDF of the distribution of Z-scores were calculated for each location, year, age group, and sex.

Fourth, PDFs were integrated to determine the prevalence between -1 and -2 Z scores (mild), between -2 and -3 Z scores (moderate), and below -3 Z scores (severe). These were categorical exposures used for subsequent attributable risk analysis.

Theoretical minimum-risk exposure level

Theoretical minimum risk exposure level (TMREL) for underweight, stunting, and wasting was assigned to be greater than or equal to -1 SD of the WHO 2006 standard weight-for-age, height-for-age, and weight-for-height curves, respectively. This has not changed since GBD 2010.

Relative risks

The relative risks for the association between child growth failure and lower respiratory infections are shown in Table 4. These were derived from a pooled cohort analysis by Olofin and colleagues.[5]

There is a high degree of correlation between stunting, wasting, and underweight. Failing to account for their covariance and assuming independence would overestimate the total burden significantly. This is the main reason that GBD 2010 only included childhood underweight. In GBD 2013, a method was developed to adjust observed RRs of Olofin and colleagues by simulating the joint distribution of the three indicators using the distribution of each indicator and covariance between indicators in the countries included in the meta-analysis (extracted from Demographic and Health Survey (DHS) micro-data).[4] Based on the analysis done by McDonald and colleagues, we assumed there is an interaction between the three indicators, and extracted the interaction terms from the corresponding analysis. We calculated the adjusted RRs by minimising the error between observed crude RRs (from meta-analysis) and expected crude RRs derived from adjusted RRs.

Table 4: Adjusted RRs for the association between child growth failure and lower respiratory infections

Outcome	Stunting	Wasting	Underweight
**Lower respiratory infections**	<-1: 1.125 (0.998-1.655)<br><-2: 1.318 (1.014-2.165)<br><-3: 2.355 (1.15-5.114)	<-1: 5.941 (1.972-11.992)<br><-2: 20.455 (70.84-37.929)<br><-3: 47.67 (15.923-94.874)	<-1: 1.145 (1.044-1.364)<br><-2: 1.365 (1.215-1.755)<br><-3: 2.593 (1.908-4.39)

References

1 WHO | WHO Global Database on Child Growth and Malnutrition. WHO. http://www.who.int/nutgrowthdb/en/ (accessed July 30, 2018).

2 Wang Y, Chen H-J. Use of Percentiles and Z-Scores in Anthropometry. In: Preedy VR, ed. Handbook of Anthropometry. New York, NY: Springer New York, 2012: 29–48.

3 Uribe Á, Cecilia M, López Gaviria A, Estrada Restrepo A. Concordance between Z scores from WHO 2006 and the NCHS 1978 growth standards of children younger than five. Antioquia-Colombia. *Perspectivas en Nutrición Humana* 2008; 10: 177–87.

4 McDonald CM, Olofin I, Flaxman S, *et al.* The effect of multiple anthropometric deficits on child mortality: meta-analysis of individual data in 10 prospective studies from developing countries. *Am J Clin Nutr* 2013; 97: 896–901.

5 Olofin I, McDonald CM, Ezzati M, *et al.* Associations of Suboptimal Growth with All-Cause and Cause-Specific Mortality in Children under Five Years: A Pooled Analysis of Ten Prospective Studies. *PLOS ONE* 2013; 8: e64636.

Smoking

Flowchart

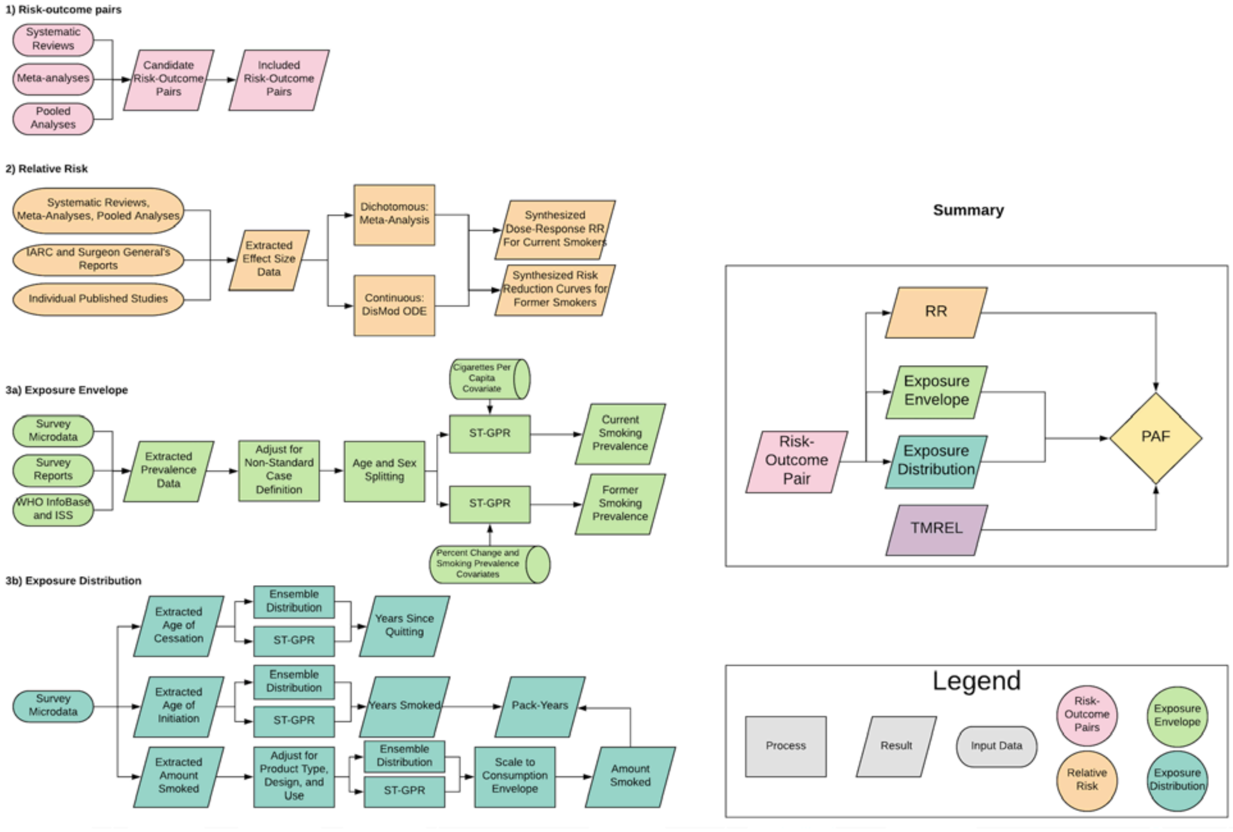

Input data and methodological summary

Definition

Exposure

As in GBD 2017, we estimated the prevalence of current smoking and the prevalence of former smoking using data from cross-sectional nationally representative household surveys. We defined current smokers as individuals who currently use any smoked tobacco product on a daily or occasional basis. We defined former smokers as individuals who quit using all smoked tobacco products for at least six months, where possible, or according to the definition used by the survey.

Input data

We extracted primary data from individual-level microdata and survey report tabulations. We extracted data on current, former, and/or ever smoked tobacco use reported as any combination of frequency of use (daily, occasional, and unspecified, which includes both daily and occasional smokers) and type of smoked tobacco used (all smoked tobacco, cigarettes, hookah, and other smoked tobacco products such as cigars or pipes), resulting in 36 possible combinations. Other variants of tobacco products, for example hand-rolled cigarettes, were grouped into the four type categories listed above based on product similarities.

For microdata, we extracted relevant demographic information, including age, sex, location, and year, as well as survey metadata, including survey weights, primary sampling units, and strata. This information allowed us to tabulate individual-level data in the standard GBD five-year age-sex groups and produce

accurate estimates of uncertainty. For survey report tabulations, we extracted data at the most granular age-sex group provided.

Table 1: Data inputs for exposure for smoking.

Input data	Exposure
Source count (total)	3439
Number of countries with data	201

Table 2: Data inputs for relative risks for smoking.

Input data	Relative risk
Source count (total)	673
Number of countries with data	16

Crosswalk

Our GBD smoking case definitions were current smoking of any tobacco product and former smoking of any tobacco product. All other data points were adjusted to be consistent with either of these definitions. Some sources contained information on more than one case definition and these sources were used to develop the adjustment coefficient to transform alternative case definitions to the GBD case definition. The adjustment coefficient was the beta value derived from a linear model with one predictor and no intercept. We used the same crosswalk adjustment coefficients as in GBD 2017, and thus we have not included a methods explanation in this appendix, as it has been detailed previously.

Age and sex splitting

As in GBD 2017, we split data reported in broader age groups than the GBD 5-year age groups or as both sexes combined by adapting the method reported in Ng et al[1] to split using a sex- geography- time-specific reference age pattern. We separated the data into two sets: a training dataset, with data already falling into GBD sex-specific 5-year age groups, and a split dataset, which reported data in aggregated age or sex groups. We then used spatiotemporal Gaussian process regression (ST-GPR) to estimate sex-geography-time-specific age patterns using data in the training dataset. The estimated age patterns were used to split each source in the split dataset.

The ST-GPR model used to estimate the age patterns for age-sex splitting used an age weight parameter value that minimises the effect of any age smoothing. This parameter choice allowed the estimated age pattern to be driven by data, rather than being enforced by any smoothing parameters of the model. Because these age-sex split data points were to be incorporated in the final ST-GPR exposure model, we did not want to doubly enforce a modelled age pattern for a given sex-location-year on a given aggregate data point.

Modelling strategy

Smoking prevalence modelling

We used ST-GPR to model current and former smoking prevalence. The model is nearly identical to that in GBD 2017. Full details on the ST-GPR method are reported elsewhere in the appendix. Briefly, the mean function input to GPR is a complete time series of estimates generated from a mixed effects hierarchical linear model plus weighted residuals smoothed across time, space, and age. The linear model formula for current smoking, fit separately by sex using restricted maximum likelihood in R, is:

$$logit(p_{g,a,t}) = \beta_0 + \beta_1 CPC_{g,t} + \sum_{k=2}^{19} \beta_k I_{A[a]} + \alpha_s + \alpha_r + \alpha_g + \epsilon_{g,a,t}$$

Where $CPC_{g,t}$ is the tobacco consumption covariate by geography g and time t, described above, $I_{A[a]}$ is a dummy variable indicating specific age group A that the prevalence point $p_{g,a,t}$ captures, and α_s, α_r, and α_g are super-region, region, and geography random intercepts, respectively. Random effects were used in model fitting but not in prediction.

The linear model formula for former smoking is:

$$logit(p_{g,a,t}) = \beta_0 + \beta_1 PctChange_{A[a],g,t} + \beta_3 CSP_{A[a],g,t} + \sum_{k=3}^{20} \beta_k I_{A[a]} + \alpha_s + \alpha_r + \alpha_g + \epsilon_{g,a,t}$$

Where $PctChange_{A[a],g,t}$ is the percentage change in current smoking prevalence from the previous year, and $CSP_{A[a],g,t}$ is the current smoking prevalence by specific age group A, geography g, and time t that point $p_{g,a,t}$ captures, both derived from the current smoking ST-GPR model defined above.

Supply-side estimation

The methods for modelling supply-side-level data were changed substantially from those used in GBD 2017. The raw data were domestic supply (USDA Global Surveillance Database and UN FAO) and retail supply (Euromonitor) of tobacco. Domestic supply was calculated as production + imports - exports. The data went through three rounds of outliering. First, they were age-sex split using daily smoking prevalence to generate number of cigarettes per smoker per day for a given location-age-sex-year. If more than 12 points for a particular source-location-year (equal to over 1/3 of the split points) were above the given thresholds, that source-location-year was outliered. A point would not be outliered if it was (in cigarettes per smoker): under five (10–14 year olds); under 20 (males, 15–19 year olds); under 18 (females, 15–19 year olds); under 38/35 and over three (males/females, 20+ year olds). These thresholds were chosen by visualising histograms of the data for each age-sex, as well as with expert knowledge about reasonable consumption levels. In the second round of outliering, the mean tobacco per capita value over a 10-year window was calculated. If a point was over 70% of that mean value away from the mean value, it was outliered. The 70% limit was chosen using histograms of these distances. Additionally, some manual outliering was performed to account for edge cases. Finally, data smoothing was performed by taking a three-year rolling mean over each location-year.

Next, a simple imputation to fill in missing years was performed for all series to remove compositional bias from our final estimates. Since the data from our main sources covered different time periods, by imputing a complete time series for each data series, we reduced the probability that compositional bias of the sources was leading to biased final estimates. To impute the missing years for each series, we modelled the log ratio of each pair of sources as a function of an intercept and nested random effects on super-region, region, and location. The appropriate predicted

ratio was multiplied by each source that we did have, and then the predictions were averaged to get the final imputed value. For example, if source A was missing for a particular location-year, but sources B and C were present, then we predicted A twice: once from the modelled ratio of A to B, and again from the modelled ratio of A to C. These two predictions were then averaged. For some locations where there was limited overlap between series, the predicted ratio did not make sense, and a regional ratio was used.

Finally, variance was calculated both across series (within a location-year) as well as across years (within a location-source). Additionally, if a location-year had one imputed point was, the variance was multiplied by 2. If a location-year had two imputed points, the variance was multiplied by 4. The average estimates in each location-year were the input to an ST-GPR model. For this, we used a simple mixed effects model, which was modelled in log space with nested location random effects. Subnational estimates were then further modelled by splitting the country-level estimates using current smoking prevalence.

Theoretical minimum-risk exposure level

The theoretical minimum-risk exposure level is 0.

Exposure among current and former smokers

Identical to GBD 2017, we estimated exposure among current smokers for two continuous indicators: cigarettes per smoker per day and pack-years. Pack-years incorporates aspects of both duration and amount. One pack-year represents the equivalent of smoking one pack of cigarettes (assuming a 20-cigarette pack) per day for one year. Since the pack-years indicator collapses duration and intensity into a single dimension, one pack-year of exposure can reflect smoking 40 cigarettes per day for six months or smoking 10 cigarettes per day for two years.

To produce these indicators, we simulated individual smoking histories based on distributions of age of initiation and amount smoked. We informed the simulation with cross-sectional survey data capturing these indicators, modelled at the mean level for all locations, years, ages, and sexes using ST-GPR. We rescaled estimates of cigarettes per smoker per day to an envelope of cigarette consumption based on supply-side data. We estimated pack-years of exposure by summing samples from age- and time-specific distributions of cigarettes per smoker for a birth cohort in order to capture both age trends and time trends and avoid the common assumption that the amount someone currently smokes is the amount they have smoked since they began smoking. All distributions were age-, sex-, and region- specific ensemble distributions, which were found to outperform any single distribution.

We estimated exposure among former smokers using years since cessation. We utilised ST-GPR to model mean age of cessation using cross-sectional survey data capturing age of cessation. Using these estimates, we generated ensemble distributions of years since cessation for every location, year, age group, and sex.

Relative risk

We examined the smoking-lower respiratory infections risk-outcome pair using the same input data for relative risks as in GBD 2017. We synthesised effect sizes by cigarettes per smoker per day, pack-years, and years since quitting from cohort and case-control studies to produce nonlinear dose-response curves using a Bayesian meta-regression model.

We estimated risk curves of former smokers compared to never smokers taking into account the rate of risk reduction among former smokers seen in the cohort and case-control studies, and the cumulative exposure among former smokers within each age, sex, location, and year group.

Population attributable fraction (PAF)

As in GBD 2017, we estimated PAFs based on the following equation:

$$PAF = \frac{p(n) + p(f) \int \exp(x) * rr(x) + p(c) \int \exp(y) * rr(y) - 1}{p(n) + p(f) \int \exp(x) * rr(x) + p(c) \int \exp(y) * rr(y)}$$

where $p(n)$ is the prevalence of never smokers, $p(f)$ is the prevalence of former smokers, $p(c)$ is the prevalence of current smokers, $\exp(x)$ is a distribution of years since quitting among former smokers, $rr(x)$ is the relative risk for years since quitting, $\exp(y)$ is a distribution of cigarettes per smoker per day or pack-years, and $rr(y)$ is the relative risk for cigarettes per smoker per day or pack-years.

We used pack-years as the exposure definition for cancers and chronic respiratory diseases, and cigarettes per smoker per day for cardiovascular diseases and all other health outcomes.

References

1. Ng M, Freeman MK, Fleming TD, Robinson M, Dwyer-Lindgren L, Thomson B, et al. Smoking Prevalence and Cigarette Consumption in 187 Countries, 1980–2012. *JAMA*. 2014 Jan 8;311(2):183–92.

Household air pollution

Flowchart

Household Air Pollution from Solid Fuels

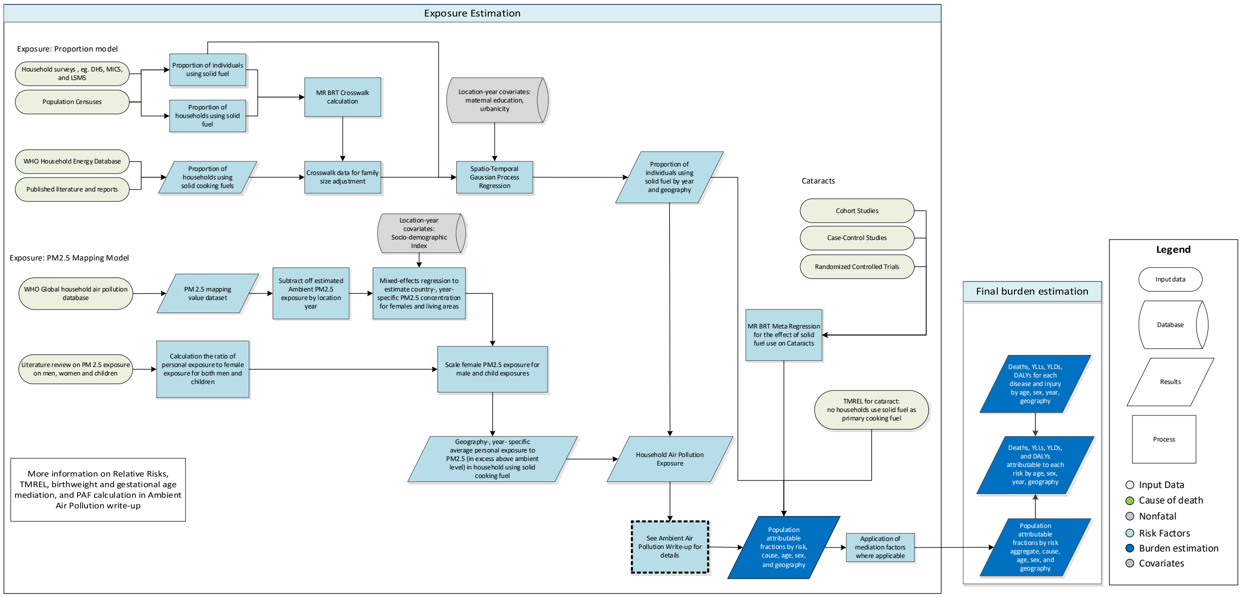

Input data and methodological summary

Exposure

Case definition

Exposure to household air pollution from solid fuels (HAP) is estimated from both the proportion of individuals using solid cooking fuels and the level of $PM_{2.5}$ air pollution exposure for these individuals. Solid fuels in our analysis include coal, wood, charcoal, dung, and agricultural residues.

Input data

We extracted information on use of solid fuels from the standard multi-country survey series such as Demographic and Health Surveys (DHS), Living Standards Measurement Surveys (LSMS), Multiple Indicator Cluster Surveys (MICS), and World Health Surveys (WHS), as well as censuses and country-specific survey series such as Kenya Welfare Monitoring Survey and South Africa General Household Survey. To fill the gaps of data in surveys and censuses, we also downloaded and updated estimates from WHO Energy Database and extracted from literature through systematic review. Each nationally or subnationally representative datapoint provided an estimate for the percentage of households using solid cooking fuels. We used studies from 1980 to 2019 to inform the time series.

We also excluded sources that did not distinguish specific primary fuel types, estimated fuel used for purposes other than cooking (eg, lighting or heating), failed to report standard error or sample size, had over 15% of households with missing responses, reported fuel use in physical units, or were secondary sources referencing primary analyses. Table 1 summarizes exposure input data.

Table 1: Exposure Input Data

Input data	Exposure
Source count (total)	1680
Number of countries with data	195

Family size crosswalk

Many estimates in the WHO Energy Database and other reports quantify the proportion of households using solid fuel for cooking; however, we are interested in the proportion of individuals using solid fuel for cooking. To crosswalk these estimates, whenever we had the available information, we extracted fuel use at both the individual and household levels. We included 3676 source-specific pairs in the MR-BRT crosswalk model.

MR-BRT crosswalk adjustment factors for household air pollution exposure

Data input	Reference or alternative case definition	Gamma	Beta coefficient, logit (95% CI)
Proportion of individuals	Ref	0.097	---
Proportion of Households	Alt		-0.095 (-0.100, -0.090)

We then apply this coefficient to household-only reports with the following formula:

$prop_{individ}$ = the proportion of individuals using solid fuel for cooking, and

$prop_{hh}$ = the proportion of households using solid fuel for cooking.

$$\log\left(\frac{prop_{individ}}{1-prop_{individ}}\right) = \log\left(\frac{prop_{hh}}{1-prop_{hh}}\right) - \beta$$

or

$$prop_{individ} = \frac{prop_{hh} * e^{-\beta}}{1 - prop_{hh} + prop_{hh} * e^{-\beta}}$$

The effect is that the household studies are inflated to account for bias. Larger households are more likely to use solid fuel for cooking. The following figure depicts the 3676 data points that informed the crosswalk model. There the red points indicate the 10% of studies that were trimmed as outliers.

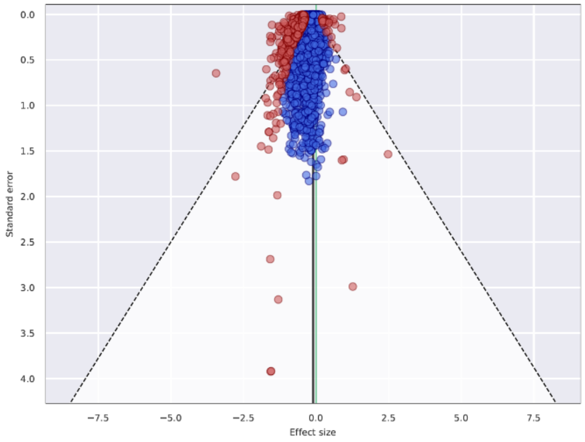

$$logit(prop_{hh}) - logit(prop_{individual})$$

Modelling strategy

Household air pollution was modelled at individual level using a three-step modelling strategy that uses linear regression, spatiotemporal regression, and Gaussian process regression (GPR). The first step is a mixed-effect linear regression of logit-transformed proportion of individuals using solid cooking fuels. The linear model contains maternal education and the proportion of population living in urban areas as covariates and has nested random effects by GBD region and GBD super-region. The full ST-GPR process is specified elsewhere this appendix. No substantial modelling changes were made in this round compared to GBD 2017.

First-stage linear model and coefficients

$$logit(proportion) \sim maternal\ education + urbanicity + (1|region) + (1|super - region)$$

Variable	Beta (95% CI)
Intercept	3.16 (1.59, 4.74)
Maternal education (years per capita)	-0.45 (-0.76, -0.15)
Urbanicity (proportion of population living in urban areas)	-1.42 (-2.67, -0.17)

Theoretical minimum-risk exposure level

For cataract, the TMREL is defined as no households using solid cooking fuel. For outcomes related to both ambient and household air pollution, the PAFs are estimated jointly and the TMREL is defined as uniform distribution between 2.4 and 5.9 ug/m^3 $PM_{2.5}$.

Relative risks

In addition to the previously included outcomes of lower respiratory infections (LRI), stroke, ischaemic heart disease (IHD), chronic obstructive pulmonary disease (COPD), lung cancer, type 2 diabetes, and cataract, in GBD 2019 we added low birthweight and short gestation as new outcomes of household air pollution through a mediation analyses. With the exception of cataract, all causes share risk curves and are jointly calculated with ambient $PM_{2.5}$ air pollution. Table 2 summarizes relative risk input data for ambient particulate matter pollution and household air pollution.

Table 2: Relative Risk Input Data

Input data	Relative risk
Source count (total)	200
Number of countries with data	40

$PM_{2.5}$ mapping value

In order to use the particulate matter risk curves, we must estimate the level of exposure to particulate matter with diameter of less than 2.5 micrometers ($PM_{2.5}$) for individuals using solid fuels for cooking. The Global Household Air Pollution (HAP) Measurements database from WHO contains 196 studies with measurements from 43 countries of various pollution metrics in households using solid fuel for cooking.[2] From this database, we take all measurements of $PM_{2.5}$ using indoor or personal monitors. In addition to the WHO database, we included eight additional studies from a systematic review conducted in 2015 for GBD.

The final dataset included 336 estimates from 75 studies in 43 unique locations. We included 260, 64, nine, and three measurements indoors, on personal monitors for females, children (under 5), and males, respectively. 274 estimates were in households using solid fuels, 47 in households only using clean (gas or electricity) fuels, and 15 in households using a mixture of solid and clean fuels.

We use the following model:

$$\log(excess\ PM) \sim solid + measure\ group + 24\ hr\ measurement + SDI + (1|study)$$

Where,

- 24-hour measurement: binary variable equal to 1 if the measurement occurred over at least a 24-hour period and not only during mealtimes
- Measure group: categorical variable indicating indoor, female, male, or children
- Solid: indicator variable equal to 1 if the measurements were among households using solid fuel only, 0.5 if the measurements represented a mix of clean and solid fuels, and 0 if the households only used clean fuels.

We also included the Socio-demographic Index (SDI) as a variable to predict a unique value of HAP for each location and year based on development. We also included a random effect on study. We weighted each study by its sample size.

Before modelling, we calculated the excess particulate matter in households using solid fuel by subtracting off the predicted ambient $PM_{2.5}$ value in the study location and year based on the GBD 2017 $PM_{2.5}$ exposure model. The final model coefficients are included below:

HAP mapping model and coefficients

Variable	Beta, log (95% CI)	Beta, adjusted (95% CI)
Intercept	6.23 (4.58, 7.88)	506 (97, 2635)
Solid	2.60 (2.06, 3.13)	13.4 (7.8, 23.0)
Measure group		
• Indoor (ref)		
• Female	-0.56 (-1.15, 0.04)	0.57 (0.32, 1.04)

• Male<br>• Child	-1.56 (-3.81, 0.70)<br>-1.13 (-2.06, -0.20)	0.21 (0.02, 2.02)<br>0.32 (0.13, 0.82)
24-hour measurement	-0.29 (-1.04, 0.46)	0.75 (0.35, 1.59)
SDI	-6.42 (-9.30, -3.54)	1.6 e -3 (9.1 e -5, 2.9 e -2)

Therefore, for females in households using solid fuel, we would expect their long-term mean excess $PM_{2.5}$ exposure due to the use of solid fuels to be 1522, 117, and 9 μg/m^3 in SDI of 0.1, 0.5, and 0.9, respectively.

Because there are so few studies of personal monitoring in men and children, rather than directly using the results of the model, we generated ratios using studies that measured at least two of the population groups for any size particulate matter. For $PM_{2.5}$ we used the predicted ambient $PM_{2.5}$ value in the study location and year based on the GBD 2017 $PM_{2.5}$ exposure model as the "outdoor" measurement, and for PM_4 and PM_{10} we used published values in the studies themselves. We first subtracted off this outdoor value from each PM measurement, and then calculated the ratio of male to female and child to female exposure, weighted by sample size.

Study	Location	Year	Pollutant	Female N	Female PM	Group	N	PM	Outdoor
Balakrishnan et al., 2004	Andhra Pradesh, Rural	2004	PM_4	591	352	male	503	187	94
Gao X et al., 2009.	Tibet	2009	$PM_{2.5}$	52	127	male	85	111	27
Dasgupta et al., 2006	Bangladesh	2006	PM_{10}	944	209	male	944	166	50
Devkumar et al., 2014	Nepal	2014	$PM_{2.5}$	405	169	male	429	167	90
Balakrishnan et al., 2004	Andhra Pradesh, Rural	2004	PM_4	591	352	child	56	262	94
Dionisio et al., 2008.	The Gambia	2008	$PM_{2.5}$	13	275	child	13	219	31
Dasgupta et al., 2006	Bangladesh	2006	PM_{10}	944	209	child	944	199	50

The final ratios were 0.64 95% CI (0.45, 0.91) for males and 0.85 95% CI (0.56, 1.31) for children. We used these results to scale the $PM_{2.5}$ mapping model for these age and sex groups to input into the $PM_{2.5}$ risk curves.

References

1. Smith KR, Bruce N, Balakrishnan K, Adair-Rohani H, Balmes J, Chafe Z, *et al*. Millions Dead: How Do We Know and What Does It Mean? Methods Used in the Comparative Risk Assessment of Household Air Pollution. Annu Rev Public Health. 2014; **35**(1):185–206.
2. Shupler M, Balakrishnan K, Ghosh S, *et al*. Global household air pollution database: Kitchen concentrations and personal exposures of particulate matter and carbon monoxide. Data in Brief 2018; **21**: 1292–5.
3. Shupler M, Godwin W, Frostad J, Gustafson P, Arku RE, Brauer M. Global estimation of exposure to fine particulate matter (PM2.5) from household air pollution. Environment International 2018; **120**: 354–63.
4. Tanchangya J, Geater AF. Use of traditional cooking fuels and the risk of young adult cataract in rural Bangladesh: a hospital-based case-control study. *BMC Ophthalmology* 2011; **11**.

Ambient particulate matter pollution

Flowchart

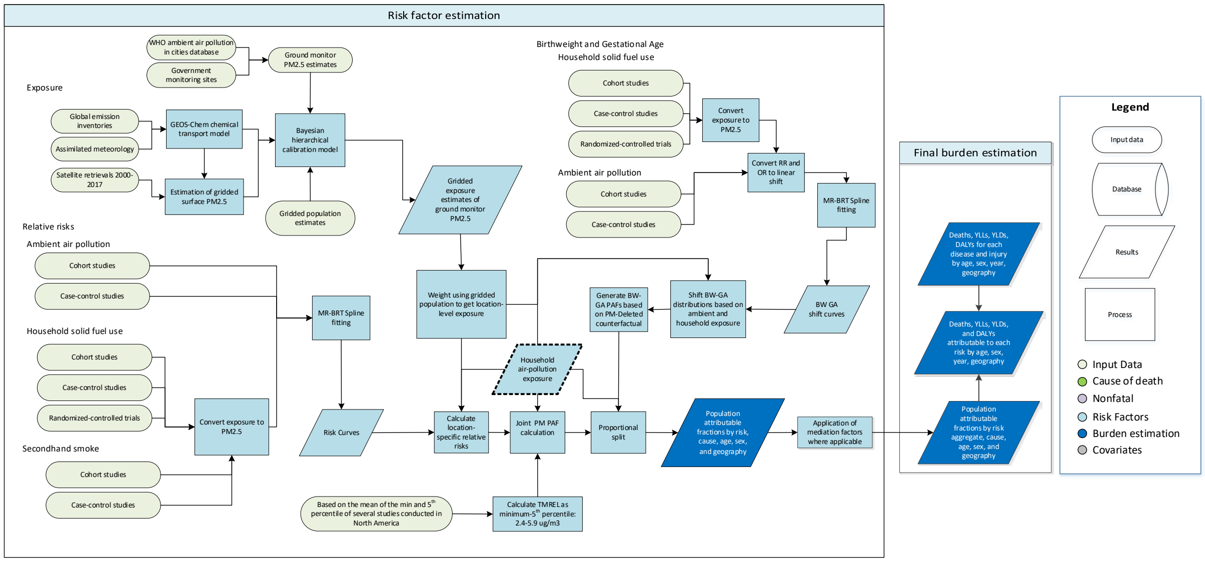

Input data and modelling strategy

Exposure

Definition

Exposure to ambient particulate matter pollution is defined as the population-weighted annual average mass concentration of particles with an aerodynamic diameter less than 2.5 micrometers ($PM_{2.5}$) in a cubic meter of air. This measurement is reported in $\mu g/m^3$.

Input data

The data used to estimate exposure to ambient particulate matter pollution comes from multiple sources, including satellite observations of aerosols in the atmosphere, ground measurements, chemical transport model simulations, population estimates, and land-use data. Table 1 summarizes exposure input data.

Table 1: Exposure Input Data

Input data	Exposure
Source count (total)	663
Number of countries with data	114

The following details the updates in methodology and input data used in GBD 2019.

$PM_{2.5}$ ground measurement database

Ground measurements used for GBD 2019 include updated measurements from sites included in 2017 and additional measurements from new locations. New and up-to-date data (mainly from the USA, Canada, EU, Bangladesh, China

and USA embassies and consulates), were added to the data from the 2018 update of the WHO Global Ambient Air Quality Database used in GBD 2017. The updated data included measurements of concentrations of PM_{10} and $PM_{2.5}$ from 10,408 ground monitors from 116 countries from 2010 to 2017. The majority of measurements were recorded in 2016 and 2017 (as there is a lag in reporting measurements, few data from 2018 or newer were available). Annual averages were excluded if they were based on less than 75% coverage within a year. If information on coverage was not available, then data were included unless there were already sufficient data within the same country (monitor density greater than 0.1).

For locations measuring only PM_{10}, $PM_{2.5}$ measurements were estimated from PM_{10}. This was performed using a hierarchy of conversion factors ($PM_{2.5}/PM_{10}$ ratios): (i) for any location a 'local' conversation factor was used, constructed as the ratio of the average measurements (of $PM_{2.5}$ and PM_{10}) from within 50km of the location of the PM_{10} measurement, and within the same country, if such measurements were available; (ii) if there was not sufficient local information to construct a conversion factor then a country-wide conversion factor was used; and (iii) if there was no appropriate information within a country, then a regional factor was used. In each case, to avoid the possible effects of outliers in the measured data (both $PM_{2.5}$ and PM_{10}), extreme values of the ratios were excluded (defined as being greater/lesser than the 95% and 5% quantiles of the empirical distributions of conversion factors). As with GBD 2013, 2015, 2016, and 2017 databases, in addition to values of $PM_{2.5}$ and whether they were direct measurement or converted from PM_{10}, the database also included additional information, where available, related to the ground measurements such as monitor geo-coordinates and monitor site type.

Satellite-based estimates
The global geophysical $PM_{2.5}$ estimates for the years 2000–2017 are from Hammer and colleagues Version V4.GL.03.NoGWR used at 0.1°x0.1° resolution (~11 x 11 km resolution at the equator).[1] The method is based on the algorithms of van Donkelaar and colleagues (2016) as used in GBD 2017,[2] with updated satellite retrievals, chemical transport modelling, and ground-based monitoring. The algorithm uses aerosol optical depth (AOD) from several updated satellite products (MAIAC, MODIS C6.1, and MISR v23), including finer resolution, increased global coverage, and improved long-term stability. Ground-based observations from a global sunphotometer network (AERONET version 3) are used to combine different AOD information sources. This is the first time that data from MAIAC at 1 km resolution was used to estimate $PM_{2.5}$ at the global scale. The GEOS-Chem chemical transport model with updated algorithms was used for geophysical relationships between surface $PM_{2.5}$ and AOD. Updates to the GEOS-Chem simulation included improved representation of mineral dust and secondary organic aerosol, as well as updated emission inventories. The resultant geophysical $PM_{2.5}$ estimates are highly consistent with ground monitors worldwide (R^2=0.81, slope = 1.03, n = 2541).

Population data
A comprehensive set of population data, adjusted to match UN2015 Population Prospectus, on a high-resolution grid was obtained from the Gridded Population of the World (GPW) database. Estimates for 2000, 2005, 2010, 2015, and 2020 were available from GPW version 4, with estimates for 1990 and 1995 obtained from the GPW version 3. These data are provided on a 0.0083°× 0.0083° resolution. Aggregation to each 0.1°×0.1° grid cell was accomplished by summing the central 12 × 12 population cells. Populations estimates for 2001–2004, 2006–2009, 2011–2014 and 2016–2019 were obtained by interpolation using natural splines with knots placed at 2000, 2005, 2010, 2015, and 2020. This was performed for each grid cell.

Chemical transport model simulations

Estimates of the sum of particulate sulfate, nitrate, ammonium, and organic carbon and the compositional concentrations of mineral dust simulated using the GEOS Chem chemical transport model, and a measure combining elevation and the distance to the nearest urban land surface (as described in van Donkelaar and colleagues 2016[2] and Hammer and colleagues (submitted))[1] were available for 2000–2017 for each 0.1°×0.1° grid cell.

Modelling strategy

The following is a summary of the modelling approach, known as the Data Integration Model for Air Quality (DIMAQ) used in GBD 2015, 2016, 2017, and now in GBD 2019.[3,4]

Before the implementation of DIMAQ (ie, in GBD 2010 and GBD 2013), exposure estimates were obtained using a single global function to calibrate available ground measurements to a "fused" estimate of $PM_{2.5}$; the mean of satellite-based estimates and those from the TM5 chemical transport model, calculated for each 0.1°×0.1° grid cell. This was recognised to represent a tradeoff between accuracy and computational efficiency when utilising all the available data sources. In particular, the GBD 2013 exposure estimates were known to underestimate ground measurements in specific locations (see discussion in Brauer and colleagues, 2015).[5] This underestimation was largely due to the use of a single, global calibration function, whereas in reality the relationship between ground measurements and other variables will vary spatially.

In GBD 2015 and GBD 2016, coefficients in the calibration model were estimated for each country. Where data were insufficient within a country, information can be "borrowed" from a higher aggregation (region) and, if enough information is still not available, from an even higher level (super-region). Individual country-level estimates were therefore based on a combination of information from the country, its region, and its super-region. This was implemented within a Bayesian hierarchical modelling (BHM) framework. BHMs provide an extremely useful and flexible framework in which to model complex relationships and dependencies in data. Uncertainty can also be propagated through the model, allowing uncertainty arising from different components, both data sources and models, to be incorporated within estimates of uncertainty associated with the final estimates. The results of the modelling comprise a posterior distribution for each grid cell, rather than just a single point estimate, allowing a variety of summaries to be calculated. The primary outputs here are the median and 95% credible intervals for each grid cell. Based on the availability of ground measurement data, modelling and evaluation were focused on the year 2016.

The model used in GBD 2017 and GBD 2019 also included within-country calibration variation.[6] The model used for GBD 2019, henceforth referred to as DIMAQ2, provides a number of substantial improvements over the initial formulation of DIMAQ. In DIMAQ, ground measurements from different years were all assumed to have been made in the primary year of interest and then regressed against values from other inputs (eg, satellites, etc.) made in that year. In the presence of changes over time, therefore, and particularly in areas where no recent measurements were available, there was the possibility of mismatches between the ground measurements and other variables. In DIMAQ2, ground measurements were matched with other inputs (over time), and the (global-level) coefficients were allowed to vary over time, subject to smoothing that is induced by a first-order random walk process. In addition, the manner in which spatial variation can be incorporated within the model has developed: where there are sufficient data, the calibration equations can now vary (smoothly) both within and between countries, achieved by allowing the coefficients to follow (smooth) Gaussian processes. Where there are insufficient data within a country, to produce accurate equations, as before, information is borrowed from lower down the hierarchy and it is supplemented with information from the wider region.

DIMAQ2 as described above is used for all regions except for the north Africa and Middle East and sub-Saharan Africa super-regions, where there are insufficient data across years to allow the extra complexities of the new model to be implemented. In these super-regions, a simplified version of DIMAQ2 is used in which the temporal component is dropped.

Model evaluation

Model development and comparison was performed using within- and out-of-sample assessment. In the evaluation, cross-validation was performed using 25 combinations of training (80%) and validation (20%) datasets. Validation sets were obtained by taking a stratified random sample, using sampling probabilities based on the cross-tabulation of $PM_{2.5}$ categories (0-24.9, 25-49.9, 50-74.9, 75-99.9, 100+ μg/m^3) and super-regions, resulting in them having the same distribution of $PM_{2.5}$ concentrations and super-regions as the overall set of sites. The following metrics were calculated for each training/evaluation set combination: for model fit – R^2 and deviance information criteria (DIC, a measure of model fit for Bayesian models); for predictive accuracy – root mean squared error (RMSE) and population weighted root mean squared error (PwRMSE). The median R^2 was 0.9, and the median PwRMSE was 10.1 μg/m^3.

All modelling was performed on the log-scale. The choice of which variables were included in the model was made based on their contribution to model fit and predictive ability. The following is a list of variables and model structures that were included in DIMAQ.

Continuous explanatory variables:

- (SAT) Estimate of $PM_{2.5}$ (in μg/m^3) from satellite remote sensing on the log-scale.
- (POP) Estimate of population for the same year as SAT on the log-scale.
- (SNAOC) Estimate of the sum of sulfate, nitrate, ammonium, and organic carbon simulated using the GEOS Chem chemical transport model.
- (DST) Estimate of compositional concentrations of mineral dust simulated using the GEOS-Chem chemical transport model.
- (EDxDU) The log of the elevation difference between the elevation at the ground measurement location and the mean elevation within the GEOS Chem simulation grid cell multiplied by the inverse distance to the nearest urban land surface.

Discrete explanatory variables:

- (LOC) Binary variable indicating whether exact location of ground measurement is known.
- (TYPE) Binary variable indicating whether exact type of ground monitor is known.
- (CONV) Binary variable indicating whether ground measurement is $PM_{2.5}$ or converted from PM_{10}.

Interactions*:*

- Interactions between the binary variables and the effects of SAT.

Random effects:

- Regional temporal (random walk) hierarchical random-effects on the intercept
- Regional hierarchical random-effects for the coefficient associated with SAT
- Regional hierarchical random-effects for the coefficient associated with POP
- Smoothed, spatially varying random-effects for the intercept
- Smoothed, spatially varying random-effects for the coefficient associated with SAT

Inference and prediction

Due to both the complexity of the models and the size of the data, notably the number of spatial predictions that are required, recently developed techniques that perform "approximate" Bayesian inference based on integrated nested Laplace approximations (INLA) were used.[7] Computation was performed using the R interface to the INLA computational engine (R-INLA). GBD 2019 also makes use of an innovation in the way that samples from the (Bayesian) model are used to represent distributions of estimated concentrations in each grid-cell. Here estimates, and distributions representing uncertainty, of concentrations for each grid are obtained by taking repeated (joint) samples from the posterior distributions of the parameters and calculating estimates based on a linear combination of those samples and the input variables.[8]

DIMAQ2 was used to produce estimates of ambient $PM_{2.5}$ for 1990, 1995, and 2010–2019 by matching the gridded estimates with the corresponding coefficients from the calibration. As there is a lag in reporting ambient air pollution based quantities, the input variables were extrapolated (as in GBD 2017), allowing estimates for 2018 and 2019 to be produced in the same way as other years and, crucially, allowing measures of uncertainty to be produced within the BHM framework rather than by using post-hoc approximations.

Estimates from the satellites and the GEOS-Chem chemical transport model in 2018 and 2019 were produced by extrapolating estimates from 2000–2017 using generalised additive models,[9] on a cell-by-cell basis, except in those grid cells that saw a >100% increase between 2016 and 2017, in which case only the 2000–2016 estimates were used for extrapolating, in order to avoid unrealistic and/or unjustified extrapolation of trends. Population estimates for 2018 and 2019 were obtained by interpolation as described above.

Theoretical minimum-risk exposure level

The TMREL was assigned a uniform distribution with lower/upper bounds given by the average of the minimum and fifth percentiles of outdoor air pollution cohort studies exposure distributions conducted in North America, with the assumption that current evidence was insufficient to precisely characterise the shape of the concentration-response function below the fifth percentile of the exposure distributions. The TMREL was defined as a uniform distribution rather than a fixed value in order to represent the uncertainty regarding the level at which the scientific evidence was consistent with adverse effects of exposure. The specific outdoor air pollution cohort studies selected for this averaging were based on the criteria that their fifth percentiles were less than that of the American Cancer Society Cancer Prevention II (CPSII) cohort's fifth percentile of 8.2 based on Turner and colleagues (2016).[10] This criterion was selected since GBD 2010 used the minimum, 5.8, and fifth percentile solely from the CPS II cohort. The resulting lower/upper bounds of the distribution for GBD 2019 were 2.4 and 5.9. This has not changed since GBD 2015.

Relative risks and population attributable fractions

We create one set of cause-specific risk curves for both household air pollution and ambient air pollution as two different sources of $PM_{2.5}$. We used results from cohort and case-control studies of ambient $PM_{2.5}$ pollution, cohort studies, case-control studies, and randomised-controlled trials of household use of solid fuel for cooking, and cohort and case-control studies of secondhand smoke.

For GBD 2019, we made several important changes to the risk functions. Previously, we have used relative risk estimates for active smoking, converting cigarettes-per-day to $PM_{2.5}$ exposure in order to estimate the $PM_{2.5}$ relative risk at the highest end of the $PM_{2.5}$ exposure-response curve. We took this approach because the vast majority of the air pollution epidemiological studies have been performed in low-pollution settings in high-income countries, preventing us from extrapolating the steep relationship at the beginning of the exposure range to locations with high exposure but no relative risk estimates, such as India and China. However, with the recent publication of studies in China and other higher-exposure settings and additional studies of HAP, we have been able to include more

estimates at high $PM_{2.5}$ levels in the model.[11,12,13,14,15] Furthermore, in contrast to previous cycles of the GBD where the power function used to develop the IER required the inclusion of active smoking data to anchor the risk function, with the current use of splines and their flexibility, it is easier to fit functions to the (ambient, household, and SHS) data without active smoking data. Beginning in GBD 2019, we excluded active smoking studies from the risk curves. Removal of active smoking information removes an important source of uncertainty in our earlier estimates related to differences in dose rates and other aspects of exposure between active smoking and the other $PM_{2.5}$ sources, including differences in voluntary (active smoking) and involuntary (ambient and household $PM_{2.5}$, secondhand smoke) exposure.[16,17]

Once we have a median age during follow-up (a), we extrapolate each study to the full set of ages where the estimated datapoint for age, a_j, is calculated with the following equation and accompanying explanatory figure:

$$\log(RR)_{a_j} = \frac{\log(RR)_a - 0}{a - 110} * (a_j - 110)$$

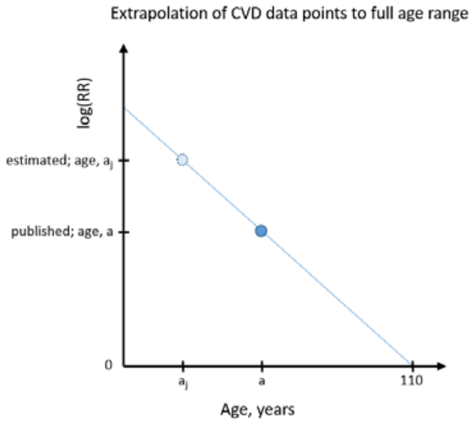

Previously we have used a fixed functional form to fit the risk curves.[16] In GBD 2019, we used MR-BRT (described in detail elsewhere) splines to fit the risk data with a more flexible shape. While previously we built in the TMREL estimates into the model fitting, this year we have fit the curve beginning at zero exposure and incorporate the TMREL into the relative risk calculation process. This allows others to use our risk curves with whatever counterfactual level is of interest to them. Relative risk curves are available upon request.

When fitting the risk curves, we consider the published relative risk over a range of exposure data. For OAP studies, the relative risk informs the curve from the fifth to the 95th percentile of observed exposure. When this is not available in the published study, we estimate the distribution from the provided information (mean and standard deviation, mean and IQR, etc.). We scale the RR to this range.

For HAP studies, we allow each study to inform the curve from the Exp_{OAP} to Exp_{OAP}+Exp_{HAP}, where Exp_{OAP} is the GBD 2017 estimate of the ambient exposure level in the study location and year, and Exp_{HAP} is the GBD 2017 estimate of the excess exposure for those who use solid fuel for cooking in the study location and year.

For SHS studies, we updated our strategy of exposure estimation in GBD 2019. For the first time, we are also accounting for outdoor exposure. Similar to the approach used for HAP, we allow each study to inform the curve from the Exp_{OAP} to Exp_{OAP}+Exp_{SHS}, where Exp_{OAP} is the GBD 2017 estimate of the ambient exposure level in the study location and year, and Exp_{SHS} is an estimate of the excess exposure for those who experience secondhand smoke. This is estimated from the number of cigarettes smoked per smoker per day in a given location and year, estimated by the smoking team of GBD, and from a study in Sweden, which measured the $PM_{2.5}$ exposure in homes of smokers.[19] We

divided the household $PM_{2.5}$ exposure level by the average number of cigarettes smoked per smoker per day in Sweden over the study duration to estimate the SHS $PM_{2.5}$ exposure per cigarette (2.31 $\mu g/m^3$ [95% UI 1.53–3.39]). To calculate Exp_{SHS} we multiplied the estimated number of cigarettes per smoker per day by the average $PM_{2.5}$ exposures per cigarette to generate a predicted $PM_{2.5}$ exposure level.

MR-BRT risk splines

We fit splines on the datasets including studies of OAP, HAP, and SHS using the following functional form, where X and X_{CF} represent the range of exposure characterised by the effect size:

$$log\left(\frac{MRBRT(X)}{MRBRT(X_{CF})}\right) \sim \log(Published\ Effect\ Size)$$

For each of the risk-outcome pairs, we tested various model settings and priors in fitting the MR-BRT splines. The final models used third-order splines with two interior knots and a constraint on the right-most segment, forcing the fit to be linear rather than cubic. We used an ensemble approach to knot placement, wherein 100 different models were run with randomly placed knots and then combined by weighting based on a measure of fit that penalises excessive changes in the third derivative of the curve. Knots were free to be placed anywhere within the fifth and 95th percentile of the data, as long as a minimum width of 10% of that domain exists between them. We included shape constraints so that the risk curves were concave down and monotonically increasing, the most biologically plausible shape for the $PM_{2.5}$ risk curve. On the non-linear segments, we included a Gaussian prior on the third derivative of mean 0 and variance 0.01 to prevent over-fitting; on the linear segment, a stronger prior of mean 0 and variance 1e-6 was used to ensure that the risk curves do not continue to increase beyond the range of the data.

Table 2 summarizes relative risk input data for ambient particulate matter pollution and household air pollution.

Table 2: Relative Risk Input Data

Input data	Relative risk
Source count (total)	200
Number of countries with data	40

The following figures display risk curves for each outcome. The dashed line depicts the GBD 2017 IER including active smoking data, the dotted line depicts the GBD 2019 IER including active smoking data and updates to the AS and SHS exposure incorporation, and the solid line depicts the GBD 2019 MR-BRT curve without the inclusion of active smoking data. The grey shaded areas represent the 95% CI. The red box represents the TMREL area of the curve. On each page, the first figure depicts the typical range of outdoor exposure, whereas the second plot includes higher levels typical of household air pollution exposure.

Each point or number represents one study effect size. Each is plotted at the 95th percentile of the exposure distribution (OAP), the expected level of exposure for individual using solid fuel (HAP), or the expected level of exposure for individuals experiencing SHS. The relative risk is plotted relative to the predicted relative risk at the fifth percentile of exposure distribution (OAP), the expected (ambient only) level of exposure for individuals not using solid fuel (HAP), or the expected (ambient only) level of exposure for individuals not exposed to SHS. For example, a study predicting a relative risk of 1.5 for an exposure range of 10 to 20 would be plotted at (20, MRBRT(10)*1.5).

Arrows represent studies that would have been outside the range of the plot but have been moved to include on the figure.

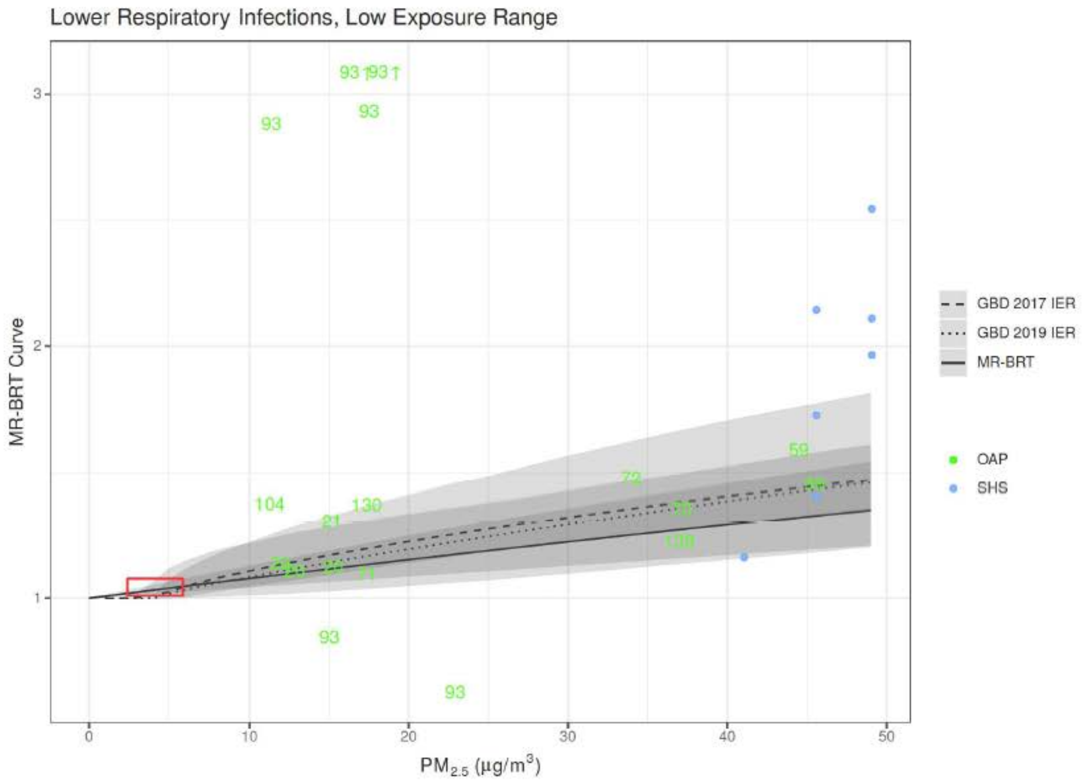

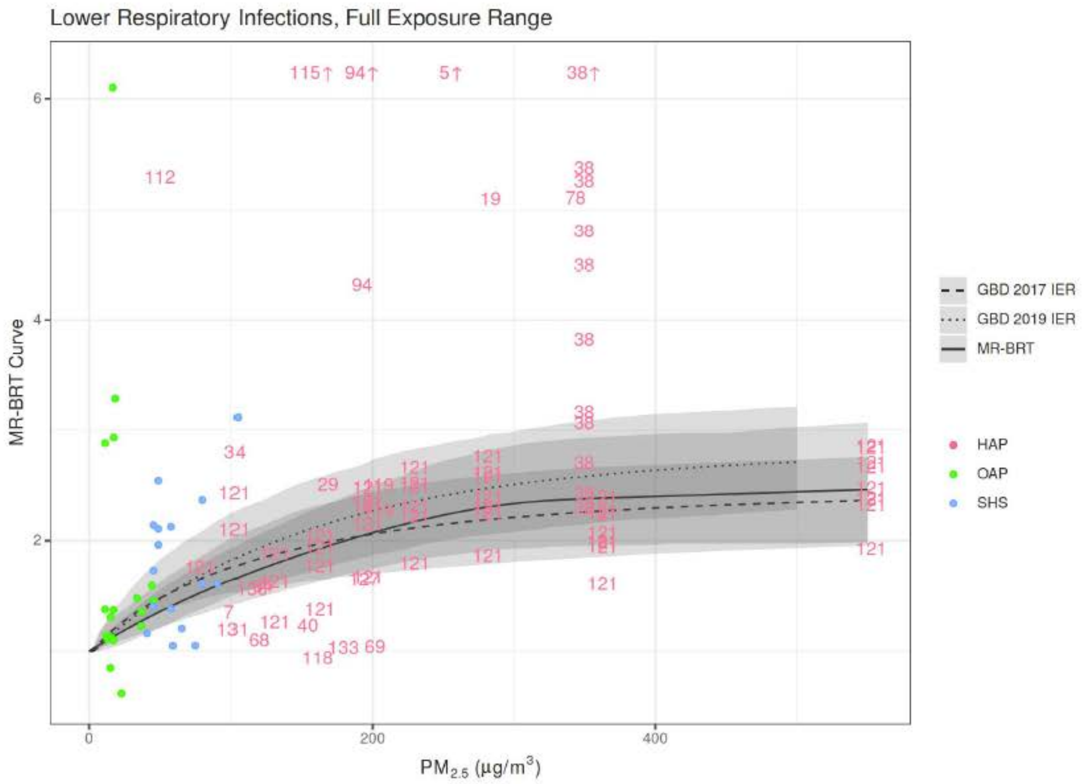

For lower respiratory infections, we have directly estimated PAFs attributable to $PM_{2.5}$ in addition to those mediated through birthweight and gestational age. We would expect that some of the directly estimated PAFs are mediated through birthweight and gestational age. Additionally, the directly estimated PAF is based on a summary of relative risks for all children under 5 years, so there is a chance that the mediated PAF, which is more finely resolved, could be greater. To avoid double-counting for these two age groups (0-6 days and 0-27 days), we take the max of the two PAF estimates. If the directly estimated PAF is greater than the birthweight-gestational age-mediated PAF, we take the direct estimate, and if the mediated PAF is greater, we take the mediated.

Limitations

Although in GBD 2019 we have not used active smoking data to estimate the risk curves, we are still using an integrated exposure response approach because we are integrating relative risk estimates across various exposure sources: ambient, SHS, and HAP. The use of various sources to construct a risk curve with $PM_{2.5}$ as the exposure indicator assumes equitoxicity of particles, despite some evidence suggesting differences in health impact by PM source, size, and chemical composition. However, in the absence of consistent and robust evidence of differential toxicity by source and sufficient estimates of source or composition-specific exposure-response relationships, integrating across OAP, SHS, and HAP studies is the approach most consistent with the current evidence, as reviewed by US EPA and WHO.[20,21] Use of a common risk function may affect the magnitude of risk estimates for HAP and OAP compared to separate risk functions. As more data from higher OAP concentration locations and from HAP studies for non-respiratory outcomes becomes available it may be possible to evaluate the strength of evidence for each and to develop separate risk functions.

Proportional PAF approach

Prior to GBD 2017, relative risks for both exposures were obtained from the IER as a function of exposure and relative to the same TMREL. In reality, were a country to reduce only one of these risk factors, the other would remain. We did not consider the joint effects of particulate matter from outdoor exposure and burning solid fuels for cooking. For GBD 2017 we developed a new approach to use the IER for obtaining PAFs for both OAP and HAP:

Let Exp_{OAP} be the ambient $PM_{2.5}$ exposure level and Exp_{HAP} be the excess exposure for those who use solid fuel for cooking. Let P_{HAP} be the proportion of the population using solid fuel for cooking. We calculated PAFs at each $0.1^{o}\times0.1^{o}$ grid cell. We assumed that the distribution of those using solid fuel for cooking (HAP) was equivalent across all grid cells of the GBD location.

For the proportion of the population not exposed to HAP the relative risk was:

$$RR_{OAP} = MRBRT(z = Exp_{OAP})/MRBRT(z = TMREL),$$

And for those exposed to HAP, the relative risk was

$$RR_{HAP} = MRBRT(z = Exp_{OAP} + Exp_{HAP})/MRBRT(z = TMREL).$$

We then calculate a population level RR and PAF for all particulate matter exposure.

$$RR_{PM} = RR_{OAP}(1 - P_{HAP}) + RR_{HAP}P_{HAP}$$

$$PAF_{PM} = \frac{RR_{PM} - 1}{RR_{PM}}$$

We population weight the grid-cell level particulate matter PAFs to get a country level PAF, and finally, we split this PAF based on the average exposure to each OAP and HAP.

$$PAF_{OAP} = \frac{Exp_{OAP}}{Exp_{OAP}+P_{HAP}*Exp_{HAP}} PAF_{PM}, \text{ and } PAF_{HAP} = \frac{P_{HAP}*Exp_{HAP}}{Exp_{OAP}+P_{HAP}*Exp_{HAP}} PAF_{PM}.$$

With this strategy, $PAF_{PM} = PAF_{HAP} + PAF_{OAP}$, and no burden is counted twice.

References

1. Hammer, M. S., A. van Donkelaar, R. V. Martin, C. Li, A. Lyapustin, A. M. Sayer, C. N. Hsu, R. C. Levy, M. J. Garay, O. V. Kalashnikova, R. A. Kahn, M. Brauer, J. S. Apte, D. K. Henze, L. Zhang, and Q. Zhang (submitted), Improved Global Estimates of Fine Particulate Matter Concentrations and Trends Derived from Updated Satellite Retrievals, Modeling Advances, and Additional Ground-Based Monitors, *Environ. Sci. Technol*.
2. van Donkelaar, A.; Martin, R. V; Brauer, M.; Hsu, N. C.; Kahn, R. A.; Levy, R. C.; Lyapustin, A.; Sayer, A. M.; Winker, D. M. Global Estimates of Fine Particulate Matter using a Combined Geophysical-Statistical Method with Information from Satellites, Models, and Monitors. Environ. Sci. Technol. 2016, 50 (7), 3762–3772
3. Shaddick, G., Thomas, M.L., Jobling, A., Brauer, M., van Donkelaar, A., Burnett, R., Chang, H., Cohen, A., Van Dingenen, R., Dora, C. and Gumy, S., 2016. Data Integration Model for Air Quality: A Hierarchical Approach to the Global Estimation of Exposures to Ambient Air Pollution. Journal of Royal Statistical Society Series C (Applied Statistics). 2017. DOI: 10.1111/rssc.12227
4. Shaddick, G., Thomas, M. L., Mudu, P., Ruggeri, G. and Gumy, S. Half the world's population are exposed to increasing air pollution. Accepted by Nature Climate and Atmospheric Science.
5. Brauer, M.; Freedman, G.; Frostad, J.; van Donkelaar, A.; Martin, R. V; Dentener, F.; Van Dingenen, R.; Estep, K.; Amini, H.; Apte, J. S.; et al. Ambient Air Pollution Exposure Estimation for the Global Burden of Disease 2013. Environ. Sci. Technol. 2015, 50 (1), 79–88.
6. Shaddick G, Thomas M, Amini H, Broday DM, Cohen A, Frostad J, Green A, Gumy S, Liu Y, Martin RV, Prüss-Üstün A, Simpson D, van Donkelaar A, Brauer M. Data integration for the assessment of population exposure to ambient air pollution for global burden of disease assessment. Environ Sci Technol. 2018 Jun 29. doi: 10.1021/acs.est.8b02864
7. Rue, H.; Martino, S.; Chopin, N.; Approximate Bayesian inference for latent Gaussian models by using integrated nested Laplace approximations. Journal of the royal statistical society: Series b (statistical methodology). 2009;71(2):319-92.
8. Thomas, M. L., Shaddick, G., Simpson, D., de Hoogh, K. and Zidek, J. V. Spatio-temporal downscaling for continental–scale estimation of air pollution concentrations. arXiv preprint arXiv:1907.00093 (also been Submitted to the Journal of the Royal Statistical Society: Series C (Applied Statistics)).
9. Wood, S. N. (2017). Generalized additive models: an introduction with R. Chapman and Hall/CRC.
10. Turner MC,Jerrett M, Pope CA 3rd, Krewski D, Gapstur SM, Diver WR, Beckerman BS, Marshall JD, Su J, Crouse DL, Burnett RT. Long-term ozone exposure and mortality in a large prospective study . Am J Respir Crit Care Med. 2016; 193(10): 1134-42.
11. Yin P, Brauer M, Cohen A, et al. Long-term Fine Particulate Matter Exposure and Nonaccidental and Cause-specific Mortality in a Large National Cohort of Chinese Men. Environ Health Perspect 2017; 125: 117002.
12. Li T, Zhang Y, Wang J, et al. All-cause mortality risk associated with long-term exposure to ambient PM2·5 in China: a cohort study. Lancet Public Health 2018; 3: e470–7.
13. Yang Y, Tang R, Qiu H, et al. Long term exposure to air pollution and mortality in an elderly cohort in Hong Kong. Environ Int 2018; 117: 99–106.
14. Hystad P, Larkin A, Rangarajan S, AlHabib KF, Avezum A, Tumerdem Calik KB; Chifamba J, Dans A, Diaz R, du Plessis JL, Gupta R, Iqbal R, Khatib R, Kelishadi R, Lanas F, Liu Z, Lopez-Jaramillo P, Nair S, Poirier P, Rahman O, Rosengren A, Swidan H, Tse L-A, Wei L, Wielgosz A, Yeates K, Yusoff K, Zatoński T, Yusuf S, Brauer M. Outdoor fine particulate matter air pollution and cardiovascular disease: Results from 747 communities across 21 countries in the PURE Study. (Submitted to Lancet Global Health)
15. Joseph P, Rangarajan S, Islam S, Mente A, Hystad P, Brauer M, Raman Kutty V, Gupta R, Wielgosz A, AlHabib KF, Dans A, Lopez-Jaramillo P, Avezum A, Lanas F, Oguz A, Kruger IM, Diaz R, Yusoff K, Mony P, Chifamba J,

Yeates K, Kelishadi R, Yusufali A, Khatib R, Rahman O, Zatonska K, Iqbal R, Wei L, Bo H, Rosengren A, Kaur M, Mohan V, Lear SA, Teo KK, O'Donnell M, McKee M, Dagenais G, Yusuf S. Modifiable risk factors, cardiovascular disease and mortality in 155,722 individuals from 21 high-, middle-, and low-income countries (PURE): a prospective cohort study. The Lancet. 2019. doi:10.1016/S0140-6736(19)32008-2

16. Burnett RT, Pope CA 3rd, Ezzati M, Olives C, Lim SS, Mehta S, Shin HH, Singh G, Hubbell B, Brauer M, Anderson HR, Smith KR, Balmes JR, Bruce NG, Kan H, Laden F, Prüss-Ustün A, Turner MC, Gapstur SM, Diver WR, Cohen A. An integrated risk function for estimating the global burden of disease attributable to ambient fine particulate matter exposure. *Environ Health Perspect*. 2014; 122(4): 397-403.
17. Pope CA III, Cohen AJ, Burnett RT. Cardiovascular Disease and Fine Particulate Matter: Lessons and Limitations of an Integrated Exposure Response Approach. Circulation Research. 2018;122:1645-1647.
18. Lind L, Sundström J, Ärnlöv J, Lampa E. Impact of Aging on the Strength of Cardiovascular Risk Factors: A Longitudinal Study Over 40 Years. *J Am Heart Assoc.* 2018;7(1):e007061. Published 2018 Jan 6. doi:10.1161/JAHA.117.007061
19. Semple S, Apsley A, Ibrahim TA, Turner SW, Cherrie JW. Fine particulate matter concentrations in smoking households: just how much secondhand smoke do you breathe in if you live with a smoker who smokes indoors? *Tob Control* 2015; 24: e205–11.
20. US Environmental Protection Agency. Integrated science assessment (ISA) for particulate matter (Final Report, Dec 2009). EPA/600/R-08/139F, 2009. Washington, DC: US Environmental Protection Agency; 2009. Available at: http://cfpub.epa.gov/ncea/risk/recordisplay.cfm?deid=216546
21. World Health Organization. Review of evidence on health aspects of air pollution – REVIHAAP Project technical report. Copenhagen: WHO Regional Office for Europe; 2013. Available at: http://www.euro.who.int/__data/assets/pdf_file/0004/193108/REVIHAAP-Final-technical-report-final-version.pdf?ua=1

Secondhand smoke

Flowchart

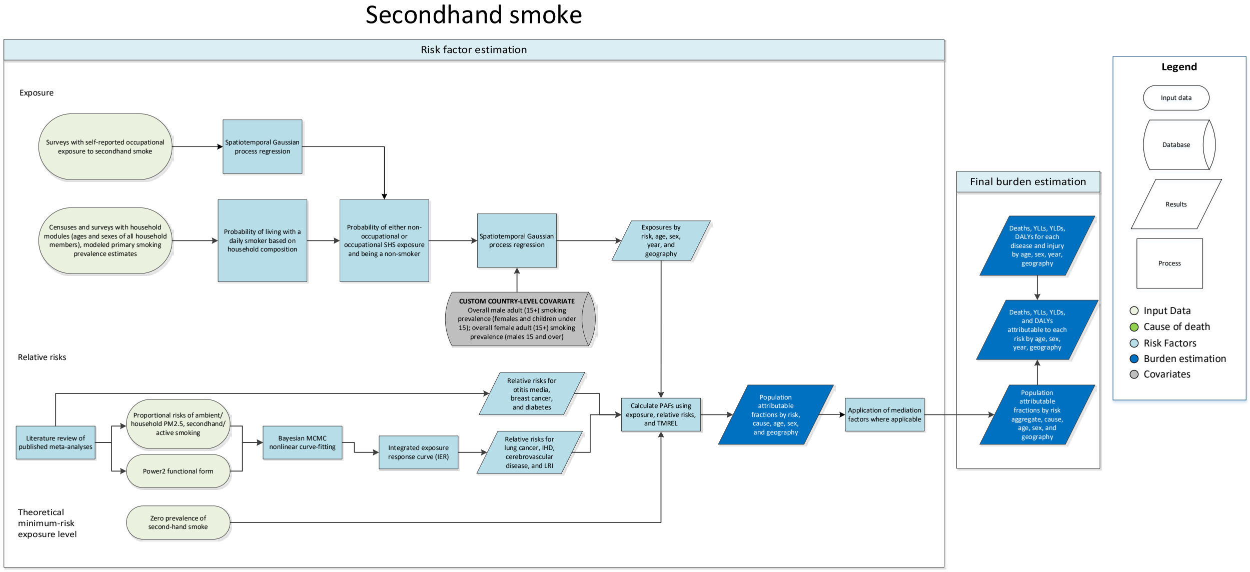

Exposure

Case definition

We define secondhand smoke exposure as current exposure to secondhand tobacco smoke at home, at work, or in other public places. We use household composition as a proxy for non-occupational secondhand smoke exposure and make the assumption that all persons living with a daily smoker are exposed to tobacco smoke. We use surveys to estimate the proportion of individuals exposed to secondhand smoke at work. We only consider non-smokers to be exposed to secondhand smoke. Non-smokers are defined as all persons who are not daily smokers. Ex-smokers and occasional smokers are considered non-smokers in this analysis. Exposure is evaluated for both children and adults.

Input data

To calculate the proportion of non-smokers who live with at least one smoker, we used unit record data on household composition, which included the ages and sexes of all persons living in the same household. Our sources included representative major survey series with a household composition module, including the Demographic Health Surveys (DHS), the Multiple Indicator Cluster Surveys (MICS), and the Living Standards Measurement Surveys (LSMS); and national and subnational censuses, which included those captured in the IPUMS project and identified using the Global Health Data Exchange catalog (GHDx).

To calculate the proportion of individuals exposed to secondhand smoke at work, by age and sex, we used cross-sectional surveys that ask respondents about self-reported occupational secondhand smoke exposure. Sources include the Global Adult Tobacco Surveys, Eurobarometer Surveys, and WHO STEPS Surveys. We identified sources using the GHDx.

No major changes have been introduced to data inputs since 2016. A new systematic review is planned for the next GBD round. Table 1 summarizes exposure input data.

Input data	Exposure
Source count (total)	721
Number of countries with data	153

Given the nature of the data used in our models (microdata), no crosswalk for case definition adjustment or age- and sex-splitting processes were required. Estimates of daily smoking prevalence in each location were also used in our calculations, as described in the modelling strategy section below.

Modelling strategy

Identical to GBD 2017, we estimated the probability that each person is living with a smoker and is also a non-smoker themselves using set theory. First, household composition data were used at the individual level to capture the ages and sexes of each person in the household. Second, we analysed surveys with both household composition data and tobacco use questions and determined that the distribution of household size, mean age of the household members, and the age distribution were not significantly different between households with and without a self-reported smoker. Since we did not find that household composition varied between smokers and non-smokers, we then used the GBD 2019 primary daily smoking prevalence model to calculate the probability that each household member is a daily smoker. Next, we used the probability of the union of sets on each individual household member to calculate the overall probability that at least one of the other household members was a daily smoker. As in GBD 2017, we incorporated occupational exposure by modelling prevalence of current exposure to secondhand smoke at work, by age, sex, location, and year, using ST-GPR. In order to avoid double counting we calculated the probability that an individual is exposed through either non-occupational exposure or occupational exposure, given their age, sex, and household composition. Finally, we multiplied this probability of exposure by the probability that the individual is not a smoker themselves (ie, 1 minus primary daily smoking prevalence for that person's location, year, age, and sex). We then collapsed these individual-level probabilities to produce average probabilities of exposure by location, year, age, and sex.

These probabilities were modelled in the GBD ST-GPR framework, which generates exposure estimates from a mixed effects hierarchical linear model plus weighted residuals smoothed across time, space, and age. The linear model formula was fit separately by sex using restricted maximum likelihood in R.

We used the sex-specific overall daily smoking prevalence for adults (age 15 and older) as a country-level covariate in the model. The overall male adult daily smoking prevalence was used as the covariate for females of all ages and for males under age 15. The overall female adult daily smoking prevalence was used as the covariate for males age 15 and older.

All input datapoints from the probability calculation had a measure of uncertainty (variance and sample size) coming from the uncertainty of the primary smoking prevalence model and the sample size from the unit record data going into the modelling process. Geographical r*andom effects were used in model fitting but were not used in prediction.*

Theoretical minimum-risk exposure level

The theoretical minimum-risk exposure level for secondhand smoke is zero exposure among non-smokers, meaning that non-smokers would not live with any primary smokers.

Relative risks

The same risk-outcome pairs from GBD 2017 were used. For LRI, we used country-specific relative risks created using integrated exposure response curves (IER) for $PM_{2.5}$ air pollution. IER curve calculation was updated with the GBD 2019 cigarettes per smoker estimates. Table 2 summarizes relative risk input data.

**Input data**	**Exposure**
Source count (total)	232
Number of countries with data	34

We used the standard GBD population attributable fraction (PAF) equation to estimate burden based on exposure and relative risks.

References

1. Jones LL, Hassanien A, Cook DG, Britton J, Leonardi-Bee J. Parental smoking and the risk of middle ear disease in children. Arch Pediatr Adolesc Med. 2012; 166: 18–27.
2. Macacu A, Autier P, Boniol M, Boyle P. Active and passive smoking and risk of breast cancer: a meta-analysis. Breast Cancer Res Treat 2015; 154:213–224.
3. Zhu B, Wu X, Wang X, Zheng Q, Sun G. The association between passive smoking and type 2 diabetes: a meta-analysis. Asia-Pacific Journal of Public Health 2014; 26:226-237.

Low birthweight and short gestation

Flowchart

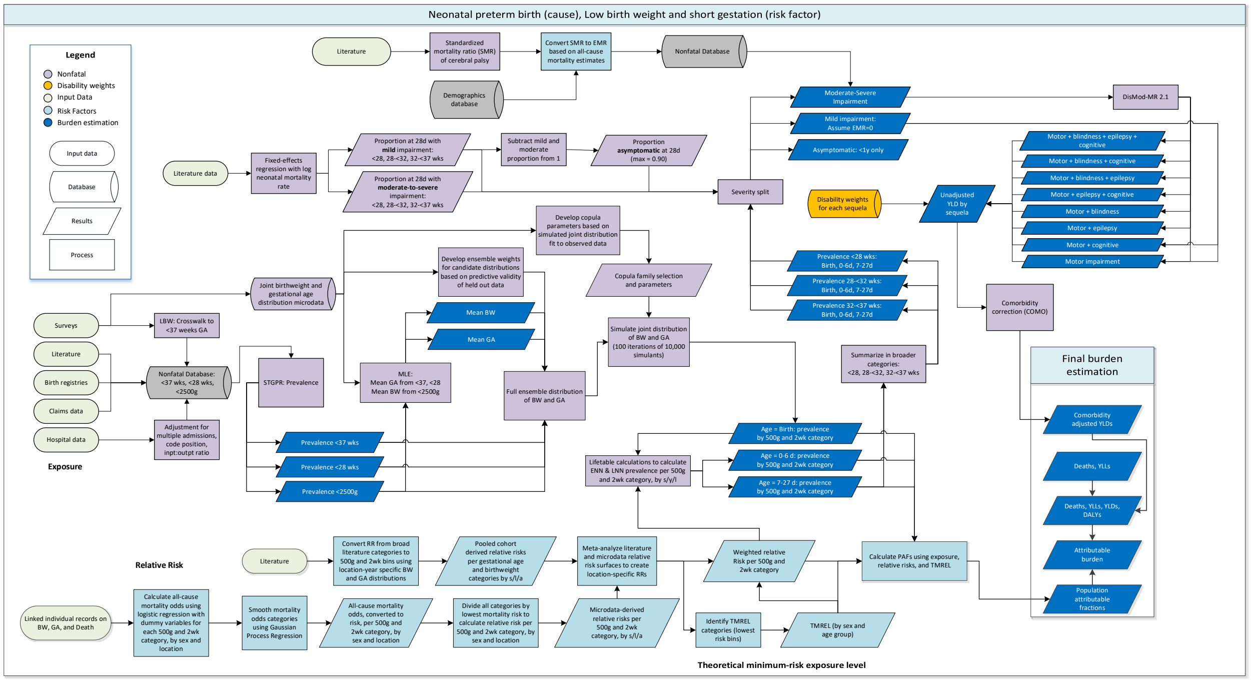

Exposure

Case definition

Low birthweight and short gestation is defined as birth at a gestational age less than the lowest-risk age (38 weeks) and at a lower birthweight than the lowest-risk weight (3500 grams). The TMREL is 38–42 weeks of gestation and birthweight of 3500–4500 grams.

Input data and methodological summary

Short gestational age and low birthweight are highly correlated risk factors associated with poor child health outcomes. The "low birthweight and short gestation" (LBWSG) risk factor quantifies the burden of disease attributable to increased risk of death and disability due to 1) less than ideal birthweight ("low birthweight") and 2) shorter than ideal length of gestation ("short gestation").

Generally, within GBD, attributable burden is estimated separately for each individual risk factor. However, within the global health community, the combined burden attributable to multiple risk factors is of general interest. In GBD, attributable burden due to multiple risk factors is typically estimated through a "mediation analysis" that is applied after independent estimation of each risk factor's exposure, relative risk, theoretical minimum risk exposure level (TMREL), and population attributable fraction (PAF). In the mediation analysis, a "mediation factor" adjusts the PAF of each risk factor by the amount of attributable burden mediated through the other GBD risk factors. While mediation analysis is the standard approach in GBD used to estimate combined attributable burden, direct quantification of the joint exposure, relative risk, and PAF of the combined risk factors is a conceptually more straightforward analysis. However, in practice, data informing joint exposure and relative risk of multiple risk factors is typically scarce.

In GBD 2016, LBWSG became the first (and, as of GBD 2019, only) group of GBD risk factors in which combined attributable burden is quantified by direct estimation of the joint exposure, relative risk, TMREL, and PAF of multiple risk factors. Compared to other risk factor groups in GBD, the data needed to estimate the joint exposure and joint relative risk of birthweight and gestational age are relatively abundant, as birthweight and gestational age are commonly reported together. LBWSG was also an appealing candidate to pilot joint direct estimation because the combined burden attributable to birthweight *and* gestational age, as well as the separately attributable burden due to birthweight *or* gestational age, are both of interest to the global health community.

After first directly estimating the joint exposure, relative risk, TMREL, and PAF of birthweight and gestational age together, we then separate out the independent PAFs due to birthweight only or gestational age only. Because of this modelling strategy, the joint GBD risk factor quantifying the burden of disease due to both less than ideal birthweight ("low birthweight") and shorter than ideal gestational age ("short gestation") is grouped into a single "parent" risk factor termed "low birthweight and short gestation". LBWSG is disaggregated into two "child" risk factors: "low birthweight for gestation" and "short gestation for birthweight". Low birthweight for gestation quantifies the burden of disease attributable to less than ideal birthweight, after adjusting for the influence of gestational age. Likewise, short gestation for birthweight quantifies the burden of disease attributable to shortened gestational age, after adjusting for the influence of birthweight.

Ideally, the model for joint exposure and joint relative risk would be fully continuous. To simplify the computation for the analysis, a grid of 500-gram and 2-week units ("bins") is used as the LBWSG dimensions and to approximate a fully continuous joint distribution model (see Figure 1).

Figure 2: Fully continuous analysis of joint gestational age and birthweight (left) is approximated with a grid of birthweight and gestational age with 500-gram and 2-week "bins" (right)

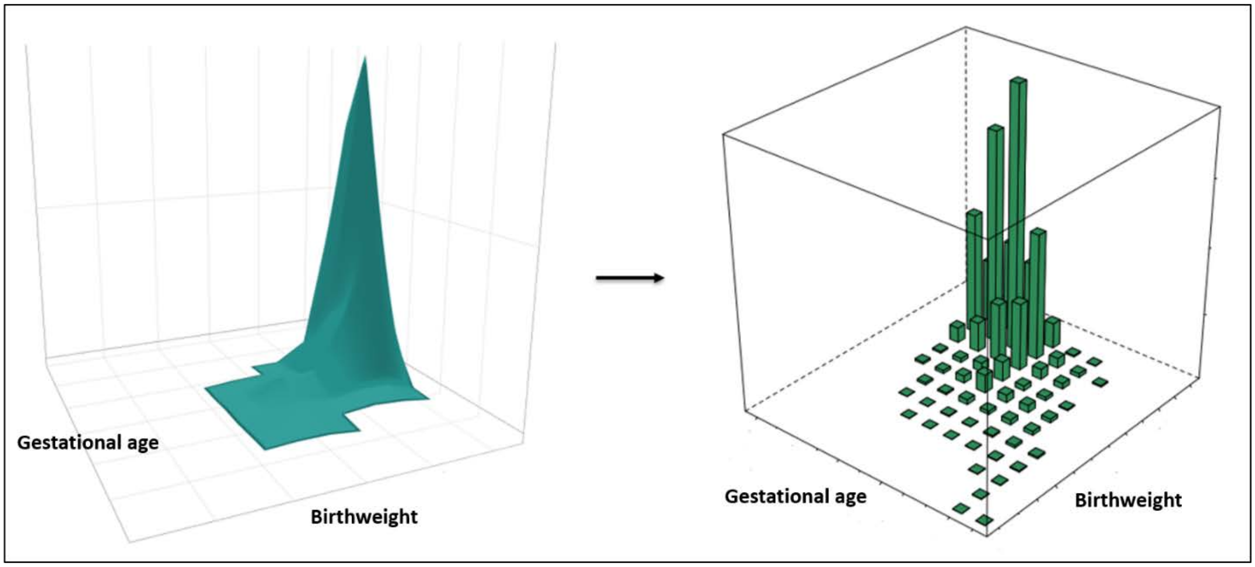

Case definition

"Low birthweight" has historically referred to any birthweight less than 2500 grams, dichotomising birthweight into two categories: "normal" and "low". In the context of the GBD LBWSG risk factor, low birthweight refers to any birthweight less than the birthweight TMREL (the birthweight that minimises risk at the population level). Because LBWSG is estimated in a grid of 500-gram and 2-week bins, any 500-gram birthweight unit less than the TMREL, which was determined as [38, 40) weeks and [3500, 4000) g for the LBWSG parent risk factor, is considered "low birthweight". This includes, for example, birthweight of [2500, 3000) grams, which the traditional, dichotomous definition of "low birthweight" would not include.

Like birthweight, gestational age is typically classified into broad categories. "Preterm" is used to describe any newborn baby born less than 37 completed weeks of gestation. In the GBD context, "short gestation" is used to refer to all gestational ages below the gestational age TMREL.

LBWSG is paired with the outcomes listed in Table 1 and is only attributed to burden in the early and late neonatal period.

Table 1: Cause list of outcomes for low birthweight and short gestation

Cause name
Diarrhoeal diseases
Lower respiratory infections
Upper respiratory infections
Otitis media
Pneumococcal meningitis
H influenzae type B meningitis
Meningococcal meningitis
Other meningitis

Encephalitis
Neonatal preterm birth complications
Neonatal encephalopathy due to birth asphyxia and trauma
Neonatal sepsis and other neonatal infections
Haemolytic disease and other neonatal jaundice
Other neonatal disorders
Sudden infant death syndrome

Exposure

In LBWSG, exposure refers to the portion of the joint distribution of gestational age and birthweight less than the TMREL, by location/year/sex (l/y/s), from birth to the end of the neonatal period. Modelling LBWSG exposure can be summarised in three steps:

A. Model univariate gestational age and birthweight distributions at birth, by l/y/s
B. Model joint distributions of gestational age and birthweight at birth, by l/y/s
C. Model joint distributions from birth to the end of the neonatal period, by l/y/s

Table 2: Analytic steps in estimation of YLDs due to preterm birth

	Summary of exposure modelling strategy
**Step A**<br>Model univariate distributions at birth	1. Model mean gestational age, prevalence of gestational age <28 weeks, and prevalence of gestational age <37 weeks, by l/y/s<br>2. Model mean birthweight and prevalence of birthweight <2500 grams, by l/y/s<br>3. Model univariate gestational age and birthweight distributions separately at birth, by l/y/s
**Step B**<br>Model joint distributions at birth	1. Use copulae to model the correlation structure of the joint distribution of gestational age and birthweight, globally<br>2. Model the joint distribution of gestational age and birthweight, by location/year/sex at birth, by applying the globally modelled correlation structure to the location/year/sex-specific univariate models of gestational age and birthweight distributions
**Step C**<br>Model joint distributions from birth to 28 days	1. Model all-cause mortality rates by gestational age and birthweight<br>2. Model gestational age and birthweight distributions of surviving neonates for all l/y/s from birth to end of the neonatal period, using all-cause mortality rates by gestational age and birthweight

Input data and data processing

Input data needed to model univariate gestational age and birthweight distributions at birth (Step A) are:

- Prevalence of preterm birth (<37 weeks), by l/y/s
- Prevalence of preterm birth (<28 weeks), by l/y/s
- Mean gestational age, by l/y/s
- Gestational age microdata

- Prevalence of low birthweight (<2500 grams), by l/y/s
- Mean birthweight, by l/y/s
- Birthweight microdata

To model joint distributions of gestational age and birthweight (Step B), joint microdata of gestational age and birthweight are also required. Additional inputs to modelling joint distributions from birth to 28 days (Step C) are all-cause mortality by l/y/s and joint birthweight and gestational age microdata linked to mortality outcomes.

Prevalence of extremely preterm birth (<28 weeks) and preterm birth (<37 weeks) were modelled using vital registration, survey, and clinical data. For the preterm models, only inpatient and insurance claims data were included from clinical informatics datasets; outpatient data were excluded because they were more likely to capture repeated visits by the same child rather than unique visits. Prevalence of low birthweight (<2500 grams) was modelled using only vital registration and survey data.

Literature review

Before GBD 2016, available preterm birth data were sourced by a technical working group. In GBD 2016 and GBD 2017, we conducted systematic reviews to identify additional sources beyond the data already used in the models. The PubMed database was searched using the following search string:

((("Infant, Premature"[Mesh] OR ("infant"[All Fields] AND "premature"[All Fields]) OR "premature infant"[All Fields] OR ("preterm"[All Fields] AND "infant"[All Fields]) OR "preterm infant"[All Fields] OR ("infant, newborn"[MeSH Terms] OR ("infant"[All Fields] AND "newborn"[All Fields]) OR "newborn infant"[All Fields] OR ("newborn"[All Fields] AND "infant"[All Fields])) AND (premature[All Fields] OR preterm[All Fields]) OR "premature birth"[MeSH Terms] OR ("premature"[All Fields] AND "birth"[All Fields]) OR "premature birth"[All Fields] OR ("preterm"[All Fields] AND "birth"[All Fields]) OR "preterm birth"[All Fields]) ((("Infant, Premature"[Mesh] OR ("infant"[All Fields] AND "premature"[All Fields]) OR "premature infant"[All Fields] OR ("preterm"[All Fields] AND "infant"[All Fields]) OR "preterm infant"[All Fields] OR ("infant, newborn"[MeSH Terms] OR ("infant"[All Fields] AND "newborn"[All Fields]) OR "newborn infant"[All Fields] OR ("newborn"[All Fields] AND "infant"[All Fields])) AND (premature[All Fields] OR preterm[All Fields]) OR "premature birth"[MeSH Terms] OR ("premature"[All Fields] AND "birth"[All Fields]) OR "premature birth"[All Fields] OR ("preterm"[All Fields] AND "birth"[All Fields]) OR "preterm birth"[All Fields]) AND ("1985"[PDAT] : "3000"[PDAT]) AND "humans"[MeSH Terms].

The exclusion criteria were: studies that did not provide primary data on epidemiological parameters, non-representative studies (eg, only high-risk pregnancies), and reviews. Table 3 shows the search hits, number of full-texts reviewed, and number of extracted sources.

Table 3. LBWSG search hits, full-text review, extracted sources

Search	Hits	Full-text review	Extracted	Search date
GBD 2017	16174	2200	154	6/6/2017

Table 4. Input data for exposure models

Input data	Exposure
Source count (total)	1695

Number of countries with data	161

Data processing

Starting in GBD 2019, as was the case with all other non-fatal analyses, we applied empirical age and sex ratios from previous GBD 2019 Decomposition 1 models to disaggregate observations that did not entirely fit in one GBD age category or sex. Ratios were determined by dividing the result for a specific age and sex by the result for the aggregate age and sex specified in a given observation. It is our intention to update this splitting process annually.

Low birthweight (<2500 grams) data were extracted from literature, vital registration systems, and surveys. DHS survey data were observed to have high missingness; to correct for the missingness, birthweight was imputed using the Amelia II (Version 1.7.6) package in R. Birthweight was predicted using standard Amelia imputation methods from the following variables also in the DHS surveys: urbanicity, sex, birthweight recorded on card, birth order, maternal education, paternal education, child age, child weight, child height, mother's age at birth, mother's weight, shared toilet facility, and household water treated.

"Crosswalking", or the process of reducing non-random bias by adjusting non-standard data to the likely value had the data been "gold-standard", was used to process data in the extremely preterm (<28 weeks) and preterm (<37 weeks) models. All preterm crosswalks were done using meta-regression – regularized, Bayesian, trimmed (MR-BRT). Insurance claims data in extremely preterm (<28 weeks) data were adjusted to vital registration data. Insurance claims data and inpatient data were also adjusted to vital registration in preterm (<37 weeks) conditions. The crosswalk for inpatient data had a spline on the prevalence of inpatient data. Once all claims and inpatient preterm (<37 weeks) data were adjusted, low birthweight data were crosswalked to post-claims and inpatient preterm (<37 weeks) data. If low birthweight data in countries that were 1) categorised as "data-rich" locations in cause-of-death modelling or had at least ten consecutive years of vital registration data recording gestational age and 2) had both preterm birth and low birthweight data, crosswalked low birthweight data were outliered so that the model was informed only by the gestational age data.

Table 5. MR-BRT VR-insurance claims crosswalk adjustment factor for extremely preterm birth (<28 weeks of gestation)

Data input	Reference or alternative case definition	Gamma	Beta coefficient, log<br><br>(95% CI)	Adjustment factor*
Vital registration	Reference	0.00	---	---
Insurance claims	Alt		-0.651 (-0.602, -0.699)	0.521 (0.500, 0.548)

**Adjustment factor is the transformed beta coefficient in normal space and can be interpreted as the factor by which the alternative case definition is adjusted to reflect what it would have been if measured as the reference.*

Table 6. MR-BRT VR-insurance claims crosswalk adjustment factor for preterm birth (<37 weeks of gestation)

Data input	Reference or alternative case definition	Gamma	Beta coefficient, log<br><br>(95% CI)	Adjustment factor*

Vital registration	Reference	0.16	---	---
Insurance claims	Alt		-0.728 (-0.705, -0.752)	0.483 (0.471, 0.494)

**Adjustment factor is the transformed beta coefficient in normal space and can be interpreted as the factor by which the alternative case definition is adjusted to reflect what it would have been if measured as the reference.*

Figure 2: MR-BRT clinical inpatient data crosswalk with spline on prevalence of preterm birth

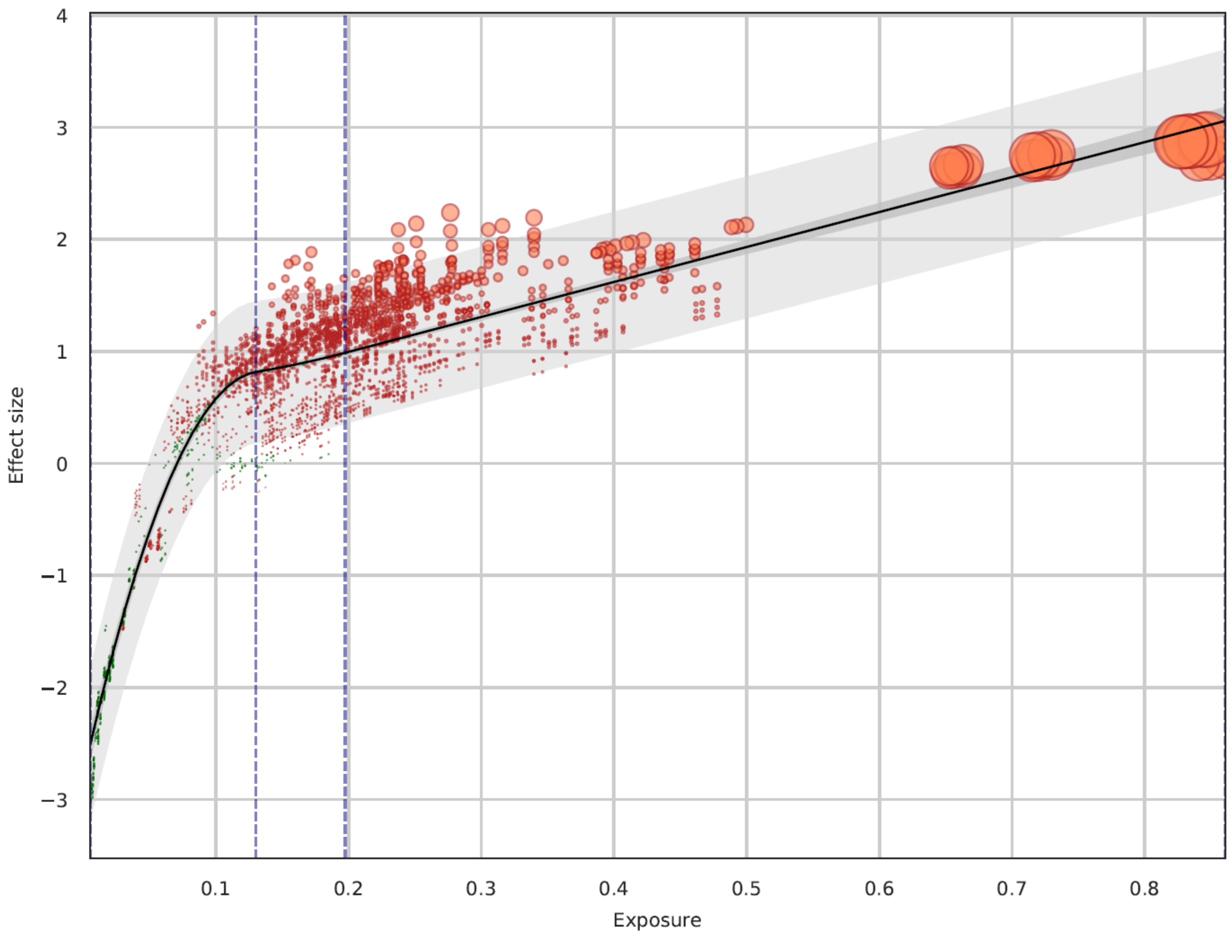

Table 7. MR-BRT preterm birth-low birthweight crosswalk adjustment factor for neonatal preterm birth (<37 weeks of gestation)

Data input	Reference or alternative case definition	Gamma	Beta coefficient, log (95% CI)	Adjustment factor*
Preterm birth	Reference	0.41	---	---
Low birthweight	Alt		-0.0974 (-0.0807, -0.1161)	0.907 (0.890, 0.922)

**Adjustment factor is the transformed beta coefficient in normal space and can be interpreted as the factor by which the alternative case definition is adjusted to reflect what it would have been if measured as the reference.*

Modelling strategy

Step A: Model univariate birthweight and gestational age distributions at birth, by l/y/s

Microdata are the ideal data source for modelling distributions; however, microdata are not widely available for birthweight and are scarcer for gestational age. Categorical prevalence data are more readily available from a wider range of locations and years for low birthweight (<2500g), extremely preterm (<28 weeks of gestation), and preterm birth (<37 weeks of gestation). Because categorical prevalence has wider availability than microdata, we use prevalence data to assist in modelling birthweight and gestational age ensemble distributions.

Ensemble distribution models can be constructed with three pieces of information: mean of the distribution, variance of the distribution, and the weights of the distributions being ensemble. To model mean and variance for all l/y/s for birthweight and gestational age, we first used spatiotemporal Gaussian process regression (ST-GPR) models to model prevalence of low birthweight, extremely preterm, and preterm birth for all l/y/s at birth. To model mean birthweight for all l/y/s, OLS linear regression was used to regress mean birthweight on log-transformed low birthweight prevalence. This model was then used to predict mean birthweight for all l/y/s, using the prevalence of low birthweight (<2500 grams) modelled for all l/y/s in ST-GPR. Similarly, to model gestational age mean for all l/y/s, OLS linear regression model was used to regress mean gestational age on log-transformed preterm prevalence. Mean gestational age for all l/y/s was predicted using the preterm birth (<37 weeks) estimated modelled in ST-GPR.

Global ensemble weights for gestational age were derived by using a 3 million sample of all available gestational age and birthweight microdata in Table 8 to select the ensemble weights. The two distribution families that received the highest weights were the Weibull (43%) and log-logistic (21%) distributions. Global ensemble weights for birthweight were derived using a 3 million sample of all available microdata in Table 8, in addition to birthweight microdata available primarily through the DHS and MICS surveys. The four distribution families that received the highest weights were the mirror gamma (31%), log-logistic (19%), normal (10%), and mirror gumbel (10%) distributions.

For each l/y/s, given the mean and ensemble weights, the variance was optimised to minimise error on the prevalence of preterm birth (<37 weeks) for the gestational age distribution and prevalence of low birthweight (<2500 grams) for the birthweight distribution.

Step B: Model joint birthweight and gestational age distributions at birth, by l/y/s

In order to model the joint distribution of gestational age and birthweight from separate distributions, information was needed about the correlation between the two distributions. Distributions of gestational age and birthweight are not independent; the Spearman correlation for each country where joint microdata were available (Table 8), pooling across all years of data available, ranged from 0.25 to 0.49. The overall Spearman correlation was 0.38, pooling across all countries in the dataset.

Table 8. Summary of microdata inputs

Location	Years of data	Total births*	Format of data	Spearman correlation	Used in ensemble weight selection	Used in copula parameter selection	Used in relative risk models
BRA	2016	2,854,380	Microdata	0.37	Yes	Yes	No
ECU	2003–2015	2,473,039	Microdata	0.34	Yes	Yes	No

ESP	1990–2014	8,537,220	Microdata	0.42	Yes	Yes	No
JPN	1995–2015	23,644,506	Tabulations	0.41	No	No	Yes
MEX	2008–2012	10,256,117	Microdata	0.35	Yes	Yes	No
NOR	1990–2014	1,489,210	Microdata	0.44	Yes	Yes	Yes
NZL	1990–2016	1,600,501	Microdata	0.25	Yes	Yes	Yes
SGP	1993–2015	972,775	Tabulations	0.41	No	No	Yes
TWN	1998–2002	1,331,760	Tabulations	0.38	No	No	Yes
URY	1996–2014	698,622	Microdata	0.49	Yes	Yes	No
USA	1990–2014	81,929,879	Microdata	0.38	Yes	Yes	Yes

** Pooled across all years and sexes, excluding data missing year of birth, gestational age, or birthweight*

Joint distributions between the birthweight and gestational age marginal distributions were modelled with copulae. The Copula and VineCopula packages in R were used to select the optimal copula family and copula parameters to model the joint distribution, using joint microdata from the country-years in Table 8. The copula family selected from the microdata was "Survival BB8", with theta parameter set to 1.75 and delta parameter set to 1.

The joint distribution of birthweight and gestational age per location-year-sex was modelled using the global copula family and parameters selected and the location-year-sex gestational age and birthweight distributions. The joint distribution was simulated 100 times to capture uncertainty. Each simulation consisted of 10,000 simulated joint birthweight and gestational age datapoints. Each joint distribution was divided into 500g by 2-week bins to match the categorical bins of the relative risk surface. Birth prevalence was then calculated for each 500g by 2-week bin.

Step C: Model joint distributions from birth to the end of the neonatal period, by l/y/s

Early neonatal prevalence and late neonatal prevalence were estimated using life table approaches for each 500g and 2-week bin. Using the all-cause early neonatal mortality rate for each location-year-sex, births per location-year-sex-bin, and the relative risks for each location-year-sex-bin in the early neonatal period, the all-cause early neonatal mortality rate was calculated for each location-year-sex-bin. The early neonatal mortality rate per bin was used to calculate the number of survivors at seven days and prevalence in the early neonatal period. Using the same process, the all-cause late neonatal mortality rate for each location-year-sex was paired with the number of survivors at seven days and late neonatal relative risks per bin to calculate late neonatal prevalence and survivors at 28 days.

Relative risks & theoretical minimum-risk exposure level

Causes

The available data for deriving relative risk was only for all-cause mortality. The exception was the USA linked infant birth-death cohort data, which contained three-digit ICD causes of death, but also had nearly 30% of deaths coded to causes that are ill-defined, or intermediate, in the GBD cause classification system. We analysed the relative risk of all-cause mortality across all available sources and selected outcomes based on criteria of biological plausibility. Some causes, most notably congenital birth defects, haemoglobinopathies, malaria, and HIV/AIDS, were excluded based on the criteria that reverse causality could not be excluded.

Input data

In the Norway, New Zealand, and USA Linked Birth/Death Cohort microdata datasets, livebirths are reported with gestational age, birthweight, and an indicator of death at 7 days and 28 days. For this analysis, gestational age was grouped into two-week categories, and birthweight was grouped into 500-gram categories. The Taiwan, Japan, and Singapore datasets were prepared in tabulations of joint 500-gram and two-week categories. A pooled country analysis of mortality risk in the early neonatal period and late neonatal period by "small for gestational age" category in developing countries in Asia and sub-Saharan Africa were also used to inform the relative risk analysis.

Table 9: Input data for relative risk models

Input data	Relative Risk
Source count (total)	113
Number of countries with data	6

Modelling strategy

For each location, data were pooled across years, and the risk of all-cause mortality at the early neonatal period and late neonatal period at joint birthweight and gestational age combinations was calculated. In all datasets except for the USA, sex-specific data were combined to maximise sample size. The USA analyses were sex-specific. To calculate relative risk at each 500-gram and two-week combination, logistic regression was first used to calculate mortality odds for each joint two-week gestational age and 500-gram birthweight category. Mortality odds were smoothed with Gaussian process regression, with the independent distributions of mortality odds by birthweight and mortality odds by gestational age serving as priors in the regression.

A pooled country analysis of mortality risk in the early neonatal period and late neonatal period by SGA category in developing countries in Asia and sub-Saharan Africa were also converted into 500-gram and two-week bin mortality odds surfaces. The relative risk surfaces produced from microdata and the Asia and Africa surfaces produced from the pooled country analysis were meta-analysed, resulting in a meta-analysed mortality odds surface for each location. The meta-analysed mortality odds surface for each location was smoothed using Gaussian process regression and then converted into mortality risk. To calculate mortality relative risks, the risk of each joint two-week gestational age and 500-gram birthweight category were divided by the risk of mortality in the joint gestational age and birthweight category with the lowest mortality risk.

For each of the country-derived relative risk surfaces, the 500-gram and two-week gestational age joint bin with the lowest risk was identified. This bin differed within each country dataset. To identify the universal 500-gram and two-week gestational age category that would serve as the universal TMREL for our analysis, we chose the bins that was identified to be the TMREL in each country dataset to contribute to the universal TMREL. Therefore, the joint categories that served as our universal TMREL for the LBWSG risk factor were “38-40 weeks of gestation and 3500-4000 grams”, “38-40 weeks of gestation and 4000-4500 grams”, and “40-42 weeks of gestation and 4000-4500 grams”. As the joint TMREL, all three categories were assigned to a relative risk equal to 1.

PAF calculations

The total PAF for the low birthweight and short gestation joint risk factor was calculated by summing the PAF calculated from each 500g x two-week category, with the lowest risk category among all the 500g x two-week categories serving as the TMREL. The equation for calculating PAF for each 500g x two-week category is:

$$PAF_{joasgt} = \frac{\sum_{x=1}^{u} RR_{joast}(x)P_{jasgt}(x) - RR_{joasg}(TMRE_{jas})}{\sum_{x=1}^{u} RR_{joas}(x)P_{jasgt}(x)}$$

To calculate the PAFs for the univariate risks (‘short gestation for birthweight’ and ‘low birth weight for gestation’), relative risks are first weighted by global exposure in 2019, summed across one of the dimensions (gestational age or birth weight), and then rescaled by the maximum RR in the TMREL block (38-42 weeks of gestation and 3500-4500 grams). Any RR less than 1 was set to 1. Exposure was also summed across the same dimension, and the univariate PAF equalled the sum of the product of the weighted RRs and exposures.

References

1. Katz J, Lee AC, Kozuki N, Lawn JE, Cousens S, Blencowe H, et al. Mortality risk in preterm and small-for-gestational-age infants in low-income and middle-income countries: a pooled country analysis. The Lancet. 2013;382(9890):417–25.

Suboptimal breastfeeding

Flowchart

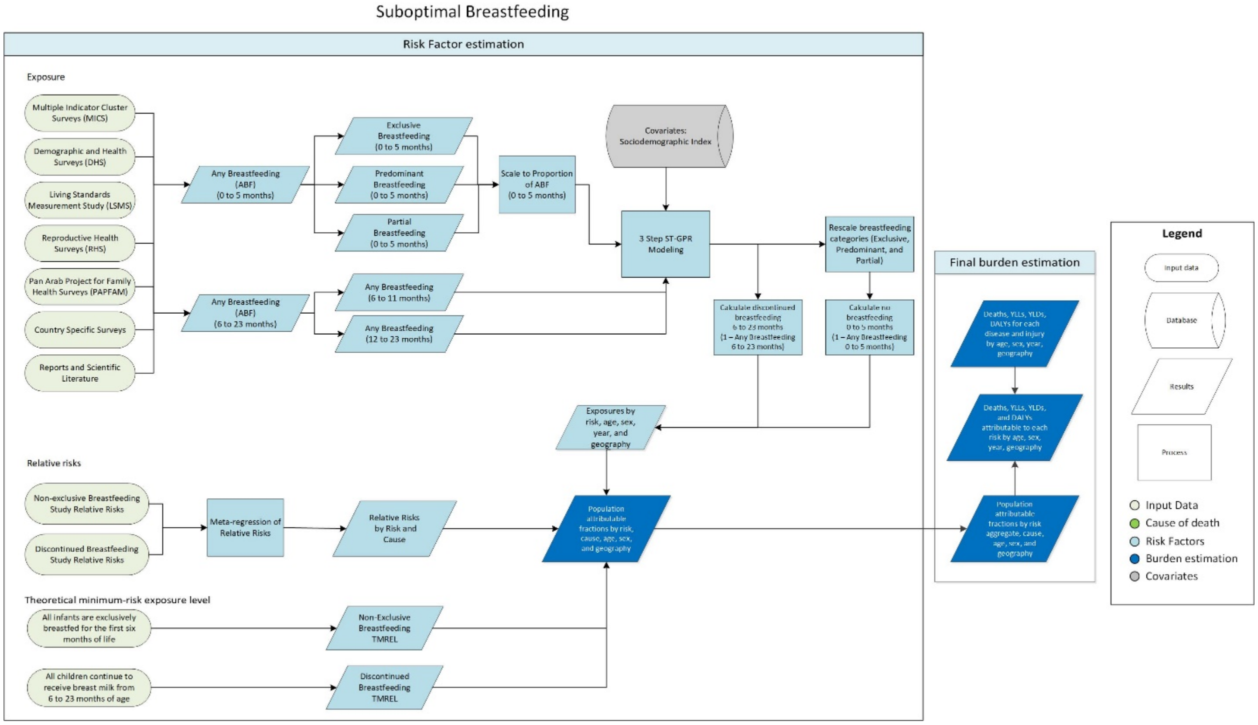

Input data and methodological summary

Exposure definitions

Exposure to suboptimal breastfeeding is composed of two distinct categories: non-exclusive breastfeeding and discontinued breastfeeding.

Non-exclusive breastfeeding is defined as the proportion of children under 6 months of age who are not exclusively breastfed. We then parse those not exclusively breastfed into three categories – predominant, partial, and no breastfeeding. Exclusive breastfeeding is defined as the proportion of children who receive no other food or drink except breastmilk (allowing for ORS, drops, or syrups containing vitamins, minerals, or medicines). Predominant breastfeeding is the proportion of children whose predominant source of nourishment is breastmilk but also receive other liquids. Partial breastfeeding refers to those infants who receive breastmilk as well as food and liquids, including non-human milk and formula. No breastfeeding refers to infants who do not receive breastmilk as a source of nourishment.

Input data

Exposure

The data used in the analysis consist mostly of processed individual-level microdata from surveys; in the cases where microdata were unavailable, we used reported tabulated data from survey reports and scientific literature. Data used to categorise type of non-exclusive breastfeeding (predominant, partial, and none) come from surveys with 24-hour dietary logs based on maternal recall.

We updated our systematic review in GBD 2019 by searching the Global Health Data Exchange (GHDx) using the keyword "breastfeeding." We prioritised extraction of surveys with microdata and new surveys from major survey series such as Demographic and Health Surveys (DHS) and Multiple Indicator Cluster Surveys (MICS).

Exposure modelling

Using the processed microdata and tabulated data from reports, we generated a complete time series from 1980 to 2019 for the prevalence of breastfeeding patterns for children 0 to 5 months and 6 to 23 months using a three-step spatiotemporal Gaussian process regression modelling process.

First, a mixed-effects linear regression with fixed effects on location-level covariates and nested geographical random effects produces a stage 1 prediction. In GBD 2019, we revised this step to include an ensemble stage 1 prediction, estimating candidate models consisting of all combinations of covariates and averaging across the top 50 models weighted by out-of-sample predictive validity. We included the following covariates: Socio-demographic Index, SEV for unsafe water (age-standardised), total fertility rate, maternal education (years per capita), antenatal care (4+ visits), HIV mortality (women of reproductive age), high BMI (women of reproductive age), and underweight (women of reproductive age).

We then followed this with a spatiotemporal regression that uses the residuals of the predictions from the linear regression to perform a locally weighted regression that provides a greater weighting factor to those nearer in space and time. The predicted residuals from this step are then added to those created in the linear regression step.

Finally, we ran a Gaussian process regression that incorporated the variance of the input data as well as the variance of the model predictions. It uses predictions from the spatiotemporal regression as the mean function and generates draws from a multinomial distribution (based on the data uncertainty in the prior) to generate the final prevalence estimates and their confidence intervals.

We estimated six models to produce each of our categories: the proportion of currently breastfeeding infants 0-5 months of age, the ratio of infants exclusively breastfed to breastfed infants 0-5 months of age, the ratio of infants predominantly breastfed to breastfed infants 0-5 months of age, the ratio of infants partially breastfed to breastfed infants 0-5 months of age, the proportion of currently breastfeeding infants 6-11 months of age, and the proportion of currently breastfeeding infants 12-23 months of age. We convert the ratios of exclusive, predominant, and partial breastfeeding to the total category prevalence proportions by multiplying each ratio by the estimates of any breastfeeding among infants aged 0-5 months. This ensures that these categories sum correctly to the "any breastfeeding 0-5 months" envelope. We calculate the proportion of infants receiving no breastmilk 0-5 months of age by subtracting the estimates of current breastfeeding from 1. We perform the same operation to estimate discontinued breastfeeding in the 6-11 months and 12-23 months categories.

Modelling strategy

Assessment of risk-outcome pairs

We included outcomes based on the strength of available evidence supporting a causal relationship. Studies evaluating the causal evidence for our risk-outcome pairs came primarily from articles found in a review published by the World Health Organization.1 Non-exclusive breastfeeding was paired with diarrhoea and lower respiratory infection as diseases outcomes.

Theoretical minimum-risk exposure level

For non-exclusive breastfeeding, those children that received no source of nourishment other than breastmilk ("exclusively breastfed") were considered to be at the lowest risk of any of the disease outcomes. For discontinued breastfeeding, we assumed that children aged 6 to 23 months who received any breastmilk as a source of nourishment to be at the lowest risk of disease outcome.

Relative risks

We estimated relative risks for both non-exclusive and discontinued breastfeeding in a meta-analysis using relative risks from studies compiled in a published review by the World Health Organization.[1]

Population attributable fraction

We used the standard GBD population attributable fraction (PAF) equation to calculate PAFs for non-exclusive breastfeeding and each of their paired outcomes using exposure estimates, the theoretical minimum-risk exposure level, and relative risks.

References

1. Horta, B., Voctora, C. (2013) Short-term effects of breastfeeding: a systematic review on the benefits of breastfeeding on diarrhoea and pneumonia mortality. The World Health Organization.

No access to handwashing facility

Flowchart

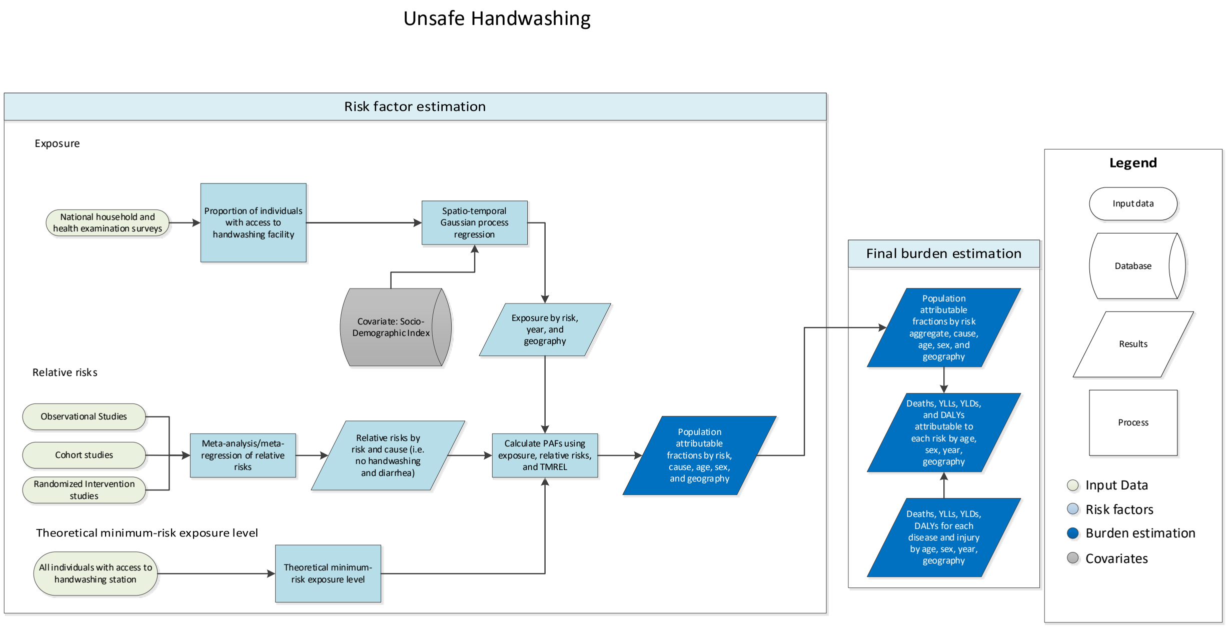

Input data and methodological summary

Exposure

Case definition

This risk is defined as lack of access to a handwashing station with available soap and water.

Input data

Since water and soap availability data are very limited, only country-specific Demographic and Health Surveys (DHS), Multiple Indicator Cluster Surveys (MICS), and Performance Monitoring and Accountability 2020 (PMA2020) surveys conducted from 2000 through 2016 were included as input data. Table 1 provides a summary of the exposure input data.

Table 1: Exposure input data

Input data	Exposure
Source count (total)	98
Number of countries with data	65

Modelling strategy

By year and location, proportion of households with a handwashing facility is modelled using a three-step modelling scheme of mixed effect linear regression followed by spatiotemporal Gaussian process regression (ST-GPR), which outputs full time-series estimates for each GBD 2019 location. Two covariates were used as fixed effects in the linear regression: Socio-demographic Index (SDI – a composite index that includes income per capita, education, and fertility) and proportion of individuals with access to piped water (see below for model equation). Random effects were set at GBD 2019 region and super-region levels to fit the model but were not used in the predictions.

$$logit(data) \sim sdi + piped\ water\ access + (1|level\_1) + (1|level\_2)$$

SDI = socio-demographic index
Piped water access = proportion of individuals with access to piped water
(1|level_1) = super-region-level random effects
(2|level_2) = region-level random effects

The process of vetting and validating models was accomplished primarily through an examination of ST-GPR scatterplots by GBD 2019 location from 1990 to 2019. Based on visual inspection, any poorly fitting datapoints were re-inspected for error at the level of extraction and survey implementation. If errors in data extraction were found, the study in question was re-extracted. In addition to SDI, a number of different potential fixed effects were considered, including lag-distributed income and urbanicity. However, SDI proved to be the strongest predictor.

Data sparseness was a considerable limitation in our modelling process. Even when data were published on handwashing prevalence, the definition as used in the publication often differed from the GBD 2019 standard definition or the data lacked representativeness at the geographical scale we required. The incorporation of questions about soap and water availability in DHS and MICS added much-needed information, but there remains a large data gap to be filled to reduce uncertainty in the estimation of access to handwashing stations.

Theoretical minimum-risk exposure level

The theoretical minimum-risk exposure level for unsafe hygiene is defined as having access to handwashing facility after any contact with excreta, including children's excreta.

Relative risks

For GBD 2019, unsafe hygiene was paired with two outcomes: diarrhoeal diseases and lower respiratory infections (LRI). A meta-analysis by Cairncross and colleagues 2010[1] provided relative risk values describing the relationship between lack of facility access and diarrhoeal diseases. A meta-analysis by Rabie & Curtis 2006[2] provided relative risk evidence for the relationship between lack of facility access and LRI. Table 2 provides a summary of the relative risk data.

Table 2: Relative risk input data

Input data	Relative risk
Source count (total)	29
Number of countries with data	19

In GBD 2019, relative risk values were calculated using MR-BRT. For the LRI model, four study-level covariates were included: whether or not the study was generalisable to the general population, whether or not the measurements used were based on self-reports, whether or not the outcome was blind to the individual level of exposure, and percentage of participants lost to follow-up. No priors were used. Table 3 explains each covariate used in more depth. Table 4 shows the result of the MR-BRT analyses, and Figures 1 shows the associated funnel plot. Table 5 shows the relative risk that was ultimately used for modelling. Note that while the MR-BRT analyses used no access to handwashing facility as the reference, the relative risks used for modelling use access to handwashing facility as the reference.

Table 3: Covariates used in MR-BRT models

Covariate	Description
Measurements based on self-reports	0 = measurements based on assays, tests, or physician observation<br>1 = measurements based on self-report
Loss to follow-up	0 = > 95% follow-up<br>1 = 85-95% follow-up<br>2 = < 85% follow-up
Study generalisation	0 = study sample based on general population<br>1 = study sample based on subgroups, eg, high-risk groups, pregnant women, hospital patients, etc.
Outcome/exposure blinded	0 = outcome was blind to individual level of exposure (or vice versa)<br>1 = unblinded

Table 4: Relative risk MR-BRT results (reference: no access to handwashing facility)

Outcome	Beta coefficient, log (95% CI)	Exponentiated beta (95% CI)
Lower respiratory infections	-0.277 (-0.430, -0.124)	0.758 (0.650, 0.883)

Table 5: Relative risks for each outcome (reference: access to handwashing facility)

Outcome	Relative risk (95% CI)
Lower respiratory infections	1.321 (1.125, 1.533)

Figure 1: MR-BRT funnel plot, lower respiratory infections

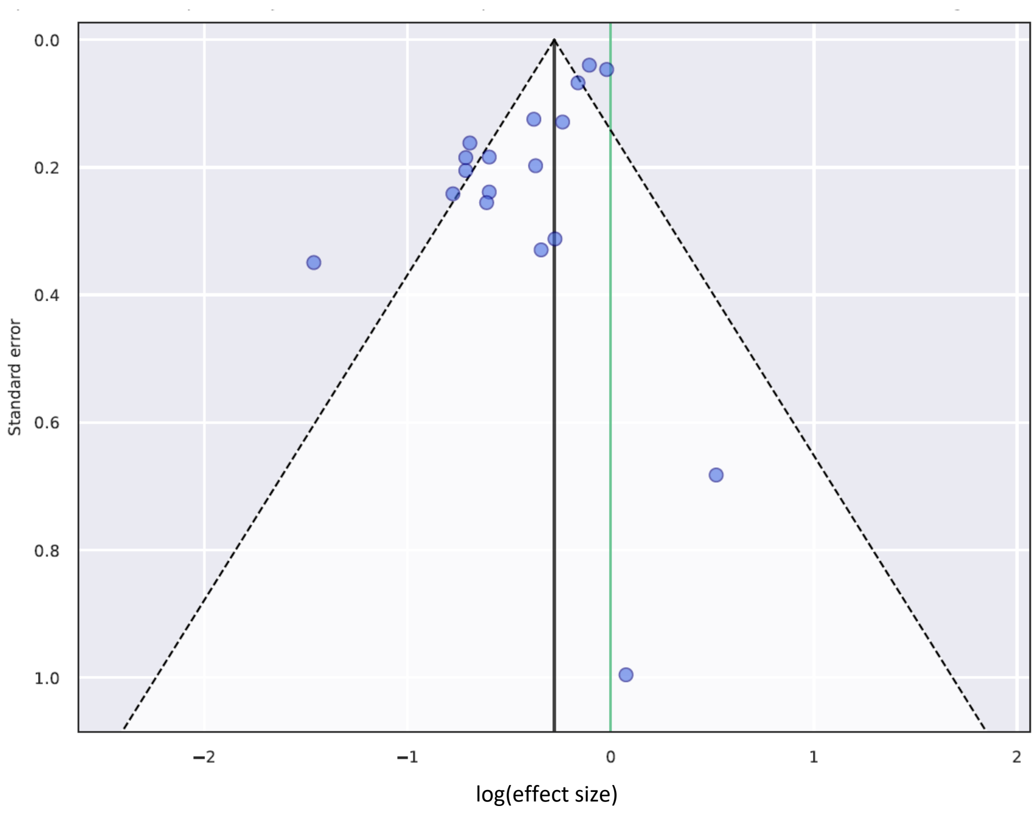

References

1. Cairncross S, Hunt C, Boisson S, *et al.* Water, sanitation and hygiene for the prevention of diarrhoea. *International Journal of Epidemiology* 2010; **39**: i193–205.

2. Rabie T, Curtis V. Handwashing and risk of respiratory infections: a quantitative systematic review. *Tropical Medicine and International Health* 2006; **11**: 258–67.

Alcohol use

Flowchart

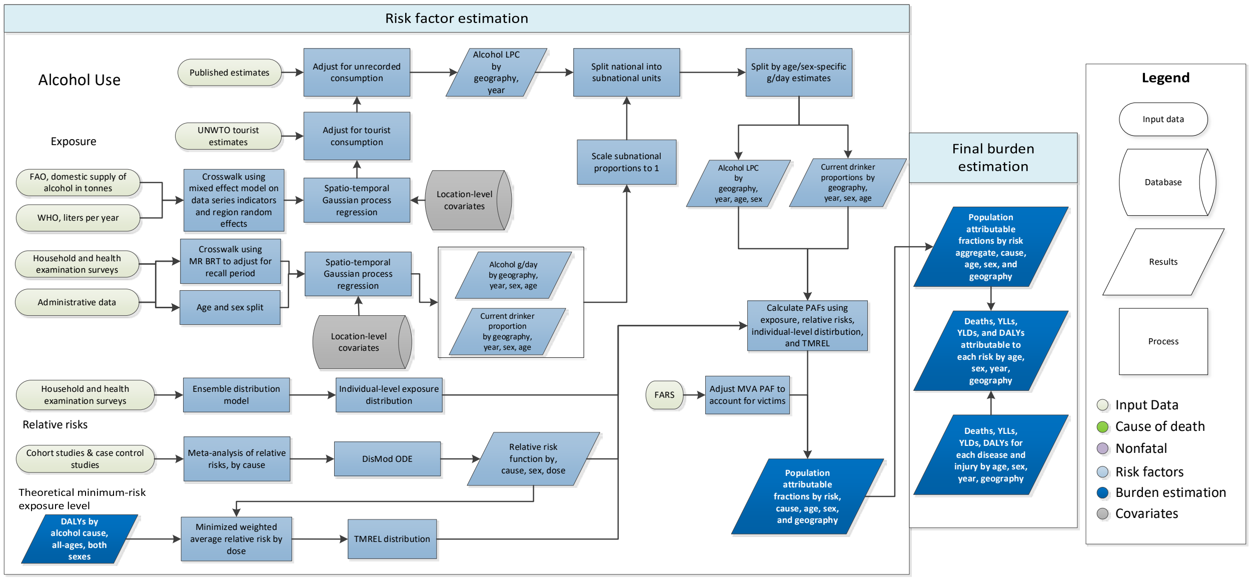

Input data and methodological summary

Definition

Exposure

We defined exposure as the grams per day of pure alcohol consumed among current drinkers. We constructed this exposure using the indicators outlined below:

1. Current drinkers, defined as the proportion of individuals who have consumed at least one alcoholic beverage (or some approximation) in a 12-month period.
2. Alcohol consumption (in grams per day), defined as grams of alcohol consumed by current drinkers, per day, over a 12-month period.
3. Alcohol litres per capita stock, defined in litres per capita of pure alcohol, over a 12-month period.

We also used three additional indicators to adjust alcohol exposure estimates to account for different types of bias:

1. Number of tourists within a location, defined as the total amount of visitors to a location within a 12-month period.
2. Tourists' duration of stay, defined as the number of days resided in a hosting country.
3. Unrecorded alcohol stock, defined as a percentage of the total alcohol stock produced outside established markets.

Input data

A systematic review of the literature was performed to extract data on our primary indicators. The Global Health Exchange (GHDx), IHME's online database of health-related data, was searched for population survey data containing participant-level information from which we could formulate the required alcohol use indicators on current drinkers

and alcohol consumption. Data sources were included if they captured a sample representative of the geographical location under study. We documented relevant survey variables from each data source in a spreadsheet and extracted using STATA 13.1 and R 3.3. A total of 6172 potential data sources were available in the GHDx, of which 5091 have been screened and 1125 accepted.

Table 1: Data inputs for exposure for alcohol use.

Input data	Exposure	Relative risk
Sources (total)	10513	495
Number of countries with data	199	-

Estimates of current drinking prevalence were split by age and sex where necessary. First, studies that reported prevalence for both sexes were split using a region-specific sex ratio estimated using MR-BRT. Second, where studies reported estimates across non-GBD age groups, these were split into standard five-year age groups using the global age pattern estimated by ST-GPR.

Table 2: MR-BRT sex splitting adjustment factors for current drinking

Data input	Gamma	Beta coefficient, log (95% CI)	Adjustment factor*
Female: Male	0	-0.16 (-0.17, -0.14)	0.85
Age < 50	0	0.06 (0.06, 0.06)	1.07
East Asia	0.36	-1.02 (-1.74, -0.29)	0.36
Southeast Asia	0.64	-1.06 (-2.34, 0.22)	0.35
Central Asia	0.41	-0.35 (-1.16, 0.46)	0.70
Central Europe	0.18	-0.21 (-0.58, 0.14)	0.80
Eastern Europe	0.10	-0.07 (-0.28, 0.14)	0.93
High-income Asia Pacific	1.27	-1.11 (-4.90, 2.68)	0.33
Western Europe	0.08	0.03 (-0.14, 0.20)	1.03
Southern Latin America	1.26	-0.67 (-4.18, 2.84)	0.51
High-income North America	0.09	-0.07 (-0.26, 0.11)	0.93
Caribbean	0.25	-0.52 (-1.02, -0.03)	0.59
Andean Latin America	0.76	-0.16 (-1.66, 1.34)	0.85
Central Latin America	0.30	-0.52 (-1.12, 0.08)	0.59
Tropical Latin America	0.08	-0.61 (-0.79, -0.44)	0.54
North Africa and Middle East	1.21	-1.44 (-3.91, 1.03)	0.24
South Asia	0.71	-1.17 (-2.57, 0.23)	0.31
Eastern sub-Saharan Africa	0.28	-0.53 (-1.10, 0.03)	0.58
Southern sub-Saharan Africa	0.20	-0.16 (-0.56, 0.23)	0.85
Western sub-Saharan Africa	0.32	-0.19 (-0.83, 0.45)	0.83
Oceania	0.94	-0.54 (-2.42, 1.34)	0.58

**Adjustment factor is the transformed beta coefficient in normal space and can be interpreted as the factor by which the alternative case definition is adjusted to reflect the ratio by which both-sex data points were split.*

To allow for the inclusion of data that did not meet our reference definition for current drinking, two crosswalks were performed using MR-BRT. The first crosswalk converted estimates of one-month drinking prevalence to what they would be if data represented estimates of 12-month drinking prevalence. This crosswalk incorporated two binary

covariates: male and age ≥ 50. The second crosswalk converted estimates of one-week drinking prevalence to 12-month drinking prevalence. This crosswalk incorporated age < 20 and male as covariates. The covariates utilised in both crosswalks were included as both x and z covariates. A uniform prior of 0 was set as the upper bound for the beta coefficients to enforce the logical constraint that one-month and one-week prevalence could not be greater than 12-month prevalence.

Table 3: MR-BRT crosswalk adjustment factors for alcohol use current drinking model

Data input	Reference or alternative case definition	Gamma	Beta coefficient, logit (95% CI)
12-month prevalence	Ref	---	---
1-month prevalence	Alt	0.22	-0.60 (-1.05, -0.16)
Age ≥ 50		0.13	0.16 (-0.10, 0.43)
Male		0.29	0.01 (-0.57, 0.59)
1-week prevalence	Alt	0.46	-1.51 (-2.42, -0.59)
Age < 20		0.47	-0.29 (-1.34, 0.76)
Male		0.00	0.38 (0.15, 0.60)

The methods for modelling supply-side-level data were changed substantially from those used in GBD 2017. The raw data are domestic supply (WHO GISAH; FAO) and retail supply (Euromonitor) of litres of pure ethanol consumed. Domestic supply is calculated as the sum of production and imports, subtracting exports. The WHO and FAO sources were combined, so that FAO data were only used if there were no data available for that location-year from WHO. This was done because the WHO source takes into consideration FAO values when available. Since the WHO data are given in more granular alcohol types, the following adjustments were made:

$$LPC\ Pure\ Ethanol = 0.13 * \left(\frac{Wine}{0.973}\right)$$

$$LPC\ Pure\ Ethanol = 0.05 * \left(\frac{Beer}{0.989}\right)$$

$$LPC\ Pure\ Ethanol = 0.4 * \left(\frac{Spirits}{0.91}\right)$$

Three outliering strategies are used to omit implausible datapoints and data that created implausible model fluctuations. First, estimates from the current drinking model are used to calculate the grams of alcohol consumed per drinker per day. A point is outliered if the grams of pure ethanol per drinker per day for a given source-location-year is greater than 100 (approximately ten drinks). These thresholds were chosen by using expert knowledge about reasonable consumption levels. In the second round of outliering, the mean liters per capita value over a ten-year window is calculated. If a point is over 70% of that mean value away from the mean value, it is outliered. The 70% limit was chosen using histograms of these distances. Additionally, some manual outliering is performed to account for edge cases. Finally, data smoothing is performed by taking a three-year rolling mean over each location-year.

Next, an imputation to fill in missing years is performed for all series to remove compositional bias from our final estimates. Since the data from our main sources cover different time periods, by imputing a complete time series for

each data series, we reduce the probability that compositional bias of the sources is leading to biased final estimates. To impute the missing years for each series, we model the log ratio of each pair of sources as a function of an intercept and nested random effects on super-region, region, and location. The appropriate predicted ratio is multiplied by the source that we do have, which generates an estimated value for the missing source. For some locations where there was limited overlap between series, the predicted ratio did not make sense, and a regional ratio was used.

Finally, variance was calculated both across series (within a location-year) as well as across years (within a location-source). Additionally, if a location-year had one imputed point, the variance was multiplied by 2. If a location-year had two imputed points, the variance was multiplied by 4. The average estimates in each location-year were the input to an ST-GPR model. This uses a mixed-effects model modelled in log space with nested location random effects.

We obtained data on the number of tourists and their duration of stay from the UNWTO.[3] We applied a crosswalk across different tourist categories, similar to the one used for the litres per capita data, to arrive at a consistent definition (ie, visitors to a country).

We obtained estimates on unrecorded alcohol stock from data available in WHO GISAH database,[2] consisting of 189 locations. For locations with no data available, the national or regional average was used.

For relative risks, in GBD 2016 we performed a systematic literature review of all cohort and case-control studies reporting a relative risk, hazard ratio, or odds ratio for any risk-outcome pairs studied in GBD 2016. Studies were included if they reported a categorical or continuous dose for alcohol consumption, as well as uncertainty measures for their outcomes, and the population under study was representative.

Modelling strategy

While population-based surveys provide accurate estimates of the prevalence of current drinkers, they typically underestimate real alcohol consumption levels.[10-12] As a result, we considered the litre per capita input to be a better estimate of overall volume of consumption. Per capita consumption, however, does not provide age- and sex-specific consumption estimates needed to compute alcohol-attributable burden of disease. Therefore, we use the age-sex pattern of consumption among drinkers modelled from the population survey data and the overall volume of consumption from FAO, GISAH, and Euromonitor to determine the total amount of alcohol consumed within a location. In the paragraphs below, we outline how we estimated each primary input in the alcohol exposure model, as well as how we combined these inputs to arrive at our final estimate of grams per day of pure alcohol. We estimated all models below using 1000 draws.

For data obtained through surveys, we used spatiotemporal Gaussian process regression (ST-GPR) to construct estimates for each location/year/age/sex. We chose to use ST-GPR due to its ability to leverage information across the nearby locations or time periods. We also modelled the alcohol litres per capita (LPC) data, as well as the total number of tourists, using ST-GPR.

Given the heterogeneous nature of the estimates on unrecorded consumption, as well as the wide variation across countries and time periods, we took 1000 draws from the uniform distribution of the lowest and highest estimates available for a given country. We did this to incorporate the diffuse uncertainty within the unrecorded estimates reported. We used these 1000 draws in the equation below.

We adjusted the alcohol LPC for unrecorded consumption using the following equation:

$$Alcohol\ LPC = \frac{Alcohol\ LPC}{(1 - \%\ Unrecorded)}$$

We then adjusted the estimates for alcohol LPC for tourist consumption by adding in the per capita rate of consumption abroad and subtracting the per capita rate of tourist consumption domestically.

$$Alcohol\ LPC_d = Unadjusted\ Alcohol\ LPC_d + Alcohol\ LPC_{Domestic\ consumption\ abroad} - Alcohol\ LPC_{Tourist\ consumption\ domestically}$$

$$Alcohol\ LPC_i = \frac{\sum_l Tourist\ Population_l * Proportion\ of\ tourists_{i,l} * Unadjusted\ Alcohol\ LPC_l * \frac{Average\ length\ of\ stay_{i,l}}{365} *}{Population_d}$$

where:

l *is the set of all locations*, i *is either* Domestic consumption abroad *or* Tourist consumption domestically, *and* d *is a domestic location.*

After adjusting alcohol LPC by tourist consumption and unrecorded consumption for all location/years reported, sex-specific and age-specific estimates were generated by incorporating estimates modelled in ST-GPR for percentage of current drinkers within a location/year/sex/age, as well as consumption trends modelled in the ST-GPR grams per day model. We do this by first calculating the proportion of total consumption for a given location/year by age and sex, using the estimates of alcohol consumed per day, the population size, and the percentage of current drinkers. We then multiply this proportion of total stock for a given location/year/sex/age by the total stock for a given location/year to calculate the consumption in terms of litres per capita for a given location/year/sex/age. We then convert these estimates to be in terms of grams/per day. The following equations describe these calculations:

$$Proportion\ of\ total\ consumption_{l,y,s,a} = \frac{Alcohol\ g/day_{l,y,s,a} * Population_{l,y,s,a} * \%\ Current\ drinkers_{l,y,s,a}}{\sum_{s,a} Alcohol\ g/day_{l,y,s,a} * Population_{l,y,s,a} * \%\ Current\ drinkers_{l,y,s,a}}$$

$$Alcohol\ LPC_{l,y,s,a} = \frac{Alcohol\ LPC_{l,y} * Population_{l,y} * Proportion\ of\ total\ consumption_{l,y,s,a}}{\%\ Current\ drinkers_{l,y,s,a} * Population_{l,y,s,a}}$$

$$Alcohol\ g/day_{l,y,s,a} = Alcohol\ LPC_{l,y,s,a} * \frac{1000}{365}$$

where:

l *is a location*, y *is a year*, s *is a sex*, *and* a *is an age group.*

We then used the gamma distribution to estimate individual-level variation within location, year, sex, age drinking populations, following the recommendations of other published alcohol studies.[7,8] We chose parameters of the gamma distribution based on the mean and standard deviation of the 1,000 draws of alcohol g/day exposure for a

given population. Standard deviation was calculated using the following formula.[15] We tested several alternative models using our data and found this model performed best.

$$standard\ deviation = mean * (0.087 * female + 1.171)$$

Theoretical minimum-risk exposure level

We calculated TMREL by first calculating the overall risk attributable to alcohol. We did this by weighting each relative risk curve by the share of overall DALYs for a given cause. We then took the minimum of this overall-risk curve as the TMREL of alcohol use. More formally,

$$TMREL = argmin\ average\ overall\ risk_{\omega}(g/day)$$

$$Average\ overall\ risk_{\omega}(g/day) = \sum_{i}^{\omega} RR_i(g/day) * \frac{DALY_i}{\sum_i^{\omega} DALY_i}$$

Where:

ω *is the set of causes associated with alcohol*, i *is a given cause from that set*, *DALY is the global DALY rate in* 2010, *and RR is the dose response curve for a given cause and exposure level in grams per day*.

In other words, we chose TMREL as being the exposure that minimises your risk of suffering burden from any given cause related to alcohol. We weight the risk for a particular cause in our aggregation by the proportion of DALYs due to that cause (eg, since more observed people die from ischaemic heart disease [IHD], we weight the risk for IHD more in the above calculation of average risk compared to, say, diabetes, even if both have the same relative risk for a given level of consumption).

Relative risks

We used the studies identified through the systematic review to calculate a dose-response, modelled using DisMod ODE. We chose DisMod ODE rather than a conventional mixed effects meta-regression because of its ability to estimate nonparametric splines over doses (ie, for most alcohol causes, there is a non-linear relationship with different doses) and incorporate heterogeneous doses through dose-integration (ie, most studies report doses categorically in wide ranges. DisMod ODE estimates specific doses when categories overlap across studies, through an integration step.). We used the results of the meta-regression to estimate a non-parametric curve for all doses between zero and 150 g/day and their corresponding relative risks.

Table 4: Data inputs for relative risks for alcohol use.

Input data	Relative risk
Sources (total)	495

Population attributable fraction

For all causes, we defined PAF as:

$$PAF(x) = \frac{P_A + \int_0^{150} P(x) * RR_C(x)\, dx - 1}{P_A + \int_0^{150} P(x) * RR_C(x)\, dx} \qquad P(x) = P_C * \Gamma(\boldsymbol{p})$$

where:

P_C *is the prevalence of current drinkers*, P_a *is the prevalence of abstainers*, $RR_c(x)$ *is the relative risk function for current drinkers, and* $\boldsymbol{p}$ *are parameters determined by the mean and sd of exposure*

We performed the above equation for 1000 draws of the exposure and relative risk models.

References

1. Food and Agriculture Organization of the United Nations (FAO). FAOSTAT Food Balance Sheets, October 2014. Rome, Italy: Food and Agriculture Organization of the United Nations (FAO).
2. World Health Organization (WHO). WHO Global Health Observatory - Recorded adult per capita alcohol consumption, Total per country. Geneva, Switzerland: World Health Organization (WHO).
3. UN World Tourism Organization (UNWTO). UN World Tourism Organization Compendium of Tourism Statistics 2015 [Electronic]. Madrid, Spain: UN World Tourism Organization (UNWTO), 2016.
4. Ramstedt, Mats. “How much alcohol do you buy? A comparison of self-reported alcohol purchases with actual sales.” *Addiction* 105.4 (2010): 649-654.
5. Stockwell, Tim, et al. “Under-reporting of alcohol consumption in household surveys: a comparison of quantity–frequency, graduated–frequency and recent recall.” *Addiction* 99.8 (2004): 1024-1033.
6. Kerr, William C., and Thomas K. Greenfield. “Distribution of alcohol consumption and expenditures and the impact of improved measurement on coverage of alcohol sales in the 2000 National Alcohol Survey.” *Alcoholism: Clinical and Experimental Research* 31.10 (2007): 1714-1722.
7. Taylor, Bruce, et al. “The more you drink, the harder you fall: a systematic review and meta-analysis of how acute alcohol consumption and injury or collision risk increase together.” *Drug and alcohol dependence* 110.1 (2010): 108-116.
8. Vinson, Daniel C., Guilherme Borges, and Cheryl J. Cherpitel. “The risk of intentional injury with acute and chronic alcohol exposures: a case-control and case-crossover study.” *Journal of studies on alcohol* 64.3 (2003): 350-357.
9. Vinson, Daniel C., et al. “A population-based case-crossover and case-control study of alcohol and the risk of injury.” *Journal of studies on alcohol*64.3 (2003): 358-366.
10. Fatal Accident Reporting System (FARS). National Highway Traffic Safety Administration, National Center for Statistics and Analysis Data Reporting and Information Division (NVS-424); 1985, 1990, 1995, 2000, 2005, 2010, 2015
11. Chen, Li-Hui, Susan P. Baker, and Guohua Li. “Drinking history and risk of fatal injury: comparison among specific injury causes.” Accident Analysis & Prevention 37.2 (2005): 245-251.
12. Bell, Nicole S., et al. “Self-reported risk-taking behaviors and hospitalization for motor vehicle injury among active duty army personnel.” American journal of preventive medicine 18.3 (2000): 85-95.
13. Margolis, Karen L., et al. “Risk factors for motor vehicle crashes in older women.” The Journals of Gerontology Series A: Biological Sciences and Medical Sciences 57.3 (2002): M186-M191.
14. Sorock, Gary S., et al. “Alcohol-drinking history and fatal injury in older adults.” Alcohol 40.3 (2006): 193-199.

15. Kehoe, Tara et al. “Determining the best population-level alcohol consumption model and its impact on estimates of alcohol-attributable harms.” Population health metrics 10 6. (2012)

Non-optimal temperature

Flowchart

Non-optimal temperature exposure

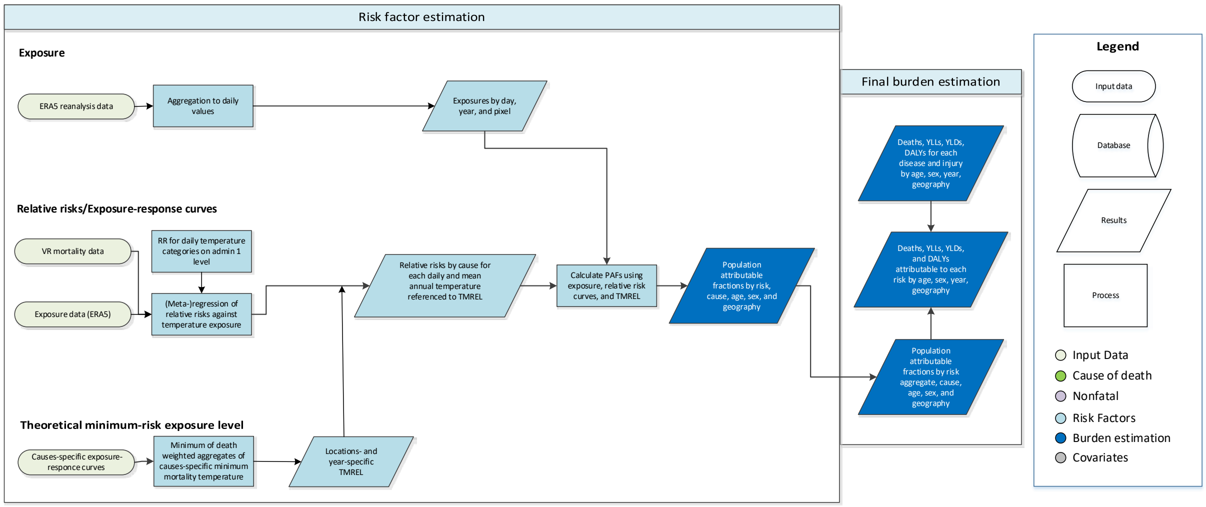

Input data and modelling strategy

Case definition

The exposure of non-optimal temperature is defined as the same day exposure to ambient temperature that is either warmer or colder than the temperature associated with the minimum mortality risk. Specifically, we define the theoretical minimum risk exposure level (TMREL) for temperature as the temperature that is associated with the lowest overall mortality attributable to the risk, in a given location and year. Given varying exposure-response curves for different mean annual temperature zones, as well as spatially and temporally varying cause compositions, we estimate TMRELs by year and location and are not using a globally uniform TMREL. High temperature (heat) exposure is defined as exposure to temperatures warmer than this TMREL and low temperature (cold) is defined as temperatures colder than this TMREL.

Exposure

ERA5 data

We derived exposure estimates from the ERA5 reanalysis dataset from the European Centre for Medium-Range Weather Forecasts (ECMWF). ECMWF produced ERA5 estimates using their Integrated Forecast System (IFS). Hourly values of surface temperature are available for a spatial resolution of 0.25°x0.25°. Uncertainty estimates for these temperature values, ie, the ensemble spread (standard deviation) is available for every 3 hours (00:00, 03:00, 06:00, 09:00, 12:00, 15:00, 18:00, 21:00) for a spatial resolution of 0.5°x0.5°. At the time of analysis, data were available from 1979 to June 2019.[1,2] We calculated daily averages of temperature and spread for each pixel and then assigned an uncertainty value to each daily temperature value. Based on the spread we derived 1,000 draws of each daily temperature pixel.

Population data

Population data for calculating population-weighted location means were derived from WorldPop, which is an open source project initiated in 2013[3]. Multi-temporal, globally consistent, high-resolution human population data at 1 km x 1 km resolution can be downloaded from http://www.worldpop.org.uk/ for 2000, 2005, 2010, 2015, and 2020. For the purpose of our work, we interpolated in-between the 5-year estimation bins to obtain annual data. Further, we extrapolated until 1990 by using the 2000-2005 growth rate for back-casting.

Table 1: Data inputs for exposure for non-optimal temperature.

Input data	Exposure
Source count (total)	203
Number of countries and territories with data	204

Exposure-response modelling

Mortality data

Deaths at the individual-level that included information regarding the cause (ie, ICD code), date, and the location at the second administrative level (admin2) or finer were collected from the GBD cause-of-death (CoD) database for vital registration data sources. We adapted the GBD standard procedure for garbage code redistribution to redistribute daily mortality data rather than annual data and mapped ICD causes to GBD causes for level 3. In total, we analysed 58.9 million deaths from eight different countries and 15,197 administrative units. For Brazil, the data covers a period from 1999 to 2016 for 5,570 municipalities and 19.9 million deaths. For Chile, the data covers the period from 1990 to 1996 and 2009 to 2011 for 15 regions and 2.46 million deaths. For Colombia, the data covers a period from 2001 to 2005 for 1,125 municipalities and 0.95 million deaths. For Guatemala, the data covers a period from 2009 to 2016 for 333 municipalities and 0.49 million deaths. For Mexico, the data covers a period from 1996 to 2015 for 2,438 municipalities and 9.88 million deaths. For New Zealand, the data covers a period from 1988 to 2014 for 20 district health boards and 0.76 million deaths. For the United States, the data covers a period from 1980 to 1988 for 3,124 municipalities and 18.3 million deaths. For China, the data covers the year 2016 for 2,556 counties and 6.1 million deaths.

Exposure-response modelling (MR-BRT)

To estimate cause-specific mortality, based on average daily temperature and temperature zone (defined by mean annual temperature), we used a robust meta-regression framework, implemented through the MR-BRT (Bayesian, regularised, trimmed) tool. The tool allows three features that are essential to the analysis:[5]

- A meta-analytic framework that can handle heterogeneous data sources
- A robust approach to outlier detection and removal (trimming)
- Specification of the functional dependence of outcome vs. average daily temperature and temperature zone as a 2-dimensional surface through a spline interface.

The use of trimming in a vast array of inference and machine learning problems is standard.[6,7,8] The use of high-dimensional splines has been proposed before,[9] but the methods used for estimation go beyond prior work, and we explain them below.

The functional relationship between any outcome y and input variables (t1, t2) models y as a linear combination of 2d spline basis elements. Each spline basis element is a product of individual basis elements for 1D splines for t1 and t2. Therefore, the inference problem looks for a combination of simple curvilinear 2D elements that fit the data while preserving smoothness across element boundaries. The MR-BRT tool also allows prior information to influence the shape of the spline, particularly in areas with sparse data.

For the purpose of modelling the relationship between mortality and mean annual and daily temperature we imposed monotonicity in the direction of daily temperature. For all J-shaped curves that depicted an increase in mortality above and below a threshold, we forced the curve to monotonically decrease at the lower end of the temperature distribution and to monotonically increase at the upper end. For all external causes that displayed a monotonic increase over the entire temperature range, we imposed monotonicity only in the direction of warmer temperatures. We placed 2 knots of degree 3 in the direction of mean annual temperature when fitting the surface. In the direction of daily mean temperature, we placed 3 knots of degree 3 for J-shaped causes and 2 knots of degree 1 for external causes that monotonically increase over temperature range. Figure 1 shows an example of a relative risk (RR) surface along daily and annual mean temperature for drowning.

We estimated uncertainty using a two-step approach. First, we derived the uncertainty of the mean surface from the measurement error using the fit-retrofit error. Second, we added uncertainty from the random effects by sampling it separately from the cold and warm side.

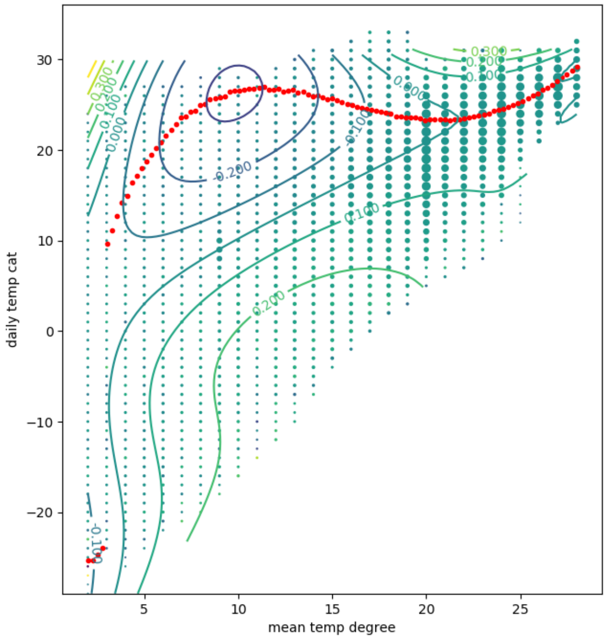

Figure 1: Log relative risk of death from lower respiratory infection along mean annual temperature (mean temp cat) and daily mean temperature (daily temp cat). The red dotted line depicts minimum mortality temperature along mean annual temperature zones. Green and blue lines depict isopleths, ie, lines of equal log RR of mortality

Table 2: Data inputs for relative risks for non-optimal temperature

Input data	Relative risk
Source count (total)	112
Number of countries with data	8

Cause selection

We excluded all causes with fewer than 100,000 deaths as well as causes of death that did not represent a particular entity but rather a summary category (eg, other cardiovascular diseases). Further, dementia and protein energy malnutrition were not considered in this analysis due to inconsistencies in data classification. The remaining causes were selected based on significance. For this, for each cause and each mean temperature zone we determined the widest range of consecutive daily temperatures with statistically significant relative risks, expressed as a percentage of the full range of daily temperatures in that mean temperature zone. Figure 2 gives an example of the temperature-mortality relationship for three selected slices (mean annual temperature of 6 °C, 17 °C and 21 °C). Significant areas along the exposure-response curves are marked in grey. We included all causes where at least 30% of zones had a consecutive significance range that spanned at least 5% of the full range of daily temperatures. Twelve causes met these criteria and were included as outcomes associated with non-optimal temperature: ischaemic heart disease, stroke, hypertensive heart disease, diabetes, chronic kidney disease, lower respiratory infection, chronic obstructive pulmonary disease, homicide, suicide, mechanical injuries, transport-related injuries, and drowning.

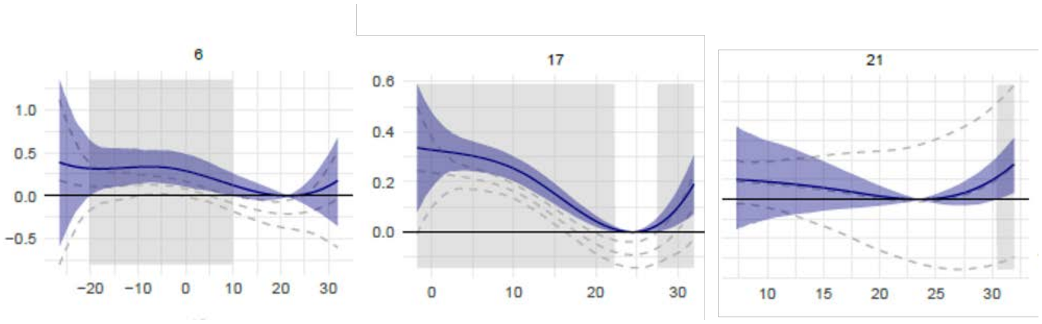

Figure 2: Selected exposure-response curves for the relationship between daily mean temperature and log RR of lower respiratory infection mortality for mean annual temperature categories of 6 °C, 17 °C and 21 °C. Temperatures where associations are significant are displayed in grey.

Theoretical minimum risk exposure level (TMREL)

For the purpose of this analysis, the TMREL was defined as the temperature associated with the lowest mortality for all included causes. We calculated a death-weighted average of the cause-specific exposure-response curves with the minimum of this average curve being the TMREL. This was done for each year and each of the 990 GBD locations using CoD estimates produced for the GBD 2019 study. As climate zones or mean annual temperature can vary within a location, we calculated the TMREL for every mean annual temperature, assuming a consistent cause composition within a location. This approach represents the first use of spatially and temporally varying TMRELs within the GBD study.

Population attributable fractions

The population attributable fraction (PAF) was calculated for each temperature pixel and each day of the year (ie, pixel-day). Subsequently, we population-weighted each pixel using the fraction of the population living in a given

pixel relative to the GBD location. Depending on whether the daily mean temperature was below or above the TMREL, the effect was assigned to either low or high temperature. Daily population-weighted high and low temperature PAFs were then aggregated for the location and the year. Temperature effects can be either harmful or protective depending on whether the RR is above or below 1. For harmful temperature effects, ie, effects with a RR above 1, we used the following equation to derive PAFs: PAF=(RR-1)/RR; For temperature effects exhibiting a protective effect the equation was adapted by implementing the reverse RR: PAF=-((1/RR)-1)/(1/RR). The PAF associated with non-optimal temperature exposure is an aggregate of heat and cold effects in each location and year. We estimated the temperature attributable burden as the product of the total burden for that cause and the corresponding PAF for each GBD location, year, age group, and sex.

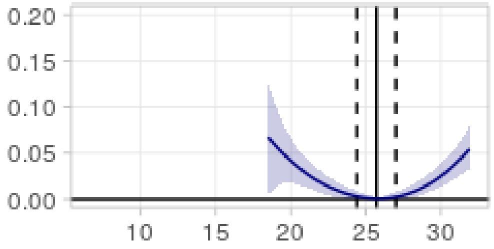

Figure 3: Schematic illustration of the exposure-response relationship between temperature and mortality and associated low temperature (cold) and high temperature effects beyond the theoretical minimum exposure level (TMREL). The blue line depicts the exposure-response curve with blue shaded line showing 95% uncertainty range. The black solid line depicts the TMREL with dashed black lines displaying 95% uncertainty range. Effects left of the TMREL are counted towards cold PAFs and right of the TMREL towards heat PAFS.

References

1 Hersbach H, Bell B, Berrisford P, et al. Global reanalysis : goodbye Global reanalysis : goodbye ERA-Interim , hello. 2019. DOI:10.21957/vf291hehd7.

2 Copernicus Climate Change Service (C3S) (2017): ERA5: Fifth generation of ECMWF atmospheric reanalyses of the global climate. Copernicus Climate Change Service Climate Data Store (CDS), September 2019. .

3 Geography and Environmental Science, University of Southampton. Age and Sex Structures, Global Per Country 2000-2020 - WorldPop. Southampton , United Kingdom: Geography and Environmental Science, University of Southampton, 2018. .

4 Roth GA, Abate D, Hassen Abate K, et al. Global, regional, and national age-sex-specific mortality for 282 causes of death in 195 countries and territories, 1980-2017: a systematic analysis for the Global Burden of Disease Study 2017 GBD 2017 Causes of Death Collaborators*. 2018 DOI:10.1016/S0140-6736(18)32203-7.

5 Zheng P, Aravkin AY, Barber R, Sorensen RJD, Murray CJL. Trimmed Constrained Mixed Effects Models: Formulations and Algorithms. 2019; published online Sept 23. http://arxiv.org/abs/1909.10700 (accessed Nov 13, 2019).

6 Rousseeuw PJ. Least Median of Squares Regression. J Am Stat Assoc 1984; 79: 871–80.

7 Aravkin A, Davis D. Trimmed Statistical Estimation via Variance Reduction. Math Oper Res 2019; : moor.2019.0992.

8 Yang E, Lozano AC, Aravkin A. A general family of trimmed estimators for robust high-dimensional data analysis. Electron J Stat 2018; 12: 3519–53.

9 Pya N, Wood SN. Shape constrained additive models. Stat Comput 2015; 25: 543–59.

Calculating the burden of multiple risk factors

Validation studies have reported congruency between the true risk associated with multiple risk factors affecting the same outcome and a multiplicative aggregation of the PAFs of the individual risk factors (formula below).[1]

$$PAF_{1..i} = 1 - \prod_{i=1}^{n}(1 - PAF_i)$$

where *PAF* is the population attributable fraction and *i* is each individual risk factor.

The same validation studies also found that the overestimation from ignoring the covariance between risk factors is small. This small overestimation was important to note because few data sources exist from which we can draw information on covariance.

Reference

Lim, S.S., Carnahan, E., Nelson, E.C. et al. Validation of a new predictive risk model: measuring the impact of the major modifiable risks of death for patients and populations. Population Health Metrics 13, 27 (2015). https://doi.org/10.1186/s12963-015-0059-8

Inclusion of a risk–outcome pair in the GBD

Inclusion of a risk–outcome pair in the GBD was determined based on the World Cancer Research Fund criteria for convincing or probable evidence.[1,2] Convincing evidence requires the following: evidence from more than one study type; evidence from at least two independent cohort studies; no substantial unexplained heterogeneity within or between study types; good-quality studies to exclude the possibility of confounding, measurement error, and selection bias; and a biologically plausible dose–response relationship. Probable evidence requires: evidence from at least two independent cohort studies or five or more case-control studies; no substantial unexplained heterogeneity within or between study types; good-quality studies to rule out the possibility of confounding, measurement error, and selection bias; and biological plausibility.

The GBD also needed evidence based on past research on the likelihood of importance of a risk factor to disease burden or policy; the availability of adequate data and methodologies for estimating exposure distributions by country; adequate data to estimate outcome-specific effect sizes per unit of exposure; and evidence that effect sizes may be extrapolated to populations other than those studied in epidemiological studies or methods for doing so.[3] Lastly, for a newly evaluated risk–outcome pair, the GBD needed a statistically significant association ($p<0{\cdot}05$) after accounting for sources of potential bias.[4]

Reference

1. World Cancer Research Fund / American Institute for Cancer Research. Judging the evidence. https://www.wcrf.org/wp-content/uploads/2021/02/judging-the-evidence.pdf (accessed May 12, 2022).

2. World Cancer Research Fund/ American Institute for Cancer Research. Food, Nutrition, and Physical Activity, and the Prevention of Cancer. A Global Perspective. Washington D.C, 2007.

3. Lim SS, Vos T, Flaxman AD, et al. A comparative risk assessment of burden of disease and injury attributable to 67 risk factors and risk factor clusters in 21 regions, 1990–2010: a systematic analysis for the Global Burden of Disease Study 2010. The lancet 2013; 380(9859): 2224-60.

4. GBD 2019 Risk Factors Collaborators. Global burden of 87 risk factors in 204 countries and territories, 1990-2019: a systematic analysis for the Global Burden of Disease Study 2019. Lancet 2020; 396(10258): 1223-49.

Lower Respiratory Infection ICD-9 and ICD-10 Codes and Definitions

ICD-9	
**ICD-9 Code**	**Definition**
079.6	Respiratory syncytial virus (RSV)
466	Acute bronchitis and bronchiolitis
466.0	Acute bronchitis
466.1	Acute bronchiolitis
466.11	Acute bronchitis and bronchiolitis due to respiratory syncytial virus (RSV)
466.19	Acute bronchitis and bronchiolitis due to other infectious organisms
466.9	Acute bronchitis and bronchiolitis due to other infectious organisms
467	Acute bronchitis and bronchiolitis due to other infectious organisms
468	Acute bronchitis and bronchiolitis due to other infectious organisms
469	Acute bronchitis and bronchiolitis due to other infectious organisms
470.0	Acute bronchitis and bronchiolitis due to other infectious organisms
480	Viral pneumonia
480.0	Pneumonia due to adenovirus
480.1	Pneumonia due to respiratory syncytial virus
480.2	Pneumonia due to parainfluenza virus
480.3	Pneumonia due to SARS-associated coronavirus
480.8	Pneumonia due to other virus not elsewhere classified
480.9	Viral pneumonia unspecified
481	Pneumococcal pneumonia [Streptococcus pneumoniae pneumonia]
481.0	Pneumococcal pneumonia [Streptococcus pneumoniae pneumonia]
481.2	Pneumococcal pneumonia [Streptococcus pneumoniae pneumonia]
481.9	Pneumococcal pneumonia [Streptococcus pneumoniae pneumonia]
482	Other bacterial pneumonia
482.0	Pneumonia due to Klebsiella pneumoniae
482.1	Pneumonia due to Pseudomonas
482.2	Pneumonia due to Hemophilus influenzae [H. influenzae]
482.3	Pneumonia due to streptococcus
482.30	Pneumonia due to Streptococcus, unspecified
482.31	Pneumonia due to Streptococcus, group A
482.32	Pneumonia due to Streptococcus, group B
482.39	Pneumonia due to other Streptococcus
482.4	Pneumonia due to staphylococcus
482.40	Pneumonia due to Staphylococcus, unspecified
482.41	Methicillin susceptible pneumonia due to Staphylococcus aureus

482.42	Methicillin resistant pneumonia due to Staphylococcus aureus
482.49	Other Staphylococcus pneumonia
482.8	Pneumonia due to other specified bacteria
483.0	Pneumonia due to mycoplasma pneumoniae
483.1	Pneumonia due to chlamydia
483.8	Pneumonia due to other specified organism
483.9	Pneumonia due to other specified organism
484.1	Pneumonia in cytomegalic inclusion disease
484.2	Pneumonia in ornithosis
484.6	Pneumonia in aspergillosis
484.7	Pneumonia in other systemic mycoses
487	Influenza
487.0	Influenza with pneumonia
487.1	Influenza with other respiratory manifestations
487.8	Influenza with other manifestations
487.9	Influenza with other manifestations
488	Influenza due to certain identified influenza viruses
488.0	Influenza due to identified avian influenza virus
488.01	Influenza due to certain identified influenza viruses with pneumonia
488.02	Influenza due to certain identified influenza viruses with other respiratory manifestations
488.09	Influenza due to certain identified influenza viruses with other manifestations
488.1	Influenza due to identified 2009 H1N1 influenza virus
488.11	Influenza due to identified 2009 H1N1 influenza virus with pneumonia
488.12	Influenza due to identified 2009 H1N1 influenza virus with other respiratory manifestations
488.19	Influenza due to identified 2009 H1N1 influenza virus with other manifestations
488.8	Influenza due to novel influenza a
488.81	Influenza due to identified novel influenza A virus with pneumonia
488.82	Influenza due to identified novel influenza A virus with other respiratory manifestations
488.89	Influenza due to identified novel influenza A virus with other manifestations
489	Influenza due to identified novel influenza A virus with other manifestations
ICD-10	
**ICD-10 Code**	**Definition**
A48.1	Legionnaires' disease
A70	Chlamydia psittaci infections(Psittacosis)
B97.4	Respiratory syncytial virus as the cause of diseases classified elsewhere
B97.5	Reovirus as the cause of diseases classified elsewhere
B97.6	Parvovirus as the cause of diseases classified elsewhere
J09	Influenza due to certain identified influenza viruses

J10	Influenza due to other identified influenza virus
J10.0	Influenza due to other identified influenza virus with pneumonia
J10.00	Influenza due to other identified influenza virus with unspecified type of pneumonia
J10.01	Influenza due to other identified influenza virus with the same other identified influenza virus pneumonia
J10.08	Influenza due to other identified influenza virus with other specified pneumonia
J10.1	Influenza due to other identified influenza virus with other respiratory manifestations
J10.2	Influenza due to other identified influenza virus with gastrointestinal manifestations
J10.8	Influenza due to other identified influenza virus with other manifestations
J10.81	Influenza due to other identified influenza virus with encephalopathy
J10.82	Influenza due to other identified influenza virus with myocarditis
J10.83	Influenza due to other identified influenza virus with otitis media
J10.89	Influenza due to other identified influenza virus with other manifestations
J10.9	Influenza due to other identified influenza virus with other manifestations
J11	Influenza due to unidentified influenza virus
J11.0	Influenza due to unidentified influenza virus with pneumonia
J11.00	Influenza due to unidentified influenza virus with unspecified type of pneumonia
J11.08	Influenza due to unidentified influenza virus with specified pneumonia
J11.1	Influenza due to unidentified influenza virus with other respiratory manifestations
J11.2	Influenza due to unidentified influenza virus with gastrointestinal manifestations
J11.8	Influenza due to unidentified influenza virus with other manifestations
J11.81	Influenza due to unidentified influenza virus with encephalopathy
J11.82	Influenza due to unidentified influenza virus with myocarditis
J11.83	Influenza due to unidentified influenza virus with otitis media
J11.89	Influenza due to unidentified influenza virus with other manifestations
J12	Viral pneumonia, not elsewhere classified
J12.0	Adenoviral pneumonia
J12.1	Respiratory syncytial virus pneumonia
J12.2	Parainfluenza virus pneumonia
J12.3	Human metapneumovirus pneumonia
J12.8	Other viral pneumonia
J12.81	Pneumonia due to SARS-associated coronavirus
J12.89	Other viral pneumonia
J12.9	Viral pneumonia, unspecified
J13	Pneumonia due to Streptococcus pneumoniae
J14	Pneumonia due to Hemophilus influenzae
J15	Bacterial pneumonia, not elsewhere classified
J15.0	Pneumonia due to Klebsiella pneumoniae
J15.1	Pneumonia due to Pseudomonas

J15.2	Pneumonia due to staphylococcus
J15.20	Pneumonia due to staphylococcus, unspecified
J15.21	Pneumonia due to staphylococcus aureus
J15.211	Pneumonia due to Methicillin susceptible Staphylococcus aureus
J15.212	Pneumonia due to Methicillin resistant Staphylococcus aureus
J15.29	Pneumonia due to other staphylococcus
J15.3	Pneumonia due to streptococcus, group B
J15.4	Pneumonia due to other streptococci
J15.5	Pneumonia due to Escherichia coli
J15.6	Pneumonia due to other aerobic Gram-negative bacteria
J15.7	Pneumonia due to Mycoplasma pneumoniae
J15.8	Pneumonia due to other specified bacteria
J16	Pneumonia due to other infectious organisms, not elsewhere classified
J16.0	Chlamydial pneumonia
J16.8	Pneumonia due to other specified infectious organisms
J20	Acute bronchitis
J20.0	Acute bronchitis due to Mycoplasma pneumoniae
J20.1	Acute bronchitis due to Hemophilus influenzae
J20.2	Acute bronchitis due to streptococcus
J20.3	Acute bronchitis due to coxsackievirus
J20.4	Acute bronchitis due to parainfluenza virus
J20.5	Acute bronchitis due to respiratory syncytial virus
J20.6	Acute bronchitis due to rhinovirus
J20.7	Acute bronchitis due to echovirus
J20.8	Acute bronchitis due to other specified organisms
J20.9	Acute bronchitis, unspecified
J21	Acute bronchiolitis
J21.0	Acute bronchiolitis due to respiratory syncytial virus
J21.1	Acute bronchiolitis due to human metapneumovirus
J21.8	Acute bronchiolitis due to other specified organisms
J21.9	Acute bronchiolitis, unspecified
J91.0	Malignant pleural effusion
P23.0	Congenital pneumonia due to viral agent
P23.1	Congenital pneumonia due to Chlamydia
P23.2	Congenital pneumonia due to staphylococcus
P23.3	Congenital pneumonia due to streptococcus, group B
P23.4	Congenital pneumonia due to Escherichia coli
U04	Severe acute respiratory syndrome [SARS]

GATHER Compliance

GATHER checklist of information that should be included in reports of global health estimates, with description of compliance and location of information for “Age-sex differences in the global burden of lower respiratory infections and risk factors: results from the Global Burden of Disease Study 2019”.

#	GATHER checklist item	Description of compliance	Reference
**Objectives and funding**			
1	Define the indicators, populations, and time periods for which estimates were made.	Narrative provided in paper and methods appendix describing indicators, definitions, and populations	Main text (Introduction, pg. 4) and methods appendix (pg. 6-10)
2	List the funding sources for the work.	Funding sources listed in paper	Main text (Methods; pg. 6)
**Data Inputs**			
For all data inputs from multiple sources that are synthesized as part of the study:			
3	Describe how the data were identified and how the data were accessed.	Narrative description of data seeking<br><br>methods provided	Main text (Methods; pg. 5-6) and methods appendix (pg. 6-10)
4	Specify the inclusion and exclusion criteria. Identify all ad-hoc exclusions.	Narrative about inclusion and exclusion criteria by data type provided	Methods appendix (pg. 7-8)
5	Provide information on all included data sources and their main characteristics. For each data source used, report reference information or contact name/institution, population represented, data collection method, year(s) of data collection, sex and age range, diagnostic criteria or measurement method, and sample size, as relevant.	An interactive, online data source tool that provides metadata for data sources by component, geography, cause, risk, or impairment has been developed	Information provided in the following online data citation tools:<br>Mortality sources:<br>https://ghdx.healthdata.org/gbd-2019/data-input-sources?components=4&causes=322&locations=1<br><br>Non-fatal sources:<br>https://ghdx.healthdata.org/gbd-2019/data-input-sources?components=

			5&causes=322&locations=1
6	Identify and describe any categories of input data that have potentially important biases (e.g., based on characteristics listed in item 5).	Summary of known biases included in methods appendix	Main text (Discussion pg. 15) Methods appendix (pg. 8)
For data inputs that contribute to the analysis but were not synthesized as part of the study:			
7	Describe and give sources for any other data inputs.	Included in online data source tool, http://ghdx.healthdata.org/gbd-2019	Information provided in the following online data citation tools: Mortality sources: https://ghdx.healthdata.org/gbd-2019/data-input-sources?components=4&causes=322&locations=1<br><br>Non-fatal sources: https://ghdx.healthdata.org/gbd-2019/data-input-sources?components=5&causes=322&locations=1
For all data inputs:			

8	Provide all data inputs in a file format from which data can be efficiently extracted (e.g., a spreadsheet as opposed to a PDF), including all relevant meta-data listed in item 5. For any data inputs that cannot be shared due to ethical or legal, reasons, such as third-party ownership, provide a contact name or the name of the institution that retains the right to the data.	Downloads of input data available through online tools, including data visualization tools and data query tools,<br><br>http://ghdx.healthdata. org/gbd-2019; input data not available in tools will be made available upon request	Online data visualization tools (https://vizhub.healthdata.org/gbd-compare/), data query tools (http://ghdx.healthdata.org/gbd-2019/data-input-sources) , and the Global Health Data Exchange (http://ghdx.healthdata.org/gbd-2019)

Data analysis			
9	Provide a conceptual overview of the data analysis method. A diagram may be helpful.	Flow diagrams of the overall methodological processes, as well as cause-specific modelling processes, have been provided	Main text (Methods; pg. 5-6) and methods appendix (pg. 5-14)
10	Provide a detailed description of all steps of the analysis, including mathematical formulae. This description should cover, as relevant, data cleaning, data pre-processing, data adjustments and weighting of data sources, and mathematical or statistical model(s).	Flow diagrams and methodological write-ups	Main text (Methods; pg. 5-6) and methods appendix (pg. 7-11)
11	Describe how candidate models were evaluated and how the final model(s) were selected.	Provided in the methodological write-up	Methods appendix (pg. 11)
12	Provide the results of an evaluation of model performance, if done, as well as the results of any relevant sensitivity analysis.	Provided in the methodological write-up	Methods appendix (pg. 11)
13	Describe methods for calculating uncertainty of the estimates. State which sources of uncertainty were, and were not, accounted for in the uncertainty analysis.	Provided in the methodological write-up	Methods appendix (pg. 8, 10-11)
14	State how analytic or statistical source code used to generate estimates can be accessed.	Access statement provided	Code is provided in an online tool, http://ghdx.healthdata.org/gbd-2019/code
**Results and Discussion**			

15	Provide published estimates in a file format from which data can be efficiently extracted.	Results are available through online data visualization tools, the Global Health Data Exchange, and the online data query tool (https://ghdx.healthdata.org/gbd-2019)	Main text (table 1-2), methods appendix (etable 1-9), and online data tools (data visualization tools, data query tools, and the Global Health Data Exchange, http://ghdx.healthdata.org/gbd-2019)
16	Report a quantitative measure of the uncertainty of the estimates (e.g. uncertainty intervals).	Uncertainty intervals are provided with all results	Main text (Table 1-2), methods appendix (1-9), and online data tools

Author Contributions

Managing the overall research enterprise

Molly H Biehl, Simon I Hay, Hmwe Hmwe Kyu, Christopher J L Murray, Amanda Novotney

Writing the first draft of the manuscript

Hmwe Hmwe Kyu, Amanda Novotney, Sarah Brooke Sirota, Avina Vongpradith

Primary responsibility for applying analytical methods to produce estimates

Samuel B Albertson, Dana Bryazka, Matthew C Doxey, William M Gardner, Christopher E Troeger, Jeff T Zhao

Primary responsibility for seeking, cataloguing, extracting, or cleaning data; designing or coding figures and tables

Samuel B Albertson, Rose G Bender, Fiona B Bennitt, Regina-Mae Villanueva Dominguez, Jorge R Ledesma, Avina Vongpradith

Providing data or critical feedback on data sources

Amir Abdoli, Hassan Abidi, Hassan Abolhassani, Hiwa Abubaker Ali, Ahmed Abu-Zaid, Mohammad Adnan, Qorinah Estiningtyas Sakilah Adnani, Saira Afzal, Muhammad Sohail Afzal, Bright Opoku Ahinkorah, Araz Ramazan Ahmad, Sajjad Ahmad, Sepideh Ahmadi, Ali Ahmadi, Tarik Ahmed Rashid, Hanadi Al Hamad, Samuel B Albertson, Kefyalew Addis Alene, Beriwan Abdulqadir Ali, Syed Shujait Ali, Liaqat Ali, Sami Almustanyir, Nelson Alvis-Guzman, Nelson J Alvis-Zakzuk, John H Amuasi, Dickson A Amugsi, Jalal Arabloo, Demelash Areda, Ayele Mamo Argaw, Judie Arulappan, Mulusew A Asemahagn, Seyyed Shamsadin Athari, Avinash Aujayeb, Samad Azari, Ashish D Badiye, Sara Bagherieh, Atif Amin Baig, Maciej Banach, Indrajit Banerjee, Mainak Bardhan, Amadou Barrow, Abdul-Monim Mohammad Batiha, Melaku Ashagrie Belete, Fiona B Bennitt, Akshaya Srikanth Bhagavathula, Zulfiqar A Bhutta, Soumitra S Bhuyan, Belay Boda Abule Bodicha, Michael Brauer, Nikolay Ivanovich Briko, Dana Bryazka, Muhammad Hammad Butt Butt, Jiao Cai, Luis Alberto Cámera, Vijay Kumar Chattu, Devasahayam J Christopher, Dinh-Toi Chu, Aaron J Cohen, Natália Cruz-Martins, Xiaochen Dai, Rakhi Dandona, Lalit Dandona, Sameer Dhingra, Linh Phuong Doan, Milad

Dodangeh, Fariba Dorostkar, Matthew C Doxey, Haneil Larson Dsouza, Ebrahim Eini, Michael Ekholuenetale, Temitope Cyrus Ekundayo, Maysaa El Sayed Zaki, Luchuo Engelbert Bain, Ryenchindorj Erkhembayar, Adeniyi Francis Fagbamigbe, Shahab Falahi, Anwar Faraj, Ali Fatehizadeh, Peter Andras Gaal, Santosh Gaihre, William M Gardner, Tushar Garg, Melaku Getachew, Asmare Getie, Ahmad Ghashghaee, Sherief Ghozy, Pouya Goleij, Habtamu Alganeh Guadie, Zewdie Gudisa, Veer Bala Gupta, Sapna Gupta, Vivek Kumar Gupta, Arvin Haj-Mirzaian, Rabih Halwani, Mehdi Harorani, Ahmed I Hasaballah, Hossein Hassanian-Moghaddam, Hadi Hassankhani, Simon I Hay, Kamal Hezam, Nobuyuki Horita, Mehdi Hosseinzadeh, Mowafa Househ, Nawfal R Hussein, Segun Emmanuel Ibitoye, Nahlah Elkudssiah Ismail, Jalil Jaafari, Amirreza Javadi Mamaghani, Tahereh Javaheri, Javad Javidnia, Sathish Kumar Jayapal, Shubha Jayaram, Jost B Jonas, Farahnaz Joukar, Jacek Jerzy Jozwiak, Zubair Kabir, Vidya Kadashetti, Himal Kandel, Tesfaye K Tesfaye Kanko, André Karch, Samad Karkhah, Bekalu Getnet Kassa, Harkiran Kaur, Yousef Saleh Khader, Himanshu Khajuria, Ejaz Ahmad Khan, Md Nuruzzaman Khan, Maseer Khan, Yusra H Khan, Imteyaz A Khan, Moien AB Khan, Sezer Kisa, Adnan Kisa, Niranjan Kissoon, Luke D Knibbs, Sonali Kochhar, Vladimir Andreevich Korshunov, Soewarta Kosen, Kewal Krishan, Barthelemy Kuate Defo, G Anil Kumar, Hmwe Hmwe Kyu, Dharmesh Kumar Lal, Judit Lám, Caterina Ledda, Sang-woong Lee, Stephen S Lim, Wei Liu, Gang Liu, László Lorenzovici, Farzan Madadizadeh, Ata Mahmoodpoor, Jamal Majidpoor, Alaa Makki, Elaheh Malakan Rad, Tauqeer Hussain Mallhi, Clara N Matei, Entezar Mehrabi Nasab, Addisu Melese, Gelana Fekadu Worku Mijena, Le Huu Nhat Minh, Abay Sisay Misganaw, Awoke Temesgen Misganaw, Mokhtar Mohammadi, Shafiu Mohammed, Mohammad Mohseni, Nagabhishek Moka, Ali H Mokdad, Sara Momtazmanesh, Lorenzo Monasta, Catrin E Moore, Abdolvahab Moradi, Ebrahim Mostafavi, Majid Motaghinejad, Haleh Mousavi Isfahani, Sumaira Mubarik, Ghulam Mustafa, Mohsen Naghavi, Sanjeev Nair, Tapas Sadasivan Nair, Sreenivas Narasimha Swamy, Zuhair S Natto, Biswa Prakash Nayak, Robina Khan Niazi, Hasti Nouraei, Chimezie Igwegbe Nzoputam, Bogdan Oancea, Chimedsuren Ochir, Andrew T Olagunju, Ahmed Omar Bali, Emad Omer, Mahesh P A, Jagadish Rao Padubidri, Keyvan Pakshir, Tamás Palicz Tamás Palicz Tamás Palicz, Anamika Pandey, Eun-Kee Park, Fatemeh Pashazadeh Kan, Shrikant Pawar, Gavin Pereira, Simone Perna, David M Pigott, Zahra Zahid Piracha, Vivek Podder, Roman V Polibin, Maarten J Postma, Hamid Pourasghari, Naeimeh Pourtaheri, Mirza Muhammad Fahd Fahd Qadir, Mathieu Raad, Saber Raeghi, Fakher Rahim, Vafa Rahimi-Movaghar, Azizur Rahman, Amir Masoud Rahmani, Pradhum Ram, Kiana Ramezanzadeh, Juwel Rana, Sowmya J Rao, Salman Rawaf, David Laith Rawaf, Reza Rawassizadeh, Marissa B Reitsma, Andre M N Renzaho, Luca Ronfani, Basema Saddik, Umar Saeed, Hossein Samadi Kafil, Abdallah M Samy, Milena M Santric-Milicevic, Brijesh Sathian, Ganesh Kumar Saya, Allen Seylani, Masood Ali Shaikh, Mequannent Melaku Sharew, Purva Sharma, Jae Il Shin, K M Shivakumar, Parnian Shobeiri, Wudneh Simegn, Jasvinder A Singh, Valentin Yurievich Skryabin, Anna Aleksandrovna Skryabina, Mohammad Sadegh Soltani-Zangbar, Chandrashekhar T Sreeramareddy, Muhammad Suleman, Alireza Tahamtan, Mircea Tampa, Yibekal Manaye Tefera, Azene Tesfaye, Degefa Gomora Tesfaye, Amir Tiyuri, Munkhsaikhan Togtmol, Marcos Roberto Tovani-Palone, Christopher E Troeger, Irfan Ullah, Era Upadhyay, Sahel Valadan Tahbaz, Pascual R Valdez, Tommi Juhani Vasankari, Bay Vo, Linh Gia Vu, Yasir Waheed, Xiaoyue Xu, Sajad Yaghoubi, Seyed Hossein Yahyazadeh Jabbari, Dong Keon Yon, Naohiro Yonemoto, Burhan Abdullah Zaman, Alireza Zandifar, Iman Zare, Zahra Zareshahrabadi, Mikhail Sergeevich Zastrozhin, Zhi-Jiang Zhang, Jeff T Zhao, Mohammad Zoladl, Alimuddin Zumla

Developing methods or computational machinery

Hiwa Abubaker Ali, Saira Afzal, Araz Ramazan Ahmad, Ali Ahmadi, Tarik Ahmed Rashid, Liaqat Ali, Adel Al-Jumaily, Indrajit Banerjee, Abdul-Monim Mohammad Batiha, Michael Brauer, Katrin Burkart, Aaron J Cohen, Xiaochen Dai, Iman El Sayed, Maysaa El Sayed Zaki, Anwar Faraj, Ali Fatehizadeh, William M Gardner, Asmare Getie, Mehdi Harorani, Simon I Hay, Mehdi Hosseinzadeh, Mowafa Househ, Tahereh Javaheri, Alelign Tasew Jema, Laleh R Kalankesh, Rohollah Kalhor, Samad Karkhah, Adnan Kisa, Hmwe Hmwe Kyu, Sang-woong Lee, Razzagh Mahmoudi, Jamal Majidpoor, Alaa Makki, Le Huu Nhat Minh, Mokhtar Mohammadi, Mohammad Mohseni, Ali H Mokdad, Majid Motaghinejad, Haleh Mousavi Isfahani, Mohsen Naghavi, Ahmed Omar Bali, Emad Omer, Simone Perna, Mirza Muhammad Fahd Fahd Qadir, Saber Raeghi, Alireza Rafiei, Amir Masoud Rahmani, Reza Rawassizadeh, Jefferson

Antonio Buendia Rodriguez, Abdallah M Samy, Ganesh Kumar Saya, Degefa Gomora Tesfaye, Christopher E Troeger, Bay Vo, Sajad Yaghoubi

Providing critical feedback on methods or results

Semagn Mekonnen Abate, Michael Abdelmasseh, Gholamreza Abdoli, Vida Abedi, Aidin Abedi, Tadesse M Abegaz, Hassan Abidi, Richard Gyan Aboagye, Hassan Abolhassani, Yonas Derso Abtew, Hiwa Abubaker Ali, Ahmed Abu-Zaid, Kidist Adamu, Isaac Yeboah Addo, Oyelola A Adegboye, Mohammad Adnan, Qorinah Estiningtyas Sakilah Adnani, Saira Afzal, Muhammad Sohail Afzal, Bright Opoku Ahinkorah, Araz Ramazan Ahmad, Sajjad Ahmad, Aqeel Ahmad, Ali Ahmadi, Haroon Ahmed, Jivan Qasim Ahmed, Tarik Ahmed Rashid, Mostafa Akbarzadeh-Khiavi, Hanadi Al Hamad, Luciana Albano, Bezatu Mengistie Alemu, Kefyalew Addis Alene, Abdelazeem M Algammal, Robert Kaba Alhassan, Syed Shujait Ali, Liaqat Ali, Yousef Alimohamadi, Vahid Alipour, Adel Al-Jumaily, Syed Mohamed Aljunid, Sami Almustanyir, Rajaa M Al-Raddadi, Rami H Hani Al-Rifai, Saif Aldeen S AlRyalat, Nelson Alvis-Guzman, Nelson J Alvis-Zakzuk, Edward Kwabena Ameyaw, Javad Javad Aminian Dehkordi, John H Amuasi, Dickson A Amugsi, Etsay Woldu Anbesu, Anayochukwu Edward Anyasodor, Jalal Arabloo, Demelash Areda, Zeleke Gebru Argaw, Raphael Taiwo Aruleba, Mulusew A Asemahagn, Seyyed Shamsadin Athari, Daniel Atlaw, Sameh Attia, Engi F Attia, Avinash Aujayeb, Tegegn Mulatu Ayana, Martin Amogre Ayanore, Sina Azadnajafabad, Mohammadreza Azangou-Khyavy, Samad Azari, Amirhossein Azari Jafari, Muhammad Badar, Ashish D Badiye, Nayereh Baghcheghi, Sara Bagherieh, Atif Amin Baig, Maciej Banach, Indrajit Banerjee, Mainak Bardhan, Amadou Barrow, Abdul-Monim Mohammad Batiha, Abate Bekele Belachew, Melaku Ashagrie Belete, Akshaya Srikanth Bhagavathula, Pankaj Bhardwaj, Nikha Bhardwaj, Parth Bhatt, Vijayalakshmi S Bhojaraja, Zulfiqar A Bhutta, Soumitra S Bhuyan, Ali Bijani, Saeid Bitaraf, Belay Boda Abule Bodicha, Michael Brauer, Nikolay Ivanovich Briko, Dana Bryazka, Danilo Buonsenso, Katrin Burkart, Muhammad Hammad Butt Butt, Jiao Cai, Luis Alberto Cámera, Promit Ananyo Chakraborty, Muluken Genetu Chanie, Jaykaran Charan, Vijay Kumar Chattu, Sungchul Choi, Sonali Gajanan Choudhari, Enayet Karim Chowdhury, Devasahayam J Christopher, Dinh-Toi Chu, Natalie L Cobb, Natália Cruz-Martins, Omid Dadras, Fentaw Teshome Dagnaw, Xiaochen Dai, Rakhi Dandona, Lalit Dandona, An Thi Minh Dao, Sisay Abebe Debela, Biniyam Demisse, Fitsum Wolde Demisse, Fahd Demissie, Hardik Dineshbhai Desai, Abebaw Alemayehu Desta, Sameer Dhingra, Nancy Diao, Daniel Diaz, Lankamo Ena Digesa, Linh Phuong Doan, Milad Dodangeh, Deepa Dongarwar, Fariba Dorostkar, Wendel Mombaque dos Santos, Matthew C Doxey, Haneil Larson Dsouza, Oyewole Christopher Durojaiye, Hisham Atan Edinur, Elham Ehsani-Chimeh, Ebrahim Eini, Michael Ekholuenetale, Temitope Cyrus Ekundayo, Eman D El Desouky, Iman El Sayed, Maysaa El Sayed Zaki, Muhammed Elhadi, Ahmed Mahmoud Rabie Elkhapery, Amir Emami, Luchuo Engelbert Bain, Ryenchindorj Erkhembayar, Farshid Etaee, Adeniyi Francis Fagbamigbe, Shahab Falahi, Aida Fallahzadeh, Anwar Faraj, Ali Fatehizadeh, Pietro Ferrara, Allegra Allegra Ferrari, Getahun Fetensa, Florian Fischer, Joanne Flavel, Masoud Foroutan, Peter Andras Gaal, Abhay Motiramji Gaidhane, Santosh Gaihre, Nasrin Galehdar, Tushar Garg, Mesfin Gebrehiwot Damtew Gebrehiwot, Mathewos Alemu Gebremichael, Belete Negese Belete Gemeda, Bradford D Gessner, Melaku Getachew, Asmare Getie, Mohammad Ghasemi Nour, Ahmad Ghashghaee, Ali Gholamrezanezhad, Rakesh Ghosh, Sherief Ghozy, Mohamad Golitaleb, Alessandra C Goulart, Habtamu Alganeh Guadie, Veer Bala Gupta, Sapna Gupta, Vivek Kumar Gupta, Alemu Guta, Parham Habibzadeh, Arvin Haj-Mirzaian, Rabih Halwani, Samer Hamidi, Mehdi Harorani, Ahmed I Hasaballah, Hamidreza Hasani, Abbas M Hassan, Hossein Hassanian-Moghaddam, Hadi Hassankhani, Simon I Hay, Khezar Hayat, Behzad Heibati, Mohammad Heidari, Demisu Zenbaba Heyi, Kamal Hezam, Ramesh Holla, Sung Hwi Hong, Nobuyuki Horita, Mohammad-Salar Hosseini, Mehdi Hosseinzadeh, Mihaela Hostiuc, Mowafa Househ, Soodabeh Hoveidamanesh, Nawfal R Hussein, Segun Emmanuel Ibitoye, Olayinka Stephen Ilesanmi, Irena M Ilic, Milena D Ilic, Mustapha Immurana, Nahlah Elkudssiah Ismail, Masao Iwagami, Jalil Jaafari, Elham Jamshidi, Sung-In Jang, Tahereh Javaheri, Fatemeh Javanmardi, Javad Javidnia, Umesh Jayarajah, Shubha Jayaram, Alelign Tasew Jema, Wonjeong Jeong, Jost B Jonas, Nitin Joseph, Farahnaz Joukar, Jacek Jerzy Jozwiak, Vaishali K, Zubair Kabir, Salah Eddine Oussama Kacimi, Vidya Kadashetti, Laleh R Kalankesh, Rohollah Kalhor, Ashwin Kamath, Himal Kandel, Tesfaye K Tesfaye Kanko, Ibraheem M Karaye, André Karch, Samad Karkhah, Bekalu Getnet

Kassa, Patrick DMC Katoto, Harkiran Kaur, Leila Keikavoosi-Arani, Mohammad Keykhaei, Yousef Saleh Khader, Himanshu Khajuria, Ejaz Ahmad Khan, Md Nuruzzaman Khan, Maseer Khan, Yusra H Khan, Imteyaz A Khan, Moien AB Khan, Moawiah Mohammad Khatatbeh, Mina Khosravifar, Jagdish Khubchandani, Min Seo Kim, Ruth W Kimokoti, Sezer Kisa, Adnan Kisa, Niranjan Kissoon, Sonali Kochhar, Farzad Kompani, Hamid Reza Koohestani, Vladimir Andreevich Korshunov, Ai Koyanagi, Kewal Krishan, Barthelemy Kuate Defo, G Anil Kumar, Om P Kurmi, Ambily Kuttikkattu, Hmwe Hmwe Kyu, Dharmesh Kumar Lal, Judit Lám, Caterina Ledda, Sang-woong Lee, Miriam Levi, Sonia Lewycka, Stephen S Lim, Wei Liu, Gang Liu, Rakesh Lodha, László Lorenzovici, Mojgan Lotfi, Joana A Loureiro, Farzan Madadizadeh, Ata Mahmoodpoor, Razzagh Mahmoudi, Marzieh Mahmoudimanesh, Alaa Makki, Elaheh Malakan Rad, Ahmad Azam Malik, Tauqeer Hussain Mallhi, Yosef Manla, Clara N Matei, Alexander G Mathioudakis, Richard James Maude, Entezar Mehrabi Nasab, Addisu Melese, Ziad A Memish, Oliver Mendoza-Cano, Alexios-Fotios A Mentis, Mehari Woldemariam Merid, Tomislav Mestrovic, Ana Carolina Micheletti Gomide Nogueira de Sá, Gelana Fekadu Worku Mijena, Le Huu Nhat Minh, Shabir Ahmad Mir, Reza Mirfakhraie, Seyyedmohammadsadeq Mirmoeeni, Moonis Mirza, Agha Zeeshan Mirza, Mohammad Mirza-Aghazadeh-Attari, Abay Sisay Misganaw, Awoke Temesgen Misganaw, Mokhtar Mohammadi, Esmaeil Mohammadi, Shafiu Mohammed, Arif Mohammed, Syam Mohan, Mohammad Mohseni, Nagabhishek Moka, Ali H Mokdad, Sara Momtazmanesh, Md Moniruzzaman, Fateme Montazeri, Catrin E Moore, Lidia Morawska, Jonathan F Mosser, Ebrahim Mostafavi, Majid Motaghinejad, Haleh Mousavi Isfahani, Seyed Ali Mousavi-Aghdas, Sumaira Mubarik, Efrén Murillo-Zamora, Ghulam Mustafa, Mohsen Naghavi, Sanjeev Nair, Tapas Sadasivan Nair, Houshang Najafi, Atta Abbas Naqvi, Sreenivas Narasimha Swamy, Zuhair S Natto, Biswa Prakash Nayak, Seyed Aria Nejadghaderi, Huy Van Nguyen Nguyen, Robina Khan Niazi, Antonio Tolentino Nogueira de Sá, Ali Nowroozi, Ogochukwu Janet Nzoputam, Chimezie Igwegbe Nzoputam, Bogdan Oancea, Chimedsuren Ochir, Oluwakemi Ololade Odukoya, Hassan Okati-Aliabad, Akinkunmi Paul Okekunle, Osaretin Christabel Okonji, Andrew T Olagunju, Isaac Iyinoluwa Olufadewa, Ahmed Omar Bali, Emad Omer, Erika Ota, Nikita Otstavnov, Abderrahim Oulhaj, Mahesh P A, Jagadish Rao Padubidri, Tamás Palicz Tamás Palicz Tamás Palicz, Anamika Pandey, Suman Pant, Shahina Pardhan, Eun-Cheol Park, Fatemeh Pashazadeh Kan, Rajan Paudel, Shrikant Pawar, Minjin Peng, Gavin Pereira, Simone Perna, Navaraj Perumalsamy, Ionela-Roxana Petcu, David M Pigott, Zahra Zahid Piracha, Vivek Podder, Roman V Polibin, Maarten J Postma, Hamid Pourasghari, Mirza Muhammad Oussama Fahd Qadir, Mathieu Raad, Mohammad Rabiee, Navid Rabiee, Saber Raeghi, Alireza Rafiei, Fakher Rahim, Mehran Rahimi, Vafa Rahimi-Movaghar, Md Obaidur Rahman, Muhammad Aziz Rahman, Azizur Rahman, Mosiur Rahman, Amir Masoud Rahmani, Vahid Rahmanian, Pradhum Ram, Kiana Ramezanzadeh, Juwel Rana, Priyanga Ranasinghe, Usha Rani, Sowmya J Rao, Mohammad-Mahdi Rashidi, Azad Rasul, Salman Rawaf, David Laith Rawaf, Reza Rawassizadeh, Mohammad Sadegh Razeghinia, Marissa B Reitsma, Andre M N Renzaho, Mohsen Rezaeian, Reza Rikhtegar, Jefferson Antonio Buendia Rodriguez, Emma L B Rogowski, Kristina E Rudd, Basema Saddik, Erfan Sadeghi, Umar Saeed, Azam Safary, Sher Zaman Safi, Maryam Sahebazzamani, Sateesh Sakhamuri, Sana Salehi, Muhammad Salman, Hossein Samadi Kafil, Abdallah M Samy, Milena M Santric-Milicevic, Maryam Sarkhosh, Brijesh Sathian, Monika Sawhney, Ganesh Kumar Saya, Abdul-Aziz Seidu, Allen Seylani, Amira A Shaheen, Masood Ali Shaikh, Elaheh Shaker, Hina Shamshad, Mequannent Melaku Sharew, Asaad Sharhani, Azam Sharifi, Purva Sharma, Ali Sheidaei, Suchitra M Shenoy, Jeevan K Shetty, Damtew Solomon Shiferaw, Mika Shigematsu, Jae Il Shin, Hesamaddin Shirzad-Aski, K M Shivakumar, Siddharudha Shivalli, Parnian Shobeiri, Wudneh Simegn, Paramdeep Singh, Jasvinder A Singh, Samarjeet Singh Siwal, Valentin Yurievich Skryabin, Anna Aleksandrovna Skryabina, Mohammad Sadegh Soltani-Zangbar, Suhang Song, Yimeng Song, Prashant Sood, Chandrashekhar T Sreeramareddy, Muhammad Suleman, Seyed-Amir Tabatabaeizadeh, Majid Taheri, Moslem Taheri Soodejani, Elahe Taki, Iman M Talaat, Mircea Tampa, Nathan Y Tat, Vivian Y Tat, Yibekal Manaye Tefera, Gebremaryam Temesgen, Mohamad-Hani Temsah, Azene Tesfaye, Degefa Gomora Tesfaye, Belay Tessema, Rekha Thapar, Jansje Henny Vera Ticoalu, Amir Tiyuri, Munkhsaikhan Togtmol, Marcos Roberto Tovani-Palone, Christopher E Troeger, Derara Girma Tufa, Irfan Ullah, Era Upadhyay, Sahel Valadan Tahbaz, Pascual R Valdez, Rohollah Valizadeh, Constantine Vardavas, Bay Vo, Theo Vos, Linh Gia Vu, Birhanu Wagaye,

Yasir Waheed, Yu Wang, Abdul Waris, Nuwan Darshana Wickramasinghe, Xiaoyue Xu, Sajad Yaghoubi, Gahin Abdulraheem Tayib Yahya Yahya, Seyed Hossein Yahyazadeh Jabbari, Dong Keon Yon, Naohiro Yonemoto, Burhan Abdullah Zaman, Alireza Zandifar, Moein Zangiabadian, Heather J Zar, Iman Zare, Mikhail Sergeevich Zastrozhin, Wu Zeng, Mengxi Zhang, Zhi-Jiang Zhang, Mohammad Zoladl

Drafting the work or revising it critically for important intellectual content

Michael Abdelmasseh, Gholamreza Abdoli, Vida Abedi, Tadesse M Abegaz, Hassan Abidi, Hassan Abolhassani, Yonas Derso Abtew, Eman Abu-Gharbieh, Ahmed Abu-Zaid, Kidist Adamu, Isaac Yeboah Addo, Oyelola A Adegboye, Mohammad Adnan, Qorinah Estiningtyas Sakilah Adnani, Saira Afzal, Muhammad Sohail Afzal, Bright Opoku Ahinkorah, Araz Ramazan Ahmad, Sepideh Ahmadi, Ali Ahmadi, Jivan Qasim Ahmed, Luciana Albano, Mamoon A Aldeyab, Bezatu Mengistie Alemu, Kefyalew Addis Alene, Abdelazeem M Algammal, Fadwa Alhalaiqa Naji Alhalaiqa, Musa Mohammed Ali, Syed Shujait Ali, Liaqat Ali, Sami Almustanyir, Rami H Hani Al-Rifai, Saif Aldeen S AlRyalat, Nelson Alvis-Guzman, Nelson J Alvis-Zakzuk, Javad Javad Aminian Dehkordi, Dickson A Amugsi, Adnan Ansar, Anayochukwu Edward Anyasodor, Jalal Arabloo, Demelash Areda, Judie Arulappan, Raphael Taiwo Aruleba, Mulusew A Asemahagn, Sameh Attia, Engi F Attia, Avinash Aujayeb, Tewachew Awoke, Tegegn Mulatu Ayana, Martin Amogre Ayanore, Mohammadreza Azangou-Khyavy, Amirhossein Azari Jafari, Muhammad Badar, Ashish D Badiye, Sara Bagherieh, Atif Amin Baig, Maciej Banach, Mainak Bardhan, Francesco Barone-Adesi, Hiba Jawdat Barqawi, Amadou Barrow, Azadeh Bashiri, Quique Bassat, Abate Bekele Belachew, Uzma Iqbal Belgaumi, Rose G Bender, Akshaya Srikanth Bhagavathula, Vijayalakshmi S Bhojaraja, Soumitra S Bhuyan, Saeid Bitaraf, Belay Boda Abule Bodicha, Michael Brauer, Nikolay Ivanovich Briko, Danilo Buonsenso, Muhammad Hammad Butt Butt, Jiao Cai, Paulo Camargos, Vijay Kumar Chattu, Patrick R Ching, Yuen Yu Chong, Devasahayam J Christopher, Dinh-Toi Chu, Natalie L Cobb, Natália Cruz-Martins, Biniyam Demisse, Fitsum Wolde Demisse, Solomon Demissie, Diriba Dereje, Hardik Dineshbhai Desai, Belay Desye, Sameer Dhingra, Nancy Diao, Daniel Diaz, Linh Phuong Doan, Milad Dodangeh, Regina-Mae Villanueva Dominguez, Deepa Dongarwar, Wendel Mombaque dos Santos, Haneil Larson Dsouza, Eleonora Dubljanin, Oyewole Christopher Durojaiye, Elham Ehsani-Chimeh, Ebrahim Eini, Temitope Cyrus Ekundayo, Iman El Sayed, Maysaa El Sayed Zaki, Muhammed Elhadi, Ahmed Mahmoud Rabie Elkhapery, Farshid Etaee, Mohamad Ezati Asar, Adeniyi Francis Fagbamigbe, Aida Fallahzadeh, Emerito Jose A Faraon, Ali Fatehizadeh, Pietro Ferrara, Getahun Fetensa, Florian Fischer, Joanne Flavel, Masoud Foroutan, Joseph Jon Frostad, Peter Andras Gaal, Santosh Gaihre, Nasrin Galehdar, Alberto L Garcia-Basteiro, Tushar Garg, Mathewos Alemu Gebremichael, Yibeltal Yismaw Gela, Bradford D Gessner, Melaku Getachew, Asmare Getie, Seyyed-Hadi Ghamari, Mohammad Ghasemi Nour, Ahmad Ghashghaee, Abdolmajid Gholizadeh, Rakesh Ghosh, Sherief Ghozy, Mohamad Golitaleb, Giuseppe Gorini, Alessandra C Goulart, Girma Garedew Goyomsa, Habtamu Alganeh Guadie, Rashid Abdi Guled, Veer Bala Gupta, Sapna Gupta, Vivek Kumar Gupta, Alemu Guta, Parham Habibzadeh, Arvin Haj-Mirzaian, Rabih Halwani, Md Abdul Hannan, Mehdi Harorani, Ahmed I Hasaballah, Hamidreza Hasani, Abbas M Hassan, Shokoufeh Hassani, Simon I Hay, Demisu Zenbaba Heyi, Kamal Hezam, Ramesh Holla, Sung Hwi Hong, Nobuyuki Horita, Mohammad-Salar Hosseini, Junjie Huang, Ivo Iavicoli, Segun Emmanuel Ibitoye, Olayinka Stephen Ilesanmi, Irena M Ilic, Milena D Ilic, Mustapha Immurana, Nahlah Elkudssiah Ismail, Sathish Kumar Jayapal, Umesh Jayarajah, Shubha Jayaram, Alelign Tasew Jema, Jost B Jonas, Nitin Joseph, Jacek Jerzy Jozwiak, Vaishali K, Salah Eddine Oussama Kacimi, Vidya Kadashetti, Laleh R Kalankesh, Ashwin Kamath, Bhushan Dattatray Kamble, Himal Kandel, Tesfaye K Tesfaye Kanko, André Karch, Samad Karkhah, Bekalu Getnet Kassa, Patrick DMC Katoto, Rimple Jeet Kaur, Leila Keikavoosi-Arani, Yousef Saleh Khader, Himanshu Khajuria, Ejaz Ahmad Khan, Md Nuruzzaman Khan, Maseer Khan, Yusra H Khan, Imteyaz A Khan, Moien AB Khan, Gulfaraz Khan, Moawiah Mohammad Khatatbeh, Jagdish Khubchandani, Min Seo Kim, Sezer Kisa, Adnan Kisa, Niranjan Kissoon, Luke D Knibbs, Sonali Kochhar, Farzad Kompani, Vladimir Andreevich Korshunov, Parvaiz A Koul, Ai Koyanagi, Kewal Krishan, Barthelemy Kuate Defo, Om P Kurmi, Ambily Kuttikkattu, Hmwe Hmwe Kyu, Dharmesh Kumar Lal, Judit Lám, Iván Landires, Jorge R Ledesma, Wei Liu, Rakesh Lodha, László Lorenzovici, Mojgan Lotfi, Joana A Loureiro, Ata Mahmoodpoor, Razzagh Mahmoudi, Jamal Majidpoor, Elaheh

Malakan Rad, Ahmad Azam Malik, Tauqeer Hussain Mallhi, Clara N Matei, Alexander G Mathioudakis, Ziad A Memish, Oliver Mendoza-Cano, Alexios-Fotios A Mentis, Tuomo J Meretoja, Mehari Woldemariam Merid, Tomislav Mestrovic, Ana Carolina Micheletti Gomide Nogueira de Sá, Gelana Fekadu Worku Mijena, Le Huu Nhat Minh, Shabir Ahmad Mir, Reza Mirfakhraie, Seyyedmohammadsadeq Mirmoeeni, Moonis Mirza, Agha Zeeshan Mirza, Mohammad Mirza-Aghazadeh-Attari, Abay Sisay Misganaw, Awoke Temesgen Misganaw, Shafiu Mohammed, Arif Mohammed, Syam Mohan, Nagabhishek Moka, Ali H Mokdad, Sara Momtazmanesh, Lorenzo Monasta, Md Moniruzzaman, Fateme Montazeri, Catrin E Moore, Jonathan F Mosser, Ebrahim Mostafavi, Majid Motaghinejad, Seyed Ali Mousavi-Aghdas, Efrén Murillo-Zamora, Ghulam Mustafa, Mohsen Naghavi, Sanjeev Nair, Tapas Sadasivan Nair, Houshang Najafi, Sreenivas Narasimha Swamy, Zuhair S Natto, Biswa Prakash Nayak, Seyed Aria Nejadghaderi, Robina Khan Niazi, Antonio Tolentino Nogueira de Sá, Amanda Novotney, Ali Nowroozi, Virginia Nuñez-Samudio, Ogochukwu Janet Nzoputam, Chimezie Igwegbe Nzoputam, Osaretin Christabel Okonji, Andrew T Olagunju, Eyal Oren, Nikita Otstavnov, Abderrahim Oulhaj, Mahesh P A, Jagadish Rao Padubidri, Reza Pakzad, Tamás Palicz, Suman Pant, Shahina Pardhan, Fatemeh Pashazadeh Kan, Shrikant Pawar, Gavin Pereira, Simone Perna, Navaraj Perumalsamy, Ionela-Roxana Petcu, Zahra Zahid Piracha, Vivek Podder, Roman V Polibin, Maarten J Postma, Mirza Muhammad Fahd Fahd Qadir, Mathieu Raad, Mohammad Rabiee, Navid Rabiee, Saber Raeghi, Fakher Rahim, Mehran Rahimi, Vafa Rahimi-Movaghar, Azizur Rahman, Pradhum Ram, Kiana Ramezanzadeh, Priyanga Ranasinghe, Usha Rani, Sowmya J Rao, Sina Rashedi, Zubair Ahmed Ratan, Salman Rawaf, David Laith Rawaf, Mohammad Sadegh Razeghinia, Elrashdy Moustafa Mohamed Redwan, Andre M N Renzaho, Abanoub Riad, Reza Rikhtegar, Jefferson Antonio Buendia Rodriguez, Luca Ronfani, Kristina E Rudd, Basema Saddik, Umar Saeed, Sher Zaman Safi, Maryam Sahebazzamani, Amirhossein Sahebkar, Sateesh Sakhamuri, Hossein Samadi Kafil, Abdallah M Samy, Milena M Santric-Milicevic, Bruno Piassi Sao Jose, Ganesh Kumar Saya, Allen Seylani, Elaheh Shaker, Hina Shamshad, Mequannent Melaku Sharew, Suchitra M Shenoy, Jeevan K Shetty, Damtew Solomon Shiferaw, Mika Shigematsu, Hesamaddin Shirzad-Aski, K M Shivakumar, Siddharudha Shivalli, Parnian Shobeiri, Wudneh Simegn, Colin R Simpson, Paramdeep Singh, Harpreet Singh, Jasvinder A Singh, Sarah Brooke Sirota, Valentin Yurievich Skryabin, Anna Aleksandrovna Skryabina, Suhang Song, Prashant Sood, Chandrashekhar T Sreeramareddy, Paschalis Steiropoulos, Muhammad Suleman, Seyed-Amir Tabatabaeizadeh, Majid Taheri, Iman M Talaat, Mircea Tampa, Sarmila Tandukar, Nathan Y Tat, Vivian Y Tat, Yibekal Manaye Tefera, Gebremaryam Temesgen, Mohamad-Hani Temsah, Azene Tesfaye, Degefa Gomora Tesfaye, Jansje Henny Vera Ticoalu, Imad I Tleyjeh, Marcos Roberto Tovani-Palone, Irfan Ullah, Era Upadhyay, Sahel Valadan Tahbaz, Constantine Vardavas, Tommi Juhani Vasankari, Avina Vongpradith, Linh Gia Vu, Birhanu Wagaye, Yu Wang, T Eoin West, Nuwan Darshana Wickramasinghe, Sajad Yaghoubi, Gahin Abdulraheem Tayib Yahya, Seyed Hossein Yahyazadeh Jabbari, Naohiro Yonemoto, Alireza Zandifar, Moein Zangiabadian, Heather J Zar, Iman Zare, Armin Zarrintan, Mikhail Sergeevich Zastrozhin, Chenwen Zhong, Mohammad Zoladl, Alimuddin Zumla

Managing the estimation or publications process

Molly H Biehl, Michael Brauer, Hailey Hagins, Simon I Hay, Hmwe Hmwe Kyu, Ali H Mokdad, Mohsen Naghavi, Amanda Novotney, and Theo Vos.
