# Supplementary material for: Age–sex differences in the global burden of lower respiratory infections and risk factors, 1990–2019: results from the Global Burden of Disease Study 2019
Source: Lancet Infect Dis. 2022 Nov;22(11):1626–47. doi: 10.1016/S1473-3099(22)00510-2 (PMC9605880; doi:10.1016/S1473-3099(22)00510-2)
Supplement: Supplementary appendix [file mmc2.pdf]

THE LANCET
Infectious Diseases

Supplementary appendix 2

This appendix formed part of the original submission and has been peer reviewed. We post it as supplied by the authors.

Supplement to: GBD 2019 LRI Collaborators. Age–sex differences in the global burden of lower respiratory infections and risk factors, 1990–2019: results from the Global Burden of Disease Study 2019. *Lancet Infect Dis* 2022; published online Aug 11. https://doi.org/10.1016/S1473-3099(22)00510-2.

Appendix 2

“Age-sex differences in the global burden of lower respiratory infections and risk factors: results from the Global Burden of Disease Study 2019”

This appendix provides additional tables and figures for “Age-sex differences in the global burden of lower respiratory infections and risk factors: results from the Global Burden of Disease Study 2019”.

TABLE OF CONTENTS

Supplementary Appendix to "Age-sex differences in the global burden of lower respiratory infections and risk factors: results from the Global Burden of Disease Study 2019" 1

Appendix Figure 1. Global time trend of lower respiratory infection incident episodes (A) and deaths (B) by age and sex, 1990-2019 3

Appendix Table 1. Lower respiratory infection episodes and age standardized incidence rates (per 100, 000 people) among males and females in 2019 and the percent change in episodes and age standardized incidence rates between 1990 and 2019 for 21 GBD regions and 204 countries and territories (95% UI) 5

Appendix Table 2. Lower respiratory infection episodes (in thousands) and incidence rates (per 100,000 people) in 2019 and the percent change in episodes and incidence rates between 1990 and 2019 by age, sex, and GBD super-regions (95% UI) 34

Appendix Table 3. Lower respiratory infection deaths and age standardized mortality rates (per 100, 000 people) among males and females in 2019 and the percent change in deaths and age standardized mortality rates between 1990 and 2019 for 21 GBD regions and 204 countries and territories (95% UI) 38

Appendix Table 4. Lower respiratory infection deaths and age standardized mortality rates attributable to all risk factors combined in 2019 and the percent change in attributable deaths and age standardized mortality rates between 1990 and 2019 for 21 GBD regions and 204 countries and territories (95% UI) 68

Appendix Table 5. Lower respiratory infection mortality attributable to all evaluated risk factors combined in 2019 and the percent change in attributable mortality between 1990 and 2019 by age, sex, and GBD super-regions (95% UI)... 97

Appendix Table 6. Lower respiratory infection age-standardized risk-deleted mortality rate (per 100,000 people) for all evaluated risk factors combined by sex for 204 countries and territories and 21 GBD regions for 1990 and 2019 (95% UI) 102

Appendix Table 7. Population-attributable fractions of lower respiratory infections due to evaluated risk factors among children younger than 5 years in 2019, males (M) and females (F) (95% UI) 117

Appendix Table 8. Population-attributable fractions of lower respiratory infections due to evaluated risk factors among children aged 5-14 years in 2019, males (M) and females (F) (95% UI) 134

Appendix Table 9. Population-attributable fractions of lower respiratory infections due to evaluated risk factors among people aged 15-49 years in 2019, males (M) and females (F) (95% UI) 147

Appendix Table 10. Population-attributable fractions of lower respiratory infections due to evaluated risk factors among people aged 50 to 69 years in 2019, males (M) and females (F) (95% UI) 160

Appendix table 11. Population-attributable fractions of lower respiratory infections due to evaluated risk factors among people aged 70 years and older in 2019, males (M) and females (F) (95% UI) 173

Appendix Figure 1. Global time trend of lower respiratory infection incident episodes (A) and deaths (B) by age and sex, 1990-2019

A

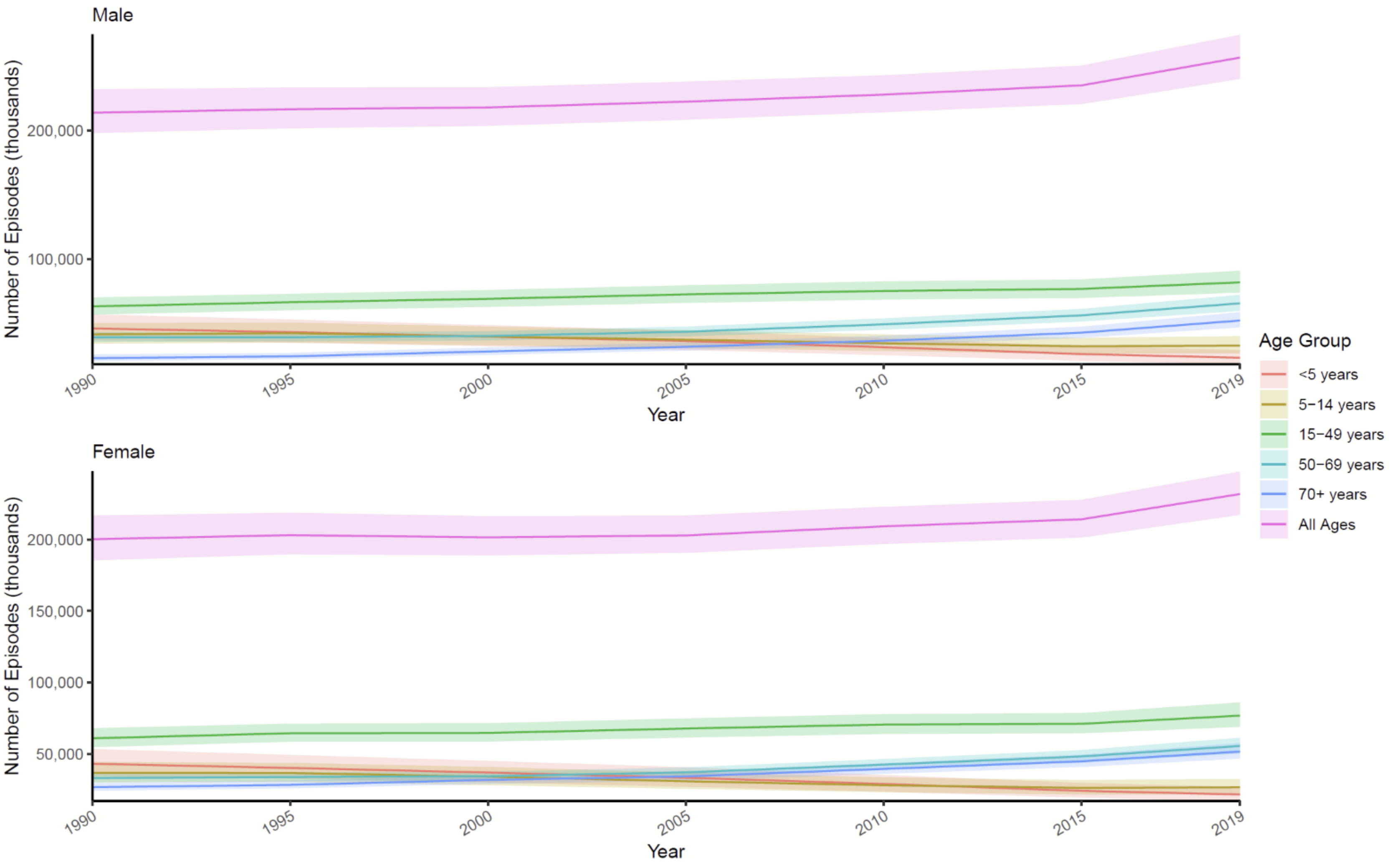

B

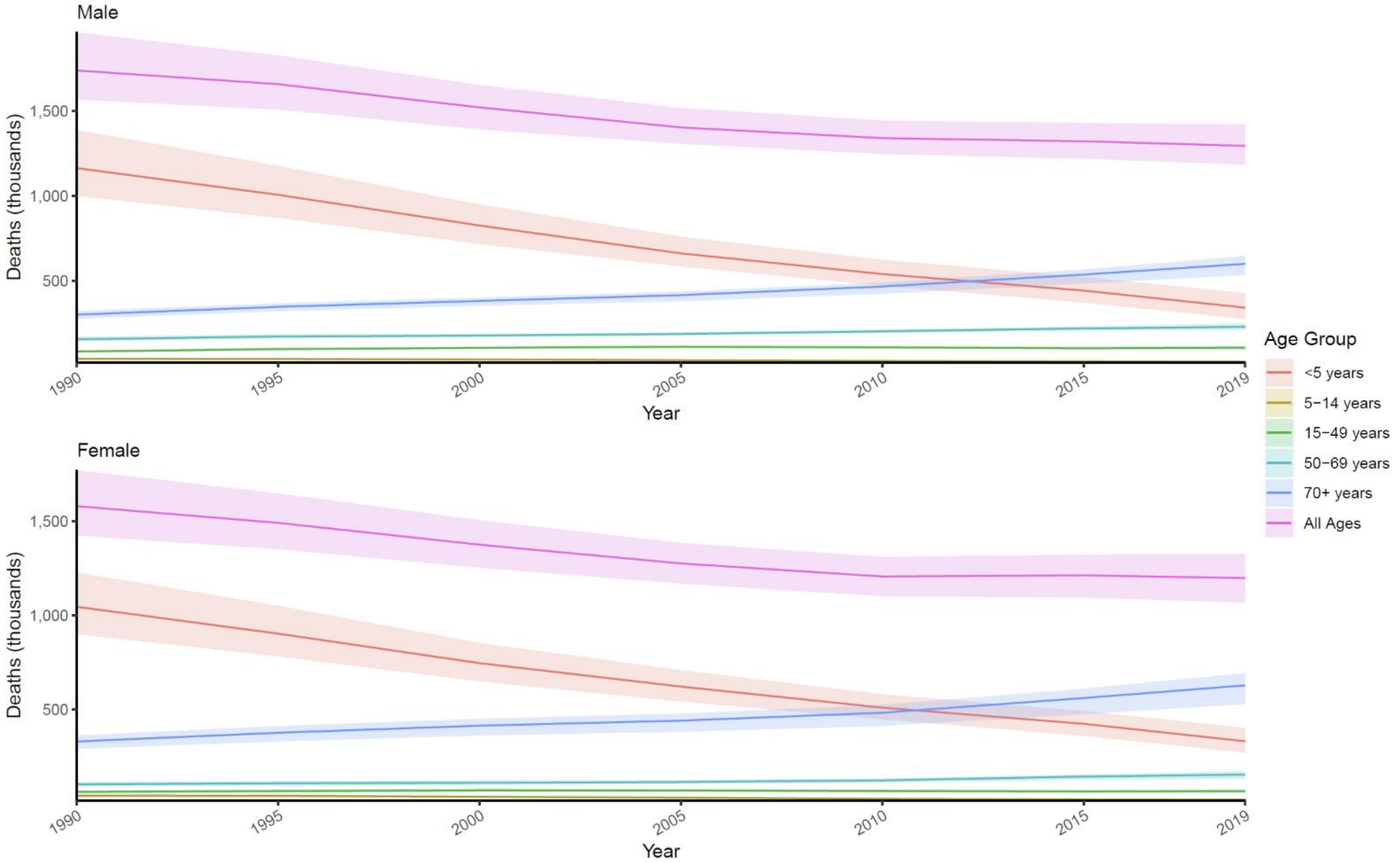
Male
Female
Deaths (thousands)
1,500
1,000
500
1990
1995
2000
2005
2010
2015
2019
Year
Age Group
<5 years
5−14 years
15−49 years
50−69 years
70+ years
All Ages

Appendix Table 1. Lower respiratory infection episodes and age standardized incidence rates (per 100, 000 people) among males and females in 2019 and the percent change in episodes and age standardized incidence rates between 1990 and 2019 for 21 GBD regions and 204 countries and territories (95% UI)

	Male				Female			
	2019		Percent change from 1990 to 2019		2019		Percent change from 1990 to 2019	
	Number of episodes	Age standardized incidence (per 100,000)	Number of episodes	Age standardized incidence	Number of episodes	Age standardized incidence (per 100,000)	Number of episodes	Age standardized incidence
**Global**	**257,000,000 (240,000,000 to 275,000,000)**	**6,832.0 (6,393.8 to 7,313.1)**	**20.0 (15.8 to 24.5)**	**-22.6 (-24.3 to -21.0)**	**232,000,000 (217,000,000 to 248,000,000)**	**5,837.3 (5,450.4 to 6,245.7)**	**15.8 (11.9 to 19.7)**	**-25.5 (-26.9 to -24.0)**
**Central Europe, Eastern Europe, and Central Asia**	**13,000,000 (12,300,000 to 13,900,000)**	**6,040.8 (5,665.9 to 6,450.1)**	**-7.3 (-10.2 to -4.1)**	**-20.2 (-22.2 to -18.1)**	**9,830,000 (9,210,000 to 10,500,000)**	**3,844.3 (3,551.0 to 4,171.0)**	**-15.1 (-17.7 to -12.4)**	**-24.5 (-26.1 to -23.0)**
Central Asia	2,510,000 (2,360,000 to 2,690,000)	6,635.8 (6,262.8 to 7,107.6)	-3.8 (-8.8 to 1.3)	-23.0 (-26.1 to -19.5)	1,940,000 (1,820,000 to 2,070,000)	4,523.5 (4,244.9 to 4,816.8)	-13.6 (-17.8 to -9.3)	-27.6 (-30.4 to -24.6)
Armenia	88,800 (81,300 to 97,600)	5,847.1 (5,333.4 to 6,424.6)	-7.5 (-14.8 to 0.8)	-12.5 (-18.7 to -5.5)	75,700 (69,000 to 83,100)	4,306.2 (3,937.4 to 4,728.5)	-8.2 (-16.3 to 0.8)	-13.9 (-19.9 to -6.9)
Azerbaijan	283,000 (260,000 to 307,000)	6,724.3 (6,216.1 to 7,350.8)	1.0 (-7.6 to 11.2)	-22.8 (-28.7 to -15.8)	208,000 (192,000 to 224,000)	4,732.7 (4,374.9 to 5,112.5)	-8.6 (-16.0 to -0.3)	-22.8 (-28.6 to -16.4)
Georgia	158,000 (144,000 to 173,000)	7,846.0 (7,155.4 to 8,546.7)	-19.6 (-26.9 to -10.3)	-5.2 (-13.9 to 5.0)	135,000 (123,000 to 148,000)	5,287.0 (4,857.7 to 5,766.4)	-23.6 (-30.9 to -15.2)	-14.2 (-21.4 to -6.3)
Kazakhstan	508,000 (467,000 to 551,000)	6,707.4 (6,211.3 to 7,314.8)	0.4 (-7.5 to 8.7)	-12.9 (-19.2 to -5.7)	391,000 (359,000 to 429,000)	4,144.8 (3,821.1 to 4,545.1)	-7.1 (-14.2 to 0.9)	-18.4 (-24.4 to -12.0)
Kyrgyzstan	127,000 (117,000 to 138,000)	4,717.6 (4,358.3 to 5,073.7)	-16.7 (-23.7 to -9.1)	-38.7 (-43.4 to -34.0)	103,000 (93,700 to 113,000)	3,327.0 (3,051.0 to 3,656.1)	-22.8 (-30.2 to -15.5)	-40.0 (-45.4 to -35.0)

	Male				Female			
	2019		Percent change from 1990 to 2019		2019		Percent change from 1990 to 2019	
	Number of episodes	Age standardized incidence (per 100,000)	Number of episodes	Age standardized incidence	Number of episodes	Age standardized incidence (per 100,000)	Number of episodes	Age standardized incidence
Mongolia	71,700 (65,700 to 77,900)	5,481.6 (5,052.0 to 5,960.9)	-19.0 (-26.9 to -11.1)	-43.4 (-47.7 to -38.3)	56,300 (51,700 to 61,600)	3,749.1 (3,454.0 to 4,083.3)	-30.6 (-36.5 to -24.4)	-50.1 (-53.9 to -46.2)
Tajikistan	265,000 (242,000 to 290,000)	7,479.2 (6,906.0 to 8,138.1)	-0.7 (-9.8 to 11.3)	-31.2 (-37.3 to -23.4)	206,000 (187,000 to 227,000)	5,449.2 (5,011.1 to 5,928.5)	-13.0 (-21.0 to -4.3)	-35.9 (-41.4 to -30.5)
Turkmenistan	118,000 (109,000 to 128,000)	5,298.9 (4,898.2 to 5,690.8)	-18.2 (-24.9 to -10.2)	-39.7 (-44.1 to -34.7)	88,400 (81,500 to 95,900)	3,715.6 (3,432.6 to 4,018.9)	-30.3 (-36.7 to -22.2)	-43.0 (-47.7 to -37.5)
Uzbekistan	890,000 (826,000 to 964,000)	6,831.8 (6,372.8 to 7,426.5)	1.3 (-7.9 to 12.3)	-25.8 (-31.4 to -19.2)	681,000 (627,000 to 737,000)	4,752.3 (4,400.1 to 5,129.0)	-10.9 (-18.9 to -2.3)	-31.2 (-36.6 to -25.1)
Central Europe	2,760,000 (2,590,000 to 2,970,000)	4,186.9 (3,859.7 to 4,558.2)	-18.5 (-21.6 to -15.4)	-30.5 (-32.6 to -28.4)	2,370,000 (2,210,000 to 2,550,000)	3,194.1 (2,902.8 to 3,509.2)	-22.9 (-26.2 to -20.0)	-33.2 (-35.5 to -31.1)
Albania	59,500 (54,200 to 65,500)	4,028.0 (3,676.7 to 4,421.0)	-49.5 (-54.7 to -43.4)	-50.6 (-54.4 to -46.4)	49,500 (45,100 to 54,300)	3,441.2 (3,090.1 to 3,805.9)	-49.7 (-54.3 to -44.7)	-46.6 (-50.6 to -42.1)
Bosnia and Herzegovina	65,000 (58,900 to 71,700)	3,797.4 (3,396.3 to 4,253.4)	-33.2 (-38.7 to -26.3)	-27.9 (-33.2 to -21.9)	60,100 (54,400 to 66,600)	3,341.3 (2,969.2 to 3,787.0)	-34.5 (-40.2 to -28.3)	-25.1 (-29.8 to -19.9)
Bulgaria	181,000 (163,000 to 201,000)	4,384.1 (3,988.4 to 4,854.1)	-49.3 (-54.2 to -44.9)	-45.9 (-50.8 to -41.2)	151,000 (137,000 to 165,000)	3,329.3 (3,010.2 to 3,675.5)	-44.4 (-49.6 to -38.7)	-45.5 (-49.7 to -40.7)

	Male				Female			
	2019		Percent change from 1990 to 2019		2019		Percent change from 1990 to 2019	
	Number of episodes	Age standardized incidence (per 100,000)	Number of episodes	Age standardized incidence	Number of episodes	Age standardized incidence (per 100,000)	Number of episodes	Age standardized incidence
Croatia	85,000 (77,000 to 93,400)	3,617.0 (3,248.5 to 4,035.7)	-31.5 (-37.2 to -25.4)	-35.7 (-40.2 to -31.5)	69,400 (63,100 to 75,800)	2,663.2 (2,357.1 to 3,006.4)	-35.8 (-41.6 to -29.6)	-34.4 (-39.1 to -29.7)
Czechia	265,000 (242,000 to 290,000)	4,047.9 (3,674.6 to 4,445.2)	4.8 (-3.7 to 15.1)	-22.8 (-27.9 to -16.5)	228,000 (207,000 to 252,000)	3,115.2 (2,789.6 to 3,463.0)	-9.5 (-17.1 to -1.1)	-26.0 (-30.5 to -21.3)
Hungary	172,000 (156,000 to 190,000)	3,413.7 (3,052.2 to 3,802.3)	-18.2 (-24.6 to -11.5)	-22.3 (-26.9 to -16.4)	190,000 (174,000 to 209,000)	3,210.4 (2,838.0 to 3,599.4)	-11.0 (-19.2 to -3.1)	-16.8 (-22.0 to -11.2)
Montenegro	12,900 (11,700 to 14,200)	4,058.8 (3,640.7 to 4,490.2)	0.3 (-7.6 to 8.9)	-12.3 (-18.2 to -6.5)	11,500 (10,500 to 12,600)	3,520.2 (3,126.0 to 3,974.5)	-4.1 (-10.9 to 3.7)	-11.5 (-16.9 to -5.6)
North Macedonia	41,200 (37,300 to 45,300)	3,978.6 (3,595.3 to 4,418.6)	-14.3 (-21.2 to -6.0)	-24.4 (-30.3 to -18.2)	34,500 (31,200 to 38,000)	3,452.5 (3,079.5 to 3,885.4)	-17.8 (-24.2 to -10.3)	-23.7 (-29.6 to -17.9)
Poland	868,000 (807,000 to 933,000)	3,823.8 (3,530.5 to 4,140.2)	16.3 (10.6 to 22.9)	-14.3 (-16.6 to -11.6)	740,000 (688,000 to 801,000)	2,737.1 (2,494.5 to 3,012.5)	-5.4 (-9.3 to -0.8)	-26.5 (-28.4 to -24.4)
Romania	628,000 (568,000 to 683,000)	5,544.4 (5,079.2 to 6,035.2)	-36.5 (-41.6 to -31.1)	-38.1 (-42.5 to -33.7)	516,000 (474,000 to 568,000)	4,148.1 (3,786.2 to 4,581.2)	-36.2 (-42.4 to -30.7)	-40.3 (-45.3 to -35.6)
Serbia	178,000 (162,000 to 199,000)	3,547.0 (3,203.5 to 3,953.1)	-2.4 (-10.1 to 8.0)	-16.5 (-22.0 to -9.9)	141,000 (127,000 to 156,000)	2,768.7 (2,459.2 to 3,085.6)	-14.4 (-21.1 to -6.9)	-20.8 (-25.9 to -15.7)

	Male				Female			
	2019		Percent change from 1990 to 2019		2019		Percent change from 1990 to 2019	
	Number of episodes	Age standardized incidence (per 100,000)	Number of episodes	Age standardized incidence	Number of episodes	Age standardized incidence (per 100,000)	Number of episodes	Age standardized incidence
Slovakia	158,000 (143,000 to 175,000)	5,378.9 (4,889.0 to 5,922.5)	-21.1 (-28.0 to -13.2)	-35.4 (-40.7 to -29.7)	131,000 (119,000 to 144,000)	3,783.8 (3,425.3 to 4,238.6)	-25.4 (-31.4 to -19.1)	-38.1 (-42.4 to -33.3)
Slovenia	51,100 (46,400 to 56,100)	4,195.5 (3,749.6 to 4,687.1)	-6.2 (-12.7 to 1.5)	-34.1 (-38.2 to -29.5)	47,400 (42,800 to 52,100)	3,319.3 (2,934.2 to 3,730.7)	-10.8 (-18.1 to -2.5)	-31.7 (-36.6 to -27.3)
Eastern Europe	7,780,000 (7,250,000 to 8,320,000)	6,964.0 (6,495.6 to 7,468.8)	-3.6 (-7.1 to 0.1)	-13.0 (-15.7 to -10.0)	5,520,000 (5,130,000 to 5,950,000)	3,988.8 (3,635.1 to 4,347.3)	-11.9 (-14.9 to -8.1)	-17.1 (-19.3 to -14.5)
Belarus	297,000 (273,000 to 322,000)	5,957.4 (5,480.8 to 6,466.5)	-20.4 (-27.2 to -12.4)	-25.2 (-30.8 to -18.6)	202,000 (183,000 to 223,000)	3,335.7 (2,964.8 to 3,728.7)	-29.0 (-34.7 to -22.9)	-30.3 (-34.8 to -25.9)
Estonia	48,600 (43,700 to 54,200)	6,427.6 (5,802.8 to 7,153.4)	-32.3 (-38.9 to -25.9)	-34.2 (-40.0 to -28.9)	38,000 (34,400 to 42,300)	3,961.0 (3,569.0 to 4,413.2)	-27.1 (-34.0 to -19.6)	-29.6 (-34.4 to -24.5)
Latvia	78,800 (71,200 to 87,700)	7,144.2 (6,487.7 to 7,874.9)	-38.4 (-44.9 to -31.2)	-29.7 (-36.0 to -22.9)	69,500 (63,000 to 76,200)	4,725.5 (4,267.1 to 5,240.7)	-31.3 (-37.7 to -24.7)	-24.8 (-29.8 to -19.8)
Lithuania	131,000 (121,000 to 144,000)	8,053.2 (7,414.9 to 8,851.2)	-13.8 (-21.1 to -6.5)	-10.9 (-17.5 to -4.4)	105,000 (94,900 to 116,000)	4,923.4 (4,399.8 to 5,515.3)	-13.4 (-21.5 to -4.7)	-17.7 (-22.4 to -11.7)
Republic of Moldova	158,000 (142,000 to 173,000)	7,657.1 (6,961.9 to 8,383.0)	-20.3 (-28.0 to -11.7)	-23.4 (-30.0 to -16.5)	87,700 (79,300 to 96,100)	3,878.8 (3,515.8 to 4,272.2)	-35.9 (-41.1 to -30.5)	-34.6 (-38.8 to -30.1)

	Male				Female			
	2019		Percent change from 1990 to 2019		2019		Percent change from 1990 to 2019	
	Number of episodes	Age standardized incidence (per 100,000)	Number of episodes	Age standardized incidence	Number of episodes	Age standardized incidence (per 100,000)	Number of episodes	Age standardized incidence
Russian Federation	5,270,000 (4,900,000 to 5,650,000)	6,822.0 (6,355.2 to 7,308.9)	2.1 (-1.4 to 5.5)	-12.6 (-14.9 to -10.1)	3,830,000 (3,570,000 to 4,110,000)	3,967.8 (3,632.5 to 4,319.3)	-5.2 (-8.2 to -2.2)	-16.0 (-18.2 to -13.9)
Ukraine	1,790,000 (1,630,000 to 1,960,000)	7,549.9 (6,905.6 to 8,194.2)	-9.8 (-18.0 to 1.3)	-6.5 (-14.0 to 3.9)	1,190,000 (1,070,000 to 1,300,000)	4,133.8 (3,735.0 to 4,548.9)	-22.4 (-29.5 to -14.2)	-13.6 (-19.8 to -6.7)
**High-income**	**21,100,000 (19,800,000 to 22,600,000)**	**3,252.5 (3,007.1 to 3,529.1)**	**12.4 (9.0 to 15.1)**	**-23.6 (-25.1 to -21.7)**	**20,100,000 (18,900,000 to 21,400,000)**	**2,830.7 (2,600.1 to 3,068.3)**	**9.2 (7.0 to 11.9)**	**-17.7 (-19.2 to -15.9)**
Australasia	449,000 (416,000 to 487,000)	2,576.1 (2,356.3 to 2,818.9)	34.6 (25.9 to 44.8)	-26.8 (-30.9 to -22.4)	399,000 (367,000 to 439,000)	2,056.1 (1,866.8 to 2,268.6)	37.0 (28.1 to 46.6)	-22.2 (-26.3 to -17.8)
Australia	346,000 (318,000 to 379,000)	2,359.5 (2,149.4 to 2,597.9)	33.6 (23.2 to 46.1)	-27.9 (-32.9 to -22.3)	305,000 (278,000 to 340,000)	1,863.6 (1,680.2 to 2,070.6)	39.2 (28.5 to 52.0)	-22.7 (-27.8 to -16.9)
New Zealand	103,000 (95,000 to 112,000)	3,744.2 (3,429.6 to 4,072.3)	37.7 (27.7 to 48.6)	-20.6 (-26.1 to -15.1)	93,900 (86,700 to 102,000)	3,087.8 (2,833.5 to 3,369.9)	30.5 (20.4 to 41.4)	-18.2 (-23.4 to -12.9)
High-income Asia Pacific	3,100,000 (2,890,000 to 3,320,000)	2,670.2 (2,411.7 to 2,943.1)	13.6 (8.2 to 19.0)	-28.8 (-31.2 to -26.3)	2,600,000 (2,430,000 to 2,790,000)	2,081.2 (1,850.5 to 2,320.7)	4.8 (-1.2 to 10.7)	-28.6 (-31.5 to -25.9)
Brunei Darussalam	5,430 (4,930 to 6,010)	3,226.9 (2,946.5 to 3,530.5)	30.3 (20.7 to 41.4)	-11.2 (-17.0 to -5.2)	4,240 (3,800 to 4,700)	2,536.1 (2,266.5 to 2,805.2)	36.7 (27.6 to 47.7)	-7.6 (-13.0 to -1.7)

	Male				Female			
	2019		Percent change from 1990 to 2019		2019		Percent change from 1990 to 2019	
	Number of episodes	Age standardized incidence (per 100,000)	Number of episodes	Age standardized incidence	Number of episodes	Age standardized incidence (per 100,000)	Number of episodes	Age standardized incidence
Japan	2,480,000 (2,310,000 to 2,650,000)	2,768.4 (2,504.9 to 3,049.7)	18.7 (13.2 to 24.3)	-26.0 (-28.3 to -23.0)	2,090,000 (1,940,000 to 2,250,000)	2,140.4 (1,904.9 to 2,380.4)	9.3 (2.5 to 15.6)	-25.2 (-28.1 to -22.4)
Republic of Korea	528,000 (481,000 to 576,000)	2,282.5 (2,029.3 to 2,553.7)	-7.6 (-15.8 to 0.7)	-25.9 (-30.8 to -20.6)	442,000 (400,000 to 483,000)	1,852.0 (1,613.1 to 2,113.4)	-15.2 (-22.7 to -7.1)	-30.1 (-35.2 to -24.1)
Singapore	86,800 (80,400 to 94,400)	3,253.8 (2,961.8 to 3,584.8)	35.9 (24.9 to 50.0)	-36.0 (-40.5 to -31.1)	69,100 (63,800 to 75,700)	2,578.2 (2,321.3 to 2,856.4)	38.3 (28.1 to 49.3)	-35.2 (-39.5 to -30.5)
High-income North America	6,770,000 (6,350,000 to 7,230,000)	3,577.8 (3,298.2 to 3,874.0)	11.2 (7.4 to 15.4)	-18.4 (-21.2 to -15.3)	8,090,000 (7,560,000 to 8,620,000)	3,942.1 (3,641.2 to 4,263.8)	16.0 (13.1 to 19.3)	-7.7 (-10.0 to -4.9)
Canada	564,000 (517,000 to 610,000)	2,875.0 (2,610.1 to 3,183.7)	8.3 (0.5 to 16.4)	-27.9 (-32.4 to -23.4)	682,000 (622,000 to 745,000)	3,155.6 (2,846.9 to 3,472.8)	14.0 (5.7 to 23.4)	-22.1 (-27.1 to -16.9)
Greenland	1,020 (929 to 1,120)	3,438.3 (3,117.3 to 3,749.0)	-15.9 (-22.6 to -8.2)	-30.5 (-35.5 to -25.3)	970 (885 to 1,060)	3,522.2 (3,209.0 to 3,840.5)	-17.9 (-23.8 to -10.7)	-32.6 (-37.3 to -27.7)
United States of America	6,210,000 (5,820,000 to 6,630,000)	3,655.0 (3,366.8 to 3,958.0)	11.5 (7.6 to 15.6)	-17.5 (-20.4 to -14.2)	7,410,000 (6,930,000 to 7,890,000)	4,027.7 (3,723.2 to 4,351.5)	16.2 (12.9 to 19.9)	-6.1 (-8.7 to -3.1)
Southern Latin America	1,760,000 (1,630,000 to 1,900,000)	5,277.1 (4,863.9 to 5,718.4)	26.7 (19.1 to 34.1)	-14.3 (-19.0 to -9.3)	1,670,000 (1,540,000 to 1,830,000)	4,290.0 (3,936.9 to 4,673.1)	36.3 (27.7 to 45.6)	-11.5 (-16.4 to -6.4)

	Male				Female			
	2019		Percent change from 1990 to 2019		2019		Percent change from 1990 to 2019	
	Number of episodes	Age standardized incidence (per 100,000)	Number of episodes	Age standardized incidence	Number of episodes	Age standardized incidence (per 100,000)	Number of episodes	Age standardized incidence
Argentina	1,300,000 (1,190,000 to 1,410,000)	5,842.7 (5,357.8 to 6,362.5)	61.0 (48.1 to 73.8)	11.4 (3.1 to 19.6)	1,240,000 (1,130,000 to 1,370,000)	4,676.1 (4,277.8 to 5,150.4)	71.0 (56.6 to 87.1)	11.8 (3.1 to 21.5)
Chile	383,000 (352,000 to 419,000)	4,234.9 (3,875.5 to 4,666.0)	-22.4 (-28.2 to -16.7)	-52.2 (-56.1 to -48.2)	357,000 (325,000 to 391,000)	3,612.9 (3,260.0 to 3,994.9)	-15.2 (-22.0 to -8.1)	-47.9 (-52.0 to -43.5)
Uruguay	79,800 (73,600 to 87,100)	4,478.5 (4,099.2 to 4,917.8)	-11.4 (-17.0 to -4.6)	-23.5 (-28.2 to -17.8)	80,700 (75,100 to 87,400)	3,640.7 (3,334.1 to 3,994.6)	-3.7 (-10.4 to 3.8)	-23.2 (-27.8 to -18.4)
Western Europe	9,050,000 (8,380,000 to 9,760,000)	2,947.8 (2,732.2 to 3,204.7)	9.6 (5.0 to 13.3)	-29.0 (-30.9 to -27.1)	7,350,000 (6,840,000 to 7,890,000)	2,041.1 (1,863.7 to 2,234.9)	-1.3 (-4.6 to 2.9)	-28.9 (-30.8 to -26.8)
Andorra	1,300 (1,170 to 1,430)	2,563.5 (2,327.2 to 2,822.8)	62.6 (48.9 to 78.2)	-22.5 (-27.7 to -16.7)	1,010 (917 to 1,120)	1,938.6 (1,738.2 to 2,145.0)	76.9 (62.8 to 93.2)	-26.1 (-30.6 to -20.8)
Austria	160,000 (144,000 to 177,000)	2,925.4 (2,627.8 to 3,218.8)	2.5 (-6.0 to 11.0)	-29.0 (-34.2 to -24.1)	127,000 (114,000 to 139,000)	2,081.1 (1,866.0 to 2,317.8)	-12.7 (-20.8 to -4.0)	-27.6 (-32.7 to -22.6)
Belgium	320,000 (293,000 to 352,000)	3,951.2 (3,634.5 to 4,329.5)	32.8 (21.9 to 46.8)	-13.8 (-19.7 to -6.1)	274,000 (249,000 to 307,000)	2,779.6 (2,531.7 to 3,093.3)	30.0 (17.5 to 45.4)	-11.4 (-17.7 to -3.6)
Cyprus	16,300 (15,000 to 17,900)	2,320.0 (2,131.8 to 2,557.2)	34.1 (23.0 to 46.8)	-33.0 (-37.6 to -27.6)	12,600 (11,400 to 13,700)	1,692.5 (1,519.3 to 1,870.7)	25.3 (15.9 to 35.4)	-37.0 (-41.2 to -32.8)

	Male				Female			
	2019		Percent change from 1990 to 2019		2019		Percent change from 1990 to 2019	
	Number of episodes	Age standardized incidence (per 100,000)	Number of episodes	Age standardized incidence	Number of episodes	Age standardized incidence (per 100,000)	Number of episodes	Age standardized incidence
Denmark	123,000 (111,000 to 136,000)	3,119.5 (2,843.8 to 3,460.1)	6.7 (-2.7 to 18.1)	-24.4 (-30.2 to -18.1)	99,700 (90,400 to 109,000)	2,251.3 (2,027.3 to 2,498.7)	-6.8 (-14.8 to 1.7)	-26.9 (-31.7 to -22.2)
Finland	98,000 (89,500 to 108,000)	2,777.7 (2,512.1 to 3,079.2)	-28.8 (-34.6 to -22.3)	-50.5 (-54.2 to -46.5)	76,200 (68,100 to 84,100)	1,981.3 (1,759.6 to 2,231.1)	-41.4 (-46.5 to -35.3)	-46.8 (-50.9 to -42.4)
France	1,350,000 (1,230,000 to 1,490,000)	2,945.4 (2,686.5 to 3,236.1)	6.6 (-2.5 to 16.4)	-29.7 (-34.9 to -24.7)	1,150,000 (1,050,000 to 1,250,000)	2,026.1 (1,835.8 to 2,246.0)	12.0 (2.8 to 22.6)	-23.8 (-28.5 to -18.7)
Germany	1,750,000 (1,580,000 to 1,930,000)	2,887.6 (2,620.0 to 3,184.7)	9.5 (-0.2 to 20.6)	-27.2 (-32.2 to -21.2)	1,310,000 (1,190,000 to 1,420,000)	1,973.0 (1,779.9 to 2,178.3)	-8.6 (-17.0 to 0.8)	-24.7 (-30.0 to -19.4)
Greece	248,000 (222,000 to 277,000)	3,135.4 (2,855.8 to 3,484.4)	7.7 (-2.3 to 20.4)	-24.1 (-29.5 to -17.0)	204,000 (185,000 to 224,000)	2,345.5 (2,130.2 to 2,588.1)	12.9 (3.2 to 23.5)	-21.3 (-26.1 to -16.2)
Iceland	6,670 (6,110 to 7,290)	3,102.7 (2,839.3 to 3,386.6)	20.7 (10.4 to 32.3)	-29.4 (-34.6 to -23.8)	5,250 (4,820 to 5,700)	2,251.8 (2,049.8 to 2,462.6)	-3.1 (-10.8 to 4.4)	-40.2 (-44.2 to -36.3)
Ireland	82,600 (75,300 to 89,400)	2,904.1 (2,646.9 to 3,174.5)	-8.3 (-16.7 to 0.4)	-43.7 (-48.5 to -38.7)	71,400 (65,400 to 78,800)	2,215.8 (2,019.3 to 2,445.7)	-7.9 (-14.8 to 0.3)	-41.3 (-45.1 to -37.2)
Israel	136,000 (124,000 to 151,000)	2,694.5 (2,466.0 to 2,984.0)	77.0 (62.5 to 95.1)	-18.3 (-24.5 to -10.8)	102,000 (91,600 to 114,000)	1,813.4 (1,633.5 to 2,034.8)	64.0 (51.7 to 77.9)	-26.1 (-31.0 to -21.0)

	Male				Female			
	2019		Percent change from 1990 to 2019		2019		Percent change from 1990 to 2019	
	Number of episodes	Age standardized incidence (per 100,000)	Number of episodes	Age standardized incidence	Number of episodes	Age standardized incidence (per 100,000)	Number of episodes	Age standardized incidence
Italy	1,360,000 (1,270,000 to 1,470,000)	2,903.0 (2,699.0 to 3,145.5)	37.1 (30.3 to 43.8)	-17.8 (-20.8 to - 14.7)	1,050,000 (971,000 to 1,130,000)	1,865.5 (1,713.0 to 2,043.5)	28.6 (21.9 to 35.1)	-22.1 (-24.8 to - 19.2)
Luxembourg	9,790 (8,850 to 10,900)	2,698.3 (2,449.8 to 2,983.3)	42.5 (30.0 to 55.2)	-26.3 (-31.9 to - 20.4)	7,810 (7,130 to 8,570)	1,910.9 (1,732.7 to 2,124.1)	26.6 (15.0 to 39.5)	-24.9 (-30.1 to - 19.6)
Malta	11,000 (9,960 to 12,300)	3,540.8 (3,240.5 to 3,883.9)	28.3 (15.1 to 43.9)	-28.3 (-34.0 to - 21.8)	8,890 (8,020 to 9,790)	2,549.5 (2,306.8 to 2,815.9)	17.1 (7.8 to 28.8)	-31.4 (-35.8 to - 26.1)
Monaco	1,030 (930 to 1,150)	3,248.8 (2,953.4 to 3,580.4)	24.8 (11.4 to 37.2)	-16.2 (-22.3 to -9.9)	871 (783 to 970)	2,336.6 (2,120.6 to 2,573.7)	13.1 (1.9 to 24.4)	-14.3 (-20.1 to - 7.9)
Netherlands	288,000 (258,000 to 321,000)	2,465.4 (2,214.1 to 2,743.7)	17.8 (6.7 to 29.1)	-26.5 (-32.8 to - 20.9)	239,000 (217,000 to 262,000)	1,777.1 (1,611.5 to 1,964.2)	6.5 (-2.0 to 15.4)	-24.3 (-29.1 to - 19.1)
Norway	121,000 (111,000 to 130,000)	3,553.8 (3,264.1 to 3,864.2)	-20.1 (-23.9 to - 16.6)	-40.4 (-42.8 to - 38.1)	94,800 (87,900 to 102,000)	2,415.6 (2,202.9 to 2,654.2)	-31.5 (-35.1 to - 27.8)	-41.3 (-43.8 to - 38.7)
Portugal	325,000 (295,000 to 358,000)	4,159.3 (3,794.9 to 4,576.2)	0.5 (-8.1 to 11.0)	-33.8 (-38.4 to - 28.4)	256,000 (231,000 to 279,000)	2,584.1 (2,345.6 to 2,841.6)	8.9 (-2.8 to 19.3)	-32.8 (-37.8 to - 28.0)
San Marino	620 (559 to 687)	2,798.7 (2,533.4 to 3,093.2)	39.5 (27.4 to 53.4)	-22.8 (-28.3 to - 17.3)	445 (404 to 488)	1,850.7 (1,655.2 to 2,044.7)	42.3 (30.5 to 54.7)	-18.5 (-23.5 to - 13.5)

	Male				Female			
	2019		Percent change from 1990 to 2019		2019		Percent change from 1990 to 2019	
	Number of episodes	Age standardized incidence (per 100,000)	Number of episodes	Age standardized incidence	Number of episodes	Age standardized incidence (per 100,000)	Number of episodes	Age standardized incidence
Spain	823,000 (748,000 to 896,000)	2,592.9 (2,367.5 to 2,830.9)	18.1 (7.4 to 29.0)	-27.3 (-32.9 to -21.8)	672,000 (615,000 to 743,000)	1,779.2 (1,597.1 to 1,979.0)	20.3 (7.9 to 33.9)	-25.1 (-30.5 to -19.2)
Sweden	227,000 (209,000 to 248,000)	3,320.4 (3,007.8 to 3,678.3)	-12.2 (-18.2 to -5.3)	-30.5 (-34.7 to -26.0)	177,000 (163,000 to 194,000)	2,522.7 (2,259.8 to 2,816.8)	-18.9 (-24.7 to -12.9)	-27.3 (-31.7 to -23.3)
Switzerland	124,000 (113,000 to 135,000)	2,114.5 (1,922.6 to 2,319.8)	-1.7 (-11.8 to 7.7)	-39.1 (-44.5 to -34.2)	99,200 (89,700 to 109,000)	1,485.3 (1,338.8 to 1,647.4)	-12.9 (-21.0 to -3.5)	-37.8 (-42.5 to -33.1)
United Kingdom	1,470,000 (1,370,000 to 1,580,000)	3,137.8 (2,922.7 to 3,391.1)	-3.5 (-6.8 to -0.4)	-36.8 (-38.3 to -35.0)	1,310,000 (1,210,000 to 1,400,000)	2,353.6 (2,184.4 to 2,545.1)	-24.5 (-26.9 to -22.3)	-39.3 (-40.8 to -38.0)
**Latin America and Caribbean**	**20,700,000 (19,400,000 to 22,100,000)**	**7,718.1 (7,213.4 to 8,238.2)**	**29.1 (22.9 to 35.4)**	**-24.7 (-26.5 to -22.8)**	**20,500,000 (19,200,000 to 21,900,000)**	**6,813.4 (6,370.4 to 7,297.4)**	**28.4 (22.3 to 34.9)**	**-32.2 (-33.6 to -30.8)**
Andean Latin America	2,790,000 (2,590,000 to 3,010,000)	9,789.2 (9,105.8 to 10,542.8)	33.6 (25.2 to 43.6)	-26.7 (-31.1 to -21.3)	2,610,000 (2,410,000 to 2,820,000)	8,749.2 (8,078.3 to 9,509.9)	33.2 (24.4 to 42.4)	-32.4 (-36.3 to -28.2)
Bolivia (Plurinational State of)	470,000 (431,000 to 511,000)	9,272.0 (8,515.6 to 10,048.0)	28.3 (17.3 to 39.4)	-30.8 (-35.4 to -25.3)	416,000 (379,000 to 454,000)	8,089.9 (7,389.9 to 8,846.3)	19.4 (10.9 to 30.1)	-37.1 (-41.5 to -31.7)
Ecuador	593,000 (550,000 to 642,000)	7,793.9 (7,238.1 to 8,444.4)	30.5 (20.9 to 40.6)	-29.0 (-34.2 to -23.5)	648,000 (598,000 to 705,000)	8,105.1 (7,472.1 to 8,834.3)	44.5 (32.1 to 58.4)	-29.4 (-35.2 to -22.5)

	Male				Female			
	2019		Percent change from 1990 to 2019		2019		Percent change from 1990 to 2019	
	Number of episodes	Age standardized incidence (per 100,000)	Number of episodes	Age standardized incidence	Number of episodes	Age standardized incidence (per 100,000)	Number of episodes	Age standardized incidence
Peru	1,730,000 (1,590,000 to 1,880,000)	10,806.1 (9,892.9 to 11,769.2)	36.2 (24.0 to 50.8)	-24.8 (-31.6 to -17.0)	1,540,000 (1,400,000 to 1,690,000)	9,203.3 (8,318.7 to 10,128.1)	32.9 (20.6 to 46.1)	-32.3 (-38.2 to -26.4)
Caribbean	1,580,000 (1,470,000 to 1,700,000)	6,809.9 (6,349.0 to 7,338.7)	21.9 (16.5 to 28.1)	-20.6 (-23.4 to -17.5)	1,540,000 (1,430,000 to 1,660,000)	6,019.0 (5,604.6 to 6,497.1)	16.2 (10.8 to 22.2)	-29.2 (-31.9 to -26.1)
Antigua and Barbuda	2,610 (2,380 to 2,850)	6,243.7 (5,709.7 to 6,824.8)	29.7 (19.3 to 42.6)	-20.9 (-27.2 to -13.9)	2,690 (2,450 to 2,930)	5,718.1 (5,229.6 to 6,227.0)	10.2 (1.7 to 19.6)	-24.4 (-29.8 to -18.4)
Bahamas	9,200 (8,470 to 9,950)	5,507.2 (5,072.7 to 5,977.4)	29.3 (20.1 to 38.8)	-31.5 (-36.5 to -26.4)	8,920 (8,130 to 9,720)	4,649.5 (4,239.8 to 5,080.1)	20.3 (11.1 to 31.2)	-36.5 (-41.5 to -30.6)
Barbados	11,900 (10,800 to 13,000)	6,752.7 (6,203.0 to 7,357.4)	24.0 (12.2 to 36.9)	-16.2 (-22.8 to -9.3)	12,600 (11,300 to 13,800)	5,857.9 (5,326.6 to 6,418.4)	13.6 (3.1 to 26.0)	-18.8 (-25.2 to -11.6)
Belize	10,700 (9,770 to 11,600)	6,596.0 (6,036.2 to 7,186.2)	79.0 (63.7 to 95.2)	-18.9 (-25.0 to -12.3)	9,050 (8,330 to 9,810)	5,760.3 (5,311.9 to 6,261.2)	51.9 (40.6 to 65.2)	-32.8 (-37.8 to -27.1)
Bermuda	1,880 (1,700 to 2,060)	4,696.5 (4,244.7 to 5,128.5)	16.8 (6.6 to 27.7)	-25.5 (-30.7 to -20.1)	2,220 (1,990 to 2,440)	4,287.8 (3,884.4 to 4,738.5)	7.3 (-3.0 to 18.2)	-34.1 (-39.1 to -28.8)
Cuba	491,000 (448,000 to 541,000)	6,626.8 (6,080.8 to 7,276.4)	30.4 (19.6 to 43.1)	-11.5 (-18.2 to -4.2)	486,000 (437,000 to 544,000)	5,803.5 (5,272.4 to 6,436.4)	29.7 (15.8 to 44.9)	-21.2 (-28.4 to -13.5)

	Male				Female			
	2019		Percent change from 1990 to 2019		2019		Percent change from 1990 to 2019	
	Number of episodes	Age standardized incidence (per 100,000)	Number of episodes	Age standardized incidence	Number of episodes	Age standardized incidence (per 100,000)	Number of episodes	Age standardized incidence
Dominica	2,330 (2,130 to 2,540)	6,197.1 (5,692.9 to 6,750.3)	-11.7 (-19.3 to -3.0)	-23.7 (-29.8 to -17.0)	2,050 (1,870 to 2,270)	5,062.7 (4,619.5 to 5,564.8)	-33.6 (-39.4 to -27.3)	-32.8 (-38.1 to -27.2)
Dominican Republic	319,000 (293,000 to 351,000)	6,557.3 (5,999.0 to 7,203.4)	31.0 (20.0 to 43.8)	-17.8 (-24.4 to -10.7)	291,000 (266,000 to 316,000)	5,813.0 (5,302.3 to 6,319.4)	22.7 (12.7 to 34.7)	-28.1 (-33.8 to -21.8)
Grenada	3,520 (3,210 to 3,840)	7,464.4 (6,842.3 to 8,105.5)	-0.1 (-8.9 to 8.5)	-23.1 (-28.6 to -17.5)	3,470 (3,160 to 3,810)	6,508.9 (5,941.6 to 7,115.6)	-21.1 (-27.6 to -14.0)	-33.1 (-38.3 to -27.6)
Guyana	20,500 (18,800 to 22,200)	6,592.2 (6,069.6 to 7,129.5)	-10.1 (-17.7 to -2.6)	-23.2 (-29.6 to -16.9)	18,000 (16,400 to 19,900)	5,385.9 (4,916.3 to 5,926.1)	-17.2 (-24.4 to -9.6)	-33.0 (-39.3 to -26.7)
Haiti	392,000 (357,000 to 427,000)	8,869.1 (8,117.3 to 9,702.0)	27.6 (18.7 to 36.7)	-27.6 (-32.2 to -22.2)	384,000 (352,000 to 418,000)	8,186.8 (7,489.7 to 8,920.7)	18.0 (10.2 to 26.7)	-33.5 (-38.1 to -28.4)
Jamaica	75,000 (68,500 to 81,900)	5,494.4 (4,992.5 to 6,011.7)	4.1 (-4.5 to 12.3)	-21.7 (-27.9 to -15.7)	71,000 (64,600 to 77,800)	4,789.2 (4,349.3 to 5,256.2)	-9.2 (-16.3 to 0.4)	-31.4 (-36.8 to -24.9)
Puerto Rico	121,000 (109,000 to 136,000)	5,257.8 (4,767.3 to 5,825.3)	-11.8 (-19.0 to -2.6)	-35.9 (-40.7 to -30.5)	136,000 (124,000 to 150,000)	4,720.5 (4,303.6 to 5,169.9)	-7.1 (-15.9 to 1.7)	-39.3 (-43.8 to -34.2)
Saint Kitts and Nevis	1,740 (1,570 to 1,920)	6,301.5 (5,734.4 to 6,879.4)	13.5 (4.0 to 23.7)	-28.3 (-33.8 to -22.7)	1,590 (1,460 to 1,740)	5,348.0 (4,911.4 to 5,803.3)	-13.8 (-21.2 to -4.3)	-40.3 (-44.8 to -34.8)

	Male				Female			
	2019		Percent change from 1990 to 2019		2019		Percent change from 1990 to 2019	
	Number of episodes	Age standardized incidence (per 100,000)	Number of episodes	Age standardized incidence	Number of episodes	Age standardized incidence (per 100,000)	Number of episodes	Age standardized incidence
Saint Lucia	5,960 (5,490 to 6,450)	6,811.9 (6,272.2 to 7,382.2)	39.6 (27.4 to 52.6)	-17.4 (-23.4 to -11.2)	5,720 (5,210 to 6,200)	5,758.1 (5,281.8 to 6,238.9)	21.5 (11.7 to 32.9)	-28.2 (-33.3 to -22.7)
Saint Vincent and the Grenadines	3,850 (3,500 to 4,210)	6,348.8 (5,797.0 to 6,918.5)	22.9 (11.3 to 35.3)	-14.8 (-22.0 to -7.7)	3,280 (2,990 to 3,610)	5,574.9 (5,079.9 to 6,119.0)	-0.5 (-9.5 to 9.2)	-20.5 (-27.5 to -13.5)
Suriname	15,100 (13,800 to 16,300)	5,675.8 (5,202.0 to 6,115.8)	42.4 (31.4 to 53.4)	-16.7 (-22.9 to -10.4)	14,200 (12,900 to 15,600)	4,695.0 (4,279.9 to 5,158.1)	34.8 (24.0 to 48.5)	-28.5 (-34.7 to -21.3)
Trinidad and Tobago	35,800 (32,600 to 38,800)	4,865.0 (4,421.7 to 5,270.3)	-7.8 (-15.5 to 0.9)	-38.5 (-43.3 to -32.9)	32,000 (29,400 to 34,900)	4,096.2 (3,746.3 to 4,480.8)	-14.3 (-21.3 to -7.0)	-44.0 (-48.2 to -39.2)
United States Virgin Islands	3,010 (2,720 to 3,320)	4,994.9 (4,539.0 to 5,449.6)	15.0 (5.4 to 25.1)	-16.7 (-22.1 to -11.4)	3,170 (2,840 to 3,520)	4,284.6 (3,871.4 to 4,712.0)	10.2 (0.8 to 21.7)	-28.4 (-32.7 to -23.0)
Central Latin America	6,690,000 (6,260,000 to 7,160,000)	6,000.8 (5,615.0 to 6,427.2)	19.7 (13.4 to 26.3)	-30.3 (-32.3 to -28.4)	6,720,000 (6,280,000 to 7,230,000)	5,477.5 (5,102.5 to 5,906.7)	16.4 (10.4 to 23.2)	-39.3 (-41.0 to -37.6)
Colombia	1,310,000 (1,200,000 to 1,420,000)	5,637.4 (5,156.0 to 6,166.6)	28.0 (16.0 to 41.7)	-28.2 (-34.2 to -21.9)	1,370,000 (1,240,000 to 1,500,000)	5,206.0 (4,721.8 to 5,753.9)	25.0 (13.7 to 38.0)	-39.7 (-44.4 to -34.6)
Costa Rica	162,000 (147,000 to 177,000)	7,234.3 (6,587.1 to 7,939.8)	60.9 (44.6 to 76.5)	-10.0 (-17.9 to -2.9)	150,000 (136,000 to 166,000)	6,062.7 (5,456.9 to 6,734.7)	46.8 (35.0 to 61.6)	-27.3 (-32.8 to -21.3)

	Male				Female			
	2019		Percent change from 1990 to 2019		2019		Percent change from 1990 to 2019	
	Number of episodes	Age standardized incidence (per 100,000)	Number of episodes	Age standardized incidence	Number of episodes	Age standardized incidence (per 100,000)	Number of episodes	Age standardized incidence
El Salvador	244,000 (223,000 to 268,000)	9,042.1 (8,248.6 to 9,942.4)	6.2 (-3.8 to 17.2)	-16.1 (-23.4 to -8.4)	277,000 (253,000 to 302,000)	8,202.2 (7,471.9 to 8,958.2)	10.0 (0.4 to 20.7)	-29.3 (-34.7 to -22.8)
Guatemala	661,000 (607,000 to 715,000)	10,233.5 (9,423.1 to 11,064.5)	16.3 (4.6 to 27.3)	-42.5 (-47.3 to -37.9)	652,000 (600,000 to 717,000)	9,186.4 (8,412.8 to 10,108.9)	12.4 (-0.6 to 24.0)	-52.0 (-56.2 to -47.9)
Honduras	266,000 (241,000 to 291,000)	7,124.7 (6,466.6 to 7,767.9)	34.9 (23.0 to 47.4)	-23.4 (-30.2 to -17.2)	253,000 (227,000 to 281,000)	6,479.8 (5,850.4 to 7,228.7)	24.7 (11.9 to 38.8)	-34.9 (-41.1 to -27.7)
Mexico	2,900,000 (2,700,000 to 3,110,000)	5,221.6 (4,877.5 to 5,590.7)	7.5 (2.0 to 13.4)	-36.0 (-37.8 to -34.1)	2,870,000 (2,670,000 to 3,090,000)	4,783.0 (4,450.1 to 5,150.7)	4.9 (-1.3 to 11.2)	-43.3 (-45.2 to -41.4)
Nicaragua	169,000 (155,000 to 186,000)	6,449.1 (5,929.5 to 7,037.5)	10.6 (0.9 to 22.4)	-25.6 (-31.0 to -19.7)	170,000 (155,000 to 188,000)	6,122.2 (5,556.5 to 6,732.5)	3.9 (-6.2 to 15.8)	-33.4 (-38.8 to -27.3)
Panama	143,000 (132,000 to 157,000)	7,090.1 (6,511.1 to 7,749.1)	103.9 (86.9 to 122.1)	-0.5 (-8.6 to 8.2)	134,000 (122,000 to 146,000)	6,352.2 (5,780.6 to 6,939.2)	90.6 (75.5 to 108.3)	-13.9 (-20.9 to -6.3)
Venezuela (Bolivarian Republic of)	846,000 (773,000 to 920,000)	6,465.9 (5,901.4 to 7,035.1)	51.3 (38.1 to 65.4)	-15.0 (-21.2 to -8.0)	847,000 (777,000 to 934,000)	5,829.4 (5,341.1 to 6,439.3)	48.6 (34.8 to 65.3)	-25.5 (-31.6 to -18.3)
Tropical Latin America	9,670,000 (9,040,000 to 10,300,000)	9,137.1 (8,539.5 to 9,778.8)	36.6 (28.8 to 44.2)	-20.9 (-23.6 to -18.0)	9,650,000 (9,010,000 to 10,300,000)	7,866.1 (7,334.9 to 8,420.1)	39.3 (31.2 to 47.6)	-27.1 (-29.4 to -24.7)

	Male				Female			
	2019		Percent change from 1990 to 2019		2019		Percent change from 1990 to 2019	
	Number of episodes	Age standardized incidence (per 100,000)	Number of episodes	Age standardized incidence	Number of episodes	Age standardized incidence (per 100,000)	Number of episodes	Age standardized incidence
Brazil	9,460,000 (8,840,000 to 10,100,000)	9,198.4 (8,595.6 to 9,847.3)	36.5 (28.8 to 44.2)	-21.1 (-23.7 to -18.2)	9,470,000 (8,850,000 to 10,100,000)	7,920.4 (7,389.6 to 8,472.5)	39.7 (31.6 to 48.0)	-27.2 (-29.5 to -24.8)
Paraguay	204,000 (188,000 to 221,000)	6,719.7 (6,200.8 to 7,274.9)	39.7 (28.6 to 51.1)	-17.1 (-23.2 to -10.8)	173,000 (159,000 to 189,000)	5,518.2 (5,072.9 to 6,021.0)	20.2 (10.7 to 29.9)	-30.0 (-35.1 to -24.6)
**North Africa and Middle East**	**17,800,000 (16,600,000 to 19,200,000)**	**6,653.1 (6,201.2 to 7,134.1)**	**18.4 (12.0 to 25.4)**	**-27.3 (-29.5 to -25.0)**	**16,400,000 (15,100,000 to 17,700,000)**	**6,365.7 (5,919.7 to 6,861.6)**	**7.3 (1.2 to 13.6)**	**-30.6 (-32.8 to -28.3)**
Afghanistan	1,220,000 (1,090,000 to 1,360,000)	7,914.8 (7,246.3 to 8,598.5)	94.3 (79.7 to 109.3)	-25.9 (-31.0 to -20.3)	1,330,000 (1,180,000 to 1,500,000)	8,162.3 (7,458.8 to 8,909.8)	93.9 (80.1 to 109.0)	-28.4 (-33.2 to -23.4)
Algeria	1,100,000 (1,010,000 to 1,210,000)	5,873.8 (5,396.9 to 6,403.9)	24.6 (14.1 to 35.4)	-25.7 (-30.9 to -20.3)	1,060,000 (972,000 to 1,150,000)	5,824.1 (5,351.8 to 6,318.6)	12.6 (4.3 to 22.5)	-28.5 (-32.7 to -23.7)
Bahrain	36,100 (32,400 to 39,900)	5,676.6 (5,143.6 to 6,227.0)	145.8 (121.6 to 171.6)	-19.5 (-25.1 to -13.7)	24,300 (21,900 to 26,800)	5,724.4 (5,147.2 to 6,313.8)	91.3 (75.8 to 108.7)	-18.4 (-23.4 to -13.0)
Egypt	3,890,000 (3,560,000 to 4,230,000)	8,599.1 (7,905.5 to 9,350.2)	10.4 (1.1 to 21.0)	-31.3 (-36.4 to -25.5)	3,110,000 (2,840,000 to 3,410,000)	7,626.2 (6,977.3 to 8,299.9)	-7.6 (-15.3 to 0.2)	-36.7 (-40.9 to -31.7)
Iran (Islamic Republic of)	2,060,000 (1,920,000 to 2,220,000)	5,339.5 (4,971.7 to 5,754.8)	-0.9 (-7.4 to 6.2)	-33.5 (-35.6 to -31.3)	1,880,000 (1,740,000 to 2,030,000)	5,044.3 (4,670.6 to 5,457.7)	-10.2 (-16.8 to -3.3)	-35.1 (-37.5 to -32.7)

	Male				Female			
	**2019**		**Percent change from 1990 to 2019**		**2019**		**Percent change from 1990 to 2019**	
	**Number of episodes**	**Age standardized incidence (per 100,000)**	**Number of episodes**	**Age standardized incidence**	**Number of episodes**	**Age standardized incidence (per 100,000)**	**Number of episodes**	**Age standardized incidence**
Iraq	991,000 (900,000 to 1,090,000)	5,556.6 (5,088.5 to 6,078.6)	23.1 (11.7 to 35.5)	-38.4 (-42.9 to -33.5)	981,000 (889,000 to 1,080,000)	5,523.4 (5,071.9 to 6,001.3)	24.5 (13.3 to 38.0)	-37.0 (-41.6 to -31.6)
Jordan	297,000 (269,000 to 331,000)	5,899.0 (5,418.8 to 6,492.7)	124.6 (107.6 to 144.1)	-28.8 (-34.0 to -22.9)	237,000 (213,000 to 262,000)	5,205.8 (4,733.9 to 5,742.0)	85.8 (71.9 to 102.0)	-35.3 (-39.6 to -30.3)
Kuwait	116,000 (107,000 to 127,000)	6,609.2 (6,062.9 to 7,277.9)	124.2 (108.4 to 143.3)	-5.1 (-11.6 to 1.9)	94,700 (86,500 to 104,000)	6,499.7 (5,930.4 to 7,140.1)	107.9 (90.3 to 126.5)	-15.5 (-21.4 to -9.9)
Lebanon	141,000 (129,000 to 156,000)	5,766.7 (5,258.4 to 6,336.8)	29.9 (20.4 to 41.1)	-18.6 (-24.3 to -12.3)	145,000 (131,000 to 160,000)	5,456.7 (4,906.3 to 6,023.9)	35.9 (25.2 to 47.7)	-20.3 (-25.6 to -14.1)
Libya	177,000 (162,000 to 192,000)	6,074.3 (5,581.9 to 6,610.0)	12.2 (2.1 to 24.0)	-24.9 (-29.5 to -18.7)	164,000 (150,000 to 178,000)	5,896.3 (5,362.4 to 6,431.8)	6.5 (-4.0 to 17.8)	-25.7 (-30.8 to -20.2)
Morocco	1,020,000 (942,000 to 1,110,000)	6,371.2 (5,865.2 to 6,901.1)	0.5 (-8.0 to 11.0)	-27.5 (-32.5 to -21.6)	1,050,000 (962,000 to 1,140,000)	6,423.6 (5,900.3 to 7,000.3)	-9.0 (-16.5 to -0.8)	-31.3 (-35.7 to -26.4)
Oman	109,000 (98,200 to 120,000)	6,347.9 (5,763.9 to 6,929.8)	40.0 (28.4 to 53.6)	-32.3 (-37.5 to -26.9)	74,600 (67,300 to 82,700)	6,255.5 (5,721.4 to 6,848.1)	5.7 (-4.2 to 16.0)	-33.8 (-38.4 to -28.6)
Palestine	146,000 (132,000 to 161,000)	7,245.1 (6,638.4 to 7,909.0)	24.6 (14.3 to 35.7)	-37.3 (-41.9 to -32.5)	138,000 (124,000 to 153,000)	6,701.0 (6,172.2 to 7,294.1)	30.4 (17.8 to 42.3)	-34.2 (-39.1 to -29.3)

	Male				Female			
	2019		Percent change from 1990 to 2019		2019		Percent change from 1990 to 2019	
	Number of episodes	Age standardized incidence (per 100,000)	Number of episodes	Age standardized incidence	Number of episodes	Age standardized incidence (per 100,000)	Number of episodes	Age standardized incidence
Qatar	68,500 (60,800 to 77,200)	5,345.3 (4,823.8 to 5,922.5)	425.4 (382.9 to 475.6)	-18.2 (-23.7 to -12.1)	28,700 (25,600 to 31,900)	5,761.5 (5,245.3 to 6,260.3)	221.3 (195.5 to 249.2)	-20.1 (-25.1 to -14.4)
Saudi Arabia	1,040,000 (949,000 to 1,140,000)	6,979.1 (6,361.8 to 7,664.6)	53.1 (39.0 to 69.0)	-28.8 (-34.1 to -23.1)	836,000 (754,000 to 923,000)	7,288.9 (6,644.9 to 7,976.3)	36.6 (23.4 to 51.7)	-28.6 (-33.8 to -22.7)
Sudan	1,060,000 (957,000 to 1,170,000)	6,251.0 (5,706.5 to 6,802.7)	12.5 (3.6 to 22.5)	-32.5 (-37.4 to -27.4)	966,000 (878,000 to 1,080,000)	5,873.9 (5,425.3 to 6,404.1)	4.1 (-4.6 to 12.5)	-36.5 (-40.8 to -32.2)
Syrian Arab Republic	439,000 (402,000 to 477,000)	6,725.0 (6,166.4 to 7,309.5)	-17.0 (-24.9 to -8.3)	-16.4 (-22.0 to -9.7)	412,000 (377,000 to 450,000)	6,338.4 (5,799.1 to 6,887.7)	-22.3 (-30.2 to -13.3)	-21.4 (-26.9 to -15.5)
Tunisia	331,000 (300,000 to 361,000)	5,970.5 (5,422.8 to 6,535.0)	11.5 (1.8 to 22.6)	-21.9 (-27.3 to -16.4)	356,000 (325,000 to 390,000)	6,245.4 (5,667.0 to 6,868.9)	11.1 (0.6 to 22.7)	-24.4 (-30.2 to -17.8)
Turkey	2,360,000 (2,170,000 to 2,590,000)	6,130.9 (5,611.2 to 6,727.4)	8.8 (-1.9 to 19.7)	-22.4 (-29.2 to -15.8)	2,340,000 (2,140,000 to 2,550,000)	5,729.5 (5,256.8 to 6,286.8)	3.3 (-7.1 to 15.3)	-28.6 (-34.4 to -22.5)
United Arab Emirates	225,000 (201,000 to 251,000)	6,632.7 (6,052.8 to 7,201.6)	324.5 (283.1 to 370.5)	-20.8 (-26.0 to -14.7)	94,700 (84,500 to 105,000)	6,297.6 (5,738.1 to 6,879.0)	157.6 (133.8 to 184.3)	-20.9 (-26.4 to -14.8)
Yemen	983,000 (894,000 to 1,080,000)	8,084.9 (7,480.0 to 8,873.9)	28.4 (16.7 to 41.0)	-29.1 (-33.5 to -23.7)	1,020,000 (918,000 to 1,120,000)	8,028.9 (7,346.0 to 8,741.5)	14.9 (5.0 to 26.6)	-34.4 (-39.1 to -29.3)

	Male				Female			
	2019		Percent change from 1990 to 2019		2019		Percent change from 1990 to 2019	
	Number of episodes	Age standardized incidence (per 100,000)	Number of episodes	Age standardized incidence	Number of episodes	Age standardized incidence (per 100,000)	Number of episodes	Age standardized incidence
**South Asia**	**91,300,000 (84,700,000 to 98,500,000)**	**11,468.0 (10,682.7 to 12,342.4)**	**37.6 (31.6 to 43.9)**	**-19.1 (-21.3 to -17.0)**	**83,700,000 (78,100,000 to 89,800,000)**	**10,542.4 (9,844.2 to 11,281.7)**	**39.0 (33.6 to 44.7)**	**-24.1 (-25.9 to -22.3)**
Bangladesh	5,710,000 (5,230,000 to 6,170,000)	7,811.7 (7,192.7 to 8,430.4)	-17.3 (-23.8 to -9.8)	-45.9 (-50.1 to -41.7)	5,020,000 (4,620,000 to 5,450,000)	6,926.7 (6,382.8 to 7,492.2)	-5.4 (-13.7 to 3.5)	-41.7 (-46.0 to -36.4)
Bhutan	23,300 (21,300 to 25,500)	6,905.5 (6,307.1 to 7,564.4)	-16.9 (-24.2 to -8.9)	-35.1 (-39.3 to -30.2)	20,100 (18,400 to 22,000)	6,285.5 (5,757.5 to 6,881.9)	-18.3 (-25.1 to -11.3)	-38.1 (-42.4 to -33.7)
India	76,600,000 (71,000,000 to 82,600,000)	12,307.8 (11,449.9 to 13,277.9)	44.5 (38.0 to 51.2)	-17.2 (-19.7 to -14.8)	71,800,000 (67,000,000 to 77,100,000)	11,451.4 (10,700.0 to 12,274.5)	43.5 (37.8 to 49.7)	-23.6 (-25.6 to -21.6)
Nepal	1,200,000 (1,090,000 to 1,310,000)	9,234.6 (8,407.7 to 10,038.2)	-4.6 (-12.5 to 3.7)	-37.1 (-41.9 to -32.0)	1,200,000 (1,100,000 to 1,320,000)	8,566.0 (7,825.1 to 9,364.1)	1.2 (-7.4 to 11.5)	-40.2 (-44.7 to -34.8)
Pakistan	7,730,000 (7,090,000 to 8,440,000)	8,304.8 (7,700.4 to 8,914.6)	50.4 (44.1 to 57.3)	-14.5 (-17.6 to -11.0)	5,680,000 (5,190,000 to 6,250,000)	6,136.6 (5,669.1 to 6,622.2)	53.9 (47.1 to 61.6)	-18.2 (-21.6 to -14.8)
**Southeast Asia, East Asia, and Oceania**	**55,600,000 (51,800,000 to 59,600,000)**	**5,293.0 (4,909.3 to 5,672.2)**	**-3.8 (-8.8 to 1.1)**	**-31.0 (-33.4 to -29.0)**	**46,700,000 (43,300,000 to 50,200,000)**	**4,298.9 (3,966.4 to 4,642.0)**	**-13.2 (-17.4 to -8.7)**	**-40.4 (-42.3 to -38.5)**
East Asia	32,600,000 (30,200,000 to 35,000,000)	4,382.2 (4,038.6 to 4,699.0)	-14.6 (-20.0 to -9.9)	-37.5 (-40.2 to -35.2)	26,900,000 (24,800,000 to 29,000,000)	3,535.3 (3,231.4 to 3,850.8)	-26.3 (-30.5 to -22.0)	-49.0 (-51.3 to -46.8)

	Male				Female			
	2019		Percent change from 1990 to 2019		2019		Percent change from 1990 to 2019	
	Number of episodes	Age standardized incidence (per 100,000)	Number of episodes	Age standardized incidence	Number of episodes	Age standardized incidence (per 100,000)	Number of episodes	Age standardized incidence
China	30,700,000 (28,400,000 to 33,000,000)	4,267.0 (3,929.5 to 4,587.0)	-16.2 (-21.5 to -11.4)	-38.5 (-41.2 to -36.2)	25,200,000 (23,200,000 to 27,200,000)	3,443.1 (3,147.9 to 3,753.4)	-28.0 (-32.2 to -23.7)	-50.0 (-52.3 to -47.8)
Democratic People's Republic of Korea	770,000 (702,000 to 837,000)	6,580.3 (6,033.9 to 7,140.4)	-1.2 (-10.1 to 8.2)	-24.7 (-29.9 to -18.8)	805,000 (738,000 to 878,000)	5,590.9 (5,131.2 to 6,109.3)	-6.0 (-13.8 to 2.8)	-30.9 (-35.7 to -25.8)
Taiwan (Province of China)	1,140,000 (1,040,000 to 1,250,000)	8,084.7 (7,372.7 to 8,821.6)	46.9 (33.6 to 62.4)	-7.1 (-14.1 to 0.7)	890,000 (806,000 to 972,000)	6,312.9 (5,709.8 to 6,925.8)	39.7 (27.9 to 52.2)	-16.9 (-21.8 to -11.3)
Oceania	473,000 (431,000 to 518,000)	8,135.7 (7,531.1 to 8,816.7)	61.1 (53.4 to 69.6)	-19.8 (-23.4 to -15.9)	418,000 (379,000 to 463,000)	7,291.7 (6,702.6 to 7,894.9)	63.9 (56.9 to 72.6)	-19.3 (-22.3 to -15.9)
American Samoa	1,530 (1,400 to 1,670)	6,064.9 (5,557.4 to 6,574.6)	-4.5 (-12.7 to 3.7)	-21.2 (-27.2 to -14.7)	1,520 (1,390 to 1,660)	5,847.7 (5,349.1 to 6,377.7)	5.2 (-2.2 to 12.9)	-15.0 (-20.0 to -9.6)
Cook Islands	759 (699 to 825)	7,702.7 (7,121.5 to 8,336.9)	-20.6 (-27.5 to -12.8)	-28.3 (-33.5 to -22.7)	607 (558 to 662)	6,109.2 (5,594.4 to 6,667.7)	-23.6 (-30.1 to -16.2)	-34.8 (-39.5 to -29.5)
Fiji	26,700 (24,300 to 29,100)	6,544.0 (6,,021.1 to 7,085.4)	-4.3 (-11.6 to 4.2)	-24.0 (-29.1 to -18.1)	22,900 (21,100 to 24,800)	5,550.6 (5,129.3 to 5,989.2)	-3.3 (-10.4 to 4.2)	-23.1 (-27.9 to -18.1)
Guam	4,860 (4,460 to 5,310)	5,551.1 (5,100.9 to 6,054.7)	22.7 (13.8 to 32.7)	-15.9 (-21.9 to -10.3)	4,340 (3,970 to 4,690)	4,966.5 (4,535.0 to 5,383.6)	24.1 (15.5 to 33.9)	-22.0 (-27.1 to -16.3)

	Male				Female			
	2019		Percent change from 1990 to 2019		2019		Percent change from 1990 to 2019	
	Number of episodes	Age standardized incidence (per 100,000)	Number of episodes	Age standardized incidence	Number of episodes	Age standardized incidence (per 100,000)	Number of episodes	Age standardized incidence
Kiribati	3,450 (3,160 to 3,800)	7,474.4 (6,907.6 to 8,162.7)	17.3 (8.5 to 27.4)	-18.9 (-25.3 to -12.4)	3,470 (3,150 to 3,810)	6,402.9 (5,864.6 to 6,954.5)	21.3 (11.7 to 32.4)	-20.6 (-26.5 to -14.4)
Marshall Islands	1,980 (1,820 to 2,170)	8,229.4 (7,606.9 to 8,926.0)	-0.7 (-8.4 to 7.8)	-20.5 (-26.0 to -14.8)	1,760 (1,600 to 1,920)	7,391.7 (6,759.4 to 7,977.7)	-1.8 (-8.7 to 6.2)	-19.2 (-24.1 to -14.2)
Micronesia (Federated States of)	3,410 (3,130 to 3,730)	7,902.6 (7,292.8 to 8,564.5)	-29.2 (-35.3 to -23.0)	-24.6 (-29.7 to -18.8)	3,060 (2,800 to 3,350)	6,881.3 (6,316.6 to 7,445.0)	-31.0 (-36.7 to -24.3)	-27.3 (-32.2 to -21.2)
Nauru	324 (294 to 359)	7,836.1 (7,180.6 to 8,513.1)	-23.4 (-29.3 to -16.7)	-15.6 (-21.3 to -9.3)	300 (272 to 331)	6,683.4 (6,173.0 to 7,226.0)	-21.3 (-27.0 to -14.7)	-17.7 (-23.3 to -11.7)
Niue	57 (52 to 62)	6,646.4 (6,110.6 to 7,196.0)	-40.1 (-44.9 to -35.5)	-22.1 (-27.2 to -17.2)	53 (49 to 58)	5,781.1 (5,307.8 to 6,258.3)	-41.2 (-44.9 to -37.2)	-19.2 (-23.8 to -14.1)
Northern Mariana Islands	1,400 (1,260 to 1,530)	6,395.9 (5,879.5 to 6,973.1)	-2.9 (-11.3 to 8.9)	-17.5 (-22.8 to -11.0)	1,030 (951 to 1,120)	5,276.1 (4,861.5 to 5,739.8)	-15.0 (-22.4 to -5.5)	-21.0 (-26.0 to -15.9)
Palau	999 (913 to 1,090)	10,427.7 (9,640.0 to 11,278.8)	7.6 (-1.3 to 18.2)	-22.3 (-27.5 to -16.2)	804 (741 to 872)	10,172.8 (9,399.2 to 10,987.7)	-10.8 (-18.4 to -2.3)	-23.1 (-28.7 to -17.3)
Papua New Guinea	355,000 (321,000 to 393,000)	8,217.4 (7,547.7 to 8,984.4)	87.3 (75.2 to 100.6)	-19.0 (-23.9 to -13.5)	314,000 (281,000 to 352,000)	7,493.7 (6,856.4 to 8,154.1)	91.3 (80.3 to 104.3)	-18.4 (-22.8 to -13.2)

	Male				Female			
	2019		Percent change from 1990 to 2019		2019		Percent change from 1990 to 2019	
	Number of episodes	Age standardized incidence (per 100,000)	Number of episodes	Age standardized incidence	Number of episodes	Age standardized incidence (per 100,000)	Number of episodes	Age standardized incidence
Samoa	7,080 (6,540 to 7,670)	7,738.1 (7,133.9 to 8,390.1)	-0.5 (-8.2 to 8.2)	-21.9 (-27.9 to -15.8)	6,820 (6,230 to 7,410)	7,400.6 (6,806.2 to 8,005.1)	7.3 (-0.5 to 15.2)	-17.5 (-22.7 to -11.8)
Solomon Islands	29,000 (26,500 to 31,600)	11,696.3 (10,790.9 to 12,613.5)	28.4 (19.5 to 37.3)	-29.2 (-34.0 to -23.9)	25,700 (23,300 to 28,700)	9,559.8 (8,732.0 to 10,428.4)	31.1 (21.0 to 42.1)	-31.1 (-35.8 to -25.9)
Tokelau	46 (42 to 50)	6,642.6 (6,104.9 to 7,211.1)	-36.8 (-41.3 to -31.9)	-26.4 (-31.5 to -21.4)	41 (37 to 44)	6,039.7 (5,526.7 to 6,566.1)	-46.9 (-50.5 to -42.9)	-29.5 (-34.1 to -24.7)
Tonga	3,240 (2,970 to 3,540)	7,240.3 (6,676.0 to 7,847.5)	-20.4 (-25.5 to -14.6)	-28.4 (-33.5 to -22.3)	3,030 (2,770 to 3,300)	6,073.3 (5,583.4 to 6,615.8)	-19.2 (-24.6 to -13.5)	-32.0 (-36.7 to -26.8)
Tuvalu	371 (340 to 403)	6,840.0 (6,289.1 to 7,420.1)	-9.7 (-17.2 to -1.8)	-30.1 (-35.2 to -24.3)	327 (302 to 354)	6,122.4 (5,652.4 to 6,609.7)	-24.6 (-30.5 to -18.0)	-33.8 (-38.4 to -28.4)
Vanuatu	9,980 (9,130 to 10,900)	7,865.7 (7,230.7 to 8,492.9)	61.0 (49.9 to 73.4)	-15.6 (-21.3 to -9.2)	8,740 (7,980 to 9,560)	6,878.7 (6,342.2 to 7,438.9)	54.8 (44.1 to 67.1)	-20.5 (-25.1 to -14.8)
Southeast Asia	22,600,000 (21,000,000 to 24,100,000)	7,654.4 (7,156.7 to 8,186.5)	16.4 (10.3 to 22.9)	-20.3 (-22.9 to -17.4)	19,400,000 (18,100,000 to 20,800,000)	6,107.9 (5,695.5 to 6,566.9)	13.7 (7.8 to 20.2)	-24.1 (-26.3 to -21.7)
Cambodia	663,000 (612,000 to 717,000)	10,311.7 (9,491.5 to 11,170.0)	-1.0 (-9.8 to 8.6)	-32.8 (-37.3 to -27.4)	637,000 (587,000 to 690,000)	8,541.5 (7,874.7 to 9,281.2)	-10.0 (-17.8 to -1.2)	-41.3 (-45.0 to -36.7)

	Male				Female			
	2019		Percent change from 1990 to 2019		2019		Percent change from 1990 to 2019	
	Number of episodes	Age standardized incidence (per 100,000)	Number of episodes	Age standardized incidence	Number of episodes	Age standardized incidence (per 100,000)	Number of episodes	Age standardized incidence
Indonesia	8,350,000 (7,700,000 to 9,050,000)	7,559.4 (7,013.4 to 8,195.0)	11.2 (4.8 to 18.2)	-19.3 (-22.9 to -15.1)	7,030,000 (6,500,000 to 7,610,000)	6,146.1 (5,690.2 to 6,649.9)	12.7 (5.8 to 20.2)	-16.9 (-20.6 to -13.1)
Lao People's Democratic Republic	223,000 (204,000 to 244,000)	7,808.8 (7,178.6 to 8,494.6)	-10.0 (-17.8 to -2.3)	-38.8 (-43.0 to -34.3)	202,000 (185,000 to 219,000)	6,804.2 (6,234.5 to 7,408.4)	-15.6 (-22.4 to -8.3)	-42.9 (-46.9 to -38.6)
Malaysia	1,370,000 (1,260,000 to 1,490,000)	9,571.3 (8,843.8 to 10,475.5)	96.3 (80.1 to 113.8)	-0.3 (-7.5 to 7.9)	1,060,000 (972,000 to 1,150,000)	7,770.2 (7,110.4 to 8,399.8)	75.7 (62.0 to 91.2)	-7.2 (-14.0 to 0.6)
Maldives	13,000 (11,700 to 14,400)	5,337.0 (4,862.8 to 5,892.4)	76.9 (60.6 to 95.3)	-26.4 (-31.0 to -21.2)	7,770 (6,990 to 8,650)	4,453.4 (4,042.2 to 4,912.7)	13.8 (3.2 to 24.5)	-39.8 (-44.5 to -35.1)
Mauritius	37,600 (34,600 to 41,300)	5,875.9 (5,392.9 to 6,459.5)	-7.9 (-16.5 to -0.1)	-36.0 (-41.4 to -30.7)	31,100 (28,700 to 34,000)	4,511.7 (4,129.9 to 4,962.2)	-10.7 (-17.6 to -3.1)	-37.5 (-42.3 to -32.7)
Myanmar	1,490,000 (1,370,000 to 1,620,000)	6,556.9 (6,060.8 to 7,093.9)	-22.0 (-28.6 to -15.0)	-38.5 (-43.2 to -33.5)	1,360,000 (1,250,000 to 1,480,000)	5,182.3 (4,752.4 to 5,633.7)	-23.0 (-30.0 to -16.0)	-44.9 (-49.0 to -40.4)
Philippines	4,490,000 (4,190,000 to 4,810,000)	10,114.1 (9,470.3 to 10,822.5)	25.8 (19.7 to 33.1)	-19.3 (-22.0 to -16.2)	3,990,000 (3,740,000 to 4,260,000)	8,382.7 (7,881.9 to 8,947.3)	25.3 (19.7 to 32.2)	-25.3 (-27.3 to -23.1)
Seychelles	4,880 (4,470 to 5,370)	9,585.8 (8,782.5 to 10,517.0)	45.1 (32.9 to 57.6)	-13.5 (-19.8 to -6.2)	3,810 (3,500 to 4,150)	7,483.7 (6,869.6 to 8,137.2)	24.0 (15.2 to 34.0)	-14.0 (-19.4 to -7.8)

	Male				Female			
	2019		Percent change from 1990 to 2019		2019		Percent change from 1990 to 2019	
	Number of episodes	Age standardized incidence (per 100,000)	Number of episodes	Age standardized incidence	Number of episodes	Age standardized incidence (per 100,000)	Number of episodes	Age standardized incidence
Sri Lanka	618,000 (567,000 to 672,000)	5,902.9 (5,420.3 to 6,419.1)	2.3 (-5.8 to 10.7)	-29.0 (-34.0 to -23.8)	555,000 (508,000 to 606,000)	4,701.0 (4,283.9 to 5,130.5)	3.2 (-5.2 to 12.7)	-37.0 (-42.0 to -31.9)
Thailand	2,530,000 (2,290,000 to 2,750,000)	6,497.2 (5,935.5 to 7,026.4)	55.8 (39.3 to 70.7)	-6.5 (-13.8 to 0.6)	2,250,000 (2,060,000 to 2,460,000)	5,277.4 (4,839.9 to 5,752.5)	52.8 (39.0 to 67.9)	-12.8 (-19.1 to -6.0)
Timor-Leste	43,400 (39,600 to 47,500)	7,786.7 (7,129.2 to 8,500.4)	7.1 (-2.7 to 17.8)	-26.8 (-32.2 to -21.0)	37,700 (34,300 to 41,300)	6,744.2 (6,210.9 to 7,332.3)	-9.3 (-17.4 to 0.4)	-40.4 (-44.7 to -35.5)
Viet Nam	2,720,000 (2,480,000 to 2,940,000)	6,532.6 (5,991.7 to 7,067.9)	10.7 (0.6 to 20.6)	-23.0 (-28.1 to -17.4)	2,190,000 (2,000,000 to 2,390,000)	4,647.2 (4,233.5 to 5,079.4)	0.1 (-7.7 to 9.7)	-31.0 (-35.7 to -26.1)
**Sub-Saharan Africa**	**37,300,000 (34,500,000 to 40,400,000)**	**10,066.6 (9,424.5 to 10,750.7)**	**44.2 (40.0 to 48.7)**	**-22.4 (-23.8 to -20.8)**	**34,700,000 (32,200,000 to 37,800,000)**	**8,232.3 (7,721.8 to 8,794.4)**	**38.7 (34.5 to 43.0)**	**-26.9 (-28.3 to -25.5)**
Central Sub-Saharan Africa	4,500,000 (4,090,000 to 4,930,000)	10,443.0 (9,607.8 to 11,271.3)	34.3 (26.2 to 42.8)	-32.7 (-36.3 to -28.5)	4,370,000 (4,000,000 to 4,750,000)	8,702.4 (8,063.1 to 9,408.8)	37.4 (29.2 to 45.1)	-33.0 (-35.9 to -29.4)
Angola	813,000 (747,000 to 897,000)	8,470.6 (7,821.2 to 9,140.0)	35.6 (24.2 to 46.5)	-41.0 (-45.5 to -36.0)	775,000 (709,000 to 847,000)	6,589.1 (6,112.7 to 7,086.1)	36.5 (26.7 to 48.1)	-46.2 (-49.9 to -41.6)
Central African Republic	219,000 (198,000 to 244,000)	12,067.5 (11,064.3 to 13,265.1)	44.6 (34.8 to 55.6)	-16.5 (-22.3 to -10.2)	217,000 (198,000 to 239,000)	10,145.4 (9,298.2 to 11,084.1)	50.4 (39.4 to 61.7)	-14.8 (-20.7 to -8.7)

	Male				Female			
	2019		Percent change from 1990 to 2019		2019		Percent change from 1990 to 2019	
	Number of episodes	Age standardized incidence (per 100,000)	Number of episodes	Age standardized incidence	Number of episodes	Age standardized incidence (per 100,000)	Number of episodes	Age standardized incidence
Congo	152,000 (139,000 to 166,000)	8,406.8 (7,720.9 to 9,151.2)	34.7 (24.4 to 44.9)	-33.0 (-38.0 to -27.9)	155,000 (142,000 to 168,000)	7,648.3 (7,051.5 to 8,358.3)	35.5 (26.3 to 45.8)	-27.6 (-32.5 to -22.1)
Democratic Republic of the Congo	3,220,000 (2,920,000 to 3,550,000)	11,185.1 (10,182.0 to 12,172.5)	33.9 (24.2 to 44.6)	-30.6 (-35.2 to -25.1)	3,140,000 (2,850,000 to 3,440,000)	9,435.2 (8,678.0 to 10,314.2)	37.8 (27.8 to 48.3)	-30.3 (-34.4 to -25.5)
Equatorial Guinea	34,700 (31,600 to 38,100)	7,815.2 (7,211.7 to 8,500.5)	19.9 (8.3 to 33.3)	-53.0 (-56.7 to -48.4)	28,400 (25,900 to 31,100)	6,162.7 (5,671.2 to 6,688.3)	6.4 (-2.9 to 16.3)	-52.3 (-55.9 to -48.2)
Gabon	58,200 (53,400 to 63,200)	9,299.3 (8,511.9 to 10,071.8)	16.3 (6.8 to 26.5)	-29.1 (-34.5 to -22.8)	49,000 (45,200 to 53,300)	6,749.9 (6,211.1 to 7,380.4)	5.9 (-2.1 to 14.0)	-33.6 (-38.6 to -28.9)
Eastern Sub-Saharan Africa	13,600,000 (12,600,000 to 14,800,000)	9,374.8 (8,770.8 to 10,007.7)	31.5 (27.1 to 36.0)	-28.5 (-30.2 to -26.7)	12,400,000 (11,400,000 to 13,500,000)	7,633.0 (7,155.7 to 8,154.9)	25.9 (21.8 to 30.1)	-32.8 (-34.4 to -31.1)
Burundi	400,000 (362,000 to 444,000)	9,411.6 (8,587.9 to 10,243.0)	35.2 (25.3 to 46.1)	-30.1 (-35.5 to -24.4)	384,000 (345,000 to 425,000)	8,131.2 (7,462.6 to 8,854.9)	31.3 (22.5 to 41.2)	-27.9 (-32.8 to -22.9)
Comoros	24,700 (22,700 to 26,800)	8,509.8 (7,864.4 to 9,195.5)	-9.5 (-16.1 to -1.4)	-37.5 (-41.9 to -32.7)	26,400 (24,200 to 28,700)	8,194.0 (7,571.5 to 8,898.7)	-6.4 (-13.5 to 1.9)	-36.0 (-40.1 to -31.0)
Djibouti	41,200 (37,600 to 45,100)	8,449.8 (7,803.4 to 9,166.5)	66.3 (53.6 to 81.6)	-27.0 (-31.8 to -21.7)	30,400 (27,600 to 33,200)	6,860.8 (6,311.6 to 7,429.5)	57.5 (44.5 to 70.3)	-29.3 (-34.1 to -24.5)

	Male				Female			
	2019		Percent change from 1990 to 2019		2019		Percent change from 1990 to 2019	
	Number of episodes	Age standardized incidence (per 100,000)	Number of episodes	Age standardized incidence	Number of episodes	Age standardized incidence (per 100,000)	Number of episodes	Age standardized incidence
Eritrea	284,000 (257,000 to 313,000)	12,209.3 (11,217.7 to 13,392.6)	46.8 (36.3 to 57.7)	-24.1 (-29.0 to -19.4)	249,000 (225,000 to 275,000)	9,311.6 (8,510.9 to 10,200.3)	43.4 (32.6 to 54.9)	-28.2 (-32.6 to -23.6)
Ethiopia	3,620,000 (3,320,000 to 3,960,000)	9,192.1 (8,555.2 to 9,898.1)	15.6 (10.6 to 21.2)	-37.4 (-40.0 to -34.3)	3,010,000 (2,760,000 to 3,310,000)	7,411.2 (6,918.4 to 7,968.3)	4.5 (-0.2 to 9.8)	-40.7 (-43.1 to -38.0)
Kenya	1,630,000 (1,510,000 to 1,750,000)	9,571.6 (8,970.0 to 10,229.1)	70.5 (62.4 to 78.5)	-11.2 (-13.2 to -9.3)	1,420,000 (1,310,000 to 1,540,000)	7,466.5 (6,971.4 to 7,993.3)	46.8 (40.5 to 52.7)	-24.7 (-26.1 to -23.3)
Madagascar	964,000 (879,000 to 1,050,000)	10,069.2 (9,222.6 to 10,929.3)	21.8 (11.8 to 33.4)	-31.5 (-36.9 to -25.5)	924,000 (840,000 to 1,010,000)	8,740.9 (8,097.0 to 9,486.2)	31.4 (20.4 to 44.1)	-29.5 (-35.6 to -22.6)
Malawi	569,000 (517,000 to 624,000)	9,289.9 (8,577.3 to 10,103.9)	18.5 (9.6 to 27.6)	-24.1 (-29.4 to -18.2)	509,000 (461,000 to 563,000)	6,897.8 (6,323.0 to 7,506.6)	4.1 (-4.3 to 13.2)	-37.1 (-41.9 to -32.1)
Mozambique	817,000 (741,000 to 901,000)	8,621.5 (7,899.9 to 9,409.9)	34.6 (23.5 to 46.4)	-22.0 (-28.1 to -15.5)	771,000 (701,000 to 849,000)	6,576.6 (6,040.9 to 7,102.4)	28.1 (18.6 to 38.4)	-31.7 (-36.7 to -26.5)
Rwanda	420,000 (383,000 to 462,000)	9,622.2 (8,813.9 to 10,391.8)	-10.7 (-17.3 to -2.9)	-37.9 (-42.2 to -32.9)	419,000 (383,000 to 461,000)	7,955.0 (7,329.7 to 8,616.0)	-10.3 (-17.2 to -3.3)	-40.3 (-44.5 to -36.3)
Somalia	788,000 (713,000 to 883,000)	10,835.5 (9,942.1 to 11,831.2)	99.0 (83.9 to 112.7)	-17.9 (-24.2 to -11.8)	743,000 (668,000 to 828,000)	9,171.3 (8,477.5 to 10,012.4)	111.3 (97.0 to 127.0)	-20.5 (-26.0 to -14.9)

	Male				Female			
	2019		Percent change from 1990 to 2019		2019		Percent change from 1990 to 2019	
	Number of episodes	Age standardized incidence (per 100,000)	Number of episodes	Age standardized incidence	Number of episodes	Age standardized incidence (per 100,000)	Number of episodes	Age standardized incidence
South Sudan	340,000 (307,000 to 377,000)	9,495.1 (8,730.1 to 10,333.2)	12.3 (5.1 to 19.9)	-20.2 (-25.4 to -13.9)	303,000 (274,000 to 335,000)	8,068.3 (7,438.6 to 8,743.6)	13.6 (5.4 to 22.1)	-25.0 (-30.0 to -19.5)
Uganda	1,270,000 (1,150,000 to 1,410,000)	9,183.5 (8,447.7 to 10,093.8)	55.8 (44.4 to 68.1)	-17.4 (-23.0 to -10.8)	1,210,000 (1,100,000 to 1,340,000)	7,375.7 (6,787.9 to 8,009.6)	59.3 (48.1 to 70.6)	-22.2 (-27.5 to -16.3)
United Republic of Tanzania	1,980,000 (1,800,000 to 2,180,000)	9,316.1 (8,607.8 to 10,097.6)	32.7 (22.7 to 42.4)	-32.1 (-37.3 to -26.7)	1,930,000 (1,750,000 to 2,140,000)	8,036.8 (7,343.0 to 8,785.3)	33.1 (24.3 to 43.8)	-32.8 (-37.2 to -27.3)
Zambia	477,000 (435,000 to 527,000)	7,730.2 (7,131.4 to 8,341.1)	26.1 (16.1 to 37.2)	-31.4 (-36.3 to -26.0)	427,000 (387,000 to 469,000)	6,178.3 (5,695.4 to 6,718.0)	15.8 (7.4 to 25.2)	-38.5 (-43.0 to -33.8)
Southern Sub-Saharan Africa	3,450,000 (3,220,000 to 3,700,000)	11,464.0 (10,765.1 to 12,247.7)	37.2 (32.0 to 42.2)	-9.0 (-12.1 to -6.1)	3,060,000 (2,860,000 to 3,270,000)	8,506.7 (7,934.0 to 9,099.0)	21.9 (17.1 to 26.7)	-16.3 (-18.7 to -13.7)
Botswana	80,600 (73,600 to 87,800)	9,286.5 (8,543.7 to 10,100.5)	44.5 (35.1 to 56.4)	-22.9 (-28.0 to -17.2)	75,000 (68,400 to 82,600)	7,420.7 (6,785.1 to 8,145.7)	57.6 (47.8 to 69.5)	-9.1 (-14.5 to -2.9)
Eswatini	45,700 (41,800 to 50,000)	10,897.0 (10,041.7 to 11,825.7)	19.5 (10.3 to 29.4)	-16.9 (-22.9 to -10.8)	38,000 (34,900 to 41,400)	7,641.8 (7,063.4 to 8,318.3)	0.2 (-6.9 to 8.0)	-25.6 (-30.6 to -20.3)
Lesotho	97,300 (88,900 to 107,000)	11,996.3 (11,021.1 to 13,010.9)	6.9 (-0.4 to 16.4)	-11.0 (-17.6 to -4.1)	83,900 (76,300 to 91,400)	8,899.8 (8,109.6 to 9,656.4)	-1.9 (-9.2 to 5.6)	-12.3 (-18.0 to -5.4)

	Male				Female			
	2019		Percent change from 1990 to 2019		2019		Percent change from 1990 to 2019	
	Number of episodes	Age standardized incidence (per 100,000)	Number of episodes	Age standardized incidence	Number of episodes	Age standardized incidence (per 100,000)	Number of episodes	Age standardized incidence
Namibia	107,000 (98,400 to 117,000)	12,180.6 (11,232.0 to 13,337.8)	22.6 (14.6 to 30.6)	-24.2 (-29.4 to -19.3)	89,000 (81,600 to 97,800)	8,285.5 (7,640.0 to 9,029.5)	9.2 (2.0 to 17.4)	-34.3 (-38.9 to -29.3)
South Africa	2,550,000 (2,390,000 to 2,730,000)	11,585.8 (10,819.3 to 12,428.3)	41.9 (35.3 to 48.1)	-8.3 (-12.0 to -4.6)	2,280,000 (2,130,000 to 2,440,000)	8,659.1 (8,072.1 to 9,262.2)	19.1 (13.5 to 24.7)	-19.3 (-22.3 to -16.3)
Zimbabwe	564,000 (518,000 to 615,000)	10,845.1 (10,035.7 to 11,728.7)	28.1 (19.0 to 37.0)	-9.6 (-16.1 to -2.5)	494,000 (448,000 to 539,000)	7,907.2 (7,265.6 to 8,550.7)	43.5 (33.7 to 54.6)	-3.5 (-10.0 to 3.8)
Western Sub-Saharan Africa	15,800,000 (14,600,000 to 17,000,000)	10,268.3 (9,599.8 to 10,984.0)	63.0 (58.2 to 68.0)	-16.3 (-18.0 to -14.5)	15,000,000 (13,800,000 to 16,300,000)	8,578.8 (8,032.5 to 9,187.5)	56.8 (52.0 to 62.5)	-22.3 (-23.9 to -20.5)
Benin	417,000 (380,000 to 453,000)	10,264.0 (9,419.6 to 11,119.4)	75.1 (63.3 to 89.0)	-19.2 (-24.7 to -12.8)	423,000 (386,000 to 461,000)	8,839.3 (8,111.7 to 9,625.1)	80.4 (65.9 to 94.2)	-17.8 (-24.1 to -11.8)
Burkina Faso	830,000 (755,000 to 910,000)	11,137.4 (10,218.0 to 12,188.6)	81.8 (70.4 to 94.9)	-12.2 (-18.4 to -5.3)	800,000 (733,000 to 879,000)	9,040.1 (8,300.5 to 9,856.1)	73.1 (61.0 to 86.5)	-16.3 (-22.5 to -10.6)
Cabo Verde	21,000 (19,200 to 22,800)	9,249.8 (8,467.9 to 10,085.5)	40.1 (29.4 to 52.2)	-15.5 (-22.0 to -8.4)	19,200 (17,700 to 20,900)	7,277.2 (6,697.0 to 7,939.5)	15.7 (6.9 to 24.9)	-21.6 (-27.4 to -15.5)
Cameroon	995,000 (910,000 to 1,080,000)	10,490.8 (9,577.6 to 11,363.5)	114.5 (99.9 to 129.8)	-11.0 (-17.3 to -4.1)	895,000 (818,000 to 976,000)	8,354.9 (7,686.3 to 9,042.3)	102.3 (87.2 to 118.4)	-15.6 (-21.7 to -9.3)

	Male				Female			
	2019		Percent change from 1990 to 2019		2019		Percent change from 1990 to 2019	
	Number of episodes	Age standardized incidence (per 100,000)	Number of episodes	Age standardized incidence	Number of episodes	Age standardized incidence (per 100,000)	Number of episodes	Age standardized incidence
Chad	745,000 (674,000 to 826,000)	12,875.3 (11,770.1 to 14,138.8)	116.9 (103.0 to 132.9)	-11.2 (-17.4 to -4.2)	716,000 (646,000 to 801,000)	11,457.3 (10,559.0 to 12,445.2)	107.3 (94.0 to 121.9)	-9.0 (-15.1 to -2.8)
Côte d'Ivoire	1,010,000 (914,000 to 1,090,000)	11,041.2 (10,060.9 to 12,032.1)	69.9 (58.0 to 82.8)	-16.4 (-22.6 to -9.4)	920,000 (839,000 to 1,010,000)	9,905.0 (9,105.7 to 10,787.7)	78.7 (66.4 to 92.9)	-8.6 (-14.8 to -1.9)
Gambia	84,700 (77,400 to 91,900)	11,477.7 (10,474.2 to 12,390.0)	47.9 (35.1 to 60.7)	-25.2 (-31.6 to -18.7)	79,300 (72,600 to 87,200)	9,468.9 (8,688.8 to 10,278.2)	53.4 (40.8 to 67.6)	-23.5 (-29.7 to -17.7)
Ghana	1,300,000 (1,180,000 to 1,400,000)	12,449.0 (11,354.3 to 13,588.4)	79.2 (66.2 to 94.7)	-8.6 (-15.2 to -1.2)	961,000 (882,000 to 1,040,000)	7,630.8 (7,018.5 to 8,251.4)	52.4 (40.4 to 66.6)	-25.7 (-31.1 to -19.5)
Guinea	586,000 (537,000 to 635,000)	13,499.7 (12,410.1 to 14,667.0)	54.8 (44.3 to 66.6)	-6.0 (-12.9 to 0.8)	558,000 (508,000 to 611,000)	11,323.9 (10,398.5 to 12,328.4)	46.1 (35.1 to 57.2)	-13.9 (-19.7 to -7.8)
Guinea-Bissau	66,900 (61,000 to 72,900)	11,596.8 (10,666.5 to 12,564.6)	26.0 (16.4 to 36.6)	-20.2 (-25.9 to -13.8)	71,900 (65,000 to 78,800)	10,049.1 (9,195.1 to 10,888.5)	34.2 (23.9 to 44.9)	-19.1 (-24.6 to -13.1)
Liberia	176,000 (161,000 to 193,000)	10,795.8 (9,869.6 to 11,822.0)	47.1 (35.4 to 59.9)	-22.8 (-28.8 to -16.3)	162,000 (147,000 to 177,000)	9,413.7 (8,645.8 to 10,240.4)	42.9 (31.3 to 55.1)	-24.7 (-30.4 to -19.1)
Mali	610,000 (557,000 to 667,000)	7,760.0 (7,151.8 to 8,399.4)	81.1 (69.6 to 95.1)	-18.7 (-24.8 to -12.4)	594,000 (536,000 to 658,000)	6,762.2 (6,247.3 to 7,318.5)	76.9 (65.4 to 89.6)	-19.5 (-24.8 to -13.7)

	Male				Female			
	2019		Percent change from 1990 to 2019		2019		Percent change from 1990 to 2019	
	**Number of episodes**	**Age standardized incidence (per 100,000)**	**Number of episodes**	**Age standardized incidence**	**Number of episodes**	**Age standardized incidence (per 100,000)**	**Number of episodes**	**Age standardized incidence**
Mauritania	150,000 (137,000 to 164,000)	10,270.7 (9,383.3 to 11,197.4)	51.7 (40.8 to 62.5)	-19.3 (-25.2 to -12.9)	136,000 (124,000 to 148,000)	8,696.9 (7,977.0 to 9,451.6)	42.1 (31.1 to 53.5)	-17.0 (-23.6 to -10.5)
Niger	933,000 (844,000 to 1,040,000)	12,313.7 (11,253.2 to 13,445.3)	85.0 (71.8 to 98.1)	-20.3 (-26.4 to -13.5)	948,000 (854,000 to 1,050,000)	10,899.8 (10,062.9 to 11,848.2)	91.3 (77.6 to 106.5)	-21.7 (-26.9 to -15.6)
Nigeria	6,630,000 (6,110,000 to 7,190,000)	9,167.8 (8,570.3 to 9,797.3)	48.1 (43.6 to 52.8)	-20.1 (-22.3 to -17.9)	6,530,000 (6,010,000 to 7,120,000)	7,982.4 (7,463.2 to 8,565.0)	41.9 (36.7 to 47.6)	-27.8 (-29.8 to -25.7)
Sao Tome and Principe	9,110 (8,330 to 9,960)	12,125.2 (11,037.7 to 13,220.2)	22.3 (11.5 to 34.4)	-19.6 (-25.8 to -12.4)	8,350 (7,640 to 9,130)	10,423.6 (9,591.3 to 11,289.9)	7.8 (-1.4 to 18.5)	-24.5 (-30.1 to -17.9)
Senegal	567,000 (521,000 to 617,000)	10,175.3 (9,360.5 to 11,026.3)	45.7 (34.6 to 58.5)	-19.1 (-25.3 to -12.6)	509,000 (468,000 to 551,000)	8,528.9 (7,890.1 to 9,192.1)	42.7 (30.2 to 55.0)	-17.7 (-24.0 to -12.1)
Sierra Leone	359,000 (327,000 to 392,000)	12,484.8 (11,477.1 to 13,645.2)	55.1 (44.5 to 67.5)	-17.9 (-23.8 to -10.8)	348,000 (317,000 to 382,000)	10,998.4 (10,109.7 to 12,026.1)	69.1 (56.7 to 81.9)	-10.7 (-16.6 to -4.1)
Togo	283,000 (258,000 to 309,000)	11,350.3 (10,375.3 to 12,364.9)	51.4 (38.8 to 63.8)	-20.4 (-26.9 to -14.0)	275,000 (251,000 to 300,000)	9,066.9 (8,335.8 to 9,934.2)	43.5 (31.6 to 55.6)	-26.6 (-31.8 to -21.0)

Appendix Table 2. Lower respiratory infection episodes (in thousands) and incidence rates (per 100,000 people) in 2019 and the percent change in episodes and incidence rates between 1990 and 2019 by age, sex, and GBD super-regions (95% UI)

		Male				Female			
		2019		Percent change from 1990 to 2019		2019		Percent change from 1990 to 2019	
	Age Group	Number of episodes (in thousands)	Incidence rate (per 100,000)	Number of episodes	Incidence rate	Number of episodes (in thousands)	Incidence rate (per 100,000)	Number of episodes	Incidence rate
Global	0-4 years	23,500 (18,600 to 29,500)	6,876.8 (5,444.3 to 8,625.5)	-49.1 (-51.1 to -47.4)	-51.7 (-53.5 to -50.0)	21,500 (17,100 to 26,800)	6,721.1 (5,337.5 to 8,363.0)	-49.9 (-51.6 to -48.5)	-52.1 (-53.7 to -50.7)
	5-14 years	33,000 (26,900 to 40,300)	4,931.0 (4,018.3 to 6,023.6)	-20.8 (-25.6 to -16.2)	-31.9 (-36.0 to -27.9)	26,600 (21,600 to 32,500)	4,235.4 (3,439.3 to 5,172.5)	-27.5 (-31.4 to -23.9)	-36.9 (-40.2 to -33.7)
	15-49 years	82,100 (74,200 to 91,200)	4,128.1 (3,726.8 to 4,583.5)	29.5 (26.8 to 31.9)	-10.6 (-12.4 to -8.9)	76,700 (68,900 to 86,000)	3,944.6 (3,541.5 to 4,421.4)	26.1 (23.2 to 29.1)	-13.3 (-15.3 to -11.2)
	50-69 years	65,800 (60,000 to 72,400)	9,737.2 (8,868.2 to 10,708.0)	66.8 (64.0 to 69.6)	-17.0 (-18.4 to -15.6)	55,500 (50,300 to 61,300)	7,894.7 (7,151.2 to 8,715.1)	67.6 (64.8 to 70.4)	-17.6 (-18.9 to -16.2)
	70+ years	52,500 (47,200 to 58,900)	25,786.6 (23,182.5 to 28,975.4)	126.0 (121.4 to 131.1)	-8.6 (-10.5 to -6.6)	51,600 (46,600 to 57,400)	19,819.9 (17,921.3 to 22,072.6)	93.7 (89.8 to 98.0)	-11.2 (-13.0 to -9.2)
Central Europe, Eastern Europe, and Central Asia	0-4 years	825 (654 to 1,030)	5,809.8 (4,602.4 to 7,284.2)	-57.6 (-60.6 to -54.6)	-45.7 (-49.7 to -42.0)	744 (587 to 934)	5,565.6 (4,395.4 to 6,990.1)	-57.1 (-60.0 to -54.5)	-43.9 (-47.6 to -40.5)
	5-14 years	1,220 (998 to 1,480)	4,371.5 (3,589.0 to 5,302.6)	-43.4 (-45.9 to -40.7)	-27.8 (-31.1 to -24.4)	1,090 (889 to 1,340)	4,171.7 (3,392.2 to 5,108.1)	-45.7 (-48.1 to -43.4)	-29.0 (-32.1 to -26.1)
	15-49 years	3,440 (3,160 to 3,720)	3,433.3 (3,155.2 to 3,713.0)	0.6 (-2.8 to 4.7)	2.9 (-0.6 to 7.1)	1,960 (1,780 to 2,150)	1,967.5 (1,787.7 to 2,157.2)	-10.8 (-14.0 to -7.4)	-8.3 (-11.5 to -4.8)

	50-69 years	4,760 (4,360 to 5,190)	10,646.2 (9,745.2 to 11,620.2)	0.8 (-3.8 to 4.7)	-17.9 (-21.7 to -14.7)	2,710 (2,470 to 2,980)	4,971.0 (4,527.6 to 5,459.1)	-3.8 (-6.9 to -0.5)	-17.0 (-19.7 to -14.2)
	70+ years	2,810 (2,510 to 3,130)	22,099.9 (19,745.1 to 24,630.1)	52.9 (46.7 to 59.0)	-9.2 (-12.9 to -5.5)	3,320 (2,980 to 3,690)	13,555.0 (12,158.2 to 15,056.5)	17.7 (12.3 to 22.5)	-15.2 (-19.1 to -11.7)
High-income	0-4 years	1,570 (1,220 to 2,020)	5,394.0 (4,175.9 to 6,938.8)	-27.7 (-30.7 to -24.6)	-21.9 (-25.1 to -18.6)	1,240 (959 to 1,600)	4,471.5 (3,453.0 to 5,773.3)	-26.1 (-29.0 to -23.3)	-20.5 (-23.6 to -17.4)
	5-14 years	1,480 (1,160 to 1,850)	2,378.1 (1,865.9 to 2,964.7)	-3.7 (-7.8 to 0.8)	-0.5 (-4.7 to 4.1)	1,370 (1,080 to 1,710)	2,298.8 (1,818.4 to 2,876.1)	-6.1 (-9.2 to -2.3)	-3.1 (-6.3 to 0.8)
	15-49 years	4,240 (3,790 to 4,760)	1,725.3 (1,544.2 to 1,938.9)	-10.9 (-13.5 to -7.7)	-14.1 (-16.6 to -11.0)	4,450 (3,930 to 5,040)	1,849.4 (1,632.7 to 2,093.6)	3.2 (0.5 to 6.4)	0.1 (-2.5 to 3.2)
	50-69 years	5,400 (4,950 to 5,880)	4,042.5 (3,706.4 to 4,409.2)	12.6 (8.3 to 16.6)	-29.7 (-32.4 to -27.3)	4,730 (4,300 to 5,200)	3,388.2 (3,078.3 to 3,723.6)	19.2 (15.1 to 23.2)	-21.4 (-24.1 to -18.8)
	70+ years	8,440 (7,660 to 9,250)	13,519.0 (12,278.1 to 14,820.7)	52.4 (46.5 to 58.6)	-30.2 (-33.0 to -27.4)	8,330 (7,610 to 9,060)	9,979.5 (9,115.8 to 10,859.0)	18.9 (14.7 to 23.7)	-32.3 (-34.7 to -29.6)
Latin America and Caribbean	0-4 years	1,840 (1,450 to 2,300)	7,515.0 (5,927.5 to 9,386.0)	-51.1 (-53.7 to -48.2)	-49.4 (-52.1 to -46.4)	1,800 (1,430 to 2,220)	7,630.2 (6,064.1 to 9,438.6)	-54.0 (-56.5 to -51.4)	-51.9 (-54.4 to -49.1)
	5-14 years	1,930 (1,570 to 2,360)	3,907.0 (3,175.2 to 4,770.8)	-34.6 (-38.7 to -30.3)	-36.8 (-40.8 to -32.6)	1,750 (1,420 to 2,140)	3,688.1 (2,986.5 to 4,511.9)	-37.6 (-41.7 to -33.8)	-38.5 (-42.5 to -34.7)
	15-49 years	5,540 (5,030 to 6,020)	3,653.9 (3,319.5 to 3,975.8)	23.9 (19.4 to 28.2)	-21.1 (-24.0 to -18.4)	4,210 (3,790 to 4,620)	2,688.9 (2,423.6 to 2,956.8)	16.8 (11.8 to 21.5)	-24.9 (-28.2 to -21.9)
	50-69 years	5,730 (5,180 to 6,310)	12,278.7 (11,096.7 to 13,509.5)	111.5 (103.8 to 118.4)	-16.3 (-19.3 to -13.6)	5,770 (5,250 to 6,320)	11,044.7 (10,057.8 to 12,099.3)	101.4 (95.5 to 107.6)	-24.4 (-26.6 to -22.1)
	70+ years	5,690 (5,120 to 6,290)	39,537.0 (35,616.5 to 43,707.7)	163.9 (153.9 to 173.1)	-7.6 (-11.1 to -4.4)	7,000 (6,350 to 7,780)	38,357.6 (34,786.1 to 42,667.0)	149.0 (141.3 to 156.5)	-19.7 (-22.2 to -17.2)

North Africa and Middle East	0-4 years	2,560 (2,010 to 3,180)	8,336.2 (6,540.8 to 10,366.6)	-48.7 (-51.7 to -46.0)	-54.4 (-57.0 to -51.9)	2,440 (1,910 to 3,030)	8,408.3 (6,590.1 to 10,434.3)	-54.3 (-57.0 to -51.9)	-59.1 (-61.4 to -56.8)
	5-14 years	3,040 (2,450 to 3,700)	5,083.2 (4,089.3 to 6,182.7)	-20.8 (-26.4 to -14.7)	-38.7 (-43.0 to -34.0)	2,970 (2,400 to 3,630)	5,272.5 (4,268.0 to 6,454.9)	-24.4 (-29.8 to -19.1)	-40.8 (-45.1 to -36.7)
	15-49 years	6,040 (5,390 to 6,750)	3,454.4 (3,084.5 to 3,857.6)	74.0 (66.3 to 81.0)	-17.1 (-20.7 to -13.8)	5,570 (4,960 to 6,250)	3,509.4 (3,122.5 to 3,933.6)	60.8 (53.5 to 68.2)	-19.9 (-23.5 to -16.2)
	50-69 years	3,530 (3,180 to 3,900)	8,584.6 (7,738.6 to 9,497.8)	110.0 (100.6 to 120.4)	-17.6 (-21.3 to -13.5)	2,980 (2,660 to 3,300)	7,730.3 (6,899.1 to 8,534.4)	105.6 (96.9 to 115.6)	-18.5 (-22.0 to -14.6)
	70+ years	2,680 (2,400 to 3,030)	27,496.0 (24,656.3 to 31,125.2)	146.6 (132.8 to 161.1)	-7.2 (-12.4 to -1.7)	2,390 (2,120 to 2,700)	24,401.5 (21,644.2 to 27,597.0)	125.6 (112.4 to 139.9)	-11.9 (-17.1 to -6.4)
South Asia	0-4 years	5,530 (4,340 to 6,880)	6,454.8 (5,066.7 to 8,032.7)	-50.7 (-53.3 to -48.1)	-51.8 (-54.4 to -49.3)	4,910 (3,880 to 6,090)	6,238.8 (4,934.7 to 7,737.2)	-50.1 (-52.1 to -48.3)	-50.6 (-52.6 to -48.8)
	5-14 years	13,600 (10,900 to 16,700)	7,402.1 (5,925.4 to 9,124.1)	-12.0 (-19.6 to -4.5)	-30.8 (-36.8 to -24.9)	8,150 (6,580 to 9,980)	4,821.9 (3,893.1 to 5,903.7)	-28.7 (-33.9 to -23.1)	-43.9 (-48.0 to -39.5)
	15-49 years	31,600 (28,200 to 35,300)	6,366.6 (5,681.3 to 7,111.8)	51.5 (45.7 to 57.0)	-16.3 (-19.5 to -13.3)	33,000 (29,400 to 37,000)	6,911.6 (6,157.3 to 7,756.1)	46.4 (40.6 to 51.8)	-21.8 (-24.9 to -19.0)
	50-69 years	24,600 (22,200 to 27,400)	20,043.3 (18,058.9 to 22,288.0)	86.0 (79.8 to 93.0)	-13.6 (-16.5 to -10.3)	23,600 (21,000 to 26,200)	19,278.2 (17,193.7 to 21,449.8)	95.7 (88.8 to 102.7)	-19.4 (-22.2 to -16.5)
	70+ years	15,900 (14,000 to 18,600)	48,185.3 (42,327.6 to 56,191.8)	185.4 (171.6 to 197.9)	-3.2 (-7.9 to 1.0)	14,000 (12,400 to 16,100)	38,852.6 (34,264.3 to 44,606.1)	223.1 (209.7 to 237.9)	-4.2 (-8.2 to 0.1)
Southeast Asia, East Asia, and Oceania	0-4 years	4,130 (3,240 to 5,200)	5,556.8 (4,360.8 to 7,004.4)	-71.1 (-73.0 to -69.1)	-63.4 (-65.8 to -60.8)	3,420 (2,660 to 4,300)	5,161.2 (4,022.2 to 6,498.3)	-73.5 (-75.3 to -71.8)	-65.6 (-67.8 to -63.3)
	5-14 years	5,270 (4,190 to 6,600)	3,746.3 (2,976.3 to 4,689.0)	-51.0 (-55.4 to -46.5)	-40.9 (-46.2 to -35.5)	4,560 (3,610 to 5,710)	3,641.9 (2,887.1 to 4,560.9)	-53.6 (-57.6 to -49.6)	-40.8 (-45.9 to -35.7)

	15-49 years	17,800 (15,800 to 20,100)	3,124.4 (2,776.8 to 3,528.9)	-7.3 (-11.0 to -3.5)	-22.7 (-25.8 to -19.5)	15,500 (13,600 to 17,800)	2,831.9 (2,486.8 to 3,249.1)	-9.3 (-14.0 to -4.4)	-24.4 (-28.3 to -20.3)
	50-69 years	15,500 (14,000 to 17,200)	6,286.4 (5,675.2 to 6,971.3)	77.3 (71.8 to 82.9)	-24.0 (-26.4 to -21.6)	10,700 (9,590 to 12,000)	4,269.8 (3,823.0 to 4,766.4)	45.1 (40.7 to 49.9)	-40.9 (-42.7 to -38.9)
	70+ years	12,900 (11,600 to 14,500)	20,702.3 (18,538.5 to 23,202.7)	165.2 (151.8 to 178.9)	-7.1 (-11.8 to -2.3)	12,500 (11,300 to 14,000)	16,156.8 (14,589.5 to 18,051.8)	89.8 (82.7 to 97.4)	-29.6 (-32.2 to -26.8)
Sub-Saharan Africa	0-4 years	7,090 (5,640 to 8,800)	8,452.1 (6,729.8 to 10,493.6)	-10.3 (-13.1 to -7.6)	-51.8 (-53.3 to -50.3)	6,990 (5,580 to 8,700)	8,540.7 (6,822.2 to 10,636.8)	-8.1 (-11.1 to -5.2)	-50.0 (-51.6 to -48.4)
	5-14 years	6,490 (5,290 to 7,860)	4,451.0 (3,626.5 to 5,391.4)	29.9 (23.7 to 35.9)	-40.1 (-42.9 to -37.3)	6,700 (5,420 to 8,160)	4,649.1 (3,765.5 to 5,668.5)	28.6 (23.1 to 34.9)	-39.8 (-42.4 to -36.8)
	15-49 years	13,500 (12,200 to 15,100)	5,353.5 (4,844.8 to 5,981.5)	85.2 (81.1 to 89.1)	-21.7 (-23.4 to -20.1)	12,100 (11,000 to 13,600)	4,535.2 (4,122.7 to 5,105.7)	57.1 (53.5 to 61.0)	-33.9 (-35.4 to -32.2)
	50-69 years	6,290 (5,650 to 6,980)	15,499.9 (13,928.8 to 17,200.7)	75.7 (72.2 to 80.1)	-15.6 (-17.3 to -13.5)	4,990 (4,490 to 5,520)	11,211.6 (10,094.9 to 12,403.1)	94.8 (90.7 to 99.5)	-17.1 (-18.9 to -15.1)
	70+ years	3,980 (3,540 to 4,530)	46,296.9 (41,168.9 to 52,655.8)	86.4 (81.5 to 91.8)	-6.6 (-9.1 to -3.9)	4,000 (3,580 to 4,530)	37,562.8 (33,598.4 to 42,583.6)	100.1 (94.9 to 105.4)	-6.1 (-8.5 to -3.6)

Appendix Table 3. Lower respiratory infection deaths and age standardized mortality rates (per 100, 000 people) among males and females in 2019 and the percent change in deaths and age standardized mortality rates between 1990 and 2019 for 21 GBD regions and 204 countries and territories (95% UI)

	Male				Female			
	2019		Percent change from 1990 to 2019		2019		Percent change from 1990 to 2019	
	Number of deaths	Age standardized mortality rate (per 100,000)	Number of deaths	Age standardized mortality rate	Number of deaths	Age standardized mortality rate (per 100,000)	Number of deaths	Age standardized mortality rate
**Global**	**1,300,000 (1,180,000 to 1,420,000)**	**39.7 (36.0 to 43.6)**	**-25.6 (-34.8 to -15.5)**	**-47.2 (-52.1 to -41.4)**	**1,200,000 (1,070,000 to 1,330,000)**	**30.5 (27.2 to 34.0)**	**-24.2 (-35.0 to -13.8)**	**-49.8 (-56.4 to -43.5)**
**Central Europe, Eastern Europe, and Central Asia**	**60,500 (54,700 to 66,600)**	**29.0 (26.1 to 32.0)**	**-9.5 (-19.6 to 0.1)**	**-29.1 (-37.0 to -21.5)**	**39,400 (35,500 to 43,300)**	**14.3 (12.8 to 16.0)**	**-26.2 (-33.5 to -18.8)**	**-47.5 (-53.4 to -40.5)**
Central Asia	13,900 (12,100 to 16,200)	37.7 (33.6 to 43.1)	-54.5 (-61.8 to -45.8)	-49.0 (-56.0 to -41.4)	10,500 (9,130 to 12,300)	25.9 (22.7 to 29.8)	-58.1 (-64.5 to -50.2)	-55.7 (-61.9 to -48.3)
Armenia	287 (245 to 331)	20.5 (17.3 to 23.5)	-52.7 (-60.9 to -43.1)	-44.3 (-53.7 to -33.9)	259 (217 to 302)	13.8 (11.6 to 16.1)	-52.0 (-61.4 to -41.4)	-56.5 (-64.3 to -45.9)
Azerbaijan	1,310 (1,010 to 1,650)	37.5 (29.8 to 46.3)	-71.4 (-78.4 to -62.5)	-64.5 (-72.6 to -54.9)	1,150 (920 to 1,420)	34.4 (27.6 to 42.9)	-71.6 (-78.1 to -63.9)	-63.6 (-71.9 to -52.8)
Georgia	413 (349 to 479)	19.3 (16.5 to 22.3)	-61.7 (-67.8 to -55.0)	-61.9 (-67.7 to -55.4)	353 (294 to 413)	9.8 (8.3 to 11.4)	-58.3 (-65.4 to -50.7)	-73.0 (-77.3 to -68.2)
Kazakhstan	2,470 (2,140 to 2,840)	35.3 (30.9 to 40.2)	-31.1 (-41.2 to -18.4)	-26.6 (-36.4 to -14.1)	1,550 (1,360 to 1,760)	17.3 (15.1 to 19.5)	-43.4 (-51.4 to -34.0)	-47.4 (-54.4 to -39.1)

	Male				Female			
	2019		Percent change from 1990 to 2019		2019		Percent change from 1990 to 2019	
	Number of deaths	Age standardized mortality rate (per 100,000)	Number of deaths	Age standardized mortality rate	Number of deaths	Age standardized mortality rate (per 100,000)	Number of deaths	Age standardized mortality rate
Kyrgyzstan	446 (381 to 501)	17.1 (14.9 to 19.0)	-75.6 (-79.6 to -71.4)	-75.1 (-78.7 to -71.4)	325 (274 to 370)	11.1 (9.6 to 12.5)	-78.3 (-82.1 to -74.0)	-79.1 (-82.4 to -75.1)
Mongolia	293 (220 to 386)	23.1 (18.6 to 28.6)	-83.1 (-87.9 to -74.0)	-81.8 (-86.1 to -73.3)	237 (181 to 312)	15.6 (12.1 to 20.1)	-82.9 (-87.5 to -75.7)	-83.6 (-87.7 to -76.6)
Tajikistan	1,900 (1,450 to 2,510)	53.5 (43.9 to 67.8)	-55.9 (-67.7 to -38.7)	-51.8 (-61.7 to -36.8)	1,380 (1,060 to 1,880)	38.5 (31.1 to 51.1)	-62.5 (-72.3 to -46.9)	-59.1 (-68.2 to -44.8)
Turkmenistan	749 (587 to 966)	31.5 (25.4 to 39.5)	-72.8 (-80.2 to -62.9)	-69.6 (-77.1 to -60.3)	534 (436 to 659)	21.5 (17.6 to 26.4)	-75.6 (-80.7 to -68.4)	-73.4 (-78.8 to -66.1)
Uzbekistan	6,050 (5,080 to 7,270)	46.3 (39.9 to 53.8)	-40.2 (-52.1 to -25.9)	-32.7 (-44.5 to -19.3)	4,740 (4,010 to 5,680)	35.0 (29.9 to 40.9)	-42.4 (-52.9 to -28.7)	-36.3 (-47.4 to -23.3)
Central Europe	16,900 (14,800 to 19,000)	21.6 (18.9 to 24.3)	-5.6 (-17.8 to 5.7)	-39.3 (-47.1 to -32.0)	13,800 (11,900 to 15,500)	11.2 (9.8 to 12.6)	-7.1 (-18.1 to 4.4)	-52.3 (-57.9 to -46.0)
Albania	255 (195 to 334)	17.1 (13.3 to 21.6)	-77.4 (-83.5 to -62.0)	-81.2 (-85.7 to -63.9)	202 (157 to 266)	11.8 (9.3 to 15.3)	-80.1 (-85.3 to -67.7)	-81.8 (-86.3 to -70.4)
Bosnia and Herzegovina	216 (168 to 276)	10.1 (8.0 to 12.7)	-10.5 (-31.9 to 18.9)	-43.6 (-56.2 to -22.8)	198 (151 to 311)	6.6 (5.0 to 10.0)	-17.5 (-38.7 to 34.5)	-52.0 (-63.7 to -22.2)

	Male				Female			
	2019		Percent change from 1990 to 2019		2019		Percent change from 1990 to 2019	
	Number of deaths	Age standardized mortality rate (per 100,000)	Number of deaths	Age standardized mortality rate	Number of deaths	Age standardized mortality rate (per 100,000)	Number of deaths	Age standardized mortality rate
Bulgaria	1,170<br>(938 to 1,430)	23.1<br>(18.8 to 28.2)	-52.3<br>(-61.7 to -41.1)	-60.3<br>(-68.0 to -51.4)	824<br>(662 to 1,010)	11.7<br>(9.3 to 14.3)	-51.4<br>(-60.7 to -40.9)	-68.0<br>(-74.2 to -60.7)
Croatia	309<br>(247 to 379)	9.5<br>(7.6 to 11.6)	-31.7<br>(-45.3 to -15.6)	-58.1<br>(-66.1 to -49.0)	270<br>(217 to 331)	4.7<br>(3.7 to 5.7)	-36.2<br>(-49.4 to -22.4)	-64.2<br>(-71.5 to -56.6)
Czechia	1,750<br>(1,450 to 2,100)	21.7<br>(18.0 to 25.8)	57.8<br>(31.0 to 89.8)	-12.4<br>(-27.0 to 4.4)	1,630<br>(1,330 to 1,960)	12.0<br>(9.9 to 14.5)	34.5<br>(12.3 to 59.6)	-22.6<br>(-35.2 to -7.1)
Hungary	596<br>(499 to 713)	9.0<br>(7.5 to 10.7)	-15.5<br>(-29.4 to 0.6)	-42.3<br>(-51.4 to -31.5)	620<br>(509 to 743)	5.0<br>(4.1 to 6.0)	4.2<br>(-12.2 to 23.3)	-45.2<br>(-53.6 to -34.7)
Montenegro	41<br>(33 to 50)	11.7<br>(9.5 to 14.4)	27.2<br>(-0.8 to 61.8)	-13.1<br>(-31.1 to 9.3)	28<br>(23 to 37)	6.0<br>(4.9 to 7.6)	12.0<br>(-11.4 to 43.0)	-30.5<br>(-45.5 to -13.3)
North Macedonia	99<br>(77 to 130)	10.0<br>(7.9 to 12.5)	-46.7<br>(-61.2 to -25.0)	-56.1<br>(-66.8 to -40.8)	82<br>(62 to 126)	7.3<br>(5.8 to 10.4)	-51.1<br>(-64.7 to -22.9)	-64.5<br>(-73.2 to -48.4)
Poland	6,480<br>(5,250 to 7,900)	25.2<br>(20.5 to 30.5)	77.5<br>(43.0 to 117.4)	2.1<br>(-16.9 to 23.5)	5,430<br>(4,310 to 6,640)	11.6<br>(9.4 to 14.2)	66.5<br>(36.5 to 100.2)	-18.7<br>(-33.2 to -1.7)
Romania	3,790<br>(3,150 to 4,500)	29.3<br>(24.9 to 34.5)	-30.9<br>(-43.9 to -17.9)	-48.4<br>(-57.0 to -38.8)	2,570<br>(2,150 to 3,040)	15.2<br>(12.9 to 17.7)	-36.5<br>(-47.4 to -23.9)	-62.6<br>(-68.4 to -55.3)

	Male				Female			
	2019		Percent change from 1990 to 2019		2019		Percent change from 1990 to 2019	
	Number of deaths	Age standardized mortality rate (per 100,000)	Number of deaths	Age standardized mortality rate	Number of deaths	Age standardized mortality rate (per 100,000)	Number of deaths	Age standardized mortality rate
Serbia	892 (688 to 1,110)	14.5 (11.4 to 17.7)	27.1 (-9.0 to 66.3)	-20.0 (-41.8 to 4.7)	680 (531 to 852)	9.0 (7.0 to 11.0)	12.7 (-14.7 to 44.9)	-33.3 (-49.3 to -13.4)
Slovakia	968 (750 to 1,210)	30.4 (23.9 to 37.6)	-35.5 (-49.9 to -16.0)	-55.2 (-64.8 to -41.9)	883 (680 to 1,130)	16.2 (12.6 to 20.7)	-31.3 (-48.9 to 6.1)	-59.3 (-69.6 to -38.0)
Slovenia	326 (259 to 413)	19.5 (15.7 to 24.4)	31.4 (-2.8 to 76.5)	-38.2 (-53.5 to -19.2)	368 (275 to 462)	9.8 (7.5 to 12.3)	33.6 (-3.6 to 76.8)	-49.7 (-62.8 to -33.7)
Eastern Europe	29,700 (25,600 to 34,100)	25.0 (21.7 to 28.5)	61.1 (39.0 to 85.2)	15.9 (0.9 to 32.1)	15,100 (13,100 to 17,200)	8.6 (7.5 to 9.8)	12.5 (-1.5 to 28.4)	-23.1 (-32.8 to -12.3)
Belarus	709 (559 to 893)	13.4 (10.7 to 16.6)	1.9 (-19.3 to 28.8)	-22.8 (-37.8 to -3.4)	446 (360 to 553)	4.4 (3.6 to 5.5)	-26.0 (-40.3 to -7.7)	-55.6 (-64.3 to -44.1)
Estonia	126 (99 to 157)	14.6 (11.6 to 17.9)	-2.3 (-24.5 to 21.9)	-25.2 (-41.3 to -7.3)	97 (77 to 119)	5.6 (4.6 to 6.8)	8.6 (-12.8 to 33.4)	-34.9 (-47.5 to -20.1)
Latvia	196 (156 to 243)	15.9 (12.8 to 19.6)	-11.5 (-30.5 to 10.5)	-15.3 (-32.7 to 4.8)	155 (122 to 197)	6.5 (5.1 to 8.4)	-4.4 (-24.0 to 22.4)	-24.8 (-40.7 to -2.3)
Lithuania	312 (254 to 378)	16.6 (13.7 to 19.9)	48.6 (20.6 to 81.7)	26.1 (3.5 to 52.5)	219 (179 to 259)	6.2 (5.2 to 7.4)	32.0 (9.3 to 56.3)	-9.3 (-24.7 to 7.7)

	Male				Female			
	2019		Percent change from 1990 to 2019		2019		Percent change from 1990 to 2019	
	Number of deaths	Age standardized mortality rate (per 100,000)	Number of deaths	Age standardized mortality rate	Number of deaths	Age standardized mortality rate (per 100,000)	Number of deaths	Age standardized mortality rate
Republic of Moldova	619 (536 to 705)	30.0 (26.2 to 33.8)	-14.9 (-28.7 to 0.8)	-23.7 (-34.9 to -10.6)	253 (218 to 287)	10.7 (9.3 to 12.1)	-48.0 (-56.5 to -38.7)	-55.6 (-62.6 to -47.3)
Russian Federation	21,800 (18,100 to 25,900)	26.5 (22.2 to 31.2)	65.4 (36.7 to 97.5)	12.2 (-6.5 to 32.4)	11,200 (9,370 to 13,100)	9.3 (7.8 to 10.8)	20.9 (2.4 to 41.7)	-22.9 (-34.7 to -9.4)
Ukraine	6,010 (4,830 to 7,520)	24.0 (19.6 to 29.7)	81.3 (44.7 to 129.4)	49.0 (20.9 to 86.7)	2,720 (2,210 to 3,310)	7.5 (6.1 to 9.1)	2.7 (-16.6 to 25.9)	-7.9 (-25.3 to 13.8)
**High-income**	**218,000 (194,000 to 231,000)**	**22.4 (19.9 to 23.8)**	**53.2 (42.9 to 59.4)**	**-35.3 (-38.6 to -33.1)**	**218,000 (177,000 to 240,000)**	**13.2 (11.1 to 14.4)**	**47.1 (33.5 to 55.1)**	**-34.1 (-38.2 to -31.3)**
Australasia	2,290 (1,970 to 2,530)	10.0 (8.7 to 11.0)	74.8 (57.3 to 91.9)	-43.1 (-48.1 to -38.0)	2,880 (2,260 to 3,280)	8.0 (6.4 to 9.0)	90.5 (68.7 to 109.2)	-31.8 (-38.8 to -25.5)
Australia	1,900 (1,640 to 2,110)	9.9 (8.5 to 10.9)	115.3 (91.1 to 140.2)	-29.1 (-36.1 to -21.6)	2,370 (1,850 to 2,720)	7.9 (6.3 to 8.9)	151.9 (120.5 to 182.3)	-10.9 (-20.9 to -1.5)
New Zealand	391 (338 to 444)	11.0 (9.5 to 12.4)	-8.6 (-20.4 to 4.2)	-68.5 (-72.2 to -64.3)	505 (392 to 591)	8.9 (7.1 to 10.3)	-11.2 (-23.4 to 1.8)	-66.0 (-70.2 to -61.6)
High-income Asia Pacific	78,700 (66,900 to 85,400)	35.3 (30.2 to 38.4)	101.6 (79.8 to 114.6)	-44.7 (-49.6 to -41.6)	59,300 (42,700 to 69,300)	13.9 (10.5 to 15.9)	99.6 (60.9 to 122.2)	-53.7 (-60.9 to -49.6)

	Male				Female			
	2019		Percent change from 1990 to 2019		2019		Percent change from 1990 to 2019	
	Number of deaths	Age standardized mortality rate (per 100,000)	Number of deaths	Age standardized mortality rate	Number of deaths	Age standardized mortality rate (per 100,000)	Number of deaths	Age standardized mortality rate
Brunei Darussalam	46 (38 to 56)	85.7 (71.6 to 100.5)	130.9 (63.1 to 190.4)	14.1 (-21.1 to 44.1)	35 (27 to 41)	40.1 (31.7 to 46.4)	186.3 (83.0 to 260.9)	26.4 (-23.2 to 62.5)
Japan	68,700 (57,600 to 74,900)	36.0 (30.5 to 39.1)	90.8 (69.5 to 103.9)	-45.2 (-50.0 to -42.1)	50,100 (35,600 to 59,000)	13.5 (10.1 to 15.5)	87.2 (49.8 to 108.7)	-55.5 (-61.9 to -51.6)
Republic of Korea	8,060 (4,480 to 9,290)	29.3 (16.6 to 33.9)	244.9 (59.6 to 306.9)	-7.0 (-61.5 to 10.6)	7,570 (3,960 to 9,280)	14.7 (7.8 to 18.0)	226.4 (67.8 to 305.2)	-23.5 (-60.9 to -5.2)
Singapore	1,840 (1,610 to 2,000)	65.1 (55.9 to 71.3)	178.8 (150.1 to 204.6)	-34.3 (-40.4 to -28.2)	1,640 (1,290 to 1,860)	39.5 (31.3 to 44.6)	158.3 (120.4 to 186.5)	-41.2 (-48.4 to -35.2)
High-income North America	43,100 (39,200 to 45,600)	15.6 (14.2 to 16.4)	10.7 (6.4 to 15.2)	-47.4 (-49.1 to -45.5)	48,000 (40,600 to 52,200)	11.5 (10.0 to 12.3)	18.7 (12.9 to 24.6)	-33.7 (-36.0 to -30.7)
Canada	4,090 (3,530 to 4,540)	13.4 (11.6 to 14.8)	25.9 (12.5 to 39.4)	-54.5 (-58.8 to -49.9)	5,040 (4,030 to 5,730)	10.3 (8.5 to 11.6)	51.4 (34.7 to 68.8)	-40.9 (-46.5 to -34.7)
Greenland	7 (6 to 9)	31.1 (24.2 to 36.8)	9.8 (-17.6 to 38.5)	-44.9 (-56.8 to -32.4)	5 (4 to 6)	20.4 (15.7 to 25.0)	-14.1 (-34.2 to 10.6)	-53.5 (-64.3 to -40.1)
United States of America	39,000 (35,500 to 41,200)	15.9 (14.5 to 16.7)	9.4 (5.1 to 13.8)	-46.6 (-48.4 to -44.7)	42,900 (36,400 to 46,600)	11.6 (10.2 to 12.4)	15.8 (10.2 to 21.7)	-33.0 (-35.4 to -30.0)

	Male				Female			
	2019		Percent change from 1990 to 2019		2019		Percent change from 1990 to 2019	
	Number of deaths	Age standardized mortality rate (per 100,000)	Number of deaths	Age standardized mortality rate	Number of deaths	Age standardized mortality rate (per 100,000)	Number of deaths	Age standardized mortality rate
Southern Latin America	20,200 (18,500 to 21,700)	60.5 (55.4 to 65.1)	135.3 (116.9 to 153.6)	24.5 (15.5 to 33.4)	22,400 (19,500 to 24,500)	42.6 (37.4 to 46.6)	205.9 (176.7 to 233.0)	38.2 (27.4 to 49.7)
Argentina	17,200 (15,800 to 18,700)	80.9 (73.6 to 87.5)	264.8 (233.8 to 299.1)	108.3 (90.8 to 126.4)	19,000 (16,500 to 20,900)	55.1 (48.4 to 60.6)	380.6 (334.4 to 429.9)	133.0 (111.4 to 156.2)
Chile	2,260 (1,990 to 2,480)	23.7 (20.7 to 26.0)	-34.3 (-41.1 to -27.6)	-72.7 (-75.3 to -69.9)	2,440 (2,040 to 2,720)	17.8 (14.9 to 19.9)	-17.1 (-27.8 to -8.2)	-73.0 (-76.0 to -70.3)
Uruguay	671 (600 to 739)	30.0 (26.8 to 32.9)	66.7 (49.5 to 84.4)	5.3 (-4.9 to 16.5)	924 (773 to 1,030)	21.9 (18.8 to 24.2)	121.2 (96.1 to 145.6)	10.9 (-0.1 to 22.3)
Western Europe	73,700 (66,100 to 78,500)	17.4 (15.6 to 18.5)	35.3 (27.3 to 41.9)	-39.2 (-42.0 to -36.6)	85,400 (70,600 to 93,500)	11.3 (9.6 to 12.3)	23.4 (13.9 to 30.2)	-38.8 (-42.3 to -35.9)
Andorra	9 (7 to 11)	13.2 (10.3 to 16.2)	183.4 (97.2 to 300.8)	-30.7 (-49.9 to -4.4)	11 (8 to 15)	11.5 (8.2 to 15.2)	338.9 (189.4 to 526.0)	-24.8 (-48.3 to 6.0)
Austria	581 (510 to 642)	7.4 (6.4 to 8.1)	-4.3 (-14.6 to 6.4)	-54.6 (-59.2 to -49.8)	624 (511 to 707)	4.4 (3.7 to 4.9)	-18.6 (-28.3 to -8.9)	-54.6 (-59.2 to -50.0)
Belgium	2,920 (2,550 to 3,210)	27.3 (23.9 to 30.0)	71.8 (54.2 to 89.9)	-19.2 (-26.7 to -11.3)	3,420 (2,710 to 3,870)	17.4 (14.2 to 19.5)	94.3 (71.0 to 117.4)	0.3 (-9.6 to 11.0)

	Male				Female			
	2019		Percent change from 1990 to 2019		2019		Percent change from 1990 to 2019	
	Number of deaths	Age standardized mortality rate (per 100,000)	Number of deaths	Age standardized mortality rate	Number of deaths	Age standardized mortality rate (per 100,000)	Number of deaths	Age standardized mortality rate
Cyprus	76 (63 to 92)	11.6 (9.6 to 13.9)	24.9 (-2.7 to 63.1)	-53.6 (-62.9 to -40.8)	93 (76 to 122)	11.2 (9.1 to 14.7)	16.7 (-11.5 to 66.4)	-65.5 (-72.7 to -53.0)
Denmark	1,160 (1,010 to 1,290)	23.5 (20.3 to 26.1)	41.9 (25.8 to 58.5)	-14.8 (-24.3 to -5.1)	1,310 (1,050 to 1,500)	15.4 (12.6 to 17.5)	28.8 (12.9 to 45.5)	-16.6 (-26.5 to -6.2)
Finland	375 (333 to 419)	7.0 (6.2 to 7.8)	-66.5 (-70.1 to -62.5)	-87.3 (-88.6 to -85.9)	400 (323 to 457)	4.0 (3.4 to 4.6)	-72.6 (-75.9 to -69.2)	-86.9 (-88.3 to -85.4)
France	10,200 (8,700 to 11,500)	15.4 (13.2 to 17.3)	26.6 (11.6 to 41.8)	-46.5 (-52.0 to -40.5)	12,900 (9,770 to 15,000)	9.4 (7.3 to 10.7)	34.2 (13.3 to 51.4)	-40.6 (-48.1 to -34.0)
Germany	12,800 (11,400 to 14,300)	14.8 (13.2 to 16.4)	47.7 (32.1 to 64.3)	-31.0 (-37.5 to -23.9)	12,200 (10,300 to 13,700)	8.5 (7.2 to 9.4)	17.9 (6.4 to 31.3)	-27.9 (-33.9 to -20.3)
Greece	2,740 (2,360 to 3,060)	20.5 (17.9 to 22.6)	184.4 (150.9 to 222.2)	24.8 (11.9 to 39.9)	3,220 (2,690 to 3,640)	17.6 (15.0 to 19.6)	246.1 (201.1 to 290.7)	45.3 (29.3 to 61.3)
Iceland	48 (41 to 55)	17.8 (15.1 to 20.3)	21.0 (4.3 to 39.8)	-44.9 (-52.2 to -36.5)	54 (40 to 65)	12.0 (9.3 to 14.1)	-12.5 (-27.0 to 0.7)	-63.7 (-69.0 to -58.5)
Ireland	735 (638 to 830)	24.3 (20.9 to 27.3)	-13.6 (-24.2 to -2.9)	-60.9 (-65.4 to -56.1)	978 (777 to 1,110)	20.1 (16.2 to 22.8)	-3.5 (-16.8 to 8.4)	-56.3 (-61.6 to -51.1)

	Male				Female			
	2019		Percent change from 1990 to 2019		2019		Percent change from 1990 to 2019	
	Number of deaths	Age standardized mortality rate (per 100,000)	Number of deaths	Age standardized mortality rate	Number of deaths	Age standardized mortality rate (per 100,000)	Number of deaths	Age standardized mortality rate
Israel	839 (732 to 930)	15.6 (13.6 to 17.3)	134.7 (109.5 to 162.7)	-15.5 (-24.3 to -5.9)	900 (746 to 1,020)	11.6 (9.7 to 13.1)	147.5 (114.0 to 177.3)	-28.9 (-37.3 to -21.2)
Italy	5,850 (5,100 to 6,300)	8.7 (7.6 to 9.4)	40.3 (27.7 to 49.3)	-40.4 (-44.3 to -37.1)	6,540 (5,270 to 7,270)	5.2 (4.3 to 5.7)	46.7 (28.7 to 57.7)	-43.8 (-48.8 to -40.4)
Luxembourg	61 (51 to 70)	14.3 (11.9 to 16.5)	43.7 (20.3 to 69.8)	-43.8 (-52.7 to -34.0)	76 (58 to 92)	9.8 (7.7 to 11.7)	64.4 (32.6 to 97.3)	-32.1 (-43.9 to -19.0)
Malta	88 (76 to 101)	23.1 (19.9 to 26.5)	86.5 (58.8 to 115.6)	-30.9 (-40.4 to -20.3)	103 (80 to 121)	15.9 (12.7 to 18.6)	125.5 (88.0 to 162.2)	-23.5 (-34.8 to -10.9)
Monaco	12 (9 to 14)	23.9 (19.2 to 29.0)	74.2 (21.2 to 129.2)	-1.2 (-29.7 to 27.4)	13 (9 to 16)	16.3 (12.1 to 20.3)	61.6 (13.6 to 119.5)	7.0 (-24.5 to 42.7)
Netherlands	2,910 (2,520 to 3,250)	20.7 (17.9 to 23.2)	70.0 (52.0 to 88.6)	-21.3 (-29.0 to -12.7)	3,420 (2,800 to 3,900)	13.8 (11.5 to 15.6)	51.7 (35.3 to 67.7)	-18.7 (-26.9 to -10.7)
Norway	909 (798 to 987)	20.5 (18.0 to 22.3)	-29.1 (-33.7 to -24.5)	-58.2 (-60.6 to -55.6)	1,130 (900 to 1,260)	14.0 (11.4 to 15.5)	-33.6 (-38.9 to -28.7)	-58.8 (-61.6 to -55.9)
Portugal	4,090 (3,560 to 4,500)	37.5 (32.7 to 41.2)	133.4 (104.4 to 159.1)	-4.4 (-15.0 to 5.3)	4,180 (3,380 to 4,720)	21.2 (17.5 to 23.7)	166.5 (128.3 to 196.6)	-5.7 (-17.2 to 4.1)

	Male				Female			
	2019		Percent change from 1990 to 2019		2019		Percent change from 1990 to 2019	
	Number of deaths	Age standardized mortality rate (per 100,000)	Number of deaths	Age standardized mortality rate	Number of deaths	Age standardized mortality rate (per 100,000)	Number of deaths	Age standardized mortality rate
San Marino	4 (3 to 6)	13.2 (9.2 to 17.6)	108.1 (39.9 to 183.6)	-27.7 (-50.7 to -0.6)	3 (2 to 5)	6.3 (4.3 to 9.0)	122.0 (49.1 to 223.6)	-23.4 (-48.9 to 12.8)
Spain	6,670 (5,780 to 7,460)	14.5 (12.7 to 16.1)	47.9 (31.3 to 65.3)	-40.0 (-46.4 to -33.5)	7,510 (5,870 to 8,720)	8.7 (7.1 to 10.0)	66.8 (45.1 to 87.8)	-40.3 (-46.9 to -33.8)
Sweden	1,410 (1,220 to 1,560)	13.3 (11.6 to 14.7)	-32.6 (-39.9 to -24.9)	-61.6 (-65.3 to -57.3)	1,440 (1,150 to 1,630)	8.5 (7.0 to 9.6)	-34.2 (-41.4 to -26.6)	-58.1 (-62.3 to -53.8)
Switzerland	869 (745 to 974)	10.4 (8.9 to 11.6)	-22.5 (-31.5 to -13.7)	-65.7 (-69.3 to -62.0)	1,080 (834 to 1,260)	7.1 (5.7 to 8.1)	-26.1 (-35.1 to -16.7)	-64.0 (-67.9 to -60.0)
United Kingdom	18,200 (16,400 to 19,200)	31.6 (28.4 to 33.4)	26.6 (19.7 to 32.5)	-37.7 (-40.2 to -35.1)	23,700 (20,000 to 25,700)	25.4 (21.9 to 27.4)	0.9 (-5.3 to 8.0)	-34.2 (-37.3 to -29.8)
**Latin America and Caribbean**	**97,700 (86,400 to 108,000)**	**39.7 (35.0 to 44.1)**	**-0.1 (-12.8 to 12.2)**	**-40.7 (-46.9 to -33.9)**	**93,700 (80,800 to 104,000)**	**30.8 (26.5 to 34.2)**	**11.2 (-5.2 to 25.6)**	**-45.0 (-51.4 to -38.7)**
Andean Latin America	15,200 (12,100 to 18,900)	59.3 (47.2 to 73.0)	-16.5 (-36.9 to 6.3)	-47.7 (-58.8 to -35.4)	15,000 (12,000 to 18,200)	52.1 (41.7 to 63.4)	-12.7 (-31.6 to 6.7)	-51.9 (-60.9 to -42.1)
Bolivia (Plurinational State of)	3,200 (2,510 to 3,920)	81.6 (63.4 to 101.1)	-41.2 (-58.6 to -17.3)	-52.2 (-64.7 to -37.9)	3,370 (2,730 to 4,310)	79.4 (63.7 to 101.8)	-29.8 (-47.9 to -7.7)	-47.9 (-59.7 to -34.3)

	Male				Female			
	2019		Percent change from 1990 to 2019		2019		Percent change from 1990 to 2019	
	Number of deaths	Age standardized mortality rate (per 100,000)	Number of deaths	Age standardized mortality rate	Number of deaths	Age standardized mortality rate (per 100,000)	Number of deaths	Age standardized mortality rate
Ecuador	2,810 (2,260 to 3,550)	45.5 (37.1 to 57.0)	22.2 (-2.7 to 55.0)	-28.2 (-41.7 to -10.4)	2,630 (2,160 to 3,370)	38.5 (31.7 to 49.1)	15.8 (-6.6 to 48.9)	-46.0 (-55.7 to -30.5)
Peru	9,200 (6,930 to 12,000)	59.4 (44.6 to 77.8)	-12.2 (-37.1 to 17.4)	-49.6 (-63.0 to -33.3)	8,980 (6,640 to 11,500)	51.0 (37.8 to 65.5)	-11.0 (-33.8 to 16.6)	-53.9 (-65.1 to -39.9)
Caribbean	9,920 (8,470 to 11,500)	44.4 (37.8 to 51.8)	6.9 (-11.3 to 28.1)	-28.4 (-39.4 to -15.6)	9,260 (7,800 to 10,800)	34.8 (29.0 to 40.9)	11.3 (-7.6 to 34.0)	-33.8 (-44.6 to -21.4)
Antigua and Barbuda	14 (12 to 17)	40.5 (34.3 to 47.9)	35.8 (12.9 to 63.7)	-19.1 (-32.0 to -3.5)	16 (13 to 18)	33.8 (28.4 to 39.0)	39.5 (16.7 to 66.6)	15.4 (-3.3 to 37.1)
Bahamas	50 (42 to 60)	36.6 (30.3 to 43.6)	46.5 (18.9 to 81.9)	-36.7 (-47.9 to -22.6)	43 (36 to 52)	23.5 (19.3 to 27.9)	52.5 (25.8 to 85.1)	-29.1 (-41.7 to -13.9)
Barbados	77 (63 to 91)	39.3 (32.5 to 46.2)	82.7 (48.0 to 119.5)	5.1 (-14.6 to 24.7)	88 (72 to 103)	32.3 (26.5 to 38.0)	94.6 (60.5 to 130.6)	27.3 (5.3 to 50.2)
Belize	63 (54 to 73)	49.4 (41.8 to 57.4)	82.6 (52.3 to 118.1)	-7.8 (-22.5 to 10.1)	49 (41 to 56)	37.5 (31.6 to 43.4)	37.3 (15.9 to 63.2)	-28.6 (-39.1 to -16.5)
Bermuda	8 (7 to 10)	16.2 (13.7 to 19.3)	47.5 (22.2 to 80.8)	-41.1 (-51.0 to -28.3)	9 (7 to 12)	10.7 (8.4 to 13.3)	42.0 (13.6 to 75.2)	-48.9 (-58.4 to -36.4)

	Male				Female			
	2019		Percent change from 1990 to 2019		2019		Percent change from 1990 to 2019	
	Number of deaths	Age standardized mortality rate (per 100,000)	Number of deaths	Age standardized mortality rate	Number of deaths	Age standardized mortality rate (per 100,000)	Number of deaths	Age standardized mortality rate
Cuba	3,710 (3,040 to 4,450)	41.5 (34.0 to 49.6)	103.2 (67.5 to 141.5)	4.6 (-13.3 to 24.2)	3,410 (2,750 to 4,120)	29.7 (24.1 to 35.7)	134.6 (95.1 to 175.8)	-2.8 (-19.1 to 14.2)
Dominica	16 (13 to 20)	43.8 (34.9 to 53.9)	16.9 (-8.3 to 46.1)	-9.3 (-28.4 to 12.9)	14 (11 to 17)	29.0 (23.0 to 36.0)	-1.0 (-21.2 to 25.9)	-7.7 (-27.0 to 16.8)
Dominican Republic	1,070 (817 to 1,390)	24.7 (19.1 to 32.0)	-3.1 (-29.8 to 38.0)	-24.3 (-42.9 to 1.8)	945 (731 to 1,220)	20.4 (15.8 to 26.4)	-12.2 (-36.4 to 20.7)	-37.9 (-53.1 to -19.5)
Grenada	20 (18 to 23)	58.0 (51.3 to 65.4)	-24.0 (-35.1 to -11.0)	-28.7 (-38.5 to -17.1)	27 (23 to 30)	49.9 (42.5 to 56.4)	-15.0 (-28.9 to -1.3)	-16.6 (-30.2 to -2.9)
Guyana	143 (111 to 179)	59.8 (47.7 to 72.8)	-8.8 (-31.8 to 19.6)	-22.8 (-39.3 to -1.9)	119 (92 to 148)	44.0 (34.6 to 54.0)	2.0 (-23.0 to 30.8)	-20.2 (-39.4 to 0.8)
Haiti	3,260 (2,320 to 4,310)	78.5 (52.4 to 107.5)	-27.2 (-51.1 to 1.8)	-43.8 (-57.9 to -27.3)	3,060 (2,220 to 4,060)	73.2 (52.2 to 99.5)	-26.5 (-48.1 to 1.5)	-42.5 (-57.0 to -24.7)
Jamaica	225 (180 to 277)	16.1 (12.8 to 19.9)	-3.4 (-21.8 to 17.7)	-38.3 (-50.4 to -24.2)	250 (195 to 304)	12.9 (10.2 to 15.9)	2.8 (-16.1 to 23.6)	-40.6 (-51.4 to -27.7)
Puerto Rico	612 (483 to 763)	19.2 (15.1 to 24.0)	-13.1 (-30.4 to 7.1)	-58.8 (-66.9 to -48.9)	655 (507 to 805)	13.6 (10.6 to 16.8)	16.0 (-6.1 to 42.5)	-56.5 (-65.1 to -46.5)

	Male				Female			
	2019		Percent change from 1990 to 2019		2019		Percent change from 1990 to 2019	
	Number of deaths	Age standardized mortality rate (per 100,000)	Number of deaths	Age standardized mortality rate	Number of deaths	Age standardized mortality rate (per 100,000)	Number of deaths	Age standardized mortality rate
Saint Kitts and Nevis	10 (9 to 12)	49.6 (43.1 to 56.4)	-2.7 (-21.5 to 19.1)	-36.3 (-46.1 to -24.6)	9 (8 to 11)	34.8 (29.1 to 40.8)	-20.6 (-34.8 to -3.3)	-43.2 (-52.8 to -31.8)
Saint Lucia	28 (24 to 33)	35.5 (29.8 to 41.5)	60.6 (33.9 to 92.1)	-33.4 (-44.2 to -20.6)	26 (21 to 31)	23.8 (19.5 to 28.2)	43.7 (19.8 to 70.2)	-37.0 (-47.4 to -25.1)
Saint Vincent and the Grenadines	21 (18 to 25)	39.5 (34.3 to 45.1)	50.0 (25.6 to 78.8)	-19.0 (-31.5 to -4.0)	18 (15 to 21)	30.3 (25.8 to 35.3)	15.9 (-0.9 to 36.4)	-18.2 (-30.1 to -3.7)
Suriname	90 (72 to 110)	38.8 (31.6 to 47.5)	49.0 (17.7 to 86.9)	-18.1 (-34.2 to 1.9)	74 (59 to 90)	24.7 (19.7 to 30.2)	50.6 (21.7 to 85.7)	-21.9 (-36.2 to -4.8)
Trinidad and Tobago	149 (114 to 190)	20.8 (15.9 to 26.3)	-19.8 (-38.7 to 2.5)	-63.2 (-71.6 to -52.8)	125 (95 to 158)	13.8 (10.5 to 17.6)	-18.5 (-37.8 to 3.4)	-63.3 (-71.7 to -52.9)
United States Virgin Islands	14 (12 to 17)	22.5 (18.7 to 26.4)	100.7 (57.2 to 159.2)	-7.7 (-26.7 to 17.4)	11 (9 to 13)	12.3 (9.8 to 14.8)	83.9 (42.4 to 132.4)	-24.3 (-41.2 to -5.0)
Central Latin America	28,000 (23,700 to 33,100)	27.0 (22.9 to 31.6)	-12.3 (-26.9 to 4.4)	-47.0 (-54.7 to -37.5)	23,800 (20,000 to 27,800)	19.6 (16.5 to 23.0)	-15.8 (-30.7 to 0.7)	-56.3 (-63.7 to -48.6)
Colombia	3,940 (3,020 to 5,020)	16.7 (12.7 to 21.3)	-4.8 (-28.6 to 24.4)	-49.7 (-61.4 to -35.4)	3,960 (3,020 to 4,950)	13.7 (10.5 to 17.4)	9.3 (-16.5 to 38.8)	-55.1 (-65.8 to -42.9)

	Male				Female			
	2019		Percent change from 1990 to 2019		2019		Percent change from 1990 to 2019	
	Number of deaths	Age standardized mortality rate (per 100,000)	Number of deaths	Age standardized mortality rate	Number of deaths	Age standardized mortality rate (per 100,000)	Number of deaths	Age standardized mortality rate
Costa Rica	392 (304 to 492)	17.2 (13.4 to 21.7)	65.7 (28.3 to 113.7)	-28.0 (-44.1 to -7.3)	284 (214 to 354)	9.8 (7.4 to 12.2)	44.8 (14.3 to 83.9)	-49.9 (-60.9 to -36.6)
El Salvador	1,190 (893 to 1,500)	44.4 (33.3 to 56.0)	23.2 (-12.9 to 62.2)	-8.9 (-34.2 to 17.8)	1,110 (811 to 1,430)	29.0 (21.2 to 37.6)	18.6 (-14.3 to 55.1)	-31.8 (-49.3 to -11.6)
Guatemala	5,250 (4,150 to 6,630)	111.2 (90.3 to 133.9)	-9.3 (-29.7 to 17.3)	-48.4 (-58.9 to -35.7)	4,630 (3,690 to 5,730)	77.1 (62.0 to 94.4)	-13.4 (-32.5 to 9.9)	-64.3 (-71.5 to -56.1)
Honduras	745 (611 to 920)	27.2 (22.0 to 33.5)	-8.1 (-32.6 to 25.1)	-26.4 (-43.2 to -4.4)	377 (272 to 750)	13.6 (9.7 to 25.7)	-47.9 (-64.3 to 6.9)	-55.7 (-68.3 to -16.1)
Mexico	12,500 (10,600 to 14,700)	24.6 (20.8 to 28.9)	-24.3 (-38.5 to -8.7)	-50.5 (-58.5 to -41.9)	10,000 (8,460 to 11,700)	17.8 (15.1 to 20.7)	-31.0 (-42.7 to -16.5)	-61.0 (-66.8 to -54.3)
Nicaragua	566 (471 to 682)	29.5 (24.5 to 35.4)	-47.4 (-60.2 to -27.8)	-44.2 (-54.7 to -30.1)	445 (371 to 545)	19.6 (16.3 to 24.6)	-46.9 (-61.0 to -29.0)	-42.4 (-55.6 to -26.7)
Panama	452 (348 to 574)	23.0 (17.7 to 29.2)	112.3 (64.0 to 174.3)	-8.5 (-29.5 to 18.2)	365 (278 to 458)	15.9 (12.1 to 20.0)	120.4 (71.6 to 178.9)	-14.1 (-32.7 to 8.9)
Venezuela (Bolivarian Republic of)	2,960 (2,280 to 3,800)	24.6 (19.1 to 31.4)	34.9 (3.8 to 71.7)	-32.9 (-48.1 to -14.4)	2,590 (1,960 to 3,370)	17.3 (12.9 to 22.7)	36.0 (1.9 to 77.1)	-42.9 (-57.2 to -26.2)

	Male				Female			
	2019		Percent change from 1990 to 2019		2019		Percent change from 1990 to 2019	
	Number of deaths	Age standardized mortality rate (per 100,000)	Number of deaths	Age standardized mortality rate	Number of deaths	Age standardized mortality rate (per 100,000)	Number of deaths	Age standardized mortality rate
Tropical Latin America	44,500 (40,400 to 47,400)	46.9 (42.1 to 50.2)	16.3 (1.2 to 28.8)	-36.5 (-42.7 to -31.2)	45,600 (38,900 to 49,400)	35.6 (30.6 to 38.5)	49.6 (24.2 to 70.2)	-35.6 (-43.7 to -29.1)
Brazil	43,700 (39,700 to 46,600)	47.3 (42.5 to 50.5)	15.8 (0.9 to 28.7)	-37.1 (-43.4 to -31.9)	44,900 (38,400 to 48,600)	35.9 (30.8 to 38.8)	49.9 (24.2 to 71.2)	-36.1 (-44.2 to -29.4)
Paraguay	780 (598 to 988)	32.3 (24.6 to 40.8)	48.7 (9.9 to 94.2)	-6.6 (-28.7 to 21.5)	693 (509 to 881)	23.0 (17.0 to 29.4)	31.0 (-2.4 to 70.0)	-24.7 (-43.0 to -3.3)
**North Africa and Middle East**	**58,500 (50,700 to 66,900)**	**28.4 (24.8 to 32.2)**	**-42.0 (-55.5 to -28.8)**	**-50.4 (-58.2 to -43.3)**	**49,300 (42,000 to 56,900)**	**24.3 (20.9 to 28.1)**	**-49.1 (-60.0 to -37.2)**	**-53.9 (-61.6 to -46.3)**
Afghanistan	9,220 (6,750 to 12,200)	66.0 (53.8 to 80.0)	-25.2 (-49.7 to 13.1)	-56.8 (-67.3 to -42.9)	9,480 (6,620 to 12,500)	57.5 (43.5 to 70.3)	-17.0 (-44.8 to 19.1)	-58.1 (-71.4 to -43.9)
Algeria	2,940 (2,310 to 3,780)	22.0 (17.2 to 28.5)	-30.8 (-55.6 to 3.4)	-57.2 (-68.0 to -44.3)	2,850 (2,210 to 3,690)	25.6 (19.6 to 34.0)	-26.6 (-51.2 to 8.5)	-49.0 (-60.8 to -34.4)
Bahrain	48 (37 to 60)	22.9 (16.6 to 28.4)	122.7 (61.7 to 191.4)	-35.6 (-51.5 to -18.2)	35 (26 to 43)	20.4 (14.2 to 25.0)	116.6 (61.9 to 183.3)	-20.9 (-40.1 to 2.2)
Egypt	12,000 (8,990 to 15,900)	33.5 (25.4 to 44.9)	-56.6 (-68.9 to -39.7)	-60.5 (-70.6 to -46.7)	9,330 (6,870 to 12,500)	35.9 (26.5 to 53.9)	-67.5 (-77.1 to -53.7)	-59.7 (-70.8 to -43.4)

	Male				Female			
	2019		Percent change from 1990 to 2019		2019		Percent change from 1990 to 2019	
	Number of deaths	Age standardized mortality rate (per 100,000)	Number of deaths	Age standardized mortality rate	Number of deaths	Age standardized mortality rate (per 100,000)	Number of deaths	Age standardized mortality rate
Iran (Islamic Republic of)	5,760 (5,160 to 6,240)	17.8 (15.8 to 19.3)	-29.3 (-46.4 to -12.0)	-49.8 (-57.4 to -42.5)	4,450 (3,720 to 4,910)	15.0 (12.4 to 16.7)	-40.1 (-55.5 to -23.6)	-50.8 (-59.8 to -42.7)
Iraq	1,710 (1,370 to 2,110)	14.9 (12.2 to 18.5)	-62.3 (-73.9 to -46.7)	-65.0 (-73.5 to -51.4)	1,460 (1,170 to 1,830)	11.0 (8.9 to 14.8)	-47.5 (-65.6 to -24.1)	-60.3 (-71.5 to -44.9)
Jordan	577 (473 to 705)	19.1 (15.4 to 23.4)	43.2 (5.3 to 94.3)	-42.4 (-55.8 to -26.3)	477 (366 to 607)	18.0 (14.2 to 22.7)	26.3 (-11.2 to 77.8)	-46.4 (-59.0 to -29.5)
Kuwait	457 (371 to 554)	41.7 (33.5 to 50.6)	351.7 (259.5 to 451.9)	41.5 (15.7 to 71.3)	211 (170 to 255)	26.7 (21.3 to 32.5)	134.1 (90.2 to 191.1)	-10.0 (-28.0 to 17.0)
Lebanon	544 (424 to 783)	24.7 (19.2 to 35.7)	59.1 (18.4 to 135.8)	-26.6 (-44.6 to 8.9)	365 (278 to 536)	13.1 (10.0 to 19.2)	19.7 (-14.8 to 69.1)	-44.7 (-58.7 to -19.7)
Libya	455 (347 to 574)	20.2 (15.5 to 25.5)	-0.3 (-29.3 to 37.8)	-31.0 (-49.4 to -7.2)	382 (298 to 490)	17.0 (13.2 to 21.9)	-0.4 (-30.6 to 45.0)	-30.4 (-49.2 to -4.0)
Morocco	3,390 (2,530 to 4,410)	27.7 (20.7 to 36.1)	-45.6 (-61.5 to -24.4)	-46.6 (-59.2 to -31.3)	2,860 (2,220 to 3,710)	22.1 (16.9 to 28.6)	-53.1 (-67.1 to -35.0)	-53.7 (-64.9 to -40.5)
Oman	232 (190 to 277)	48.1 (37.7 to 59.1)	5.9 (-19.8 to 42.3)	-28.6 (-45.0 to -0.4)	176 (135 to 210)	35.4 (26.2 to 43.6)	-20.0 (-40.4 to 9.4)	-31.5 (-47.8 to -0.7)

	Male				Female			
	2019		Percent change from 1990 to 2019		2019		Percent change from 1990 to 2019	
	Number of deaths	Age standardized mortality rate (per 100,000)	Number of deaths	Age standardized mortality rate	Number of deaths	Age standardized mortality rate (per 100,000)	Number of deaths	Age standardized mortality rate
Palestine	276 (237 to 343)	33.9 (28.5 to 44.0)	6.9 (-19.1 to 38.7)	-27.8 (-42.5 to -5.3)	224 (184 to 322)	21.2 (17.2 to 31.4)	-2.1 (-31.9 to 32.6)	-33.0 (-47.7 to -13.5)
Qatar	46 (34 to 64)	21.4 (16.5 to 27.9)	246.4 (141.2 to 395.4)	-14.6 (-38.0 to 19.5)	24 (19 to 32)	37.0 (29.1 to 46.6)	96.3 (42.4 to 169.5)	27.3 (-10.6 to 75.4)
Saudi Arabia	2,770 (2,170 to 3,470)	33.6 (27.9 to 40.4)	86.5 (39.1 to 163.8)	-25.5 (-42.7 to 8.9)	1,930 (1,430 to 2,460)	30.3 (23.9 to 38.6)	45.4 (8.5 to 101.1)	-28.4 (-46.9 to 3.8)
Sudan	3,990 (2,790 to 5,470)	32.3 (22.6 to 43.6)	-57.0 (-77.1 to -27.6)	-56.1 (-73.1 to -38.3)	3,040 (2,150 to 4,050)	26.2 (19.6 to 34.6)	-63.8 (-79.5 to -35.5)	-61.3 (-75.2 to -43.8)
Syrian Arab Republic	1,320 (981 to 1,700)	25.3 (19.3 to 32.0)	-32.8 (-54.7 to 1.8)	-16.7 (-39.8 to 17.7)	967 (735 to 1,230)	23.2 (18.3 to 29.4)	-40.7 (-59.7 to -15.1)	-13.2 (-36.9 to 19.3)
Tunisia	1,030 (760 to 1,410)	21.3 (15.7 to 29.0)	-11.6 (-41.8 to 34.0)	-41.2 (-57.7 to -18.3)	804 (591 to 1,070)	14.3 (10.5 to 19.0)	-31.8 (-56.0 to 0.6)	-56.2 (-69.4 to -40.6)
Turkey	8,000 (6,250 to 9,730)	22.8 (17.9 to 27.5)	-51.2 (-66.3 to -31.5)	-58.9 (-69.9 to -45.8)	6,860 (4,890 to 8,470)	15.9 (11.6 to 19.6)	-56.2 (-69.9 to -36.6)	-68.1 (-77.2 to -55.8)
United Arab Emirates	474 (357 to 623)	53.8 (29.5 to 65.9)	341.5 (214.3 to 534.4)	-36.8 (-51.8 to -10.6)	125 (92 to 162)	42.5 (18.2 to 57.3)	79.7 (29.8 to 151.6)	-48.0 (-60.9 to -26.9)

	Male				Female			
	2019		Percent change from 1990 to 2019		2019		Percent change from 1990 to 2019	
	Number of deaths	Age standardized mortality rate (per 100,000)	Number of deaths	Age standardized mortality rate	Number of deaths	Age standardized mortality rate (per 100,000)	Number of deaths	Age standardized mortality rate
Yemen	3,120 (2,080 to 4,510)	39.2 (25.8 to 58.1)	-41.3 (-70.1 to 3.9)	-43.9 (-66.4 to -18.1)	3,170 (2,210 to 4,280)	33.0 (23.9 to 47.3)	-51.8 (-70.3 to -22.1)	-54.0 (-69.4 to -33.5)
**South Asia**	**262,000 (225,000 to 300,000)**	**41.8 (35.7 to 47.8)**	**-44.4 (-53.0 to -32.5)**	**-51.7 (-58.5 to -43.2)**	**286,000 (245,000 to 333,000)**	**43.4 (37.0 to 50.6)**	**-35.5 (-48.3 to -21.3)**	**-48.7 (-58.2 to -38.4)**
Bangladesh	18,700 (15,200 to 24,000)	31.1 (25.5 to 39.9)	-68.5 (-76.2 to -57.8)	-67.3 (-74.3 to -57.7)	18,600 (13,800 to 22,500)	32.7 (23.3 to 39.7)	-65.9 (-75.3 to -55.3)	-61.3 (-71.2 to -50.7)
Bhutan	83 (51 to 124)	31.8 (19.4 to 47.9)	-70.0 (-83.2 to -36.1)	-57.9 (-74.3 to -27.9)	78 (49 to 110)	31.3 (19.1 to 43.2)	-62.0 (-78.7 to -15.3)	-50.3 (-69.3 to -14.1)
India	202,000 (171,000 to 234,000)	42.3 (35.6 to 49.0)	-42.7 (-52.6 to -28.9)	-52.8 (-60.2 to -43.1)	232,000 (193,000 to 274,000)	45.1 (37.8 to 53.5)	-31.6 (-46.5 to -13.8)	-50.9 (-61.0 to -39.3)
Nepal	4,100 (3,040 to 5,260)	40.1 (28.6 to 52.4)	-71.9 (-80.3 to -59.9)	-63.2 (-75.7 to -47.0)	4,660 (3,540 to 5,780)	43.2 (31.5 to 54.8)	-66.8 (-75.5 to -55.6)	-60.3 (-72.4 to -45.9)
Pakistan	36,800 (27,000 to 48,100)	45.4 (31.8 to 60.6)	-16.4 (-37.5 to 14.3)	-28.7 (-45.4 to -8.9)	31,400 (24,400 to 40,000)	29.0 (23.5 to 36.0)	-14.9 (-39.3 to 18.3)	-35.7 (-51.2 to -16.6)
**Southeast Asia, East Asia, and Oceania**	**236,000 (212,000 to 262,000)**	**29.4 (26.2 to 32.5)**	**-46.4 (-54.3 to -37.5)**	**-57.1 (-62.0 to -51.2)**	**197,000 (166,000 to 221,000)**	**18.4 (15.7 to 20.6)**	**-50.8 (-58.3 to -42.0)**	**-69.0 (-73.1 to -64.5)**

	Male				Female			
	2019		Percent change from 1990 to 2019		2019		Percent change from 1990 to 2019	
	Number of deaths	Age standardized mortality rate (per 100,000)	Number of deaths	Age standardized mortality rate	Number of deaths	Age standardized mortality rate (per 100,000)	Number of deaths	Age standardized mortality rate
East Asia	113,000 (97,100 to 133,000)	21.1 (18.4 to 23.8)	-60.6 (-67.3 to -46.8)	-67.9 (-72.4 to -59.1)	92,000 (75,800 to 115,000)	11.4 (9.6 to 13.9)	-66.4 (-72.9 to -54.6)	-80.8 (-84.0 to -74.7)
China	103,000 (86,800 to 122,000)	19.8 (17.0 to 22.5)	-63.2 (-69.9 to -49.5)	-70.1 (-74.5 to -61.8)	82,700 (67,800 to 105,000)	10.8 (9.0 to 13.4)	-68.8 (-75.0 to -57.2)	-82.0 (-85.3 to -76.0)
Democratic People's Republic of Korea	2,860 (2,300 to 3,500)	34.5 (27.1 to 42.2)	-55.0 (-69.2 to -36.1)	-47.8 (-61.8 to -31.9)	4,140 (3,200 to 5,280)	25.1 (19.5 to 32.0)	-39.3 (-57.7 to -14.3)	-57.3 (-67.9 to -43.7)
Taiwan (Province of China)	7,910 (6,360 to 9,880)	43.7 (34.9 to 54.8)	239.9 (174.2 to 319.8)	10.0 (-11.2 to 36.7)	5,160 (3,990 to 6,410)	22.9 (17.9 to 28.5)	247.7 (180.7 to 326.2)	-13.5 (-29.0 to 6.2)
Oceania	4,210 (3,150 to 5,660)	74.2 (59.0 to 106.1)	30.8 (-1.5 to 74.4)	-30.9 (-44.2 to -13.9)	3,560 (2,670 to 4,630)	65.3 (51.6 to 85.9)	42.2 (5.1 to 89.7)	-24.6 (-40.4 to -6.5)
American Samoa	6 (5 to 7)	31.3 (26.0 to 36.8)	13.2 (-9.4 to 41.0)	-28.5 (-41.6 to -13.1)	6 (5 to 7)	26.7 (21.2 to 33.2)	31.5 (-0.5 to 70.8)	-22.3 (-39.4 to -1.6)
Cook Islands	6 (5 to 7)	56.0 (45.9 to 68.1)	-10.0 (-28.6 to 13.0)	-44.9 (-55.7 to -31.3)	4 (3 to 5)	32.1 (25.0 to 39.9)	-12.4 (-36.1 to 16.0)	-53.4 (-65.6 to -38.5)
Fiji	120 (92 to 153)	45.2 (36.2 to 55.1)	2.6 (-25.7 to 41.7)	-25.1 (-43.2 to -1.3)	96 (72 to 121)	29.1 (22.0 to 36.4)	8.7 (-23.5 to 48.7)	-29.7 (-49.0 to -4.6)

	Male				Female			
	2019		Percent change from 1990 to 2019		2019		Percent change from 1990 to 2019	
	Number of deaths	Age standardized mortality rate (per 100,000)	Number of deaths	Age standardized mortality rate	Number of deaths	Age standardized mortality rate (per 100,000)	Number of deaths	Age standardized mortality rate
Guam	18 (15 to 22)	21.7 (18.0 to 26.4)	56.8 (25.9 to 95.4)	-36.7 (-48.9 to -21.3)	16 (13 to 20)	16.8 (13.6 to 20.5)	56.7 (24.3 to 96.9)	-51.8 (-61.7 to -39.1)
Kiribati	18 (15 to 23)	60.0 (48.8 to 73.0)	-14.9 (-37.9 to 12.1)	-28.7 (-44.5 to -9.8)	17 (13 to 21)	49.2 (38.5 to 60.5)	-14.7 (-38.9 to 15.6)	-27.8 (-44.2 to -4.4)
Marshall Islands	11 (9 to 15)	67.4 (53.1 to 86.7)	-5.6 (-27.7 to 21.6)	-30.0 (-44.6 to -11.2)	9 (6 to 12)	61.0 (45.9 to 82.4)	-1.2 (-27.5 to 33.1)	-13.2 (-35.4 to 13.2)
Micronesia (Federated States of)	21 (15 to 27)	72.0 (56.0 to 90.7)	-36.9 (-55.2 to -16.8)	-27.2 (-44.4 to -8.3)	18 (13 to 24)	59.8 (46.0 to 80.1)	-35.8 (-53.4 to -14.8)	-27.8 (-45.8 to -3.8)
Nauru	2 (2 to 3)	81.9 (64.7 to 103.7)	-34.6 (-48.3 to -16.1)	-17.7 (-35.3 to 2.4)	2 (1 to 2)	62.3 (48.0 to 81.4)	-39.0 (-53.5 to -19.7)	-24.3 (-40.6 to -2.7)
Niue	0 (0 to 1)	51.6 (42.3 to 64.9)	-37.5 (-50.5 to -20.9)	-27.6 (-42.6 to -8.4)	0 (0 to 1)	36.9 (27.8 to 48.7)	-46.4 (-59.4 to -26.8)	-27.6 (-45.2 to -1.6)
Northern Mariana Islands	7 (6 to 8)	38.9 (33.4 to 45.0)	31.6 (4.5 to 65.2)	-24.5 (-38.0 to -9.3)	4 (3 to 4)	21.8 (17.1 to 26.7)	27.0 (-3.6 to 64.9)	-39.1 (-54.2 to -21.2)
Palau	10 (8 to 12)	119.2 (98.7 to 144.0)	16.1 (-12.9 to 54.0)	-27.8 (-44.2 to -8.3)	8 (6 to 10)	116.3 (90.9 to 143.6)	4.2 (-23.8 to 38.8)	-27.7 (-46.5 to -3.6)

	Male				Female			
	2019		Percent change from 1990 to 2019		2019		Percent change from 1990 to 2019	
	Number of deaths	Age standardized mortality rate (per 100,000)	Number of deaths	Age standardized mortality rate	Number of deaths	Age standardized mortality rate (per 100,000)	Number of deaths	Age standardized mortality rate
Papua New Guinea	3,410 (2,470 to 4,730)	73.7 (55.5 to 118.2)	38.7 (-0.1 to 92.2)	-32.5 (-48.3 to -11.5)	2,900 (2,110 to 3,880)	68.5 (52.4 to 95.2)	51.1 (6.7 to 109.2)	-25.2 (-43.2 to -3.7)
Samoa	31 (25 to 40)	48.2 (38.9 to 60.3)	-12.6 (-35.7 to 18.0)	-37.0 (-51.1 to -19.9)	32 (24 to 43)	45.4 (34.0 to 61.1)	2.3 (-28.2 to 39.2)	-26.7 (-46.8 to 0.3)
Solomon Islands	273 (221 to 328)	161.8 (133.3 to 191.6)	2.9 (-20.2 to 36.5)	-40.9 (-53.4 to -14.9)	206 (167 to 252)	141.1 (111.7 to 166.4)	13.5 (-13.6 to 51.5)	-35.9 (-51.0 to -13.8)
Tokelau	0 (0 to 0)	34.6 (27.4 to 43.6)	-49.5 (-61.6 to -34.4)	-39.4 (-52.3 to -24.2)	0 (0 to 0)	44.0 (33.9 to 57.3)	-55.1 (-67.2 to -39.6)	-40.9 (-56.8 to -21.0)
Tonga	18 (15 to 23)	54.0 (43.4 to 65.2)	5.3 (-16.6 to 34.2)	-17.8 (-34.8 to 4.5)	16 (12 to 20)	33.7 (25.9 to 43.3)	15.1 (-12.4 to 53.2)	-25.8 (-43.0 to -0.6)
Tuvalu	2 (2 to 3)	57.0 (44.7 to 75.8)	-49.3 (-65.5 to -28.7)	-51.6 (-64.6 to -34.3)	2 (2 to 3)	50.6 (38.3 to 68.2)	-54.9 (-68.5 to -36.8)	-54.1 (-66.6 to -38.1)
Vanuatu	64 (49 to 85)	68.8 (52.3 to 89.8)	62.2 (24.0 to 120.6)	-11.9 (-31.1 to 13.8)	49 (36 to 65)	57.2 (42.7 to 76.9)	57.8 (13.9 to 120.5)	-17.8 (-42.1 to 16.0)
Southeast Asia	118,000 (101,000 to 134,000)	54.0 (45.4 to 61.0)	-20.8 (-36.2 to -3.1)	-32.3 (-42.2 to -20.2)	101,000 (76,900 to 115,000)	38.0 (28.8 to 43.1)	-18.5 (-38.0 to 0.4)	-38.5 (-53.3 to -26.3)

	Male				Female			
	2019		Percent change from 1990 to 2019		2019		Percent change from 1990 to 2019	
	Number of deaths	Age standardized mortality rate (per 100,000)	Number of deaths	Age standardized mortality rate	Number of deaths	Age standardized mortality rate (per 100,000)	Number of deaths	Age standardized mortality rate
Cambodia	6,000 (4,980 to 7,070)	128.9 (107.4 to 150.2)	-54.5 (-63.9 to -42.3)	-45.0 (-56.0 to -33.5)	6,070 (4,920 to 7,010)	105.0 (83.4 to 121.0)	-47.9 (-58.2 to -34.1)	-48.3 (-57.9 to -36.8)
Indonesia	23,800 (20,000 to 27,900)	33.3 (28.1 to 38.5)	-53.7 (-63.0 to -39.1)	-51.3 (-60.1 to -37.0)	20,500 (16,000 to 23,700)	24.6 (19.2 to 28.3)	-48.9 (-61.8 to -34.3)	-49.6 (-60.3 to -36.0)
Lao People's Democratic Republic	1,830 (1,380 to 2,320)	84.3 (65.5 to 103.5)	-66.3 (-75.7 to -53.2)	-60.1 (-69.4 to -48.0)	1,570 (1,150 to 2,000)	69.0 (51.3 to 87.0)	-65.2 (-75.0 to -53.2)	-60.8 (-70.6 to -49.2)
Malaysia	12,400 (7,880 to 15,600)	112.1 (72.2 to 140.3)	327.7 (111.4 to 461.5)	77.1 (-15.5 to 133.8)	9,350 (5,230 to 11,800)	89.4 (50.9 to 112.6)	302.6 (87.0 to 440.6)	78.7 (-18.0 to 145.1)
Maldives	18 (15 to 22)	13.1 (10.3 to 15.9)	-7.2 (-38.2 to 33.4)	-48.3 (-61.0 to -32.7)	16 (13 to 20)	14.2 (10.7 to 17.4)	-24.4 (-48.7 to 11.0)	-59.1 (-69.5 to -43.5)
Mauritius	162 (133 to 195)	26.6 (22.0 to 31.9)	-11.4 (-27.6 to 7.5)	-61.2 (-68.2 to -53.5)	127 (103 to 153)	14.9 (12.0 to 17.9)	-7.2 (-22.9 to 12.4)	-62.8 (-69.1 to -54.8)
Myanmar	11,700 (9,460 to 14,500)	64.4 (53.4 to 77.0)	-58.3 (-73.6 to -33.5)	-55.2 (-67.1 to -37.3)	9,720 (7,130 to 12,100)	43.2 (31.8 to 53.1)	-65.4 (-77.5 to -43.1)	-64.8 (-75.4 to -48.8)
Philippines	30,400 (24,700 to 36,600)	101.6 (80.3 to 124.1)	30.8 (2.3 to 62.3)	2.4 (-20.9 to 24.6)	28,000 (19,800 to 33,900)	85.7 (56.5 to 104.0)	49.3 (5.4 to 90.0)	-4.7 (-41.7 to 18.8)

	Male				Female			
	2019		Percent change from 1990 to 2019		2019		Percent change from 1990 to 2019	
	Number of deaths	Age standardized mortality rate (per 100,000)	Number of deaths	Age standardized mortality rate	Number of deaths	Age standardized mortality rate (per 100,000)	Number of deaths	Age standardized mortality rate
Seychelles	44 (35 to 52)	108.8 (88.0 to 127.4)	55.7 (26.8 to 89.6)	-13.2 (-29.3 to 5.1)	34 (26 to 40)	63.1 (48.5 to 75.0)	59.7 (29.5 to 93.8)	5.2 (-14.2 to 27.8)
Sri Lanka	2,610 (1,910 to 3,490)	29.1 (21.5 to 38.7)	-12.7 (-37.7 to 20.0)	-56.1 (-68.1 to -38.8)	2,090 (1,530 to 3,030)	17.9 (13.3 to 26.6)	-5.9 (-33.3 to 52.8)	-65.0 (-74.8 to -42.9)
Thailand	17,200 (10,300 to 22,600)	42.1 (25.8 to 54.9)	181.9 (26.4 to 295.6)	18.8 (-49.1 to 65.9)	13,900 (8,020 to 18,400)	26.1 (15.4 to 34.4)	197.9 (17.0 to 334.8)	2.9 (-60.3 to 50.6)
Timor-Leste	321 (234 to 410)	80.1 (55.2 to 102.8)	-56.9 (-70.4 to -39.0)	-40.5 (-56.6 to -20.5)	280 (209 to 351)	70.0 (51.9 to 88.0)	-57.2 (-69.0 to -38.5)	-48.3 (-60.6 to -32.2)
Viet Nam	11,700 (9,700 to 14,100)	39.5 (33.4 to 47.4)	-20.5 (-38.6 to 3.0)	-31.3 (-45.7 to -11.2)	9,620 (7,610 to 12,800)	21.4 (17.0 to 28.1)	-13.5 (-34.2 to 15.1)	-44.0 (-57.1 to -24.7)
**Sub-Saharan Africa**	**364,000 (306,000 to 436,000)**	**116.9 (103.1 to 131.6)**	**-13.8 (-30.2 to 8.3)**	**-35.0 (-42.6 to -24.8)**	**314,000 (263,000 to 373,000)**	**86.8 (74.2 to 99.4)**	**-11.0 (-29.7 to 10.5)**	**-37.1 (-45.7 to -27.4)**
Central Sub-Saharan Africa	34,700 (26,500 to 45,700)	132.4 (102.5 to 173.4)	-25.2 (-44.3 to -2.1)	-36.6 (-47.9 to -22.8)	31,500 (23,800 to 41,100)	91.6 (67.7 to 125.9)	-20.8 (-43.6 to 6.9)	-37.7 (-51.2 to -21.5)
Angola	6,990 (5,540 to 8,770)	117.8 (98.3 to 141.5)	-28.6 (-57.5 to 18.6)	-42.1 (-58.2 to -17.7)	5,790 (4,310 to 7,510)	71.5 (56.4 to 90.3)	-41.1 (-66.7 to 10.7)	-54.8 (-68.7 to -28.8)

	Male				Female			
	2019		Percent change from 1990 to 2019		2019		Percent change from 1990 to 2019	
	Number of deaths	Age standardized mortality rate (per 100,000)	Number of deaths	Age standardized mortality rate	Number of deaths	Age standardized mortality rate (per 100,000)	Number of deaths	Age standardized mortality rate
Central African Republic	3,180 (2,130 to 4,380)	199.4 (145.1 to 263.0)	14.6 (-21.2 to 63.2)	-20.8 (-38.5 to 0.2)	2,500 (1,770 to 3,640)	133.6 (92.3 to 184.6)	12.9 (-26.9 to 69.5)	-18.8 (-40.6 to 9.9)
Congo	1,050 (790 to 1,340)	98.1 (73.5 to 124.0)	-5.0 (-28.0 to 25.7)	-40.9 (-52.7 to -25.1)	984 (731 to 1,290)	81.6 (60.7 to 104.7)	4.7 (-26.2 to 41.5)	-29.9 (-47.5 to -8.0)
Democratic Republic of the Congo	22,800 (16,200 to 32,100)	136.3 (97.3 to 190.6)	-28.2 (-47.7 to -3.4)	-35.3 (-48.5 to -20.1)	21,800 (15,300 to 30,300)	96.5 (67.1 to 141.8)	-16.6 (-42.8 to 14.6)	-34.3 (-50.2 to -15.3)
Equatorial Guinea	191 (136 to 262)	96.9 (74.5 to 122.6)	-54.3 (-71.6 to -23.3)	-54.0 (-69.0 to -34.5)	172 (118 to 249)	67.1 (47.7 to 92.0)	-50.4 (-72.6 to -10.5)	-52.0 (-69.7 to -22.8)
Gabon	418 (310 to 532)	107.4 (80.6 to 135.9)	-12.0 (-33.3 to 15.9)	-30.5 (-44.5 to -13.7)	279 (199 to 371)	55.2 (38.8 to 73.3)	-22.3 (-44.0 to 6.6)	-39.1 (-54.9 to -19.2)
Eastern Sub-Saharan Africa	113,000 (96,000 to 134,000)	111.8 (99.3 to 125.2)	-35.4 (-48.2 to -16.9)	-43.8 (-51.6 to -33.6)	97,400 (81,800 to 116,000)	82.6 (70.3 to 93.7)	-29.5 (-46.3 to -13.1)	-44.0 (-52.1 to -34.5)
Burundi	3,340 (2,380 to 4,700)	120.9 (92.5 to 153.8)	-26.7 (-48.5 to 10.3)	-41.1 (-55.0 to -21.0)	3,120 (2,120 to 4,310)	99.6 (69.7 to 138.1)	-22.4 (-49.4 to 14.0)	-35.3 (-53.8 to -12.2)
Comoros	208 (159 to 268)	95.6 (74.1 to 121.4)	-41.1 (-57.7 to -1.7)	-40.1 (-54.9 to -10.5)	242 (177 to 303)	89.5 (65.3 to 112.8)	-29.5 (-48.6 to 17.1)	-37.1 (-51.6 to -6.5)

	Male				Female			
	2019		Percent change from 1990 to 2019		2019		Percent change from 1990 to 2019	
	Number of deaths	Age standardized mortality rate (per 100,000)	Number of deaths	Age standardized mortality rate	Number of deaths	Age standardized mortality rate (per 100,000)	Number of deaths	Age standardized mortality rate
Djibouti	347 (256 to 476)	108.4 (84.8 to 142.0)	35.7 (-9.5 to 107.7)	-23.8 (-40.7 to -0.5)	246 (178 to 333)	78.8 (60.2 to 103.5)	16.7 (-25.7 to 81.2)	-28.4 (-47.8 to -1.9)
Eritrea	2,440 (1,440 to 3,940)	189.4 (107.0 to 313.1)	-20.7 (-55.3 to 26.8)	-29.5 (-51.0 to 5.9)	1,990 (1,290 to 3,270)	123.4 (78.8 to 213.6)	-9.2 (-50.6 to 63.6)	-25.9 (-53.9 to 20.7)
Ethiopia	26,100 (21,900 to 31,800)	100.7 (84.1 to 121.4)	-58.3 (-68.6 to -40.5)	-61.8 (-71.2 to -46.2)	20,200 (16,500 to 24,400)	71.8 (60.2 to 83.0)	-55.9 (-69.8 to -40.4)	-60.7 (-70.0 to -47.0)
Kenya	11,600 (9,410 to 14,000)	115.9 (94.6 to 141.2)	-4.7 (-25.2 to 20.3)	-15.7 (-28.5 to -1.5)	9,930 (7,890 to 12,600)	81.0 (62.4 to 104.5)	-1.0 (-26.0 to 28.8)	-25.8 (-40.4 to -11.8)
Madagascar	6,840 (5,210 to 8,940)	104.2 (83.4 to 130.7)	-29.5 (-47.6 to -7.4)	-37.1 (-50.6 to -20.2)	6,830 (5,140 to 8,710)	94.8 (70.4 to 121.0)	-17.8 (-40.8 to 8.9)	-30.4 (-47.9 to -10.4)
Malawi	5,040 (4,040 to 6,260)	123.5 (104.3 to 143.7)	-33.8 (-50.6 to -10.3)	-32.2 (-46.1 to -14.3)	4,170 (3,290 to 5,180)	76.2 (62.1 to 91.4)	-41.1 (-57.1 to -17.9)	-47.9 (-59.7 to -32.2)
Mozambique	8,280 (6,430 to 10,500)	131.4 (108.3 to 159.4)	-25.7 (-51.0 to 23.1)	-23.4 (-40.7 to 3.0)	7,240 (5,230 to 9,540)	79.2 (58.8 to 101.2)	-25.8 (-57.6 to 27.2)	-40.7 (-58.8 to -14.4)
Rwanda	2,820 (2,150 to 3,700)	101.2 (78.0 to 126.3)	-59.5 (-71.8 to -35.2)	-52.9 (-64.7 to -36.9)	2,820 (2,120 to 3,580)	76.3 (56.1 to 95.7)	-47.7 (-65.5 to -25.6)	-50.8 (-61.7 to -35.3)

	Male				Female			
	2019		Percent change from 1990 to 2019		2019		Percent change from 1990 to 2019	
	Number of deaths	Age standardized mortality rate (per 100,000)	Number of deaths	Age standardized mortality rate	Number of deaths	Age standardized mortality rate (per 100,000)	Number of deaths	Age standardized mortality rate
Somalia	11,600 (7,930 to 16,400)	173.1 (132.4 to 221.4)	36.6 (-7.3 to 108.6)	-29.1 (-45.1 to -7.0)	9,130 (6,350 to 13,000)	125.1 (90.2 to 172.4)	57.9 (9.8 to 128.3)	-25.8 (-45.4 to 0.7)
South Sudan	4,490 (3,290 to 5,970)	120.5 (94.9 to 150.6)	-28.8 (-49.9 to -1.7)	-37.8 (-52.2 to -21.6)	3,230 (2,290 to 4,640)	90.0 (69.4 to 115.8)	-28.7 (-52.1 to 4.0)	-39.0 (-52.5 to -21.3)
Uganda	8,620 (6,670 to 11,000)	104.6 (84.5 to 126.3)	-12.0 (-38.6 to 27.8)	-23.0 (-39.1 to -2.9)	7,870 (5,990 to 10,200)	75.1 (57.2 to 94.1)	14.3 (-26.2 to 72.0)	-18.6 (-40.5 to 12.2)
United Republic of Tanzania	16,400 (12,800 to 20,800)	101.0 (83.4 to 120.3)	-31.2 (-49.6 to -6.8)	-47.4 (-58.3 to -34.8)	16,600 (12,900 to 21,100)	85.3 (69.4 to 103.1)	-21.7 (-41.4 to 8.3)	-45.0 (-56.7 to -30.9)
Zambia	4,490 (3,540 to 5,620)	110.4 (91.2 to 134.0)	-40.1 (-56.8 to -13.4)	-42.6 (-54.3 to -26.7)	3,700 (2,940 to 4,640)	76.9 (61.8 to 95.8)	-43.9 (-60.4 to -20.8)	-51.0 (-63.0 to -36.2)
Southern Sub-Saharan Africa	26,300 (23,500 to 29,500)	109.3 (98.7 to 119.9)	19.1 (3.9 to 37.9)	-12.0 (-21.8 to -1.7)	21,200 (18,000 to 24,100)	68.3 (58.2 to 76.8)	11.8 (-2.9 to 28.3)	-21.0 (-30.2 to -10.6)
Botswana	859 (645 to 1,140)	144.1 (113.2 to 182.6)	70.8 (25.4 to 129.4)	-14.3 (-36.2 to 13.6)	669 (455 to 958)	89.0 (62.1 to 126.8)	83.4 (31.7 to 151.9)	-7.9 (-33.8 to 28.6)
Eswatini	431 (317 to 571)	145.8 (113.1 to 187.8)	8.0 (-21.8 to 47.6)	-5.2 (-23.7 to 20.7)	290 (197 to 396)	78.3 (53.6 to 106.0)	-0.3 (-31.9 to 42.6)	-18.2 (-43.4 to 14.7)

	Male				Female			
	2019		Percent change from 1990 to 2019		2019		Percent change from 1990 to 2019	
	Number of deaths	Age standardized mortality rate (per 100,000)	Number of deaths	Age standardized mortality rate	Number of deaths	Age standardized mortality rate (per 100,000)	Number of deaths	Age standardized mortality rate
Lesotho	1,120 (887 to 1,370)	187.8 (153.5 to 225.7)	16.3 (-9.4 to 49.2)	15.0 (-6.6 to 43.2)	820 (605 to 1,070)	110.3 (81.5 to 142.3)	6.2 (-23.7 to 46.1)	15.6 (-16.5 to 59.1)
Namibia	865 (636 to 1,180)	143.3 (107.8 to 191.7)	23.0 (-8.4 to 71.4)	-16.5 (-34.3 to 7.7)	596 (421 to 841)	71.2 (50.9 to 99.4)	15.9 (-19.1 to 66.6)	-31.6 (-50.8 to -5.2)
South Africa	15,700 (14,200 to 17,300)	88.9 (80.5 to 97.1)	6.7 (-7.9 to 22.6)	-20.4 (-30.2 to -11.1)	13,300 (11,600 to 15,000)	56.4 (49.3 to 63.3)	-1.8 (-14.8 to 13.1)	-32.2 (-39.6 to -23.7)
Zimbabwe	7,310 (5,870 to 8,860)	196.7 (158.1 to 232.0)	53.0 (17.8 to 100.4)	17.0 (-5.9 to 44.7)	5,590 (4,130 to 7,130)	122.7 (80.4 to 161.1)	57.8 (17.6 to 113.5)	10.0 (-16.4 to 45.3)
Western Sub-Saharan Africa	190,000 (155,000 to 233,000)	119.9 (101.7 to 140.4)	6.2 (-16.2 to 40.2)	-30.3 (-40.8 to -16.1)	164,000 (134,000 to 200,000)	94.1 (78.9 to 111.4)	5.3 (-19.0 to 35.4)	-33.9 (-45.7 to -20.3)
Benin	4,810 (3,420 to 6,700)	131.7 (100.7 to 167.1)	2.8 (-29.6 to 48.4)	-33.4 (-48.2 to -15.2)	4,240 (2,960 to 5,810)	95.8 (73.1 to 123.6)	4.7 (-28.7 to 51.3)	-37.3 (-52.9 to -19.0)
Burkina Faso	12,600 (9,060 to 17,200)	163.2 (133.0 to 199.8)	31.1 (-6.9 to 90.0)	-25.8 (-40.8 to -6.5)	11,700 (8,730 to 15,600)	119.2 (96.4 to 145.3)	29.8 (-9.8 to 88.1)	-30.2 (-46.8 to -8.9)
Cabo Verde	168 (145 to 192)	98.9 (84.6 to 112.1)	74.8 (37.9 to 120.7)	40.8 (13.5 to 72.8)	126 (104 to 156)	48.5 (40.0 to 60.3)	23.0 (-2.9 to 62.3)	-15.0 (-31.7 to 9.6)

	Male				Female			
	2019		Percent change from 1990 to 2019		2019		Percent change from 1990 to 2019	
	Number of deaths	Age standardized mortality rate (per 100,000)	Number of deaths	Age standardized mortality rate	Number of deaths	Age standardized mortality rate (per 100,000)	Number of deaths	Age standardized mortality rate
Cameroon	9,230 (6,640 to 12,200)	129.3 (94.8 to 168.5)	62.0 (15.5 to 127.3)	-13.7 (-32.9 to 12.2)	7,790 (5,620 to 11,200)	91.1 (67.2 to 122.8)	46.6 (-0.8 to 114.9)	-26.9 (-45.8 to -1.5)
Chad	11,000 (8,070 to 14,400)	165.5 (135.0 to 200.6)	60.1 (14.1 to 125.2)	-20.9 (-40.9 to 3.1)	8,480 (6,090 to 11,600)	129.4 (101.2 to 159.9)	46.5 (1.3 to 109.6)	-22.8 (-41.5 to 1.3)
Côte d'Ivoire	9,650 (7,210 to 12,500)	135.4 (104.1 to 168.9)	3.8 (-24.5 to 41.7)	-30.8 (-45.2 to -13.8)	7,330 (5,170 to 9,570)	93.2 (70.3 to 116.7)	-5.2 (-33.7 to 33.7)	-33.5 (-49.0 to -14.0)
Gambia	678 (526 to 840)	141.8 (108.7 to 176.4)	2.7 (-25.1 to 39.3)	-21.0 (-41.4 to 3.3)	546 (420 to 681)	98.4 (75.2 to 123.3)	13.6 (-20.3 to 60.5)	-18.5 (-40.0 to 10.5)
Ghana	10,700 (8,580 to 12,800)	155.0 (126.9 to 185.5)	60.3 (16.6 to 115.4)	-5.9 (-28.5 to 22.8)	5,620 (4,480 to 7,030)	60.7 (49.5 to 73.9)	-0.3 (-29.7 to 39.0)	-42.6 (-57.6 to -22.1)
Guinea	7,390 (5,530 to 9,820)	171.9 (133.0 to 217.4)	-15.9 (-40.5 to 19.4)	-22.8 (-40.5 to 3.3)	6,160 (4,450 to 8,230)	134.8 (100.1 to 173.7)	-24.7 (-48.5 to 6.7)	-37.1 (-53.1 to -16.8)
Guinea-Bissau	612 (475 to 779)	172.8 (136.9 to 209.4)	-36.8 (-55.7 to -9.0)	-33.8 (-49.3 to -13.0)	509 (392 to 648)	112.9 (84.5 to 143.5)	-25.9 (-49.6 to 6.5)	-31.6 (-50.4 to -5.4)
Liberia	1,010 (768 to 1,320)	91.5 (70.7 to 116.3)	-56.0 (-68.4 to -38.3)	-55.2 (-65.4 to -41.2)	927 (658 to 1,220)	81.2 (58.2 to 103.3)	-52.9 (-67.2 to -34.2)	-55.3 (-67.8 to -41.5)

	Male				Female			
	2019		Percent change from 1990 to 2019		2019		Percent change from 1990 to 2019	
	Number of deaths	Age standardized mortality rate (per 100,000)	Number of deaths	Age standardized mortality rate	Number of deaths	Age standardized mortality rate (per 100,000)	Number of deaths	Age standardized mortality rate
Mali	6,960 (4,840 to 9,810)	69.2 (49.4 to 94.0)	30.6 (-14.6 to 95.3)	-34.7 (-53.6 to -9.6)	8,220 (5,880 to 10,900)	73.3 (53.5 to 94.3)	60.0 (10.8 to 135.8)	-27.5 (-46.9 to -3.3)
Mauritania	935 (683 to 1,270)	86.3 (66.6 to 110.7)	-19.8 (-42.2 to 11.6)	-44.8 (-57.3 to -28.7)	862 (630 to 1,150)	79.3 (59.4 to 100.2)	-21.3 (-45.4 to 8.3)	-39.0 (-54.6 to -19.3)
Niger	12,200 (8,620 to 16,800)	138.6 (105.3 to 177.9)	-10.3 (-44.0 to 44.9)	-46.7 (-61.3 to -23.7)	11,400 (8,110 to 15,500)	119.4 (92.0 to 152.3)	-7.1 (-45.0 to 48.4)	-48.1 (-62.6 to -29.0)
Nigeria	92,100 (73,300 to 117,000)	103.9 (83.6 to 129.5)	0.4 (-26.4 to 43.6)	-36.2 (-50.6 to -14.1)	80,900 (64,900 to 100,000)	91.5 (74.1 to 111.9)	4.1 (-26.1 to 49.1)	-34.2 (-51.1 to -9.9)
Sao Tome and Principe	57 (45 to 70)	121.9 (96.8 to 148.6)	-24.1 (-44.9 to 4.1)	-24.2 (-41.4 to -1.6)	52 (38 to 67)	101.8 (75.7 to 133.3)	-27.9 (-48.9 to -1.8)	-26.2 (-45.2 to -1.7)
Senegal	3,570 (2,690 to 4,560)	84.9 (66.6 to 108.1)	-22.3 (-43.6 to 7.8)	-37.4 (-51.9 to -19.3)	3,220 (2,410 to 4,050)	71.5 (55.3 to 89.5)	-18.6 (-42.1 to 14.7)	-36.8 (-52.3 to -17.4)
Sierra Leone	4,040 (2,900 to 5,490)	147.6 (112.4 to 190.0)	-16.1 (-42.3 to 21.2)	-39.1 (-54.2 to -19.4)	3,420 (2,380 to 4,760)	118.6 (88.3 to 153.9)	-17.7 (-45.0 to 22.1)	-37.7 (-54.3 to -16.5)
Togo	2,320 (1,810 to 3,030)	142.3 (114.6 to 173.9)	16.8 (-15.0 to 56.4)	-13.6 (-30.9 to 7.5)	2,030 (1,530 to 2,650)	92.3 (69.9 to 117.8)	6.9 (-23.9 to 51.5)	-31.6 (-46.7 to -12.0)

Appendix Table 4. Lower respiratory infection deaths and age standardized mortality rates attributable to all risk factors combined in 2019 and the percent change in attributable deaths and age standardized mortality rates between 1990 and 2019 for 21 GBD regions and 204 countries and territories (95% UI)

	Male				Female			
	2019		Percent change from 1990 to 2019		2019		Percent change from 1990 to 2019	
	Number of attributable deaths	Attributable mortality rate (per 100,000)	Number of attributable deaths	Attributable mortality rate	Number of attributable deaths	Attributable mortality rate (per 100,000)	Number of attributable deaths	Attributable mortality rate
**Global**	**876,000 (770,000 to 987,000)**	**26.2 (23.1 to 29.5)**	**-41.4 (-49.3 to -32.0)**	**-56.1 (-60.9 to -50.0)**	**725,000 (629,000 to 826,000)**	**19.4 (16.8 to 22.2)**	**-44.5 (-53.9 to -34.9)**	**-59.1 (-65.6 to -52.5)**
**Central Europe, Eastern Europe, and Central Asia**	**40,400 (34,900 to 45,500)**	**19.4 (16.8 to 21.8)**	**-23.3 (-32.7 to -13.5)**	**-38.6 (-46.1 to -30.9)**	**19,700 (16,500 to 23,000)**	**8.4 (7.1 to 9.8)**	**-47.5 (-54.4 to -39.9)**	**-58.6 (-64.3 to -51.6)**
Central Asia	10,200 (8,560 to 12,200)	26.3 (22.5 to 30.8)	-61.1 (-67.9 to -52.9)	-57.2 (-63.8 to -49.4)	6,960 (5,730 to 8,500)	16.5 (13.7 to 20.1)	-66.9 (-72.8 to -59.4)	-65.4 (-71.1 to -57.9)
Armenia	197 (163 to 234)	14.2 (11.8 to 16.8)	-60.5 (-68.1 to -51.5)	-51.9 (-60.7 to -41.8)	134 (105 to 167)	8.0 (6.4 to 9.9)	-69.6 (-76.7 to -61.0)	-68.6 (-75.3 to -59.6)
Azerbaijan	981 (743 to 1,260)	27.6 (21.4 to 34.6)	-75.1 (-81.3 to -66.9)	-69.2 (-76.6 to -60.0)	776 (582 to 1,020)	23.2 (17.3 to 30.4)	-77.4 (-83.3 to -69.6)	-70.8 (-78.5 to -60.9)
Georgia	270 (222 to 325)	12.7 (10.6 to 15.1)	-68.4 (-73.8 to -62.3)	-68.2 (-73.4 to -62.2)	166 (128 to 207)	5.0 (3.9 to 6.1)	-72.5 (-77.9 to -66.3)	-81.5 (-85.0 to -77.6)
Kazakhstan	1,610 (1,350 to 1,910)	22.0 (18.4 to 26.0)	-44.4 (-53.2 to -33.1)	-41.4 (-50.2 to -30.3)	752 (614 to 898)	8.4 (6.8 to 10.0)	-64.6 (-70.7 to -57.3)	-66.7 (-72.3 to -60.0)
Kyrgyzstan	325 (268 to 379)	12.0 (10.1 to 14.0)	-78.2 (-82.0 to -74.0)	-78.1 (-81.5 to -74.4)	224 (179 to 265)	7.3 (5.8 to 8.6)	-81.5 (-85.2 to -77.2)	-82.6 (-85.9 to -78.8)

	Male				Female			
	2019		Percent change from 1990 to 2019		2019		Percent change from 1990 to 2019	
	**Number of attributable deaths**	**Attributable mortality rate (per 100,000)**	**Number of attributable deaths**	**Attributable mortality rate**	**Number of attributable deaths**	**Attributable mortality rate (per 100,000)**	**Number of attributable deaths**	**Attributable mortality rate**
Mongolia	219 (160 to 289)	16.3 (12.7 to 20.8)	-85.1 (-89.5 to -77.2)	-84.3 (-88.4 to -77.2)	171 (126 to 231)	10.6 (8.0 to 14.1)	-85.5 (-89.8 to -79.2)	-86.4 (-90.1 to -80.6)
Tajikistan	1,510 (1,110 to 2,030)	38.5 (30.4 to 49.9)	-61.4 (-72.2 to -46.4)	-60.7 (-69.9 to -47.8)	1,030 (757 to 1,430)	25.3 (19.3 to 34.1)	-68.7 (-78.0 to -55.0)	-68.7 (-77.0 to -56.5)
Turkmenistan	569 (429 to 747)	22.8 (17.7 to 29.5)	-75.7 (-82.8 to -66.0)	-73.1 (-80.3 to -63.4)	387 (308 to 488)	15.1 (12.0 to 19.1)	-78.4 (-83.2 to -71.4)	-76.2 (-81.4 to -68.7)
Uzbekistan	4,470 (3,640 to 5,530)	31.5 (26.1 to 37.9)	-48.7 (-59.7 to -35.5)	-44.2 (-55.1 to -32.2)	3,320 (2,700 to 4,090)	22.6 (18.6 to 27.4)	-52.4 (-61.8 to -39.6)	-49.5 (-58.9 to -37.3)
Central Europe	10,600 (8,930 to 12,200)	13.5 (11.5 to 15.6)	-21.0 (-31.9 to -10.0)	-48.8 (-56.0 to -41.8)	6,370 (5,200 to 7,640)	5.6 (4.6 to 6.6)	-31.6 (-41.1 to -20.3)	-64.3 (-69.3 to -58.4)
Albania	173 (130 to 225)	11.7 (9.1 to 14.8)	-81.6 (-86.7 to -69.9)	-83.6 (-87.5 to -68.8)	107 (80 to 140)	6.9 (5.3 to 9.1)	-87.0 (-90.6 to -80.7)	-86.2 (-89.8 to -79.5)
Bosnia and Herzegovina	150 (114 to 195)	6.9 (5.4 to 8.9)	-15.6 (-36.1 to 11.4)	-46.2 (-58.4 to -26.7)	112 (82 to 172)	3.7 (2.8 to 5.6)	-30.9 (-49.0 to 12.8)	-59.0 (-69.0 to -34.9)
Bulgaria	772 (605 to 970)	15.4 (12.1 to 19.2)	-57.3 (-66.3 to -46.8)	-63.5 (-71.1 to -54.9)	401 (308 to 513)	6.4 (5.0 to 8.1)	-61.1 (-69.4 to -51.7)	-72.3 (-78.2 to -65.2)

	Male				Female			
	2019		Percent change from 1990 to 2019		2019		Percent change from 1990 to 2019	
	**Number of attributable deaths**	**Attributable mortality rate (per 100,000)**	**Number of attributable deaths**	**Attributable mortality rate**	**Number of attributable deaths**	**Attributable mortality rate (per 100,000)**	**Number of attributable deaths**	**Attributable mortality rate**
Croatia	192 (151 to 241)	5.9 (4.6 to 7.3)	-42.0 (-54.2 to -27.4)	-63.9 (-71.2 to -55.5)	128 (97 to 162)	2.3 (1.7 to 2.9)	-48.7 (-60.0 to -36.9)	-70.5 (-76.7 to -63.7)
Czechia	1,090 (866 to 1,330)	13.3 (10.7 to 16.2)	40.1 (14.3 to 69.5)	-21.5 (-35.5 to -5.6)	734 (560 to 925)	5.6 (4.3 to 7.0)	13.6 (-8.3 to 38.4)	-34.2 (-46.6 to -19.7)
Hungary	374 (300 to 460)	5.6 (4.5 to 6.9)	-28.3 (-40.9 to -13.2)	-50.6 (-59.2 to -40.2)	287 (218 to 359)	2.5 (1.9 to 3.1)	-20.0 (-34.9 to -3.9)	-57.1 (-64.9 to -47.9)
Montenegro	28 (22 to 36)	7.9 (6.3 to 9.9)	19.9 (-6.8 to 55.4)	-19.1 (-36.6 to 3.3)	16 (12 to 21)	3.4 (2.6 to 4.4)	-6.2 (-27.2 to 20.8)	-43.0 (-56.4 to -27.0)
North Macedonia	68 (52 to 91)	6.6 (5.2 to 8.5)	-54.6 (-67.7 to -35.5)	-63.3 (-72.9 to -49.6)	46 (34 to 70)	4.1 (3.2 to 5.9)	-63.5 (-74.1 to -41.8)	-73.1 (-80.2 to -60.6)
Poland	3,990 (3,130 to 4,980)	15.3 (12.0 to 19.0)	47.3 (16.8 to 81.7)	-14.2 (-31.6 to 5.0)	2,480 (1,870 to 3,200)	5.5 (4.2 to 7.1)	29.3 (4.2 to 60.9)	-36.3 (-48.9 to -20.4)
Romania	2,380 (1,910 to 2,900)	19.0 (15.7 to 22.8)	-43.2 (-53.9 to -31.7)	-56.5 (-64.2 to -48.4)	1,200 (945 to 1,490)	8.2 (6.8 to 9.8)	-57.0 (-65.0 to -48.0)	-72.1 (-76.9 to -66.3)
Serbia	586 (443 to 746)	9.4 (7.2 to 11.8)	11.7 (-20.4 to 49.0)	-31.0 (-50.7 to -8.0)	355 (267 to 462)	4.7 (3.5 to 6.0)	-11.6 (-35.1 to 18.1)	-49.2 (-62.9 to -31.4)

	Male				Female			
	2019		Percent change from 1990 to 2019		2019		Percent change from 1990 to 2019	
	Number of attributable deaths	Attributable mortality rate (per 100,000)	Number of attributable deaths	Attributable mortality rate	Number of attributable deaths	Attributable mortality rate (per 100,000)	Number of attributable deaths	Attributable mortality rate
Slovakia	584 (440 to 744)	18.0 (13.6 to 22.9)	-44.1 (-57.8 to -25.8)	-61.3 (-70.4 to -49.1)	356 (257 to 478)	6.9 (5.0 to 9.1)	-45.2 (-59.5 to -16.2)	-66.9 (-75.3 to -50.3)
Slovenia	172 (131 to 227)	10.2 (7.8 to 13.3)	4.1 (-24.4 to 44.0)	-50.6 (-63.5 to -32.5)	152 (112 to 203)	4.2 (3.1 to 5.6)	3.2 (-26.3 to 38.8)	-59.8 (-70.7 to -45.9)
Eastern Europe	19,700 (16,200 to 23,300)	16.3 (13.5 to 19.2)	49.7 (28.0 to 73.9)	8.5 (-6.5 to 24.6)	6,320 (4,520 to 8,040)	4.1 (3.1 to 5.1)	-11.0 (-26.3 to 4.5)	-38.8 (-48.8 to -29.0)
Belarus	470 (357 to 610)	8.7 (6.7 to 11.1)	-6.7 (-28.1 to 19.8)	-30.1 (-45.1 to -11.3)	174 (126 to 232)	1.9 (1.4 to 2.5)	-46.0 (-57.9 to -31.0)	-67.5 (-75.2 to -58.2)
Estonia	77 (57 to 100)	8.8 (6.6 to 11.4)	-11.7 (-33.6 to 11.7)	-30.8 (-47.2 to -12.5)	34 (22 to 50)	2.3 (1.6 to 3.2)	-19.6 (-41.9 to 3.7)	-49.2 (-62.0 to -35.6)
Latvia	121 (90 to 156)	9.7 (7.3 to 12.6)	-21.3 (-39.8 to -0.1)	-23.4 (-40.6 to -4.6)	58 (40 to 82)	2.8 (2.0 to 3.9)	-26.4 (-44.9 to -4.3)	-39.2 (-54.2 to -20.4)
Lithuania	189 (147 to 235)	10.1 (7.9 to 12.4)	36.8 (10.3 to 69.5)	17.4 (-4.5 to 43.9)	81 (57 to 106)	2.7 (2.1 to 3.5)	4.0 (-18.6 to 26.9)	-22.9 (-38.5 to -5.4)
Republic of Moldova	430 (358 to 500)	20.8 (17.5 to 24.1)	-25.5 (-38.4 to -10.2)	-31.8 (-42.5 to -19.0)	122 (97 to 147)	6.2 (5.1 to 7.4)	-66.0 (-72.4 to -58.5)	-65.5 (-72.1 to -57.7)

	Male				Female			
	2019		Percent change from 1990 to 2019		2019		Percent change from 1990 to 2019	
	Number of attributable deaths	Attributable mortality rate (per 100,000)	Number of attributable deaths	Attributable mortality rate	Number of attributable deaths	Attributable mortality rate (per 100,000)	Number of attributable deaths	Attributable mortality rate
Russian Federation	14,400 (11,300 to 17,700)	17.2 (13.5 to 21.0)	54.5 (27.4 to 85.5)	6.5 (-11.5 to 27.2)	4,660 (3,010 to 6,200)	4.4 (3.1 to 5.6)	-4.4 (-25.9 to 15.6)	-39.5 (-52.1 to -27.2)
Ukraine	3,960 (3,060 to 5,080)	15.6 (12.3 to 19.7)	68.9 (34.0 to 116.9)	39.8 (11.3 to 77.0)	1,200 (915 to 1,520)	3.8 (2.9 to 4.7)	-11.3 (-29.5 to 11.0)	-18.4 (-34.8 to 2.3)
**High-income**	**106,000 (90,900 to 119,000)**	**11.0 (9.4 to 12.3)**	**23.6 (14.5 to 30.7)**	**-46.0 (-49.5 to -43.2)**	**81,000 (64,100 to 95,800)**	**5.2 (4.3 to 6.0)**	**19.6 (7.3 to 29.4)**	**-44.6 (-48.7 to -41.0)**
Australasia	868 (706 to 1,020)	3.9 (3.2 to 4.5)	23.2 (7.1 to 38.8)	-56.7 (-61.9 to -51.3)	856 (654 to 1,040)	2.5 (2.0 to 3.0)	37.2 (17.5 to 56.6)	-48.5 (-55.0 to -42.0)
Australia	702 (566 to 832)	3.7 (3.0 to 4.4)	50.4 (26.8 to 72.3)	-45.8 (-53.3 to -38.1)	682 (517 to 838)	2.4 (1.9 to 2.9)	87.2 (56.1 to 118.9)	-30.7 (-40.9 to -19.8)
New Zealand	166 (130 to 204)	4.7 (3.7 to 5.8)	-30.1 (-41.2 to -18.9)	-74.5 (-78.2 to -70.7)	174 (129 to 222)	3.2 (2.5 to 4.1)	-32.9 (-44.7 to -21.5)	-73.0 (-77.1 to -68.7)
High-income Asia Pacific	38,200 (31,500 to 43,700)	17.3 (14.4 to 19.7)	60.7 (41.2 to 75.7)	-54.0 (-58.8 to -49.9)	20,200 (14,400 to 24,900)	4.9 (3.7 to 6.0)	66.5 (30.2 to 95.3)	-59.4 (-66.2 to -53.4)
Brunei Darussalam	17 (13 to 22)	24.2 (17.6 to 32.2)	61.3 (10.7 to 115.7)	-26.8 (-51.0 to -3.2)	7 (5 to 10)	7.1 (4.9 to 9.9)	54.5 (-0.0 to 108.9)	-21.7 (-54.0 to 10.7)

	Male				Female			
	2019		Percent change from 1990 to 2019		2019		Percent change from 1990 to 2019	
	Number of attributable deaths	Attributable mortality rate (per 100,000)	Number of attributable deaths	Attributable mortality rate	Number of attributable deaths	Attributable mortality rate (per 100,000)	Number of attributable deaths	Attributable mortality rate
Japan	32,700 (27,100 to 37,700)	17.5 (14.7 to 20.0)	49.6 (31.0 to 63.9)	-54.8 (-59.5 to -51.0)	16,500 (11,500 to 20,400)	4.8 (3.6 to 5.7)	54.0 (18.3 to 81.6)	-60.8 (-67.2 to -55.4)
Republic of Korea	4,910 (2,720 to 5,800)	17.4 (9.6 to 20.6)	206.1 (42.3 to 268.4)	-16.1 (-64.9 to 1.5)	3,320 (1,730 to 4,250)	6.5 (3.4 to 8.3)	177.3 (39.4 to 255.4)	-32.8 (-67.0 to -14.2)
Singapore	554 (413 to 721)	18.7 (13.9 to 24.6)	86.3 (31.3 to 131.9)	-53.6 (-68.2 to -41.8)	313 (197 to 476)	7.7 (4.9 to 11.6)	78.4 (-4.0 to 155.3)	-57.5 (-77.4 to -39.5)
High-income North America	19,000 (16,400 to 21,600)	6.9 (6.0 to 7.8)	-11.7 (-18.8 to -5.3)	-56.8 (-60.1 to -53.7)	17,400 (14,200 to 20,200)	4.4 (3.7 to 5.1)	-3.1 (-13.0 to 6.1)	-44.6 (-49.8 to -40.2)
Canada	1,850 (1,200 to 2,470)	6.1 (3.9 to 8.1)	-4.4 (-24.1 to 11.3)	-64.4 (-71.1 to -59.0)	1,880 (997 to 2,710)	4.0 (2.3 to 5.7)	18.5 (-10.0 to 39.3)	-51.9 (-62.2 to -44.6)
Greenland	4 (3 to 6)	16.7 (9.4 to 23.4)	-6.8 (-31.8 to 19.5)	-52.3 (-65.2 to -39.4)	3 (1 to 4)	10.1 (5.3 to 14.8)	-27.9 (-50.6 to -4.9)	-59.3 (-71.2 to -46.1)
United States of America	17,100 (14,900 to 19,300)	7.0 (6.1 to 7.9)	-12.5 (-19.2 to -5.8)	-56.0 (-59.4 to -52.8)	15,500 (12,800 to 17,900)	4.4 (3.8 to 5.1)	-5.2 (-14.3 to 4.1)	-44.0 (-49.1 to -39.2)
Southern Latin America	10,300 (8,650 to 11,700)	30.4 (25.6 to 34.6)	87.3 (68.6 to 107.1)	2.3 (-7.7 to 12.2)	9,450 (7,800 to 11,100)	18.5 (15.4 to 21.6)	128.3 (102.1 to 157.5)	7.5 (-4.2 to 20.6)

	Male				Female			
	2019		Percent change from 1990 to 2019		2019		Percent change from 1990 to 2019	
	Number of attributable deaths	Attributable mortality rate (per 100,000)	Number of attributable deaths	Attributable mortality rate	Number of attributable deaths	Attributable mortality rate (per 100,000)	Number of attributable deaths	Attributable mortality rate
Argentina	8,840 (7,430 to 10,100)	40.7 (34.2 to 46.6)	180.9 (147.7 to 215.7)	64.4 (45.7 to 83.4)	8,110 (6,690 to 9,510)	24.2 (20.0 to 28.2)	247.7 (204.3 to 295.0)	74.5 (53.0 to 97.6)
Chile	1,140 (940 to 1,360)	11.9 (9.7 to 14.2)	-46.0 (-53.0 to -38.9)	-76.5 (-79.5 to -73.4)	1,050 (808 to 1,290)	7.8 (6.0 to 9.5)	-35.5 (-45.8 to -25.6)	-77.6 (-81.1 to -74.3)
Uruguay	290 (238 to 337)	13.2 (11.0 to 15.3)	33.4 (17.3 to 51.3)	-10.6 (-21.3 to 1.4)	289 (226 to 349)	7.5 (6.0 to 8.9)	64.7 (41.6 to 94.2)	-14.5 (-26.1 to -0.7)
Western Europe	37,600 (31,900 to 42,600)	8.9 (7.6 to 10.1)	9.8 (0.3 to 17.8)	-49.2 (-53.3 to -45.9)	33,200 (26,400 to 39,500)	4.6 (3.7 to 5.4)	0.7 (-10.5 to 9.8)	-48.6 (-53.6 to -44.3)
Andorra	4 (3 to 6)	6.4 (4.4 to 8.8)	124.4 (49.3 to 227.6)	-41.0 (-59.0 to -17.5)	4 (2 to 6)	4.0 (2.2 to 6.3)	223.2 (94.4 to 380.1)	-38.7 (-61.6 to -10.8)
Austria	324 (268 to 383)	4.1 (3.4 to 4.8)	-15.8 (-26.3 to -4.5)	-59.1 (-64.0 to -54.0)	247 (185 to 313)	1.8 (1.4 to 2.3)	-29.2 (-41.5 to -17.5)	-59.7 (-66.1 to -53.7)
Belgium	1,610 (1,320 to 1,870)	15.1 (12.4 to 17.5)	43.8 (26.3 to 60.6)	-30.7 (-38.5 to -22.6)	1,490 (1,120 to 1,830)	7.9 (6.1 to 9.5)	61.8 (36.9 to 86.6)	-13.8 (-24.8 to -1.8)
Cyprus	39 (31 to 50)	5.6 (4.5 to 7.1)	17.4 (-9.5 to 53.6)	-55.2 (-65.1 to -42.2)	28 (20 to 37)	3.2 (2.3 to 4.3)	-0.5 (-27.2 to 45.4)	-68.8 (-76.8 to -56.2)

	Male				Female			
	2019		Percent change from 1990 to 2019		2019		Percent change from 1990 to 2019	
	**Number of attributable deaths**	**Attributable mortality rate (per 100,000)**	**Number of attributable deaths**	**Attributable mortality rate**	**Number of attributable deaths**	**Attributable mortality rate (per 100,000)**	**Number of attributable deaths**	**Attributable mortality rate**
Denmark	658 (536 to 778)	13.3 (10.7 to 15.7)	18.2 (3.3 to 33.7)	-28.8 (-37.8 to -19.5)	608 (459 to 759)	7.3 (5.6 to 9.1)	6.3 (-9.4 to 23.0)	-30.4 (-39.9 to -19.8)
Finland	155 (105 to 209)	2.9 (2.0 to 3.9)	-72.4 (-77.3 to -68.2)	-88.9 (-90.8 to -87.1)	116 (62 to 180)	1.3 (0.7 to 1.9)	-77.3 (-83.4 to -73.1)	-88.4 (-91.1 to -86.5)
France	5,180 (4,110 to 6,110)	7.9 (6.4 to 9.3)	0.7 (-13.2 to 15.2)	-55.9 (-61.5 to -49.9)	4,860 (3,490 to 6,150)	3.7 (2.8 to 4.6)	14.5 (-6.6 to 36.4)	-48.2 (-56.2 to -39.3)
Germany	7,050 (5,860 to 8,220)	8.2 (6.8 to 9.5)	21.5 (6.1 to 38.2)	-42.2 (-49.0 to -35.1)	5,210 (4,080 to 6,390)	3.7 (3.0 to 4.5)	-4.7 (-17.7 to 8.7)	-40.1 (-47.3 to -32.6)
Greece	1,540 (1,280 to 1,780)	11.8 (10.1 to 13.5)	141.0 (109.1 to 178.0)	10.3 (-3.1 to 26.4)	1,170 (938 to 1,390)	6.8 (5.5 to 7.9)	184.6 (139.7 to 230.9)	25.7 (7.7 to 44.5)
Iceland	23 (15 to 29)	8.4 (5.7 to 10.8)	2.8 (-17.1 to 22.2)	-52.3 (-61.3 to -43.5)	20 (11 to 29)	4.6 (2.8 to 6.7)	-28.5 (-47.6 to -13.6)	-69.5 (-77.2 to -63.3)
Ireland	389 (308 to 466)	12.7 (10.1 to 15.3)	-30.6 (-40.5 to -20.0)	-67.9 (-72.3 to -63.2)	424 (308 to 539)	8.8 (6.5 to 11.1)	-24.3 (-37.3 to -12.4)	-65.0 (-70.6 to -60.0)
Israel	318 (253 to 384)	5.9 (4.8 to 7.2)	83.1 (54.4 to 114.4)	-30.1 (-40.7 to -18.0)	239 (172 to 311)	3.2 (2.3 to 4.1)	85.0 (47.1 to 127.1)	-43.2 (-54.2 to -30.8)

	Male				Female			
	2019		Percent change from 1990 to 2019		2019		Percent change from 1990 to 2019	
	**Number of attributable deaths**	**Attributable mortality rate (per 100,000)**	**Number of attributable deaths**	**Attributable mortality rate**	**Number of attributable deaths**	**Attributable mortality rate (per 100,000)**	**Number of attributable deaths**	**Attributable mortality rate**
Italy	3,020 (2,570 to 3,440)	4.5 (3.9 to 5.1)	10.2 (-2.0 to 20.0)	-51.4 (-56.2 to -47.1)	2,430 (1,860 to 2,880)	2.0 (1.6 to 2.4)	16.7 (-1.9 to 33.9)	-54.7 (-60.7 to -48.5)
Luxembourg	33 (26 to 40)	7.7 (6.1 to 9.5)	22.0 (1.1 to 46.8)	-51.2 (-59.7 to -41.7)	32 (23 to 42)	4.3 (3.2 to 5.4)	39.7 (9.4 to 70.1)	-40.5 (-52.2 to -27.4)
Malta	34 (24 to 43)	8.6 (6.3 to 11.0)	47.1 (19.0 to 77.7)	-42.0 (-52.9 to -30.0)	24 (14 to 35)	4.1 (2.5 to 5.7)	63.7 (14.7 to 109.1)	-40.2 (-56.0 to -24.9)
Monaco	5 (4 to 7)	10.8 (7.9 to 13.9)	49.2 (1.4 to 105.6)	-13.4 (-40.4 to 18.1)	4 (3 to 5)	5.5 (4.0 to 7.3)	42.4 (-1.3 to 100.5)	-7.2 (-34.3 to 29.6)
Netherlands	1,540 (1,240 to 1,830)	10.8 (8.7 to 12.9)	32.4 (15.1 to 50.6)	-38.4 (-46.1 to -30.2)	1,470 (1,110 to 1,850)	6.1 (4.7 to 7.6)	21.5 (3.2 to 39.7)	-33.7 (-43.1 to -24.3)
Norway	348 (244 to 487)	7.8 (5.5 to 11.0)	-44.8 (-54.2 to -36.1)	-66.7 (-72.0 to -61.7)	339 (197 to 521)	4.4 (2.6 to 6.6)	-45.8 (-57.7 to -35.9)	-65.8 (-72.5 to -60.2)
Portugal	1,840 (1,520 to 2,160)	17.1 (14.1 to 19.8)	79.9 (53.8 to 104.3)	-22.6 (-32.9 to -13.1)	1,170 (888 to 1,410)	6.2 (4.8 to 7.4)	98.6 (64.1 to 129.1)	-29.3 (-40.4 to -18.9)
San Marino	2 (1 to 3)	6.2 (4.0 to 8.7)	73.0 (12.4 to 148.1)	-37.1 (-58.9 to -10.5)	1 (1 to 2)	2.2 (1.4 to 3.3)	85.1 (19.6 to 180.3)	-33.2 (-56.3 to 2.7)

	Male				Female			
	2019		Percent change from 1990 to 2019		2019		Percent change from 1990 to 2019	
	Number of attributable deaths	Attributable mortality rate (per 100,000)	Number of attributable deaths	Attributable mortality rate	Number of attributable deaths	Attributable mortality rate (per 100,000)	Number of attributable deaths	Attributable mortality rate
Spain	3,270 (2,740 to 3,840)	7.2 (6.1 to 8.4)	13.1 (-2.9 to 28.8)	-52.4 (-58.6 to -46.1)	2,300 (1,750 to 2,820)	2.9 (2.3 to 3.5)	31.8 (10.0 to 52.9)	-51.0 (-57.7 to -43.7)
Sweden	631 (451 to 798)	6.0 (4.3 to 7.5)	-42.2 (-51.3 to -34.2)	-66.3 (-71.2 to -61.9)	537 (360 to 719)	3.3 (2.3 to 4.4)	-39.4 (-50.0 to -28.7)	-60.5 (-66.8 to -54.0)
Switzerland	451 (354 to 537)	5.4 (4.3 to 6.4)	-37.4 (-46.8 to -28.6)	-71.9 (-75.9 to -68.0)	433 (309 to 565)	3.0 (2.2 to 3.7)	-38.4 (-49.4 to -27.1)	-69.6 (-74.5 to -64.6)
United Kingdom	9,100 (7,500 to 10,700)	15.8 (13.1 to 18.6)	2.3 (-8.2 to 12.3)	-48.2 (-53.2 to -43.2)	9,990 (7,720 to 12,400)	11.0 (8.7 to 13.5)	-15.5 (-25.5 to -5.6)	-43.8 (-49.9 to -37.8)
**Latin America and Caribbean**	**45,100 (37,400 to 53,200)**	**18.1 (15.0 to 21.5)**	**-37.4 (-47.1 to -26.9)**	**-59.4 (-64.8 to -53.4)**	**34,300 (28,000 to 41,300)**	**11.7 (9.6 to 14.1)**	**-41.5 (-52.0 to -30.2)**	**-66.0 (-71.1 to -60.6)**
Andean Latin America	6,800 (5,140 to 8,990)	25.8 (19.7 to 34.1)	-48.2 (-61.3 to -33.3)	-64.8 (-73.0 to -55.1)	5,820 (4,360 to 7,540)	20.1 (15.0 to 26.0)	-51.5 (-63.4 to -37.3)	-70.1 (-76.9 to -62.3)
Bolivia (Plurinational State of)	1,680 (1,270 to 2,150)	38.8 (29.3 to 49.9)	-61.4 (-72.7 to -44.9)	-68.9 (-77.3 to -58.8)	1,560 (1,190 to 2,000)	33.6 (25.3 to 44.5)	-58.2 (-70.0 to -42.7)	-68.0 (-76.3 to -58.2)
Ecuador	1,150 (858 to 1,500)	17.5 (13.0 to 22.7)	-25.9 (-43.0 to -4.1)	-53.6 (-63.7 to -40.7)	823 (610 to 1,090)	11.6 (8.6 to 15.6)	-40.1 (-54.5 to -21.0)	-67.4 (-74.8 to -57.5)

	Male				Female			
	2019		Percent change from 1990 to 2019		2019		Percent change from 1990 to 2019	
	**Number of attributable deaths**	**Attributable mortality rate (per 100,000)**	**Number of attributable deaths**	**Attributable mortality rate**	**Number of attributable deaths**	**Attributable mortality rate (per 100,000)**	**Number of attributable deaths**	**Attributable mortality rate**
Peru	3,970 (2,840 to 5,530)	25.6 (18.3 to 35.7)	-45.1 (-61.7 to -24.4)	-65.4 (-75.4 to -52.6)	3,450 (2,400 to 4,680)	19.7 (13.7 to 26.7)	-50.1 (-64.4 to -30.6)	-71.3 (-79.2 to -61.3)
Caribbean	5,540 (4,430 to 6,810)	25.2 (20.0 to 31.1)	-18.7 (-35.8 to 2.5)	-38.8 (-50.6 to -23.9)	4,260 (3,350 to 5,330)	17.4 (13.6 to 21.9)	-26.0 (-43.5 to -4.7)	-47.0 (-59.2 to -33.1)
Antigua and Barbuda	5 (4 to 7)	14.2 (10.5 to 18.9)	30.7 (0.4 to 65.6)	-20.7 (-38.4 to 1.2)	4 (3 to 6)	9.1 (6.3 to 12.4)	14.8 (-14.9 to 50.9)	-3.7 (-28.8 to 26.4)
Bahamas	18 (14 to 24)	12.8 (9.5 to 17.2)	8.6 (-16.9 to 42.2)	-47.9 (-59.5 to -32.6)	11 (8 to 16)	6.4 (4.6 to 8.9)	1.0 (-26.0 to 36.1)	-45.3 (-58.8 to -27.6)
Barbados	30 (22 to 38)	15.6 (11.7 to 19.9)	60.6 (22.9 to 101.5)	-5.2 (-27.2 to 18.3)	20 (13 to 30)	8.2 (5.5 to 12.1)	63.0 (8.4 to 125.6)	7.0 (-26.7 to 45.8)
Belize	30 (23 to 38)	22.3 (17.1 to 28.5)	30.2 (1.1 to 66.0)	-28.8 (-43.8 to -11.3)	17 (13 to 23)	12.8 (9.1 to 17.2)	-16.5 (-36.6 to 10.0)	-51.2 (-62.4 to -37.3)
Bermuda	3 (2 to 4)	5.3 (3.4 to 7.2)	13.4 (-18.3 to 48.0)	-52.1 (-65.2 to -37.9)	2 (1 to 3)	2.5 (1.2 to 3.8)	-4.4 (-51.6 to 36.9)	-60.8 (-76.7 to -46.4)
Cuba	1,680 (1,290 to 2,110)	19.1 (14.6 to 23.9)	67.7 (34.2 to 104.5)	-10.4 (-28.1 to 9.1)	1,050 (757 to 1,370)	9.6 (7.1 to 12.4)	71.7 (33.6 to 116.6)	-23.9 (-39.9 to -4.3)

	Male				Female			
	2019		Percent change from 1990 to 2019		2019		Percent change from 1990 to 2019	
	**Number of attributable deaths**	**Attributable mortality rate (per 100,000)**	**Number of attributable deaths**	**Attributable mortality rate**	**Number of attributable deaths**	**Attributable mortality rate (per 100,000)**	**Number of attributable deaths**	**Attributable mortality rate**
Dominica	6 (4 to 8)	17.2 (12.3 to 23.2)	-13.3 (-34.7 to 14.9)	-28.4 (-46.0 to -5.3)	4 (3 to 5)	9.3 (6.4 to 12.7)	-36.5 (-54.0 to -12.9)	-33.2 (-51.0 to -9.8)
Dominican Republic	551 (395 to 750)	12.5 (9.0 to 17.0)	-34.4 (-54.7 to -3.1)	-44.7 (-59.6 to -23.5)	419 (299 to 580)	8.9 (6.4 to 12.2)	-48.8 (-64.3 to -24.2)	-60.2 (-71.3 to -45.4)
Grenada	9 (7 to 11)	22.6 (17.5 to 28.8)	-37.6 (-50.8 to -23.5)	-44.6 (-56.2 to -31.7)	8 (5 to 10)	14.8 (10.6 to 20.0)	-45.1 (-59.5 to -26.9)	-44.7 (-58.9 to -27.3)
Guyana	71 (51 to 95)	27.3 (19.9 to 36.3)	-30.1 (-48.8 to -6.1)	-37.0 (-52.4 to -17.5)	44 (29 to 62)	15.5 (10.4 to 21.8)	-32.3 (-51.7 to -6.4)	-38.7 (-55.0 to -16.9)
Haiti	2,530 (1,820 to 3,390)	53.8 (37.1 to 73.5)	-36.7 (-57.0 to -8.8)	-52.1 (-64.7 to -37.1)	2,260 (1,590 to 3,060)	49.1 (33.8 to 67.4)	-37.6 (-56.4 to -11.0)	-51.1 (-64.0 to -35.2)
Jamaica	95 (72 to 124)	7.0 (5.3 to 9.1)	-32.0 (-46.8 to -13.7)	-52.7 (-63.1 to -39.5)	77 (54 to 104)	4.4 (3.1 to 5.9)	-41.0 (-55.7 to -24.0)	-61.8 (-71.3 to -50.7)
Puerto Rico	188 (136 to 252)	6.4 (4.7 to 8.5)	-29.2 (-46.4 to -7.8)	-62.2 (-71.6 to -51.3)	122 (80 to 169)	3.0 (2.1 to 4.1)	-11.4 (-38.7 to 18.3)	-60.5 (-71.6 to -47.5)
Saint Kitts and Nevis	3 (2 to 4)	14.0 (10.2 to 18.7)	-19.9 (-40.9 to 6.8)	-47.7 (-60.1 to -31.4)	2 (1 to 3)	8.0 (5.8 to 10.8)	-44.4 (-58.1 to -28.5)	-56.9 (-67.2 to -45.5)

	Male				Female			
	2019		Percent change from 1990 to 2019		2019		Percent change from 1990 to 2019	
	Number of attributable deaths	Attributable mortality rate (per 100,000)	Number of attributable deaths	Attributable mortality rate	Number of attributable deaths	Attributable mortality rate (per 100,000)	Number of attributable deaths	Attributable mortality rate
Saint Lucia	11 (8 to 14)	13.8 (10.4 to 17.6)	11.8 (-11.9 to 41.4)	-49.2 (-60.2 to -35.8)	7 (5 to 10)	6.7 (4.7 to 9.4)	-17.1 (-39.0 to 11.6)	-57.9 (-69.0 to -43.5)
Saint Vincent and the Grenadines	9 (7 to 11)	15.7 (12.0 to 20.0)	10.9 (-13.4 to 38.4)	-33.7 (-47.8 to -18.5)	5 (4 to 7)	9.4 (6.6 to 12.9)	-26.7 (-45.4 to -3.6)	-42.6 (-57.0 to -25.4)
Suriname	46 (35 to 60)	19.4 (14.7 to 25.3)	13.8 (-13.7 to 48.4)	-34.0 (-49.1 to -15.5)	28 (20 to 38)	9.8 (7.0 to 13.1)	-1.9 (-26.1 to 30.4)	-43.9 (-58.0 to -26.2)
Trinidad and Tobago	63 (45 to 88)	8.8 (6.4 to 12.2)	-35.5 (-53.6 to -13.2)	-67.1 (-76.2 to -56.2)	37 (24 to 53)	4.5 (3.1 to 6.5)	-40.0 (-57.4 to -19.2)	-67.5 (-77.1 to -55.3)
United States Virgin Islands	5 (3 to 6)	7.0 (4.9 to 9.4)	54.2 (4.6 to 118.7)	-21.6 (-47.7 to 11.9)	3 (2 to 3)	2.9 (2.1 to 3.9)	36.3 (-6.3 to 86.8)	-37.5 (-57.1 to -14.4)
Central Latin America	13,400 (10,700 to 16,500)	12.7 (10.2 to 15.7)	-44.0 (-54.6 to -30.9)	-62.9 (-69.6 to -54.7)	9,270 (7,350 to 11,800)	7.8 (6.2 to 10.0)	-53.2 (-62.6 to -41.7)	-71.2 (-76.9 to -64.6)
Colombia	1,650 (1,210 to 2,220)	7.2 (5.2 to 9.6)	-42.7 (-58.8 to -22.4)	-65.4 (-74.3 to -53.5)	1,390 (1,010 to 1,860)	5.2 (3.8 to 7.1)	-40.2 (-56.2 to -19.8)	-69.7 (-78.0 to -59.7)
Costa Rica	154 (113 to 206)	6.8 (5.0 to 9.1)	8.3 (-20.4 to 47.8)	-48.2 (-60.8 to -31.4)	80 (57 to 110)	2.9 (2.1 to 4.0)	-19.4 (-39.9 to 8.5)	-66.7 (-74.7 to -56.1)

	Male				Female			
	2019		Percent change from 1990 to 2019		2019		Percent change from 1990 to 2019	
	Number of attributable deaths	Attributable mortality rate (per 100,000)	Number of attributable deaths	Attributable mortality rate	Number of attributable deaths	Attributable mortality rate (per 100,000)	Number of attributable deaths	Attributable mortality rate
El Salvador	445 (310 to 618)	16.7 (11.6 to 23.1)	-35.2 (-56.1 to -8.5)	-46.4 (-62.6 to -26.3)	341 (220 to 488)	9.3 (6.0 to 13.4)	-47.3 (-65.9 to -24.3)	-65.6 (-76.4 to -52.3)
Guatemala	2,890 (2,170 to 3,790)	54.4 (41.6 to 70.4)	-36.3 (-53.3 to -14.9)	-64.2 (-72.8 to -53.3)	2,330 (1,740 to 3,030)	36.0 (27.0 to 47.1)	-42.6 (-56.8 to -24.0)	-74.9 (-80.9 to -67.7)
Honduras	416 (318 to 536)	14.1 (10.9 to 18.4)	-37.1 (-55.0 to -12.2)	-48.3 (-60.7 to -31.2)	195 (130 to 375)	6.3 (4.2 to 11.8)	-65.5 (-77.9 to -31.2)	-70.7 (-80.3 to -45.6)
Mexico	5,990 (4,820 to 7,380)	11.6 (9.4 to 14.3)	-52.1 (-62.5 to -40.4)	-65.8 (-72.3 to -59.1)	3,750 (3,060 to 4,610)	6.7 (5.5 to 8.3)	-63.6 (-71.0 to -54.4)	-75.5 (-79.7 to -70.4)
Nicaragua	322 (253 to 399)	15.3 (11.9 to 19.0)	-64.8 (-74.2 to -50.6)	-62.6 (-71.0 to -51.3)	221 (168 to 282)	9.0 (6.8 to 11.7)	-68.4 (-78.1 to -56.4)	-65.6 (-74.9 to -53.9)
Panama	176 (128 to 237)	8.9 (6.5 to 12.1)	47.2 (9.6 to 98.0)	-31.0 (-49.4 to -8.9)	108 (75 to 147)	5.0 (3.5 to 6.9)	28.1 (-6.1 to 74.4)	-40.4 (-56.1 to -20.3)
Venezuela (Bolivarian Republic of)	1,320 (948 to 1,780)	10.8 (7.7 to 14.6)	-6.7 (-32.8 to 24.2)	-48.2 (-61.7 to -32.2)	858 (600 to 1,220)	6.1 (4.3 to 8.6)	-17.1 (-41.7 to 13.2)	-54.8 (-68.2 to -38.3)
Tropical Latin America	19,400 (16,400 to 22,400)	20.2 (16.9 to 23.4)	-31.3 (-42.8 to -20.4)	-59.6 (-64.7 to -54.5)	15,000 (11,900 to 18,100)	12.2 (9.9 to 14.6)	-28.9 (-45.1 to -13.6)	-64.6 (-71.0 to -58.4)

	Male				Female			
	2019		Percent change from 1990 to 2019		2019		Percent change from 1990 to 2019	
	Number of attributable deaths	Attributable mortality rate (per 100,000)	Number of attributable deaths	Attributable mortality rate	Number of attributable deaths	Attributable mortality rate (per 100,000)	Number of attributable deaths	Attributable mortality rate
Brazil	19,000 (16,000 to 21,900)	20.2 (17.1 to 23.4)	-31.8 (-43.4 to -20.8)	-60.1 (-65.2 to -55.1)	14,700 (11,700 to 17,700)	12.3 (9.9 to 14.7)	-29.1 (-45.4 to -13.4)	-65.0 (-71.4 to -58.8)
Paraguay	431 (318 to 571)	17.6 (13.0 to 23.3)	7.4 (-22.0 to 43.4)	-30.9 (-48.7 to -9.9)	308 (217 to 416)	10.3 (7.3 to 13.9)	-18.1 (-40.8 to 9.2)	-50.6 (-63.4 to -34.7)
**North Africa and Middle East**	**39,700 (33,400 to 46,800)**	**18.3 (15.5 to 21.4)**	**-54.1 (-65.7 to -42.3)**	**-59.2 (-66.4 to -52.1)**	**30,500 (25,200 to 36,100)**	**13.9 (11.6 to 16.4)**	**-62.9 (-71.5 to -53.5)**	**-65.1 (-72.1 to -58.3)**
Afghanistan	8,090 (5,740 to 10,900)	50.8 (40.2 to 62.8)	-29.3 (-53.8 to 8.2)	-62.0 (-71.9 to -48.7)	8,340 (5,700 to 11,200)	44.6 (33.4 to 55.7)	-21.6 (-49.0 to 14.8)	-63.4 (-74.9 to -50.5)
Algeria	1,810 (1,400 to 2,330)	13.1 (10.0 to 17.2)	-46.2 (-67.9 to -16.4)	-63.1 (-73.0 to -50.7)	1,410 (1,070 to 1,840)	11.9 (8.8 to 16.1)	-51.4 (-69.9 to -20.9)	-59.6 (-70.8 to -44.7)
Bahrain	29 (22 to 37)	13.4 (9.6 to 17.2)	92.4 (37.9 to 154.9)	-41.1 (-56.5 to -24.6)	18 (13 to 23)	10.0 (6.7 to 13.1)	69.3 (24.9 to 128.2)	-29.5 (-47.3 to -8.1)
Egypt	8,410 (6,140 to 11,400)	22.6 (16.7 to 30.8)	-64.1 (-74.9 to -49.1)	-66.1 (-75.4 to -53.4)	5,740 (4,130 to 7,920)	19.4 (13.7 to 28.2)	-76.0 (-83.4 to -64.9)	-70.9 (-79.7 to -58.3)
Iran (Islamic Republic of)	3,170 (2,730 to 3,610)	9.7 (8.3 to 11.0)	-51.7 (-64.9 to -36.4)	-60.4 (-67.8 to -53.1)	2,070 (1,690 to 2,410)	6.9 (5.6 to 8.0)	-65.1 (-75.8 to -52.7)	-65.6 (-74.2 to -57.9)

	Male				Female			
	2019		Percent change from 1990 to 2019		2019		Percent change from 1990 to 2019	
	Number of attributable deaths	Attributable mortality rate (per 100,000)	Number of attributable deaths	Attributable mortality rate	Number of attributable deaths	Attributable mortality rate (per 100,000)	Number of attributable deaths	Attributable mortality rate
Iraq	1,200 (939 to 1,530)	9.9 (8.0 to 12.4)	-69.6 (-79.6 to -56.3)	-71.2 (-78.7 to -60.4)	913 (689 to 1,180)	6.2 (4.8 to 8.1)	-60.3 (-75.3 to -39.4)	-68.9 (-78.4 to -55.8)
Jordan	370 (293 to 460)	11.3 (8.8 to 14.0)	18.1 (-15.4 to 64.7)	-49.5 (-61.6 to -35.6)	261 (194 to 347)	8.4 (6.2 to 10.9)	-1.6 (-34.1 to 47.3)	-53.0 (-65.3 to -36.8)
Kuwait	282 (216 to 351)	25.4 (19.3 to 31.7)	292.6 (208.4 to 389.5)	32.0 (5.8 to 60.4)	115 (87 to 146)	14.1 (10.4 to 18.1)	94.4 (53.8 to 143.1)	-15.7 (-32.6 to 8.5)
Lebanon	337 (262 to 483)	15.1 (11.7 to 21.8)	41.6 (4.5 to 109.4)	-29.0 (-46.7 to 4.0)	202 (150 to 299)	7.3 (5.4 to 10.7)	-1.8 (-32.3 to 43.8)	-48.5 (-61.9 to -25.7)
Libya	266 (199 to 346)	11.7 (8.7 to 15.2)	-24.1 (-48.5 to 10.2)	-41.4 (-57.2 to -21.1)	171 (127 to 232)	7.6 (5.6 to 10.3)	-37.2 (-59.3 to -3.2)	-45.9 (-61.6 to -23.3)
Morocco	1,890 (1,400 to 2,490)	14.8 (10.9 to 19.8)	-63.9 (-75.5 to -48.0)	-62.1 (-72.2 to -49.7)	1,350 (963 to 1,810)	10.2 (7.2 to 13.7)	-72.9 (-82.2 to -59.7)	-70.5 (-79.9 to -59.6)
Oman	115 (81 to 151)	22.3 (14.8 to 30.7)	-27.6 (-51.0 to 0.9)	-47.4 (-62.9 to -25.6)	79 (52 to 106)	14.1 (8.7 to 20.5)	-51.2 (-67.8 to -28.0)	-53.9 (-69.9 to -30.8)
Palestine	161 (131 to 202)	18.7 (15.0 to 24.6)	-13.6 (-36.9 to 14.6)	-38.3 (-51.8 to -19.2)	98 (75 to 142)	8.6 (6.4 to 12.8)	-36.9 (-59.5 to -7.9)	-50.9 (-63.3 to -35.6)

	Male				Female			
	2019		Percent change from 1990 to 2019		2019		Percent change from 1990 to 2019	
	Number of attributable deaths	Attributable mortality rate (per 100,000)	Number of attributable deaths	Attributable mortality rate	Number of attributable deaths	Attributable mortality rate (per 100,000)	Number of attributable deaths	Attributable mortality rate
Qatar	28 (20 to 39)	12.1 (8.7 to 16.7)	191.5 (96.7 to 329.3)	-22.6 (-45.2 to 9.4)	13 (9 to 18)	18.3 (12.6 to 24.8)	59.9 (9.1 to 127.6)	16.5 (-21.8 to 62.7)
Saudi Arabia	1,620 (1,200 to 2,080)	18.6 (14.5 to 23.4)	58.8 (17.1 to 125.3)	-34.7 (-50.2 to -3.2)	967 (678 to 1,300)	14.7 (10.7 to 19.5)	9.9 (-20.7 to 53.1)	-40.8 (-57.3 to -13.8)
Sudan	3,080 (2,070 to 4,440)	22.8 (15.7 to 31.2)	-64.7 (-80.9 to -39.1)	-64.7 (-78.8 to -49.1)	2,250 (1,510 to 3,200)	17.0 (12.1 to 22.9)	-71.4 (-84.2 to -48.3)	-71.0 (-81.9 to -56.1)
Syrian Arab Republic	787 (568 to 1,060)	14.7 (10.8 to 19.4)	-50.9 (-68.0 to -21.9)	-33.0 (-52.9 to -3.1)	455 (331 to 605)	10.3 (7.7 to 13.4)	-64.3 (-76.9 to -45.9)	-37.9 (-55.7 to -14.1)
Tunisia	619 (448 to 861)	12.6 (9.2 to 17.6)	-32.2 (-56.9 to 6.8)	-51.0 (-66.0 to -29.7)	332 (232 to 464)	6.0 (4.3 to 8.3)	-63.0 (-77.1 to -40.3)	-72.1 (-81.3 to -59.5)
Turkey	4,740 (3,540 to 5,910)	13.4 (10.1 to 16.6)	-65.4 (-76.7 to -50.1)	-69.5 (-78.0 to -58.9)	3,220 (2,250 to 4,080)	7.6 (5.6 to 9.6)	-75.6 (-83.9 to -63.7)	-80.7 (-86.7 to -72.6)
United Arab Emirates	261 (170 to 365)	27.7 (15.3 to 37.6)	302.8 (174.4 to 482.0)	-39.7 (-58.4 to -11.5)	57 (36 to 82)	18.2 (7.4 to 27.3)	41.4 (-11.5 to 109.4)	-50.4 (-69.8 to -29.4)
Yemen	2,400 (1,540 to 3,590)	26.8 (17.4 to 40.2)	-52.2 (-75.2 to -15.5)	-55.1 (-73.1 to -32.9)	2,420 (1,630 to 3,490)	21.4 (15.0 to 29.7)	-60.9 (-75.7 to -37.0)	-65.0 (-76.8 to -47.9)

	Male				Female			
	2019		Percent change from 1990 to 2019		2019		Percent change from 1990 to 2019	
	Number of attributable deaths	Attributable mortality rate (per 100,000)	Number of attributable deaths	Attributable mortality rate	Number of attributable deaths	Attributable mortality rate (per 100,000)	Number of attributable deaths	Attributable mortality rate
**South Asia**	**199,000 (170,000 to 230,000)**	**30.7 (26.0 to 35.2)**	**-54.1 (-61.8 to -43.4)**	**-58.9 (-64.7 to -50.9)**	**208,000 (175,000 to 243,000)**	**30.6 (25.5 to 36.0)**	**-48.6 (-58.7 to -37.1)**	**-56.7 (-64.7 to -47.5**
Bangladesh	14,300 (11,400 to 18,400)	23.4 (18.8 to 30.2)	-74.3 (-80.8 to -65.0)	-72.2 (-78.3 to -63.4)	14,000 (10,400 to 17,400)	24.1 (17.6 to 30.0)	-72.5 (-80.3 to -63.1)	-67.2 (-75.8 to -58.1)
Bhutan	56 (35 to 85)	20.8 (12.8 to 30.8)	-78.2 (-87.8 to -53.2)	-68.7 (-81.1 to -44.4)	51 (32 to 74)	19.5 (12.2 to 28.1)	-73.3 (-84.9 to -40.0)	-63.7 (-78.1 to -33.6)
India	151,000 (127,000 to 176,000)	30.8 (25.9 to 35.6)	-53.4 (-62.0 to -41.2)	-59.8 (-66.1 to -51.1)	163,000 (134,000 to 194,000)	31.3 (25.7 to 37.2)	-46.8 (-58.2 to -33.0)	-58.6 (-67.1 to -48.3)
Nepal	3,320 (2,490 to 4,290)	31.1 (22.4 to 41.0)	-75.9 (-83.0 to -65.6)	-68.2 (-78.6 to -54.8)	3,630 (2,840 to 4,510)	32.7 (24.8 to 41.2)	-72.4 (-79.4 to -63.0)	-66.4 (-76.0 to -54.2)
Pakistan	30,400 (22,400 to 40,200)	34.1 (23.9 to 45.3)	-24.7 (-44.1 to 3.4)	-38.4 (-52.7 to -20.2)	27,600 (20,800 to 35,800)	23.3 (18.2 to 29.2)	-20.3 (-43.9 to 12.0)	-42.1 (-57.3 to -22.0)
**Southeast Asia, East Asia, and Oceania**	**151,000 (131,000 to 174,000)**	**18.4 (15.8 to 21.0)**	**-60.5 (-67.0 to -52.4)**	**-67.3 (-71.9 to -62.0)**	**99,500 (81,900 to 120,000)**	**10.0 (8.4 to 11.8)**	**-70.7 (-76.1 to -64.0)**	**-79.2 (-82.4 to -75.5)**
East Asia	76,500 (64,600 to 90,500)	13.5 (11.5 to 15.6)	-69.7 (-75.3 to -58.5)	-75.3 (-79.2 to -68.4)	48,600 (39,200 to 61,400)	6.3 (5.3 to 7.8)	-79.1 (-83.5 to -71.4)	-87.0 (-89.4 to -82.7)

	Male				Female			
	2019		Percent change from 1990 to 2019		2019		Percent change from 1990 to 2019	
	**Number of attributable deaths**	**Attributable mortality rate (per 100,000)**	**Number of attributable deaths**	**Attributable mortality rate**	**Number of attributable deaths**	**Attributable mortality rate (per 100,000)**	**Number of attributable deaths**	**Attributable mortality rate**
China	70,600 (58,800 to 84,700)	13.0 (11.0 to 15.3)	-71.2 (-76.7 to -60.0)	-76.4 (-80.4 to -69.7)	44,500 (35,800 to 56,900)	6.1 (5.0 to 7.6)	-80.3 (-84.6 to -72.7)	-87.6 (-89.9 to -83.3)
Democratic People's Republic of Korea	2,120 (1,660 to 2,620)	24.8 (19.0 to 30.8)	-62.9 (-75.0 to -46.6)	-55.0 (-67.6 to -40.0)	2,640 (2,010 to 3,460)	16.4 (12.6 to 21.3)	-55.5 (-70.1 to -34.1)	-66.1 (-75.6 to -53.5)
Taiwan (Province of China)	3,780 (2,880 to 4,820)	20.9 (15.8 to 26.8)	153.2 (98.9 to 219.3)	-14.8 (-33.3 to 7.4)	1,480 (1,060 to 1,980)	6.7 (4.9 to 8.9)	121.3 (70.6 to 186.3)	-41.9 (-55.0 to -25.6)
Oceania	3,460 (2,550 to 4,710)	52.9 (40.4 to 74.0)	23.1 (-8.8 to 66.2)	-36.9 (-50.4 to -20.0)	2,900 (2,110 to 3,840)	46.2 (35.3 to 60.3)	34.3 (-3.0 to 81.3)	-30.1 (-46.6 to -11.3)
American Samoa	2 (2 to 3)	11.8 (8.8 to 16.0)	-17.2 (-41.4 to 11.9)	-42.7 (-58.7 to -25.7)	2 (1 to 3)	7.9 (5.4 to 11.1)	-24.2 (-50.1 to 10.8)	-44.5 (-64.4 to -22.1)
Cook Islands	2 (2 to 3)	21.3 (15.3 to 28.0)	-26.1 (-44.8 to -2.6)	-50.2 (-62.7 to -36.1)	1 (1 to 1)	7.5 (4.9 to 10.4)	-51.5 (-67.0 to -31.7)	-69.2 (-78.7 to -57.5)
Fiji	58 (41 to 78)	18.3 (13.4 to 24.5)	-21.4 (-45.7 to 13.7)	-45.7 (-61.0 to -24.5)	37 (26 to 51)	10.1 (7.2 to 13.9)	-27.3 (-50.8 to 5.4)	-51.2 (-66.4 to -29.8)
Guam	7 (5 to 9)	8.2 (5.8 to 11.1)	30.6 (-9.1 to 72.6)	-36.1 (-57.8 to -15.3)	5 (4 to 7)	5.5 (3.9 to 7.8)	13.6 (-24.2 to 54.0)	-47.5 (-68.1 to -26.8)

	Male				Female			
	2019		Percent change from 1990 to 2019		2019		Percent change from 1990 to 2019	
	**Number of attributable deaths**	**Attributable mortality rate (per 100,000)**	**Number of attributable deaths**	**Attributable mortality rate**	**Number of attributable deaths**	**Attributable mortality rate (per 100,000)**	**Number of attributable deaths**	**Attributable mortality rate**
Kiribati	12<br>(9 to 16)	38.6<br>(29.6 to 47.9)	-28.2<br>(-48.4 to -2.7)	-37.8<br>(-51.8 to -21.3)	11<br>(8 to 14)	29.8<br>(21.9 to 39.0)	-31.0<br>(-52.5 to -3.7)	-40.2<br>(-54.5 to -20.9)
Marshall Islands	5<br>(4 to 8)	28.5<br>(20.1 to 39.4)	-32.9<br>(-51.6 to -9.8)	-49.7<br>(-64.2 to -33.5)	4<br>(3 to 5)	21.7<br>(14.6 to 32.7)	-38.0<br>(-57.3 to -13.1)	-45.1<br>(-62.5 to -23.6)
Micronesia (Federated States of)	10<br>(7 to 14)	32.2<br>(22.4 to 43.8)	-57.3<br>(-70.6 to -41.4)	-49.8<br>(-63.9 to -33.5)	7<br>(5 to 10)	21.8<br>(14.6 to 32.1)	-63.4<br>(-75.6 to -48.3)	-57.3<br>(-71.0 to -40.0)
Nauru	1<br>(1 to 2)	33.5<br>(23.0 to 49.5)	-45.5<br>(-59.5 to -28.7)	-31.4<br>(-50.7 to -10.9)	1<br>(1 to 1)	21.6<br>(14.4 to 34.5)	-50.1<br>(-63.7 to -32.7)	-42.3<br>(-60.0 to -22.7)
Niue	0<br>(0 to 0)	20.6<br>(15.1 to 27.3)	-50.9<br>(-63.5 to -35.2)	-39.6<br>(-54.7 to -20.9)	0<br>(0 to 0)	11.8<br>(8.4 to 15.8)	-62.1<br>(-72.4 to -47.2)	-44.1<br>(-58.9 to -23.1)
Northern Mariana Islands	3<br>(2 to 4)	14.4<br>(10.8 to 19.0)	6.5<br>(-22.5 to 39.3)	-34.5<br>(-52.8 to -17.4)	1<br>(1 to 2)	6.1<br>(4.3 to 8.9)	-12.6<br>(-38.5 to 16.5)	-48.5<br>(-67.0 to -30.2)
Palau	4<br>(2 to 5)	41.1<br>(28.9 to 62.4)	-11.1<br>(-40.1 to 23.5)	-42.0<br>(-60.9 to -22.2)	2<br>(1 to 4)	29.5<br>(19.3 to 49.9)	-36.6<br>(-61.1 to -9.6)	-47.0<br>(-68.6 to -27.7)
Papua New Guinea	2,940<br>(2,090 to 4,040)	56.0<br>(41.9 to 85.4)	31.6<br>(-5.7 to 81.7)	-38.4<br>(-53.4 to -18.4)	2,510<br>(1,790 to 3,350)	52.5<br>(39.3 to 70.8)	44.3<br>(0.9 to 98.9)	-30.9<br>(-48.5 to -9.8)

	Male				Female			
	2019		Percent change from 1990 to 2019		2019		Percent change from 1990 to 2019	
	Number of attributable deaths	Attributable mortality rate (per 100,000)	Number of attributable deaths	Attributable mortality rate	Number of attributable deaths	Attributable mortality rate (per 100,000)	Number of attributable deaths	Attributable mortality rate
Samoa	18 (13 to 23)	26.4 (20.0 to 34.0)	-26.7 (-47.9 to 0.4)	-45.7 (-59.0 to -30.1)	15 (10 to 21)	20.8 (14.7 to 29.4)	-22.4 (-47.5 to 9.4)	-41.9 (-59.6 to -20.4)
Solomon Islands	190 (147 to 234)	106.1 (81.1 to 131.5)	-10.4 (-30.6 to 17.2)	-48.6 (-60.6 to -28.6)	137 (107 to 172)	87.3 (65.3 to 109.3)	-3.3 (-27.0 to 29.4)	-44.3 (-58.4 to -24.3)
Tokelau	0 (0 to 0)	12.5 (8.6 to 18.1)	-64.1 (-75.0 to -49.5)	-52.9 (-66.3 to -37.4)	0 (0 to 0)	11.1 (7.1 to 16.9)	-72.4 (-82.6 to -60.5)	-59.0 (-72.7 to -42.2)
Tonga	9 (7 to 12)	26.5 (19.9 to 34.6)	-20.8 (-39.2 to 4.8)	-35.9 (-50.5 to -17.6)	6 (4 to 8)	12.3 (8.5 to 17.1)	-29.4 (-49.8 to -1.2)	-51.0 (-65.3 to -32.8)
Tuvalu	1 (1 to 1)	22.4 (15.4 to 31.9)	-71.4 (-81.8 to -57.6)	-70.9 (-80.7 to -58.7)	1 (0 to 1)	15.2 (9.7 to 22.9)	-79.6 (-87.1 to -67.8)	-78.5 (-86.1 to -67.7)
Vanuatu	41 (30 to 54)	40.3 (29.5 to 53.8)	33.2 (-0.0 to 83.2)	-26.4 (-43.5 to -3.1)	30 (22 to 41)	31.9 (22.5 to 44.6)	26.9 (-10.2 to 79.5)	-31.5 (-51.8 to -3.3)
Southeast Asia	71,500 (59,100 to 83,300)	31.4 (25.6 to 36.8)	-44.3 (-56.0 to -30.6)	-50.1 (-58.1 to -40.0)	48,000 (36,700 to 58,500)	18.0 (13.8 to 21.9)	-54.0 (-64.8 to -39.7)	-61.8 (-70.1 to -52.2)
Cambodia	4,630 (3,760 to 5,620)	95.2 (77.5 to 115.4)	-62.1 (-70.2 to -51.6)	-53.2 (-62.6 to -42.9)	4,000 (3,130 to 4,870)	66.2 (50.4 to 81.9)	-62.1 (-70.8 to -50.6)	-60.4 (-69.0 to -50.4)

	Male				Female			
	2019		Percent change from 1990 to 2019		2019		Percent change from 1990 to 2019	
	Number of attributable deaths	Attributable mortality rate (per 100,000)	Number of attributable deaths	Attributable mortality rate	Number of attributable deaths	Attributable mortality rate (per 100,000)	Number of attributable deaths	Attributable mortality rate
Indonesia	14,000 (11,100 to 16,900)	18.4 (14.6 to 22.3)	-68.1 (-75.4 to -57.4)	-65.0 (-72.0 to -55.0)	9,590 (7,430 to 11,900)	11.2 (8.6 to 14.0)	-71.9 (-80.0 to -62.2)	-70.4 (-78.0 to -61.9)
Lao People's Democratic Republic	1,430 (1,050 to 1,880)	60.9 (45.4 to 77.2)	-71.4 (-79.6 to -60.2)	-67.4 (-75.3 to -57.4)	1,130 (798 to 1,520)	45.6 (33.1 to 59.7)	-72.5 (-80.9 to -61.4)	-69.7 (-78.3 to -59.4)
Malaysia	5,250 (3,290 to 7,000)	46.0 (28.7 to 61.1)	209.4 (56.1 to 308.6)	32.8 (-36.1 to 76.1)	2,260 (1,180 to 3,230)	21.2 (11.3 to 30.4)	129.7 (13.0 to 231.2)	17.8 (-44.9 to 72.1)
Maldives	9 (7 to 12)	6.5 (4.6 to 8.4)	-44.4 (-65.1 to -15.4)	-66.1 (-75.5 to -53.6)	6 (4 to 9)	4.8 (3.4 to 6.7)	-65.9 (-79.1 to -43.5)	-79.0 (-85.7 to -69.1)
Mauritius	66 (48 to 88)	10.8 (7.9 to 14.3)	-31.7 (-46.1 to -14.1)	-68.3 (-74.9 to -60.7)	28 (15 to 42)	3.8 (2.3 to 5.4)	-38.2 (-59.0 to -20.4)	-69.3 (-77.5 to -60.9)
Myanmar	8,240 (6,140 to 10,600)	43.4 (33.2 to 54.1)	-67.7 (-80.2 to -47.1)	-65.1 (-75.1 to -49.3)	6,410 (4,660 to 8,330)	28.1 (20.5 to 35.8)	-74.9 (-83.9 to -57.1)	-73.6 (-82.1 to -60.0)
Philippines	19,500 (15,300 to 24,200)	60.2 (44.9 to 76.9)	-0.8 (-23.2 to 25.4)	-20.5 (-39.2 to -1.7)	14,400 (10,200 to 19,000)	41.3 (26.7 to 56.1)	-4.1 (-30.1 to 26.6)	-34.2 (-58.4 to -16.6)
Seychelles	22 (16 to 27)	53.1 (38.9 to 65.6)	52.8 (21.4 to 90.9)	-15.3 (-32.3 to 3.7)	9 (6 to 12)	16.8 (11.5 to 22.6)	36.5 (6.3 to 69.6)	-7.2 (-26.9 to 16.1)

	Male				Female			
	2019		Percent change from 1990 to 2019		2019		Percent change from 1990 to 2019	
	Number of attributable deaths	Attributable mortality rate (per 100,000)	Number of attributable deaths	Attributable mortality rate	Number of attributable deaths	Attributable mortality rate (per 100,000)	Number of attributable deaths	Attributable mortality rate
Sri Lanka	1,200 (795 to 1,720)	13.1 (8.7 to 18.6)	-38.9 (-58.5 to -12.9)	-68.9 (-78.4 to -55.4)	690 (432 to 1,100)	6.0 (3.8 to 9.6)	-47.7 (-66.2 to -14.9)	-78.9 (-86.2 to -65.9)
Thailand	9,320 (5,520 to 12,900)	22.9 (14.2 to 31.3)	96.5 (-9.9 to 187.3)	-13.7 (-62.3 to 24.9)	4,990 (2,680 to 7,290)	9.8 (5.5 to 14.0)	58.8 (-34.5 to 141.4)	-40.0 (-75.0 to -9.5)
Timor-Leste	231 (168 to 299)	51.3 (35.4 to 67.8)	-67.4 (-77.7 to -53.3)	-57.0 (-69.2 to -41.5)	186 (133 to 240)	39.7 (28.5 to 52.3)	-69.8 (-79.0 to -55.6)	-65.2 (-74.7 to -52.0)
Viet Nam	7,580 (6,080 to 9,400)	24.8 (20.1 to 30.7)	-40.3 (-54.7 to -20.9)	-46.0 (-57.5 to -28.8)	4,290 (3,150 to 5,720)	9.9 (7.3 to 13.0)	-51.3 (-64.7 to -33.4)	-65.2 (-74.4 to -52.7)
**Sub-Saharan Africa**	**294,000 (240,000 to 358,000)**	**82.8 (69.2 to 97.2)**	**-22.7 (-37.8 to -1.1)**	**-43.6 (-51.1 to -33.5)**	**252,000 (207,000 to 304,000)**	**60.7 (50.2 to 71.2)**	**-20.2 (-37.7 to 0.4)**	**-45.5 (-54.1 to -35.4)**
Central Sub-Saharan Africa	25,600 (18,800 to 33,800)	86.8 (62.4 to 116.4)	-37.7 (-54.0 to -17.0)	-46.9 (-57.2 to -34.2)	23,100 (17,000 to 30,400)	60.3 (42.3 to 83.4)	-34.5 (-55.1 to -9.2)	-47.8 (-60.7 to -32.6)
Angola	4,990 (3,850 to 6,520)	72.0 (55.9 to 90.4)	-43.3 (-67.2 to -1.8)	-56.0 (-70.0 to -36.7)	4,060 (2,910 to 5,480)	41.4 (31.1 to 54.4)	-54.7 (-74.8 to -12.5)	-68.2 (-79.6 to -46.8)
Central African Republic	2,690 (1,780 to 3,860)	151.3 (108.0 to 202.3)	9.3 (-26.6 to 58.3)	-25.0 (-41.5 to -4.2)	2,110 (1,470 to 3,090)	101.2 (68.6 to 143.9)	7.3 (-31.5 to 65.1)	-23.2 (-45.0 to 6.0)

	Male				Female			
	2019		Percent change from 1990 to 2019		2019		Percent change from 1990 to 2019	
	Number of attributable deaths	Attributable mortality rate (per 100,000)	Number of attributable deaths	Attributable mortality rate	Number of attributable deaths	Attributable mortality rate (per 100,000)	Number of attributable deaths	Attributable mortality rate
Congo	653 (471 to 869)	56.1 (40.2 to 75.8)	-26.5 (-45.9 to -0.8)	-53.3 (-64.1 to -40.2)	553 (393 to 750)	42.5 (29.5 to 58.2)	-25.6 (-49.2 to 4.3)	-48.5 (-63.8 to -30.2)
Democratic Republic of the Congo	16,900 (11,500 to 23,700)	91.2 (61.3 to 130.7)	-40.0 (-57.9 to -16.9)	-44.6 (-57.1 to -29.9)	16,200 (11,100 to 22,400)	65.6 (43.1 to 96.1)	-29.8 (-53.9 to -0.9)	-42.7 (-57.2 to -25.4)
Equatorial Guinea	116 (78 to 167)	51.8 (36.7 to 69.9)	-69.1 (-81.8 to -44.5)	-69.1 (-79.8 to -54.0)	92 (59 to 136)	31.2 (20.2 to 44.8)	-70.6 (-85.1 to -42.5)	-72.3 (-83.8 to -51.3)
Gabon	226 (161 to 304)	53.0 (37.3 to 72.3)	-38.1 (-54.4 to -15.8)	-49.8 (-62.0 to -35.1)	125 (86 to 176)	23.1 (15.5 to 33.5)	-53.0 (-67.3 to -31.9)	-61.9 (-73.0 to -46.4)
Eastern Sub-Saharan Africa	89,300 (72,800 to 109,000)	79.4 (65.7 to 93.1)	-43.4 (-54.9 to -26.7)	-51.3 (-59.0 to -41.5)	77,100 (63,000 to 93,800)	58.4 (47.9 to 68.7)	-38.1 (-53.4 to -22.1)	-51.3 (-59.3 to -42.1)
Burundi	2,670 (1,820 to 3,870)	89.1 (65.2 to 116.2)	-34.7 (-55.6 to 0.7)	-47.5 (-60.4 to -29.7)	2,500 (1,640 to 3,550)	71.5 (48.3 to 98.3)	-29.4 (-56.1 to 6.5)	-41.4 (-58.5 to -20.1)
Comoros	142 (104 to 188)	61.6 (45.6 to 81.0)	-54.9 (-69.2 to -25.7)	-52.1 (-64.5 to -26.9)	166 (118 to 215)	58.5 (41.4 to 75.4)	-45.6 (-61.8 to -8.7)	-48.5 (-61.7 to -21.3)
Djibouti	251 (179 to 354)	69.6 (50.9 to 93.6)	9.6 (-29.0 to 72.1)	-37.6 (-53.1 to -15.1)	176 (122 to 244)	47.1 (33.7 to 64.9)	-7.3 (-42.8 to 49.1)	-43.7 (-60.5 to -22.5)

	Male				Female			
	2019		Percent change from 1990 to 2019		2019		Percent change from 1990 to 2019	
	Number of attributable deaths	Attributable mortality rate (per 100,000)	Number of attributable deaths	Attributable mortality rate	Number of attributable deaths	Attributable mortality rate (per 100,000)	Number of attributable deaths	Attributable mortality rate
Eritrea	1,790 (1,060 to 2,900)	124.8 (69.8 to 213.1)	-35.8 (-63.7 to 5.7)	-41.8 (-60.8 to -11.3)	1,450 (908 to 2,290)	81.1 (49.1 to 138.6)	-26.5 (-61.7 to 35.3)	-38.7 (-62.3 to 2.1)
Ethiopia	20,600 (16,600 to 25,900)	72.4 (57.7 to 88.9)	-63.7 (-73.3 to -47.2)	-66.6 (-74.8 to -52.6)	16,400 (13,100 to 20,400)	52.2 (42.5 to 61.7)	-60.9 (-73.6 to -46.1)	-65.5 (-73.9 to -54.1)
Kenya	8,130 (6,370 to 10,000)	73.9 (56.4 to 93.7)	-24.1 (-41.8 to -1.6)	-32.0 (-43.6 to -18.5)	6,910 (5,310 to 8,880)	51.0 (37.6 to 67.8)	-21.5 (-41.5 to 3.9)	-39.5 (-51.8 to -25.3)
Madagascar	5,290 (3,930 to 7,070)	70.8 (53.9 to 91.9)	-38.9 (-55.6 to -16.8)	-47.6 (-59.4 to -32.9)	5,300 (3,870 to 6,940)	65.6 (48.0 to 86.1)	-28.5 (-49.2 to -3.3)	-39.9 (-55.3 to -22.4)
Malawi	3,970 (3,060 to 5,070)	89.6 (71.4 to 109.2)	-41.5 (-57.2 to -19.2)	-39.3 (-52.2 to -23.3)	3,250 (2,490 to 4,190)	54.2 (42.1 to 66.9)	-48.3 (-63.9 to -26.4)	-53.7 (-64.6 to -39.7)
Mozambique	6,600 (4,880 to 8,690)	94.5 (73.9 to 118.4)	-35.3 (-59.3 to 12.9)	-34.2 (-50.9 to -7.7)	5,860 (4,120 to 7,920)	57.4 (41.4 to 74.0)	-34.2 (-63.1 to 16.4)	-48.7 (-66.2 to -24.0)
Rwanda	2,220 (1,650 to 2,950)	75.0 (55.6 to 95.4)	-64.4 (-75.6 to -42.1)	-58.0 (-68.6 to -43.0)	2,170 (1,620 to 2,780)	55.7 (40.2 to 70.6)	-54.2 (-70.4 to -33.2)	-55.6 (-66.3 to -39.7)
Somalia	10,400 (6,970 to 14,900)	138.5 (102.6 to 183.3)	31.9 (-12.5 to 105.3)	-33.3 (-49.0 to -11.3)	8,080 (5,460 to 11,800)	99.1 (68.9 to 144.0)	51.7 (4.2 to 123.2)	-30.2 (-49.4 to -4.0)

	Male				Female			
	2019		Percent change from 1990 to 2019		2019		Percent change from 1990 to 2019	
	**Number of attributable deaths**	**Attributable mortality rate (per 100,000)**	**Number of attributable deaths**	**Attributable mortality rate**	**Number of attributable deaths**	**Attributable mortality rate (per 100,000)**	**Number of attributable deaths**	**Attributable mortality rate**
South Sudan	3,920 (2,820 to 5,360)	93.2 (71.6 to 118.8)	-32.6 (-53.5 to -6.1)	-43.2 (-56.9 to -27.5)	2,820 (1,930 to 4,100)	69.4 (52.4 to 90.3)	-32.2 (-55.1 to 1.6)	-43.6 (-56.9 to -25.3)
Uganda	6,720 (5,040 to 8,870)	73.3 (56.3 to 92.1)	-22.6 (-47.3 to 16.4)	-32.3 (-47.0 to -13.0)	6,020 (4,370 to 7,880)	52.1 (38.8 to 66.5)	-0.4 (-38.1 to 53.3)	-27.7 (-48.2 to 1.6)
United Republic of Tanzania	13,200 (10,100 to 17,200)	73.8 (58.6 to 90.7)	-38.7 (-56.1 to -15.4)	-53.8 (-63.9 to -41.5)	13,300 (10,000 to 17,400)	61.6 (48.9 to 76.7)	-30.4 (-49.0 to -1.2)	-51.4 (-62.6 to -37.5)
Zambia	3,240 (2,460 to 4,210)	68.7 (52.4 to 86.5)	-51.5 (-65.8 to -28.3)	-55.1 (-65.4 to -40.8)	2,690 (2,020 to 3,500)	48.9 (37.1 to 62.4)	-54.1 (-68.3 to -33.9)	-61.0 (-71.2 to -47.7)
Southern Sub-Saharan Africa	17,100 (14,500 to 19,800)	67.0 (56.8 to 76.8)	-3.9 (-18.0 to 14.4)	-28.7 (-37.6 to -19.1)	12,300 (10,200 to 14,600)	38.1 (31.1 to 45.3)	-14.8 (-28.5 to 1.0)	-37.8 (-46.3 to -28.2)
Botswana	554 (405 to 729)	88.1 (65.5 to 113.6)	42.6 (3.8 to 90.4)	-28.7 (-47.3 to -5.2)	378 (254 to 546)	47.5 (31.9 to 69.1)	39.6 (0.7 to 94.6)	-28.9 (-48.6 to -1.1)
Eswatini	262 (184 to 353)	79.6 (57.9 to 107.0)	-18.4 (-41.6 to 15.0)	-27.9 (-43.8 to -6.2)	167 (111 to 231)	41.3 (27.2 to 57.0)	-25.7 (-49.9 to 7.7)	-38.7 (-57.3 to -14.1)
Lesotho	879 (684 to 1,090)	141.4 (113.0 to 174.7)	4.5 (-20.9 to 34.8)	5.3 (-15.2 to 31.0)	606 (445 to 803)	78.8 (57.9 to 103.6)	-8.5 (-34.3 to 24.1)	1.9 (-25.7 to 38.8)

	Male				Female			
	2019		Percent change from 1990 to 2019		2019		Percent change from 1990 to 2019	
	Number of attributable deaths	Attributable mortality rate (per 100,000)	Number of attributable deaths	Attributable mortality rate	Number of attributable deaths	Attributable mortality rate (per 100,000)	Number of attributable deaths	Attributable mortality rate
Namibia	499 (350 to 693)	77.6 (55.9 to 106.2)	-7.4 (-34.0 to 30.3)	-35.9 (-51.2 to -15.6)	335 (229 to 482)	37.4 (25.5 to 53.0)	-14.7 (-41.5 to 25.6)	-48.9 (-63.8 to -29.5)
South Africa	9,570 (8,210 to 10,900)	51.7 (44.1 to 59.1)	-18.9 (-31.0 to -4.8)	-38.9 (-46.8 to -30.5)	6,900 (5,720 to 8,110)	29.1 (24.1 to 34.3)	-31.6 (-42.7 to -18.5)	-50.7 (-57.4 to -42.7)
Zimbabwe	5,350 (4,140 to 6,600)	132.9 (102.0 to 162.5)	36.6 (5.0 to 81.4)	4.9 (-16.1 to 30.2)	3,930 (2,910 to 5,110)	78.0 (52.9 to 104.2)	39.6 (2.4 to 88.9)	-1.0 (-24.5 to 30.1)
Western Sub-Saharan Africa	162,000 (129,000 to 201,000)	88.8 (72.6 to 106.6)	-1.0 (-23.2 to 32.7)	-37.9 (-48.1 to -23.4)	139,000 (112,000 to 172,000)	69.3 (56.9 to 83.0)	-1.5 (-26.0 to 28.8)	-41.2 (-52.3 to -27.4)
Benin	4,000 (2,750 to 5,610)	95.7 (70.2 to 125.6)	-5.4 (-37.0 to 38.9)	-40.9 (-55.4 to -22.8)	3,530 (2,400 to 4,920)	70.1 (50.6 to 91.9)	-3.9 (-36.4 to 41.6)	-43.8 (-58.9 to -25.5)
Burkina Faso	11,200 (7,790 to 15,500)	127.2 (100.2 to 160.1)	26.5 (-12.7 to 86.3)	-30.1 (-45.4 to -9.8)	10,400 (7,510 to 14,200)	94.4 (74.2 to 120.2)	24.4 (-15.2 to 85.7)	-34.5 (-51.1 to -12.6)
Cabo Verde	89 (68 to 110)	51.5 (39.2 to 63.5)	18.0 (-13.0 to 55.9)	-0.5 (-22.9 to 26.6)	61 (45 to 80)	23.6 (17.4 to 30.9)	-21.7 (-43.3 to 7.2)	-42.8 (-57.3 to -24.3)
Cameroon	6,980 (5,020 to 9,590)	86.7 (62.3 to 115.8)	43.9 (1.2 to 107.6)	-24.3 (-41.9 to -0.3)	5,880 (4,090 to 8,450)	60.4 (44.0 to 82.7)	31.1 (-12.0 to 94.8)	-35.4 (-53.1 to -12.6)

	Male				Female			
	2019		Percent change from 1990 to 2019		2019		Percent change from 1990 to 2019	
	Number of attributable deaths	Attributable mortality rate (per 100,000)	Number of attributable deaths	Attributable mortality rate	Number of attributable deaths	Attributable mortality rate (per 100,000)	Number of attributable deaths	Attributable mortality rate
Chad	9,770 (7,030 to 13,100)	131.2 (103.7 to 163.2)	55.5 (9.0 to 124.6)	-25.4 (-43.8 to -2.2)	7,590 (5,330 to 10,500)	102.0 (77.1 to 129.8)	43.1 (-2.6 to 107.5)	-27.6 (-45.5 to -3.5)
Côte d'Ivoire	7,790 (5,640 to 10,400)	96.4 (71.6 to 125.3)	-6.2 (-32.6 to 30.3)	-37.4 (-51.2 to -21.1)	5,990 (4,140 to 8,050)	66.8 (49.0 to 85.9)	-14.7 (-42.0 to 22.0)	-40.2 (-54.7 to -22.5)
Gambia	518 (402 to 655)	101.8 (76.3 to 131.3)	-13.3 (-37.8 to 20.0)	-30.7 (-49.0 to -7.6)	405 (306 to 523)	68.9 (51.6 to 89.0)	-5.7 (-35.1 to 36.8)	-28.5 (-48.6 to -1.8)
Ghana	6,950 (5,300 to 8,650)	94.4 (72.7 to 118.1)	27.8 (-11.2 to 74.0)	-22.5 (-41.7 to 1.9)	3,730 (2,750 to 4,920)	36.8 (27.6 to 47.1)	-21.0 (-47.4 to 17.1)	-53.2 (-66.2 to -35.3)
Guinea	6,230 (4,570 to 8,400)	132.6 (98.9 to 172.2)	-22.3 (-46.1 to 12.6)	-29.6 (-47.4 to -6.6)	5,110 (3,580 to 6,920)	101.2 (73.8 to 131.5)	-31.0 (-53.6 to -0.4)	-43.3 (-58.3 to -24.8)
Guinea-Bissau	464 (353 to 609)	121.8 (92.9 to 156.1)	-45.4 (-62.5 to -20.6)	-41.3 (-56.1 to -22.0)	388 (287 to 505)	79.9 (58.9 to 104.5)	-35.8 (-56.7 to -5.7)	-39.0 (-56.1 to -15.6)
Liberia	763 (558 to 1,020)	64.3 (47.6 to 83.9)	-63.0 (-74.1 to -47.2)	-62.3 (-71.9 to -49.8)	700 (494 to 952)	57.0 (40.2 to 75.1)	-60.3 (-73.2 to -42.8)	-62.0 (-72.9 to -49.3)
Mali	6,270 (4,310 to 8,900)	56.3 (39.4 to 76.1)	27.1 (-18.3 to 92.9)	-37.3 (-55.7 to -12.9)	7,490 (5,260 to 9,950)	60.1 (43.6 to 77.7)	57.7 (8.2 to 133.4)	-29.9 (-48.4 to -5.0)

	Male				Female			
	2019		Percent change from 1990 to 2019		2019		Percent change from 1990 to 2019	
	**Number of attributable deaths**	**Attributable mortality rate (per 100,000)**	**Number of attributable deaths**	**Attributable mortality rate**	**Number of attributable deaths**	**Attributable mortality rate (per 100,000)**	**Number of attributable deaths**	**Attributable mortality rate**
Mauritania	648 (453 to 905)	55.2 (40.5 to 73.7)	-35.1 (-54.8 to -5.7)	-54.2 (-65.4 to -38.3)	593 (418 to 815)	50.2 (36.4 to 66.7)	-36.1 (-57.7 to -7.3)	-49.6 (-64.3 to -31.4)
Niger	11,100 (7,660 to 15,400)	110.1 (81.7 to 142.7)	-14.3 (-47.3 to 40.3)	-51.1 (-65.6 to -29.3)	10,400 (7,220 to 14,400)	95.8 (72.3 to 126.5)	-11.2 (-48.3 to 43.7)	-52.6 (-66.8 to -33.8)
Nigeria	81,200 (63,300 to 104,000)	79.4 (62.8 to 99.4)	-4.2 (-30.7 to 38.7)	-42.9 (-56.3 to -23.2)	70,300 (55,200 to 87,200)	67.5 (54.5 to 83.1)	-1.3 (-30.9 to 42.8)	-42.4 (-57.6 to -19.4)
Sao Tome and Principe	33 (25 to 42)	65.7 (49.8 to 85.2)	-47.7 (-63.4 to -26.4)	-45.1 (-58.8 to -27.7)	29 (21 to 39)	53.2 (37.9 to 73.8)	-51.4 (-66.9 to -30.7)	-48.4 (-63.3 to -29.4)
Senegal	2,720 (2,000 to 3,570)	59.5 (45.1 to 77.6)	-33.4 (-53.4 to -5.4)	-45.7 (-59.5 to -29.2)	2,420 (1,750 to 3,190)	49.7 (37.3 to 64.8)	-30.7 (-51.9 to 0.5)	-44.8 (-59.0 to -26.3)
Sierra Leone	3,430 (2,430 to 4,700)	114.1 (84.7 to 149.0)	-21.7 (-47.7 to 13.9)	-44.6 (-59.1 to -26.1)	2,860 (1,950 to 4,070)	90.0 (66.3 to 119.5)	-24.7 (-50.6 to 14.3)	-44.3 (-59.9 to -22.7)
Togo	1,780 (1,320 to 2,390)	100.3 (77.7 to 127.4)	1.1 (-26.7 to 39.2)	-24.3 (-40.1 to -5.6)	1,520 (1,110 to 2,000)	63.8 (46.1 to 84.5)	-9.0 (-37.0 to 34.7)	-39.4 (-53.5 to -21.0)

Appendix Table 5. Lower respiratory infection mortality attributable to all evaluated risk factors combined in 2019 and the percent change in attributable mortality between 1990 and 2019 by age, sex, and GBD super-regions (95% UI)

		Male				Female			
		2019		Percent change from 1990 to 2019		2019		Percent change from 1990 to 2019	
	Age Group	Number of attributable deaths	Attributable mortality rate (per 100 000 people)	Number of attributable deaths	Attributable mortality rate (per 100 000 people)	Number of attributable deaths	Attributable mortality rate (per 100 000 people)	Number of attributable deaths	Attributable mortality rate (per 100 000 people)
Global	0-4 years	319,000 (256,000 to 401,000)	93.0 (74.8 to 117.1)	-71.0 (-77.5 to -62.3)	-72.4 (-78.6 to -64.1)	310,000 (254,000 to 378,000)	96.7 (79.2 to 117.8)	-68.7 (-76.1 to -60.1)	-70.0 (-77.2 to -61.9)
	5-14 years	12,500 (9,960 to 15,500)	1.9 (1.5 to 2.3)	-51.2 (-59.8 to -38.0)	-58.0 (-65.5 to -46.7)	11,800 (9,010 to 14,900)	1.9 (1.4 to 2.4)	-55.6 (-64.6 to -45.9)	-61.3 (-69.2 to -52.9)
	15-49 years	64,700 (55,700 to 74,200)	3.3 (2.8 to 3.7)	13.9 (0.7 to 28.8)	-21.4 (-30.5 to -11.0)	36,600 (29,800 to 44,000)	1.9 (1.5 to 2.3)	-9.1 (-21.8 to 4.9)	-37.5 (-46.2 to -27.9)
	50-69 years	149,000 (129,000 to 168,000)	22.0 (19.1 to 24.8)	29.3 (14.6 to 44.3)	-35.7 (-43.0 to -28.2)	84,700 (69,400 to 100,000)	12.1 (9.9 to 14.3)	25.4 (8.6 to 45.7)	-38.3 (-46.6 to -28.4)
	70+ years	331,000 (287,000 to 375,000)	162.9 (141.3 to 184.6)	66.6 (50.4 to 82.8)	-32.7 (-39.2 to -26.1)	282,000 (230,000 to 334,000)	108.4 (88.6 to 128.4)	54.1 (37.6 to 70.5)	-29.3 (-36.9 to -21.8)
Central Europe, Eastern Europe, and	0-4 years	6,710 (5,370 to 8,420)	47.2 (37.8 to 59.3)	-78.6 (-83.2 to -72.8)	-72.7 (-78.5 to -65.2)	5,340 (4,300 to 6,660)	39.9 (32.2 to 49.9)	-78.7 (-83.0 to -73.2)	-72.2 (-77.7 to -65.0)
	5-14 years	360 (288 to 440)	1.3 (1.0 to 1.6)	-50.8 (-58.4 to -41.8)	-37.4 (-47.0 to -25.8)	297 (241 to 363)	1.1 (0.9 to 1.4)	-54.9 (-61.3 to -46.2)	-41.0 (-49.4 to -29.6)

Central Asia	15-49 years	8,680 (7,280 to 10,000)	8.7 (7.3 to 10.0)	77.9 (55.8 to 99.8)	81.8 (59.3 to 104.2)	2,300 (1,850 to 2,780)	2.3 (1.9 to 2.8)	36.7 (17.0 to 58.7)	40.7 (20.3 to 63.2)
	50-69 years	13,800 (11,700 to 15,900)	31.0 (26.3 to 35.6)	63.0 (43.9 to 82.0)	32.8 (17.2 to 48.3)	3,280 (2,680 to 3,860)	6.0 (4.9 to 7.1)	22.3 (7.7 to 36.4)	5.5 (-7.1 to 17.6)
	70+ years	10,800 (9,130 to 12,300)	84.8 (71.8 to 96.3)	51.6 (35.6 to 65.9)	-10.0 (-19.4 to -1.4)	8,450 (6,590 to 10,300)	34.4 (26.9 to 42.1)	14.4 (1.9 to 26.6)	-17.6 (-26.6 to -8.8)
High-income	0-4 years	644 (500 to 796)	2.2 (1.7 to 2.7)	-76.1 (-80.4 to -71.3)	-74.2 (-78.8 to -69.0)	495 (388 to 600)	1.8 (1.4 to 2.2)	-76.8 (-80.6 to -72.9)	-75.0 (-79.1 to -70.8)
	5-14 years	56 (46 to 68)	0.1 (0.1 to 0.1)	-72.1 (-75.4 to -67.2)	-71.2 (-74.6 to -66.2)	57 (46 to 68)	0.1 (0.1 to 0.1)	-70.1 (-73.9 to -62.2)	-69.2 (-73.0 to -60.9)
	15-49 years	2,540 (2,240 to 2,790)	1.0 (0.9 to 1.1)	-38.4 (-41.8 to -34.7)	-40.6 (-43.9 to -37.0)	1,430 (1,240 to 1,600)	0.6 (0.5 to 0.7)	-27.2 (-31.6 to -22.5)	-29.3 (-33.7 to -24.8)
	50-69 years	14,200 (12,700 to 15,600)	10.6 (9.5 to 11.7)	-3.8 (-8.8 to 1.3)	-40.0 (-43.1 to -36.8)	6,640 (5,830 to 7,440)	4.8 (4.2 to 5.3)	2.8 (-3.4 to 8.6)	-32.3 (-36.3 to -28.4)
	70+ years	88,400 (75,000 to 100,000)	141.7 (120.1 to 160.9)	38.4 (27.4 to 46.7)	-36.7 (-41.7 to -32.9)	72,400 (56,000 to 86,800)	86.8 (67.1 to 104.0)	27.0 (13.3 to 37.9)	-27.7 (-35.5 to -21.5)
Latin America and Caribbean	0-4 years	8,820 (6,570 to 11,600)	36.0 (26.8 to 47.3)	-81.2 (-86.6 to -75.0)	-80.5 (-86.1 to -74.1)	6,900 (5,230 to 8,810)	29.3 (22.2 to 37.4)	-82.6 (-87.3 to -77.3)	-81.8 (-86.7 to -76.2)
	5-14 years	324 (238 to 419)	0.7 (0.5 to 0.8)	-73.7 (-79.1 to -67.8)	-74.6 (-79.8 to -68.9)	308 (230 to 398)	0.6 (0.5 to 0.8)	-74.2 (-78.7 to -69.1)	-74.5 (-79.0 to -69.5)
	15-49 years	4,660 (3,860 to 5,500)	3.1 (2.5 to 3.6)	-7.6 (-18.3 to 4.7)	-41.2 (-48.0 to -33.4)	2,140 (1,720 to 2,640)	1.4 (1.1 to 1.7)	-26.8 (-37.6 to -14.8)	-53.0 (-59.9 to -45.3)
	50-69 years	9,730 (8,150 to 11,300)	20.8 (17.5 to 24.3)	41.8 (25.9 to 60.4)	-43.9 (-50.2 to -36.5)	5,240 (4,290 to 6,250)	10.0 (8.2 to 12.0)	35.4 (18.3 to 52.7)	-49.2 (-55.6 to -42.7)
	70+ years	21,600 (17,400 to 25,800)	150.0 (120.6 to 179.4)	78.6 (59.4 to 99.3)	-37.4 (-44.2 to -30.2)	19,700 (15,200 to 24,000)	108.1 (83.3 to 131.6)	81.1 (59.7 to 103.7)	-41.6 (-48.5 to -34.3)

North Africa and Middle East	0-4 years	14,100 (10,800 to 18,200)	46.1 (35.2 to 59.2)	-80.3 (-86.6 to -72.7)	-82.4 (-88.0 to -75.7)	14,200 (11,000 to 17,700)	49.0 (37.8 to 60.9)	-80.0 (-85.6 to -73.6)	-82.1 (-87.1 to -76.4)
	5-14 years	949 (694 to 1,250)	1.6 (1.2 to 2.1)	-49.2 (-65.1 to -33.4)	-60.7 (-73.0 to -48.4)	870 (566 to 1,150)	1.5 (1.0 to 2.0)	-53.0 (-64.9 to -38.7)	-63.2 (-72.5 to -52.0)
	15-49 years	3,860 (3,090 to 4,810)	2.2 (1.8 to 2.8)	60.2 (32.5 to 92.9)	-23.7 (-36.9 to -8.1)	2,540 (1,890 to 3,270)	1.6 (1.2 to 2.1)	25.2 (-0.0 to 54.4)	-37.6 (-50.2 to -23.1)
	50-69 years	7,510 (6,010 to 9,140)	18.3 (14.6 to 22.3)	74.6 (43.6 to 116.4)	-31.5 (-43.7 to -15.1)	3,890 (2,890 to 4,990)	10.1 (7.5 to 12.9)	57.6 (26.4 to 110.7)	-37.6 (-49.9 to -16.5)
	70+ years	13,200 (11,000 to 15,800)	136.1 (112.9 to 162.6)	112.3 (81.8 to 158.1)	-20.1 (-31.6 to -2.9)	8,980 (7,280 to 11,000)	91.8 (74.4 to 112.4)	94.4 (61.8 to 155.5)	-24.1 (-36.9 to -0.3)
South Asia	0-4 years	83,900 (66,100 to 105,000)	97.9 (77.1 to 122.9)	-76.5 (-82.6 to -67.7)	-77.0 (-83.0 to -68.4)	98,500 (79,200 to 121,000)	125.2 (100.7 to 154.2)	-70.5 (-78.7 to -61.1)	-70.8 (-78.9 to -61.5)
	5-14 years	3,940 (2,870 to 5,170)	2.2 (1.6 to 2.8)	-54.1 (-64.6 to -38.4)	-63.9 (-72.2 to -51.6)	4,450 (3,100 to 5,800)	2.6 (1.8 to 3.4)	-63.7 (-72.6 to -52.9)	-71.4 (-78.4 to -63.0)
	15-49 years	11,000 (8,700 to 13,700)	2.2 (1.8 to 2.7)	7.6 (-15.6 to 34.9)	-40.6 (-53.4 to -25.5)	9,180 (6,910 to 11,600)	1.9 (1.4 to 2.4)	-5.7 (-29.1 to 22.5)	-49.7 (-62.1 to -34.6)
	50-69 years	33,400 (26,000 to 41,000)	27.2 (21.2 to 33.4)	27.3 (-1.0 to 60.2)	-40.9 (-54.0 to -25.6)	30,200 (22,600 to 38,900)	24.7 (18.4 to 31.8)	47.4 (8.1 to 99.1)	-39.3 (-55.5 to -18.0)
	70+ years	67,300 (54,000 to 82,400)	203.9 (163.6 to 249.5)	103.1 (60.5 to 155.6)	-31.1 (-45.6 to -13.3)	65,700 (49,000 to 83,800)	182.1 (135.8 to 232.1)	136.3 (72.4 to 218.9)	-30.0 (-48.9 to -5.5)
Southeast Asia, East Asia, and Oceania	0-4 years	27,100 (22,700 to 32,000)	36.5 (30.5 to 43.1)	-90.7 (-92.8 to -88.3)	-88.2 (-90.9 to -85.1)	20,800 (17,600 to 24,400)	31.3 (26.5 to 36.8)	-92.0 (-93.7 to -89.8)	-89.6 (-91.8 to -86.8)
	5-14 years	965 (751 to 1,240)	0.7 (0.5 to 0.9)	-85.4 (-88.3 to -75.0)	-82.4 (-85.8 to -69.8)	737 (563 to 964)	0.6 (0.4 to 0.8)	-85.8 (-88.8 to -78.9)	-81.9 (-85.7 to -73.1)

	15-49 years	9,700 (8,060 to 11,600)	1.7 (1.4 to 2.0)	-33.2 (-45.5 to -10.9)	-44.3 (-54.5 to -25.7)	3,910 (3,040 to 4,960)	0.7 (0.6 to 0.9)	-56.0 (-65.3 to -44.3)	-63.3 (-71.1 to -53.6)
	50-69 years	31,200 (26,400 to 36,600)	12.7 (10.7 to 14.8)	20.1 (-0.9 to 46.8)	-48.5 (-57.6 to -37.1)	11,500 (9,020 to 14,300)	4.6 (3.6 to 5.7)	-23.1 (-37.5 to -4.6)	-68.7 (-74.5 to -61.1)
	70+ years	82,500 (69,800 to 95,600)	131.9 (111.7 to 152.9)	77.7 (49.9 to 116.3)	-37.8 (-47.5 to -24.3)	62,600 (49,200 to 78,100)	80.8 (63.4 to 100.7)	24.3 (3.2 to 51.1)	-53.9 (-61.7 to -43.9)
Sub-Saharan Africa	0-4 years	177,000 (135,000 to 230,000)	211.4 (160.9 to 274.5)	-40.8 (-55.6 to -18.1)	-68.2 (-76.1 to -55.9)	164,000 (128,000 to 208,000)	199.9 (156.2 to 254.5)	-36.1 (-53.4 to -15.2)	-65.2 (-74.7 to -53.9)
	5-14 years	5,860 (4,380 to 7,690)	4.0 (3.0 to 5.3)	-6.9 (-29.6 to 31.2)	-57.0 (-67.5 to -39.4)	5,120 (3,670 to 6,800)	3.6 (2.5 to 4.7)	-4.1 (-29.4 to 28.1)	-55.1 (-66.9 to -40.0)
	15-49 years	24,300 (19,400 to 30,100)	9.6 (7.7 to 11.9)	55.2 (26.9 to 85.7)	-34.4 (-46.4 to -21.5)	15,100 (11,400 to 19,300)	5.7 (4.3 to 7.3)	15.7 (-7.7 to 42.7)	-51.3 (-61.1 to -39.9)
	50-69 years	38,900 (31,400 to 46,800)	95.9 (77.4 to 115.3)	36.7 (13.5 to 63.4)	-34.4 (-45.5 to -21.5)	24,000 (18,300 to 29,800)	53.9 (41.2 to 67.0)	44.4 (18.4 to 78.4)	-38.5 (-49.6 to -24.1)
	70+ years	47,500 (39,600 to 55,800)	552.4 (461.0 to 649.0)	58.5 (35.0 to 83.2)	-20.6 (-32.4 to -8.2)	44,300 (35,100 to 53,300)	415.9 (329.6 to 500.8)	77.1 (50.8 to 106.3)	-16.9 (-29.2 to -3.2)

Appendix Table 6. Lower respiratory infection age-standardized risk-deleted mortality rate (per 100,000 people) for all evaluated risk factors combined by sex for 204 countries and territories and 21 GBD regions for 1990 and 2019 (95% UI)

	1990			2019		
	**Male**	**Female**	**Male to female ratio**	**Male**	**Female**	**Male to female ratio**
**Global**	**15·6 (13·0–18·3)**	**13·4 (11·2–15·7)**	**1·17 (1·07–1·28)**	**13·5 (11·4–15·7)**	**11·1 (9·24–12·7)**	**1·22 (1·12–1·36)**
**Central Europe, Eastern Europe, and Central Asia**	**9·29 (7·57–11·1)**	**7·02 (5·82–8·32)**	**1·32 (1·24–1·42)**	**9·58 (7·85–11·5)**	**5·93 (4·90–6·95)**	**1·62 (1·43–1·83)**
Central Asia	12·7 (9·77–16·2)	10·7 (8·41–13·3)	1·19 (1·12–1·26)	11·5 (9·50–13·7)	9·36 (7·80–11·1)	1·23 (1·13–1·35)
Armenia	7·28 (5·19–9·61)	6·38 (4·62–8·31)	1·14 (1·05–1·25)	6·27 (4·67–8·02)	5·84 (4·31–7·27)	1·08 (0·941–1·24)
Azerbaijan	16·2 (12·1–21·9)	14·9 (10·7–20·2)	1·10 (0·971–1·21)	9·88 (7·50–12·8)	11·2 (8·45–15·1)	0·895 (0·675–1·15)
Georgia	10·9 (8·28–13·6)	9·43 (7·19–11·8)	1·15 (1·06–1·25)	6·63 (5·05–8·21)	4·82 (3·72–5·96)	1·38 (1·21–1·58)
Kazakhstan	10·5 (8·02–13·1)	7·75 (5·96–9·64)	1·35 (1·24–1·47)	13·3 (10·7–16·3)	8·92 (7·23–10·6)	1·49 (1·31–1·70)
Kyrgyzstan	13·6 (9·52–17·9)	11·0 (7·82–14·1)	1·24 (1·15–1·34)	5·07 (3·64–6·53)	3·79 (2·77–4·86)	1·34 (1·20–1·49)
Mongolia	23·4 (14·6–32·7)	17·3 (10·9–24·4)	1·36 (1·14–1·64)	6·82 (4·72–9·29)	5·02 (3·41–7·20)	1·37 (1·10–1·64)
Tajikistan	13·2 (9·16–17·9)	13·4 (9·55–17·9)	0·982 (0·851–1·14)	15·0 (10·9–20·7)	13·2 (9·27–18·9)	1·15 (0·908–1·38)
Turkmenistan	18·8 (14·3–24·8)	17·2 (13·3–21·9)	1·09 (1·01–1·18)	8·62 (6·68–11·1)	6·33 (4·86–8·13)	1·36 (1·25–1·51)
Uzbekistan	12·1 (9·24–15·6)	10·2 (7·93–12·7)	1·19 (1·10–1·28)	14·7 (12·0–18·1)	12·4 (10·1–15·0)	1·19 (1·10–1·29)
Central Europe	9·30 (7·42–11·2)	7·86 (6·36–9·30)	1·18 (1·09–1·29)	8·15 (6·59–9·84)	5·62 (4·56–6·64)	1·45 (1·26–1·66)
Albania	19·8 (10·6–25·2)	15·2 (7·74–19·8)	1·33 (1·08–2·28)	5·39 (3·93–7·30)	4·93 (3·71–6·65)	1·10 (0·857–1·38)

	1990			2019		
	**Male**	**Female**	**Male to female ratio**	**Male**	**Female**	**Male to female ratio**
Bosnia and Herzegovina	5·09 (3·70–6·57)	4·55 (3·24–5·77)	1·12 (0·939–1·42)	3·21 (2·32–4·22)	2·82 (2·03–4·41)	1·16 (0·775–1·42)
Bulgaria	16·1 (12·9–19·6)	13·5 (11·1–16·0)	1·19 (1·06–1·33)	7·73 (6·01–9·69)	5·30 (4·12–6·65)	1·46 (1·31–1·65)
Croatia	6·41 (5·00–7·97)	5·29 (4·18–6·42)	1·21 (1·06–1·38)	3·64 (2·78–4·69)	2·38 (1·82–3·03)	1·53 (1·34–1·73)
Czechia	7·93 (6·25–9·74)	7·06 (5·57–8·44)	1·12 (1·01–1·23)	8·48 (6·49–10·7)	6·46 (4·97–8·00)	1·32 (1·19–1·46)
Hungary	4·21 (3·31–5·23)	3·35 (2·64–4·13)	1·26 (1·14–1·38)	3·37 (2·58–4·22)	2·53 (1·96–3·16)	1·34 (1·20–1·49)
Montenegro	3·73 (2·81–4·79)	2·73 (2·00–3·52)	1·37 (1·06–1·73)	3·82 (2·84–4·96)	2·64 (2·02–3·52)	1·46 (1·12–1·80)
North Macedonia	4·62 (3·41–6·17)	5·16 (3·83–6·74)	0·899 (0·736–1·08)	3·31 (2·44–4·46)	3·16 (2·33–4·66)	1·06 (0·750–1·33)
Poland	6·83 (5·34–8·46)	5·61 (4·46–6·75)	1·22 (1·11–1·34)	9·89 (7·49–12·9)	6·09 (4·55–7·74)	1·64 (1·23–2·15)
Romania	13·3 (10·5–16·5)	11·2 (8·79–13·8)	1·19 (1·09–1·29)	10·4 (8·06–13·0)	6·96 (5·58–8·56)	1·49 (1·34–1·66)
Serbia	4·57 (3·44–5·95)	4·24 (3·20–5·50)	1·08 (0·864–1·34)	5·14 (3·82–6·60)	4·29 (3·23–5·43)	1·21 (0·969–1·50)
Slovakia	21·2 (15·2–26·8)	19·1 (11·8–23·9)	1·12 (0·911–1·69)	12·3 (9·00–16·4)	9·37 (7·02–12·4)	1·32 (1·04–1·62)
Slovenia	11·0 (8·05–14·2)	9·00 (6·71–11·6)	1·22 (1·09–1·35)	9·36 (6·97–12·0)	5·60 (4·11–7·26)	1·68 (1·46–1·92)
Eastern Europe	6·60 (5·26–8·11)	4·47 (3·59–5·39)	1·48 (1·34–1·63)	8·76 (6·76–11·2)	4·49 (3·52–5·59)	1·96 (1·57–2·42)
Belarus	4·86 (3·63–5·97)	4·03 (3·11–4·84)	1·21 (1·08–1·34)	4·66 (3·45–6·07)	2·50 (1·89–3·18)	1·87 (1·58–2·20)
Estonia	6·81 (5·01–8·41)	4·06 (3·03–4·87)	1·68 (1·49–1·87)	5·80 (4·08–7·76)	3·30 (2·38–4·36)	1·76 (1·52–2·01)

	1990			2019		
	**Male**	**Female**	**Male to female ratio**	**Male**	**Female**	**Male to female ratio**
Latvia	6·01 (4·53–7·39)	3·99 (3·10–4·76)	1·51 (1·35–1·68)	6·14 (4·46–8·03)	3·67 (2·72–4·85)	1·69 (1·22–2·26)
Lithuania	4·61 (3·53–5·66)	3·37 (2·67–4·03)	1·37 (1·20–1·53)	6·56 (4·92–8·39)	3·54 (2·72–4·45)	1·85 (1·61–2·12)
Republic of Moldova	8·73 (6·68–11·2)	6·13 (4·73–7·81)	1·42 (1·28–1·57)	9·13 (7·27–11·3)	4·49 (3·68–5·46)	2·04 (1·73–2·34)
Russian Federation	7·43 (5·84–9·41)	4·81 (3·77–5·95)	1·55 (1·39–1·70)	9·27 (6·83–12·3)	4·90 (3·68–6·36)	1·90 (1·45–2·45)
Ukraine	4·96 (3·97–5·95)	3·58 (2·98–4·19)	1·39 (1·23–1·55)	8·42 (6·44–10·9)	3·78 (2·94–4·74)	2·25 (1·66–3·01)
**High-income**	**14·3 (12·2–16·5)**	**10·7 (8·93–12·1)**	**1·34 (1·26–1·44)**	**11·4 (9·62–13·1)**	**8·03 (6·42–9·13)**	**1·43 (1·34–1·54)**
Australasia	8·72 (7·42–10·0)	6·86 (5·71–7·77)	1·27 (1·18–1·38)	6·17 (5·11–7·10)	5·49 (4·24–6·40)	1·13 (1·03–1·25)
Australia	7·06 (6·08–8·15)	5·35 (4·44–6·05)	1·32 (1·21–1·44)	6·15 (5·07–7·13)	5·46 (4·21–6·39)	1·13 (1·03–1·26)
New Zealand	16·3 (13·5–19·6)	14·2 (11·3–16·8)	1·15 (1·04–1·26)	6·24 (5·08–7·49)	5·66 (4·34–6·74)	1·11 (1·02–1·22)
High-income Asia Pacific	26·4 (22·0–31·0)	17·8 (14·7–20·0)	1·49 (1·33–1·65)	18·0 (14·5–21·0)	8·90 (6·57–10·5)	2·04 (1·82–2·32)
Brunei Darussalam	42·1 (31·4–57·2)	22·6 (16·8–35·5)	1·89 (1·19–2·43)	61·5 (50·0–73·2)	32·9 (25·5–38·9)	1·88 (1·51–2·39)
Japan	27·1 (22·6–31·8)	18·2 (15·1–20·5)	1·49 (1·33–1·66)	18·5 (14·9–21·6)	8·73 (6·34–10·3)	2·14 (1·91–2·46)
Republic of Korea	10·8 (8·50–14·0)	9·52 (7·72–12·4)	1·14 (0·903–1·38)	11·9 (6·86–15·2)	8·18 (4·41–10·4)	1·50 (1·16–2·75)
Singapore	59·0 (36·1–69·9)	49·2 (28·8–57·6)	1·20 (1·10–1·30)	46·5 (36·5–53·6)	31·9 (22·9–37·2)	1·46 (1·33–1·63)
High-income North America	13·6 (11·7–15·7)	9·37 (7·77–10·7)	1·46 (1·35–1·57)	8·67 (7·49–9·76)	7·08 (5·94–7·96)	1·23 (1·15–1·31)

	1990			2019		
	**Male**	**Female**	**Male to female ratio**	**Male**	**Female**	**Male to female ratio**
Canada	12·3 (8·93–16·3)	9·02 (6·41–11·9)	1·37 (1·24–1·52)	7·31 (5·41–9·66)	6·26 (4·40–8·29)	1·17 (1·07–1·29)
United States of America	13·8 (11·8–15·8)	9·42 (7·85–10·8)	1·46 (1·36–1·59)	8·84 (7·67–9·88)	7·18 (6·05–8·03)	1·23 (1·16–1·31)
Southern Latin America	18·9 (15·5–22·3)	13·6 (11·2–15·9)	1·39 (1·30–1·49)	30·1 (25·6–35·4)	24·1 (19·8–28·0)	1·25 (1·14–1·38)
Argentina	14·0 (11·5–16·9)	9·83 (8·03–11·6)	1·43 (1·30–1·58)	40·1 (34·0–47·2)	31·0 (25·5–36·2)	1·30 (1·18–1·44)
Chile	35·9 (28·7–42·8)	31·2 (24·8–37·2)	1·15 (1·07–1·25)	11·8 (9·27–14·3)	10·0 (7·50–12·2)	1·18 (1·08–1·29)
Uruguay	13·7 (11·9–15·7)	10·9 (9·29–12·4)	1·25 (1·17–1·35)	16·8 (14·3–19·5)	14·4 (11·9–16·5)	1·17 (1·08–1·27)
Western Europe	11·0 (9·14–13·1)	9·56 (7·80–11·1)	1·16 (1·07–1·24)	8·49 (7·13–9·88)	6·72 (5·39–7·74)	1·26 (1·18–1·37)
Andorra	8·17 (5·30–11·5)	8·67 (5·45–12·5)	0·954 (0·691–1·26)	6·77 (4·77–9·04)	7·44 (4·79–10·4)	0·923 (0·701–1·19)
Austria	6·14 (4·83–7·45)	5·10 (3·99–6·05)	1·20 (1·09–1·33)	3·24 (2·55–3·93)	2·54 (1·95–3·09)	1·28 (1·14–1·42)
Belgium	12·1 (9·42–14·9)	8·22 (6·35–9·86)	1·47 (1·32–1·63)	12·2 (9·88–14·8)	9·53 (7·38–11·3)	1·29 (1·16–1·42)
Cyprus	12·4 (9·37–15·4)	22·0 (16·7–27·2)	0·570 (0·436–0·758)	5·96 (4·58–7·45)	7·92 (6·33–10·6)	0·758 (0·572–0·929)
Denmark	8·97 (6·96–11·1)	7·93 (6·05–9·84)	1·13 (1·01–1·26)	10·3 (7·98–12·7)	8·07 (6·20–9·94)	1·28 (1·15–1·42)
Finland	28·7 (20·9–35·1)	19·9 (14·3–24·2)	1·44 (1·31–1·58)	4·07 (3·03–5·04)	2·78 (2·03–3·45)	1·46 (1·34–1·62)
France	10·9 (8·74–13·1)	8·65 (6·83–10·2)	1·26 (1·11–1·41)	7·52 (6·03–9·04)	5·68 (4·29–6·86)	1·33 (1·20–1·50)
Germany	7·39 (5·88–9·18)	5·47 (4·31–6·65)	1·35 (1·21–1·51)	6·69 (5·39–8·02)	4·71 (3·74–5·61)	1·42 (1·29–1·58)

	1990			2019		
	**Male**	**Female**	**Male to female ratio**	**Male**	**Female**	**Male to female ratio**
Greece	5·71 (4·63–6·85)	6·73 (5·64–7·71)	0·850 (0·744–0·964)	8·69 (7·09–10·4)	10·8 (8·89–12·4)	0·803 (0·708–0·902)
Iceland	14·5 (10·5–18·4)	18·0 (12·7–22·9)	0·809 (0·702–0·923)	9·33 (6·60–12·0)	7·41 (5·03–9·82)	1·27 (1·11–1·45)
Ireland	22·3 (17·5–27·7)	20·7 (16·3–25·1)	1·08 (0·977–1·19)	11·5 (9·10–14·1)	11·2 (8·70–13·8)	1·03 (0·913–1·16)
Israel	9·96 (8·03–11·7)	10·7 (8·79–12·4)	0·931 (0·836–1·03)	9·65 (8·04–11·3)	8·40 (6·77–9·86)	1·15 (1·05–1·28)
Italy	5·33 (4·34–6·35)	4·73 (3·90–5·45)	1·13 (1·03–1·23)	4·20 (3·47–4·83)	3·14 (2·49–3·60)	1·34 (1·23–1·47)
Luxembourg	9·66 (7·52–12·2)	7·24 (5·53–8·80)	1·33 (1·19–1·49)	6·61 (5·02–8·35)	5·51 (4·08–7·02)	1·20 (1·04–1·38)
Malta	18·6 (15·1–22·0)	14·0 (11·4–16·4)	1·33 (1·19–1·47)	14·5 (11·5–17·6)	11·8 (9·08–14·4)	1·23 (1·12–1·36)
Monaco	11·8 (8·96–15·8)	9·22 (6·80–12·4)	1·29 (0·949–1·65)	13·2 (10·1–16·9)	10·7 (7·93–13·7)	1·24 (0·941–1·60)
Netherlands	8·68 (6·63–11·0)	7·81 (5·88–9·66)	1·11 (0·989–1·25)	9·83 (7·71–12·1)	7·73 (5·95–9·47)	1·27 (1·17–1·38)
Norway	25·6 (19·0–31·2)	21·2 (15·4–26·2)	1·21 (1·12–1·32)	12·7 (9·42–15·5)	9·61 (6·86–11·7)	1·32 (1·21–1·46)
Portugal	17·2 (14·7–19·8)	13·7 (12·0–15·2)	1·26 (1·14–1·39)	20·5 (17·1–23·7)	15·0 (12·2–17·2)	1·37 (1·23–1·53)
San Marino	8·53 (6·56–10·9)	4·96 (3·77–6·55)	1·74 (1·32–2·23)	7·08 (4·72–9·97)	4·12 (2·75–5·94)	1·74 (1·31–2·23)
Spain	9·09 (7·52–10·7)	8·75 (7·28–9·93)	1·04 (0·914–1·18)	7·32 (6·03–8·61)	5·85 (4·59–6·83)	1·25 (1·12–1·43)
Sweden	16·8 (13·0–20·9)	11·9 (8·83–14·6)	1·42 (1·25–1·60)	7·31 (5·68–9·09)	5·19 (3·89–6·47)	1·41 (1·29–1·57)
Switzerland	11·0 (8·53–13·5)	9·93 (7·47–11·9)	1·11 (0·989–1·23)	4·96 (3·92–6·09)	4·12 (3·05–5·03)	1·21 (1·09–1·35)

	1990			2019		
	**Male**	**Female**	**Male to female ratio**	**Male**	**Female**	**Male to female ratio**
United Kingdom	20·1 (16·3–24·2)	18·9 (15·3–22·5)	1·06 (0·993–1·15)	15·8 (12·9–18·7)	14·4 (11·4–16·9)	1·10 (1·03–1·18)
**Latin America and Caribbean**	**22·4 (18·8–26·3)**	**21·6 (18·2–25·0)**	**1·04 (0·973–1·11)**	**21·6 (18·3–24·9)**	**19·1 (16·0–21·8)**	**1·13 (1·06–1·21)**
Andean Latin America	39·8 (31·5–49·2)	41·1 (32·6–51·4)	0·971 (0·855–1·08)	33·4 (26·0–42·6)	32·0 (24·9–40·3)	1·05 (0·910–1·18)
Bolivia (Plurinational State of)	46·4 (34·2–60·9)	47·3 (35·3–63·1)	0·988 (0·763–1·26)	42·9 (31·6–55·6)	45·8 (35·3–60·1)	0·942 (0·721–1·15)
Ecuador	25·8 (20·7–30·7)	35·7 (28·7–42·5)	0·725 (0·638–0·852)	28·1 (22·0–35·4)	26·9 (21·5–35·1)	1·05 (0·850–1·23)
Peru	44·0 (34·0–55·8)	41·8 (32·2–53·3)	1·06 (0·921–1·20)	33·8 (24·7–45·3)	31·2 (22·6–41·7)	1·09 (0·922–1·28)
Caribbean	20·9 (17·3–24·5)	19·7 (16·3–23·2)	1·06 (0·958–1·18)	19·3 (15·8–23·0)	17·4 (14·1–20·6)	1·11 (1·01–1·21)
Antigua and Barbuda	32·2 (24·6–38·2)	19·9 (15·0–24·0)	1·62 (1·41–1·85)	26·3 (20·7–31·9)	24·7 (19·8–29·4)	1·06 (0·955–1·18)
Bahamas	33·2 (27·1–39·1)	21·5 (17·6–25·1)	1·55 (1·40–1·71)	23·8 (19·3–29·1)	17·1 (13·7–20·8)	1·39 (1·25–1·53)
Barbados	21·0 (16·1–25·0)	17·7 (13·4–20·8)	1·18 (1·05–1·31)	23·7 (18·2–28·7)	24·1 (18·8–29·4)	0·984 (0·884–1·10)
Belize	22·3 (17·3–27·5)	26·3 (20·6–32·0)	0·848 (0·750–0·960)	27·1 (21·3–32·9)	24·7 (19·4–29·7)	1·10 (0·996–1·20)
Cuba	18·4 (14·9–21·4)	17·9 (14·5–20·5)	1·03 (0·939–1·11)	22·4 (17·4–27·6)	20·1 (15·5–24·6)	1·12 (1·02–1·25)
Dominica	24·3 (18·8–29·8)	17·5 (13·8–21·7)	1·39 (1·13–1·70)	26·6 (20·4–33·7)	19·7 (15·0–24·8)	1·36 (1·10–1·73)
Dominican Republic	10·1 (7·56–13·1)	10·5 (7·80–14·1)	0·969 (0·754–1·16)	12·2 (8·95–16·6)	11·5 (8·37–15·4)	1·07 (0·817–1·35)
Grenada	40·6 (31·7–49·9)	33·0 (25·5–39·7)	1·23 (1·08–1·40)	35·4 (28·9–42·1)	35·1 (27·7–41·3)	1·01 (0·906–1·15)

	1990			2019		
	**Male**	**Female**	**Male to female ratio**	**Male**	**Female**	**Male to female ratio**
Guyana	34·1 (26·6–41·8)	29·9 (23·6–36·4)	1·14 (0·991–1·31)	32·5 (24·4–41·5)	28·5 (21·1–36·4)	1·14 (1·01–1·32)
Haiti	27·4 (16·2–43·6)	26·7 (15·9–42·3)	1·08 (0·549–1·99)	24·6 (14·1–37·8)	24·0 (14·8–36·7)	1·05 (0·613–1·69)
Jamaica	11·3 (9·03–13·7)	10·3 (8·16–12·5)	1·10 (0·997–1·22)	9·12 (6·93–11·4)	8·54 (6·47–10·7)	1·07 (0·971–1·19)
Saint Kitts and Nevis	51·0 (43·0–59·2)	42·5 (36·4–48·6)	1·20 (1·08–1·34)	35·6 (29·2–41·8)	26·7 (21·4–32·1)	1·33 (1·19–1·49)
Saint Lucia	26·1 (20·3–31·2)	21·7 (16·8–26·0)	1·20 (1·05–1·37)	21·7 (17·1–26·2)	17·0 (13·1–20·7)	1·27 (1·15–1·43)
Saint Vincent and the Grenadines	25·0 (19·3–30·3)	20·6 (15·7–24·9)	1·22 (1·09–1·35)	23·7 (18·9–28·8)	20·8 (16·4–25·4)	1·14 (1·03–1·27)
Suriname	18·1 (13·7–22·7)	14·1 (10·6–17·6)	1·29 (1·06–1·53)	19·4 (14·7–24·6)	14·9 (11·0–18·8)	1·32 (1·06–1·64)
Trinidad and Tobago	29·6 (22·5–35·0)	23·8 (18·4–27·7)	1·25 (1·14–1·35)	12·0 (8·59–15·7)	9·30 (6·61–12·1)	1·29 (1·15–1·45)
Central Latin America	16·7 (13·5–20·0)	17·8 (14·8–20·8)	0·941 (0·873–1·01)	14·3 (11·8–17·3)	11·8 (9·62–14·2)	1·21 (1·08–1·37)
Colombia	12·5 (10·0–14·9)	13·3 (10·8–15·8)	0·939 (0·866–1·01)	9·53 (7·20–12·4)	8·51 (6·37–10·9)	1·12 (1·03–1·21)
Costa Rica	10·8 (8·73–13·0)	10·7 (8·66–12·6)	1·01 (0·921–1·11)	10·4 (7·88–13·4)	6·86 (5·08–8·63)	1·53 (1·39–1·72)
El Salvador	17·7 (12·8–22·5)	15·6 (11·3–19·7)	1·14 (0·984–1·37)	27·7 (19·5–36·2)	19·7 (13·7–26·1)	1·42 (1·19–1·80)
Guatemala	63·4 (46·8–81·4)	72·4 (55·2–90·9)	0·876 (0·744–1·04)	56·8 (43·5–71·2)	41·1 (31·5–52·0)	1·38 (1·27–1·51)
Honduras	9·66 (7·01–12·9)	9·28 (6·70–12·5)	1·05 (0·820–1·31)	13·1 (9·67–17·0)	7·33 (4·73–14·3)	1·89 (0·904–2·65)
Mexico	15·8 (12·8–19·1)	18·2 (15·0–21·4)	0·870 (0·805–0·938)	13·0 (10·6–15·8)	11·1 (9·02–13·3)	1·18 (0·928–1·47)

	1990			2019		
	**Male**	**Female**	**Male to female ratio**	**Male**	**Female**	**Male to female ratio**
Nicaragua	12·1 (8·69–15·9)	7·77 (5·62–10·3)	1·56 (1·29–1·88)	14·2 (10·9–18·6)	10·6 (8·12–13·8)	1·36 (1·08–1·62)
Panama	12·1 (9·89–14·5)	10·1 (8·08–11·9)	1·20 (1·08–1·33)	14·0 (10·7–17·9)	10·9 (8·03–13·8)	1·29 (1·17–1·45)
Venezuela (Bolivarian Republic of)	15·7 (12·2–18·7)	16·9 (12·9–19·8)	0·931 (0·843–1·01)	13·8 (10·1–17·8)	11·2 (8·10–14·8)	1·23 (1·10–1·42)
Tropical Latin America	23·9 (19·9–28·5)	20·8 (17·2–24·4)	1·15 (1·06–1·24)	26·8 (22·7–30·4)	23·4 (19·6–26·3)	1·14 (1·06–1·24)
Brazil	24·5 (20·4–29·1)	21·2 (17·6–24·9)	1·15 (1·07–1·25)	27·1 (23·0–30·7)	23·7 (19·8–26·6)	1·14 (1·06–1·24)
Paraguay	9·09 (6·78–12·0)	9·84 (7·32–12·7)	0·927 (0·772–1·13)	14·7 (10·4–19·2)	12·8 (9·17–16·6)	1·15 (0·960–1·45)
**North Africa and Middle East**	**12·6 (10·3–15·3)**	**12·9 (10·5–16·2)**	**0·974 (0·824–1·13)**	**10·2 (8·28–12·2)**	**10·5 (8·41–12·4)**	**0·977 (0·856–1·16)**
Afghanistan	19·1 (11·9–26·3)	15·4 (8·80–22·5)	1·26 (0·999–1·67)	15·2 (11·0–20·8)	12·9 (8·49–17·9)	1·21 (0·900–1·76)
Algeria	16·1 (12·0–21·4)	20·7 (15·0–29·0)	0·788 (0·548–1·06)	8·96 (6·62–12·4)	13·7 (9·95–18·8)	0·664 (0·472–0·889)
Bahrain	12·8 (9·34–16·3)	11·6 (8·58–15·0)	1·11 (0·875–1·45)	9·47 (6·65–12·3)	10·4 (6·89–13·4)	0·920 (0·709–1·35)
Egypt	18·3 (14·5–22·9)	22·2 (17·4–29·9)	0·826 (0·665–0·952)	10·9 (7·72–15·5)	16·4 (11·4–25·6)	0·675 (0·459–0·895)
Iran (Islamic Republic of)	11·0 (8·94–13·2)	10·6 (8·48–12·8)	1·05 (0·887–1·28)	8·13 (6·76–9·46)	8·14 (6·42–9·47)	1·00 (0·887–1·24)
Iraq	8·35 (6·20–10·8)	7·70 (5·91–9·75)	1·09 (0·840–1·37)	5·02 (3·83–6·66)	4·78 (3·61–6·75)	1·06 (0·817–1·30)
Jordan	10·8 (8·27–13·8)	15·6 (11·8–20·0)	0·696 (0·528–0·907)	7·80 (5·93–10·4)	9·55 (7·34–12·3)	0·826 (0·591–1·08)
Kuwait	10·2 (7·66–13·2)	13·0 (9·42–16·4)	0·794 (0·671–1·05)	16·3 (11·8–21·8)	12·6 (9·01–16·6)	1·30 (1·04–1·58)

	1990			2019		
	**Male**	**Female**	**Male to female ratio**	**Male**	**Female**	**Male to female ratio**
Lebanon	12·4 (9·59–15·5)	9·61 (7·47–12·0)	1·30 (1·03–1·66)	9·63 (6·97–14·0)	5·84 (4·26–8·65)	1·67 (1·19–2·22)
Libya	9·37 (6·72–12·6)	10·5 (7·59–13·7)	0·904 (0·705–1·16)	8·56 (6·17–11·3)	9·44 (6·84–12·4)	0·913 (0·717–1·17)
Morocco	12·8 (9·03–17·1)	13·2 (9·51–17·7)	0·983 (0·741–1·33)	12·9 (9·36–17·4)	11·9 (8·79–15·8)	1·09 (0·841–1·43)
Oman	24·9 (15·7–34·4)	21·0 (13·2–29·3)	1·20 (0·895–1·62)	25·8 (17·4–34·9)	21·2 (14·6–28·5)	1·23 (0·918–1·69)
Palestine	16·6 (11·8–23·9)	14·1 (10·1–21·6)	1·19 (0·835–1·50)	15·2 (11·9–20·2)	12·6 (9·60–19·2)	1·22 (0·861–1·49)
Qatar	9·36 (6·25–14·5)	13·4 (8·75–19·4)	0·707 (0·505–0·983)	9·26 (6·25–13·2)	18·8 (13·0–25·9)	0·498 (0·370–0·669)
Saudi Arabia	16·6 (11·6–22·0)	17·6 (12·3–23·9)	0·955 (0·712–1·20)	15·0 (11·5–19·1)	15·6 (11·5–21·3)	0·968 (0·770–1·21)
Sudan	8·98 (5·28–14·2)	9·02 (5·16–13·9)	1·03 (0·621–1·73)	9·52 (5·94–14·4)	9·17 (6·08–13·4)	1·06 (0·666–1·60)
Syrian Arab Republic	8·38 (6·39–11·0)	10·2 (7·51–15·2)	0·830 (0·558–1·06)	10·6 (7·82–13·7)	13·0 (9·82–17·0)	0·818 (0·648–0·995)
Tunisia	10·4 (7·84–13·7)	11·2 (8·67–14·1)	0·939 (0·716–1·21)	8·68 (6·03–12·3)	8·31 (5·90–11·2)	1·05 (0·799–1·36)
Turkey	11·4 (8·71–14·9)	10·4 (7·86–13·8)	1·11 (0·858–1·37)	9·38 (7·08–12·0)	8·30 (5·80–10·6)	1·14 (0·938–1·55)
United Arab Emirates	39·1 (19·2–55·6)	45·0 (17·4–67·9)	0·920 (0·608–1·85)	26·0 (14·1–36·7)	24·3 (10·1–36·6)	1·13 (0·806–2·50)
Yemen	10·2 (5·78–16·3)	10·5 (5·57–16·8)	1·01 (0·579–1·73)	12·4 (7·29–19·6)	11·6 (7·22–18·8)	1·11 (0·618–1·82)
**South Asia**	**12·0 (8·67–15·8)**	**13·8 (9·53–18·7)**	**0·881 (0·650–1·14)**	**11·1 (8·32–14·2)**	**12·8 (9·61–16·8)**	**0·881 (0·669–1·14)**
Bangladesh	11·1 (7·59–15·6)	10·7 (6·81–15·0)	1·05 (0·806–1·48)	7·73 (5·43–10·7)	8·52 (5·03–11·5)	0·932 (0·688–1·58)

	1990			2019		
	**Male**	**Female**	**Male to female ratio**	**Male**	**Female**	**Male to female ratio**
Bhutan	9·01 (4·99–13·9)	9·22 (4·62–14·9)	1·02 (0·624–1·69)	11·0 (5·89–18·6)	11·8 (6·21–17·4)	0·987 (0·502–2·11)
India	13·1 (9·43–17·4)	16·2 (11·1–22·2)	0·824 (0·582–1·09)	11·5 (8·54–14·9)	13·8 (10·2–18·4)	0·848 (0·632–1·12)
Nepal	10·9 (6·68–16·0)	11·5 (6·33–19·0)	0·982 (0·591–1·55)	9·00 (5·31–13·6)	10·5 (6·20–15·3)	0·878 (0·544–1·46)
Pakistan	8·39 (5·26–12·5)	4·80 (3·09–6·87)	1·78 (1·15–2·44)	11·4 (6·70–17·2)	5·66 (4·07–7·91)	2·04 (1·15–3·08)
**Southeast Asia, East Asia, and Oceania**	**12·4 (9·40–15·7)**	**11·7 (9·01–14·8)**	**1·06 (0·901–1·26)**	**11·0 (8·95–13·2)**	**8·48 (6·69–10·1)**	**1·31 (1·11–1·58)**
East Asia	11·0 (8·39–14·1)	10·9 (8·07–14·0)	1·01 (0·858–1·25)	7·58 (6·04–9·27)	5·07 (3·87–6·33)	1·51 (1·21–1·86)
China	10·9 (8·25–14·1)	10·9 (8·01–14·1)	1·01 (0·846–1·25)	6·74 (5·32–8·36)	4·68 (3·56–5·94)	1·45 (1·13–1·83)
Democratic People's Republic of Korea	11·1 (7·55–15·6)	10·3 (7·06–14·2)	1·09 (0·825–1·43)	9·76 (6·73–13·3)	8·64 (6·01–11·8)	1·15 (0·821–1·58)
Taiwan (Province of China)	15·2 (12·2–18·3)	14·9 (12·2–17·4)	1·02 (0·892–1·16)	22·8 (17·6–29·0)	16·2 (12·2–20·3)	1·41 (1·26–1·62)
Oceania	23·7 (17·9–32·3)	20·6 (15·0–29·2)	1·16 (0·881–1·42)	21·3 (15·6–32·3)	19·1 (14·3–28·2)	1·12 (0·869–1·39)
Fiji	26·7 (20·2–33·9)	20·6 (15·1–27·2)	1·31 (1·02–1·65)	27·0 (21·0–34·9)	19·0 (14·1–24·7)	1·43 (1·15–1·80)
Kiribati	22·1 (14·7–30·2)	18·4 (12·3–26·0)	1·21 (0·922–1·54)	21·4 (15·5–28·4)	19·5 (13·9–26·3)	1·11 (0·843–1·47)
Marshall Islands	39·6 (23·3–53·7)	30·8 (17·8–44·7)	1·31 (0·940–1·75)	38·9 (28·6–51·6)	39·3 (26·3–54·9)	1·00 (0·758–1·30)
Micronesia (Federated States of)	34·8 (19·7–47·4)	31·9 (18·9–47·5)	1·11 (0·816–1·44)	39·8 (28·3–53·0)	38·1 (26·6–53·8)	1·06 (0·810–1·34)
Nauru	50·6 (29·2–68·7)	45·0 (27·6–59·2)	1·13 (0·861–1·45)	48·4 (31·8–65·3)	40·8 (28·3–55·7)	1·20 (0·851–1·63)

	1990			2019		
	**Male**	**Female**	**Male to female ratio**	**Male**	**Female**	**Male to female ratio**
Palau	94·1 (51·7–121)	105 (60·0–139)	0·901 (0·728–1·09)	78·1 (57·0–97·1)	86·8 (59·3–110)	0·905 (0·749–1·08)
Papua New Guinea	18·1 (12·4–28·9)	15·6 (10·4–26·8)	1·18 (0·744–1·60)	17·7 (11·5–32·5)	16·0 (10·6–27·2)	1·11 (0·746–1·53)
Samoa	27·9 (20·2–37·1)	26·2 (18·5–37·0)	1·08 (0·795–1·40)	21·8 (16·1–28·6)	24·6 (17·4–34·1)	0·900 (0·651–1·20)
Solomon Islands	67·2 (43·4–93·5)	63·5 (37·8–92·2)	1·09 (0·769–1·63)	55·7 (38·3–75·1)	53·8 (37·5–71·6)	1·04 (0·835–1·40)
Tonga	24·3 (17·8–32·3)	20·2 (14·9–26·2)	1·21 (0·940–1·55)	27·4 (20·9–34·8)	21·4 (15·7–28·1)	1·30 (1·02–1·67)
Tuvalu	40·7 (22·7–54·1)	39·7 (24·0–55·6)	1·03 (0·805–1·30)	34·6 (24·5–46·9)	35·4 (25·1–49·3)	0·984 (0·782–1·22)
Vanuatu	23·2 (15·5–33·7)	22·9 (14·0–35·1)	1·04 (0·671–1·50)	28·4 (19·1–40·0)	25·2 (16·6–36·8)	1·14 (0·840–1·53)
Southeast Asia	16·9 (12·3–21·7)	14·5 (10·6–19·3)	1·17 (0·929–1·41)	22·7 (17·5–27·7)	20·0 (14·0–24·1)	1·15 (0·967–1·54)
Cambodia	31·1 (17·4–43·8)	35·7 (19·9–49·5)	0·882 (0·657–1·17)	33·7 (23·0–45·3)	38·8 (26·2–50·7)	0·876 (0·670–1·19)
Indonesia	15·7 (10·7–21·0)	11·0 (7·78–14·9)	1·44 (1·00–1·98)	14·9 (11·4–18·9)	13·4 (9·63–16·4)	1·12 (0·837–1·55)
Lao People's Democratic Republic	24·6 (16·4–35·6)	25·7 (16·7–36·7)	0·976 (0·648–1·39)	23·4 (15·9–32·2)	23·4 (15·7–32·5)	1·01 (0·736–1·35)
Malaysia	28·7 (22·1–38·5)	32·0 (24·2–47·7)	0·906 (0·666–1·12)	66·1 (43·6–84·5)	68·1 (39·6–87·6)	0·987 (0·800–1·51)
Maldives	6·26 (3·60–8·69)	11·9 (7·16–16·4)	0·533 (0·377–0·779)	6·61 (4·74–8·59)	9·41 (6·65–12·0)	0·709 (0·548–0·915)
Mauritius	34·4 (27·6–40·9)	27·8 (22·9–31·7)	1·24 (1·07–1·43)	15·8 (12·2–19·6)	11·1 (8·64–13·9)	1·42 (1·24–1·62)
Myanmar	19·4 (13·4–27·3)	16·3 (11·0–23·2)	1·20 (0·884–1·64)	20·9 (15·7–27·1)	15·1 (10·3–19·6)	1·40 (1·15–1·92)

	1990			2019		
	**Male**	**Female**	**Male to female ratio**	**Male**	**Female**	**Male to female ratio**
Philippines	23·4 (15·1–30·9)	27·1 (17·2–35·9)	0·867 (0·688–1·02)	41·4 (28·3–54·8)	44·3 (27·0–57·0)	0·951 (0·677–1·41)
Seychelles	62·6 (48·8–77·1)	41·9 (33·3–49·7)	1·50 (1·19–1·85)	55·7 (42·3–70·5)	46·3 (34·5–55·9)	1·21 (0·922–1·61)
Sri Lanka	24·2 (16·0–32·2)	22·4 (14·5–29·5)	1·09 (0·843–1·43)	16·0 (10·8–22·3)	11·8 (7·97–18·2)	1·37 (0·941–1·76)
Thailand	8·96 (6·26–13·2)	9·10 (6·08–17·3)	1·02 (0·580–1·34)	19·3 (11·5–26·2)	16·4 (9·15–22·4)	1·20 (0·945–1·91)
Timor-Leste	15·2 (9·90–22·8)	21·4 (13·7–30·4)	0·722 (0·482–1·08)	28·8 (17·4–40·6)	30·3 (20·2–41·6)	0·964 (0·625–1·43)
Viet Nam	11·5 (7·85–15·8)	9·82 (6·56–14·7)	1·19 (0·858–1·56)	14·7 (11·3–19·2)	11·5 (8·47–15·9)	1·30 (0·941–1·70)
**Sub-Saharan Africa**	**33·0 (22·3–44·5)**	**26·4 (17·1–36·2)**	**1·26 (1·04–1·53)**	**34·1 (25·7–43·8)**	**26·1 (19·2–33·3)**	**1·31 (1·13–1·63)**
Central Sub-Saharan Africa	45·3 (28·8–67·0)	31·6 (19·8–46·9)	1·47 (0·940–2·16)	45·5 (30·7–66·2)	31·3 (20·8–46·7)	1·49 (1·02–2·22)
Angola	39·7 (25·4–58·3)	27·7 (16·3–41·7)	1·47 (0·927–2·15)	45·9 (34·0–59·9)	30·1 (21·6–40·5)	1·54 (1·21–1·95)
Central African Republic	50·1 (30·7–73·9)	32·8 (18·2–49·0)	1·59 (1·02–2·48)	48·1 (31·3–71·0)	32·4 (19·5–49·2)	1·53 (0·966–2·28)
Congo	45·8 (29·3–67·7)	33·8 (22·0–49·3)	1·37 (0·963–1·90)	42·0 (27·8–59·0)	39·1 (26·3–54·3)	1·09 (0·783–1·60)
Democratic Republic of the Congo	46·1 (27·2–73·2)	32·5 (19·1–53·4)	1·47 (0·804–2·43)	45·1 (27·7–71·7)	30·9 (18·8–51·1)	1·52 (0·885–2·54)
Equatorial Guinea	42·5 (24·3–66·4)	27·4 (13·2–43·7)	1·64 (0·920–2·67)	45·0 (31·8–61·9)	35·9 (23·2–52·3)	1·28 (0·949–1·71)
Gabon	48·8 (32·1–69·2)	30·0 (20·9–41·1)	1·64 (1·12–2·30)	54·4 (36·9–75·3)	32·1 (20·9–44·8)	1·74 (1·09–2·54)
Eastern Sub-Saharan Africa	35·8 (22·3–50·0)	27·7 (16·2–39·6)	1·31 (1·02–1·75)	32·4 (23·6–42·7)	24·2 (17·8–31·6)	1·34 (1·14–1·65)

	1990			2019		
	**Male**	**Female**	**Male to female ratio**	**Male**	**Female**	**Male to female ratio**
Burundi	35·5 (22·2–53·5)	31·8 (17·6–48·7)	1·16 (0·724–1·83)	31·8 (20·8–45·3)	28·1 (16·8–43·3)	1·17 (0·762–1·74)
Comoros	31·2 (18·5–46·8)	28·5 (16·5–42·8)	1·11 (0·788–1·53)	34·0 (23·0–49·1)	31·0 (20·0–44·1)	1·12 (0·783–1·58)
Djibouti	30·9 (16·8–44·8)	26·3 (14·3–39·2)	1·19 (0·870–1·60)	38·8 (24·0–55·3)	31·7 (19·1–46·8)	1·25 (0·852–1·69)
Eritrea	54·1 (23·2–102)	34·4 (14·4–67·7)	1·77 (0·549–4·18)	64·5 (32·1–119)	42·3 (22·9–79·5)	1·62 (0·689–3·31)
Ethiopia	46·7 (23·1–71·5)	31·7 (13·2–51·3)	1·55 (0·902–2·46)	28·3 (19·7–38·9)	19·7 (13·8–26·4)	1·45 (1·11–1·94)
Kenya	28·8 (19·4–40·0)	24·7 (16·6–34·5)	1·18 (0·862–1·62)	41·9 (29·5–56·2)	30·0 (20·7–42·1)	1·42 (1·02–1·90)
Madagascar	30·6 (21·9–41·9)	27·0 (18·6–37·0)	1·14 (0·949–1·41)	33·5 (21·5–48·5)	29·3 (19·0–41·7)	1·16 (0·886–1·58)
Malawi	34·5 (21·9–48·6)	29·2 (17·1–41·7)	1·20 (0·887–1·64)	33·9 (23·7–46·8)	22·0 (15·4–30·4)	1·55 (1·20–2·01)
Mozambique	28·0 (14·6–41·3)	21·5 (9·26–33·7)	1·34 (0·981–1·88)	36·9 (25·1–52·1)	21·8 (14·1–32·3)	1·72 (1·31–2·38)
Rwanda	36·4 (24·0–52·1)	29·7 (18·2–43·5)	1·25 (0·916–1·71)	26·2 (16·8–38·6)	20·6 (12·8–30·2)	1·30 (0·871–1·90)
Somalia	36·5 (14·2–58·4)	26·5 (8·12–46·9)	1·46 (0·931–2·33)	34·6 (16·1–53·6)	26·0 (9·19–42·9)	1·39 (0·972–2·11)
South Sudan	29·6 (17·1–43·3)	24·5 (13·8–37·3)	1·23 (0·914–1·64)	27·3 (17·8–39·5)	20·6 (12·9–29·5)	1·34 (0·991–1·79)
Uganda	27·6 (16·3–40·0)	20·1 (10·5–30·0)	1·40 (1·00–1·97)	31·3 (21·3–44·0)	23·0 (15·3–32·4)	1·38 (0·963–1·87)
United Republic of Tanzania	32·5 (22·6–44·8)	28·5 (19·0–39·4)	1·15 (0·914–1·47)	27·3 (18·4–38·1)	23·7 (16·1–32·7)	1·16 (0·877–1·57)
Zambia	39·5 (27·5–54·6)	31·6 (21·6–44·7)	1·26 (0·961–1·64)	41·7 (30·3–54·8)	28·0 (19·7–38·8)	1·50 (1·19–1·91)

	1990			2019		
	**Male**	**Female**	**Male to female ratio**	**Male**	**Female**	**Male to female ratio**
Southern Sub-Saharan Africa	30·1 (23·0–38·5)	25·0 (19·7–31·3)	1·21 (1·03–1·45)	42·3 (34·7–51·1)	30·1 (23·9–36·2)	1·41 (1·22–1·68)
Botswana	44·6 (27·8–66·0)	29·9 (18·5–48·0)	1·53 (1·01–2·24)	56·0 (40·2–75·5)	41·5 (26·8–63·6)	1·39 (0·956–1·97)
Eswatini	43·3 (28·6–63·1)	28·5 (19·7–38·7)	1·53 (1·12–2·00)	66·3 (47·3–90·5)	37·1 (24·1–53·6)	1·82 (1·38–2·43)
Lesotho	29·0 (19·5–40·0)	18·1 (12·5–25·5)	1·61 (1·19–2·14)	46·4 (33·1–63·2)	31·5 (20·7–45·9)	1·50 (1·14–2·03)
Namibia	50·6 (32·2–75·7)	30·9 (20·0–46·4)	1·67 (1·07–2·39)	65·7 (46·3–91·6)	33·8 (22·5–49·1)	1·98 (1·40–2·78)
South Africa	26·9 (20·6–34·4)	24·2 (19·1–30·8)	1·12 (0·935–1·32)	37·1 (30·6–44·6)	27·3 (22·1–32·8)	1·37 (1·17–1·60)
Zimbabwe	41·4 (28·8–55·6)	32·7 (21·6–44·6)	1·28 (0·974–1·88)	63·8 (45·2–85·1)	44·6 (26·1–64·1)	1·47 (1·09–2·23)
Western Sub-Saharan Africa	29·0 (17·6–40·2)	24·5 (14·5–34·3)	1·19 (0·972–1·46)	31·1 (22·3–40·7)	24·8 (16·7–33·0)	1·26 (0·989–1·63)
Benin	35·8 (18·7–50·2)	28·0 (14·6–40·2)	1·30 (1·01–1·66)	36·0 (22·6–52·0)	25·7 (16·1–35·9)	1·41 (1·08–1·92)
Burkina Faso	38·0 (15·7–57·2)	26·8 (10·8–42·5)	1·46 (1·08–1·97)	36·0 (21·8–50·4)	24·8 (14·1–35·0)	1·47 (1·16–1·88)
Cabo Verde	18·5 (12·9–25·7)	15·7 (10·9–21·8)	1·19 (0·882–1·51)	47·4 (35·9–59·3)	24·8 (18·4–32·6)	1·92 (1·52–2·38)
Cameroon	35·4 (24·2–49·6)	31·1 (19·9–44·4)	1·16 (0·828–1·59)	42·7 (27·7–61·2)	30·7 (19·4–44·5)	1·41 (1·02–1·96)
Chad	33·3 (15·3–52·9)	26·8 (10·3–45·5)	1·29 (0·829–2·00)	34·2 (22·2–48·5)	27·4 (17·2–39·6)	1·26 (0·992–1·70)
Côte d'Ivoire	41·7 (27·5–57·0)	28·5 (17·8–39·7)	1·47 (1·17–1·87)	39·0 (25·7–54·9)	26·4 (17·3–36·7)	1·49 (1·10–1·98)
Gambia	32·6 (17·3–48·0)	24·4 (12·4–36·7)	1·35 (1·04–1·72)	40·0 (25·0–57·1)	29·6 (17·9–41·9)	1·37 (0·997–1·85)

	1990			2019		
	**Male**	**Female**	**Male to female ratio**	**Male**	**Female**	**Male to female ratio**
Ghana	42·8 (25·5–60·6)	27·0 (15·6–39·7)	1·62 (1·15–2·16)	60·6 (41·9–82·1)	23·9 (16·6–32·8)	2·56 (1·96–3·24)
Guinea	34·3 (19·9–49·7)	35·9 (18·6–54·0)	0·981 (0·696–1·42)	39·3 (26·1–57·0)	33·6 (21·4–48·0)	1·19 (0·885–1·66)
Guinea-Bissau	53·7 (25·9–81·1)	34·1 (15·8–53·2)	1·61 (1·11–2·32)	51·0 (33·2–72·8)	33·0 (19·7–47·3)	1·56 (1·23–2·05)
Liberia	33·5 (22·5–46·4)	31·9 (20·1–44·6)	1·06 (0·848–1·31)	27·2 (18·5–39·2)	24·2 (15·3–34·5)	1·14 (0·825–1·64)
Mali	16·1 (7·81–25·0)	15·4 (6·48–24·4)	1·07 (0·823–1·45)	12·9 (7·49–20·5)	13·2 (7·25–20·5)	0·997 (0·641–1·58)
Mauritania	35·8 (23·5–50·0)	30·2 (20·1–43·0)	1·20 (0·910–1·56)	31·1 (20·7–44·4)	29·0 (18·6–41·3)	1·09 (0·804–1·49)
Niger	35·0 (16·0–54·5)	28·1 (9·83–47·7)	1·30 (0·850–1·97)	28·5 (16·7–43·8)	23·6 (12·1–37·1)	1·23 (0·914–1·70)
Nigeria	23·7 (13·5–34·2)	22·0 (12·2–31·9)	1·10 (0·760–1·55)	24·5 (15·2–34·5)	24·1 (14·5–34·7)	1·04 (0·655–1·52)
Sao Tome and Principe	41·1 (29·1–56·1)	34·7 (24·1–46·8)	1·19 (0·960–1·49)	56·1 (40·5–73·4)	48·6 (33·7–69·2)	1·18 (0·854–1·57)
Senegal	26·2 (13·4–38·6)	23·3 (11·8–34·0)	1·14 (0·864–1·47)	25·4 (16·4–37·0)	21·8 (13·6–31·5)	1·18 (0·850–1·64)
Sierra Leone	36·1 (22·5–50·2)	28·8 (16·0–41·6)	1·27 (0·961–1·62)	33·4 (22·2–47·7)	28·6 (18·5–41·0)	1·18 (0·858–1·65)
Togo	32·3 (20·8–45·2)	29·8 (17·9–41·7)	1·10 (0·840–1·44)	42·0 (28·2–59·5)	28·5 (18·5–40·5)	1·50 (1·12–2·14)

Appendix Table 7. Population-attributable fractions of lower respiratory infections due to evaluated risk factors among children younger than 5 years in 2019, males (M) and females (F) (95% UI)

	Child wasting		Child stunting		Household air pollution		Low birth weight		Handwashing		Short gestation		Ambient particulate matter		Child underweight		Non-exclusive breastfeeding		Secondhand smoke		High temperature		Low temperature	
	M	F	M	F	M	F	M	F	M	F	M	F	M	F	M	F	M	F	M	F	M	F	M	F
**Global**	**53.0 (37.7–61.8)**	**56.4 (40.7–65.1)**	**11.4 (1.0–28.9)**	**12.7 (1.1–32.1)**	**31.4 (21.5–41.5)**	**31.2 (21.3–41.5)**	**22.7 (20.5–24.9)**	**20.2 (18.3–22.6)**	**17.0 (7.7–25.4)**	**17.0 (7.7–25.4)**	**16.2 (14.7–17.9)**	**15.2 (13.7–17.0)**	**13.6 (9.3–19.1)**	**14.3 (9.7–20.1)**	**9.9 (6.4–17.9)**	**11.0 (7.3–19.7)**	**7.3 (4.0–11.0)**	**7.4 (4.0–11.2)**	**6.9 (3.7–10.1)**	**7.0 (3.8–10.3)**	**5.9 (2.0–15.0)**	**6.1 (2.5–14.7)**	**2.7 (<0.1–5.6)**	**2.7 (<0.1–5.9)**
**Central Europe, Eastern Europe, and Central Asia**	**51.3 (32.6–62.3)**	**54.6 (35.9–65.1)**	**7.0 (0.2–21.1)**	**7.0 (0.2–21.2)**	**3.2 (1.3–5.9)**	**3.0 (1.2–5.8)**	**13.7 (12.3–15.3)**	**11.3 (10.2–12.5)**	**4.5 (1.8–7.2)**	**4.4 (1.8–7.2)**	**11.5 (10.2–12.8)**	**8.3 (7.4–9.3)**	**14.6 (8.6–22.9)**	**14.4 (8.2–22.7)**	**3.1 (1.6–6.6)**	**4.7 (2.8–9.5)**	**9.8 (5.3–14.9)**	**10.7 (5.9–16.1)**	**14.0 (9.0–19.3)**	**14.4 (9.2–19.7)**	**0.8 (<0.1–1.8)**	**0.8 (<0.1–1.8)**	**18.2 (13.0–24.2)**	**18.1 (13.1–23.8)**
Central Asia	51.5 (33.2–62.2)	55.2 (36.9–65.3)	7.0 (0.2–20.7)	7.1 (0.2–21.5)	3.6 (1.5–6.6)	3.3 (1.4–6.5)	13.8 (12.3–15.6)	11.2 (10.0–12.6)	4.9 (2.0–7.9)	4.8 (1.9–7.8)	11.7 (10.4–13.3)	8.1 (7.1–9.2)	15.9 (9.2–25.0)	15.6 (8.6–25.0)	3.2 (1.6–6.9)	5.0 (3.0–10.2)	9.5 (4.8–14.7)	10.6 (5.5–16.4)	13.0 (8.2–18.1)	13.5 (8.6–18.6)	0.9 (<0.1–2.0)	0.9 (<0.1–2.1)	18.1 (13.3–23.6)	18.0 (13.3–23.1)
Armenia	39.4 (18.2–55.2)	48.8 (27.2–62.6)	6.3 (0.1–20.1)	8.8 (0.5–24.6)	0.5 (0.2–1.1)	0.5 (0.1–1.1)	13.6 (11.2–16.2)	12.8 (10.4–15.5)	3.8 (1.5–6.2)	3.8 (1.5–6.2)	10.8 (8.8–13.0)	9.3 (7.4–11.6)	17.1 (9.9–26.9)	17.0 (9.9–26.9)	2.1 (0.9–4.6)	2.0 (0.8–4.5)	7.6 (4.7–11.1)	9.1 (5.7–13.3)	23.2 (15.6–30.7)	23.0 (15.8–30.3)	<0.1 (<0.1–0.2)	<0.1 (<0.1–0.2)	18.4 (8.4–31.6)	18.4 (8.4–31.6)
Azerbaijan	43.8 (25.2–56.5)	47.5 (29.0–60.7)	5.9 (0.1–18.2)	5.7 (0.2–17.8)	1.3 (0.4–2.9)	1.3 (0.4–3.0)	19.3 (14.6–25.0)	15.6 (11.4–20.4)	3.5 (1.4–5.7)	3.5 (1.4–5.7)	15.1 (11.1–19.9)	11.0 (7.7–15.0)	13.4 (7.2–22.5)	13.3 (6.8–22.5)	2.8 (1.2–6.5)	4.5 (2.4–9.5)	10.9 (6.7–16.2)	13.9 (8.4–20.1)	21.0 (14.4–28.1)	21.2 (14.5–28.2)	0.5 (<0.1–1.1)	0.5 (<0.1–1.1)	15.7 (12.6–19.1)	15.7 (12.6–19.1)
Georgia	53.7 (32.7–68.6)	46.4 (23.9–63.6)	6.8 (0.1–21.7)	7.1 (0.2–23.2)	5.2 (1.7–11.8)	5.2 (1.6–11.8)	6.6 (4.2–9.7)	4.7 (3.0–6.9)	3.3 (1.3–5.4)	3.3 (1.3–5.4)	5.9 (3.8–8.7)	3.8 (2.4–5.7)	9.1 (5.6–14.0)	9.1 (5.5–13.9)	2.1 (1.0–4.7)	2.2 (1.0–5.1)	9.8 (6.6–13.3)	8.8 (5.8–12.0)	22.5 (15.6–29.7)	22.4 (15.2–29.8)	<0.1 (<0.1–0.2)	<0.1 (<0.1–0.2)	18.9 (11.2–29.0)	18.9 (11.2–29.0)
Kazakhstan	45.4 (27.1–57.9)	48.9 (30.4–61.3)	4.4 (0.1–15.0)	5.0 (0.1–16.2)	1.7 (0.5–4.0)	1.7 (0.5–4.0)	17.9 (14.1–22.3)	13.3 (10.3–16.8)	3.4 (1.4–5.7)	3.4 (1.4–5.7)	15.2 (11.8–19.1)	9.7 (7.2–12.5)	10.8 (6.1–17.0)	10.6 (6.0–16.7)	2.1 (1.0–4.4)	2.2 (1.1–4.8)	9.6 (5.0–15.1)	9.2 (4.8–14.4)	16.4 (9.9–22.7)	16.6 (10.3–22.8)	0.5 (<0.1–2.0)	0.5 (<0.1–2.0)	18.4 (11.3–27.6)	18.4 (11.3–27.6)
Kyrgyzstan	44.4 (20.2–62.3)	54.2 (30.7–69.8)	6.7 (0.2–21.2)	7.3 (0.2–23.1)	7.0 (2.9–13.6)	7.0 (2.9–13.6)	4.7 (3.7–5.8)	3.8 (3.0–4.8)	4.0 (1.6–6.5)	4.0 (1.6–6.5)	3.4 (2.7–4.3)	2.4 (1.9–3.1)	11.5 (6.4–18.6)	11.4 (6.3–18.6)	2.3 (1.1–5.3)	3.4 (1.8–7.4)	7.2 (4.0–11.1)	7.1 (3.9–11.0)	20.5 (13.4–27.7)	20.5 (13.8–27.2)	<0.1 (<0.1–<0.1)	<0.1 (<0.1–<0.1)	21.4 (8.4–37.4)	21.4 (8.4–37.4)
Mongolia	36.0 (17.5–51.2)	52.1 (28.8–68.8)	5.0 (0.1–16.8)	6.5 (0.1–20.1)	8.7 (3.7–16.8)	8.5 (3.4–17.0)	15.7 (11.5–20.9)	5.4 (3.5–7.8)	8.5 (3.6–13.6)	8.5 (3.6–13.6)	13.0 (9.2–17.4)	4.2 (2.7–6.4)	16.8 (11.1–24.0)	16.7 (10.8–24.1)	2.5 (1.3–5.3)	2.8 (1.5–6.2)	6.8 (4.1–10.0)	7.0 (4.2–10.4)	14.7 (8.5–21.1)	14.7 (8.5–21.1)	0.2 (<0.1–0.9)	0.2 (<0.1–0.9)	18.3 (3.6–34.9)	18.3 (3.6–34.9)
Tajikistan	63.1 (41.1–76.2)	65.4 (45.5–76.8)	11.0 (0.3–31.2)	11.3 (0.6–32.0)	9.6 (4.3–17.7)	9.6 (4.3–17.7)	9.0 (5.9–13.3)	7.8 (5.3–11.1)	7.1 (2.9–11.3)	7.1 (2.9–11.3)	6.5 (4.2–9.7)	5.3 (3.6–7.8)	16.2 (7.9–27.7)	16.2 (7.8–27.7)	5.5 (2.9–11.9)	7.5 (4.6–14.9)	8.3 (3.7–13.8)	8.2 (3.6–13.8)	11.6 (7.0–16.4)	13.5 (8.6–19.1)	0.3 (0.1–0.5)	0.3 (0.1–0.5)	21.4 (9.3–34.9)	21.4 (9.3–34.9)
Turkmenistan	58.5 (37.9–70.7)	58.1 (37.3–70.1)	5.7 (0.1–19.1)	6.7 (0.1–21.2)	<0.1 (<0.1–0.1)	<0.1 (<0.1–0.1)	6.8 (5.4–8.5)	5.3 (4.2–6.5)	4.6 (1.9–7.4)	4.6 (1.9–7.4)	6.4 (5.0–8.1)	5.4 (4.4–6.6)	13.7 (6.8–23.1)	13.5 (6.8–23.0)	4.7 (2.7–9.5)	4.6 (2.5–9.2)	6.9 (3.0–11.4)	6.8 (3.1–11.2)	20.7 (14.0–27.5)	20.7 (14.1–27.8)	2.6 (0.9–4.3)	2.6 (0.9–4.3)	12.7 (10.0–15.4)	12.7 (10.0–15.4)
Uzbekistan	50.0 (33.5–59.5)	54.6 (38.2–63.8)	6.3 (0.3–18.7)	6.5 (0.2–20.2)	2.3 (0.8–5.0)	2.2 (0.8–4.9)	15.5 (13.6–17.6)	12.5 (10.9–14.1)	4.5 (1.8–7.3)	4.5 (1.8–7.3)	13.8 (12.1–15.9)	9.0 (7.7–10.2)	17.2 (9.2–27.6)	16.8 (8.8–27.6)	2.5 (1.3–5.4)	5.0 (3.1–10.2)	10.3 (5.1–16.0)	11.6 (5.7–18.0)	9.8 (5.7–14.3)	9.9 (5.7–14.5)	1.0 (<0.1–2.7)	1.0 (<0.1–2.7)	18.0 (13.9–22.0)	18.0 (13.9–22.0)
Central Europe	49.9 (27.7–64.2)	46.5 (23.9–61.8)	6.6 (0.2–21.0)	6.9 (0.2–21.9)	1.9 (0.6–4.4)	2.0 (0.6–4.7)	12.3 (10.9–13.8)	11.4 (10.0–12.9)	1.2 (0.5–1.9)	1.2 (0.5–2.0)	8.5 (7.5–9.7)	8.4 (7.4–9.5)	9.2 (5.9–13.5)	9.2 (5.9–13.5)	2.7 (1.3–5.8)	2.7 (1.3–6.0)	11.7 (7.6–16.2)	10.5 (6.9–14.5)	18.0 (11.3–24.5)	18.3 (11.6–24.9)	<0.1 (<0.1–0.1)	<0.1 (<0.1–0.1)	20.7 (14.8–27.5)	20.7 (14.8–27.5)

	Child wasting		Child stunting		Household air pollution		Low birth weight		Handwashing		Short gestation		Ambient particulate matter		Child underweight		Non-exclusive breastfeeding		Secondhand smoke		High temperature		Low temperature	
	**M**	**F**	**M**	**F**	**M**	**F**	**M**	**F**	**M**	**F**	**M**	**F**	**M**	**F**	**M**	**F**	**M**	**F**	**M**	**F**	**M**	**F**	**M**	**F**
Albania	49.0 (25.8–65.4)	39.5 (17.5–57.5)	15.1 (1.4–37.8)	12.9 (0.9–34.0)	5.1 (1.7–11.2)	5.1 (1.7–11.3)	8.5 (5.2–13.3)	5.2 (2.2–8.7)	1.7 (0.7–2.9)	1.7 (0.7–2.9)	7.2 (4.3–11.4)	5.2 (2.3–8.5)	9.5 (6.1–14.1)	9.5 (6.0–14.1)	5.5 (3.3–11.0)	4.5 (2.6–9.4)	8.7 (4.3–13.9)	2.4 (0.8–4.9)	20.8 (14.0–28.1)	20.9 (14.2–28.0)	<0.1 (<0.1–0.1)	<0.1 (<0.1–0.1)	19.2 (14.6–24.1)	19.2 (14.6–24.1)
Bosnia and Herzegovina	24.6 (10.4–39.3)	25.5 (11.1–39.9)	3.6 (0.1–11.6)	4.2 (0.3–13.4)	5.4 (1.9–11.0)	5.4 (1.9–11.0)	26.3 (17.5–36.2)	25.3 (17.4–34.9)	1.3 (0.5–2.1)	1.3 (0.5–2.1)	24.4 (16.6–33.2)	24.1 (16.9–33.0)	14.7 (9.7–20.1)	14.8 (10.0–20.4)	1.0 (0.4–2.2)	1.3 (0.6–2.6)	17.7 (11.9–24.6)	16.6 (11.1–22.5)	23.3 (15.5–30.8)	23.2 (15.6–30.8)	<0.1 (<0.1–<0.1)	<0.1 (<0.1–<0.1)	22.3 (15.8–29.9)	22.3 (15.8–29.9)
Bulgaria	49.6 (25.7–65.1)	50.0 (26.1–66.0)	6.4 (0.1–20.2)	6.8 (0.3–21.1)	2.2 (0.6–5.2)	2.2 (0.6–5.2)	11.0 (8.7–13.9)	10.0 (7.9–12.7)	1.1 (0.4–1.8)	1.1 (0.4–1.8)	6.8 (5.3–8.5)	6.7 (5.2–8.5)	10.1 (6.4–14.8)	10.0 (6.3–14.8)	3.5 (1.8–7.5)	3.9 (2.1–8.4)	11.3 (7.2–15.7)	10.6 (6.7–15.0)	22.6 (14.8–30.3)	22.7 (14.9–30.4)	<0.1 (<0.1–<0.1)	<0.1 (<0.1–<0.1)	21.3 (14.8–27.9)	21.3 (14.8–27.9)
Croatia	42.1 (22.1–56.0)	42.3 (22.0–56.9)	4.8 (0.2–15.5)	5.7 (0.2–17.9)	0.6 (0.1–1.6)	0.6 (0.2–1.7)	16.2 (11.9–20.9)	16.1 (11.7–21.5)	1.0 (0.4–1.6)	1.0 (0.4–1.6)	13.4 (10.0–17.5)	13.7 (9.8–18.2)	10.0 (6.2–14.5)	10.0 (6.3–14.7)	1.5 (0.7–3.4)	2.3 (1.1–5.2)	10.2 (6.4–14.3)	11.0 (7.1–15.7)	20.6 (13.5–28.1)	21.0 (13.8–28.1)	<0.1 (<0.1–0.1)	<0.1 (<0.1–0.1)	20.6 (13.3–27.4)	20.6 (13.3–27.4)
Czechia	43.5 (23.8–57.7)	35.6 (18.2–49.3)	2.9 (0.0–10.9)	2.5 (0.0–9.2)	0.1 (<0.1–0.4)	0.1 (<0.1–0.4)	18.0 (13.2–23.0)	24.4 (18.5–30.4)	0.9 (0.3–1.4)	0.9 (0.3–1.4)	13.6 (10.1–17.3)	18.9 (14.6–23.8)	9.1 (5.6–13.6)	9.1 (5.7–13.5)	1.5 (0.6–3.4)	1.3 (0.5–3.1)	11.7 (8.6–15.3)	10.5 (7.6–13.7)	17.3 (11.2–23.7)	16.8 (10.6–23.2)	<0.1 (<0.1–0.2)	<0.1 (<0.1–0.2)	21.1 (13.6–30.0)	21.1 (13.6–30.0)
Hungary	43.3 (23.3–57.5)	40.9 (21.4–54.9)	5.0 (0.2–16.4)	5.6 (0.1–17.4)	1.9 (0.5–4.9)	1.9 (0.5–5.0)	19.1 (14.8–23.8)	19.5 (14.7–24.4)	0.9 (0.4–1.4)	0.9 (0.4–1.4)	14.8 (11.4–18.5)	16.0 (11.9–20.3)	8.7 (5.7–12.8)	8.8 (5.6–13.0)	1.2 (0.5–2.9)	1.6 (0.7–3.6)	10.9 (7.1–15.3)	10.0 (6.5–14.0)	19.4 (12.3–26.8)	19.5 (12.7–26.4)	<0.1 (<0.1–0.2)	<0.1 (<0.1–0.2)	21.4 (11.9–30.6)	21.4 (11.9–30.6)
Montenegro	44.4 (23.6–59.6)	49.5 (28.2–64.7)	5.6 (0.2–17.4)	5.6 (0.3–17.0)	3.7 (1.0–8.9)	3.7 (1.0–8.9)	14.6 (10.0–20.2)	8.8 (5.6–13.5)	1.2 (0.5–2.0)	1.2 (0.5–2.0)	11.5 (8.0–15.8)	9.7 (6.2–14.7)	11.1 (7.1–15.9)	11.2 (7.2–16.2)	1.1 (0.4–2.5)	1.1 (0.5–2.6)	12.2 (8.3–16.3)	14.5 (10.2–19.0)	23.0 (15.4–30.4)	23.2 (15.7–30.8)	<0.1 (<0.1–0.1)	<0.1 (<0.1–0.1)	20.4 (14.1–28.1)	20.4 (14.1–28.1)
North Macedonia	49.0 (24.8–65.5)	41.1 (19.4–58.9)	4.2 (0.1–14.5)	4.8 (0.1–15.8)	3.2 (1.0–7.4)	3.3 (1.0–7.4)	8.1 (5.3–11.7)	10.2 (6.8–14.5)	1.0 (0.4–1.7)	1.0 (0.4–1.7)	5.8 (3.8–8.3)	7.5 (5.0–10.9)	15.1 (10.0–21.4)	15.1 (10.1–21.4)	1.3 (0.5–3.0)	1.3 (0.5–2.9)	12.7 (8.4–17.4)	13.7 (9.3–18.5)	22.8 (14.8–30.5)	22.8 (15.1–30.3)	<0.1 (<0.1–0.1)	<0.1 (<0.1–0.1)	21.8 (15.6–28.1)	21.8 (15.6–28.1)
Poland	47.2 (24.9–62.2)	45.2 (23.6–60.3)	5.2 (0.1–17.3)	5.9 (0.3–18.7)	1.4 (0.4–3.8)	1.4 (0.4–3.8)	11.4 (10.1–12.9)	11.7 (10.4–13.4)	1.0 (0.4–1.6)	1.0 (0.4–1.6)	8.8 (7.8–10.0)	9.7 (8.5–11.0)	11.8 (7.8–17.1)	11.8 (7.7–16.9)	1.6 (0.7–3.6)	2.2 (1.1–4.8)	8.9 (5.8–12.2)	8.8 (5.8–12.0)	16.0 (9.8–22.6)	16.2 (10.1–22.4)	<0.1 (<0.1–0.2)	<0.1 (<0.1–0.2)	21.1 (13.3–30.0)	21.1 (13.3–30.1)
Romania	51.5 (29.3–65.2)	48.3 (26.0–63.4)	5.9 (0.1–19.8)	6.2 (<0.1–20.8)	1.5 (0.4–3.7)	1.5 (0.4–3.7)	12.7 (10.9–14.9)	12.1 (10.2–14.3)	1.2 (0.5–2.0)	1.2 (0.5–2.0)	8.4 (7.0–10.0)	8.3 (7.0–10.1)	8.2 (5.1–12.2)	8.1 (5.0–12.3)	2.6 (1.2–5.9)	2.4 (1.1–5.6)	12.8 (8.2–17.7)	12.5 (8.0–17.1)	17.0 (10.3–23.5)	17.4 (10.6–24.1)	<0.1 (<0.1–0.1)	<0.1 (<0.1–0.1)	20.7 (14.2–28.2)	20.7 (14.2–28.2)
Serbia	47.8 (27.3–61.7)	47.5 (27.1–62.6)	4.0 (0.1–13.5)	5.2 (0.2–15.7)	2.7 (0.7–6.4)	2.7 (0.8–6.4)	15.8 (10.8–22.0)	15.4 (10.6–21.1)	1.0 (0.4–1.6)	1.0 (0.4–1.6)	11.8 (8.0–16.5)	12.6 (8.5–17.6)	13.1 (8.7–18.9)	13.2 (8.6–19.0)	1.5 (0.6–3.3)	2.4 (1.2–5.1)	15.0 (10.2–20.2)	14.8 (9.9–20.0)	19.0 (12.0–25.7)	19.2 (12.1–26.2)	<0.1 (<0.1–0.1)	<0.1 (<0.1–0.1)	21.9 (14.8–28.9)	21.9 (14.8–28.9)
Slovakia	50.8 (28.0–66.5)	50.1 (26.9–66.1)	5.9 (0.2–18.8)	6.9 (0.2–21.5)	0.1 (<0.1–0.3)	0.1 (<0.1–0.3)	9.6 (6.7–13.3)	8.7 (5.9–12.3)	0.9 (0.3–1.4)	0.9 (0.3–1.4)	6.3 (4.4–8.7)	6.1 (4.1–8.5)	9.6 (5.9–14.4)	9.6 (5.8–14.5)	1.8 (0.8–3.9)	2.8 (1.5–6.3)	8.3 (5.4–11.5)	8.1 (5.3–11.3)	14.3 (8.7–20.3)	15.4 (9.3–21.7)	<0.1 (<0.1–0.1)	<0.1 (<0.1–0.1)	20.2 (13.2–29.4)	20.2 (13.2–29.4)
Slovenia	42.9 (22.4–58.1)	42.6 (22.5–58.0)	4.7 (0.1–15.6)	5.4 (0.1–17.0)	0.8 (0.2–2.1)	0.8 (0.2–2.2)	15.1 (11.2–19.6)	16.2 (11.9–21.3)	0.8 (0.3–1.4)	0.8 (0.3–1.4)	11.8 (8.7–15.2)	13.1 (9.6–17.4)	9.0 (5.4–13.4)	9.2 (5.7–13.6)	1.4 (0.6–3.3)	2.1 (1.0–4.7)	9.0 (5.8–12.7)	11.7 (7.6–16.2)	18.2 (12.2–24.4)	18.5 (12.2–25.4)	<0.1 (<0.1–0.1)	<0.1 (<0.1–0.1)	22.1 (15.2–30.1)	22.1 (15.2–30.1)

	**Child wasting**		**Child stunting**		**Household air pollution**		**Low birth weight**		**Handwashing**		**Short gestation**		**Ambient particulate matter**		**Child underweight**		**Non-exclusive breastfeeding**		**Secondhand smoke**		**High temperature**		**Low temperature**	
	**M**	**F**	**M**	**F**	**M**	**F**	**M**	**F**	**M**	**F**	**M**	**F**	**M**	**F**	**M**	**F**	**M**	**F**	**M**	**F**	**M**	**F**	**M**	**F**
Eastern Europe	50.3 (29.1–63.1)	52.6 (31.3–65.3)	7.9 (0.3–23.2)	6.2 (0.1–20.1)	0.3 (0.1–0.8)	0.3 (0.1–0.8)	13.6 (12.4–14.9)	12.4 (11.2–13.7)	2.4 (1.0–3.9)	2.4 (1.0–4.0)	10.7 (9.7–11.7)	10.5 (9.4–11.6)	6.1 (3.7–9.7)	6.1 (3.7–9.7)	2.2 (1.0–5.1)	2.3 (1.0–5.4)	11.5 (8.1–15.1)	11.6 (8.1–15.2)	20.7 (13.6–27.5)	20.9 (13.7–28.0)	<0.1 (<0.1–0.4)	<0.1 (<0.1–0.3)	17.2 (2.7–29.9)	17.3 (3.2–29.7)
Belarus	41.7 (21.0–57.7)	40.6 (19.9–57.3)	2.7 (0.0–9.5)	3.8 (<0.1–12.4)	0.1 (<0.1–0.3)	0.1 (<0.1–0.3)	13.6 (10.0–17.8)	12.4 (9.1–16.1)	1.9 (0.8–3.1)	1.9 (0.8–3.1)	11.6 (8.4–15.2)	11.5 (8.3–15.1)	8.8 (5.3–13.4)	8.9 (5.3–13.8)	1.4 (0.6–3.2)	0.3 (0.1–0.9)	12.2 (8.6–16.1)	12.6 (8.8–16.6)	20.8 (13.8–27.7)	20.5 (13.5–27.5)	<0.1 (<0.1–0.2)	<0.1 (<0.1–0.2)	18.3 (9.6–30.8)	18.3 (9.6–30.8)
Estonia	46.6 (26.3–60.4)	50.2 (29.5–64.1)	4.4 (0.1–15.0)	3.9 (0.0–13.7)	0.7 (0.1–2.0)	0.7 (0.1–2.1)	14.4 (10.9–18.5)	12.7 (9.7–16.1)	1.8 (0.7–3.0)	1.8 (0.7–3.0)	12.0 (9.0–15.4)	11.1 (8.4–14.2)	2.5 (0.9–4.9)	2.4 (0.9–4.5)	1.7 (0.7–3.9)	1.9 (0.8–4.5)	9.7 (6.0–13.7)	9.6 (5.9–13.6)	16.5 (10.6–22.9)	17.2 (11.0–23.3)	0.1 (<0.1–0.5)	0.1 (<0.1–0.5)	15.8 (3.4–32.6)	15.8 (3.4–32.6)
Latvia	37.1 (20.9–50.5)	41.1 (24.0–54.2)	3.8 (0.1–12.8)	3.6 (0.1–12.6)	0.7 (0.1–1.9)	0.7 (0.1–1.7)	23.6 (18.3–29.0)	20.7 (16.0–25.9)	1.9 (0.8–3.1)	1.9 (0.8–3.1)	20.4 (15.7–25.4)	18.9 (14.6–23.7)	6.7 (3.6–11.2)	6.7 (3.5–11.0)	1.4 (0.6–3.2)	1.7 (0.8–3.8)	8.5 (5.2–12.2)	7.8 (4.8–11.4)	16.0 (9.6–22.7)	16.5 (9.9–23.2)	<0.1 (<0.1–0.3)	<0.1 (<0.1–0.3)	17.2 (7.2–31.3)	17.2 (7.2–31.3)
Lithuania	44.9 (24.2–58.7)	45.7 (25.5–58.5)	4.5 (0.1–15.0)	3.6 (<0.1–12.8)	0.3 (0.1–0.8)	0.3 (0.1–0.8)	14.9 (11.3–19.1)	16.2 (12.6–20.9)	1.9 (0.8–3.2)	1.9 (0.8–3.2)	12.0 (9.0–15.4)	13.9 (10.8–18.0)	5.2 (2.7–8.5)	5.3 (2.7–8.7)	1.7 (0.7–3.7)	1.8 (0.8–3.9)	8.3 (5.2–12.0)	8.9 (5.6–12.8)	15.3 (9.2–21.4)	15.6 (9.6–21.8)	<0.1 (<0.1–0.2)	<0.1 (<0.1–0.2)	19.1 (9.2–31.0)	19.1 (9.2–31.0)
Republic of Moldova	19.6 (8.9–28.6)	26.0 (13.3–35.5)	2.0 (0.0–7.2)	2.2 (<0.1–7.9)	1.3 (0.4–2.9)	1.2 (0.4–2.8)	34.0 (29.0–39.4)	30.3 (25.3–35.7)	4.6 (1.9–7.4)	4.6 (1.9–7.4)	27.7 (23.4–32.0)	26.8 (22.9–31.2)	8.2 (3.9–13.9)	7.9 (3.9–13.2)	1.2 (0.5–2.7)	1.1 (0.5–2.5)	9.3 (6.0–13.0)	9.7 (6.4–13.5)	19.5 (13.2–26.1)	18.9 (12.7–25.3)	<0.1 (<0.1–0.1)	<0.1 (<0.1–0.1)	22.1 (13.3–30.8)	22.1 (13.3–30.8)
Russian Federation	52.6 (30.9–65.8)	54.1 (32.4–67.1)	8.3 (0.3–24.2)	5.5 (0.1–18.8)	0.2 (0.1–0.6)	0.2 (0.1–0.6)	12.2 (11.0–13.3)	11.9 (10.8–12.9)	2.2 (0.9–3.6)	2.2 (0.9–3.6)	9.4 (8.4–10.2)	9.8 (8.9–10.7)	5.7 (3.2–9.2)	5.6 (3.2–9.1)	2.3 (1.0–5.2)	2.5 (1.1–6.0)	12.1 (8.5–15.7)	11.9 (8.3–15.3)	21.1 (13.8–28.1)	21.4 (14.2–28.6)	<0.1 (<0.1–0.4)	<0.1 (<0.1–0.4)	16.4 (<0.1–31.1)	16.5 (<0.1–31.1)
Ukraine	50.2 (30.0–63.1)	55.0 (33.2–69.7)	8.9 (0.7–25.0)	11.9 (0.9–32.4)	0.5 (0.1–1.3)	0.5 (0.1–1.3)	13.6 (10.6–17.3)	9.0 (5.9–12.8)	3.0 (1.2–5.0)	3.0 (1.2–5.0)	11.5 (8.9–14.5)	8.3 (5.4–11.9)	7.6 (4.2–12.5)	7.5 (4.1–12.7)	2.3 (1.1–4.9)	2.0 (0.9–4.5)	8.6 (5.3–12.2)	11.3 (7.3–15.9)	19.4 (12.3–26.0)	19.2 (12.4–26.3)	<0.1 (<0.1–0.2)	<0.1 (<0.1–0.2)	19.7 (13.4–26.1)	19.7 (13.5–26.1)
**High-income**	**40.7 (18.0–58.0)**	**40.4 (17.8–58.3)**	**3.4 (0.0–12.0)**	**3.4 (0.0–12.3)**	**0.2 (<0.1–0.4)**	**0.2 (<0.1–0.4)**	**9.4 (8.4–10.4)**	**9.9 (8.8–10.9)**	**1.6 (0.6–2.6)**	**1.6 (0.6–2.6)**	**7.5 (6.8–8.3)**	**8.1 (7.2–9.0)**	**5.4 (3.3–8.5)**	**5.5 (3.4–8.6)**	**2.2 (0.9–5.2)**	**2.2 (0.9–5.1)**	**10.0 (7.4–12.6)**	**9.4 (7.0–11.9)**	**13.9 (8.6–19.7)**	**14.2 (8.8–19.8)**	**0.7 (0.4–1.1)**	**0.7 (0.4–1.1)**	**16.2 (11.8–21.2)**	**16.4 (11.9–21.4)**
Australasia	46.1 (23.2–62.1)	44.8 (22.9–60.4)	3.4 (0.0–12.7)	3.2 (0.0–11.9)	<0.1 (<0.1–0.1)	<0.1 (<0.1–0.1)	11.4 (8.8–14.3)	13.6 (10.7–16.6)	1.9 (0.8–3.3)	1.9 (0.8–3.3)	9.9 (7.6–12.4)	12.2 (9.6–15.1)	2.5 (0.9–4.9)	2.7 (0.9–5.2)	2.0 (0.8–4.7)	1.9 (0.8–4.4)	9.8 (7.1–12.7)	9.9 (7.2–12.8)	12.1 (7.1–17.5)	12.3 (7.3–17.7)	5.8 (3.9–7.8)	5.6 (3.7–7.5)	8.8 (5.8–11.9)	9.3 (6.2–12.4)
Australia	47.8 (23.9–64.7)	46.6 (23.4–62.9)	3.5 (0.0–13.2)	3.3 (0.0–12.4)	<0.1 (<0.1–0.1)	<0.1 (<0.1–0.1)	9.4 (6.8–12.5)	11.4 (8.3–14.9)	1.9 (0.8–3.2)	1.9 (0.8–3.2)	7.9 (5.7–10.6)	10.2 (7.3–13.5)	2.4 (0.7–4.9)	2.7 (0.7–5.4)	2.1 (0.8–4.8)	2.0 (0.8–4.5)	9.9 (7.0–13.1)	10.2 (7.2–13.5)	12.2 (7.0–17.8)	12.5 (7.4–18.0)	7.3 (4.8–9.7)	7.3 (4.8–9.7)	5.9 (2.7–8.9)	5.9 (2.7–8.9)
New Zealand	39.5 (20.0–53.0)	38.9 (19.8–52.5)	2.9 (0.0–10.9)	2.9 (0.0–10.5)	<0.1 (<0.1–0.1)	<0.1 (<0.1–0.1)	19.5 (15.6–24.1)	20.8 (16.8–25.1)	2.0 (0.8–3.3)	2.0 (0.8–3.3)	17.7 (14.2–22.0)	18.9 (15.2–23.2)	2.8 (0.6–6.3)	2.7 (0.6–6.4)	1.7 (0.7–4.0)	1.7 (0.6–3.8)	9.2 (6.7–11.8)	8.9 (6.4–11.5)	11.7 (6.6–17.2)	11.6 (6.7–16.8)	<0.1 (<0.1–<0.1)	<0.1 (<0.1–<0.1)	20.5 (13.3–27.8)	20.5 (13.3–27.8)
High-income Asia Pacific	48.1 (23.6–65.4)	49.2 (24.7–66.3)	5.3 (0.1–17.9)	5.6 (0.1–18.7)	<0.1 (<0.1–<0.1)	<0.1 (<0.1–<0.1)	7.9 (6.9–8.9)	8.0 (6.9–9.0)	1.9 (0.7–3.1)	1.9 (0.7–3.1)	5.4 (4.7–6.1)	5.2 (4.4–5.9)	8.0 (4.8–12.5)	8.1 (4.7–12.6)	3.6 (1.8–7.7)	3.5 (1.7–7.6)	6.6 (4.4–8.9)	6.5 (4.4–9.0)	17.0 (11.0–23.2)	16.7 (10.7–23.0)	0.5 (<0.1–1.3)	0.5 (<0.1–1.4)	15.1 (12.0–18.4)	15.2 (12.1–18.5)

	Child wasting		Child stunting		Household air pollution		Low birth weight		Handwashing		Short gestation		Ambient particulate matter		Child underweight		Non-exclusive breastfeeding		Secondhand smoke		High temperature		Low temperature	
	M	F	M	F	M	F	M	F	M	F	M	F	M	F	M	F	M	F	M	F	M	F	M	F
Brunei Darussalam	37.1 (16.2–52.8)	50.4 (29.1–64.1)	6.8 (0.2–20.9)	7.3 (0.3–22.1)	0.1 (<0.1–0.2)	0.1 (<0.1–0.2)	17.4 (12.7–23.2)	16.9 (12.1–22.5)	2.0 (0.8–3.2)	2.0 (0.8–3.2)	12.2 (8.9–16.3)	11.2 (8.0–15.2)	3.4 (1.1–6.7)	3.3 (1.1–6.1)	6.3 (3.9–12.8)	6.5 (4.0–13.0)	7.5 (4.8–10.4)	8.2 (5.3–11.5)	14.4 (9.2–20.3)	14.6 (8.9–20.1)	0.9 (<0.1–5.8)	0.9 (<0.1–5.8)	<0.1 (<0.1–0.5)	<0.1 (<0.1–0.5)
Japan	51.5 (26.0–68.6)	51.4 (26.1–68.5)	6.3 (0.1–21.2)	6.3 (0.1–21.1)	<0.1 (<0.1–<0.1)	<0.1 (<0.1–<0.1)	6.2 (5.0–6.8)	6.4 (5.3–7.1)	1.9 (0.7–3.1)	1.9 (0.7–3.1)	4.0 (3.3–4.5)	4.0 (3.3–4.5)	6.7 (3.8–10.7)	6.6 (3.8–10.7)	4.0 (2.0–8.6)	4.0 (2.0–8.6)	6.2 (4.0–8.5)	6.1 (4.0–8.4)	17.6 (11.4–24.0)	17.3 (11.1–23.8)	0.5 (<0.1–1.0)	0.5 (<0.1–1.1)	15.9 (12.8–19.2)	15.8 (12.8–19.1)
Republic of Korea	35.0 (13.3–53.8)	38.1 (15.0–56.7)	2.1 (0.0–8.3)	3.2 (0.0–12.1)	<0.1 (<0.1–<0.1)	<0.1 (<0.1–<0.1)	11.7 (7.9–16.3)	11.3 (7.5–15.9)	1.9 (0.7–3.1)	1.9 (0.7–3.1)	8.0 (5.4–11.3)	7.6 (5.0–10.8)	14.4 (8.9–21.2)	14.3 (8.9–21.1)	1.7 (0.5–4.2)	1.5 (0.4–4.0)	6.2 (3.8–9.1)	6.1 (3.8–9.0)	17.0 (10.6–23.6)	16.7 (10.5–23.0)	0.2 (<0.1–0.9)	0.2 (<0.1–0.9)	20.4 (14.7–25.9)	20.4 (14.7–25.9)
Singapore	47.0 (23.8–61.9)	52.2 (28.7–66.7)	2.2 (0.0–8.4)	3.2 (0.0–12.3)	<0.1 (<0.1–0.1)	<0.1 (<0.1–0.1)	14.9 (11.5–19.1)	13.5 (10.4–17.1)	1.9 (0.8–3.1)	1.9 (0.8–3.1)	11.1 (8.6–14.2)	9.3 (7.2–12.0)	10.0 (5.5–16.2)	9.8 (5.6–16.3)	2.3 (1.0–5.2)	2.1 (0.9–4.9)	12.4 (8.9–15.8)	12.1 (8.8–15.5)	10.7 (6.2–15.6)	11.2 (6.4–16.1)	1.3 (<0.1–11.7)	1.3 (<0.1–11.7)	0.1 (<0.1–1.2)	0.1 (<0.1–1.2)
High-income North America	31.5 (10.5–50.7)	31.9 (11.0–51.0)	2.6 (0.0–10.2)	2.3 (0.0–8.7)	<0.1 (<0.1–<0.1)	<0.1 (<0.1–<0.1)	8.8 (8.0–9.5)	10.2 (9.3–11.0)	1.0 (0.4–1.6)	1.0 (0.4–1.6)	7.8 (7.1–8.5)	8.8 (8.1–9.6)	3.0 (1.7–5.3)	3.1 (1.7–5.2)	2.1 (0.8–5.0)	1.8 (0.7–4.5)	11.2 (8.5–14.3)	10.7 (8.2–13.6)	12.7 (8.3–17.9)	12.9 (8.3–18.2)	0.7 (0.3–1.1)	0.7 (0.3–1.1)	14.9 (11.2–18.7)	15.0 (11.3–18.9)
Canada	35.0 (12.9–53.9)	35.8 (13.1–55.1)	2.4 (0.0–9.5)	2.2 (0.0–8.5)	<0.1 (<0.1–<0.1)	<0.1 (<0.1–<0.1)	10.1 (7.3–13.7)	9.3 (6.8–12.5)	1.0 (0.4–1.7)	1.0 (0.4–1.7)	8.8 (6.4–11.7)	8.6 (6.2–11.6)	2.7 (1.1–5.1)	2.8 (1.1–5.2)	1.9 (0.7–4.5)	1.8 (0.6–4.3)	11.2 (8.0–14.8)	10.4 (7.5–13.9)	12.6 (8.0–17.9)	12.9 (8.3–18.4)	<0.1 (<0.1–0.3)	<0.1 (<0.1–0.3)	20.8 (0.8–39.3)	20.8 (0.8–39.3)
United States of America	31.3 (10.4–50.4)	31.6 (10.9–50.6)	2.6 (0.0–10.3)	2.3 (0.0–8.7)	<0.1 (<0.1–<0.1)	<0.1 (<0.1–<0.1)	8.7 (7.9–9.5)	10.2 (9.4–11.1)	1.0 (0.4–1.6)	1.0 (0.4–1.6)	7.7 (7.1–8.5)	8.9 (8.1–9.6)	3.1 (1.7–5.3)	3.1 (1.7–5.2)	2.1 (0.8–5.0)	1.8 (0.7–4.5)	11.2 (8.5–14.4)	10.7 (8.1–13.7)	12.7 (8.2–17.9)	12.9 (8.3–18.2)	0.7 (0.4–1.2)	0.7 (0.4–1.1)	14.6 (11.1–18.1)	14.7 (11.2–18.2)
Southern Latin America	48.8 (25.2–65.0)	48.3 (24.3–65.1)	4.8 (0.0–16.2)	4.7 (0.0–16.3)	0.5 (0.1–1.1)	0.5 (0.1–1.1)	8.5 (6.6–10.6)	8.4 (6.6–10.4)	2.4 (1.0–4.0)	2.4 (1.0–4.0)	6.3 (4.9–7.9)	6.5 (5.1–8.1)	7.2 (4.1–11.6)	7.3 (4.2–11.8)	2.5 (1.0–5.7)	2.5 (1.1–5.7)	9.8 (6.5–13.2)	9.2 (6.1–12.5)	14.3 (8.3–20.5)	14.7 (8.8–21.0)	0.8 (0.5–1.2)	0.8 (0.5–1.2)	16.0 (10.2–22.5)	16.1 (10.2–22.5)
Argentina	49.9 (26.2–66.2)	49.8 (25.4–66.9)	4.9 (0.0–16.6)	4.9 (0.0–16.8)	0.4 (0.1–1.1)	0.4 (0.1–1.1)	8.0 (6.0–10.2)	7.7 (5.7–9.8)	2.5 (1.0–4.1)	2.5 (1.0–4.1)	5.7 (4.2–7.4)	5.7 (4.2–7.4)	6.7 (3.7–11.2)	6.8 (3.7–11.3)	2.6 (1.1–5.9)	2.6 (1.1–5.9)	10.1 (6.6–13.7)	9.5 (6.2–13.0)	14.3 (8.4–20.6)	14.9 (8.8–21.2)	0.9 (0.5–1.4)	0.9 (0.5–1.4)	16.1 (10.5–22.4)	16.1 (10.5–22.4)
Chile	39.9 (18.4–55.9)	38.1 (17.2–55.2)	3.3 (0.0–12.6)	3.1 (0.0–11.5)	0.8 (0.2–1.9)	0.8 (0.2–1.9)	12.6 (9.5–16.2)	12.5 (9.5–16.0)	2.0 (0.8–3.4)	2.0 (0.8–3.4)	10.4 (7.7–13.6)	11.0 (8.3–14.3)	12.1 (7.7–18.1)	12.1 (7.6–18.2)	1.7 (0.7–4.1)	1.7 (0.6–3.9)	8.0 (5.4–10.9)	7.7 (5.1–10.4)	12.8 (6.7–19.3)	13.1 (7.0–19.6)	<0.1 (<0.1–<0.1)	<0.1 (<0.1–<0.1)	19.3 (10.4–29.4)	19.3 (10.4–29.4)
Uruguay	46.6 (22.8–63.3)	44.7 (21.8–60.6)	6.5 (0.1–20.6)	5.2 (0.1–17.9)	0.4 (0.1–0.9)	0.4 (0.1–0.9)	10.1 (7.8–13.0)	13.2 (10.3–16.6)	2.3 (0.9–3.7)	2.3 (0.9–3.7)	8.2 (6.4–10.6)	10.4 (8.0–13.2)	4.4 (2.0–7.9)	4.4 (2.2–7.9)	2.9 (1.3–6.3)	2.7 (1.2–5.9)	7.7 (5.2–10.7)	7.2 (4.8–10.2)	16.4 (10.2–23.1)	16.9 (10.4–23.4)	0.1 (<0.1–0.3)	0.1 (<0.1–0.3)	8.0 (4.5–11.6)	8.0 (4.5–11.6)
Western Europe	39.7 (17.6–56.0)	36.9 (15.4–53.9)	1.6 (0.0–6.4)	2.0 (0.0–7.8)	<0.1 (<0.1–0.1)	<0.1 (<0.1–0.1)	12.9 (11.2–14.7)	12.4 (10.8–14.3)	1.0 (0.4–1.7)	1.0 (0.4–1.7)	10.3 (8.9–11.8)	10.4 (9.1–12.0)	5.8 (3.7–8.9)	5.8 (3.7–8.9)	1.4 (0.5–3.4)	1.7 (0.6–3.9)	9.7 (7.2–12.3)	9.0 (6.7–11.5)	14.4 (9.1–20.0)	14.5 (8.9–20.0)	0.1 (<0.1–0.3)	0.1 (<0.1–0.3)	20.9 (14.6–27.4)	20.7 (14.6–27.2)
Andorra	40.6 (18.2–58.1)	38.6 (15.1–57.7)	1.6 (0.0–6.5)	2.1 (0.0–8.5)	<0.1 (<0.1–<0.1)	<0.1 (<0.1–<0.1)	11.1 (6.6–17.9)	7.8 (4.5–13.0)	1.0 (0.4–1.6)	1.0 (0.4–1.6)	8.9 (5.2–14.5)	6.5 (3.8–10.7)	4.2 (2.0–7.7)	4.0 (1.9–7.2)	1.4 (0.5–3.3)	1.7 (0.7–4.2)	6.8 (4.2–10.1)	4.8 (2.8–7.6)	15.6 (9.8–22.1)	15.7 (10.1–21.9)	<0.1 (<0.1–<0.1)	<0.1 (<0.1–<0.1)	17.0 (1.9–35.8)	17.0 (1.9–35.8)

	Child wasting		Child stunting		Household air pollution		Low birth weight		Handwashing		Short gestation		Ambient particulate matter		Child underweight		Non-exclusive breastfeeding		Secondhand smoke		High temperature		Low temperature	
	**M**	**F**	**M**	**F**	**M**	**F**	**M**	**F**	**M**	**F**	**M**	**F**	**M**	**F**	**M**	**F**	**M**	**F**	**M**	**F**	**M**	**F**	**M**	**F**
Austria	39.6 (17.7–55.7)	38.8 (16.6–55.8)	1.6 (0.0–6.1)	2.0 (0.0–7.8)	<0.1 (<0.1–0.1)	<0.1 (<0.1–0.1)	13.9 (10.1–18.7)	13.1 (9.5–17.6)	1.0 (0.4–1.6)	1.0 (0.4–1.6)	11.9 (8.6–16.1)	11.3 (8.2–15.3)	6.3 (3.5–10.4)	6.2 (3.6–9.8)	1.4 (0.5–3.4)	1.6 (0.7–3.9)	8.0 (5.2–11.0)	8.8 (5.9–11.9)	19.9 (13.4–26.7)	20.0 (13.6–26.9)	<0.1 (<0.1–0.1)	<0.1 (<0.1–0.1)	19.7 (10.6–31.5)	19.7 (10.6–31.5)
Belgium	43.3 (19.1–61.0)	40.4 (16.4–59.1)	1.7 (0.0–6.7)	2.2 (0.0–8.5)	<0.1 (<0.1–<0.1)	<0.1 (<0.1–<0.1)	10.5 (7.8–13.9)	7.8 (5.7–10.3)	1.0 (0.4–1.6)	1.0 (0.4–1.6)	7.8 (5.7–10.4)	6.2 (4.5–8.2)	6.3 (3.8–10.0)	6.3 (3.7–9.9)	1.5 (0.5–3.6)	1.8 (0.7–4.3)	8.7 (5.9–11.7)	6.3 (4.1–8.7)	14.9 (9.2–20.7)	14.8 (9.5–20.5)	<0.1 (<0.1–0.1)	<0.1 (<0.1–0.1)	23.4 (14.7–32.7)	23.4 (14.7–32.7)
Cyprus	36.9 (17.4–52.6)	34.5 (14.8–50.9)	1.5 (0.0–5.5)	1.9 (0.0–7.2)	<0.1 (<0.1–0.1)	<0.1 (<0.1–0.1)	24.1 (16.7–32.4)	22.5 (15.7–30.2)	1.0 (0.4–1.6)	1.0 (0.4–1.6)	14.9 (10.3–20.2)	14.6 (10.3–19.9)	8.2 (5.0–12.7)	8.2 (4.9–12.7)	1.3 (0.5–3.1)	1.6 (0.6–3.7)	10.5 (7.2–14.1)	9.3 (6.4–12.7)	18.4 (11.7–25.1)	18.3 (12.0–24.6)	2.1 (0.9–3.6)	2.1 (0.9–3.6)	4.5 (<0.1–10.9)	4.5 (<0.1–10.9)
Denmark	42.7 (19.5–60.5)	36.1 (15.8–52.9)	1.7 (0.0–6.7)	1.9 (0.0–7.4)	<0.1 (<0.1–<0.1)	<0.1 (<0.1–<0.1)	6.8 (4.6–9.3)	10.2 (7.1–13.9)	1.0 (0.4–1.6)	1.0 (0.4–1.6)	8.2 (5.8–11.2)	13.0 (9.2–17.4)	4.7 (2.4–8.0)	5.1 (2.4–9.0)	1.5 (0.5–3.5)	1.6 (0.6–3.8)	9.2 (6.3–12.5)	8.8 (6.1–11.8)	14.2 (8.9–19.9)	14.9 (9.5–20.5)	<0.1 (<0.1–<0.1)	<0.1 (<0.1–<0.1)	23.2 (13.0–34.4)	23.2 (13.0–34.4)
Finland	38.7 (18.5–54.3)	35.3 (15.4–51.3)	1.5 (0.0–6.3)	1.9 (0.0–7.1)	<0.1 (<0.1–0.1)	<0.1 (<0.1–0.1)	14.7 (11.0–19.0)	15.3 (11.6–19.6)	1.0 (0.4–1.7)	1.0 (0.4–1.7)	11.7 (8.8–15.3)	13.4 (10.1–17.3)	2.0 (0.3–4.6)	2.2 (0.3–5.1)	1.4 (0.5–3.3)	1.6 (0.6–3.7)	9.3 (6.5–12.5)	8.7 (5.9–11.8)	12.5 (7.5–17.6)	12.5 (7.5–17.3)	<0.1 (<0.1–0.3)	<0.1 (<0.1–0.3)	17.9 (3.8–36.4)	17.9 (3.8–36.4)
France	41.7 (19.1–58.9)	39.5 (16.7–56.9)	1.7 (0.0–6.6)	2.1 (0.0–8.1)	<0.1 (<0.1–0.1)	<0.1 (<0.1–<0.1)	9.2 (6.6–12.3)	7.9 (5.8–10.4)	1.0 (0.4–1.7)	1.0 (0.4–1.7)	7.8 (5.5–10.5)	7.1 (5.1–9.4)	5.7 (3.2–9.0)	5.6 (3.2–8.9)	1.5 (0.5–3.4)	1.7 (0.7–4.1)	8.2 (5.8–11.0)	7.2 (4.9–9.7)	13.6 (7.8–19.8)	13.9 (8.1–19.8)	<0.1 (<0.1–0.1)	<0.1 (<0.1–0.1)	21.7 (14.9–28.4)	21.7 (14.9–28.4)
Germany	32.5 (11.7–50.5)	30.5 (10.3–48.8)	1.4 (0.0–5.9)	2.0 (0.0–7.5)	<0.1 (<0.1–<0.1)	<0.1 (<0.1–<0.1)	10.8 (8.0–14.2)	9.0 (6.7–11.7)	1.0 (0.4–1.6)	1.0 (0.4–1.6)	9.4 (7.0–12.5)	8.3 (6.2–10.8)	5.9 (3.5–9.5)	5.9 (3.4–9.4)	1.7 (0.6–3.9)	1.7 (0.7–4.1)	9.1 (6.5–12.3)	7.8 (5.4–10.6)	15.6 (9.9–22.0)	14.3 (8.6–20.2)	<0.1 (<0.1–0.2)	<0.1 (<0.1–0.2)	22.5 (14.3–31.1)	22.5 (14.3–31.1)
Greece	46.1 (21.3–64.7)	45.4 (19.8–64.8)	1.8 (0.0–7.4)	2.4 (0.0–9.7)	0.1 (<0.1–0.2)	0.1 (<0.1–0.2)	7.1 (4.8–9.9)	4.7 (3.2–6.5)	1.0 (0.4–1.7)	1.0 (0.4–1.7)	5.4 (3.6–7.5)	3.8 (2.6–5.3)	7.2 (4.3–11.1)	7.1 (4.2–10.9)	1.6 (0.6–3.7)	1.9 (0.8–4.5)	11.5 (8.4–15.2)	11.4 (8.3–15.0)	20.7 (13.5–27.7)	20.8 (13.6–28.1)	0.1 (<0.1–0.3)	0.1 (<0.1–0.3)	15.7 (12.3–19.4)	15.7 (12.3–19.4)
Iceland	36.9 (16.2–52.3)	39.3 (17.0–57.1)	1.5 (0.0–5.8)	2.1 (0.0–8.1)	<0.1 (<0.1–<0.1)	<0.1 (<0.1–<0.1)	16.4 (12.5–21.2)	11.1 (8.2–14.4)	1.0 (0.4–1.7)	1.0 (0.4–1.7)	13.4 (10.0–17.3)	9.4 (6.9–12.5)	2.3 (0.4–5.2)	1.9 (0.3–4.1)	1.3 (0.5–3.1)	1.7 (0.7–4.0)	7.4 (5.0–9.9)	7.8 (5.4–10.4)	17.1 (11.2–23.4)	17.9 (12.1–24.3)	<0.1 (0.0–0.0)	<0.1 (0.0–0.0)	21.5 (5.1–38.7)	21.5 (5.1–38.7)
Ireland	37.7 (16.9–53.5)	33.9 (14.7–49.3)	1.5 (0.0–5.7)	1.8 (0.0–7.0)	<0.1 (<0.1–<0.1)	<0.1 (<0.1–0.1)	15.3 (11.2–20.2)	17.1 (12.8–22.2)	1.0 (0.4–1.6)	1.0 (0.4–1.6)	12.4 (9.1–16.8)	14.7 (10.9–19.2)	3.6 (1.5–7.0)	3.7 (1.5–7.1)	1.3 (0.4–3.0)	1.4 (0.5–3.3)	8.2 (5.6–11.2)	8.2 (5.5–11.1)	12.1 (7.2–17.4)	12.4 (7.3–17.7)	<0.1 (0.0–0.0)	<0.1 (0.0–0.0)	25.7 (14.4–37.2)	25.7 (14.4–37.2)
Israel	40.0 (17.9–57.0)	39.4 (16.9–57.2)	1.6 (0.0–6.0)	2.1 (0.0–8.1)	<0.1 (<0.1–0.1)	<0.1 (<0.1–0.1)	13.5 (10.2–17.4)	11.8 (8.6–15.3)	1.0 (0.4–1.7)	1.0 (0.4–1.7)	11.5 (8.5–14.8)	10.2 (7.5–13.5)	10.7 (6.9–15.6)	10.6 (6.8–15.8)	1.4 (0.5–3.3)	1.7 (0.7–4.0)	8.1 (5.5–10.9)	8.9 (6.1–11.9)	13.2 (7.8–18.7)	13.5 (8.0–18.8)	2.1 (0.8–3.9)	2.1 (0.8–3.9)	3.5 (<0.1–10.4)	3.5 (<0.1–10.4)
Italy	40.4 (18.4–56.6)	37.0 (15.7–53.4)	1.6 (0.0–6.7)	1.9 (0.0–7.5)	<0.1 (<0.1–0.1)	<0.1 (<0.1–0.1)	14.6 (12.3–16.7)	15.0 (12.8–17.2)	1.0 (0.4–1.7)	1.0 (0.4–1.7)	10.9 (9.1–12.5)	11.9 (10.1–13.6)	8.2 (5.4–12.2)	8.4 (5.5–12.5)	1.4 (0.5–3.2)	1.6 (0.6–3.7)	11.6 (8.7–14.6)	10.6 (7.9–13.3)	14.5 (9.1–20.0)	15.0 (9.6–21.0)	0.1 (<0.1–0.2)	0.1 (<0.1–0.2)	17.1 (13.0–21.2)	17.2 (13.0–21.3)
Luxembourg	39.6 (17.9–56.2)	39.5 (16.3–57.1)	1.6 (0.0–5.9)	2.1 (0.0–8.2)	<0.1 (<0.1–<0.1)	<0.1 (<0.1–<0.1)	15.1 (11.0–19.9)	10.3 (7.6–13.8)	1.0 (0.4–1.6)	1.0 (0.4–1.6)	12.2 (8.9–16.2)	8.5 (6.2–11.4)	5.0 (2.6–8.3)	4.7 (2.5–7.9)	1.4 (0.5–3.4)	1.8 (0.7–4.2)	8.7 (6.2–11.5)	7.0 (4.8–9.4)	15.7 (10.0–22.0)	15.2 (9.8–21.2)	<0.1 (<0.1–0.1)	<0.1 (<0.1–0.1)	23.1 (13.3–33.6)	23.1 (13.3–33.6)

	Child wasting		Child stunting		Household air pollution		Low birth weight		Handwashing		Short gestation		Ambient particulate matter		Child underweight		Non-exclusive breastfeeding		Secondhand smoke		High temperature		Low temperature	
	**M**	**F**	**M**	**F**	**M**	**F**	**M**	**F**	**M**	**F**	**M**	**F**	**M**	**F**	**M**	**F**	**M**	**F**	**M**	**F**	**M**	**F**	**M**	**F**
Malta	42.1 (19.1–58.3)	39.4 (17.1–56.1)	1.7 (0.0–6.7)	2.1 (0.0–8.2)	<0.1 (<0.1–0.1)	<0.1 (<0.1–0.1)	14.3 (10.6–18.7)	13.3 (9.9–17.5)	1.0 (0.4–1.7)	1.0 (0.4–1.7)	11.4 (8.4–15.2)	11.3 (8.2–15.0)	6.7 (3.9–10.4)	6.7 (3.8–10.4)	1.5 (0.5–3.5)	1.7 (0.7–4.0)	9.3 (6.5–12.5)	8.6 (5.9–11.5)	16.7 (11.0–23.0)	17.2 (11.3–23.7)	0.3 (<0.1–0.9)	0.3 (<0.1–0.9)	3.6 (<0.1–15.0)	3.6 (<0.1–15.0)
Monaco	48.0 (22.4–66.1)	39.1 (15.0–59.3)	1.9 (0.0–7.6)	2.1 (0.0–8.2)	<0.1 (<0.1–<0.1)	<0.1 (<0.1–<0.1)	5.3 (2.6–9.6)	5.5 (3.0–9.1)	1.0 (0.4–1.6)	1.0 (0.4–1.6)	4.3 (2.1–7.9)	4.6 (2.6–7.7)	5.5 (2.8–9.4)	5.6 (2.8–9.4)	1.6 (0.6–3.8)	1.7 (0.7–4.0)	10.3 (7.1–13.8)	3.9 (2.0–6.4)	15.8 (9.6–22.0)	16.1 (10.4–22.3)	<0.1 (<0.1–0.1)	<0.1 (<0.1–0.1)	15.4 (11.9–19.0)	15.4 (11.9–19.0)
Netherlands	40.0 (17.8–56.4)	38.9 (16.5–56.8)	1.6 (0.0–6.2)	2.0 (0.0–7.6)	<0.1 (<0.1–<0.1)	<0.1 (<0.1–<0.1)	13.9 (10.4–17.9)	12.2 (9.1–16.0)	1.0 (0.4–1.6)	1.0 (0.4–1.6)	10.8 (8.0–13.9)	10.0 (7.4–13.1)	6.2 (3.5–10.0)	6.1 (3.4–9.6)	1.4 (0.5–3.3)	1.7 (0.7–3.9)	12.7 (9.3–16.3)	12.7 (9.3–16.4)	13.1 (8.0–18.7)	13.5 (8.2–19.3)	<0.1 (<0.1–0.1)	<0.1 (<0.1–0.1)	23.6 (13.4–33.5)	23.6 (13.4–33.5)
Norway	39.9 (17.6–56.2)	37.2 (15.8–53.7)	1.6 (0.0–6.2)	1.9 (0.0–7.7)	<0.1 (<0.1–<0.1)	<0.1 (<0.1–<0.1)	12.2 (10.9–13.6)	12.8 (11.5–14.2)	1.0 (0.4–1.6)	1.0 (0.4–1.6)	10.3 (9.2–11.5)	10.8 (9.8–11.9)	2.6 (1.4–4.4)	2.7 (1.4–4.6)	1.4 (0.5–3.3)	1.6 (0.6–3.7)	6.3 (4.1–8.6)	6.4 (4.3–8.8)	12.8 (7.6–18.3)	13.4 (8.4–19.1)	<0.1 (<0.1–0.1)	<0.1 (<0.1–0.1)	19.0 (5.8–35.9)	18.9 (5.6–35.9)
Portugal	37.0 (16.9–51.9)	32.7 (13.7–47.8)	1.5 (0.0–6.2)	1.8 (0.0–7.2)	0.1 (<0.1–0.2)	0.1 (<0.1–0.2)	19.9 (15.1–25.5)	22.1 (17.2–27.7)	1.1 (0.4–1.8)	1.1 (0.4–1.8)	13.9 (10.5–17.7)	16.3 (12.6–20.5)	3.8 (1.7–6.7)	3.9 (1.6–6.9)	1.3 (0.5–3.1)	1.5 (0.6–3.5)	11.7 (8.6–15.4)	11.7 (8.7–15.4)	12.8 (7.6–18.0)	13.0 (7.7–18.3)	0.2 (<0.1–0.3)	0.2 (<0.1–0.3)	15.3 (11.7–19.2)	15.3 (11.7–19.2)
San Marino	42.0 (18.2–61.0)	38.4 (15.9–56.5)	1.7 (0.0–6.7)	2.1 (0.0–8.3)	<0.1 (<0.1–<0.1)	<0.1 (<0.1–<0.1)	10.1 (5.9–15.9)	9.2 (5.2–14.4)	1.0 (0.4–1.7)	1.0 (0.4–1.7)	8.1 (4.6–13.0)	7.7 (4.4–12.1)	4.6 (2.0–8.2)	4.6 (1.9–8.4)	1.5 (0.6–3.6)	1.8 (0.7–4.1)	6.3 (3.6–9.2)	5.1 (2.9–8.1)	17.1 (11.2–23.8)	17.4 (11.3–23.8)	0.1 (<0.1–0.2)	0.1 (<0.1–0.2)	15.7 (12.5–19.0)	15.7 (12.5–19.0)
Spain	36.2 (15.2–52.7)	32.8 (12.1–50.2)	1.4 (0.0–5.5)	1.8 (0.0–7.1)	0.1 (<0.1–0.3)	0.1 (<0.1–0.3)	17.0 (11.3–23.4)	15.4 (10.0–21.4)	1.0 (0.4–1.7)	1.0 (0.4–1.7)	11.7 (7.8–16.0)	10.5 (6.8–14.7)	4.7 (2.5–8.1)	4.6 (2.5–7.6)	1.1 (0.3–2.5)	1.6 (0.6–3.7)	11.7 (8.5–15.4)	11.6 (8.4–15.2)	16.3 (10.2–22.4)	16.2 (10.0–22.3)	0.2 (<0.1–0.5)	0.2 (<0.1–0.5)	17.3 (13.3–21.5)	17.3 (13.3–21.5)
Sweden	41.1 (18.4–57.4)	39.1 (16.1–57.0)	1.6 (0.0–6.2)	2.0 (0.0–8.2)	<0.1 (<0.1–<0.1)	<0.1 (<0.1–<0.1)	10.8 (8.2–13.9)	9.6 (7.5–12.2)	1.0 (0.4–1.7)	1.0 (0.4–1.7)	9.8 (7.4–12.6)	9.2 (7.1–11.7)	2.2 (0.6–4.6)	2.1 (0.6–4.3)	1.4 (0.5–3.4)	1.7 (0.7–4.0)	8.3 (5.7–11.1)	8.0 (5.5–10.5)	10.2 (6.3–15.0)	10.4 (6.5–14.8)	<0.1 (<0.1–0.1)	<0.1 (<0.1–0.1)	18.5 (5.2–32.8)	18.5 (5.2–32.8)
Switzerland	40.2 (18.2–56.3)	34.0 (14.8–49.6)	1.6 (0.0–6.1)	1.8 (0.0–6.8)	<0.1 (<0.1–<0.1)	<0.1 (<0.1–<0.1)	14.5 (10.9–19.3)	19.1 (14.3–24.8)	1.0 (0.4–1.6)	1.0 (0.4–1.6)	11.6 (8.5–15.5)	16.6 (12.4–21.9)	4.8 (2.4–8.3)	5.2 (2.7–9.3)	1.4 (0.5–3.2)	1.5 (0.6–3.5)	9.3 (6.6–12.4)	8.6 (6.0–11.5)	13.9 (8.3–19.6)	14.1 (8.4–19.9)	<0.1 (<0.1–<0.1)	<0.1 (<0.1–<0.1)	21.1 (11.3–32.9)	21.1 (11.3–32.9)
United Kingdom	40.9 (18.9–57.1)	37.7 (16.5–54.5)	1.7 (0.0–6.6)	2.0 (0.0–7.7)	<0.1 (<0.1–<0.1)	<0.1 (<0.1–<0.1)	13.8 (10.6–17.6)	14.4 (11.1–18.4)	1.0 (0.4–1.7)	1.0 (0.4–1.7)	11.2 (8.5–14.3)	12.3 (9.5–15.7)	5.1 (3.3–8.0)	5.2 (3.4–8.2)	1.4 (0.5–3.4)	1.7 (0.6–4.0)	9.3 (6.5–12.4)	8.6 (6.0–11.5)	13.5 (8.4–19.3)	13.7 (8.2–19.2)	<0.1 (<0.1–<0.1)	<0.1 (<0.1–<0.1)	24.8 (15.7–34.9)	24.8 (15.9–34.9)
**Latin America and Caribbean**	**47.1 (25.8–61.3)**	**47.2 (25.8–61.5)**	**8.0 (0.3–23.6)**	**8.6 (0.2–25.0)**	**11.7 (6.5–18.1)**	**11.6 (6.4–18.5)**	**13.6 (11.9–15.6)**	**12.1 (10.4–14.1)**	**9.1 (3.9–14.2)**	**9.1 (3.8–14.2)**	**9.5 (8.3–11.0)**	**8.8 (7.5–10.3)**	**8.7 (5.9–12.6)**	**8.8 (5.9–12.6)**	**4.4 (2.5–9.2)**	**4.6 (2.6–9.5)**	**9.4 (6.2–13.1)**	**8.9 (5.8–12.3)**	**5.4 (2.8–8.5)**	**5.4 (2.7–8.3)**	**1.3 (<0.1–4.4)**	**1.3 (<0.1–4.6)**	**2.8 (1.3–4.4)**	**3.0 (1.5–4.5)**
Andean Latin America	37.6 (17.3–53.3)	37.7 (17.8–53.6)	8.1 (0.2–25.3)	8.4 (<0.1–26.2)	5.4 (2.3–10.3)	5.5 (2.4–10.5)	11.0 (8.4–14.2)	10.2 (7.9–13.3)	7.9 (3.3–12.5)	7.9 (3.3–12.5)	7.8 (5.9–10.1)	7.8 (6.0–10.0)	12.8 (8.0–19.3)	12.9 (8.1–19.4)	3.3 (1.6–7.4)	3.3 (1.5–7.5)	6.1 (3.4–9.2)	5.8 (3.2–8.9)	2.9 (1.0–5.2)	2.9 (1.1–5.3)	0.8 (0.2–1.8)	0.8 (0.2–1.9)	8.9 (5.6–12.3)	8.9 (5.6–12.4)
Bolivia (Plurinational State of)	39.6 (18.7–56.3)	43.2 (22.7–59.7)	7.6 (0.1–25.3)	8.9 (0.0–27.6)	7.8 (3.3–14.5)	7.8 (3.4–14.5)	9.9 (6.1–14.7)	8.3 (5.0–13.4)	8.4 (3.5–13.2)	8.4 (3.5–13.2)	6.8 (4.2–10.3)	6.7 (4.1–10.5)	12.4 (7.4–19.3)	12.4 (7.5–19.3)	3.0 (1.3–6.9)	3.5 (1.7–8.1)	6.4 (3.3–10.0)	6.1 (3.2–9.8)	2.7 (0.9–5.0)	2.7 (1.0–5.2)	1.5 (0.5–2.7)	1.5 (0.5–2.7)	9.1 (5.9–12.6)	9.1 (5.9–12.6)

	**Child wasting**		**Child stunting**		**Household air pollution**		**Low birth weight**		**Handwashing**		**Short gestation**		**Ambient particulate matter**		**Child underweight**		**Non-exclusive breastfeeding**		**Secondhand smoke**		**High temperature**		**Low temperature**	
	**M**	**F**	**M**	**F**	**M**	**F**	**M**	**F**	**M**	**F**	**M**	**F**	**M**	**F**	**M**	**F**	**M**	**F**	**M**	**F**	**M**	**F**	**M**	**F**
Ecuador	51.1 (27.4–67.2)	44.1 (22.2–59.9)	9.3 (0.3–27.2)	8.0 (0.2–25.0)	1.3 (0.4–3.0)	1.3 (0.4–3.0)	8.7 (6.1–12.1)	11.7 (8.8–15.3)	6.9 (2.8–11.0)	6.9 (2.8–11.0)	6.0 (4.1–8.7)	7.6 (5.7–10.1)	10.4 (5.8–16.4)	10.3 (5.8–16.3)	4.1 (2.2–8.9)	3.4 (1.6–8.0)	7.9 (4.9–11.2)	7.4 (4.5–10.7)	2.7 (0.9–5.2)	2.8 (1.0–5.3)	0.3 (<0.1–1.2)	0.3 (<0.1–1.2)	5.8 (3.6–8.1)	5.8 (3.6–8.1)
Peru	26.3 (9.7–42.2)	26.2 (9.2–42.1)	8.0 (0.1–25.4)	7.9 (<0.1–24.8)	4.5 (1.8–8.9)	4.5 (1.8–8.9)	14.3 (9.5–20.0)	12.4 (8.5–17.7)	7.9 (3.3–12.5)	7.9 (3.3–12.5)	10.5 (7.0–15.1)	9.8 (6.5–14.1)	15.0 (9.5–22.3)	15.0 (9.5–22.3)	3.4 (1.7–7.7)	2.9 (1.2–6.9)	4.6 (2.2–7.5)	4.4 (2.0–7.4)	3.1 (1.1–5.8)	3.1 (1.2–5.8)	0.2 (<0.1–0.9)	0.2 (<0.1–0.9)	10.4 (6.0–15.2)	10.4 (6.0–15.2)
Caribbean	49.0 (29.1–64.1)	45.5 (27.6–60.4)	8.0 (0.3–23.3)	8.1 (0.4–22.6)	34.1 (20.9–47.1)	34.1 (20.9–46.9)	22.1 (14.7–30.8)	24.6 (16.6–33.8)	18.8 (8.4–28.2)	18.8 (8.4–28.0)	15.3 (10.0–21.5)	17.3 (11.7–24.1)	5.8 (2.7–10.9)	5.8 (2.7–10.9)	6.5 (3.8–13.0)	5.9 (3.5–11.9)	8.8 (5.4–12.6)	9.1 (5.6–12.9)	3.6 (1.7–5.8)	3.6 (1.8–5.8)	0.8 (<0.1–2.9)	0.8 (<0.1–2.9)	<0.1 (<0.1–1.3)	<0.1 (<0.1–1.3)
Antigua and Barbuda	44.0 (23.8–58.5)	48.3 (26.1–63.7)	4.1 (0.0–15.0)	5.1 (0.0–17.9)	0.2 (<0.1–0.4)	0.2 (<0.1–0.4)	19.7 (14.5–25.7)	14.0 (9.8–19.2)	7.5 (3.1–11.9)	7.5 (3.1–11.9)	13.7 (10.1–18.0)	10.3 (7.1–14.2)	9.2 (3.5–18.4)	9.1 (3.5–18.4)	2.7 (1.3–6.0)	3.5 (1.6–7.5)	9.2 (5.4–13.9)	8.7 (4.9–13.3)	6.3 (3.6–9.5)	6.6 (3.8–9.7)	0.4 (<0.1–2.3)	0.4 (<0.1–2.3)	0.3 (<0.1–1.7)	0.3 (<0.1–1.7)
Bahamas	48.0 (27.1–62.3)	45.9 (26.5–60.1)	4.2 (0.0–15.2)	4.6 (<0.1–15.5)	0.2 (<0.1–0.5)	0.2 (<0.1–0.5)	17.5 (13.5–22.2)	18.9 (14.3–24.5)	6.3 (2.6–10.2)	6.3 (2.6–10.2)	12.6 (9.6–16.3)	14.0 (10.6–18.0)	8.0 (2.6–16.5)	8.1 (2.7–16.4)	2.8 (1.3–6.4)	3.2 (1.6–7.1)	10.5 (6.5–14.9)	11.2 (7.0–15.8)	7.6 (4.3–11.3)	7.8 (4.6–11.3)	1.6 (<0.1–4.5)	1.6 (<0.1–4.5)	0.3 (<0.1–2.0)	0.3 (<0.1–2.0)
Barbados	39.5 (23.2–51.9)	39.2 (21.5–52.4)	4.4 (0.1–13.9)	3.7 (0.1–11.9)	<0.1 (<0.1–<0.1)	<0.1 (<0.1–<0.1)	29.0 (23.1–35.2)	23.6 (18.6–29.0)	7.1 (2.9–11.4)	7.1 (2.9–11.4)	21.1 (16.6–25.9)	18.0 (14.2–22.2)	11.4 (4.9–21.4)	11.5 (4.9–21.6)	2.1 (0.9–4.7)	1.1 (0.5–2.6)	10.1 (6.2–14.3)	12.0 (7.4–16.9)	5.5 (3.0–8.2)	5.5 (3.1–8.2)	1.2 (<0.1–3.9)	1.2 (<0.1–3.9)	0.4 (<0.1–2.0)	0.4 (<0.1–2.0)
Belize	41.5 (22.0–55.2)	37.1 (19.5–52.1)	6.3 (0.3–19.4)	6.1 (0.2–18.2)	3.8 (1.6–7.8)	3.9 (1.6–7.7)	20.4 (16.2–24.8)	23.3 (17.7–29.0)	9.5 (4.0–15.0)	9.5 (4.0–15.0)	14.4 (11.3–17.5)	17.2 (12.7–21.8)	10.6 (3.8–20.6)	10.6 (4.0–20.5)	3.0 (1.6–6.2)	3.0 (1.6–6.2)	11.3 (6.9–16.2)	10.2 (6.2–14.9)	7.0 (3.8–10.5)	7.1 (3.9–10.7)	1.7 (<0.1–4.2)	1.7 (<0.1–4.2)	0.1 (<0.1–1.3)	0.1 (<0.1–1.3)
Cuba	34.2 (16.1–48.7)	38.6 (19.2–53.5)	2.4 (0.0–8.9)	5.2 (0.2–16.3)	0.4 (0.1–1.0)	0.4 (0.1–0.9)	18.6 (14.8–22.8)	15.7 (12.5–19.6)	6.0 (2.5–9.6)	6.0 (2.5–9.6)	14.7 (11.5–18.1)	12.7 (10.0–15.8)	9.6 (4.6–16.9)	9.5 (4.6–17.0)	1.3 (0.6–2.9)	1.8 (0.8–3.9)	10.2 (7.1–13.4)	10.0 (7.1–13.2)	10.5 (6.1–15.3)	10.1 (6.2–14.8)	1.5 (<0.1–4.0)	1.5 (<0.1–4.0)	0.2 (<0.1–1.6)	0.2 (<0.1–1.6)
Dominica	44.7 (24.8–60.6)	46.7 (26.1–62.2)	4.1 (<0.1–14.7)	4.9 (<0.1–17.2)	1.4 (0.4–3.3)	1.4 (0.4–3.3)	19.6 (12.8–27.2)	16.1 (10.7–23.3)	8.3 (3.5–13.2)	8.3 (3.5–13.2)	13.2 (8.6–18.9)	11.6 (7.7–16.7)	9.6 (4.0–18.9)	9.6 (4.0–18.9)	2.7 (1.2–5.8)	3.2 (1.6–7.1)	9.5 (5.6–14.3)	9.9 (5.9–14.7)	5.5 (2.8–8.5)	5.7 (3.0–8.9)	1.1 (<0.1–3.5)	1.1 (<0.1–3.5)	0.3 (<0.1–3.2)	0.3 (<0.1–3.2)
Dominican Republic	39.6 (19.3–56.3)	41.3 (21.3–58.0)	4.3 (<0.1–14.6)	5.2 (0.1–17.6)	2.5 (0.9–5.6)	2.5 (0.9–5.6)	21.7 (12.7–33.9)	18.8 (10.5–30.4)	16.0 (7.1–24.3)	16.0 (7.1–24.3)	15.6 (9.3–24.8)	13.5 (7.4–22.2)	8.9 (3.9–17.7)	9.0 (3.8–17.7)	2.2 (1.0–4.7)	3.0 (1.4–6.4)	10.6 (5.9–16.2)	12.8 (7.0–19.1)	5.6 (3.2–8.3)	5.7 (3.2–8.5)	0.6 (<0.1–2.2)	0.6 (<0.1–2.2)	<0.1 (<0.1–1.7)	<0.1 (<0.1–1.7)
Grenada	46.2 (26.2–60.1)	46.8 (26.1–61.3)	4.2 (<0.1–14.6)	5.2 (0.1–16.8)	0.6 (0.2–1.3)	0.6 (0.2–1.3)	19.6 (15.6–24.2)	16.8 (12.8–21.2)	7.6 (3.2–12.2)	7.6 (3.2–12.2)	13.7 (10.6–17.0)	12.1 (9.3–15.3)	11.2 (4.2–21.4)	11.1 (4.4–21.4)	2.9 (1.4–6.4)	3.5 (1.7–7.6)	9.7 (5.7–14.2)	9.7 (5.8–14.2)	5.4 (2.6–8.8)	5.5 (2.8–8.9)	1.4 (<0.1–4.2)	1.4 (<0.1–4.2)	0.3 (<0.1–1.8)	0.3 (<0.1–1.8)
Guyana	48.5 (31.8–60.9)	58.7 (40.2–71.0)	4.6 (0.1–14.9)	8.1 (0.4–24.3)	1.5 (0.6–3.1)	1.5 (0.6–3.2)	27.0 (19.9–34.9)	17.7 (12.5–24.1)	14.1 (6.1–21.9)	14.1 (6.1–21.9)	19.2 (14.0–25.1)	13.8 (9.6–19.1)	10.2 (4.3–20.4)	10.3 (3.9–20.4)	4.5 (2.7–9.5)	6.6 (4.0–13.2)	9.4 (5.6–14.0)	8.7 (5.1–13.2)	7.4 (3.9–11.4)	7.4 (3.9–11.3)	0.9 (<0.1–2.8)	0.9 (<0.1–2.8)	<0.1 (<0.1–1.3)	<0.1 (<0.1–1.3)
Haiti	50.2 (30.2–65.9)	45.9 (27.9–61.4)	8.6 (0.4–24.6)	8.5 (0.4–23.3)	38.5 (23.7–52.8)	38.5 (23.8–52.6)	22.1 (13.7–31.7)	25.3 (16.2–35.9)	19.5 (8.7–29.2)	19.5 (8.7–29.2)	15.2 (9.2–21.9)	17.8 (11.5–25.3)	5.3 (2.3–10.4)	5.3 (2.3–10.4)	7.0 (4.1–14.0)	6.3 (3.7–12.6)	8.6 (5.1–12.4)	8.7 (5.2–12.7)	3.2 (1.4–5.3)	3.2 (1.5–5.2)	0.8 (<0.1–3.0)	0.8 (<0.1–3.0)	<0.1 (<0.1–1.3)	<0.1 (<0.1–1.3)

	Child wasting		Child stunting		Household air pollution		Low birth weight		Handwashing		Short gestation		Ambient particulate matter		Child underweight		Non-exclusive breastfeeding		Secondhand smoke		High temperature		Low temperature	
	**M**	**F**	**M**	**F**	**M**	**F**	**M**	**F**	**M**	**F**	**M**	**F**	**M**	**F**	**M**	**F**	**M**	**F**	**M**	**F**	**M**	**F**	**M**	**F**
Jamaica	28.2 (14.8–40.3)	28.7 (14.9–41.5)	2.6 (<0.1–8.8)	2.4 (0.0–8.8)	2.0 (0.7–4.5)	1.9 (0.6–4.4)	39.8 (32.0–47.8)	37.1 (29.2–44.9)	11.2 (4.8–17.4)	11.2 (4.8–17.4)	27.1 (21.4–32.7)	26.0 (20.3–31.7)	8.2 (4.6–13.2)	8.0 (4.7–13.0)	1.6 (0.8–3.7)	1.4 (0.6–3.4)	9.0 (5.3–13.3)	9.6 (5.7–14.0)	9.0 (5.2–13.0)	9.0 (5.2–13.0)	1.2 (<0.1–4.3)	1.2 (<0.1–4.3)	0.1 (<0.1–1.1)	0.1 (<0.1–1.1)
Saint Kitts and Nevis	34.1 (17.4–48.1)	39.6 (21.2–53.8)	3.0 (0.0–10.4)	3.9 (0.0–14.0)	0.4 (0.1–1.2)	0.4 (0.1–1.1)	31.4 (24.2–44.1)	24.2 (18.2–33.9)	7.6 (3.2–12.1)	7.6 (3.2–12.1)	22.7 (17.1–32.0)	18.2 (13.6–25.7)	4.5 (1.8–8.3)	4.4 (1.8–8.0)	1.9 (0.8–4.4)	2.6 (1.1–5.9)	9.6 (5.9–14.2)	10.4 (6.4–15.4)	7.1 (3.7–11.0)	7.1 (3.9–10.7)	1.0 (<0.1–3.9)	1.0 (<0.1–3.9)	0.4 (<0.1–2.3)	0.4 (<0.1–2.3)
Saint Lucia	39.2 (22.1–52.2)	31.5 (15.9–44.3)	1.4 (0.0–5.5)	2.6 (0.0–9.5)	0.7 (0.2–1.6)	0.7 (0.2–1.6)	26.0 (20.5–31.8)	26.6 (22.0–31.8)	5.7 (2.3–9.2)	5.7 (2.3–9.2)	18.2 (14.1–22.7)	19.0 (15.6–23.0)	11.2 (4.8–21.0)	11.2 (4.6–21.0)	1.3 (0.5–3.0)	1.2 (0.5–2.8)	9.6 (5.5–14.1)	9.9 (5.7–14.3)	5.8 (3.0–8.8)	5.9 (3.2–8.9)	1.7 (<0.1–4.8)	1.7 (<0.1–4.8)	0.3 (<0.1–1.9)	0.3 (<0.1–1.9)
Saint Vincent and the Grenadines	42.0 (23.4–55.1)	45.1 (25.2–58.6)	3.9 (0.0–13.4)	4.8 (0.1–16.9)	0.9 (0.3–1.9)	0.9 (0.3–1.9)	22.5 (18.2–27.2)	19.1 (15.1–23.5)	9.1 (3.8–14.3)	9.1 (3.8–14.3)	15.6 (12.3–19.0)	13.5 (10.6–16.7)	11.0 (4.3–21.1)	11.0 (4.2–21.1)	2.6 (1.3–5.9)	3.1 (1.6–7.0)	9.5 (5.7–14.0)	11.9 (7.3–17.3)	6.4 (3.4–10.1)	6.4 (3.4–10.0)	1.3 (<0.1–4.0)	1.3 (<0.1–4.0)	0.4 (<0.1–2.0)	0.4 (<0.1–2.0)
Suriname	48.6 (29.3–62.8)	48.5 (28.5–63.2)	3.6 (0.0–12.8)	4.8 (0.1–15.8)	2.2 (0.8–4.7)	2.2 (0.8–4.7)	21.3 (14.2–30.0)	20.9 (13.9–29.8)	9.3 (3.9–14.9)	9.3 (3.9–14.9)	14.8 (9.5–21.2)	15.1 (9.8–21.4)	10.8 (4.5–20.5)	10.8 (4.7–20.4)	4.0 (2.1–8.4)	5.2 (2.9–10.5)	12.5 (7.2–19.0)	13.9 (8.3–20.6)	11.7 (6.6–17.0)	11.8 (6.7–17.1)	1.0 (<0.1–3.0)	1.0 (<0.1–3.0)	0.3 (<0.1–1.6)	0.3 (<0.1–1.6)
Trinidad and Tobago	41.5 (23.0–53.8)	41.5 (22.2–55.2)	1.5 (0.0–5.6)	2.9 (0.1–10.1)	<0.1 (<0.1–<0.1)	<0.1 (<0.1–<0.1)	28.4 (22.7–34.6)	26.4 (20.5–33.1)	6.8 (2.8–10.9)	6.8 (2.8–10.9)	17.2 (13.7–21.3)	16.3 (12.6–20.4)	11.4 (4.2–22.8)	11.3 (4.2–22.8)	2.2 (1.1–4.9)	2.6 (1.4–5.4)	12.1 (7.0–17.8)	12.1 (6.9–18.0)	8.8 (4.6–13.3)	8.9 (4.7–13.8)	0.4 (<0.1–2.6)	0.4 (<0.1–2.6)	0.2 (<0.1–1.2)	0.2 (<0.1–1.2)
Central Latin America	44.3 (23.4–58.7)	46.7 (24.8–61.9)	9.3 (0.5–26.1)	10.5 (0.6–29.2)	8.9 (4.5–15.1)	8.8 (4.5–15.0)	15.2 (13.4–17.3)	11.5 (10.0–13.1)	6.3 (2.6–10.1)	6.3 (2.6–10.1)	10.2 (8.9–11.6)	7.9 (6.9–9.1)	10.6 (7.2–15.3)	10.6 (7.2–15.3)	4.4 (2.4–9.3)	4.8 (2.7–10.2)	9.5 (5.9–13.7)	9.0 (5.6–12.9)	4.0 (1.6–7.1)	4.1 (1.6–7.2)	1.3 (<0.1–3.2)	1.3 (<0.1–3.1)	3.3 (1.5–5.2)	3.4 (1.5–5.3)
Colombia	39.2 (19.1–55.3)	43.8 (20.6–61.3)	4.5 (0.0–15.4)	7.2 (0.1–22.8)	2.3 (0.8–5.0)	2.3 (0.8–5.0)	16.1 (11.0–21.5)	9.9 (6.4–13.8)	6.1 (2.5–9.9)	6.1 (2.5–9.9)	10.7 (7.2–14.4)	7.1 (4.6–10.0)	11.3 (6.9–17.4)	11.2 (6.8–17.4)	2.4 (1.1–5.4)	3.9 (2.0–8.5)	9.2 (5.3–13.9)	8.9 (5.0–13.4)	5.0 (2.4–8.3)	5.2 (2.4–8.4)	1.0 (<0.1–3.4)	1.0 (<0.1–3.4)	3.4 (1.8–5.1)	3.4 (1.8–5.1)
Costa Rica	37.9 (17.8–53.8)	43.5 (22.5–59.3)	3.6 (0.0–12.4)	4.4 (0.0–15.2)	1.1 (0.4–2.6)	1.1 (0.4–2.6)	14.1 (10.9–18.2)	11.8 (8.9–15.5)	6.8 (2.8–10.9)	6.8 (2.8–10.9)	9.8 (7.5–12.7)	9.2 (6.9–12.1)	9.1 (5.5–13.7)	9.2 (5.8–13.7)	1.9 (0.8–4.6)	2.3 (1.0–5.3)	8.3 (5.1–11.9)	8.5 (5.2–12.1)	8.1 (4.5–12.1)	8.2 (4.5–12.1)	0.3 (<0.1–2.2)	0.3 (<0.1–2.2)	0.6 (<0.1–2.7)	0.6 (<0.1–2.7)
El Salvador	40.0 (18.5–57.2)	48.1 (24.8–65.4)	5.8 (0.0–19.2)	7.7 (0.2–24.1)	3.9 (1.6–7.5)	3.9 (1.6–7.5)	13.6 (8.7–20.5)	8.6 (4.9–13.8)	9.0 (3.8–14.4)	9.0 (3.8–14.4)	9.0 (5.7–13.9)	6.2 (3.4–10.0)	11.1 (6.1–17.8)	11.1 (6.1–17.8)	2.6 (1.2–5.7)	5.6 (3.3–11.2)	8.2 (4.6–12.2)	8.9 (5.1–13.4)	3.5 (1.7–5.6)	3.4 (1.7–5.4)	1.4 (<0.1–6.7)	1.4 (<0.1–6.7)	<0.1 (<0.1–0.6)	<0.1 (<0.1–0.6)
Guatemala	47.0 (23.3–63.0)	48.6 (24.9–65.7)	16.5 (1.6–41.2)	18.1 (1.6–44.5)	17.2 (8.6–29.6)	17.2 (8.5–29.6)	10.6 (9.0–12.4)	7.4 (6.1–8.8)	7.6 (3.2–12.1)	7.6 (3.2–12.1)	7.0 (5.8–8.2)	5.0 (4.1–5.9)	11.2 (6.9–16.9)	11.2 (7.0–16.9)	7.1 (4.2–14.4)	7.4 (4.3–15.1)	7.1 (3.8–10.8)	6.7 (3.6–10.2)	3.2 (1.2–5.8)	3.2 (1.2–5.6)	1.7 (0.5–3.5)	1.7 (0.5–3.5)	1.5 (<0.1–3.2)	1.5 (<0.1–3.2)
Honduras	30.7 (13.5–47.2)	28.6 (12.5–46.0)	6.8 (0.1–20.1)	6.9 (0.2–20.3)	21.4 (11.9–33.7)	21.5 (12.1–33.7)	28.2 (17.2–41.2)	29.9 (18.0–41.9)	5.4 (2.2–8.6)	5.4 (2.2–8.6)	18.6 (11.3–27.4)	20.9 (12.6–30.0)	8.5 (5.2–13.0)	8.5 (5.3–12.7)	3.8 (1.8–8.1)	3.9 (1.8–8.1)	9.5 (4.7–15.0)	8.9 (4.5–14.2)	6.8 (3.6–10.4)	7.0 (3.7–10.6)	0.7 (<0.1–2.6)	0.7 (<0.1–2.6)	<0.1 (<0.1–2.4)	<0.1 (<0.1–2.4)
Mexico	45.6 (25.3–60.1)	48.5 (26.9–63.8)	6.2 (0.1–19.5)	7.1 (0.1–22.0)	4.4 (2.0–7.9)	4.8 (2.1–8.6)	16.6 (11.8–21.9)	12.9 (8.8–17.3)	5.2 (2.1–8.3)	5.2 (2.1–8.4)	11.2 (7.9–14.8)	8.8 (6.0–11.8)	10.2 (6.9–15.0)	10.1 (6.6–15.0)	3.4 (1.8–7.5)	3.7 (1.8–8.2)	11.2 (7.4–15.4)	10.5 (6.9–14.5)	3.3 (0.9–6.7)	3.4 (0.9–6.8)	1.1 (<0.1–2.2)	1.1 (<0.1–2.1)	6.9 (4.3–9.7)	6.7 (4.2–9.5)

	Child wasting		Child stunting		Household air pollution		Low birth weight		Handwashing		Short gestation		Ambient particulate matter		Child underweight		Non-exclusive breastfeeding		Secondhand smoke		High temperature		Low temperature	
	**M**	**F**	**M**	**F**	**M**	**F**	**M**	**F**	**M**	**F**	**M**	**F**	**M**	**F**	**M**	**F**	**M**	**F**	**M**	**F**	**M**	**F**	**M**	**F**
Nicaragua	41.2 (18.9–58.1)	39.2 (18.8–55.1)	6.5 (0.1–21.2)	7.0 (0.1–22.2)	16.7 (8.1–28.3)	16.8 (8.3–28.3)	14.4 (9.4–20.4)	12.9 (9.3–16.9)	8.1 (3.4–12.8)	8.1 (3.4–12.8)	9.8 (6.4–14.2)	9.3 (6.7–12.3)	8.6 (4.9–13.7)	8.6 (4.9–13.5)	3.0 (1.4–6.7)	2.9 (1.3–6.6)	9.5 (5.7–13.7)	8.3 (4.8–12.4)	5.5 (2.5–9.2)	5.5 (2.5–9.0)	0.8 (<0.1–4.9)	0.8 (<0.1–4.9)	<0.1 (<0.1–1.1)	<0.1 (<0.1–1.1)
Panama	43.9 (20.9–60.7)	45.7 (21.8–63.5)	7.7 (0.1–25.2)	7.7 (0.2–24.6)	1.8 (0.5–3.8)	1.7 (0.5–3.8)	9.5 (7.5–11.8)	6.6 (5.2–8.3)	6.1 (2.5–9.8)	6.1 (2.5–9.8)	6.4 (5.0–8.0)	4.8 (3.7–6.2)	6.7 (3.5–10.9)	6.6 (3.4–10.9)	3.3 (1.6–7.4)	3.2 (1.5–7.2)	10.8 (7.5–14.6)	10.4 (7.0–14.0)	4.2 (2.4–6.3)	4.3 (2.3–6.6)	0.6 (<0.1–2.3)	0.6 (<0.1–2.3)	<0.1 (<0.1–1.0)	<0.1 (<0.1–1.0)
Venezuela (Bolivarian Republic of)	43.8 (25.1–56.5)	46.2 (26.7–59.5)	6.1 (0.2–18.4)	6.7 (0.3–19.5)	0.1 (<0.1–0.2)	0.1 (<0.1–0.2)	22.6 (18.5–27.3)	18.5 (14.7–22.8)	5.7 (2.4–9.2)	5.7 (2.4–9.2)	15.3 (12.3–18.6)	13.1 (10.4–16.5)	11.6 (6.8–18.6)	11.5 (6.5–18.6)	2.7 (1.3–5.6)	3.0 (1.5–6.3)	11.6 (7.5–16.2)	11.2 (7.1–15.7)	6.3 (2.8–10.3)	6.5 (3.0–10.7)	1.2 (<0.1–9.4)	1.2 (<0.1–9.4)	0.1 (<0.1–1.7)	0.1 (<0.1–1.7)
Tropical Latin America	55.6 (31.4–70.7)	55.5 (31.3–71.3)	5.8 (0.1–19.6)	6.1 (<0.1–20.4)	3.2 (1.2–6.6)	3.4 (1.2–7.0)	6.7 (6.1–7.5)	5.3 (4.8–5.8)	6.7 (2.8–10.7)	7.0 (2.8–11.1)	5.5 (4.9–6.1)	4.6 (4.2–5.1)	5.5 (3.4–8.7)	5.4 (3.3–8.6)	3.7 (1.9–7.8)	4.1 (2.2–8.6)	11.9 (8.3–15.9)	10.8 (7.4–14.5)	10.4 (6.2–15.4)	10.7 (6.1–15.4)	2.0 (<0.1–10.3)	2.1 (<0.1–11.2)	0.7 (<0.1–2.1)	0.6 (<0.1–1.9)
Brazil	55.8 (31.7–70.9)	56.0 (31.9–71.6)	5.9 (0.1–19.6)	6.1 (<0.1–20.5)	3.0 (1.1–6.3)	3.2 (1.1–6.7)	6.8 (6.1–7.5)	5.4 (4.8–5.9)	6.7 (2.8–10.7)	6.9 (2.8–11.1)	5.5 (5.0–6.2)	4.7 (4.2–5.2)	5.5 (3.3–8.6)	5.4 (3.2–8.5)	3.7 (1.9–7.8)	4.2 (2.3–8.8)	11.9 (8.3–15.9)	10.8 (7.4–14.3)	10.5 (6.2–15.5)	10.8 (6.2–15.5)	1.9 (<0.1–10.4)	2.0 (<0.1–11.5)	0.7 (<0.1–2.1)	0.6 (<0.1–1.9)
Paraguay	46.3 (22.3–65.3)	41.5 (17.3–60.7)	5.5 (0.1–19.1)	5.3 (0.0–18.3)	8.0 (3.0–15.8)	7.9 (3.0–15.8)	4.7 (2.8–7.5)	2.8 (1.5–4.5)	7.9 (3.3–12.7)	7.9 (3.3–12.7)	3.8 (2.2–6.0)	2.4 (1.3–3.9)	6.2 (3.6–10.4)	6.2 (3.6–10.4)	2.4 (1.0–5.5)	1.9 (0.8–4.7)	11.6 (6.8–17.0)	12.2 (7.2–18.0)	8.4 (4.5–13.0)	8.6 (4.8–12.7)	5.2 (2.9–7.6)	5.2 (2.9–7.6)	0.9 (<0.1–5.0)	0.9 (<0.1–5.0)
**North Africa and Middle East**	**55.2 (36.8–66.3)**	**58.6 (39.2–70.1)**	**11.8 (1.0–30.0)**	**13.2 (1.0–33.0)**	**18.4 (10.6–26.5)**	**19.7 (11.6–28.4)**	**20.0 (15.7–24.6)**	**16.5 (12.4–20.8)**	**11.4 (4.9–18.1)**	**11.7 (4.9–18.5)**	**14.8 (11.5–18.4)**	**12.7 (9.6–16.2)**	**17.2 (11.6–24.2)**	**16.7 (11.3–23.6)**	**8.9 (5.4–16.4)**	**9.9 (6.3–19.2)**	**7.8 (4.0–12.1)**	**8.0 (4.2–12.7)**	**13.0 (8.3–18.2)**	**12.9 (8.1–17.6)**	**5.2 (3.3–7.3)**	**5.1 (3.2–7.2)**	**8.1 (4.0–12.2)**	**8.3 (4.2–12.6)**
Afghanistan	54.3 (34.6–67.7)	58.7 (37.9–72.7)	15.4 (1.7–36.9)	15.9 (1.4–38.2)	36.3 (23.1–48.0)	36.2 (23.0–48.0)	21.2 (13.3–29.9)	15.9 (9.4–23.7)	15.7 (6.8–23.8)	15.7 (6.8–23.8)	15.4 (9.6–22.0)	11.8 (7.0–17.5)	10.6 (4.7–18.8)	10.6 (4.7–19.0)	11.5 (7.3–20.8)	12.1 (7.7–22.4)	6.9 (3.4–11.7)	7.2 (3.4–11.8)	10.5 (6.2–15.1)	10.3 (6.1–14.9)	3.1 (1.9–4.2)	3.1 (1.9–4.2)	13.7 (9.0–19.6)	13.7 (9.0–19.6)
Algeria	57.2 (37.1–72.3)	58.3 (39.1–72.7)	5.7 (0.1–18.5)	6.6 (0.2–20.9)	0.1 (<0.1–0.1)	0.1 (<0.1–0.1)	12.7 (6.9–21.2)	11.7 (6.5–18.8)	4.9 (2.0–7.9)	4.9 (2.0–7.9)	10.7 (5.6–17.6)	11.1 (6.2–17.9)	17.0 (10.2–25.7)	17.0 (10.0–25.7)	3.6 (1.9–7.6)	4.5 (2.4–9.2)	10.4 (6.5–15.0)	9.9 (6.1–14.3)	16.0 (10.2–21.9)	16.2 (10.3–22.4)	9.2 (6.2–12.2)	9.2 (6.2–12.2)	4.3 (<0.1–10.1)	4.3 (<0.1–10.1)
Bahrain	63.5 (37.8–79.0)	61.7 (37.3–77.0)	4.5 (0.0–16.0)	4.3 (0.0–15.7)	0.1 (<0.1–0.2)	0.1 (<0.1–0.2)	3.9 (2.4–6.4)	6.3 (3.6–10.2)	4.6 (1.9–7.4)	4.6 (1.9–7.4)	3.2 (1.9–5.2)	5.4 (3.1–8.8)	26.5 (18.0–36.4)	26.6 (18.0–36.4)	4.0 (1.9–8.9)	3.9 (1.9–8.6)	6.9 (3.8–11.0)	7.3 (3.9–11.1)	12.5 (7.7–17.9)	13.3 (8.3–18.8)	11.6 (5.8–16.6)	11.6 (5.8–16.6)	2.0 (<0.1–10.1)	2.0 (<0.1–10.1)
Egypt	50.6 (31.8–65.3)	51.6 (31.4–66.8)	7.5 (0.4–23.3)	9.4 (0.4–26.8)	<0.1 (<0.1–0.1)	<0.1 (<0.1–0.1)	22.9 (13.7–35.3)	22.3 (13.0–33.6)	3.8 (1.5–6.3)	3.8 (1.5–6.3)	17.5 (10.5–27.3)	18.1 (10.5–27.5)	29.0 (19.6–40.0)	29.0 (19.8–40.0)	2.7 (1.4–6.0)	3.7 (1.9–8.0)	8.5 (3.9–13.7)	8.7 (4.1–14.2)	16.2 (10.4–22.3)	16.3 (10.3–22.1)	7.7 (4.8–10.9)	7.7 (4.8–10.9)	2.7 (<0.1–9.1)	2.7 (<0.1–9.1)
Iran (Islamic Republic of)	51.7 (32.6–62.9)	52.2 (32.9–64.0)	5.0 (0.1–16.8)	5.1 (0.1–16.4)	0.1 (<0.1–0.2)	0.1 (<0.1–0.2)	18.2 (15.5–21.5)	17.9 (14.7–21.4)	4.4 (1.8–7.2)	4.4 (1.8–7.2)	13.3 (11.1–15.7)	14.1 (11.5–16.8)	19.9 (13.5–27.7)	19.8 (13.6–27.5)	3.3 (1.7–7.0)	3.5 (1.8–7.7)	7.6 (4.4–11.2)	7.7 (4.4–11.2)	13.1 (8.1–18.5)	13.4 (8.3–18.5)	4.3 (2.9–5.6)	4.2 (2.9–5.6)	11.4 (8.0–14.6)	11.4 (7.8–14.6)
Iraq	48.0 (30.3–62.1)	52.3 (33.7–65.4)	7.6 (0.2–22.4)	8.1 (0.3–23.6)	0.1 (<0.1–0.1)	0.1 (<0.1–0.1)	25.7 (17.7–35.5)	20.2 (13.5–28.1)	2.7 (1.1–4.5)	2.7 (1.1–4.5)	19.6 (13.3–27.1)	16.6 (11.0–23.2)	22.9 (15.2–32.4)	22.9 (15.2–32.4)	4.6 (2.6–9.2)	4.7 (2.6–9.6)	7.9 (4.1–12.8)	8.2 (4.4–13.6)	15.9 (10.1–21.9)	15.7 (10.1–21.5)	10.8 (7.5–13.8)	10.8 (7.5–13.8)	4.7 (<0.1–10.6)	4.7 (<0.1–10.6)

	Child wasting		Child stunting		Household air pollution		Low birth weight		Handwashing		Short gestation		Ambient particulate matter		Child underweight		Non-exclusive breastfeeding		Secondhand smoke		High temperature		Low temperature	
	M	F	M	F	M	F	M	F	M	F	M	F	M	F	M	F	M	F	M	F	M	F	M	F
Jordan	46.0 (26.2–60.0)	51.2 (28.8–65.8)	3.9 (0.1–13.5)	5.1 (0.1–16.7)	<0.1 (<0.1–<0.1)	<0.1 (<0.1–<0.1)	21.6 (16.0–28.1)	14.2 (10.1–19.2)	4.2 (1.7–6.9)	4.2 (1.7–6.9)	16.3 (12.1–21.3)	11.6 (8.4–15.7)	15.9 (10.4–23.4)	15.9 (10.4–23.4)	1.9 (0.8–4.2)	3.2 (1.8–6.7)	11.3 (7.0–15.9)	10.8 (6.8–15.3)	16.0 (10.0–22.2)	16.5 (10.3–22.5)	3.2 (1.7–4.9)	3.2 (1.7–4.9)	6.0 (<0.1–12.1)	6.0 (<0.1–12.1)
Kuwait	48.6 (24.9–64.5)	49.6 (26.2–65.6)	3.0 (0.0–11.3)	2.8 (0.0–10.5)	<0.1 (<0.1–<0.1)	<0.1 (<0.1–<0.1)	13.4 (10.0–17.5)	12.7 (9.4–16.7)	3.7 (1.5–6.0)	3.7 (1.5–6.0)	11.3 (8.3–14.9)	11.3 (8.3–15.0)	27.2 (19.1–36.9)	27.2 (19.2–36.9)	2.2 (0.9–5.1)	2.0 (0.8–4.7)	10.9 (7.6–14.6)	11.0 (7.7–14.7)	18.4 (12.0–25.1)	18.2 (12.1–24.4)	13.1 (7.6–18.0)	13.1 (7.6–18.0)	3.1 (<0.1–15.6)	3.1 (<0.1–15.6)
Lebanon	53.4 (33.6–67.8)	56.2 (35.6–71.3)	5.9 (0.2–19.5)	4.7 (0.1–15.7)	<0.1 (<0.1–0.1)	0.1 (<0.1–0.1)	16.8 (9.4–26.8)	13.7 (7.4–22.3)	5.0 (2.1–8.0)	5.0 (2.1–8.0)	13.1 (7.3–20.9)	12.0 (6.5–19.6)	15.2 (9.5–22.6)	15.3 (9.8–22.7)	2.5 (1.2–5.9)	1.6 (0.7–3.7)	10.8 (6.4–15.6)	12.3 (7.5–17.7)	19.4 (12.7–26.6)	19.5 (12.9–26.2)	0.1 (<0.1–0.2)	0.1 (<0.1–0.2)	14.0 (10.4–17.5)	14.0 (10.4–17.5)
Libya	57.5 (33.2–72.8)	55.6 (32.1–71.0)	11.6 (0.6–31.1)	7.6 (0.1–24.5)	<0.1 (<0.1–0.1)	<0.1 (<0.1–0.1)	11.0 (6.2–17.7)	11.2 (6.4–17.5)	4.5 (1.9–7.4)	4.5 (1.9–7.4)	8.5 (4.8–13.6)	9.3 (5.2–14.3)	19.1 (11.5–29.3)	19.2 (11.6–29.3)	3.3 (1.4–7.3)	3.5 (1.7–7.8)	4.8 (2.3–8.3)	4.5 (2.2–7.7)	16.8 (10.8–23.2)	16.8 (10.9–23.1)	8.2 (4.7–12.0)	8.2 (4.7–12.0)	2.8 (<0.1–9.8)	2.8 (<0.1–9.8)
Morocco	44.8 (26.6–61.6)	41.3 (23.7–58.6)	4.5 (0.1–15.1)	5.2 (0.2–16.2)	1.2 (0.5–2.5)	1.3 (0.5–2.5)	27.9 (16.2–42.7)	28.3 (16.4–41.8)	7.6 (3.2–12.2)	7.6 (3.2–12.2)	20.9 (11.9–31.8)	22.9 (13.2–34.3)	17.5 (11.4–25.0)	17.7 (12.0–25.0)	1.6 (0.6–3.6)	2.0 (0.9–4.4)	10.0 (6.0–14.8)	10.3 (6.0–15.7)	10.6 (6.1–15.1)	10.8 (6.4–15.2)	2.5 (1.6–3.4)	2.5 (1.6–3.4)	8.2 (4.8–11.8)	8.2 (4.8–11.8)
Oman	59.7 (39.9–73.0)	61.1 (40.5–74.3)	7.1 (0.2–22.1)	7.3 (0.2–23.0)	0.1 (<0.1–0.3)	0.1 (<0.1–0.3)	15.0 (8.6–23.7)	13.2 (7.7–21.1)	5.0 (2.0–8.2)	5.0 (2.0–8.2)	12.0 (6.9–19.0)	11.2 (6.5–17.6)	21.5 (13.4–31.3)	21.6 (13.5–31.3)	6.2 (3.7–12.3)	6.2 (3.8–12.4)	6.1 (3.0–9.9)	6.0 (3.1–9.7)	11.3 (7.0–16.2)	12.1 (7.7–17.0)	6.6 (<0.1–22.3)	6.6 (<0.1–22.3)	1.3 (<0.1–13.3)	1.3 (<0.1–13.3)
Palestine	50.6 (27.9–66.6)	49.5 (26.3–66.3)	5.0 (0.1–17.0)	5.7 (0.1–18.8)	0.3 (0.1–0.6)	0.3 (0.1–0.6)	11.5 (7.2–17.2)	9.8 (6.2–14.6)	7.1 (3.0–11.4)	7.1 (3.0–11.4)	8.6 (5.4–12.8)	8.4 (5.2–12.5)	16.1 (10.1–24.7)	16.2 (10.3–24.7)	2.2 (1.0–5.0)	2.3 (1.0–5.2)	8.5 (4.6–13.4)	7.5 (3.9–11.9)	16.2 (10.3–22.3)	16.2 (10.4–22.3)	2.2 (0.9–4.0)	2.2 (0.9–4.0)	3.9 (<0.1–10.5)	3.9 (<0.1–10.5)
Qatar	59.1 (35.9–73.4)	56.8 (34.3–71.8)	5.0 (0.0–17.7)	5.0 (0.0–17.6)	<0.1 (<0.1–<0.1)	<0.1 (<0.1–<0.1)	10.3 (6.0–16.9)	12.1 (7.0–18.8)	3.8 (1.5–6.3)	3.8 (1.5–6.3)	8.5 (4.9–13.8)	10.6 (6.2–16.4)	31.2 (22.2–41.1)	31.2 (22.5–41.1)	2.9 (1.3–7.1)	3.0 (1.3–6.6)	11.2 (7.3–15.5)	9.6 (6.1–13.9)	14.4 (9.0–20.5)	15.1 (9.9–20.6)	10.6 (3.7–19.5)	10.6 (3.7–19.5)	1.8 (<0.1–15.2)	1.8 (<0.1–15.2)
Saudi Arabia	61.7 (36.4–78.0)	61.7 (36.2–77.1)	7.1 (0.0–23.3)	7.0 (0.0–23.6)	0.1 (<0.1–0.2)	0.1 (<0.1–0.2)	4.5 (2.4–8.0)	4.8 (2.6–8.1)	4.2 (1.7–6.7)	4.2 (1.7–6.7)	4.1 (2.2–7.2)	4.6 (2.5–7.7)	27.1 (18.7–37.3)	27.1 (18.6–37.3)	4.8 (2.4–10.6)	5.1 (2.5–11.1)	5.0 (2.4–8.6)	5.4 (2.8–8.7)	15.8 (10.0–22.2)	16.3 (10.5–22.3)	9.7 (5.9–14.1)	9.7 (5.9–14.1)	2.4 (<0.1–8.8)	2.4 (<0.1–8.8)
Sudan	67.5 (51.2–79.9)	70.2 (53.7–81.6)	11.9 (0.9–32.3)	13.3 (1.3–34.7)	14.7 (7.5–24.8)	14.6 (7.4–24.8)	12.5 (6.5–21.8)	10.6 (5.8–17.3)	17.7 (7.9–26.7)	17.7 (7.9–26.7)	7.4 (3.7–13.0)	7.0 (3.7–11.6)	19.6 (11.5–30.0)	19.6 (11.5–30.2)	13.0 (8.4–23.5)	13.4 (8.5–23.7)	5.3 (2.5–9.2)	6.0 (2.7–10.0)	12.5 (8.3–17.1)	12.3 (8.3–16.8)	8.3 (3.6–15.7)	8.3 (3.6–15.7)	1.5 (<0.1–7.2)	1.5 (<0.1–7.2)
Syrian Arab Republic	68.4 (45.9–81.3)	68.4 (45.3–81.4)	16.4 (1.6–41.4)	16.3 (1.5–42.1)	<0.1 (<0.1–0.1)	<0.1 (<0.1–0.1)	5.5 (3.1–9.0)	4.7 (2.7–7.8)	4.9 (2.0–7.9)	4.9 (2.0–7.9)	4.3 (2.4–6.9)	4.0 (2.3–6.7)	15.9 (9.9–23.6)	16.0 (9.7–23.6)	6.9 (4.3–13.7)	5.9 (3.5–12.6)	3.3 (1.6–5.8)	3.0 (1.4–5.4)	14.3 (8.7–20.0)	14.4 (9.0–20.4)	4.4 (2.5–6.3)	4.4 (2.5–6.3)	7.7 (2.5–12.6)	7.7 (2.5–12.6)
Tunisia	50.0 (27.6–65.3)	39.5 (21.9–56.1)	5.0 (0.1–15.4)	5.1 (0.1–17.0)	0.1 (<0.1–0.1)	0.1 (<0.1–0.1)	14.9 (8.3–24.1)	25.3 (14.8–37.8)	5.7 (2.4–9.2)	5.7 (2.4–9.2)	11.5 (6.7–18.8)	21.5 (12.8–32.2)	15.9 (9.7–24.1)	16.3 (10.1–24.1)	1.8 (0.8–4.2)	1.9 (0.8–4.1)	11.9 (6.5–18.0)	12.6 (6.9–19.7)	16.2 (10.5–22.2)	16.4 (10.4–22.4)	4.5 (2.3–6.7)	4.5 (2.3–6.7)	5.1 (<0.1–11.3)	5.1 (<0.1–11.3)
Turkey	42.8 (21.8–59.2)	51.9 (30.5–66.9)	5.0 (<0.1–16.7)	5.1 (0.1–16.7)	0.1 (<0.1–0.2)	0.1 (<0.1–0.2)	14.4 (9.3–20.7)	13.7 (9.1–20.1)	5.8 (2.4–9.3)	5.8 (2.4–9.3)	11.6 (7.4–16.6)	11.7 (7.7–17.2)	13.7 (8.7–20.4)	13.8 (8.9–20.5)	1.9 (0.8–4.1)	1.9 (0.8–4.4)	7.4 (3.0–12.5)	6.5 (2.6–11.2)	18.0 (11.5–24.7)	18.6 (11.9–25.2)	0.4 (0.1–0.7)	0.4 (0.1–0.7)	18.3 (13.7–23.4)	18.3 (13.7–23.4)

	Child wasting		Child stunting		Household air pollution		Low birth weight		Handwashing		Short gestation		Ambient particulate matter		Child underweight		Non-exclusive breastfeeding		Secondhand smoke		High temperature		Low temperature	
	**M**	**F**	**M**	**F**	**M**	**F**	**M**	**F**	**M**	**F**	**M**	**F**	**M**	**F**	**M**	**F**	**M**	**F**	**M**	**F**	**M**	**F**	**M**	**F**
United Arab Emirates	65.5 (43.9–78.4)	64.7 (43.7–78.0)	7.6 (0.1–24.0)	7.6 (0.1–23.8)	<0.1 (<0.1–<0.1)	<0.1 (<0.1–<0.1)	9.6 (5.2–15.8)	10.0 (5.7–16.8)	3.8 (1.5–6.2)	3.8 (1.5–6.2)	7.9 (4.2–13.0)	8.6 (4.9–14.5)	21.3 (13.7–30.8)	21.3 (13.8–30.8)	4.2 (2.0–9.6)	4.5 (2.2–9.5)	7.1 (4.2–10.3)	7.0 (4.3–10.3)	14.2 (9.0–19.9)	15.3 (9.6–20.6)	8.4 (<0.1–25.0)	8.4 (<0.1–25.0)	1.9 (<0.1–18.2)	1.9 (<0.1–18.2)
Yemen	63.0 (43.7–78.2)	69.2 (50.6–81.0)	14.8 (1.2–35.0)	16.7 (1.5–40.5)	15.0 (7.5–25.2)	14.9 (7.3–25.0)	19.9 (8.9–34.4)	13.6 (6.9–23.5)	13.5 (5.8–20.7)	13.5 (5.8–20.7)	15.9 (7.2–27.0)	10.6 (5.3–18.4)	15.9 (8.5–25.7)	16.0 (8.4–25.8)	16.6 (10.4–29.3)	18.1 (12.0–31.4)	11.1 (6.0–17.1)	11.2 (5.8–17.7)	15.4 (9.8–21.1)	15.5 (10.0–21.4)	5.5 (1.6–12.3)	5.5 (1.6–12.3)	0.9 (<0.1–5.1)	0.9 (<0.1–5.1)
**South Asia**	**46.5 (35.8–53.8)**	**51.3 (39.8–58.7)**	**10.0 (0.9–25.1)**	**11.5 (1.0–28.1)**	**22.2 (13.5–31.8)**	**22.0 (13.4–31.6)**	**36.5 (32.3–40.6)**	**31.3 (27.5–35.4)**	**12.8 (5.5–19.5)**	**12.8 (5.6–19.8)**	**22.6 (19.7–25.6)**	**20.5 (17.8–23.9)**	**22.4 (16.1–29.2)**	**22.9 (16.6–29.9)**	**10.5 (7.0–18.7)**	**11.8 (7.9–20.9)**	**6.3 (3.5–9.6)**	**6.9 (3.8–10.4)**	**9.5 (5.1–14.2)**	**9.7 (5.4–14.4)**	**8.1 (4.9–12.2)**	**8.3 (5.0–12.3)**	**3.1 (<0.1–8.8)**	**3.1 (<0.1–8.8)**
Bangladesh	41.3 (26.4–55.1)	49.8 (34.8–63.2)	8.2 (0.3–22.9)	9.4 (0.5–25.7)	26.5 (16.1–38.7)	26.4 (15.8–38.6)	40.2 (27.9–52.1)	30.8 (21.1–41.8)	18.1 (8.1–27.1)	18.1 (8.1–27.1)	26.1 (17.7–34.5)	21.2 (14.3–29.1)	17.6 (11.7–23.5)	17.7 (11.9–23.7)	7.8 (4.5–14.7)	9.6 (5.9–18.6)	6.1 (2.8–10.3)	6.0 (2.7–10.2)	10.2 (5.1–16.0)	10.2 (5.2–15.8)	5.9 (2.7–9.8)	5.9 (2.7–9.8)	1.3 (<0.1–4.8)	1.3 (<0.1–4.8)
Bhutan	45.2 (27.6–58.6)	52.6 (32.2–66.7)	7.9 (0.2–22.4)	9.2 (0.3–26.5)	20.1 (10.9–31.5)	20.0 (10.8–31.3)	27.7 (19.0–37.7)	21.7 (13.6–32.8)	6.1 (2.5–9.8)	6.1 (2.5–9.8)	17.3 (11.8–23.9)	14.4 (8.9–21.8)	13.6 (9.0–19.6)	13.7 (9.0–20.0)	4.5 (2.3–9.2)	5.4 (2.9–11.5)	6.7 (3.6–10.6)	7.0 (3.8–10.7)	4.9 (2.7–7.6)	5.0 (2.7–7.5)	<0.1 (<0.1–0.1)	<0.1 (<0.1–0.1)	18.6 (11.8–26.9)	18.6 (11.8–26.9)
India	44.7 (35.2–51.6)	50.3 (39.7–57.7)	9.5 (0.9–23.6)	11.2 (1.1–27.4)	21.8 (13.3–31.2)	21.5 (13.1–30.8)	40.2 (35.9–44.9)	33.8 (29.7–38.5)	12.4 (5.4–19.0)	12.5 (5.5–19.1)	24.4 (21.5–27.7)	21.7 (18.9–25.2)	24.2 (17.6–31.3)	25.0 (18.3–32.2)	10.2 (6.9–18.4)	12.1 (8.2–21.2)	6.1 (3.1–9.5)	6.7 (3.4–10.6)	9.8 (5.3–14.6)	10.2 (5.6–15.0)	8.1 (4.6–12.4)	8.3 (4.7–12.6)	2.3 (<0.1–7.6)	2.3 (<0.1–7.8)
Nepal	36.4 (22.5–50.1)	44.8 (28.7–59.9)	4.7 (0.1–13.5)	6.1 (0.1–18.3)	27.4 (16.8–39.0)	27.2 (16.3–39.0)	42.9 (31.5–53.7)	33.1 (22.9–43.3)	11.3 (4.9–17.8)	11.3 (4.9–17.8)	29.0 (21.1–36.8)	22.0 (14.9–29.7)	19.8 (12.2–28.1)	19.9 (12.4–28.4)	4.3 (2.2–9.1)	5.5 (3.1–11.2)	4.9 (2.4–7.9)	5.3 (2.7–8.4)	7.2 (3.1–12.2)	7.3 (3.2–12.2)	1.4 (0.7–2.2)	1.4 (0.7–2.2)	13.0 (7.6–19.0)	13.0 (7.6–19.0)
Pakistan	53.5 (39.1–64.2)	54.7 (39.6–65.9)	12.4 (1.2–30.6)	13.0 (1.0–31.7)	21.7 (12.5–32.5)	21.7 (12.6–32.7)	24.8 (18.5–31.6)	24.6 (17.7–31.4)	12.4 (5.3–19.2)	12.4 (5.2–19.2)	16.5 (12.2–21.3)	17.2 (12.3–22.1)	18.8 (12.5–26.1)	18.7 (12.5–25.7)	12.3 (7.8–21.5)	12.2 (7.7–22.0)	7.2 (4.2–10.6)	7.8 (4.6–11.3)	8.8 (4.7–13.1)	8.7 (4.8–12.8)	9.1 (5.3–17.1)	9.3 (5.6–15.9)	5.0 (<0.1–12.8)	4.9 (<0.1–12.4)
**Southeast Asia, East Asia, and Oceania**	**54.3 (36.8–65.2)**	**56.0 (37.7–66.6)**	**9.3 (0.4–26.0)**	**10.4 (0.5–29.0)**	**17.3 (9.7–26.4)**	**17.8 (10.1–26.9)**	**15.6 (13.5–17.7)**	**13.9 (12.3–15.6)**	**7.4 (3.1–11.8)**	**7.6 (3.2–12.0)**	**10.5 (9.0–12.1)**	**9.9 (8.7–11.2)**	**11.5 (8.0–15.7)**	**11.2 (7.8–15.2)**	**6.7 (4.2–13.3)**	**7.5 (4.7–15.0)**	**8.2 (5.4–11.5)**	**8.1 (5.2–11.2)**	**14.8 (9.0–20.8)**	**14.7 (8.9–20.6)**	**1.4 (<0.1–7.0)**	**1.4 (<0.1–6.5)**	**4.4 (2.4–6.4)**	**4.1 (2.2–6.1)**
East Asia	43.0 (23.3–55.5)	44.3 (24.9–56.4)	6.0 (0.1–19.5)	6.5 (0.1–20.3)	8.5 (4.0–15.2)	8.7 (4.2–15.3)	15.1 (13.8–16.5)	15.7 (14.4–16.9)	4.2 (1.7–6.8)	4.2 (1.7–6.8)	11.2 (10.2–12.3)	12.1 (10.9–13.2)	19.4 (13.8–25.5)	19.0 (13.6–25.1)	3.3 (1.6–7.1)	3.6 (1.9–7.6)	8.8 (6.1–11.6)	8.8 (6.0–11.5)	19.6 (12.8–26.3)	19.6 (12.9–26.2)	0.6 (<0.1–1.1)	0.6 (<0.1–1.1)	13.9 (8.6–18.7)	13.7 (8.5–18.3)
China	42.9 (23.1–55.5)	44.2 (24.7–56.2)	5.9 (0.1–19.4)	6.4 (0.1–20.2)	8.0 (3.6–14.6)	8.1 (3.7–14.7)	14.8 (13.6–16.2)	15.4 (14.1–16.6)	4.3 (1.7–7.0)	4.3 (1.7–6.9)	11.0 (10.1–12.0)	11.9 (10.9–13.0)	19.6 (13.9–25.7)	19.2 (13.7–25.3)	3.2 (1.5–6.9)	3.4 (1.8–7.3)	8.9 (6.1–11.8)	8.9 (6.1–11.7)	19.6 (12.9–26.4)	19.7 (13.0–26.3)	0.6 (<0.1–1.1)	0.6 (<0.1–1.1)	13.8 (8.6–18.4)	13.4 (8.4–17.9)
Democratic People's Republic of Korea	45.9 (29.6–59.4)	47.0 (31.3–60.5)	8.8 (0.4–25.1)	9.0 (0.4–25.3)	24.2 (13.7–36.5)	24.2 (13.6–36.5)	24.2 (16.6–34.2)	23.1 (15.7–31.5)	2.3 (0.9–3.7)	2.3 (0.9–3.7)	16.5 (11.2–23.3)	16.4 (11.1–22.7)	14.6 (9.2–20.8)	14.6 (9.3–20.9)	7.1 (4.1–14.3)	7.6 (4.5–14.2)	5.8 (3.5–8.6)	5.9 (3.6–8.7)	18.0 (11.3–24.4)	18.0 (11.5–24.5)	0.1 (<0.1–0.7)	0.1 (<0.1–0.7)	19.3 (11.7–29.7)	19.3 (11.7–29.7)
Taiwan (Province of China)	46.1 (22.3–63.5)	44.6 (21.3–62.2)	3.4 (0.0–12.2)	3.4 (0.0–12.0)	0.6 (0.1–1.6)	0.6 (0.1–1.6)	8.0 (6.0–10.4)	8.7 (6.5–11.4)	1.8 (0.7–3.0)	1.8 (0.7–3.0)	6.3 (4.7–8.2)	6.9 (5.2–9.2)	12.3 (8.0–17.9)	12.3 (7.9–17.9)	1.8 (0.7–4.4)	1.8 (0.7–4.2)	5.8 (3.6–8.3)	5.2 (3.2–7.4)	14.0 (8.4–19.9)	15.0 (9.3–20.6)	0.9 (0.2–2.3)	0.9 (0.2–2.3)	3.5 (<0.1–6.8)	3.5 (<0.1–6.8)

	Child wasting		Child stunting		Household air pollution		Low birth weight		Handwashing		Short gestation		Ambient particulate matter		Child underweight		Non-exclusive breastfeeding		Secondhand smoke		High temperature		Low temperature	
	**M**	**F**	**M**	**F**	**M**	**F**	**M**	**F**	**M**	**F**	**M**	**F**	**M**	**F**	**M**	**F**	**M**	**F**	**M**	**F**	**M**	**F**	**M**	**F**
Oceania	61.2 (45.7–71.9)	62.8 (45.5–73.9)	15.7 (1.3–37.3)	16.3 (1.0–39.4)	41.8 (26.0–55.1)	41.7 (25.9–55.2)	15.8 (11.2–21.6)	12.8 (8.8–17.3)	11.9 (5.0–18.6)	11.9 (5.0–18.6)	10.4 (7.2–14.4)	8.6 (6.0–11.9)	3.9 (1.0–10.5)	4.0 (1.0–10.6)	7.3 (3.6–14.9)	6.5 (2.9–14.3)	5.7 (2.9–9.1)	6.0 (3.2–9.4)	9.9 (4.7–15.9)	9.5 (4.5–15.5)	0.4 (<0.1–2.0)	0.4 (<0.1–1.9)	1.6 (<0.1–3.5)	1.6 (<0.1–3.5)
Fiji	62.1 (42.1–75.0)	62.4 (41.6–75.6)	2.8 (0.0–10.8)	4.7 (<0.1–16.3)	5.8 (1.9–12.3)	5.8 (1.9–12.3)	9.0 (5.6–13.3)	9.1 (5.6–13.6)	2.9 (1.2–4.8)	2.9 (1.2–4.8)	6.9 (4.3–10.5)	7.0 (4.3–10.7)	5.5 (1.6–13.1)	5.5 (1.6–13.1)	3.0 (1.3–6.6)	2.8 (1.2–6.2)	5.4 (3.0–8.2)	6.0 (3.4–8.9)	7.3 (3.3–12.2)	7.3 (3.3–12.3)	0.5 (<0.1–2.4)	0.5 (<0.1–2.4)	<0.1 (<0.1–1.7)	<0.1 (<0.1–1.7)
Kiribati	63.1 (43.3–76.0)	65.1 (45.9–77.3)	10.3 (0.3–29.4)	10.6 (0.4–30.2)	28.5 (16.3–42.4)	28.5 (16.3–42.4)	11.6 (7.2–18.0)	9.7 (5.8–15.0)	6.5 (2.7–10.4)	6.5 (2.7–10.4)	8.0 (4.9–12.5)	7.1 (4.1–11.0)	3.3 (1.1–7.8)	3.3 (1.1–7.7)	4.6 (2.4–9.9)	4.4 (2.3–9.3)	5.8 (3.0–9.1)	6.3 (3.3–9.7)	12.6 (6.8–18.9)	12.1 (6.5–18.4)	0.9 (<0.1–5.0)	0.9 (<0.1–5.0)	<0.1 (<0.1–0.1)	<0.1 (<0.1–0.1)
Marshall Islands	55.8 (35.7–69.6)	56.6 (36.3–70.4)	7.0 (0.1–21.4)	7.6 (0.2–23.7)	10.0 (4.2–18.5)	9.9 (4.2–18.6)	13.6 (8.8–19.9)	12.4 (7.6–18.5)	4.5 (1.8–7.3)	4.5 (1.8–7.3)	9.8 (6.4–14.6)	9.0 (5.5–13.7)	4.2 (1.6–8.8)	4.2 (1.6–8.8)	3.8 (1.8–8.5)	3.6 (1.6–7.7)	6.9 (4.1–10.3)	6.8 (4.1–9.8)	10.0 (5.1–15.4)	10.0 (5.3–14.9)	0.2 (<0.1–11.3)	0.2 (<0.1–11.3)	<0.1 (<0.1–0.1)	<0.1 (<0.1–0.1)
Micronesia (Federated States of)	57.6 (36.2–71.8)	56.5 (35.8–71.0)	7.3 (0.2–22.6)	7.2 (0.2–22.6)	8.8 (3.4–16.7)	8.9 (3.5–16.9)	12.9 (7.8–19.6)	13.2 (8.2–20.7)	3.9 (1.6–6.4)	3.9 (1.6–6.4)	9.4 (5.6–14.3)	9.8 (6.1–15.3)	5.0 (1.5–11.6)	5.0 (1.6–11.7)	3.8 (1.7–8.3)	3.4 (1.5–7.7)	6.4 (3.5–10.0)	7.1 (4.0–10.9)	8.1 (3.6–13.6)	8.0 (3.6–13.3)	0.2 (<0.1–11.3)	0.2 (<0.1–11.3)	<0.1 (<0.1–0.2)	<0.1 (<0.1–0.2)
Nauru	59.3 (37.1–73.0)	59.6 (36.3–73.9)	6.3 (0.0–21.3)	6.6 (0.0–22.4)	0.9 (0.2–2.2)	0.9 (0.2–2.2)	9.2 (5.7–14.0)	7.9 (5.1–11.6)	3.1 (1.2–5.1)	3.1 (1.2–5.1)	6.9 (4.3–10.5)	6.1 (3.9–9.0)	2.3 (0.7–5.4)	2.3 (0.7–5.3)	3.2 (1.4–7.4)	3.1 (1.3–7.4)	5.3 (2.9–8.0)	5.3 (3.0–7.9)	8.9 (3.8–14.7)	8.6 (3.8–14.1)	2.4 (<0.1–31.1)	2.4 (<0.1–31.1)	<0.1 (<0.1–0.3)	<0.1 (<0.1–0.3)
Palau	57.2 (34.7–71.0)	51.2 (30.5–63.6)	5.8 (0.0–19.8)	5.1 (0.0–17.3)	<0.1 (<0.1–<0.1)	<0.1 (<0.1–<0.1)	10.9 (7.3–15.0)	15.8 (12.7–19.1)	2.4 (0.9–3.9)	2.4 (0.9–3.9)	8.9 (5.9–12.3)	13.0 (10.4–16.0)	2.7 (0.3–6.3)	3.0 (0.2–7.1)	3.0 (1.2–7.0)	2.5 (1.0–5.6)	4.3 (2.6–6.4)	5.9 (3.6–8.4)	12.5 (6.7–18.6)	12.1 (6.8–17.8)	0.9 (<0.1–28.7)	0.9 (<0.1–28.7)	<0.1 (<0.1–0.4)	<0.1 (<0.1–0.4)
Papua New Guinea	61.5 (46.0–72.4)	63.1 (45.8–74.4)	16.0 (1.3–38.0)	16.6 (1.0–40.1)	42.3 (26.4–55.8)	42.3 (26.3–55.8)	15.9 (11.1–21.9)	12.8 (8.6–17.5)	12.2 (5.2–19.0)	12.2 (5.2–19.0)	10.4 (7.1–14.6)	8.6 (5.8–12.0)	3.9 (1.0–10.6)	4.0 (1.0–10.7)	7.4 (3.6–15.2)	6.6 (2.9–14.5)	5.7 (2.9–9.1)	6.0 (3.1–9.4)	9.9 (4.7–16.0)	9.6 (4.5–15.5)	0.4 (<0.1–1.9)	0.4 (<0.1–1.9)	1.6 (<0.1–3.7)	1.6 (<0.1–3.7)
Samoa	45.0 (23.2–60.8)	44.8 (23.2–60.8)	4.3 (0.1–14.4)	4.2 (0.1–13.8)	18.7 (9.1–31.3)	18.6 (9.1–31.4)	16.3 (10.9–23.5)	16.9 (11.4–24.6)	2.2 (0.9–3.6)	2.2 (0.9–3.6)	12.0 (8.0–17.4)	12.9 (8.6–18.6)	4.8 (1.3–11.3)	4.8 (1.3–11.4)	3.1 (1.5–6.7)	2.6 (1.2–5.9)	6.2 (3.7–9.0)	7.2 (4.4–10.3)	14.9 (8.8–20.9)	14.5 (8.8–20.4)	0.4 (<0.1–2.2)	0.4 (<0.1–2.2)	0.1 (<0.1–1.4)	0.1 (<0.1–1.4)
Solomon Islands	49.5 (30.7–62.6)	51.6 (30.9–65.4)	9.3 (0.5–26.4)	9.6 (0.4–27.3)	42.8 (27.4–56.9)	42.8 (27.3–56.9)	17.8 (12.7–23.1)	15.2 (10.6–20.2)	4.9 (2.0–7.9)	4.9 (2.0–7.9)	12.2 (8.7–16.2)	10.7 (7.5–14.1)	3.3 (0.9–7.6)	3.3 (0.9–7.7)	3.9 (1.9–8.4)	3.8 (1.9–8.2)	3.9 (2.1–5.8)	4.4 (2.5–6.6)	10.4 (5.3–16.1)	10.2 (5.3–15.4)	0.5 (<0.1–3.3)	0.5 (<0.1–3.3)	<0.1 (<0.1–0.5)	<0.1 (<0.1–0.5)
Tonga	54.4 (33.1–69.1)	52.1 (33.3–67.0)	5.6 (0.1–18.0)	5.0 (0.1–16.6)	9.0 (3.5–18.0)	9.2 (3.7–17.9)	13.4 (8.6–19.8)	16.1 (9.8–24.4)	3.3 (1.3–5.4)	3.3 (1.3–5.4)	10.5 (6.7–15.4)	13.0 (8.1–19.5)	5.2 (1.6–11.7)	5.2 (1.6–11.8)	2.3 (1.0–5.0)	2.2 (1.0–4.8)	5.6 (3.2–8.4)	7.5 (4.4–10.9)	12.0 (6.5–17.8)	11.7 (6.5–17.5)	1.2 (<0.1–3.0)	1.2 (<0.1–3.0)	0.2 (<0.1–2.6)	0.2 (<0.1–2.6)
Tuvalu	55.3 (35.3–69.5)	56.4 (36.3–70.3)	6.6 (0.1–21.0)	7.5 (0.3–23.5)	2.6 (0.8–5.7)	2.5 (0.7–5.6)	15.2 (9.7–22.1)	13.2 (8.6–19.3)	3.7 (1.5–6.1)	3.7 (1.5–6.1)	11.3 (7.1–16.5)	9.9 (6.4–14.5)	2.8 (1.2–5.8)	2.7 (1.1–5.6)	3.7 (1.7–8.2)	3.3 (1.5–7.6)	7.2 (4.1–10.5)	6.5 (3.7–9.5)	10.5 (5.4–16.2)	10.2 (5.1–15.8)	1.4 (<0.1–12.8)	1.4 (<0.1–12.8)	0.1 (<0.1–0.7)	0.1 (<0.1–0.7)
Vanuatu	52.6 (32.5–66.5)	53.5 (31.5–68.5)	9.8 (0.6–27.3)	10.5 (0.7–28.7)	33.5 (20.0–48.5)	33.5 (20.0–48.5)	14.6 (9.7–21.5)	13.7 (9.0–20.1)	10.0 (4.3–15.7)	10.0 (4.3–15.7)	10.2 (6.8–15.1)	9.6 (6.2–14.0)	4.3 (1.3–9.8)	4.3 (1.3–9.8)	5.8 (3.4–11.8)	6.3 (3.9–12.5)	7.3 (4.1–11.3)	8.2 (4.5–12.4)	6.6 (2.8–11.6)	6.6 (2.9–11.3)	0.4 (<0.1–2.1)	0.4 (<0.1–2.1)	<0.1 (<0.1–1.8)	<0.1 (<0.1–1.8)

	Child wasting		Child stunting		Household air pollution		Low birth weight		Handwashing		Short gestation		Ambient particulate matter		Child underweight		Non-exclusive breastfeeding		Secondhand smoke		High temperature		Low temperature	
	**M**	**F**	**M**	**F**	**M**	**F**	**M**	**F**	**M**	**F**	**M**	**F**	**M**	**F**	**M**	**F**	**M**	**F**	**M**	**F**	**M**	**F**	**M**	**F**
Southeast Asia	58.6 (41.4–69.0)	59.9 (42.0–70.4)	10.0 (0.5–28.1)	11.2 (0.7–30.6)	17.8 (9.5–28.2)	17.8 (9.4–27.9)	15.8 (12.8–19.0)	13.4 (11.2–15.8)	8.2 (3.4–13.1)	8.4 (3.5–13.2)	10.3 (8.2–12.5)	9.2 (7.6–10.9)	8.9 (6.0–12.2)	8.9 (6.0–12.3)	8.3 (5.2–16.0)	9.4 (6.1–18.0)	8.3 (5.1–11.9)	8.1 (4.9–11.5)	13.3 (7.6–19.4)	13.4 (7.7–19.5)	2.0 (<0.1–10.7)	1.9 (<0.1–10.2)	0.3 (<0.1–1.6)	0.3 (<0.1–1.7)
Cambodia	53.7 (36.7–65.2)	54.2 (37.4–65.2)	9.0 (0.3–25.4)	10.7 (0.6–29.5)	31.7 (18.8–46.1)	31.7 (18.8–46.1)	21.6 (16.6–27.2)	19.3 (14.5–24.5)	8.6 (3.5–13.5)	8.6 (3.5–13.5)	14.2 (10.6–18.3)	13.7 (10.2–18.0)	7.0 (3.9–11.3)	7.1 (3.9–11.3)	7.0 (4.0–13.9)	8.2 (4.8–15.9)	6.1 (3.5–9.1)	5.3 (3.0–7.9)	15.5 (9.8–21.5)	15.8 (9.9–21.7)	2.7 (<0.1–21.8)	2.7 (<0.1–21.8)	<0.1 (<0.1–0.3)	<0.1 (<0.1–0.3)
Indonesia	61.8 (47.1–72.7)	65.3 (49.3–75.7)	12.9 (1.2–33.0)	13.4 (1.1–35.0)	8.6 (3.7–16.2)	8.7 (3.7–16.4)	14.3 (10.5–18.6)	11.1 (7.8–14.7)	7.8 (3.2–12.3)	7.8 (3.3–12.3)	9.6 (7.0–12.5)	8.0 (5.7–10.8)	9.3 (6.0–13.5)	9.2 (6.0–13.3)	9.0 (5.7–17.0)	9.7 (6.3–18.3)	8.3 (5.5–11.3)	8.1 (5.4–11.0)	15.8 (9.3–22.5)	15.9 (9.1–22.8)	0.3 (<0.1–3.8)	0.4 (<0.1–4.0)	<0.1 (<0.1–0.9)	<0.1 (<0.1–1.0)
Lao People's Democratic Republic	61.8 (42.3–74.4)	66.0 (47.2–78.0)	10.7 (0.6–31.4)	12.2 (0.6–34.6)	34.0 (20.5–49.1)	34.0 (20.4–49.1)	11.1 (6.5–16.9)	8.7 (5.3–14.2)	10.2 (4.3–15.9)	10.2 (4.3–15.9)	7.2 (4.2–11.4)	6.5 (3.7–10.5)	6.6 (3.8–10.1)	6.6 (3.8–10.2)	8.8 (5.2–17.4)	11.2 (7.1–21.1)	9.7 (5.1–14.9)	9.3 (4.8–14.6)	13.0 (7.0–19.1)	13.1 (7.3–19.4)	1.8 (0.6–3.9)	1.8 (0.6–3.9)	<0.1 (<0.1–2.9)	<0.1 (<0.1–2.9)
Malaysia	57.5 (39.4–69.9)	56.4 (39.2–69.1)	7.2 (0.3–21.7)	7.3 (0.4–22.5)	0.1 (<0.1–0.3)	0.1 (<0.1–0.3)	18.6 (11.6–27.7)	17.5 (11.0–26.3)	2.0 (0.8–3.2)	2.0 (0.8–3.2)	14.0 (8.7–20.7)	14.0 (8.7–21.0)	8.8 (5.4–13.6)	9.0 (5.4–13.7)	6.8 (4.1–13.5)	6.5 (3.8–13.2)	8.4 (4.9–12.6)	8.5 (5.0–12.7)	13.0 (7.4–19.2)	13.1 (7.5–18.8)	0.8 (<0.1–3.9)	0.8 (<0.1–3.9)	<0.1 (<0.1–0.8)	<0.1 (<0.1–0.8)
Maldives	70.3 (52.5–81.8)	64.8 (47.2–76.5)	8.9 (0.2–26.7)	9.9 (0.3–28.5)	2.9 (1.0–6.9)	3.0 (1.0–6.7)	10.2 (5.4–17.3)	14.1 (8.6–21.6)	8.1 (3.5–13.0)	8.1 (3.5–13.0)	7.3 (3.8–12.6)	10.3 (6.3–15.6)	5.3 (3.0–8.5)	5.3 (3.2–8.4)	10.9 (6.9–20.7)	10.2 (6.4–19.5)	6.6 (3.1–10.9)	6.8 (3.3–11.0)	15.6 (9.3–22.3)	15.6 (9.1–21.9)	0.4 (<0.1–11.4)	0.4 (<0.1–11.4)	<0.1 (<0.1–0.1)	<0.1 (<0.1–0.1)
Mauritius	65.4 (48.3–74.9)	67.0 (50.4–77.7)	6.5 (0.1–20.6)	6.5 (0.1–21.1)	0.2 (0.1–0.5)	0.2 (0.1–0.5)	12.6 (9.9–15.9)	10.8 (7.1–14.9)	1.1 (0.4–1.8)	1.1 (0.4–1.8)	8.7 (6.8–11.0)	7.9 (5.4–10.9)	7.6 (3.5–13.8)	7.6 (3.5–13.8)	6.6 (4.0–13.6)	7.0 (4.2–14.3)	7.8 (4.8–11.3)	7.8 (4.7–11.3)	12.0 (6.7–17.8)	12.0 (6.6–17.7)	0.7 (<0.1–3.5)	0.7 (<0.1–3.5)	<0.1 (<0.1–5.4)	<0.1 (<0.1–5.4)
Myanmar	51.2 (34.8–64.8)	54.0 (35.6–66.6)	5.1 (0.1–17.1)	7.9 (0.3–24.1)	24.7 (13.8–38.0)	24.6 (13.7–38.2)	21.7 (14.3–31.2)	15.3 (10.0–21.4)	11.1 (4.7–17.5)	11.1 (4.7–17.5)	14.6 (9.5–21.4)	10.9 (7.1–15.8)	10.5 (7.0–14.6)	10.6 (7.1–14.8)	5.5 (3.0–11.4)	7.5 (4.7–14.8)	8.5 (4.6–12.9)	9.4 (4.9–14.1)	8.3 (3.8–13.9)	8.7 (4.0–14.3)	2.9 (1.3–5.3)	2.9 (1.3–5.3)	1.9 (<0.1–4.3)	1.9 (<0.1–4.3)
Philippines	63.0 (42.6–75.7)	58.9 (37.8–72.4)	11.4 (0.3–32.5)	11.6 (0.7–32.0)	17.0 (8.5–27.5)	17.0 (8.6–27.3)	12.3 (8.5–16.4)	14.2 (10.1–18.4)	6.6 (2.7–10.6)	6.6 (2.7–10.5)	7.0 (4.7–9.3)	8.3 (5.9–10.9)	8.1 (5.4–11.4)	8.1 (5.3–11.4)	10.4 (6.6–19.7)	11.0 (7.1–20.7)	8.5 (5.3–12.0)	7.4 (4.5–10.5)	13.3 (7.5–19.4)	13.4 (7.7–19.7)	2.5 (<0.1–23.6)	2.5 (<0.1–23.6)	0.2 (<0.1–0.7)	0.2 (<0.1–0.8)
Seychelles	54.1 (33.3–67.6)	50.6 (31.6–64.3)	4.7 (0.1–15.9)	4.4 (0.0–15.3)	0.1 (<0.1–0.2)	0.1 (<0.1–0.3)	15.2 (11.2–20.1)	16.9 (12.4–21.8)	3.9 (1.6–6.3)	3.9 (1.6–6.3)	10.9 (8.0–14.5)	12.7 (9.2–16.7)	8.2 (3.9–14.3)	8.4 (4.0–14.6)	3.2 (1.5–7.2)	3.0 (1.5–6.7)	7.7 (4.3–11.1)	8.0 (4.7–11.7)	11.2 (6.1–16.3)	11.9 (6.8–17.4)	1.0 (<0.1–3.8)	1.0 (<0.1–3.8)	0.2 (<0.1–1.3)	0.2 (<0.1–1.3)
Sri Lanka	69.2 (52.1–80.4)	70.1 (53.5–80.7)	7.0 (0.1–22.8)	7.0 (0.1–22.5)	10.0 (3.8–20.0)	10.0 (3.7–20.1)	11.1 (6.7–16.8)	9.5 (5.6–14.7)	5.0 (2.0–8.1)	5.0 (2.0–8.1)	7.7 (4.6–11.9)	7.0 (4.1–10.9)	9.7 (5.8–14.9)	9.7 (5.9–14.8)	9.5 (5.7–18.9)	9.4 (5.5–18.9)	3.6 (2.1–5.4)	3.7 (2.1–5.4)	6.6 (2.7–11.4)	6.4 (2.6–11.2)	1.2 (<0.1–13.3)	1.2 (<0.1–13.3)	<0.1 (<0.1–0.8)	<0.1 (<0.1–0.8)
Thailand	58.0 (37.4–71.0)	59.4 (38.3–72.8)	7.1 (0.2–22.0)	7.4 (0.3–22.5)	3.6 (1.3–7.8)	3.6 (1.3–7.8)	14.4 (9.8–20.4)	12.0 (7.7–18.4)	7.5 (3.1–12.1)	7.5 (3.1–12.1)	10.0 (6.7–14.2)	9.1 (5.8–14.0)	13.8 (9.1–19.7)	13.8 (8.9–19.7)	5.1 (2.8–10.6)	5.3 (3.0–11.2)	12.2 (7.5–17.5)	11.3 (6.8–16.2)	10.7 (5.9–15.8)	10.6 (5.8–15.8)	2.9 (<0.1–10.6)	2.9 (<0.1–10.6)	<0.1 (<0.1–1.0)	<0.1 (<0.1–1.0)
Timor-Leste	66.5 (48.9–77.2)	72.8 (56.2–82.3)	14.9 (1.2–37.3)	18.5 (2.3–43.6)	30.2 (17.1–44.9)	30.1 (17.1–44.9)	15.2 (9.3–23.6)	10.7 (6.6–17.6)	6.6 (2.7–10.6)	6.6 (2.7–10.6)	10.5 (6.2–16.6)	7.9 (4.7–12.8)	5.5 (2.6–9.8)	5.5 (2.6–10.0)	15.1 (9.5–26.1)	18.2 (11.6–32.1)	6.2 (3.5–9.3)	6.3 (3.6–9.2)	10.8 (5.1–17.2)	10.9 (5.2–17.2)	0.5 (<0.1–2.1)	0.5 (<0.1–2.1)	<0.1 (<0.1–1.7)	<0.1 (<0.1–1.7)

	Child wasting		Child stunting		Household air pollution		Low birth weight		Handwashing		Short gestation		Ambient particulate matter		Child underweight		Non-exclusive breastfeeding		Secondhand smoke		High temperature		Low temperature	
	**M**	**F**	**M**	**F**	**M**	**F**	**M**	**F**	**M**	**F**	**M**	**F**	**M**	**F**	**M**	**F**	**M**	**F**	**M**	**F**	**M**	**F**	**M**	**F**
Viet Nam	51.5 (34.3–64.4)	60.9 (42.5–72.8)	8.1 (0.3–23.4)	9.2 (0.4–27.2)	10.1 (4.4–18.0)	10.0 (4.2–18.3)	16.2 (11.4–22.4)	10.3 (6.7–15.1)	8.6 (3.6–13.6)	8.6 (3.6–13.6)	12.3 (8.4–17.5)	8.3 (5.5–12.1)	9.6 (6.2–14.0)	9.7 (6.4–14.0)	5.9 (3.4–12.1)	6.9 (4.1–13.4)	8.9 (4.1–14.3)	9.9 (4.6–15.7)	15.9 (9.8–22.1)	15.9 (9.7–22.5)	2.7 (0.9–5.9)	2.7 (0.9–5.9)	0.2 (<0.1–3.2)	0.2 (<0.1–3.2)
**Sub-Saharan Africa**	**56.2 (39.6–65.4)**	**59.8 (42.5–69.2)**	**12.7 (1.2–31.5)**	**14.1 (1.3–34.9)**	**41.5 (29.7–54.1)**	**41.7 (29.4–54.2)**	**18.6 (16.5–21.1)**	**15.7 (13.7–18.2)**	**22.1 (10.2–32.5)**	**22.1 (10.2–32.6)**	**14.9 (13.2–16.9)**	**13.6 (11.9–15.8)**	**9.8 (5.6–15.8)**	**9.7 (5.4–15.7)**	**10.9 (7.1–19.5)**	**11.6 (7.7–20.7)**	**7.4 (3.8–11.4)**	**7.4 (3.8–11.4)**	**3.6 (1.8–5.6)**	**3.6 (1.8–5.8)**	**6.1 (1.0–20.8)**	**6.0 (1.0–19.7)**	**1.1 (<0.1–3.5)**	**1.1 (<0.1–3.6)**
Central Sub-Saharan Africa	48.0 (30.6–62.0)	53.1 (35.2–66.2)	9.9 (0.6–26.1)	12.6 (1.0–31.3)	37.4 (24.9–49.6)	37.3 (24.6–49.3)	22.6 (15.8–29.8)	17.6 (12.3–23.4)	22.5 (10.4–33.1)	22.6 (10.4–33.1)	18.3 (12.8–24.4)	16.2 (11.2–21.8)	8.3 (4.0–14.0)	8.4 (4.0–14.2)	6.7 (4.1–13.1)	8.6 (5.3–16.4)	6.3 (3.5–9.6)	6.5 (3.7–9.8)	3.1 (1.4–5.2)	3.1 (1.3–5.2)	1.8 (0.5–3.7)	1.7 (0.4–3.6)	<0.1 (<0.1–2.3)	<0.1 (<0.1–2.4)
Angola	46.2 (26.5–62.2)	49.8 (29.1–64.7)	10.9 (0.6–30.4)	14.4 (1.1–36.9)	18.2 (9.8–29.0)	18.2 (9.6–29.0)	21.2 (11.2–33.8)	14.8 (8.9–22.9)	22.3 (10.2–32.8)	22.3 (10.2–32.8)	17.2 (9.3–27.2)	13.5 (8.1–21.0)	10.7 (5.6–17.3)	10.8 (5.7–17.7)	6.8 (3.9–13.4)	8.2 (4.9–15.7)	6.9 (3.8–10.9)	6.6 (3.5–10.3)	7.2 (3.8–10.9)	7.1 (3.8–10.7)	1.7 (0.5–3.8)	1.7 (0.5–3.8)	<0.1 (<0.1–2.9)	<0.1 (<0.1–2.9)
Central African Republic	51.2 (32.7–65.1)	58.2 (37.8–73.0)	11.2 (1.1–28.1)	15.6 (1.6–36.7)	50.0 (35.8–62.2)	49.9 (35.9–62.3)	21.5 (13.7–31.8)	14.5 (8.5–22.8)	21.6 (9.9–31.8)	21.6 (9.9–31.8)	16.1 (10.1–24.0)	11.8 (6.8–18.7)	6.3 (2.0–13.3)	6.3 (2.0–13.5)	8.1 (4.5–15.8)	10.2 (6.3–18.6)	8.0 (4.0–12.7)	7.7 (3.8–12.7)	3.2 (1.5–5.6)	3.3 (1.5–5.5)	3.8 (1.5–7.0)	3.8 (1.5–7.0)	<0.1 (<0.1–1.2)	<0.1 (<0.1–1.2)
Congo	40.7 (22.8–57.3)	38.0 (19.4–55.0)	6.1 (0.2–17.6)	6.7 (0.3–19.8)	15.4 (7.9–26.1)	15.4 (8.1–26.1)	30.7 (18.3–44.2)	30.2 (18.8–44.8)	21.0 (9.5–31.0)	21.0 (9.5–31.0)	25.9 (15.7–37.2)	27.9 (17.1–42.0)	15.4 (8.5–26.1)	15.5 (8.5–26.3)	4.4 (2.2–9.0)	4.9 (2.3–9.8)	8.1 (4.6–12.7)	8.4 (4.4–13.6)	4.2 (1.9–7.0)	4.1 (2.0–6.6)	0.9 (<0.1–3.0)	0.9 (<0.1–3.0)	<0.1 (<0.1–1.9)	<0.1 (<0.1–1.9)
Democratic Republic of the Congo	47.8 (30.5–64.1)	53.5 (36.2–68.8)	9.3 (0.4–23.7)	11.3 (0.9–29.0)	43.0 (28.6–55.6)	43.0 (28.3–55.6)	23.5 (14.1–34.6)	19.1 (11.6–28.7)	23.0 (10.6–33.7)	23.0 (10.6–33.7)	19.3 (11.4–28.4)	18.1 (10.8–27.0)	7.5 (3.2–13.7)	7.6 (3.3–14.0)	6.4 (3.6–12.4)	8.5 (5.1–16.4)	5.5 (2.9–8.6)	6.2 (3.3–9.5)	1.4 (0.4–2.8)	1.4 (0.4–2.8)	1.3 (0.1–3.1)	1.3 (0.1–3.1)	0.1 (<0.1–2.6)	0.1 (<0.1–2.6)
Equatorial Guinea	43.5 (25.1–60.3)	43.9 (24.1–61.7)	7.4 (0.5–22.5)	6.9 (0.4–19.6)	4.6 (1.7–9.2)	4.6 (1.8–9.3)	28.4 (16.8–42.4)	29.9 (17.5–45.8)	19.9 (9.0–29.7)	19.9 (9.0–29.7)	26.2 (15.3–39.1)	29.6 (17.1–45.4)	20.8 (11.2–33.1)	20.8 (11.3–33.2)	2.9 (1.4–6.3)	3.0 (1.3–6.7)	9.0 (4.8–14.1)	9.1 (5.0–14.3)	3.9 (1.6–6.9)	4.0 (1.6–7.0)	0.4 (<0.1–1.3)	0.4 (<0.1–1.3)	<0.1 (<0.1–2.4)	<0.1 (<0.1–2.4)
Gabon	36.2 (19.3–53.1)	31.8 (15.1–48.1)	5.0 (0.2–17.0)	5.5 (0.2–17.3)	1.7 (0.6–3.4)	1.8 (0.7–3.6)	33.1 (20.9–49.5)	38.0 (24.0–55.2)	16.9 (7.4–25.6)	16.9 (7.4–25.6)	28.2 (17.5–42.1)	34.7 (21.6–50.7)	18.2 (10.4–29.8)	18.3 (10.2–29.8)	2.4 (1.0–5.5)	2.9 (1.3–5.9)	9.9 (5.1–15.7)	10.1 (5.5–16.0)	4.6 (2.0–7.6)	4.7 (2.1–7.7)	0.7 (<0.1–2.6)	0.7 (<0.1–2.6)	<0.1 (<0.1–1.5)	<0.1 (<0.1–1.5)
Eastern Sub-Saharan Africa	54.4 (35.9–65.1)	56.6 (38.3–67.2)	12.2 (1.0–31.0)	12.9 (1.0–33.6)	46.7 (33.5–59.7)	46.2 (32.5–59.4)	17.3 (14.8–20.2)	15.7 (13.0–18.7)	22.6 (10.4–33.3)	22.6 (10.4–33.3)	14.3 (12.2–16.7)	13.6 (11.2–16.3)	5.3 (2.7–8.9)	5.4 (2.8–9.1)	8.8 (5.6–16.9)	9.6 (6.3–18.0)	6.3 (3.2–9.6)	6.3 (3.4–9.6)	3.6 (1.5–6.1)	3.6 (1.5–6.4)	2.3 (0.3–7.0)	2.2 (0.3–6.1)	1.0 (<0.1–3.3)	1.1 (<0.1–3.4)
Burundi	53.1 (35.7–68.1)	56.7 (37.8–72.0)	14.4 (1.2–35.9)	19.1 (2.5–42.3)	51.2 (37.4–63.5)	51.2 (37.5–63.5)	21.1 (11.8–31.5)	15.4 (8.8–24.8)	22.9 (10.6–33.7)	22.9 (10.6–33.7)	16.1 (9.0–24.2)	13.3 (7.5–21.3)	4.6 (1.5–10.1)	4.7 (1.5–10.2)	10.9 (6.6–20.9)	13.6 (8.4–24.7)	3.0 (1.5–4.9)	2.9 (1.4–4.8)	2.3 (0.8–4.3)	2.3 (0.8–4.3)	<0.1 (<0.1–0.3)	<0.1 (<0.1–0.3)	<0.1 (<0.1–6.1)	<0.1 (<0.1–6.1)
Comoros	47.4 (30.0–62.6)	54.4 (36.3–68.6)	9.1 (0.7–24.2)	13.3 (1.5–33.1)	34.3 (20.7–48.8)	34.2 (20.7–48.8)	30.0 (19.1–43.1)	24.2 (15.4–37.3)	21.0 (9.6–31.1)	21.0 (9.6–31.1)	24.5 (15.2–35.6)	20.9 (13.2–32.6)	5.2 (2.9–8.5)	5.3 (2.9–8.6)	5.8 (3.2–11.4)	8.2 (4.7–15.9)	9.7 (5.7–14.1)	9.3 (5.4–14.2)	5.0 (2.3–8.3)	5.0 (2.3–8.0)	0.7 (<0.1–2.9)	0.7 (<0.1–2.9)	0.2 (<0.1–2.9)	0.2 (<0.1–2.9)
Djibouti	63.8 (46.5–75.6)	66.3 (49.4–77.9)	12.1 (1.1–30.9)	14.0 (1.6–36.3)	8.6 (3.8–16.3)	8.5 (3.7–16.1)	14.7 (9.0–22.2)	14.6 (8.5–22.9)	21.6 (9.9–32.0)	21.6 (9.9–32.0)	12.8 (7.8–19.3)	13.0 (7.6–20.6)	18.0 (9.1–31.0)	17.9 (8.8–31.0)	11.2 (7.0–19.7)	9.4 (5.8–18.1)	9.0 (4.0–15.1)	9.4 (4.2–15.7)	8.4 (3.8–14.0)	8.3 (3.7–13.6)	5.9 (<0.1–30.0)	5.9 (<0.1–30.0)	0.2 (<0.1–5.2)	0.2 (<0.1–5.2)

	**Child wasting**		**Child stunting**		**Household air pollution**		**Low birth weight**		**Handwashing**		**Short gestation**		**Ambient particulate matter**		**Child underweight**		**Non-exclusive breastfeeding**		**Secondhand smoke**		**High temperature**		**Low temperature**	
	**M**	**F**	**M**	**F**	**M**	**F**	**M**	**F**	**M**	**F**	**M**	**F**	**M**	**F**	**M**	**F**	**M**	**F**	**M**	**F**	**M**	**F**	**M**	**F**
Eritrea	60.0 (43.0–73.2)	65.8 (48.0–78.1)	13.2 (0.8–32.6)	14.6 (1.1–36.0)	33.7 (20.0–46.4)	33.7 (19.8–46.4)	18.7 (11.7–27.5)	12.7 (7.1–20.4)	22.6 (10.4–33.1)	22.6 (10.4–33.1)	14.9 (9.2–22.0)	11.2 (6.2–18.0)	11.0 (5.1–19.1)	11.1 (5.1–19.2)	9.6 (5.1–18.7)	13.7 (8.5–25.1)	3.7 (1.2–7.0)	3.8 (1.2–7.3)	3.9 (2.0–6.1)	4.0 (2.1–6.2)	4.5 (<0.1–22.1)	4.5 (<0.1–22.1)	0.1 (<0.1–2.5)	0.1 (<0.1–2.5)
Ethiopia	52.3 (36.6–62.8)	56.3 (42.3–66.5)	11.2 (1.0–28.1)	13.3 (1.3–32.6)	49.6 (36.4–61.3)	49.5 (36.1–61.4)	23.1 (18.3–28.3)	19.8 (15.4–25.4)	23.2 (10.7–33.9)	23.2 (10.7–34.0)	17.8 (14.1–22.0)	17.1 (13.2–22.0)	5.1 (2.1–9.6)	5.2 (2.2–9.8)	8.6 (5.4–16.1)	11.7 (7.8–21.3)	4.4 (2.2–7.1)	4.5 (2.3–7.3)	1.4 (0.5–2.6)	1.3 (0.5–2.6)	3.7 (0.7–12.7)	3.6 (0.7–11.7)	4.0 (2.5–5.8)	4.0 (2.5–6.0)
Kenya	43.0 (27.2–52.5)	47.5 (31.4–57.0)	8.2 (0.6–22.6)	9.8 (0.8–25.8)	37.2 (25.0–49.2)	37.1 (25.2–48.9)	26.6 (23.3–29.9)	23.1 (20.0–26.8)	22.5 (10.4–33.1)	22.5 (10.4–33.1)	22.2 (19.4–25.2)	21.1 (18.2–24.4)	6.2 (3.6–9.9)	6.2 (3.6–9.9)	6.2 (4.0–11.9)	7.7 (4.8–14.6)	7.5 (3.9–11.6)	7.9 (4.2–11.9)	3.9 (1.7–6.6)	4.0 (1.7–7.0)	4.2 (0.3–16.6)	4.3 (0.3–17.5)	2.4 (1.0–4.1)	2.4 (1.0–4.2)
Madagascar	49.7 (32.9–63.9)	55.2 (39.1–68.2)	11.9 (1.2–28.8)	15.3 (1.8–36.2)	44.6 (29.4–58.1)	44.6 (29.2–58.1)	29.7 (19.3–41.6)	24.9 (16.0–35.2)	22.4 (10.3–33.0)	22.4 (10.3–33.0)	23.6 (15.3–33.0)	21.2 (13.4–30.4)	4.2 (2.1–7.2)	4.2 (2.1–7.2)	8.8 (5.3–16.8)	10.7 (6.4–19.5)	6.0 (3.1–9.6)	6.2 (3.3–9.7)	3.4 (1.1–6.7)	3.5 (1.1–6.7)	0.8 (<0.1–2.4)	0.8 (<0.1–2.4)	1.0 (<0.1–3.8)	1.0 (<0.1–3.8)
Malawi	45.5 (25.0–61.0)	49.9 (28.4–64.6)	12.2 (0.6–32.0)	14.9 (1.4–37.2)	46.8 (31.4–58.9)	46.8 (31.5–58.9)	19.1 (13.0–27.5)	16.0 (10.5–22.8)	22.6 (10.4–33.3)	22.6 (10.4–33.3)	15.5 (10.3–22.5)	14.1 (9.2–20.3)	4.6 (2.0–8.5)	4.6 (2.1–8.6)	7.0 (4.1–14.2)	6.2 (3.5–12.6)	4.9 (2.8–7.3)	4.8 (2.8–7.3)	3.3 (1.4–5.6)	3.4 (1.4–6.1)	0.5 (<0.1–1.9)	0.5 (<0.1–1.9)	0.2 (<0.1–4.4)	0.2 (<0.1–4.4)
Mozambique	56.2 (34.0–70.7)	54.8 (33.3–69.5)	11.9 (0.5–33.3)	10.0 (0.1–31.2)	50.3 (36.0–62.0)	50.3 (36.2–61.9)	13.0 (7.5–20.0)	12.8 (8.0–20.3)	22.2 (10.2–32.7)	22.2 (10.2–32.7)	9.6 (5.5–14.9)	10.8 (6.8–17.3)	3.5 (1.4–6.7)	3.5 (1.4–6.7)	6.4 (3.6–13.5)	7.9 (4.7–15.5)	8.9 (4.9–13.3)	8.9 (5.1–13.3)	3.6 (1.2–7.0)	3.6 (1.2–7.0)	2.4 (1.0–4.4)	2.4 (1.0–4.4)	0.2 (<0.1–3.3)	0.2 (<0.1–3.3)
Rwanda	50.6 (30.0–64.9)	52.4 (32.1–68.1)	12.1 (0.6–32.1)	14.7 (1.3–37.3)	39.9 (25.4–54.4)	39.9 (25.6–54.4)	12.9 (8.5–18.2)	10.7 (6.5–16.2)	23.2 (10.7–34.0)	23.2 (10.7–34.0)	11.2 (7.4–15.3)	11.3 (6.9–16.8)	8.8 (3.9–16.4)	8.9 (3.9–16.4)	5.8 (3.2–11.8)	6.6 (3.8–13.1)	1.7 (0.9–2.7)	1.6 (0.8–2.5)	4.1 (2.0–6.5)	4.2 (2.1–6.6)	<0.1 (<0.1–0.1)	<0.1 (<0.1–0.1)	2.0 (<0.1–7.5)	2.0 (<0.1–7.5)
Somalia	63.8 (47.7–75.2)	66.5 (49.0–77.9)	12.7 (1.1–32.8)	11.4 (0.8–31.2)	60.3 (46.2–83.5)	60.3 (46.2–83.5)	9.0 (5.7–13.1)	12.4 (7.3–19.8)	22.7 (10.5–33.4)	22.7 (10.5–33.4)	11.9 (7.6–17.7)	10.3 (6.0–16.5)	1.6 (0.3–5.0)	1.6 (0.3–5.0)	13.1 (8.2–23.9)	12.4 (7.6–23.0)	10.0 (3.7–16.9)	10.3 (3.8–18.3)	3.5 (1.5–6.3)	3.4 (1.4–6.0)	2.3 (<0.1–15.8)	2.3 (<0.1–15.8)	0.1 (<0.1–1.3)	0.1 (<0.1–1.3)
South Sudan	64.5 (48.2–76.3)	66.3 (48.5–77.7)	12.9 (1.2–32.8)	12.6 (1.3–32.4)	44.4 (28.9–57.3)	44.4 (28.7–57.3)	14.5 (9.1–21.7)	12.8 (8.2–19.3)	22.9 (10.6–33.6)	22.9 (10.6–33.6)	10.7 (6.5–16.0)	10.3 (6.4–15.3)	7.7 (3.5–14.1)	7.7 (3.6–14.1)	12.8 (8.4–22.9)	12.1 (7.7–22.4)	7.2 (3.5–11.7)	7.2 (3.4–11.8)	3.7 (1.5–6.3)	3.7 (1.6–6.3)	5.9 (<0.1–23.1)	5.9 (<0.1–23.1)	<0.1 (<0.1–0.9)	<0.1 (<0.1–0.9)
Uganda	51.6 (29.8–67.1)	49.9 (28.2–65.5)	11.2 (0.6–30.2)	10.4 (0.6–28.9)	42.1 (27.3–55.4)	42.2 (27.0–55.3)	14.9 (8.5–23.5)	15.3 (8.7–23.9)	22.1 (10.1–32.5)	22.1 (10.1–32.5)	11.7 (6.7–18.9)	13.6 (7.7–21.1)	8.0 (3.9–13.2)	8.0 (3.9–13.1)	6.6 (3.7–13.6)	6.5 (3.6–13.3)	4.2 (2.2–6.8)	4.3 (2.4–6.9)	3.3 (1.3–5.8)	3.3 (1.4–5.8)	1.1 (0.2–2.9)	1.1 (0.2–2.9)	<0.1 (<0.1–2.3)	<0.1 (<0.1–2.3)
United Republic of Tanzania	51.7 (30.9–66.1)	55.6 (32.9–70.6)	13.6 (1.3–34.7)	14.0 (1.0–36.7)	41.6 (26.2–55.7)	41.6 (26.5–55.7)	15.1 (9.6–22.7)	11.7 (7.3–18.0)	22.5 (10.4–33.2)	22.5 (10.4–33.2)	11.5 (7.1–17.2)	10.0 (6.2–15.6)	6.2 (3.3–10.1)	6.3 (3.3–10.2)	7.2 (4.2–14.4)	7.5 (4.5–14.9)	5.6 (3.1–8.6)	5.7 (3.2–8.7)	6.2 (2.9–10.0)	6.2 (3.0–9.9)	0.9 (0.1–2.7)	0.9 (0.1–2.7)	0.1 (<0.1–3.5)	0.1 (<0.1–3.5)
Zambia	52.8 (32.1–66.6)	52.9 (31.2–67.7)	14.6 (1.3–37.2)	15.0 (1.2–38.3)	28.6 (16.8–43.0)	28.6 (16.8–43.0)	16.3 (11.2–23.0)	13.8 (8.6–20.0)	21.3 (9.7–31.5)	21.3 (9.7–31.5)	13.7 (9.4–19.3)	12.6 (7.9–18.6)	8.8 (5.1–13.9)	8.8 (5.2–14.0)	6.9 (4.1–13.8)	6.8 (3.9–13.6)	5.4 (3.0–8.3)	5.8 (3.5–8.6)	4.6 (1.4–9.0)	4.5 (1.4–8.8)	1.2 (0.3–3.5)	1.2 (0.3–3.5)	<0.1 (<0.1–5.0)	<0.1 (<0.1–5.0)
Southern Sub-Saharan Africa	50.0 (29.4–62.8)	54.7 (34.3–67.2)	9.8 (0.5–27.0)	10.6 (0.6–29.3)	17.1 (9.9–26.2)	17.1 (9.8–26.2)	18.1 (15.1–21.7)	15.2 (12.5–18.6)	18.4 (8.3–27.3)	18.3 (8.3–27.1)	16.9 (14.1–20.3)	15.1 (12.4–18.6)	10.5 (7.0–15.1)	10.4 (6.9–15.1)	5.3 (3.2–10.7)	5.4 (3.2–11.2)	9.4 (6.0–13.4)	9.8 (6.4–13.8)	7.4 (3.9–11.3)	7.6 (4.0–11.7)	1.3 (0.6–2.7)	1.3 (0.6–2.8)	5.2 (2.0–8.3)	5.1 (2.0–8.2)

	Child wasting		Child stunting		Household air pollution		Low birth weight		Handwashing		Short gestation		Ambient particulate matter		Child underweight		Non-exclusive breastfeeding		Secondhand smoke		High temperature		Low temperature	
	M	F	M	F	M	F	M	F	M	F	M	F	M	F	M	F	M	F	M	F	M	F	M	F
Botswana	54.6 (31.9–69.3)	58.7 (37.5–73.5)	13.1 (0.9–34.4)	12.4 (0.8–33.8)	8.9 (3.8–16.7)	8.8 (3.7–16.7)	14.3 (8.9–21.5)	11.8 (6.4–20.4)	17.0 (7.5–25.6)	17.0 (7.5–25.6)	14.0 (8.8–21.2)	12.4 (6.6–21.8)	12.1 (7.4–18.0)	12.1 (7.5–18.1)	4.8 (2.4–10.3)	5.8 (3.4–12.0)	6.8 (3.6–10.5)	6.5 (3.1–10.9)	8.8 (4.3–14.1)	8.9 (4.4–14.2)	4.0 (1.5–7.7)	4.0 (1.5–7.7)	0.9 (<0.1–7.0)	0.9 (<0.1–7.0)
Eswatini	46.3 (22.4–63.3)	38.1 (16.5–56.2)	9.0 (0.3–27.0)	12.3 (0.8–33.9)	15.7 (7.4–27.0)	15.7 (7.5–27.0)	8.4 (4.5–13.9)	10.8 (6.0–18.8)	16.5 (7.4–25.0)	16.5 (7.4–25.0)	8.3 (4.5–13.8)	11.0 (6.1–19.3)	10.2 (6.2–15.7)	10.2 (6.2–15.4)	4.4 (2.5–9.5)	4.4 (2.4–9.4)	9.0 (5.4–12.9)	9.6 (5.8–13.8)	2.4 (0.9–4.6)	2.5 (0.9–4.4)	0.4 (<0.1–1.2)	0.4 (<0.1–1.2)	3.3 (<0.1–8.1)	3.3 (<0.1–8.1)
Lesotho	58.2 (38.1–73.0)	54.7 (33.5–70.6)	10.1 (0.4–29.0)	12.8 (0.8–34.0)	22.0 (12.3–34.1)	22.0 (12.3–34.2)	14.9 (8.4–23.1)	16.3 (8.7–27.0)	23.1 (10.7–33.9)	23.1 (10.7–33.9)	13.6 (7.6–21.0)	16.1 (8.6–26.7)	10.4 (6.2–16.1)	10.3 (6.2–16.0)	5.6 (3.1–11.7)	7.6 (4.5–15.0)	6.3 (3.5–9.7)	6.1 (3.2–9.5)	10.6 (6.1–15.6)	10.6 (6.0–15.4)	<0.1 (<0.1–<0.1)	<0.1 (<0.1–<0.1)	21.7 (15.2–28.4)	21.7 (15.2–28.4)
Namibia	56.7 (35.3–70.7)	61.8 (39.7–75.9)	7.7 (0.2–22.7)	9.1 (0.2–27.2)	12.0 (5.6–21.6)	12.0 (5.4–21.6)	16.3 (10.2–24.8)	12.8 (6.8–22.7)	14.3 (6.2–22.0)	14.3 (6.2–22.0)	15.1 (9.3–22.9)	12.4 (6.6–21.9)	11.1 (6.4–17.0)	11.1 (6.3–17.1)	5.3 (2.7–11.2)	7.3 (4.3–14.8)	7.9 (4.6–11.8)	8.7 (5.1–13.2)	4.5 (1.7–8.1)	4.6 (1.8–8.2)	3.2 (1.3–6.0)	3.2 (1.3–6.0)	1.3 (<0.1–6.0)	1.3 (<0.1–6.0)
South Africa	52.3 (32.6–64.2)	62.2 (43.0–72.3)	11.1 (0.8–29.4)	10.4 (0.5–30.3)	3.7 (1.6–7.0)	3.5 (1.5–6.8)	18.7 (16.2–21.6)	14.2 (12.1–16.8)	15.3 (6.7–23.2)	15.1 (6.7–23.0)	18.1 (15.6–20.7)	14.4 (12.2–17.1)	14.3 (9.7–20.4)	14.2 (9.3–20.5)	5.7 (3.4–11.2)	5.2 (3.1–10.5)	11.0 (7.5–15.0)	12.2 (8.4–16.6)	7.3 (3.4–11.8)	7.6 (3.6–12.3)	0.8 (0.4–2.1)	0.8 (0.3–2.1)	8.6 (6.3–11.1)	8.8 (6.5–11.2)
Zimbabwe	46.4 (25.9–61.5)	48.3 (26.2–63.0)	8.6 (0.3–25.0)	10.3 (0.7–28.4)	29.1 (17.5–43.3)	29.1 (17.4–43.3)	18.9 (12.8–26.3)	16.7 (11.4–23.3)	20.9 (9.6–31.0)	20.9 (9.6–31.0)	17.2 (11.6–24.1)	16.4 (11.2–22.9)	7.1 (4.1–11.1)	7.1 (4.1–11.1)	5.0 (2.8–10.4)	5.3 (3.0–11.0)	8.6 (5.0–13.0)	8.3 (4.8–12.4)	7.5 (4.1–11.2)	7.6 (4.2–11.4)	1.7 (0.7–3.8)	1.7 (0.7–3.8)	0.7 (<0.1–5.1)	0.7 (<0.1–5.1)
Western Sub-Saharan Africa	58.0 (42.6–66.9)	62.0 (45.6–70.8)	13.4 (1.4–32.5)	14.9 (1.6–35.6)	40.9 (28.7–53.5)	41.2 (29.1–53.7)	18.8 (16.5–21.6)	15.5 (13.4–18.1)	22.1 (10.1–32.4)	22.0 (10.1–32.4)	14.7 (12.9–16.9)	13.3 (11.4–15.5)	11.8 (6.3–19.6)	11.7 (6.2–19.6)	12.3 (8.1–21.6)	13.0 (8.6–23.1)	7.9 (3.9–12.4)	7.9 (3.9–12.4)	3.4 (1.8–5.3)	3.5 (1.8–5.4)	8.3 (1.3–28.7)	8.2 (1.1–28.5)	1.1 (<0.1–4.4)	1.1 (<0.1–4.5)
Benin	60.3 (41.7–74.2)	63.7 (45.9–75.8)	15.2 (1.6–37.4)	17.4 (1.8–41.4)	42.0 (27.6–54.9)	42.0 (27.6–55.0)	14.7 (8.4–23.0)	13.6 (8.2–20.8)	22.1 (10.1–32.6)	22.1 (10.1–32.6)	10.9 (6.1–17.1)	11.1 (6.7–16.8)	9.5 (4.3–16.4)	9.5 (4.3–16.3)	9.1 (5.6–17.5)	10.3 (6.4–19.3)	6.0 (2.8–9.6)	6.2 (3.0–10.3)	3.6 (1.7–5.7)	3.7 (1.8–6.0)	3.3 (<0.1–18.5)	3.3 (<0.1–18.5)	<0.1 (<0.1–1.2)	<0.1 (<0.1–1.2)
Burkina Faso	65.8 (50.5–76.2)	69.3 (53.4–79.0)	12.2 (0.9–32.8)	13.5 (1.1–34.9)	49.4 (36.1–63.0)	49.4 (35.9–63.0)	16.7 (10.6–25.0)	12.6 (8.2–18.8)	22.2 (10.2–32.7)	22.2 (10.2–32.7)	12.9 (8.0–19.3)	10.5 (6.7–15.7)	6.7 (2.1–14.2)	6.7 (2.1–14.4)	11.2 (7.1–20.8)	13.1 (8.4–23.9)	7.1 (2.5–12.4)	6.7 (2.4–12.0)	3.8 (1.5–6.7)	3.9 (1.6–6.8)	4.6 (<0.1–34.1)	4.6 (<0.1–34.1)	<0.1 (<0.1–3.1)	<0.1 (<0.1–3.1)
Cabo Verde	52.9 (30.1–69.0)	54.2 (31.6–68.7)	5.2 (0.0–17.1)	5.4 (0.0–18.2)	7.3 (3.5–13.3)	7.3 (3.4–13.3)	16.5 (9.5–30.6)	14.7 (8.8–25.4)	19.0 (8.5–28.3)	19.0 (8.5–28.3)	14.7 (8.4–26.7)	14.1 (8.5–24.3)	21.3 (13.4–31.1)	21.3 (13.4–31.1)	3.7 (1.7–8.0)	4.1 (2.0–9.0)	8.0 (4.7–12.0)	8.2 (4.8–11.8)	3.9 (1.8–6.5)	3.9 (1.8–6.4)	0.2 (<0.1–1.1)	0.2 (<0.1–1.1)	<0.1 (<0.1–6.3)	<0.1 (<0.1–6.3)
Cameroon	51.3 (31.1–67.0)	53.8 (32.7–69.9)	10.3 (0.6–27.6)	11.7 (1.0–31.7)	23.8 (13.5–35.7)	23.8 (13.7–35.9)	16.5 (9.6–25.9)	13.2 (6.8–22.1)	21.8 (10.0–32.1)	21.8 (10.0–32.1)	14.2 (8.1–22.5)	13.5 (7.0–22.9)	18.9 (11.8–27.2)	19.0 (11.9–27.1)	5.2 (2.9–10.9)	7.4 (4.4–15.0)	7.9 (3.7–12.8)	7.8 (3.6–13.7)	3.8 (1.8–6.1)	3.9 (1.9–6.4)	2.0 (0.1–7.1)	2.0 (0.1–7.1)	<0.1 (<0.1–1.6)	<0.1 (<0.1–1.6)
Chad	62.5 (45.1–74.0)	68.3 (51.2–80.2)	12.0 (1.2–28.8)	14.4 (1.5–36.3)	50.2 (36.9–64.5)	50.2 (37.0–64.5)	14.5 (9.6–21.5)	10.2 (5.3–17.1)	22.3 (10.2–32.8)	22.3 (10.2–32.8)	11.1 (7.2–16.6)	9.0 (4.8–15.0)	6.7 (2.3–14.9)	6.7 (2.3–15.1)	9.9 (6.1–19.1)	13.6 (8.7–24.8)	9.3 (3.7–16.2)	8.8 (3.4–15.7)	4.7 (2.1–7.8)	4.7 (2.1–7.8)	7.0 (0.4–22.9)	7.0 (0.4–22.9)	1.3 (<0.1–7.7)	1.3 (<0.1–7.7)
Côte d'Ivoire	46.5 (29.8–62.4)	50.1 (32.0–64.4)	9.0 (0.4–24.5)	10.4 (0.8–27.4)	34.4 (21.5–48.1)	34.3 (21.5–48.1)	28.7 (17.8–40.6)	25.2 (15.7–37.1)	20.8 (9.5–31.0)	20.8 (9.5–31.0)	22.8 (14.3–32.4)	21.7 (13.5–32.1)	13.2 (6.7–22.3)	13.3 (6.8–22.5)	5.8 (3.1–11.6)	6.7 (3.7–13.6)	6.9 (2.9–11.8)	6.9 (3.0–11.5)	6.8 (3.7–10.4)	6.8 (3.8–10.5)	1.6 (<0.1–5.7)	1.6 (<0.1–5.7)	0.2 (<0.1–1.2)	0.2 (<0.1–1.2)

	Child wasting		Child stunting		Household air pollution		Low birth weight		Handwashing		Short gestation		Ambient particulate matter		Child underweight		Non-exclusive breastfeeding		Secondhand smoke		High temperature		Low temperature	
	**M**	**F**	**M**	**F**	**M**	**F**	**M**	**F**	**M**	**F**	**M**	**F**	**M**	**F**	**M**	**F**	**M**	**F**	**M**	**F**	**M**	**F**	**M**	**F**
Gambia	53.5 (36.8–66.5)	52.9 (35.1–67.5)	8.7 (0.6–25.0)	6.9 (0.1–21.4)	39.7 (25.7–52.7)	39.7 (25.8–52.7)	24.5 (16.2–35.5)	20.5 (12.7–29.7)	21.9 (10.1–32.3)	21.9 (10.1–32.3)	18.8 (12.4–27.1)	18.8 (11.7–27.4)	11.9 (5.9–19.8)	12.0 (5.9–20.0)	10.3 (6.4–18.9)	5.6 (2.9–11.7)	4.9 (2.5–8.1)	4.8 (2.4–7.8)	7.4 (3.8–11.2)	7.4 (3.9–11.2)	2.2 (<0.1–20.9)	2.2 (<0.1–20.9)	0.1 (<0.1–1.5)	0.1 (<0.1–1.5)
Ghana	54.4 (35.6–69.1)	56.2 (37.7–71.2)	7.0 (0.1–21.9)	7.3 (0.2–22.9)	18.0 (9.5–29.9)	18.0 (9.3–29.9)	17.6 (10.4–27.2)	15.9 (9.2–25.4)	22.3 (10.3–32.8)	22.3 (10.3–32.8)	13.1 (7.8–20.4)	13.9 (7.8–22.1)	18.8 (12.0–28.5)	18.8 (11.8–28.4)	6.7 (3.9–13.1)	6.5 (3.4–12.7)	5.4 (2.7–8.8)	5.8 (2.9–9.4)	2.1 (1.0–3.5)	2.1 (1.0–3.5)	2.9 (<0.1–18.5)	2.9 (<0.1–18.5)	<0.1 (<0.1–0.7)	<0.1 (<0.1–0.7)
Guinea	58.9 (38.2–72.9)	61.4 (40.8–74.3)	12.3 (0.8–31.8)	12.8 (0.7–33.0)	47.1 (32.7–59.1)	47.1 (32.7–59.1)	13.5 (7.8–20.4)	11.5 (6.9–17.8)	22.3 (10.3–32.8)	22.3 (10.3–32.8)	10.1 (5.8–15.5)	9.8 (5.9–15.2)	8.1 (3.2–15.9)	8.1 (3.2–15.7)	9.2 (5.7–18.2)	9.8 (6.0–18.6)	7.5 (3.2–13.0)	6.8 (2.9–11.9)	5.1 (2.6–8.1)	5.0 (2.6–7.8)	2.7 (0.7–7.1)	2.7 (0.7–7.1)	0.1 (<0.1–1.3)	0.1 (<0.1–1.3)
Guinea-Bissau	49.8 (30.9–65.7)	55.4 (36.6–69.9)	10.1 (0.6–26.3)	11.6 (0.6–32.5)	45.1 (31.0–57.4)	45.0 (30.9–57.4)	25.0 (14.4–38.8)	19.8 (11.4–32.1)	22.3 (10.3–32.8)	22.3 (10.3–32.8)	19.4 (11.2–30.2)	16.8 (9.6–27.3)	9.1 (3.9–16.7)	9.2 (4.0–16.6)	6.5 (3.5–13.0)	8.7 (5.2–16.7)	5.1 (1.9–9.1)	5.0 (1.9–9.0)	4.8 (2.5–7.8)	4.7 (2.3–7.5)	2.2 (<0.1–22.7)	2.2 (<0.1–22.7)	<0.1 (<0.1–1.3)	<0.1 (<0.1–1.3)
Liberia	50.5 (30.8–66.4)	54.2 (33.1–69.2)	9.0 (0.6–25.3)	10.1 (0.6–27.0)	42.8 (28.6–55.1)	42.8 (28.6–55.0)	22.6 (13.5–33.4)	18.7 (10.7–28.8)	23.3 (10.8–34.1)	23.3 (10.8–34.1)	18.2 (10.8–27.0)	16.1 (9.1–25.1)	9.7 (4.5–17.1)	9.7 (4.5–17.0)	4.8 (2.6–9.8)	8.8 (5.4–16.6)	5.9 (3.1–9.2)	6.0 (3.1–9.6)	2.6 (1.1–4.5)	2.7 (1.1–4.6)	0.9 (<0.1–3.3)	0.9 (<0.1–3.3)	0.1 (<0.1–2.0)	0.1 (<0.1–2.0)
Mali	66.8 (48.5–78.8)	68.3 (48.2–79.0)	12.2 (0.9–32.7)	10.2 (0.4–29.1)	49.7 (35.8–63.4)	49.7 (35.7–63.4)	12.7 (7.6–19.6)	10.9 (6.8–15.7)	21.9 (10.1–32.3)	21.9 (10.1–32.3)	9.9 (5.9–15.5)	8.8 (5.4–12.7)	7.2 (2.4–15.6)	7.2 (2.4–15.6)	11.1 (7.1–21.0)	9.0 (5.4–17.8)	5.6 (2.4–9.5)	7.3 (3.3–12.4)	3.9 (1.7–6.5)	3.8 (1.7–6.5)	7.3 (<0.1–26.7)	7.3 (<0.1–26.7)	1.3 (<0.1–13.3)	1.3 (<0.1–13.3)
Mauritania	40.4 (23.5–58.8)	41.9 (25.5–58.2)	5.8 (0.3–17.0)	5.7 (0.3–16.7)	19.1 (10.3–30.5)	19.1 (10.3–30.5)	35.7 (21.5–49.3)	32.9 (21.0–44.8)	19.6 (8.8–29.2)	19.6 (8.8–29.2)	29.3 (17.6–41.1)	30.1 (19.0–41.5)	20.5 (11.6–31.5)	20.6 (11.6–31.5)	5.7 (2.9–11.7)	5.7 (2.9–11.3)	6.9 (3.2–11.9)	7.5 (3.6–12.5)	4.2 (1.9–6.9)	4.1 (1.8–7.0)	8.0 (1.7–19.5)	8.0 (1.7–19.5)	1.9 (<0.1–13.0)	1.9 (<0.1–13.0)
Niger	62.6 (46.2–74.3)	68.4 (50.9–79.2)	16.5 (1.7–38.7)	19.1 (2.3–43.4)	53.8 (38.7–73.9)	53.7 (38.5–73.9)	18.6 (12.0–27.3)	13.9 (8.5–21.4)	21.4 (9.8–31.7)	21.4 (9.8–31.7)	14.9 (9.6–21.6)	11.6 (7.1–17.8)	5.9 (1.5–15.4)	6.0 (1.5–15.6)	15.2 (9.8–26.6)	18.0 (11.8–30.6)	7.0 (2.7–12.3)	7.0 (2.6–12.7)	3.8 (1.8–6.0)	3.7 (1.8–5.9)	8.4 (0.5–23.3)	8.4 (0.5–23.3)	1.6 (<0.1–11.5)	1.6 (<0.1–11.5)
Nigeria	56.3 (41.6–64.4)	60.5 (45.0–68.7)	14.2 (1.8–32.8)	16.1 (1.9–36.8)	38.2 (26.2–50.9)	38.5 (26.6–51.0)	20.1 (17.9–23.1)	17.0 (14.8–19.8)	22.2 (10.2–32.6)	22.2 (10.2–32.7)	15.7 (13.9–18.1)	14.6 (12.7–17.1)	13.8 (7.3–22.8)	13.7 (7.3–23.0)	14.1 (9.4–24.0)	14.2 (9.4–24.4)	8.4 (4.5–12.9)	8.5 (4.4–13.0)	2.7 (1.5–4.1)	2.8 (1.5–4.2)	10.8 (2.2–43.0)	10.9 (2.2–43.1)	1.5 (<0.1–4.1)	1.5 (<0.1–4.1)
Sao Tome and Principe	50.3 (33.1–64.7)	56.0 (37.2–69.0)	10.1 (0.8–28.4)	10.8 (0.8–30.0)	21.5 (11.6–33.8)	21.4 (11.3–33.8)	23.3 (15.1–33.5)	19.5 (11.9–28.3)	17.4 (7.7–26.1)	17.4 (7.7–26.1)	19.6 (12.5–28.3)	17.1 (10.4–24.9)	11.3 (6.1–18.3)	11.3 (6.0–18.4)	4.2 (2.2–8.9)	6.6 (4.0–12.7)	5.4 (2.9–8.6)	5.6 (3.1–8.8)	1.3 (0.6–2.1)	1.4 (0.6–2.2)	0.9 (<0.1–3.7)	0.9 (<0.1–3.7)	<0.1 (<0.1–1.9)	<0.1 (<0.1–1.9)
Senegal	46.8 (28.0–62.3)	47.4 (28.9–62.7)	5.5 (0.1–17.0)	6.9 (0.3–21.4)	36.7 (23.5–49.1)	36.7 (23.2–48.8)	26.6 (17.1–39.2)	26.0 (15.9–39.1)	18.8 (8.4–28.1)	18.8 (8.4–28.1)	21.4 (13.7–31.5)	22.3 (13.5–33.7)	12.7 (6.3–21.1)	12.7 (6.3–21.1)	5.2 (2.8–10.8)	6.1 (3.3–12.2)	7.8 (3.0–13.4)	7.6 (2.9–13.4)	7.6 (4.3–11.2)	7.6 (4.3–11.3)	4.2 (<0.1–30.1)	4.2 (<0.1–30.1)	0.1 (<0.1–2.2)	0.1 (<0.1–2.2)
Sierra Leone	66.8 (46.0–79.3)	66.9 (46.1–79.4)	13.6 (0.8–36.3)	15.9 (1.4–39.8)	45.5 (31.1–58.0)	45.5 (31.0–58.0)	9.1 (5.4–14.1)	8.9 (5.0–14.1)	22.2 (10.2–32.6)	22.2 (10.2–32.6)	7.0 (4.2–10.9)	7.4 (4.1–11.8)	8.7 (3.7–16.4)	8.7 (3.8–16.5)	9.0 (5.5–17.9)	10.7 (6.8–20.6)	7.7 (4.2–12.0)	7.5 (3.9–11.9)	5.5 (2.2–10.1)	5.5 (2.1–9.8)	1.8 (<0.1–4.3)	1.8 (<0.1–4.3)	0.2 (<0.1–1.5)	0.2 (<0.1–1.5)
Togo	46.9 (28.4–64.2)	51.0 (31.5–66.7)	7.9 (0.4–23.8)	8.6 (0.3–25.4)	37.4 (23.7–50.8)	37.3 (23.6–50.8)	25.6 (14.2–39.1)	19.4 (11.0–30.4)	22.0 (10.1–32.5)	22.0 (10.1–32.5)	20.4 (11.3–31.6)	17.2 (9.7–26.9)	11.0 (6.0–17.6)	11.1 (6.1–18.1)	6.2 (3.3–13.0)	7.0 (3.8–13.9)	4.8 (2.3–8.2)	5.1 (2.5–8.4)	5.0 (2.8–7.4)	5.1 (2.9–7.6)	2.7 (<0.1–13.0)	2.7 (<0.1–13.0)	0.1 (<0.1–0.9)	0.1 (<0.1–0.9)

Appendix Table 8. Population-attributable fractions of lower respiratory infections due to evaluated risk factors among children aged 5-14 years in 2019, males (M) and females (F) (95% UI)

	**Household air pollution**		**Handwashing**		**Ambient particulate matter**		**Secondhand smoke**		**High temperature**		**Low temperature**	
	**M**	**F**	**M**	**F**	**M**	**F**	**M**	**F**	**M**	**F**	**M**	**F**
**Global**	**26.0 (16.6–35.5)**	**25.8 (16.3–35.4)**	**15.1 (6.7–22.7)**	**15.1 (6.7–22.7)**	**14.4 (10.0–19.7)**	**15.3 (10.7–20.8)**	**7.9 (4.5–11.5)**	**8.1 (4.6–11.9)**	**4.8 (2.3–10.1)**	**4.9 (2.4–9.9)**	**3.3 (0.5–6.2)**	**3.0 (0.1–6.0)**
**Central Europe, Eastern Europe, and Central Asia**	**2.7 (1.0–5.4)**	**2.5 (0.9–5.1)**	**4.2 (1.7–6.8)**	**4.1 (1.7–6.8)**	**13.9 (7.7–21.9)**	**13.6 (7.5–21.2)**	**13.3 (8.4–18.4)**	**13.8 (8.7–18.9)**	**0.7 (<0.1–1.7)**	**0.7 (<0.1–1.7)**	**18.2 (13.1–24.4)**	**18.2 (13.0–24.1)**
Central Asia	3.2 (1.3–6.4)	3.0 (1.1–6.3)	4.8 (1.9–7.7)	4.7 (1.9–7.7)	15.7 (8.3–25.3)	15.6 (8.2–25.1)	12.0 (7.5–16.8)	12.3 (7.8–17.0)	0.9 (<0.1–2.1)	0.9 (<0.1–2.1)	18.1 (13.5–23.4)	18.1 (13.5–23.0)
Armenia	0.5 (0.1–1.1)	0.5 (0.1–1.1)	3.8 (1.5–6.2)	3.8 (1.5–6.2)	16.8 (9.1–27.0)	16.8 (9.1–27.0)	21.6 (14.4–28.2)	21.2 (14.2–28.1)	<0.1 (<0.1–0.2)	<0.1 (<0.1–0.2)	18.4 (8.4–31.6)	18.4 (8.4–31.6)
Azerbaijan	1.2 (0.4–3.0)	1.2 (0.4–3.0)	3.5 (1.4–5.7)	3.5 (1.4–5.7)	12.9 (6.1–22.5)	12.9 (6.1–22.5)	20.1 (13.3–27.4)	20.1 (13.5–27.6)	0.5 (<0.1–1.1)	0.5 (<0.1–1.1)	15.7 (12.6–19.1)	15.7 (12.6–19.1)

	Household air pollution		Handwashing		Ambient particulate matter		Secondhand smoke		High temperature		Low temperature	
	**M**	**F**	**M**	**F**	**M**	**F**	**M**	**F**	**M**	**F**	**M**	**F**
Georgia	5.1 (1.5–11.8)	5.1 (1.5–11.8)	3.3 (1.3–5.4)	3.3 (1.3–5.4)	9.0 (5.3–14.1)	9.0 (5.3–14.1)	21.1 (14.2–28.3)	21.1 (14.2–28.0)	<0.1 (<0.1–0.2)	<0.1 (<0.1–0.2)	18.9 (11.2–29.0)	18.9 (11.2–29.0)
Kazakhstan	1.6 (0.4–4.0)	1.6 (0.4–4.0)	3.4 (1.4–5.7)	3.4 (1.4–5.7)	10.3 (5.4–16.8)	10.3 (5.4–16.8)	15.6 (9.4–21.8)	15.7 (9.6–21.9)	0.5 (<0.1–2.0)	0.5 (<0.1–2.0)	18.4 (11.3–27.6)	18.4 (11.3–27.6)
Kyrgyzstan	6.9 (2.7–13.7)	6.9 (2.7–13.7)	4.0 (1.6–6.5)	4.0 (1.6–6.5)	11.5 (6.2–18.7)	11.5 (6.2–18.7)	19.8 (13.3–26.5)	19.7 (13.1–26.6)	<0.1 (<0.1–<0.1)	<0.1 (<0.1–<0.1)	21.4 (8.4–37.4)	21.4 (8.4–37.4)
Mongolia	8.4 (3.3–17.1)	8.4 (3.3–17.1)	8.5 (3.6–13.6)	8.5 (3.6–13.6)	16.8 (10.7–24.1)	16.8 (10.7–24.1)	14.4 (8.6–20.7)	14.5 (8.4–20.6)	0.2 (<0.1–0.9)	0.2 (<0.1–0.9)	18.3 (3.6–34.9)	18.3 (3.6–34.9)
Tajikistan	9.5 (4.1–18.0)	9.5 (4.1–18.0)	7.1 (2.9–11.3)	7.1 (2.9–11.3)	16.3 (7.9–27.9)	16.3 (7.9–27.9)	11.2 (6.6–16.4)	12.7 (8.1–17.8)	0.3 (0.1–0.5)	0.3 (0.1–0.5)	21.4 (9.3–34.9)	21.4 (9.3–34.9)
Turkmenistan	<0.1 (<0.1–0.1)	<0.1 (<0.1–0.1)	4.6 (1.9–7.4)	4.6 (1.9–7.4)	13.3 (6.3–23.0)	13.3 (6.3–23.0)	19.4 (12.7–26.7)	19.9 (13.3–26.6)	2.6 (0.9–4.3)	2.6 (0.9–4.3)	12.7 (10.0–15.4)	12.7 (10.0–15.4)
Uzbekistan	2.1 (0.7–4.8)	2.1 (0.7–4.8)	4.5 (1.8–7.3)	4.5 (1.8–7.3)	16.8 (8.4–27.7)	16.8 (8.4–27.7)	9.2 (5.2–13.9)	9.4 (5.4–13.7)	1.0 (<0.1–2.7)	1.0 (<0.1–2.7)	18.0 (13.9–22.0)	18.0 (13.9–22.0)
Central Europe	1.8 (0.5–4.2)	1.7 (0.5–4.0)	1.1 (0.5–1.9)	1.1 (0.4–1.9)	9.4 (5.8–14.1)	9.4 (5.8–14.1)	17.0 (10.9–23.7)	17.1 (10.8–23.2)	<0.1 (<0.1–0.1)	<0.1 (<0.1–0.1)	20.8 (15.0–27.7)	20.8 (14.9–27.9)
Albania	4.9 (1.5–11.3)	4.9 (1.5–11.3)	1.7 (0.7–2.9)	1.7 (0.7–2.9)	9.4 (5.8–14.1)	9.4 (5.8–14.1)	19.6 (12.8–26.1)	19.7 (13.0–26.5)	<0.1 (<0.1–0.1)	<0.1 (<0.1–0.1)	19.2 (14.6–24.1)	19.2 (14.6–24.1)
Bosnia and Herzegovina	4.9 (1.6–11.0)	4.9 (1.6–11.0)	1.3 (0.5–2.1)	1.3 (0.5–2.1)	14.4 (9.3–20.3)	14.4 (9.3–20.3)	22.8 (15.3–30.2)	22.6 (14.8–30.2)	<0.1 (<0.1–<0.1)	<0.1 (<0.1–<0.1)	22.3 (15.8–29.9)	22.3 (15.8–29.9)
Bulgaria	2.1 (0.6–5.2)	2.1 (0.6–5.2)	1.1 (0.4–1.8)	1.1 (0.4–1.8)	9.9 (6.2–14.8)	9.9 (6.2–14.8)	21.3 (13.8–28.7)	21.2 (13.8–28.4)	<0.1 (<0.1–<0.1)	<0.1 (<0.1–<0.1)	21.3 (14.8–27.9)	21.3 (14.8–27.9)
Croatia	0.5 (0.1–1.6)	0.5 (0.1–1.6)	1.0 (0.4–1.6)	1.0 (0.4–1.6)	9.4 (5.7–14.2)	9.4 (5.7–14.2)	19.2 (12.5–26.0)	19.0 (12.5–25.6)	<0.1 (<0.1–0.1)	<0.1 (<0.1–0.1)	20.6 (13.3–27.4)	20.6 (13.3–27.4)
Czechia	0.1 (<0.1–0.3)	0.1 (<0.1–0.3)	0.9 (0.3–1.4)	0.9 (0.3–1.4)	8.4 (5.0–13.0)	8.4 (5.0–13.0)	16.6 (10.6–22.7)	16.4 (10.4–22.8)	<0.1 (<0.1–0.2)	<0.1 (<0.1–0.2)	21.1 (13.6–30.0)	21.1 (13.6–30.0)
Hungary	1.7 (0.4–4.8)	1.7 (0.4–4.8)	0.9 (0.4–1.4)	0.9 (0.4–1.4)	8.4 (5.0–12.9)	8.4 (5.0–12.9)	18.1 (11.7–24.8)	18.3 (11.7–25.0)	<0.1 (<0.1–0.2)	<0.1 (<0.1–0.2)	21.4 (11.9–30.6)	21.4 (11.9–30.6)
Montenegro	3.5 (0.9–8.7)	3.5 (0.9–8.7)	1.2 (0.5–2.0)	1.2 (0.5–2.0)	10.9 (6.8–15.8)	10.9 (6.8–15.8)	23.2 (15.7–30.6)	23.1 (15.7–30.6)	<0.1 (<0.1–0.1)	<0.1 (<0.1–0.1)	20.4 (14.1–28.1)	20.4 (14.1–28.1)
North Macedonia	3.2 (0.9–7.4)	3.2 (0.9–7.4)	1.0 (0.4–1.7)	1.0 (0.4–1.7)	15.1 (9.7–21.5)	15.1 (9.7–21.5)	21.9 (14.4–29.4)	22.0 (14.3–29.4)	<0.1 (<0.1–0.1)	<0.1 (<0.1–0.1)	21.8 (15.6–28.1)	21.8 (15.6–28.1)
Poland	1.3 (0.3–3.6)	1.3 (0.3–3.5)	1.0 (0.4–1.6)	1.0 (0.4–1.6)	11.4 (7.2–16.8)	11.5 (7.2–16.9)	15.4 (9.4–22.3)	15.8 (9.8–21.9)	<0.1 (<0.1–0.2)	<0.1 (<0.1–0.2)	21.1 (13.4–30.1)	21.1 (13.4–30.1)
Romania	1.4 (0.3–3.7)	1.4 (0.3–3.7)	1.2 (0.5–2.0)	1.2 (0.5–2.0)	7.9 (4.7–12.1)	7.9 (4.7–12.1)	16.0 (9.6–22.7)	16.2 (9.7–22.7)	<0.1 (<0.1–0.1)	<0.1 (<0.1–0.1)	20.7 (14.2–28.2)	20.7 (14.2–28.2)
Serbia	2.6 (0.7–6.5)	2.6 (0.7–6.5)	1.0 (0.4–1.6)	1.0 (0.4–1.6)	12.9 (8.2–19.1)	12.9 (8.2–19.1)	18.7 (11.9–25.7)	18.8 (11.8–25.5)	<0.1 (<0.1–0.1)	<0.1 (<0.1–0.1)	21.9 (14.8–28.9)	21.9 (14.8–28.9)

	Household air pollution		Handwashing		Ambient particulate matter		Secondhand smoke		High temperature		Low temperature	
	M	F	M	F	M	F	M	F	M	F	M	F
Slovakia	0.1 (<0.1–0.3)	0.1 (<0.1–0.3)	0.9 (0.3–1.4)	0.9 (0.3–1.4)	9.4 (5.5–14.5)	9.4 (5.5–14.5)	14.0 (8.1–19.9)	14.2 (8.4–20.0)	<0.1 (<0.1–0.1)	<0.1 (<0.1–0.1)	20.2 (13.2–29.4)	20.2 (13.2–29.4)
Slovenia	0.7 (0.1–2.0)	0.7 (0.1–2.0)	0.8 (0.3–1.4)	0.8 (0.3–1.4)	8.6 (5.3–13.3)	8.6 (5.3–13.3)	17.4 (11.3–24.2)	17.7 (11.7–23.9)	<0.1 (<0.1–0.1)	<0.1 (<0.1–0.1)	22.1 (15.2–30.1)	22.1 (15.2–30.1)
Eastern Europe	0.3 (0.1–0.8)	0.3 (0.1–0.8)	2.5 (1.0–4.0)	2.5 (1.0–4.0)	5.7 (3.0–9.8)	5.6 (2.9–9.5)	19.3 (12.5–26.0)	19.4 (12.5–26.0)	<0.1 (<0.1–0.3)	<0.1 (<0.1–0.3)	17.4 (3.4–29.6)	17.4 (3.7–29.8)
Belarus	0.1 (<0.1–0.3)	0.1 (<0.1–0.3)	1.9 (0.8–3.1)	1.9 (0.8–3.1)	8.2 (4.7–13.0)	8.2 (4.7–13.0)	20.0 (12.8–27.0)	19.7 (12.9–26.4)	<0.1 (<0.1–0.2)	<0.1 (<0.1–0.2)	18.3 (9.6–30.8)	18.3 (9.6–30.8)
Estonia	0.5 (<0.1–1.8)	0.5 (<0.1–1.8)	1.8 (0.7–3.0)	1.8 (0.7–3.0)	1.8 (0.6–3.6)	1.8 (0.6–3.6)	15.5 (9.9–22.0)	16.2 (10.2–22.3)	0.1 (<0.1–0.5)	0.1 (<0.1–0.5)	15.8 (3.4–32.6)	15.8 (3.4–32.6)
Latvia	0.5 (0.1–1.5)	0.5 (0.1–1.5)	1.9 (0.8–3.1)	1.9 (0.8–3.1)	5.6 (3.1–9.1)	5.6 (3.1–9.1)	16.0 (9.6–22.8)	16.2 (9.6–23.0)	<0.1 (<0.1–0.3)	<0.1 (<0.1–0.3)	17.2 (7.2–31.3)	17.2 (7.2–31.3)
Lithuania	0.2 (<0.1–0.7)	0.2 (<0.1–0.7)	1.9 (0.8–3.2)	1.9 (0.8–3.2)	4.5 (2.3–7.6)	4.5 (2.3–7.6)	14.3 (8.3–20.4)	14.5 (8.7–20.7)	<0.1 (<0.1–0.2)	<0.1 (<0.1–0.2)	19.1 (9.2–31.0)	19.1 (9.2–31.0)
Republic of Moldova	1.0 (0.3–2.2)	1.0 (0.3–2.2)	4.6 (1.9–7.4)	4.6 (1.9–7.4)	6.7 (3.3–11.9)	6.7 (3.3–11.9)	18.3 (12.3–24.7)	18.1 (12.4–24.5)	<0.1 (<0.1–0.1)	<0.1 (<0.1–0.1)	22.1 (13.3–30.8)	22.1 (13.3–30.8)
Russian Federation	0.2 (<0.1–0.6)	0.2 (<0.1–0.6)	2.2 (0.9–3.7)	2.3 (0.9–3.7)	5.2 (2.6–9.1)	5.1 (2.6–8.9)	19.6 (12.8–26.4)	19.9 (13.0–26.8)	<0.1 (<0.1–0.4)	<0.1 (<0.1–0.4)	16.4 (<0.1–31.7)	16.5 (<0.1–31.5)
Ukraine	0.5 (0.1–1.2)	0.5 (0.1–1.2)	3.1 (1.2–5.0)	3.1 (1.2–5.0)	7.1 (3.7–12.4)	7.1 (3.7–12.4)	18.4 (11.7–25.2)	18.1 (11.3–25.0)	<0.1 (<0.1–0.2)	<0.1 (<0.1–0.2)	19.7 (13.3–26.3)	19.7 (13.3–26.3)
**High-income**	**0.1 (<0.1–0.3)**	**0.1 (<0.1–0.3)**	**1.5 (0.6–2.5)**	**1.5 (0.6–2.5)**	**5.2 (2.9–8.6)**	**5.1 (2.9–8.5)**	**13.4 (8.4–18.6)**	**13.7 (8.6–18.8)**	**0.6 (0.3–1.0)**	**0.7 (0.3–1.0)**	**16.5 (12.4–21.3)**	**16.6 (12.4–21.5)**
Australasia	<0.1 (<0.1–<0.1)	<0.1 (<0.1–<0.1)	1.9 (0.8–3.2)	1.9 (0.8–3.2)	1.9 (0.4–4.2)	1.9 (0.4–4.2)	11.3 (6.5–16.2)	11.7 (6.9–16.8)	6.0 (4.1–8.0)	6.4 (4.3–8.5)	8.3 (5.3–11.3)	7.6 (4.5–10.6)
Australia	<0.1 (<0.1–0.1)	<0.1 (<0.1–0.1)	1.9 (0.8–3.2)	1.9 (0.8–3.2)	1.9 (0.5–4.3)	1.9 (0.5–4.3)	11.3 (6.6–16.4)	11.8 (7.0–16.9)	7.3 (4.8–9.7)	7.3 (4.8–9.7)	5.9 (2.7–8.9)	5.9 (2.7–8.9)
New Zealand	<0.1 (<0.1–0.1)	<0.1 (<0.1–0.1)	2.0 (0.8–3.3)	2.0 (0.8–3.3)	1.5 (0.3–3.5)	1.5 (0.3–3.5)	10.9 (6.0–16.1)	11.1 (6.3–16.5)	<0.1 (<0.1–<0.1)	<0.1 (<0.1–<0.1)	20.5 (13.3–27.8)	20.5 (13.3–27.8)
High-income Asia Pacific	<0.1 (<0.1–<0.1)	<0.1 (<0.1–<0.1)	1.9 (0.7–3.1)	1.9 (0.7–3.1)	7.9 (4.5–12.5)	8.0 (4.5–12.7)	16.2 (10.3–22.4)	16.0 (10.1–22.1)	0.5 (<0.1–1.3)	0.5 (<0.1–1.4)	15.3 (12.3–18.7)	15.4 (12.2–18.8)
Brunei Darussalam	0.1 (<0.1–0.2)	0.1 (<0.1–0.2)	2.0 (0.8–3.2)	2.0 (0.8–3.2)	2.6 (0.8–5.4)	2.6 (0.8–5.4)	13.6 (8.3–19.3)	13.8 (8.4–19.6)	0.9 (<0.1–5.8)	0.9 (<0.1–5.8)	<0.1 (<0.1–0.5)	<0.1 (<0.1–0.5)
Japan	<0.1 (<0.1–<0.1)	<0.1 (<0.1–<0.1)	1.9 (0.7–3.1)	1.9 (0.7–3.1)	6.4 (3.5–10.6)	6.4 (3.4–10.5)	16.9 (10.8–23.4)	16.7 (10.5–23.0)	0.5 (<0.1–1.0)	0.5 (<0.1–1.0)	16.0 (12.9–19.3)	16.1 (13.0–19.5)
Republic of Korea	<0.1 (<0.1–<0.1)	<0.1 (<0.1–<0.1)	1.9 (0.7–3.1)	1.9 (0.7–3.1)	14.2 (8.6–21.1)	14.2 (8.6–21.1)	16.3 (9.7–23.1)	16.0 (9.7–22.4)	0.2 (<0.1–0.9)	0.2 (<0.1–0.9)	20.4 (14.7–25.9)	20.4 (14.7–25.9)
Singapore	<0.1 (<0.1–0.1)	<0.1 (<0.1–0.1)	1.9 (0.8–3.1)	1.9 (0.8–3.1)	9.5 (5.0–16.2)	9.5 (5.0–16.2)	10.0 (5.6–14.8)	10.5 (6.0–15.4)	1.3 (<0.1–11.7)	1.3 (<0.1–11.7)	0.1 (<0.1–1.2)	0.1 (<0.1–1.2)

	Household air pollution		Handwashing		Ambient particulate matter		Secondhand smoke		High temperature		Low temperature	
	M	F	M	F	M	F	M	F	M	F	M	F
High-income North America	<0.1 (<0.1–<0.1)	<0.1 (<0.1–<0.1)	1.0 (0.4–1.6)	1.0 (0.4–1.6)	2.6 (1.2–5.0)	2.6 (1.2–5.0)	12.4 (7.9–17.4)	12.6 (8.1–17.3)	0.7 (0.3–1.1)	0.7 (0.3–1.0)	15.3 (11.5–19.4)	15.4 (11.4–19.4)
Canada	<0.1 (<0.1–<0.1)	<0.1 (<0.1–<0.1)	1.0 (0.4–1.7)	1.0 (0.4–1.7)	2.2 (0.9–4.4)	2.2 (0.9–4.4)	11.8 (6.9–17.5)	12.2 (7.7–17.2)	<0.1 (<0.1–0.3)	<0.1 (<0.1–0.3)	20.8 (0.8–39.3)	20.8 (0.8–39.3)
United States of America	<0.1 (<0.1–<0.1)	<0.1 (<0.1–<0.1)	1.0 (0.4–1.6)	1.0 (0.4–1.6)	2.6 (1.2–5.0)	2.7 (1.2–5.0)	12.4 (8.0–17.5)	12.6 (8.1–17.4)	0.7 (0.4–1.1)	0.7 (0.3–1.1)	15.0 (11.5–18.6)	15.0 (11.5–18.6)
Southern Latin America	0.4 (0.1–1.1)	0.4 (0.1–1.1)	2.4 (1.0–4.0)	2.4 (1.0–4.0)	6.9 (3.7–11.5)	6.9 (3.7–11.6)	12.7 (7.5–18.6)	13.5 (7.8–19.2)	0.8 (0.5–1.3)	0.8 (0.5–1.3)	16.2 (10.3–22.7)	16.2 (10.3–22.8)
Argentina	0.4 (0.1–1.1)	0.4 (0.1–1.1)	2.5 (1.0–4.1)	2.5 (1.0–4.1)	6.5 (3.4–11.1)	6.5 (3.4–11.1)	12.7 (7.4–18.6)	13.6 (7.9–19.4)	0.9 (0.5–1.4)	0.9 (0.5–1.4)	16.1 (10.5–22.4)	16.1 (10.5–22.4)
Chile	0.7 (0.2–1.9)	0.7 (0.2–1.9)	2.0 (0.8–3.4)	2.0 (0.8–3.4)	11.7 (7.1–18.0)	11.7 (7.1–18.0)	11.7 (6.2–17.6)	12.1 (6.4–18.5)	<0.1 (<0.1–<0.1)	<0.1 (<0.1–<0.1)	19.3 (10.4–29.4)	19.3 (10.4–29.4)
Uruguay	0.3 (0.1–0.9)	0.3 (0.1–0.9)	2.3 (0.9–3.7)	2.3 (0.9–3.7)	4.0 (1.7–7.6)	4.0 (1.7–7.6)	14.7 (8.6–20.6)	15.6 (9.5–21.9)	0.1 (<0.1–0.3)	0.1 (<0.1–0.3)	8.0 (4.5–11.6)	8.0 (4.5–11.6)
Western Europe	<0.1 (<0.1–0.1)	<0.1 (<0.1–0.1)	1.0 (0.4–1.7)	1.0 (0.4–1.7)	5.3 (3.0–8.7)	5.3 (3.0–8.6)	13.8 (8.4–19.2)	14.0 (8.6–19.6)	0.2 (<0.1–0.3)	0.2 (<0.1–0.3)	20.1 (14.4–26.3)	20.2 (14.5–26.3)
Andorra	<0.1 (<0.1–<0.1)	<0.1 (<0.1–<0.1)	1.0 (0.4–1.6)	1.0 (0.4–1.6)	3.5 (1.7–6.3)	3.5 (1.7–6.3)	14.7 (8.8–20.6)	14.8 (9.3–20.9)	<0.1 (<0.1–<0.1)	<0.1 (<0.1–<0.1)	17.0 (1.9–35.8)	17.0 (1.9–35.8)
Austria	<0.1 (<0.1–0.1)	<0.1 (<0.1–0.1)	1.0 (0.4–1.6)	1.0 (0.4–1.6)	5.6 (3.1–9.1)	5.6 (3.1–9.1)	19.0 (12.8–25.7)	19.0 (12.7–25.8)	<0.1 (<0.1–0.1)	<0.1 (<0.1–0.1)	19.7 (10.6–31.5)	19.7 (10.6–31.5)
Belgium	<0.1 (<0.1–<0.1)	<0.1 (<0.1–<0.1)	1.0 (0.4–1.6)	1.0 (0.4–1.6)	5.9 (3.4–9.7)	5.9 (3.4–9.7)	14.6 (9.2–20.3)	15.3 (9.8–21.4)	<0.1 (<0.1–0.1)	<0.1 (<0.1–0.1)	23.4 (14.7–32.7)	23.4 (14.7–32.7)
Cyprus	<0.1 (<0.1–<0.1)	<0.1 (<0.1–<0.1)	1.0 (0.4–1.6)	1.0 (0.4–1.6)	7.7 (4.5–12.5)	7.7 (4.5–12.5)	17.9 (11.5–24.5)	18.0 (11.5–24.9)	2.1 (0.9–3.6)	2.1 (0.9–3.6)	4.5 (<0.1–10.9)	4.5 (<0.1–10.9)
Denmark	<0.1 (<0.1–<0.1)	<0.1 (<0.1–<0.1)	1.0 (0.4–1.6)	1.0 (0.4–1.6)	4.0 (2.1–7.2)	4.0 (2.1–7.2)	14.5 (8.9–20.2)	14.8 (9.1–20.6)	<0.1 (<0.1–<0.1)	<0.1 (<0.1–<0.1)	23.2 (13.0–34.4)	23.2 (13.0–34.4)
Finland	<0.1 (<0.1–<0.1)	<0.1 (<0.1–<0.1)	1.0 (0.4–1.7)	1.0 (0.4–1.7)	1.2 (0.2–2.9)	1.2 (0.2–2.9)	12.1 (7.2–17.1)	11.7 (7.2–16.6)	<0.1 (<0.1–0.3)	<0.1 (<0.1–0.3)	17.9 (3.8–36.4)	17.9 (3.8–36.4)
France	<0.1 (<0.1–<0.1)	<0.1 (<0.1–<0.1)	1.0 (0.4–1.7)	1.0 (0.4–1.7)	5.1 (2.8–8.5)	5.1 (2.8–8.5)	12.8 (7.2–18.3)	12.9 (7.4–18.8)	<0.1 (<0.1–0.1)	<0.1 (<0.1–0.1)	21.7 (14.9–28.4)	21.7 (14.9–28.4)
Germany	<0.1 (<0.1–<0.1)	<0.1 (<0.1–<0.1)	1.0 (0.4–1.6)	1.0 (0.4–1.6)	5.3 (3.0–8.8)	5.3 (3.0–8.8)	14.5 (8.8–20.3)	14.6 (9.0–20.3)	<0.1 (<0.1–0.2)	<0.1 (<0.1–0.2)	22.5 (14.3–31.1)	22.5 (14.3–31.1)
Greece	0.1 (<0.1–0.2)	0.1 (<0.1–0.2)	1.0 (0.4–1.7)	1.0 (0.4–1.7)	6.9 (4.0–10.9)	6.9 (4.0–10.9)	19.7 (12.4–27.1)	19.7 (12.7–26.8)	0.1 (<0.1–0.3)	0.1 (<0.1–0.3)	15.7 (12.3–19.4)	15.7 (12.3–19.4)
Iceland	<0.1 (<0.1–<0.1)	<0.1 (<0.1–<0.1)	1.0 (0.4–1.7)	1.0 (0.4–1.7)	1.2 (0.2–3.0)	1.2 (0.2–3.0)	16.0 (10.2–22.2)	16.8 (11.3–22.9)	<0.1 (0.0–0.0)	<0.1 (0.0–0.0)	21.5 (5.1–38.7)	21.5 (5.1–38.7)
Ireland	<0.1 (<0.1–<0.1)	<0.1 (<0.1–<0.1)	1.0 (0.4–1.6)	1.0 (0.4–1.6)	2.7 (1.2–5.1)	2.7 (1.2–5.1)	11.8 (6.9–16.9)	12.0 (7.0–16.8)	<0.1 (0.0–0.0)	<0.1 (0.0–0.0)	25.7 (14.4–37.2)	25.7 (14.4–37.2)

	Household air pollution		Handwashing		Ambient particulate matter		Secondhand smoke		High temperature		Low temperature	
	**M**	**F**	**M**	**F**	**M**	**F**	**M**	**F**	**M**	**F**	**M**	**F**
Israel	<0.1 (<0.1–0.1)	<0.1 (<0.1–0.1)	1.0 (0.4–1.7)	1.0 (0.4–1.7)	10.1 (6.1–15.3)	10.1 (6.1–15.3)	12.7 (7.3–18.6)	13.3 (7.9–19.0)	2.1 (0.8–3.9)	2.1 (0.8–3.9)	3.5 (<0.1–10.4)	3.5 (<0.1–10.4)
Italy	<0.1 (<0.1–0.1)	<0.1 (<0.1–0.1)	1.0 (0.4–1.7)	1.0 (0.4–1.7)	8.0 (4.8–12.4)	7.9 (4.7–12.2)	14.1 (8.8–19.5)	14.8 (9.4–20.8)	0.1 (<0.1–0.2)	0.1 (<0.1–0.2)	17.5 (13.1–21.9)	17.4 (13.0–21.7)
Luxembourg	<0.1 (<0.1–<0.1)	<0.1 (<0.1–<0.1)	1.0 (0.4–1.6)	1.0 (0.4–1.6)	4.2 (2.2–7.3)	4.2 (2.2–7.3)	14.9 (9.3–21.3)	15.1 (9.5–20.9)	<0.1 (<0.1–0.1)	<0.1 (<0.1–0.1)	23.1 (13.3–33.6)	23.1 (13.3–33.6)
Malta	<0.1 (<0.1–<0.1)	<0.1 (<0.1–<0.1)	1.0 (0.4–1.7)	1.0 (0.4–1.7)	6.1 (3.4–9.9)	6.1 (3.4–9.9)	15.7 (9.9–22.0)	16.2 (10.7–22.0)	0.3 (<0.1–0.9)	0.3 (<0.1–0.9)	3.6 (<0.1–15.0)	3.6 (<0.1–15.0)
Monaco	<0.1 (<0.1–<0.1)	<0.1 (<0.1–<0.1)	1.0 (0.4–1.6)	1.0 (0.4–1.6)	5.3 (2.5–9.2)	5.3 (2.5–9.2)	14.9 (9.3–20.9)	15.2 (9.4–21.2)	<0.1 (<0.1–0.1)	<0.1 (<0.1–0.1)	15.4 (11.9–19.0)	15.4 (11.9–19.0)
Netherlands	<0.1 (<0.1–<0.1)	<0.1 (<0.1–<0.1)	1.0 (0.4–1.6)	1.0 (0.4–1.6)	5.5 (3.1–9.0)	5.5 (3.1–9.0)	12.2 (7.1–17.6)	12.6 (7.6–18.0)	<0.1 (<0.1–0.1)	<0.1 (<0.1–0.1)	23.6 (13.4–33.5)	23.6 (13.4–33.5)
Norway	<0.1 (<0.1–<0.1)	<0.1 (<0.1–<0.1)	1.0 (0.4–1.6)	1.0 (0.4–1.6)	1.8 (0.6–3.9)	1.8 (0.6–3.9)	12.0 (7.0–17.5)	12.6 (7.5–18.1)	<0.1 (<0.1–0.1)	<0.1 (<0.1–0.1)	19.0 (5.9–35.8)	19.0 (5.8–35.8)
Portugal	0.1 (<0.1–0.2)	0.1 (<0.1–0.2)	1.1 (0.4–1.8)	1.1 (0.4–1.8)	3.0 (1.4–5.5)	3.0 (1.4–5.5)	12.1 (7.0–17.2)	12.3 (7.0–17.8)	0.2 (<0.1–0.3)	0.2 (<0.1–0.3)	15.3 (11.7–19.2)	15.3 (11.7–19.2)
San Marino	<0.1 (<0.1–<0.1)	<0.1 (<0.1–<0.1)	1.0 (0.4–1.7)	1.0 (0.4–1.7)	4.1 (1.5–7.9)	4.1 (1.5–7.9)	16.1 (10.0–22.5)	16.5 (10.6–22.5)	0.1 (<0.1–0.2)	0.1 (<0.1–0.2)	15.7 (12.5–19.0)	15.7 (12.5–19.0)
Spain	0.1 (<0.1–0.2)	0.1 (<0.1–0.2)	1.0 (0.4–1.7)	1.0 (0.4–1.7)	4.0 (2.1–6.9)	4.0 (2.1–6.9)	15.5 (9.3–21.7)	15.4 (9.6–21.8)	0.2 (<0.1–0.5)	0.2 (<0.1–0.5)	17.3 (13.3–21.5)	17.3 (13.3–21.5)
Sweden	<0.1 (<0.1–<0.1)	<0.1 (<0.1–<0.1)	1.0 (0.4–1.7)	1.0 (0.4–1.7)	1.2 (0.3–3.0)	1.2 (0.3–3.0)	11.4 (6.9–16.5)	10.9 (6.7–15.7)	<0.1 (<0.1–0.1)	<0.1 (<0.1–0.1)	18.5 (5.5–33.0)	18.5 (5.9–32.8)
Switzerland	<0.1 (<0.1–<0.1)	<0.1 (<0.1–<0.1)	1.0 (0.4–1.6)	1.0 (0.4–1.6)	4.1 (2.2–7.0)	4.1 (2.2–7.0)	13.0 (7.7–18.5)	13.2 (8.0–19.0)	<0.1 (<0.1–<0.1)	<0.1 (<0.1–<0.1)	21.1 (11.3–32.9)	21.1 (11.3–32.9)
United Kingdom	<0.1 (<0.1–<0.1)	<0.1 (<0.1–<0.1)	1.0 (0.4–1.7)	1.0 (0.4–1.7)	4.3 (2.3–7.4)	4.3 (2.3–7.4)	12.7 (7.3–18.5)	12.9 (7.6–18.4)	<0.1 (<0.1–<0.1)	<0.1 (<0.1–<0.1)	24.8 (15.7–34.9)	24.9 (15.8–34.8)
**Latin America and Caribbean**	**8.4 (4.3–13.9)**	**9.7 (5.0–15.8)**	**8.2 (3.4–12.8)**	**8.6 (3.7–13.5)**	**9.2 (6.1–13.3)**	**9.0 (6.0–13.1)**	**5.6 (3.0–8.6)**	**5.7 (3.0–8.8)**	**1.3 (<0.1–4.3)**	**1.3 (<0.1–4.4)**	**3.2 (1.5–4.9)**	**3.1 (1.5–4.8)**
Andean Latin America	4.4 (1.8–8.8)	4.5 (1.8–9.1)	7.8 (3.2–12.2)	7.8 (3.2–12.3)	13.1 (8.1–19.7)	13.2 (8.2–19.9)	2.8 (1.0–5.3)	2.8 (1.1–5.3)	0.6 (<0.1–1.5)	0.6 (<0.1–1.5)	8.9 (5.5–12.5)	9.0 (5.7–12.6)
Bolivia (Plurinational State of)	7.7 (3.1–14.6)	7.7 (3.1–14.6)	8.4 (3.5–13.2)	8.4 (3.5–13.2)	12.5 (7.4–19.5)	12.5 (7.4–19.5)	2.7 (0.9–5.2)	2.8 (1.0–5.3)	1.5 (0.5–2.7)	1.5 (0.5–2.7)	9.1 (5.9–12.6)	9.1 (5.9–12.6)
Ecuador	1.2 (0.4–3.0)	1.2 (0.4–3.0)	6.9 (2.8–11.0)	6.9 (2.8–11.0)	10.2 (5.4–16.3)	10.2 (5.4–16.3)	2.6 (0.9–5.1)	2.7 (1.0–5.2)	0.3 (<0.1–1.2)	0.3 (<0.1–1.2)	5.8 (3.6–8.1)	5.8 (3.6–8.1)
Peru	4.3 (1.6–8.9)	4.3 (1.6–8.9)	7.9 (3.3–12.5)	7.9 (3.3–12.5)	14.9 (9.3–22.4)	14.9 (9.3–22.4)	2.9 (1.1–5.4)	2.9 (1.1–5.5)	0.2 (<0.1–0.9)	0.2 (<0.1–0.9)	10.4 (6.0–15.2)	10.4 (6.0–15.2)
Caribbean	26.8 (15.8–38.6)	30.3 (18.3–43.2)	17.8 (8.0–26.7)	18.2 (8.2–27.1)	6.6 (3.0–12.7)	6.2 (2.8–11.8)	4.3 (2.2–6.7)	4.0 (2.0–6.4)	0.8 (<0.1–2.7)	0.8 (<0.1–2.8)	<0.1 (<0.1–1.3)	<0.1 (<0.1–1.3)

	Household air pollution		Handwashing		Ambient particulate matter		Secondhand smoke		High temperature		Low temperature	
	**M**	**F**	**M**	**F**	**M**	**F**	**M**	**F**	**M**	**F**	**M**	**F**
Antigua and Barbuda	0.1 (<0.1–0.4)	0.1 (<0.1–0.4)	7.5 (3.1–11.9)	7.5 (3.1–11.9)	8.7 (2.7–18.4)	8.7 (2.7–18.4)	6.4 (3.3–9.7)	6.6 (3.7–10.1)	0.4 (<0.1–2.3)	0.4 (<0.1–2.3)	0.3 (<0.1–1.7)	0.3 (<0.1–1.7)
Bahamas	0.2 (<0.1–0.5)	0.2 (<0.1–0.5)	6.3 (2.6–10.2)	6.3 (2.6–10.2)	7.6 (1.7–16.4)	7.6 (1.7–16.4)	7.6 (4.3–11.8)	7.7 (4.4–11.3)	1.6 (<0.1–4.5)	1.6 (<0.1–4.5)	0.3 (<0.1–2.0)	0.3 (<0.1–2.0)
Barbados	<0.1 (<0.1–<0.1)	<0.1 (<0.1–<0.1)	7.1 (2.9–11.4)	7.1 (2.9–11.4)	10.8 (3.8–21.4)	10.8 (3.8–21.4)	5.1 (2.7–7.8)	5.2 (2.9–8.0)	1.2 (<0.1–3.9)	1.2 (<0.1–3.9)	0.4 (<0.1–2.0)	0.4 (<0.1–2.0)
Belize	3.6 (1.3–7.6)	3.6 (1.3–7.6)	9.5 (4.0–15.0)	9.5 (4.0–15.0)	10.5 (3.4–20.6)	10.5 (3.4–20.6)	7.4 (4.0–11.1)	7.3 (4.0–10.9)	1.7 (<0.1–4.2)	1.7 (<0.1–4.2)	0.1 (<0.1–1.3)	0.1 (<0.1–1.3)
Cuba	0.3 (0.1–0.9)	0.3 (0.1–0.9)	6.0 (2.5–9.6)	6.0 (2.5–9.6)	8.8 (3.7–16.9)	8.8 (3.7–16.9)	10.5 (6.0–15.6)	10.0 (5.9–14.5)	1.5 (<0.1–4.0)	1.5 (<0.1–4.0)	0.2 (<0.1–1.6)	0.2 (<0.1–1.6)
Dominica	1.3 (0.3–3.2)	1.3 (0.3–3.2)	8.3 (3.5–13.2)	8.3 (3.5–13.2)	9.4 (3.3–18.9)	9.4 (3.3–18.9)	5.6 (2.8–9.0)	5.7 (3.0–8.7)	1.1 (<0.1–3.5)	1.1 (<0.1–3.5)	0.3 (<0.1–3.2)	0.3 (<0.1–3.2)
Dominican Republic	2.4 (0.8–5.5)	2.4 (0.8–5.5)	16.0 (7.1–24.3)	16.0 (7.1–24.3)	8.8 (3.4–17.8)	8.8 (3.4–17.8)	6.0 (3.4–8.9)	6.2 (3.6–9.1)	0.6 (<0.1–2.2)	0.6 (<0.1–2.2)	<0.1 (<0.1–1.7)	<0.1 (<0.1–1.7)
Grenada	0.5 (0.1–1.2)	0.5 (0.1–1.2)	7.6 (3.2–12.2)	7.6 (3.2–12.2)	10.8 (3.4–21.4)	10.8 (3.4–21.4)	5.5 (2.6–9.1)	5.4 (2.6–8.8)	1.4 (<0.1–4.2)	1.4 (<0.1–4.2)	0.3 (<0.1–1.8)	0.3 (<0.1–1.8)
Guyana	1.4 (0.5–3.2)	1.4 (0.5–3.2)	14.1 (6.1–21.9)	14.1 (6.1–21.9)	10.0 (3.2–20.4)	10.0 (3.2–20.4)	7.4 (3.8–11.2)	7.3 (3.8–11.2)	0.9 (<0.1–2.8)	0.9 (<0.1–2.8)	<0.1 (<0.1–1.3)	<0.1 (<0.1–1.3)
Haiti	38.3 (23.2–52.8)	38.3 (23.2–52.8)	19.5 (8.7–29.2)	19.5 (8.7–29.2)	5.5 (2.2–11.4)	5.5 (2.2–11.4)	3.2 (1.4–5.4)	3.2 (1.4–5.4)	0.8 (<0.1–3.0)	0.8 (<0.1–3.0)	<0.1 (<0.1–1.3)	<0.1 (<0.1–1.3)
Jamaica	1.7 (0.5–4.2)	1.7 (0.5–4.2)	11.2 (4.8–17.4)	11.2 (4.8–17.4)	7.7 (4.0–13.1)	7.7 (4.0–13.1)	8.9 (5.2–13.0)	8.8 (5.1–12.9)	1.2 (<0.1–4.3)	1.2 (<0.1–4.3)	0.1 (<0.1–1.1)	0.1 (<0.1–1.1)
Saint Kitts and Nevis	0.3 (0.1–0.9)	0.3 (0.1–0.9)	7.6 (3.2–12.1)	7.6 (3.2–12.1)	3.5 (1.3–6.7)	3.5 (1.3–6.7)	7.1 (3.7–11.2)	7.1 (3.8–10.9)	1.0 (<0.1–3.9)	1.0 (<0.1–3.9)	0.4 (<0.1–2.3)	0.4 (<0.1–2.3)
Saint Lucia	0.6 (0.2–1.5)	0.6 (0.2–1.5)	5.7 (2.3–9.2)	5.7 (2.3–9.2)	10.7 (3.7–21.0)	10.7 (3.7–21.0)	5.9 (3.1–8.9)	5.7 (3.0–8.8)	1.7 (<0.1–4.8)	1.7 (<0.1–4.8)	0.3 (<0.1–1.9)	0.3 (<0.1–1.9)
Saint Vincent and the Grenadines	0.8 (0.2–1.9)	0.8 (0.2–1.9)	9.1 (3.8–14.3)	9.1 (3.8–14.3)	10.6 (3.3–21.1)	10.6 (3.3–21.1)	6.5 (3.2–10.0)	6.4 (3.3–9.9)	1.3 (<0.1–4.0)	1.3 (<0.1–4.0)	0.4 (<0.1–2.0)	0.4 (<0.1–2.0)
Suriname	2.0 (0.6–4.6)	2.0 (0.6–4.6)	9.3 (3.9–14.9)	9.3 (3.9–14.9)	10.6 (4.2–20.5)	10.6 (4.2–20.5)	11.7 (6.6–17.1)	11.7 (6.6–17.0)	1.0 (<0.1–3.0)	1.0 (<0.1–3.0)	0.3 (<0.1–1.6)	0.3 (<0.1–1.6)
Trinidad and Tobago	<0.1 (<0.1–<0.1)	<0.1 (<0.1–<0.1)	6.8 (2.8–10.9)	6.8 (2.8–10.9)	11.0 (3.1–22.8)	11.0 (3.1–22.8)	8.6 (4.5–13.2)	8.4 (4.5–12.8)	0.4 (<0.1–2.6)	0.4 (<0.1–2.6)	0.2 (<0.1–1.2)	0.2 (<0.1–1.2)
Central Latin America	9.0 (4.3–15.6)	8.9 (4.3–15.4)	6.4 (2.7–10.3)	6.5 (2.7–10.3)	10.6 (7.0–15.3)	10.7 (7.0–15.5)	4.0 (1.7–6.9)	4.0 (1.6–7.0)	1.3 (0.1–3.3)	1.3 (0.1–3.2)	2.8 (1.0–4.7)	3.1 (1.3–4.9)
Colombia	2.2 (0.7–5.0)	2.2 (0.7–5.0)	6.1 (2.5–9.9)	6.1 (2.5–9.9)	11.2 (6.5–17.4)	11.2 (6.5–17.4)	4.9 (2.1–8.0)	5.1 (2.4–8.3)	1.0 (<0.1–3.4)	1.0 (<0.1–3.4)	3.4 (1.8–5.1)	3.4 (1.8–5.1)
Costa Rica	1.1 (0.3–2.5)	1.1 (0.3–2.5)	6.8 (2.8–10.9)	6.8 (2.8–10.9)	8.8 (5.2–13.7)	8.8 (5.2–13.7)	8.0 (4.5–11.7)	8.0 (4.4–11.7)	0.3 (<0.1–2.2)	0.3 (<0.1–2.2)	0.6 (<0.1–2.7)	0.6 (<0.1–2.7)

	Household air pollution		Handwashing		Ambient particulate matter		Secondhand smoke		High temperature		Low temperature	
	M	F	M	F	M	F	M	F	M	F	M	F
El Salvador	3.8 (1.5–7.7)	3.8 (1.5–7.7)	9.0 (3.8–14.4)	9.0 (3.8–14.4)	11.1 (5.9–18.0)	11.1 (5.9–18.0)	3.6 (1.7–5.8)	3.6 (1.8–5.9)	1.4 (<0.1–6.7)	1.4 (<0.1–6.7)	<0.1 (<0.1–0.6)	<0.1 (<0.1–0.6)
Guatemala	17.1 (8.3–29.5)	17.1 (8.3–29.5)	7.6 (3.2–12.1)	7.6 (3.2–12.1)	11.3 (6.9–17.1)	11.3 (6.9–17.1)	3.2 (1.2–5.8)	3.3 (1.3–5.8)	1.7 (0.5–3.5)	1.7 (0.5–3.5)	1.5 (<0.1–3.2)	1.5 (<0.1–3.2)
Honduras	21.0 (11.0–33.9)	21.0 (11.0–33.9)	5.4 (2.2–8.6)	5.4 (2.2–8.6)	8.9 (5.3–13.6)	8.9 (5.3–13.6)	6.7 (3.4–10.3)	6.8 (3.6–10.4)	0.7 (<0.1–2.6)	0.7 (<0.1–2.6)	<0.1 (<0.1–2.4)	<0.1 (<0.1–2.4)
Mexico	4.9 (2.1–8.8)	4.9 (2.1–8.9)	5.2 (2.1–8.4)	5.2 (2.1–8.4)	9.9 (6.3–14.8)	10.0 (6.3–14.8)	3.1 (0.8–6.6)	3.2 (0.9–6.5)	1.1 (0.1–2.2)	1.1 (0.1–2.1)	6.2 (3.6–9.1)	6.4 (3.9–9.2)
Nicaragua	16.5 (7.7–28.1)	16.5 (7.7–28.1)	8.1 (3.4–12.8)	8.1 (3.4–12.8)	8.7 (5.0–14.1)	8.7 (5.0–14.1)	5.6 (2.6–9.2)	5.7 (2.7–9.5)	0.8 (<0.1–4.9)	0.8 (<0.1–4.9)	<0.1 (<0.1–1.1)	<0.1 (<0.1–1.1)
Panama	1.7 (0.5–3.8)	1.7 (0.5–3.8)	6.1 (2.5–9.8)	6.1 (2.5–9.8)	6.5 (3.3–10.9)	6.5 (3.3–10.9)	4.2 (2.2–6.3)	4.4 (2.4–6.5)	0.6 (<0.1–2.3)	0.6 (<0.1–2.3)	<0.1 (<0.1–1.0)	<0.1 (<0.1–1.0)
Venezuela (Bolivarian Republic of)	0.1 (<0.1–0.2)	0.1 (<0.1–0.2)	5.7 (2.4–9.2)	5.7 (2.4–9.2)	11.1 (5.9–18.6)	11.1 (5.9–18.6)	6.2 (2.7–10.3)	6.4 (2.9–10.8)	1.2 (<0.1–9.4)	1.2 (<0.1–9.4)	0.1 (<0.1–1.7)	0.1 (<0.1–1.7)
Tropical Latin America	2.7 (0.9–6.0)	2.9 (1.0–6.4)	6.6 (2.7–10.7)	6.7 (2.8–10.8)	5.4 (3.1–8.8)	5.4 (3.1–8.6)	10.4 (6.2–15.1)	10.8 (6.4–15.7)	2.0 (<0.1–9.4)	2.0 (<0.1–9.7)	0.8 (<0.1–2.3)	0.8 (<0.1–2.3)
Brazil	2.5 (0.8–5.6)	2.7 (0.9–6.0)	6.6 (2.7–10.6)	6.7 (2.7–10.7)	5.4 (3.1–8.7)	5.3 (3.1–8.6)	10.4 (6.2–15.3)	10.9 (6.4–15.8)	1.9 (<0.1–9.5)	1.9 (<0.1–10.0)	0.8 (<0.1–2.3)	0.8 (<0.1–2.2)
Paraguay	7.9 (2.9–15.8)	7.9 (2.9–15.8)	7.9 (3.3–12.7)	7.9 (3.3–12.7)	6.2 (3.6–10.5)	6.2 (3.6–10.5)	8.9 (5.0–13.1)	8.8 (4.9–13.0)	5.2 (2.9–7.6)	5.2 (2.9–7.6)	0.9 (<0.1–5.0)	0.9 (<0.1–5.0)
**North Africa and Middle East**	**11.0 (6.3–16.5)**	**11.8 (6.5–18.0)**	**8.9 (4.0–13.8)**	**9.4 (4.0–14.6)**	**20.0 (14.0–26.9)**	**19.2 (13.5–25.9)**	**14.2 (9.0–19.5)**	**14.0 (9.0–19.1)**	**5.9 (3.7–8.2)**	**5.8 (3.7–8.3)**	**6.6 (2.7–11.0)**	**6.8 (2.8–11.0)**
Afghanistan	35.9 (22.5–48.0)	35.9 (22.5–48.0)	15.7 (6.8–23.8)	15.7 (6.8–23.8)	10.9 (4.6–19.4)	10.9 (4.6–19.4)	10.1 (5.8–14.7)	10.1 (5.9–14.5)	3.1 (1.9–4.2)	3.1 (1.9–4.2)	13.7 (9.0–19.6)	13.7 (9.0–19.6)
Algeria	0.1 (<0.1–0.1)	0.1 (<0.1–0.1)	4.9 (2.0–7.9)	4.9 (2.0–7.9)	16.6 (9.1–25.5)	16.6 (9.1–25.5)	16.6 (10.6–22.5)	16.8 (10.8–23.0)	9.2 (6.2–12.2)	9.2 (6.2–12.2)	4.3 (<0.1–10.1)	4.3 (<0.1–10.1)
Bahrain	0.1 (<0.1–0.2)	0.1 (<0.1–0.2)	4.6 (1.9–7.4)	4.6 (1.9–7.4)	26.5 (17.9–36.4)	26.5 (17.9–36.4)	12.3 (7.5–17.7)	12.9 (7.9–18.6)	11.6 (5.8–16.6)	11.6 (5.8–16.6)	2.0 (<0.1–10.1)	2.0 (<0.1–10.1)
Egypt	<0.1 (<0.1–0.1)	<0.1 (<0.1–0.1)	3.8 (1.5–6.3)	3.8 (1.5–6.3)	28.9 (19.3–40.0)	28.9 (19.3–40.0)	16.3 (10.3–22.3)	16.3 (10.3–22.3)	7.7 (4.8–10.9)	7.7 (4.8–10.9)	2.7 (<0.1–9.1)	2.7 (<0.1–9.1)
Iran (Islamic Republic of)	0.1 (<0.1–0.2)	0.1 (<0.1–0.2)	4.3 (1.7–7.1)	4.3 (1.7–7.1)	19.3 (12.7–27.1)	19.6 (12.9–27.6)	13.3 (8.1–19.1)	13.8 (8.7–19.2)	3.7 (2.6–5.0)	4.1 (2.8–5.4)	12.3 (8.9–15.5)	11.9 (8.3–15.1)
Iraq	0.1 (<0.1–0.1)	0.1 (<0.1–0.1)	2.7 (1.1–4.5)	2.7 (1.1–4.5)	22.7 (14.6–32.4)	22.7 (14.6–32.4)	16.1 (10.4–22.2)	15.8 (10.0–21.7)	10.8 (7.5–13.8)	10.8 (7.5–13.8)	4.7 (<0.1–10.6)	4.7 (<0.1–10.6)
Jordan	<0.1 (<0.1–<0.1)	<0.1 (<0.1–<0.1)	4.2 (1.7–6.9)	4.2 (1.7–6.9)	15.7 (9.8–23.4)	15.7 (9.8–23.4)	16.6 (10.3–22.8)	17.1 (10.7–23.3)	3.2 (1.7–4.9)	3.2 (1.7–4.9)	6.0 (<0.1–12.1)	6.0 (<0.1–12.1)
Kuwait	<0.1 (<0.1–<0.1)	<0.1 (<0.1–<0.1)	3.7 (1.5–6.0)	3.7 (1.5–6.0)	27.1 (18.5–36.9)	27.1 (18.5–36.9)	18.6 (12.1–25.3)	18.4 (12.4–25.1)	13.1 (7.6–18.0)	13.1 (7.6–18.0)	3.1 (<0.1–15.6)	3.1 (<0.1–15.6)

	Household air pollution		Handwashing		Ambient particulate matter		Secondhand smoke		High temperature		Low temperature	
	M	F	M	F	M	F	M	F	M	F	M	F
Lebanon	<0.1 (<0.1–0.1)	<0.1 (<0.1–0.1)	5.0 (2.1–8.0)	5.0 (2.1–8.0)	14.9 (9.0–22.6)	14.9 (9.0–22.6)	20.3 (13.7–27.7)	20.3 (13.7–27.4)	0.1 (<0.1–0.2)	0.1 (<0.1–0.2)	14.0 (10.4–17.5)	14.0 (10.4–17.5)
Libya	<0.1 (<0.1–0.1)	<0.1 (<0.1–0.1)	4.5 (1.9–7.4)	4.5 (1.9–7.4)	19.0 (11.0–29.3)	19.0 (11.0–29.3)	18.1 (11.7–24.7)	17.9 (11.7–24.2)	8.2 (4.7–12.0)	8.2 (4.7–12.0)	2.8 (<0.1–9.8)	2.8 (<0.1–9.8)
Morocco	1.2 (0.4–2.5)	1.2 (0.4–2.5)	7.6 (3.2–12.2)	7.6 (3.2–12.2)	17.3 (10.8–24.8)	17.3 (10.8–24.8)	10.4 (6.1–15.1)	10.6 (6.3–15.0)	2.5 (1.6–3.4)	2.5 (1.6–3.4)	8.2 (4.8–11.8)	8.2 (4.8–11.8)
Oman	0.1 (<0.1–0.2)	0.1 (<0.1–0.2)	5.0 (2.0–8.2)	5.0 (2.0–8.2)	21.4 (12.7–31.3)	21.4 (12.7–31.3)	11.0 (6.5–15.8)	11.5 (6.9–16.8)	6.6 (<0.1–22.3)	6.6 (<0.1–22.3)	1.3 (<0.1–13.3)	1.3 (<0.1–13.3)
Palestine	0.3 (0.1–0.6)	0.3 (0.1–0.6)	7.1 (3.0–11.4)	7.1 (3.0–11.4)	15.9 (9.5–24.7)	15.9 (9.5–24.7)	17.0 (11.1–23.0)	17.1 (10.9–22.9)	2.2 (0.9–4.0)	2.2 (0.9–4.0)	3.9 (<0.1–10.5)	3.9 (<0.1–10.5)
Qatar	<0.1 (<0.1–<0.1)	<0.1 (<0.1–<0.1)	3.8 (1.5–6.3)	3.8 (1.5–6.3)	31.1 (22.0–41.1)	31.1 (22.0–41.1)	14.7 (9.1–20.7)	15.2 (9.5–21.3)	10.6 (3.7–19.5)	10.6 (3.7–19.5)	1.8 (<0.1–15.2)	1.8 (<0.1–15.2)
Saudi Arabia	0.1 (<0.1–0.2)	0.1 (<0.1–0.2)	4.2 (1.7–6.7)	4.2 (1.7–6.7)	27.0 (18.4–37.3)	27.0 (18.4–37.3)	16.3 (10.5–22.4)	16.6 (10.9–22.3)	9.7 (5.9–14.1)	9.7 (5.9–14.1)	2.4 (<0.1–8.8)	2.4 (<0.1–8.8)
Sudan	14.4 (7.1–24.8)	14.4 (7.1–24.8)	17.7 (7.9–26.7)	17.7 (7.9–26.7)	19.8 (11.5–30.1)	19.8 (11.5–30.1)	12.8 (8.5–17.7)	12.5 (8.3–17.2)	8.3 (3.6–15.7)	8.3 (3.6–15.7)	1.5 (<0.1–7.2)	1.5 (<0.1–7.2)
Syrian Arab Republic	<0.1 (<0.1–0.1)	<0.1 (<0.1–0.1)	4.9 (2.0–7.9)	4.9 (2.0–7.9)	15.8 (9.6–23.6)	15.8 (9.6–23.6)	15.3 (9.4–21.5)	15.3 (9.7–20.8)	4.4 (2.5–6.3)	4.4 (2.5–6.3)	7.7 (2.5–12.6)	7.7 (2.5–12.6)
Tunisia	<0.1 (<0.1–0.1)	<0.1 (<0.1–0.1)	5.7 (2.4–9.2)	5.7 (2.4–9.2)	15.5 (8.9–24.1)	15.5 (8.9–24.1)	16.7 (10.6–23.2)	16.7 (10.7–22.9)	4.5 (2.3–6.7)	4.5 (2.3–6.7)	5.1 (<0.1–11.3)	5.1 (<0.1–11.3)
Turkey	0.1 (<0.1–0.2)	0.1 (<0.1–0.2)	5.8 (2.4–9.3)	5.8 (2.4–9.3)	13.5 (8.4–20.4)	13.5 (8.4–20.4)	17.4 (10.9–23.8)	18.1 (11.5–24.8)	0.4 (0.1–0.7)	0.4 (0.1–0.7)	18.3 (13.7–23.4)	18.3 (13.7–23.4)
United Arab Emirates	<0.1 (<0.1–<0.1)	<0.1 (<0.1–<0.1)	3.8 (1.5–6.2)	3.8 (1.5–6.2)	21.1 (13.2–30.8)	21.1 (13.2–30.8)	15.0 (9.5–21.3)	15.6 (9.9–21.6)	8.4 (<0.1–25.0)	8.4 (<0.1–25.0)	1.9 (<0.1–18.2)	1.9 (<0.1–18.2)
Yemen	14.7 (7.0–25.0)	14.7 (7.0–25.0)	13.5 (5.8–20.7)	13.5 (5.8–20.7)	16.2 (8.4–26.1)	16.2 (8.4–26.1)	15.6 (10.1–21.4)	15.6 (9.9–21.4)	5.5 (1.6–12.3)	5.5 (1.6–12.3)	0.9 (<0.1–5.1)	0.9 (<0.1–5.1)
**South Asia**	**21.1 (12.0–31.8)**	**20.5 (11.6–30.9)**	**12.7 (5.6–19.6)**	**12.9 (5.7–19.7)**	**22.0 (15.7–29.2)**	**23.4 (17.0–30.4)**	**9.2 (4.9–13.9)**	**9.7 (5.3–14.8)**	**8.0 (4.7–12.1)**	**7.8 (4.4–11.9)**	**2.9 (<0.1–8.3)**	**2.6 (<0.1–7.7)**
Bangladesh	25.8 (14.7–39.0)	25.8 (14.7–39.0)	18.1 (8.1–27.1)	18.1 (8.1–27.1)	18.3 (12.4–25.1)	18.3 (12.4–25.1)	10.0 (4.8–15.8)	10.0 (4.8–15.6)	5.9 (2.7–9.8)	5.9 (2.7–9.8)	1.3 (<0.1–4.8)	1.3 (<0.1–4.8)
Bhutan	19.6 (10.0–31.3)	19.6 (10.0–31.3)	6.1 (2.5–9.8)	6.1 (2.5–9.8)	14.0 (9.2–20.5)	14.0 (9.2–20.5)	4.8 (2.6–7.4)	4.9 (2.6–7.5)	<0.1 (<0.1–0.1)	<0.1 (<0.1–0.1)	18.6 (11.8–26.9)	18.6 (11.8–26.9)
India	20.1 (11.5–30.2)	19.2 (10.9–28.9)	11.9 (5.2–18.5)	12.1 (5.3–18.6)	24.1 (17.6–31.4)	25.4 (18.6–32.6)	9.5 (5.2–14.3)	9.9 (5.5–15.2)	7.6 (4.1–12.0)	7.9 (4.3–12.3)	2.2 (<0.1–7.2)	2.2 (<0.1–7.4)
Nepal	26.5 (15.1–39.3)	26.5 (15.1–39.3)	11.3 (4.9–17.8)	11.3 (4.9–17.8)	20.7 (12.3–29.8)	20.7 (12.3–29.8)	7.0 (3.1–11.6)	7.1 (3.1–11.8)	1.4 (0.7–2.2)	1.4 (0.7–2.2)	13.0 (7.6–19.0)	13.0 (7.6–19.0)
Pakistan	21.2 (11.2–33.0)	20.9 (11.1–32.5)	12.4 (5.3–19.2)	12.4 (5.3–19.2)	19.1 (12.5–26.8)	19.3 (12.8–27.1)	8.4 (4.4–12.6)	8.4 (4.4–12.7)	9.6 (5.9–15.6)	9.6 (5.8–15.5)	4.6 (<0.1–12.2)	4.6 (<0.1–12.4)

	Household air pollution		Handwashing		Ambient particulate matter		Secondhand smoke		High temperature		Low temperature	
	**M**	**F**	**M**	**F**	**M**	**F**	**M**	**F**	**M**	**F**	**M**	**F**
**Southeast Asia, East Asia, and Oceania**	**13.5 (6.9–21.9)**	**13.8 (7.1–22.4)**	**6.3 (2.6–10.2)**	**6.5 (2.7–10.3)**	**12.1 (8.3–16.5)**	**11.3 (7.8–15.5)**	**15.1 (9.3–21.1)**	**14.8 (8.9–20.6)**	**1.5 (<0.1–9.4)**	**1.6 (<0.1–10.6)**	**4.2 (2.5–6.0)**	**3.5 (1.9–5.0)**
East Asia	8.1 (3.5–14.8)	8.5 (3.8–15.5)	4.1 (1.7–6.7)	4.1 (1.7–6.6)	19.4 (13.8–25.9)	19.0 (13.3–25.3)	19.5 (12.9–26.6)	19.5 (12.7–26.3)	0.6 (<0.1–1.1)	0.6 (<0.1–1.1)	13.4 (8.4–18.0)	13.4 (8.4–18.2)
China	7.6 (3.2–14.2)	7.8 (3.3–14.5)	4.2 (1.7–6.9)	4.2 (1.7–6.8)	19.7 (13.9–26.1)	19.3 (13.5–25.6)	19.6 (13.0–26.7)	19.7 (12.7–26.5)	0.6 (<0.1–1.1)	0.6 (<0.1–1.2)	13.3 (8.2–17.8)	13.2 (8.2–17.8)
Democratic People's Republic of Korea	23.7 (12.8–36.8)	23.7 (12.8–36.8)	2.3 (0.9–3.7)	2.3 (0.9–3.7)	15.2 (9.5–21.8)	15.2 (9.5–21.8)	17.9 (11.5–25.1)	18.0 (11.7–24.9)	0.1 (<0.1–0.7)	0.1 (<0.1–0.7)	19.3 (11.7–29.7)	19.3 (11.7–29.7)
Taiwan (Province of China)	0.6 (0.1–1.6)	0.6 (0.1–1.6)	1.8 (0.7–3.0)	1.8 (0.7–3.0)	12.1 (7.5–17.9)	12.1 (7.5–17.9)	14.0 (8.5–19.8)	15.1 (9.6–20.9)	0.9 (0.2–2.3)	0.9 (0.2–2.3)	3.5 (<0.1–6.8)	3.5 (<0.1–6.8)
Oceania	38.2 (23.6–51.1)	38.1 (23.9–51.2)	10.6 (4.5–16.9)	10.6 (4.5–16.8)	4.1 (1.1–10.8)	4.1 (1.1–10.8)	9.7 (4.6–15.5)	9.5 (4.6–15.2)	0.5 (<0.1–2.2)	0.5 (<0.1–2.3)	1.2 (<0.1–3.0)	1.2 (<0.1–2.9)
Fiji	5.6 (1.7–12.4)	5.6 (1.7–12.4)	2.9 (1.2–4.8)	2.9 (1.2–4.8)	5.4 (1.5–13.1)	5.4 (1.5–13.1)	7.1 (3.1–11.9)	7.1 (3.2–11.9)	0.5 (<0.1–2.4)	0.5 (<0.1–2.4)	<0.1 (<0.1–1.7)	<0.1 (<0.1–1.7)
Kiribati	28.4 (16.1–42.4)	28.4 (16.1–42.4)	6.5 (2.7–10.4)	6.5 (2.7–10.4)	3.4 (1.1–7.9)	3.4 (1.1–7.9)	12.5 (6.6–18.4)	12.1 (6.4–17.9)	0.9 (<0.1–5.0)	0.9 (<0.1–5.0)	<0.1 (<0.1–0.1)	<0.1 (<0.1–0.1)
Marshall Islands	9.7 (3.8–18.5)	9.7 (3.8–18.5)	4.5 (1.8–7.3)	4.5 (1.8–7.3)	4.2 (1.6–9.1)	4.2 (1.6–9.1)	9.9 (5.2–15.2)	10.1 (5.3–15.3)	0.2 (<0.1–11.3)	0.2 (<0.1–11.3)	<0.1 (<0.1–0.1)	<0.1 (<0.1–0.1)
Micronesia (Federated States of)	8.6 (3.0–16.8)	8.6 (3.0–16.8)	3.9 (1.6–6.4)	3.9 (1.6–6.4)	5.0 (1.5–11.8)	5.0 (1.5–11.8)	8.1 (3.6–13.6)	8.1 (3.6–13.6)	0.2 (<0.1–11.3)	0.2 (<0.1–11.3)	<0.1 (<0.1–0.2)	<0.1 (<0.1–0.2)
Nauru	0.7 (0.1–2.1)	0.7 (0.1–2.1)	3.1 (1.2–5.1)	3.1 (1.2–5.1)	2.0 (0.5–5.2)	2.0 (0.5–5.2)	8.8 (3.9–14.9)	8.6 (3.9–14.7)	2.4 (<0.1–31.1)	2.4 (<0.1–31.1)	<0.1 (<0.1–0.3)	<0.1 (<0.1–0.3)
Palau	<0.1 (<0.1–<0.1)	<0.1 (<0.1–<0.1)	2.4 (0.9–3.9)	2.4 (0.9–3.9)	2.0 (<0.1–5.9)	2.0 (<0.1–5.9)	12.4 (6.9–18.8)	12.2 (6.8–18.1)	0.9 (<0.1–28.7)	0.9 (<0.1–28.7)	<0.1 (<0.1–0.4)	<0.1 (<0.1–0.4)
Papua New Guinea	42.2 (26.2–55.8)	42.2 (26.2–55.8)	12.2 (5.2–19.0)	12.2 (5.2–19.0)	4.1 (1.0–11.1)	4.1 (1.0–11.1)	9.8 (4.6–15.9)	9.6 (4.6–15.5)	0.4 (<0.1–1.9)	0.4 (<0.1–1.9)	1.6 (<0.1–3.7)	1.6 (<0.1–3.7)
Samoa	18.4 (8.3–31.4)	18.4 (8.3–31.4)	2.2 (0.9–3.6)	2.2 (0.9–3.6)	4.9 (1.4–12.0)	4.9 (1.4–12.0)	14.8 (8.7–21.0)	14.7 (8.6–20.5)	0.4 (<0.1–2.2)	0.4 (<0.1–2.2)	0.1 (<0.1–1.4)	0.1 (<0.1–1.4)
Solomon Islands	42.7 (27.0–56.9)	42.7 (27.0–56.9)	4.9 (2.0–7.9)	4.9 (2.0–7.9)	3.4 (1.0–8.3)	3.4 (1.0–8.3)	10.3 (5.0–16.0)	10.3 (5.4–15.9)	0.5 (<0.1–3.3)	0.5 (<0.1–3.3)	<0.1 (<0.1–0.5)	<0.1 (<0.1–0.5)
Tonga	8.7 (3.0–18.1)	8.7 (3.0–18.1)	3.3 (1.3–5.4)	3.3 (1.3–5.4)	5.2 (1.6–11.8)	5.2 (1.6–11.8)	11.8 (6.3–17.5)	11.8 (6.5–17.5)	1.2 (<0.1–3.0)	1.2 (<0.1–3.0)	0.2 (<0.1–2.6)	0.2 (<0.1–2.6)
Tuvalu	2.3 (0.5–5.3)	2.3 (0.5–5.3)	3.7 (1.5–6.1)	3.7 (1.5–6.1)	2.5 (0.9–5.6)	2.5 (0.9–5.6)	10.4 (5.2–16.2)	10.3 (5.1–15.9)	1.4 (<0.1–12.8)	1.4 (<0.1–12.8)	0.1 (<0.1–0.7)	0.1 (<0.1–0.7)
Vanuatu	33.3 (19.6–48.5)	33.3 (19.6–48.5)	10.0 (4.3–15.7)	10.0 (4.3–15.7)	4.4 (1.3–10.3)	4.4 (1.3–10.3)	6.7 (2.8–11.2)	6.9 (2.9–11.8)	0.4 (<0.1–2.1)	0.4 (<0.1–2.1)	<0.1 (<0.1–1.8)	<0.1 (<0.1–1.8)
Southeast Asia	14.6 (7.3–23.9)	14.6 (7.3–24.0)	7.1 (2.9–11.3)	7.1 (2.9–11.3)	9.0 (5.9–12.7)	8.9 (5.9–12.6)	13.4 (7.6–19.3)	13.4 (7.8–19.5)	2.0 (<0.1–13.9)	2.0 (<0.1–14.5)	0.1 (<0.1–1.0)	0.1 (<0.1–0.9)

	Household air pollution		Handwashing		Ambient particulate matter		Secondhand smoke		High temperature		Low temperature	
	**M**	**F**	**M**	**F**	**M**	**F**	**M**	**F**	**M**	**F**	**M**	**F**
Cambodia	31.5 (17.9–46.3)	31.5 (17.9–46.3)	8.6 (3.5–13.5)	8.6 (3.5–13.5)	7.3 (3.9–11.8)	7.3 (3.9–11.8)	15.6 (9.9–21.8)	15.6 (9.8–21.4)	2.7 (<0.1–21.8)	2.7 (<0.1–21.8)	<0.1 (<0.1–0.3)	<0.1 (<0.1–0.3)
Indonesia	8.0 (3.3–15.9)	8.4 (3.4–16.4)	7.6 (3.1–12.1)	7.8 (3.2–12.3)	9.4 (6.0–13.8)	9.2 (5.9–13.4)	15.4 (8.8–21.9)	15.6 (8.8–22.4)	0.3 (<0.1–3.8)	0.4 (<0.1–3.8)	<0.1 (<0.1–1.0)	<0.1 (<0.1–0.9)
Lao People's Democratic Republic	33.9 (20.0–49.2)	33.9 (20.0–49.2)	10.2 (4.3–15.9)	10.2 (4.3–15.9)	6.7 (3.8–10.5)	6.7 (3.8–10.5)	12.9 (7.1–18.9)	13.0 (7.3–19.2)	1.8 (0.6–3.9)	1.8 (0.6–3.9)	<0.1 (<0.1–2.9)	<0.1 (<0.1–2.9)
Malaysia	0.1 (<0.1–0.3)	0.1 (<0.1–0.3)	2.0 (0.8–3.2)	2.0 (0.8–3.2)	8.3 (4.7–13.2)	8.3 (4.7–13.2)	12.9 (7.3–19.0)	13.1 (7.4–18.9)	0.8 (<0.1–3.9)	0.8 (<0.1–3.9)	<0.1 (<0.1–0.8)	<0.1 (<0.1–0.8)
Maldives	2.8 (0.9–6.8)	2.8 (0.9–6.8)	8.1 (3.5–13.0)	8.1 (3.5–13.0)	5.2 (2.9–8.5)	5.2 (2.9–8.5)	14.9 (8.8–21.5)	14.8 (8.5–21.2)	0.4 (<0.1–11.4)	0.4 (<0.1–11.4)	<0.1 (<0.1–0.1)	<0.1 (<0.1–0.1)
Mauritius	0.2 (<0.1–0.5)	0.2 (<0.1–0.5)	1.1 (0.4–1.8)	1.1 (0.4–1.8)	7.3 (3.1–13.8)	7.3 (3.1–13.8)	11.6 (6.3–17.3)	11.8 (6.4–17.6)	0.7 (<0.1–3.5)	0.7 (<0.1–3.5)	<0.1 (<0.1–5.4)	<0.1 (<0.1–5.4)
Myanmar	24.3 (12.8–38.5)	24.3 (12.8–38.5)	11.1 (4.7–17.5)	11.1 (4.7–17.5)	10.9 (7.1–15.4)	10.9 (7.1–15.4)	8.4 (3.7–14.1)	8.7 (3.9–14.3)	2.9 (1.3–5.3)	2.9 (1.3–5.3)	1.9 (<0.1–4.3)	1.9 (<0.1–4.3)
Philippines	15.3 (7.3–25.6)	15.2 (7.2–25.6)	6.4 (2.6–10.1)	6.4 (2.6–10.1)	8.6 (5.7–12.1)	8.6 (5.6–12.2)	13.1 (7.3–19.2)	13.3 (7.6–19.1)	2.4 (<0.1–23.2)	2.4 (<0.1–22.7)	0.2 (<0.1–0.8)	0.2 (<0.1–0.8)
Seychelles	0.1 (<0.1–0.2)	0.1 (<0.1–0.2)	3.9 (1.6–6.3)	3.9 (1.6–6.3)	7.7 (3.3–14.3)	7.7 (3.3–14.3)	11.1 (6.1–16.5)	12.0 (6.7–17.4)	1.0 (<0.1–3.8)	1.0 (<0.1–3.8)	0.2 (<0.1–1.3)	0.2 (<0.1–1.3)
Sri Lanka	9.8 (3.4–20.2)	9.8 (3.4–20.2)	5.0 (2.0–8.1)	5.0 (2.0–8.1)	9.7 (5.7–15.1)	9.7 (5.7–15.1)	6.1 (2.4–11.0)	6.5 (2.7–11.2)	1.2 (<0.1–13.3)	1.2 (<0.1–13.3)	<0.1 (<0.1–0.8)	<0.1 (<0.1–0.8)
Thailand	3.5 (1.1–7.9)	3.5 (1.1–7.9)	7.5 (3.1–12.1)	7.5 (3.1–12.1)	13.7 (8.8–19.8)	13.7 (8.8–19.8)	10.1 (5.6–15.1)	10.0 (5.4–15.0)	2.9 (<0.1–10.6)	2.9 (<0.1–10.6)	<0.1 (<0.1–1.0)	<0.1 (<0.1–1.0)
Timor-Leste	30.0 (16.7–45.1)	30.0 (16.7–45.1)	6.6 (2.7–10.6)	6.6 (2.7–10.6)	5.6 (2.6–10.1)	5.6 (2.6–10.1)	10.7 (5.2–16.8)	10.9 (5.1–17.4)	0.5 (<0.1–2.1)	0.5 (<0.1–2.1)	<0.1 (<0.1–1.7)	<0.1 (<0.1–1.7)
Viet Nam	9.8 (3.8–18.3)	9.8 (3.8–18.3)	8.6 (3.6–13.6)	8.6 (3.6–13.6)	9.7 (6.3–14.1)	9.7 (6.3–14.1)	15.1 (9.1–21.2)	15.2 (8.9–21.5)	2.7 (0.9–5.9)	2.7 (0.9–5.9)	0.2 (<0.1–3.2)	0.2 (<0.1–3.2)
**Sub-Saharan Africa**	**40.0 (27.6–52.2)**	**40.7 (28.1–52.9)**	**22.1 (10.1–32.5)**	**22.1 (10.2–32.5)**	**9.2 (5.2–14.4)**	**8.9 (5.0–14.0)**	**3.7 (1.7–6.1)**	**3.8 (1.7–6.1)**	**3.9 (0.7–11.7)**	**3.7 (0.7–10.9)**	**1.1 (<0.1–3.1)**	**1.0 (<0.1–3.1)**
Central Sub-Saharan Africa	36.5 (23.1–49.3)	37.5 (23.5–49.8)	22.6 (10.4–33.2)	22.7 (10.5–33.2)	8.8 (4.1–15.1)	8.6 (3.9–14.9)	3.0 (1.4–5.2)	2.7 (1.2–4.9)	1.6 (0.4–3.4)	1.5 (0.3–3.4)	<0.1 (<0.1–2.4)	<0.1 (<0.1–2.4)
Angola	17.9 (9.0–28.8)	17.9 (9.0–28.8)	22.3 (10.2–32.8)	22.3 (10.2–32.8)	11.0 (5.7–18.3)	11.0 (5.7–18.3)	7.4 (4.0–11.6)	7.2 (4.0–11.0)	1.7 (0.5–3.8)	1.7 (0.5–3.8)	<0.1 (<0.1–2.9)	<0.1 (<0.1–2.9)
Central African Republic	49.7 (35.1–62.3)	49.7 (35.1–62.3)	21.6 (9.9–31.8)	21.6 (9.9–31.8)	6.5 (2.1–14.1)	6.5 (2.1–14.1)	3.4 (1.5–5.6)	3.4 (1.6–5.9)	3.8 (1.5–7.0)	3.8 (1.5–7.0)	<0.1 (<0.1–1.2)	<0.1 (<0.1–1.2)
Congo	14.7 (7.0–26.0)	14.7 (7.0–26.0)	21.0 (9.5–31.0)	21.0 (9.5–31.0)	15.7 (8.4–26.9)	15.7 (8.4–26.9)	4.3 (1.9–7.1)	4.2 (2.0–7.0)	0.9 (<0.1–3.0)	0.9 (<0.1–3.0)	<0.1 (<0.1–1.9)	<0.1 (<0.1–1.9)
Democratic Republic of the Congo	42.7 (27.5–55.5)	42.7 (27.5–55.5)	23.0 (10.6–33.7)	23.0 (10.6–33.7)	7.9 (3.2–14.5)	7.9 (3.2–14.5)	1.4 (0.4–2.9)	1.4 (0.4–2.9)	1.3 (0.1–3.1)	1.3 (0.1–3.1)	0.1 (<0.1–2.6)	0.1 (<0.1–2.6)

	Household air pollution		Handwashing		Ambient particulate matter		Secondhand smoke		High temperature		Low temperature	
	**M**	**F**	**M**	**F**	**M**	**F**	**M**	**F**	**M**	**F**	**M**	**F**
Equatorial Guinea	4.2 (1.4–9.1)	4.2 (1.4–9.1)	19.9 (9.0–29.7)	19.9 (9.0–29.7)	20.2 (10.1–33.3)	20.2 (10.1–33.3)	3.9 (1.6–6.8)	4.0 (1.6–7.4)	0.4 (<0.1–1.3)	0.4 (<0.1–1.3)	<0.1 (<0.1–2.4)	<0.1 (<0.1–2.4)
Gabon	1.6 (0.5–3.3)	1.6 (0.5–3.3)	16.9 (7.4–25.6)	16.9 (7.4–25.6)	17.7 (9.2–29.9)	17.7 (9.2–29.9)	4.5 (2.0–7.8)	4.6 (2.1–7.8)	0.7 (<0.1–2.6)	0.7 (<0.1–2.6)	<0.1 (<0.1–1.5)	<0.1 (<0.1–1.5)
Eastern Sub-Saharan Africa	45.2 (31.7–58.1)	45.3 (31.2–58.2)	22.6 (10.4–33.2)	22.6 (10.4–33.2)	5.8 (2.9–9.8)	5.7 (2.9–9.6)	3.5 (1.5–6.0)	3.5 (1.5–6.2)	2.1 (0.4–5.8)	2.0 (0.4–5.5)	1.2 (<0.1–3.7)	1.1 (<0.1–3.6)
Burundi	51.0 (37.0–63.4)	51.0 (37.0–63.4)	22.9 (10.6–33.7)	22.9 (10.6–33.7)	4.8 (1.5–10.8)	4.8 (1.5–10.8)	2.4 (0.9–4.5)	2.3 (0.8–4.4)	<0.1 (<0.1–0.3)	<0.1 (<0.1–0.3)	<0.1 (<0.1–6.1)	<0.1 (<0.1–6.1)
Comoros	34.0 (20.0–49.0)	34.0 (20.0–49.0)	21.0 (9.6–31.1)	21.0 (9.6–31.1)	5.5 (2.9–9.1)	5.5 (2.9–9.1)	5.2 (2.3–8.6)	5.2 (2.4–8.5)	0.7 (<0.1–2.9)	0.7 (<0.1–2.9)	0.2 (<0.1–2.9)	0.2 (<0.1–2.9)
Djibouti	8.3 (3.4–16.2)	8.3 (3.4–16.2)	21.6 (9.9–32.0)	21.6 (9.9–32.0)	17.9 (8.6–31.1)	17.9 (8.6–31.1)	8.6 (3.9–14.2)	8.5 (3.8–13.9)	5.9 (<0.1–30.0)	5.9 (<0.1–30.0)	0.2 (<0.1–5.2)	0.2 (<0.1–5.2)
Eritrea	33.4 (19.2–46.3)	33.4 (19.2–46.3)	22.6 (10.4–33.1)	22.6 (10.4–33.1)	11.3 (5.0–20.0)	11.3 (5.0–20.0)	4.0 (1.9–6.4)	3.9 (2.0–6.3)	4.5 (<0.1–22.1)	4.5 (<0.1–22.1)	0.1 (<0.1–2.5)	0.1 (<0.1–2.5)
Ethiopia	49.0 (35.6–61.0)	49.0 (35.4–60.9)	23.2 (10.7–33.9)	23.2 (10.7–33.9)	5.5 (2.2–10.7)	5.5 (2.2–10.7)	1.4 (0.5–2.7)	1.4 (0.5–2.6)	3.6 (0.6–11.7)	3.7 (0.7–11.9)	4.1 (2.5–6.0)	4.0 (2.5–5.9)
Kenya	34.8 (22.9–47.4)	35.2 (22.6–47.7)	22.3 (10.3–33.0)	22.4 (10.3–33.0)	6.5 (3.7–10.6)	6.4 (3.6–10.6)	3.9 (1.7–6.7)	4.0 (1.7–7.0)	3.8 (0.3–15.4)	3.9 (0.3–15.2)	2.7 (1.2–4.7)	2.6 (1.1–4.5)
Madagascar	44.4 (28.7–58.2)	44.4 (28.7–58.2)	22.4 (10.3–33.0)	22.4 (10.3–33.0)	4.4 (2.1–8.0)	4.4 (2.1–8.0)	3.5 (1.1–6.7)	3.5 (1.1–6.9)	0.8 (<0.1–2.4)	0.8 (<0.1–2.4)	1.0 (<0.1–3.8)	1.0 (<0.1–3.8)
Malawi	46.6 (30.9–59.0)	46.6 (30.9–59.0)	22.6 (10.4–33.3)	22.6 (10.4–33.3)	4.8 (2.0–9.0)	4.8 (2.0–9.0)	3.5 (1.4–6.1)	3.5 (1.5–6.2)	0.5 (<0.1–1.9)	0.5 (<0.1–1.9)	0.2 (<0.1–4.4)	0.2 (<0.1–4.4)
Mozambique	50.2 (35.8–61.8)	50.2 (35.8–61.8)	22.2 (10.2–32.7)	22.2 (10.2–32.7)	3.6 (1.4–7.0)	3.6 (1.4–7.0)	3.7 (1.2–6.9)	3.6 (1.2–6.8)	2.4 (1.0–4.4)	2.4 (1.0–4.4)	0.2 (<0.1–3.3)	0.2 (<0.1–3.3)
Rwanda	39.6 (24.7–54.3)	39.6 (24.7–54.3)	23.2 (10.7–34.0)	23.2 (10.7–34.0)	9.1 (4.0–16.8)	9.1 (4.0–16.8)	4.7 (2.3–7.4)	4.7 (2.3–7.3)	<0.1 (<0.1–0.1)	<0.1 (<0.1–0.1)	2.0 (<0.1–7.5)	2.0 (<0.1–7.5)
Somalia	60.2 (46.1–83.5)	60.2 (46.1–83.5)	22.7 (10.5–33.4)	22.7 (10.5–33.4)	1.7 (0.3–5.2)	1.7 (0.3–5.2)	3.5 (1.4–6.2)	3.5 (1.4–6.2)	2.3 (<0.1–15.8)	2.3 (<0.1–15.8)	0.1 (<0.1–1.3)	0.1 (<0.1–1.3)
South Sudan	44.2 (28.5–57.1)	44.2 (28.5–57.1)	22.9 (10.6–33.6)	22.9 (10.6–33.6)	7.9 (3.5–14.5)	7.9 (3.5–14.5)	3.7 (1.6–6.3)	3.6 (1.6–6.2)	5.9 (<0.1–23.1)	5.9 (<0.1–23.1)	<0.1 (<0.1–0.9)	<0.1 (<0.1–0.9)
Uganda	41.9 (26.7–55.2)	41.9 (26.7–55.2)	22.1 (10.1–32.5)	22.1 (10.1–32.5)	8.2 (3.9–13.7)	8.2 (3.9–13.7)	3.3 (1.4–5.9)	3.3 (1.3–5.8)	1.1 (0.2–2.9)	1.1 (0.2–2.9)	<0.1 (<0.1–2.3)	<0.1 (<0.1–2.3)
United Republic of Tanzania	41.4 (26.1–55.9)	41.4 (26.1–55.9)	22.5 (10.4–33.2)	22.5 (10.4–33.2)	6.4 (3.4–10.5)	6.4 (3.4–10.5)	6.3 (3.0–10.2)	6.1 (2.9–9.9)	0.9 (0.1–2.7)	0.9 (0.1–2.7)	0.1 (<0.1–3.5)	0.1 (<0.1–3.5)
Zambia	28.3 (15.9–42.9)	28.3 (15.9–42.9)	21.3 (9.7–31.5)	21.3 (9.7–31.5)	9.0 (5.2–14.6)	9.0 (5.2–14.6)	4.5 (1.3–9.1)	4.4 (1.4–8.5)	1.2 (0.3–3.5)	1.2 (0.3–3.5)	<0.1 (<0.1–5.0)	<0.1 (<0.1–5.0)
Southern Sub-Saharan Africa	13.8 (7.3–21.9)	12.1 (6.3–19.4)	17.5 (7.8–26.2)	17.0 (7.6–25.4)	11.4 (7.4–16.8)	12.0 (7.8–17.3)	7.3 (3.6–11.2)	7.5 (3.7–11.8)	1.2 (0.5–2.5)	1.2 (0.5–2.4)	6.1 (3.3–9.1)	6.4 (3.6–9.1)

	Household air pollution		Handwashing		Ambient particulate matter		Secondhand smoke		High temperature		Low temperature	
	**M**	**F**	**M**	**F**	**M**	**F**	**M**	**F**	**M**	**F**	**M**	**F**
Botswana	8.4 (3.2–16.7)	8.4 (3.2–16.7)	17.0 (7.5–25.6)	17.0 (7.5–25.6)	11.8 (7.0–18.1)	11.8 (7.0–18.1)	8.6 (4.2–13.5)	8.6 (4.2–13.8)	4.0 (1.5–7.7)	4.0 (1.5–7.7)	0.9 (<0.1–7.0)	0.9 (<0.1–7.0)
Eswatini	15.4 (7.0–27.1)	15.4 (7.0–27.1)	16.5 (7.4–25.0)	16.5 (7.4–25.0)	10.2 (6.1–15.5)	10.2 (6.1–15.5)	2.5 (1.0–4.6)	2.5 (1.0–4.7)	0.4 (<0.1–1.2)	0.4 (<0.1–1.2)	3.3 (<0.1–8.1)	3.3 (<0.1–8.1)
Lesotho	21.5 (11.2–34.2)	21.5 (11.2–34.2)	23.1 (10.7–33.9)	23.1 (10.7–33.9)	10.4 (6.1–16.4)	10.4 (6.1–16.4)	10.4 (5.8–15.1)	10.4 (5.9–15.0)	<0.1 (<0.1–<0.1)	<0.1 (<0.1–<0.1)	21.7 (15.2–28.4)	21.7 (15.2–28.4)
Namibia	11.7 (4.8–21.6)	11.7 (4.8–21.6)	14.3 (6.2–22.0)	14.3 (6.2–22.0)	11.1 (6.3–17.3)	11.1 (6.3–17.3)	4.4 (1.7–8.1)	4.5 (1.7–8.4)	3.2 (1.3–6.0)	3.2 (1.3–6.0)	1.3 (<0.1–6.0)	1.3 (<0.1–6.0)
South Africa	2.9 (1.1–6.0)	2.9 (1.0–6.0)	14.7 (6.4–22.1)	14.6 (6.4–22.1)	14.6 (9.2–21.3)	14.7 (9.3–21.4)	7.1 (3.2–11.8)	7.5 (3.5–12.4)	0.6 (0.3–1.7)	0.6 (0.2–1.7)	8.8 (6.7–11.3)	8.9 (6.7–11.2)
Zimbabwe	28.7 (16.2–43.3)	28.7 (16.2–43.3)	20.9 (9.6–31.0)	20.9 (9.6–31.0)	7.2 (4.0–11.5)	7.2 (4.0–11.5)	7.4 (4.0–11.2)	7.5 (4.1–11.5)	1.7 (0.7–3.8)	1.7 (0.7–3.8)	0.7 (<0.1–5.1)	0.7 (<0.1–5.1)
Western Sub-Saharan Africa	38.7 (26.8–51.1)	40.2 (27.9–53.1)	21.9 (10.1–32.2)	21.9 (10.1–32.2)	12.4 (6.9–19.9)	11.8 (6.3–19.3)	3.8 (1.9–5.9)	4.0 (2.0–6.2)	6.5 (0.9–21.0)	6.3 (0.8–20.0)	0.8 (<0.1–3.5)	0.8 (<0.1–4.0)
Benin	41.8 (27.0–55.1)	41.8 (27.0–55.1)	22.1 (10.1–32.6)	22.1 (10.1–32.6)	9.7 (4.3–16.9)	9.7 (4.3–16.9)	3.8 (1.8–6.0)	3.9 (1.9–6.3)	3.3 (<0.1–18.5)	3.3 (<0.1–18.5)	<0.1 (<0.1–1.2)	<0.1 (<0.1–1.2)
Burkina Faso	49.2 (35.7–63.0)	49.2 (35.7–63.0)	22.2 (10.2–32.7)	22.2 (10.2–32.7)	6.9 (2.2–14.9)	6.9 (2.2–14.9)	3.7 (1.5–6.3)	3.7 (1.6–6.5)	4.6 (<0.1–34.1)	4.6 (<0.1–34.1)	<0.1 (<0.1–3.1)	<0.1 (<0.1–3.1)
Cabo Verde	7.1 (3.2–13.4)	7.1 (3.2–13.4)	19.0 (8.5–28.3)	19.0 (8.5–28.3)	21.3 (13.3–31.2)	21.3 (13.3–31.2)	4.0 (1.8–6.9)	4.1 (1.9–6.9)	0.2 (<0.1–1.1)	0.2 (<0.1–1.1)	<0.1 (<0.1–6.3)	<0.1 (<0.1–6.3)
Cameroon	23.4 (12.7–36.0)	23.4 (12.7–36.0)	21.8 (10.0–32.1)	21.8 (10.0–32.1)	19.3 (11.9–28.0)	19.3 (11.9–28.0)	3.7 (1.8–6.0)	3.9 (1.9–6.3)	2.0 (0.1–7.1)	2.0 (0.1–7.1)	<0.1 (<0.1–1.6)	<0.1 (<0.1–1.6)
Chad	50.0 (36.3–64.4)	50.0 (36.3–64.4)	22.3 (10.2–32.8)	22.3 (10.2–32.8)	6.9 (2.2–15.5)	6.9 (2.2–15.5)	4.6 (2.1–7.6)	4.7 (2.1–7.9)	7.0 (0.4–22.9)	7.0 (0.4–22.9)	1.3 (<0.1–7.7)	1.3 (<0.1–7.7)
Côte d'Ivoire	33.9 (20.2–47.8)	33.9 (20.2–47.8)	20.8 (9.5–31.0)	20.8 (9.5–31.0)	13.8 (7.0–23.9)	13.8 (7.0–23.9)	6.9 (3.7–10.9)	6.9 (3.6–10.4)	1.6 (<0.1–5.7)	1.6 (<0.1–5.7)	0.2 (<0.1–1.2)	0.2 (<0.1–1.2)
Gambia	39.3 (24.8–52.6)	39.3 (24.8–52.6)	21.9 (10.1–32.3)	21.9 (10.1–32.3)	12.4 (5.9–21.0)	12.4 (5.9–21.0)	7.3 (3.8–11.3)	7.3 (3.9–11.2)	2.2 (<0.1–20.9)	2.2 (<0.1–20.9)	0.1 (<0.1–1.5)	0.1 (<0.1–1.5)
Ghana	17.6 (8.8–30.1)	17.6 (8.8–30.1)	22.3 (10.3–32.8)	22.3 (10.3–32.8)	19.1 (12.0–28.9)	19.1 (12.0–28.9)	2.2 (1.0–3.6)	2.1 (1.0–3.5)	2.9 (<0.1–18.5)	2.9 (<0.1–18.5)	<0.1 (<0.1–0.7)	<0.1 (<0.1–0.7)
Guinea	46.9 (32.2–59.1)	46.9 (32.2–59.1)	22.3 (10.3–32.8)	22.3 (10.3–32.8)	8.3 (3.2–16.4)	8.3 (3.2–16.4)	5.3 (2.6–8.7)	5.2 (2.6–8.2)	2.7 (0.7–7.1)	2.7 (0.7–7.1)	0.1 (<0.1–1.3)	0.1 (<0.1–1.3)
Guinea-Bissau	44.7 (29.9–57.5)	44.7 (29.9–57.5)	22.3 (10.3–32.8)	22.3 (10.3–32.8)	9.5 (3.9–17.8)	9.5 (3.9–17.8)	5.0 (2.4–8.0)	5.0 (2.4–7.7)	2.2 (<0.1–22.7)	2.2 (<0.1–22.7)	<0.1 (<0.1–1.3)	<0.1 (<0.1–1.3)
Liberia	42.5 (27.8–55.2)	42.5 (27.8–55.2)	23.3 (10.8–34.1)	23.3 (10.8–34.1)	10.1 (4.5–17.9)	10.1 (4.5–17.9)	2.7 (1.1–4.7)	2.7 (1.2–4.6)	0.9 (<0.1–3.3)	0.9 (<0.1–3.3)	0.1 (<0.1–2.0)	0.1 (<0.1–2.0)
Mali	49.6 (35.5–63.4)	49.6 (35.5–63.4)	21.9 (10.1–32.3)	21.9 (10.1–32.3)	7.4 (2.4–16.1)	7.4 (2.4–16.1)	3.9 (1.7–6.6)	3.9 (1.8–6.6)	7.3 (<0.1–26.7)	7.3 (<0.1–26.7)	1.3 (<0.1–13.3)	1.3 (<0.1–13.3)

	Household air pollution		Handwashing		Ambient particulate matter		Secondhand smoke		High temperature		Low temperature	
	M	F	M	F	M	F	M	F	M	F	M	F
Mauritania	18.3 (9.0–30.0)	18.3 (9.0–30.0)	19.6 (8.8–29.2)	19.6 (8.8–29.2)	21.2 (12.3–32.0)	21.2 (12.3–32.0)	4.3 (1.8–7.6)	4.2 (1.8–7.3)	8.0 (1.7–19.5)	8.0 (1.7–19.5)	1.9 (<0.1–13.0)	1.9 (<0.1–13.0)
Niger	53.6 (38.2–73.9)	53.6 (38.2–73.9)	21.4 (9.8–31.7)	21.4 (9.8–31.7)	6.1 (1.5–15.9)	6.1 (1.5–15.9)	3.6 (1.7–5.8)	3.6 (1.8–5.8)	8.4 (0.5–23.3)	8.4 (0.5–23.3)	1.6 (<0.1–11.5)	1.6 (<0.1–11.5)
Nigeria	34.9 (23.3–47.5)	36.1 (23.7–49.5)	22.1 (10.1–32.4)	22.2 (10.2–32.6)	15.2 (8.2–24.3)	14.9 (7.7–24.6)	2.7 (1.3–4.2)	2.7 (1.4–4.2)	10.3 (1.8–42.5)	10.5 (2.0–43.2)	1.3 (<0.1–3.7)	1.4 (<0.1–3.9)
Sao Tome and Principe	21.1 (10.6–33.9)	21.1 (10.6–33.9)	17.4 (7.7–26.1)	17.4 (7.7–26.1)	11.6 (6.0–18.7)	11.6 (6.0–18.7)	1.4 (0.7–2.3)	1.5 (0.7–2.5)	0.9 (<0.1–3.7)	0.9 (<0.1–3.7)	<0.1 (<0.1–1.9)	<0.1 (<0.1–1.9)
Senegal	36.2 (22.2–49.0)	36.2 (22.2–49.0)	18.8 (8.4–28.1)	18.8 (8.4–28.1)	13.2 (6.3–22.1)	13.2 (6.3–22.1)	7.4 (4.2–11.0)	7.5 (4.2–11.1)	4.2 (<0.1–30.1)	4.2 (<0.1–30.1)	0.1 (<0.1–2.2)	0.1 (<0.1–2.2)
Sierra Leone	45.4 (30.7–58.1)	45.4 (30.7–58.1)	22.2 (10.2–32.6)	22.2 (10.2–32.6)	8.9 (3.7–16.9)	8.9 (3.7–16.9)	5.5 (2.1–9.8)	5.4 (2.1–9.7)	1.8 (<0.1–4.3)	1.8 (<0.1–4.3)	0.2 (<0.1–1.5)	0.2 (<0.1–1.5)
Togo	36.9 (22.8–50.9)	36.9 (22.8–50.9)	22.0 (10.1–32.5)	22.0 (10.1–32.5)	11.5 (5.9–18.8)	11.5 (5.9–18.8)	5.2 (2.9–7.9)	5.2 (3.1–7.7)	2.7 (<0.1–13.0)	2.7 (<0.1–13.0)	0.1 (<0.1–0.9)	0.1 (<0.1–0.9)

Appendix Table 9. Population-attributable fractions of lower respiratory infections due to evaluated risk factors among people aged 15-49 years in 2019, males (M) and females (F) (95% UI)

	Household air pollution		Smoking		Ambient particulate matter		Handwashing		Secondhand smoke		Alcohol use		High temperature		Low temperature	
	**M**	**F**	**M**	**F**	**M**	**F**	**M**	**F**	**M**	**F**	**M**	**F**	**M**	**F**	**M**	**F**
**Global**	**15.6 (10.0–21.9)**	**21.1 (14.5–27.9)**	**20.4 (15.4–25.2)**	**4.3 (2.8–5.9)**	**13.2 (9.4–17.6)**	**13.4 (9.5–17.8)**	**11.8 (5.3–17.9)**	**12.9 (5.7–19.5)**	**5.8 (3.2–8.6)**	**10.1 (5.8–14.4)**	**9.1 (4.6–12.8)**	**2.0 (0.6–3.4)**	**3.0 (1.3–6.9)**	**3.7 (1.6–8.1)**	**5.3 (2.7–7.9)**	**4.3 (1.9–6.7)**
**Central Europe, Eastern Europe, and Central Asia**	**0.7 (0.2–1.4)**	**1.4 (0.6–2.7)**	**39.9 (33.0–46.4)**	**16.4 (11.3–21.6)**	**7.6 (4.4–12.0)**	**8.7 (5.1–13.4)**	**2.6 (1.1–4.3)**	**2.9 (1.2–4.7)**	**6.9 (4.2–9.7)**	**14.6 (9.3–20.1)**	**16.8 (9.2–23.3)**	**5.6 (1.8–9.4)**	**0.1 (<0.1–0.5)**	**0.2 (<0.1–0.7)**	**17.8 (7.4–28.3)**	**17.8 (8.3–28.0)**
Central Asia	2.4 (1.0–4.7)	3.7 (1.7–6.8)	25.6 (20.1–31.1)	3.4 (2.0–5.0)	14.3 (7.9–22.5)	14.9 (8.1–23.7)	4.5 (1.8–7.2)	4.7 (1.9–7.7)	6.6 (4.0–9.5)	13.3 (8.3–18.4)	9.9 (4.5–14.8)	1.4 (0.0–3.2)	0.8 (<0.1–1.9)	0.8 (<0.1–2.0)	18.2 (13.1–24.4)	18.4 (13.3–24.2)
Armenia	0.4 (0.1–0.9)	0.5 (0.2–1.2)	39.9 (33.4–46.3)	3.1 (1.7–4.9)	16.8 (9.1–26.9)	16.8 (9.2–26.9)	3.8 (1.5–6.2)	3.8 (1.5–6.2)	7.9 (5.0–11.2)	21.5 (14.6–28.9)	6.6 (1.6–11.4)	4.9 (1.9–8.0)	<0.1 (<0.1–0.2)	<0.1 (<0.1–0.2)	18.4 (8.4–31.6)	18.4 (8.4–31.6)
Azerbaijan	0.9 (0.3–2.2)	1.3 (0.4–2.9)	29.6 (23.4–35.6)	2.2 (1.1–3.6)	12.9 (6.1–22.6)	12.9 (6.2–22.5)	3.5 (1.4–5.7)	3.5 (1.4–5.7)	8.8 (5.7–12.3)	21.4 (14.6–28.5)	11.5 (5.0–17.3)	2.8 (0.3–5.8)	0.5 (<0.1–1.1)	0.5 (<0.1–1.1)	15.7 (12.6–19.1)	15.7 (12.6–19.1)
Georgia	4.0 (1.3–8.7)	5.7 (2.0–12.0)	38.7 (32.2–45.4)	6.5 (4.1–9.3)	9.1 (5.3–14.5)	9.0 (5.3–13.7)	3.3 (1.3–5.4)	3.3 (1.3–5.4)	8.5 (5.3–12.2)	20.8 (13.9–27.9)	14.6 (7.8–20.8)	0.8 (0.0–3.0)	<0.1 (<0.1–0.2)	<0.1 (<0.1–0.2)	18.9 (11.2–29.0)	18.9 (11.2–29.0)
Kazakhstan	1.2 (0.3–2.8)	1.8 (0.6–4.1)	36.7 (28.0–44.7)	9.0 (5.3–13.0)	10.3 (5.4–16.8)	10.3 (5.4–16.7)	3.4 (1.4–5.7)	3.4 (1.4–5.7)	5.4 (3.2–8.1)	14.8 (9.1–20.8)	12.2 (5.7–18.5)	3.5 (<0.1–7.5)	0.5 (<0.1–2.0)	0.5 (<0.1–2.0)	18.4 (11.3–27.6)	18.4 (11.3–27.6)
Kyrgyzstan	5.6 (2.3–10.7)	7.6 (3.5–14.0)	29.9 (23.7–35.8)	4.6 (2.9–6.7)	11.7 (6.3–19.4)	11.4 (6.2–18.5)	4.0 (1.6–6.5)	4.0 (1.6–6.5)	7.9 (5.0–11.0)	19.9 (13.2–26.8)	11.2 (5.6–16.1)	1.9 (0.1–3.7)	<0.1 (<0.1–<0.1)	<0.1 (<0.1–<0.1)	21.4 (8.4–37.4)	21.4 (8.4–37.4)
Mongolia	6.7 (2.7–12.9)	9.3 (4.3–16.9)	29.7 (21.4–37.7)	4.1 (2.2–6.3)	17.2 (10.9–24.5)	16.6 (10.7–23.7)	8.5 (3.6–13.6)	8.5 (3.6–13.6)	4.9 (2.7–7.3)	15.4 (9.1–21.9)	14.3 (7.6–20.6)	2.1 (0.0–5.1)	0.2 (<0.1–0.9)	0.2 (<0.1–0.9)	18.3 (3.6–34.9)	18.3 (3.6–34.9)
Tajikistan	8.0 (3.5–14.6)	10.4 (5.1–17.8)	17.8 (13.4–22.6)	1.9 (1.1–2.9)	16.7 (8.1–28.7)	16.0 (7.8–27.2)	7.1 (2.9–11.3)	7.1 (2.9–11.3)	8.4 (5.2–11.8)	13.2 (8.5–18.5)	6.1 (2.2–10.6)	0.6 (0.0–1.8)	0.3 (0.1–0.5)	0.3 (0.1–0.5)	21.4 (9.3–34.9)	21.4 (9.3–34.9)

	Household air pollution		Smoking		Ambient particulate matter		Handwashing		Secondhand smoke		Alcohol use		High temperature		Low temperature	
	M	F	M	F	M	F	M	F	M	F	M	F	M	F	M	F
Turkmenistan	<0.1 (<0.1–<0.1)	<0.1 (<0.1–0.1)	24.5 (19.5–30.1)	3.0 (1.8–4.6)	13.3 (6.3–23.0)	13.3 (6.3–23.0)	4.6 (1.9–7.4)	4.6 (1.9–7.4)	10.6 (7.0–14.7)	20.6 (14.1–27.4)	10.9 (5.1–16.4)	1.1 (0.0–3.0)	2.6 (0.9–4.3)	2.6 (0.9–4.3)	12.7 (10.0–15.4)	12.7 (10.0–15.4)
Uzbekistan	1.7 (0.6–3.6)	2.3 (0.8–5.0)	18.0 (13.4–23.1)	1.8 (0.9–3.0)	16.9 (8.4–27.8)	16.7 (8.4–27.5)	4.5 (1.8–7.3)	4.5 (1.8–7.3)	6.0 (3.4–8.8)	10.6 (6.1–15.3)	8.3 (3.0–13.2)	0.6 (0.0–2.1)	1.0 (<0.1–2.7)	1.0 (<0.1–2.7)	18.0 (13.9–22.0)	18.0 (13.9–22.0)
Central Europe	1.1 (0.3–2.5)	1.6 (0.5–3.6)	34.7 (27.9–41.6)	22.0 (16.2–28.1)	9.8 (6.0–14.7)	9.8 (6.0–14.4)	1.1 (0.4–1.7)	1.1 (0.4–1.7)	8.9 (5.4–12.6)	12.1 (7.4–16.9)	18.7 (10.8–25.1)	4.3 (0.5–8.2)	<0.1 (<0.1–0.1)	<0.1 (<0.1–0.1)	21.0 (14.9–28.5)	21.0 (14.8–28.3)
Albania	4.1 (1.4–9.0)	5.4 (1.9–11.6)	24.6 (18.9–30.4)	6.5 (3.9–9.2)	9.5 (5.8–14.1)	9.4 (5.8–14.0)	1.7 (0.7–2.9)	1.7 (0.7–2.9)	10.3 (6.6–14.6)	18.8 (12.5–25.2)	8.9 (3.8–14.0)	3.0 (0.8–5.5)	<0.1 (<0.1–0.1)	<0.1 (<0.1–0.1)	19.2 (14.6–24.1)	19.2 (14.6–24.1)
Bosnia and Herzegovina	4.0 (1.3–8.7)	5.5 (1.9–11.4)	36.4 (29.8–43.5)	25.0 (18.8–30.9)	14.6 (9.3–20.5)	14.3 (9.3–20.0)	1.3 (0.5–2.1)	1.3 (0.5–2.1)	12.0 (7.7–17.0)	16.0 (10.3–21.6)	13.9 (7.2–19.8)	1.6 (0.0–3.8)	<0.1 (<0.1–<0.1)	<0.1 (<0.1–<0.1)	22.3 (15.8–29.9)	22.3 (15.8–29.9)
Bulgaria	1.7 (0.4–4.2)	2.4 (0.7–5.6)	41.9 (34.9–49.1)	29.5 (22.7–36.6)	10.0 (6.2–14.9)	9.9 (6.2–14.7)	1.1 (0.4–1.8)	1.1 (0.4–1.8)	9.6 (5.8–13.7)	13.4 (8.3–18.6)	18.9 (10.7–25.8)	6.3 (1.4–10.8)	<0.1 (<0.1–<0.1)	<0.1 (<0.1–<0.1)	21.3 (14.8–27.9)	21.3 (14.8–27.9)
Croatia	0.4 (0.1–1.2)	0.6 (0.2–1.7)	37.5 (29.9–44.5)	26.6 (19.5–33.7)	9.4 (5.7–14.3)	9.4 (5.7–14.2)	1.0 (0.4–1.6)	1.0 (0.4–1.6)	13.3 (8.4–18.4)	14.9 (9.5–20.6)	15.2 (7.0–22.5)	2.6 (0.0–6.8)	<0.1 (<0.1–0.1)	<0.1 (<0.1–0.1)	20.6 (13.3–27.4)	20.6 (13.3–27.4)
Czechia	0.1 (<0.1–0.2)	0.1 (<0.1–0.4)	33.7 (26.5–40.7)	22.2 (16.0–28.8)	8.4 (5.0–13.0)	8.4 (5.0–13.0)	0.9 (0.3–1.4)	0.9 (0.3–1.4)	10.2 (6.2–14.5)	13.3 (8.5–18.6)	20.1 (11.6–27.0)	7.8 (2.2–13.1)	<0.1 (<0.1–0.2)	<0.1 (<0.1–0.2)	21.1 (13.6–30.0)	21.1 (13.6–30.0)
Hungary	1.3 (0.3–3.6)	2.0 (0.5–5.4)	38.3 (30.8–45.7)	26.2 (19.5–32.9)	8.4 (5.0–12.9)	8.4 (5.1–12.8)	0.9 (0.4–1.4)	0.9 (0.4–1.4)	10.3 (6.2–14.8)	13.7 (8.7–18.9)	17.5 (9.1–24.7)	2.2 (0.0–5.9)	<0.1 (<0.1–0.2)	<0.1 (<0.1–0.2)	21.4 (11.9–30.6)	21.4 (11.9–30.6)
Montenegro	2.8 (0.7–6.7)	3.9 (1.1–9.0)	35.5 (28.3–42.4)	26.2 (20.0–33.0)	10.9 (6.8–16.0)	10.8 (6.8–15.8)	1.2 (0.5–2.0)	1.2 (0.5–2.0)	13.9 (9.1–19.2)	14.8 (9.8–20.4)	14.4 (7.0–20.6)	3.1 (0.0–7.7)	<0.1 (<0.1–0.1)	<0.1 (<0.1–0.1)	20.4 (14.1–28.1)	20.4 (14.1–28.1)
North Macedonia	2.5 (0.8–5.7)	3.5 (1.2–7.6)	34.6 (27.9–41.2)	20.6 (15.0–26.3)	15.2 (9.7–21.6)	15.0 (9.7–21.3)	1.0 (0.4–1.7)	1.0 (0.4–1.7)	12.8 (8.2–17.7)	15.3 (9.7–21.0)	13.4 (6.2–19.8)	0.6 (0.0–2.6)	<0.1 (<0.1–0.1)	<0.1 (<0.1–0.1)	21.8 (15.6–28.1)	21.8 (15.6–28.1)
Poland	0.9 (0.2–2.4)	1.4 (0.4–3.5)	32.2 (25.4–39.0)	22.9 (16.8–29.1)	11.7 (7.3–17.2)	11.7 (7.3–17.2)	1.0 (0.4–1.6)	1.0 (0.4–1.6)	8.6 (5.0–12.4)	10.9 (6.5–15.3)	18.7 (10.5–25.2)	4.7 (0.4–9.0)	<0.1 (<0.1–0.2)	<0.1 (<0.1–0.2)	21.1 (13.2–30.1)	21.1 (13.3–30.0)
Romania	1.1 (0.3–2.8)	1.6 (0.4–4.0)	35.5 (28.2–43.0)	18.6 (12.8–24.8)	7.9 (4.7–12.1)	7.9 (4.8–12.1)	1.2 (0.5–2.0)	1.2 (0.5–2.0)	8.2 (4.7–11.9)	12.1 (7.1–17.1)	19.4 (11.3–26.2)	3.4 (0.0–7.5)	<0.1 (<0.1–0.1)	<0.1 (<0.1–0.1)	20.7 (14.2–28.2)	20.7 (14.2–28.2)
Serbia	2.0 (0.6–4.9)	2.9 (0.9–6.7)	37.1 (30.2–43.9)	27.5 (20.4–34.7)	13.0 (8.2–19.2)	12.9 (8.2–18.9)	1.0 (0.4–1.6)	1.0 (0.4–1.6)	10.2 (6.2–14.5)	12.4 (7.6–17.4)	16.9 (8.9–23.7)	3.8 (0.8–7.0)	<0.1 (<0.1–0.1)	<0.1 (<0.1–0.1)	21.9 (14.8–28.9)	21.9 (14.8–28.9)
Slovakia	0.1 (<0.1–0.2)	0.1 (<0.1–0.3)	32.0 (24.5–39.4)	18.9 (13.4–25.0)	9.4 (5.5–14.5)	9.4 (5.5–14.4)	0.9 (0.3–1.4)	0.9 (0.3–1.4)	9.0 (5.3–13.1)	11.2 (6.9–16.1)	19.0 (10.8–25.8)	3.8 (0.0–8.5)	<0.1 (<0.1–0.1)	<0.1 (<0.1–0.1)	20.2 (13.2–29.4)	20.2 (13.2–29.4)
Slovenia	0.5 (0.1–1.5)	0.8 (0.2–2.2)	37.3 (30.2–44.0)	25.3 (19.3–31.6)	8.6 (5.3–13.3)	8.6 (5.3–13.2)	0.8 (0.3–1.4)	0.8 (0.3–1.4)	12.3 (7.8–16.9)	13.3 (8.6–18.6)	7.5 (0.0–16.9)	1.5 (0.0–5.8)	<0.1 (<0.1–0.1)	<0.1 (<0.1–0.1)	22.1 (15.2–30.1)	22.1 (15.2–30.1)
Eastern Europe	0.2 (0.1–0.5)	0.3 (0.1–0.8)	43.8 (36.5–50.5)	21.2 (14.7–27.8)	5.8 (3.1–9.9)	5.7 (3.0–9.8)	2.4 (1.0–4.0)	2.4 (1.0–4.0)	6.6 (4.0–9.4)	15.7 (10.1–21.3)	18.1 (9.8–24.9)	7.8 (2.8–12.5)	<0.1 (<0.1–0.3)	<0.1 (<0.1–0.3)	17.2 (3.1–29.6)	17.0 (1.6–30.1)
Belarus	0.1 (<0.1–0.2)	0.1 (<0.1–0.3)	40.1 (31.8–47.6)	22.6 (15.4–29.8)	8.2 (4.7–13.0)	8.2 (4.7–13.0)	1.9 (0.8–3.1)	1.9 (0.8–3.1)	6.5 (3.9–9.5)	14.6 (9.2–20.1)	19.4 (10.8–26.2)	6.2 (1.0–11.9)	<0.1 (<0.1–0.2)	<0.1 (<0.1–0.2)	18.3 (9.6–30.8)	18.3 (9.6–30.8)

	Household air pollution		Smoking		Ambient particulate matter		Handwashing		Secondhand smoke		Alcohol use		High temperature		Low temperature	
	**M**	**F**	**M**	**F**	**M**	**F**	**M**	**F**	**M**	**F**	**M**	**F**	**M**	**F**	**M**	**F**
Estonia	0.4 (<0.1–1.2)	0.6 (0.1–1.9)	40.5 (32.9–48.1)	23.2 (16.2–30.3)	1.7 (0.5–3.5)	1.9 (0.7–3.7)	1.8 (0.7–3.0)	1.8 (0.7–3.0)	7.7 (4.5–11.4)	11.8 (7.3–16.4)	22.2 (13.3–29.5)	8.4 (2.4–14.2)	0.1 (<0.1–0.5)	0.1 (<0.1–0.5)	15.8 (3.4–32.6)	15.8 (3.4–32.6)
Latvia	0.4 (0.1–1.1)	0.6 (0.1–1.7)	40.4 (32.2–48.3)	21.2 (13.8–28.7)	5.6 (3.1–9.1)	5.6 (3.1–9.1)	1.9 (0.8–3.1)	1.9 (0.8–3.1)	5.2 (2.8–7.9)	12.3 (7.3–17.8)	21.6 (12.5–28.5)	6.6 (0.9–11.8)	<0.1 (<0.1–0.3)	<0.1 (<0.1–0.3)	17.2 (7.2–31.3)	17.2 (7.2–31.3)
Lithuania	0.2 (<0.1–0.5)	0.3 (<0.1–0.7)	35.4 (27.2–43.0)	19.9 (14.0–26.1)	4.5 (2.3–7.6)	4.5 (2.3–7.6)	1.9 (0.8–3.2)	1.9 (0.8–3.2)	6.0 (3.4–9.2)	12.0 (7.2–17.1)	20.9 (12.3–27.9)	9.2 (3.2–15.3)	<0.1 (<0.1–0.2)	<0.1 (<0.1–0.2)	19.1 (9.2–31.0)	19.1 (9.2–31.0)
Republic of Moldova	0.7 (0.2–1.7)	1.1 (0.4–2.4)	46.8 (39.4–53.9)	8.9 (5.7–12.7)	6.7 (3.3–11.9)	6.7 (3.4–11.9)	4.6 (1.9–7.4)	4.6 (1.9–7.4)	6.9 (4.5–9.7)	17.9 (12.2–24.0)	18.7 (10.5–25.4)	8.1 (2.8–13.3)	<0.1 (<0.1–0.1)	<0.1 (<0.1–0.1)	22.1 (13.3–30.8)	22.1 (13.3–30.8)
Russian Federation	0.1 (<0.1–0.4)	0.2 (0.1–0.6)	44.7 (37.6–51.6)	22.2 (15.6–28.7)	5.2 (2.6–9.0)	5.2 (2.6–9.1)	2.1 (0.9–3.5)	2.2 (0.9–3.6)	6.6 (4.1–9.5)	15.8 (10.2–21.7)	18.5 (10.4–25.4)	6.9 (2.0–11.9)	<0.1 (<0.1–0.4)	<0.1 (<0.1–0.4)	16.0 (<0.1–32.1)	16.1 (<0.1–32.3)
Ukraine	0.4 (0.1–0.9)	0.6 (0.2–1.3)	41.9 (33.6–49.2)	18.7 (11.8–26.0)	7.1 (3.7–12.4)	7.1 (3.8–12.4)	3.1 (1.2–5.1)	3.1 (1.2–5.1)	6.7 (3.8–9.9)	15.4 (9.7–21.0)	16.8 (8.8–24.1)	10.6 (4.6–16.3)	<0.1 (<0.1–0.2)	<0.1 (<0.1–0.2)	19.7 (13.3–26.3)	19.7 (13.3–26.3)
**High-income**	**0.1 (<0.1–0.2)**	**0.1 (<0.1–0.3)**	**28.9 (22.8–34.8)**	**20.8 (15.8–26.0)**	**5.1 (2.8–8.4)**	**4.6 (2.5–7.7)**	**1.5 (0.6–2.4)**	**1.4 (0.6–2.3)**	**8.6 (5.3–12.1)**	**9.7 (6.0–13.5)**	**14.0 (6.9–19.8)**	**7.9 (2.9–12.4)**	**0.6 (0.3–0.9)**	**0.6 (0.3–1.0)**	**17.0 (12.5–22.0)**	**16.7 (12.4–21.7)**
Australasia	<0.1 (<0.1–<0.1)	<0.1 (<0.1–0.1)	27.0 (20.8–33.6)	21.2 (15.4–27.3)	1.9 (0.5–4.2)	1.9 (0.5–4.2)	1.9 (0.8–3.2)	1.9 (0.8–3.2)	8.2 (4.6–12.1)	8.8 (5.1–13.0)	17.5 (9.5–24.0)	7.3 (1.6–12.7)	6.6 (4.4–8.8)	6.6 (4.4–8.9)	7.1 (4.1–10.1)	7.1 (4.1–10.0)
Australia	<0.1 (<0.1–<0.1)	<0.1 (<0.1–0.1)	27.3 (21.0–34.0)	21.4 (15.5–27.4)	1.9 (0.5–4.3)	1.9 (0.5–4.3)	1.9 (0.8–3.2)	1.9 (0.8–3.2)	8.2 (4.6–12.1)	8.8 (5.2–13.0)	17.8 (9.8–24.5)	7.2 (1.5–12.7)	7.3 (4.8–9.7)	7.3 (4.8–9.7)	5.9 (2.7–8.9)	5.9 (2.7–8.9)
New Zealand	<0.1 (<0.1–<0.1)	<0.1 (<0.1–0.1)	22.8 (16.3–29.4)	19.2 (13.1–25.5)	1.5 (0.2–3.5)	1.5 (0.3–3.5)	2.0 (0.8–3.2)	2.0 (0.8–3.2)	8.6 (4.9–12.7)	8.4 (4.7–12.2)	14.8 (6.8–20.8)	8.6 (2.8–13.9)	<0.1 (<0.1–<0.1)	<0.1 (<0.1–<0.1)	20.5 (13.3–27.8)	20.5 (13.3–27.8)
High-income Asia Pacific	<0.1 (<0.1–<0.1)	<0.1 (<0.1–<0.1)	33.8 (27.0–40.5)	12.2 (8.6–16.2)	8.5 (4.9–13.5)	8.2 (4.7–13.1)	1.9 (0.7–3.1)	1.9 (0.7–3.1)	7.6 (4.6–11.0)	12.9 (8.1–17.9)	12.8 (6.2–19.0)	8.0 (3.1–12.8)	0.5 (<0.1–1.3)	0.5 (<0.1–1.4)	15.7 (12.4–19.0)	15.4 (12.2–18.6)
Brunei Darussalam	<0.1 (<0.1–0.1)	0.1 (<0.1–0.2)	26.5 (20.6–33.1)	4.2 (2.5–6.4)	2.6 (0.8–5.4)	2.6 (0.8–5.4)	2.0 (0.8–3.2)	2.0 (0.8–3.2)	7.0 (4.2–10.1)	11.1 (6.9–15.8)	0.2 (0.0–1.5)	0.1 (0.0–0.7)	0.9 (<0.1–5.8)	0.9 (<0.1–5.8)	<0.1 (<0.1–0.5)	<0.1 (<0.1–0.5)
Japan	<0.1 (<0.1–<0.1)	<0.1 (<0.1–0.1)	33.8 (26.8–40.7)	14.5 (10.3–19.2)	6.4 (3.5–10.5)	6.4 (3.5–10.5)	1.9 (0.7–3.1)	1.9 (0.7–3.1)	8.2 (5.0–11.9)	13.6 (8.5–18.7)	12.0 (5.4–18.7)	7.9 (2.6–13.5)	0.5 (<0.1–1.0)	0.5 (<0.1–1.0)	16.0 (12.9–19.4)	16.0 (12.9–19.3)
Republic of Korea	<0.1 (<0.1–<0.1)	<0.1 (<0.1–<0.1)	38.3 (30.8–45.5)	6.9 (4.5–10.0)	14.2 (8.6–21.1)	14.2 (8.6–21.1)	1.9 (0.7–3.1)	1.9 (0.7–3.1)	6.8 (3.9–10.0)	13.0 (8.0–18.1)	18.5 (10.5–25.3)	11.4 (5.6–17.1)	0.2 (<0.1–0.9)	0.2 (<0.1–0.9)	20.4 (14.7–25.9)	20.4 (14.7–25.9)
Singapore	<0.1 (<0.1–<0.1)	<0.1 (<0.1–0.1)	20.2 (14.4–26.4)	7.2 (4.3–10.5)	9.5 (5.0–16.2)	9.5 (5.0–16.2)	1.9 (0.8–3.1)	1.9 (0.8–3.1)	5.7 (3.1–8.4)	8.0 (4.6–11.6)	3.3 (0.0–7.1)	1.4 (0.0–3.3)	1.3 (<0.1–11.7)	1.3 (<0.1–11.7)	0.1 (<0.1–1.2)	0.1 (<0.1–1.2)
High-income North America	<0.1 (<0.1–<0.1)	<0.1 (<0.1–<0.1)	27.2 (21.6–32.8)	21.5 (16.4–26.4)	2.6 (1.2–4.9)	2.6 (1.2–5.0)	1.0 (0.4–1.6)	1.0 (0.4–1.6)	9.2 (5.8–13.1)	8.9 (5.7–12.7)	13.4 (6.8–18.8)	9.0 (3.8–13.6)	0.7 (0.3–1.1)	0.7 (0.3–1.0)	15.3 (11.5–19.2)	15.1 (11.4–19.0)
Canada	<0.1 (<0.1–<0.1)	<0.1 (<0.1–<0.1)	26.5 (20.6–32.5)	20.9 (15.5–26.9)	2.2 (0.9–4.4)	2.2 (0.9–4.4)	1.0 (0.4–1.7)	1.0 (0.4–1.7)	9.2 (5.8–13.0)	9.2 (5.7–12.9)	14.4 (7.0–20.5)	8.4 (2.8–13.5)	<0.1 (<0.1–0.3)	<0.1 (<0.1–0.3)	20.8 (0.8–39.3)	20.8 (0.8–39.3)
United States of America	<0.1 (<0.1–<0.1)	<0.1 (<0.1–<0.1)	27.2 (21.6–32.8)	21.5 (16.5–26.5)	2.6 (1.2–5.0)	2.6 (1.2–5.0)	1.0 (0.4–1.6)	1.0 (0.4–1.6)	9.2 (5.8–13.0)	8.9 (5.7–12.7)	13.3 (6.8–18.8)	9.0 (3.9–13.5)	0.7 (0.3–1.1)	0.7 (0.3–1.1)	14.9 (11.5–18.6)	14.8 (11.4–18.3)

	Household air pollution		Smoking		Ambient particulate matter		Handwashing		Secondhand smoke		Alcohol use		High temperature		Low temperature	
	**M**	**F**	**M**	**F**	**M**	**F**	**M**	**F**	**M**	**F**	**M**	**F**	**M**	**F**	**M**	**F**
Southern Latin America	0.3 (0.1–0.8)	0.5 (0.1–1.1)	26.3 (20.2–32.4)	19.2 (13.4–24.7)	6.9 (3.7–11.6)	6.7 (3.6–11.3)	2.4 (1.0–4.0)	2.5 (1.0–4.1)	7.5 (4.1–11.1)	9.2 (5.2–13.5)	13.5 (6.0–20.0)	6.2 (1.0–11.2)	0.8 (0.5–1.2)	0.9 (0.5–1.3)	16.2 (10.3–22.8)	16.0 (10.3–22.4)
Argentina	0.3 (0.1–0.8)	0.4 (0.1–1.0)	25.8 (19.7–31.7)	18.8 (13.0–24.2)	6.5 (3.4–11.1)	6.5 (3.4–11.1)	2.5 (1.0–4.1)	2.5 (1.0–4.1)	7.5 (4.2–11.1)	9.2 (5.2–13.5)	13.5 (6.0–20.1)	6.2 (1.0–11.3)	0.9 (0.5–1.4)	0.9 (0.5–1.4)	16.1 (10.5–22.4)	16.1 (10.5–22.4)
Chile	0.6 (0.1–1.4)	0.8 (0.2–2.1)	30.8 (22.0–38.8)	24.5 (16.1–32.3)	11.7 (7.1–18.0)	11.7 (7.1–17.9)	2.0 (0.8–3.4)	2.0 (0.8–3.4)	7.3 (3.7–11.3)	8.1 (4.1–12.5)	14.0 (6.5–20.9)	6.5 (1.5–11.9)	<0.1 (<0.1–<0.1)	<0.1 (<0.1–<0.1)	19.3 (10.4–29.4)	19.3 (10.4–29.4)
Uruguay	0.2 (0.1–0.6)	0.3 (0.1–0.9)	29.7 (23.9–35.9)	22.7 (16.7–28.9)	4.0 (1.7–7.6)	4.0 (1.8–7.6)	2.3 (0.9–3.7)	2.3 (0.9–3.7)	8.7 (5.2–12.6)	10.7 (6.4–15.3)	12.6 (5.7–18.8)	5.5 (1.1–10.1)	0.1 (<0.1–0.3)	0.1 (<0.1–0.3)	8.0 (4.5–11.6)	8.0 (4.5–11.6)
Western Europe	<0.1 (<0.1–<0.1)	<0.1 (<0.1–0.1)	31.0 (24.2–37.9)	24.6 (18.0–31.1)	4.9 (2.7–8.2)	4.9 (2.7–8.1)	1.0 (0.4–1.7)	1.0 (0.4–1.7)	9.2 (5.6–13.1)	10.2 (6.2–14.4)	15.8 (8.3–21.9)	7.4 (2.5–12.1)	0.1 (<0.1–0.2)	0.1 (<0.1–0.2)	20.9 (14.9–27.3)	21.3 (15.0–28.1)
Andorra	<0.1 (<0.1–<0.1)	<0.1 (<0.1–<0.1)	32.7 (24.2–40.4)	25.6 (17.6–33.9)	3.5 (1.7–6.3)	3.5 (1.7–6.3)	1.0 (0.4–1.6)	1.0 (0.4–1.6)	9.6 (5.8–13.8)	11.2 (6.8–15.9)	16.2 (8.1–23.1)	7.0 (1.4–12.0)	<0.1 (<0.1–<0.1)	<0.1 (<0.1–<0.1)	17.0 (1.9–35.8)	17.0 (1.9–35.8)
Austria	<0.1 (<0.1–0.1)	<0.1 (<0.1–0.1)	35.9 (29.3–42.4)	27.1 (20.9–33.6)	5.6 (3.1–9.1)	5.6 (3.1–9.1)	1.0 (0.4–1.6)	1.0 (0.4–1.6)	12.3 (7.9–17.0)	13.5 (8.9–18.5)	18.1 (9.8–24.5)	7.6 (2.3–12.7)	<0.1 (<0.1–0.1)	<0.1 (<0.1–0.1)	19.7 (10.6–31.5)	19.7 (10.6–31.5)
Belgium	<0.1 (<0.1–<0.1)	<0.1 (<0.1–<0.1)	32.0 (25.7–38.5)	25.0 (19.0–31.2)	5.9 (3.4–9.7)	5.9 (3.4–9.7)	1.0 (0.4–1.6)	1.0 (0.4–1.6)	11.0 (6.8–15.6)	10.9 (6.8–15.3)	16.9 (9.2–23.7)	8.9 (3.2–14.3)	<0.1 (<0.1–0.1)	<0.1 (<0.1–0.1)	23.4 (14.7–32.7)	23.4 (14.7–32.7)
Cyprus	<0.1 (<0.1–<0.1)	<0.1 (<0.1–0.1)	38.8 (31.0–46.6)	24.1 (17.7–31.2)	7.7 (4.5–12.5)	7.7 (4.5–12.5)	1.0 (0.4–1.6)	1.0 (0.4–1.6)	10.2 (6.3–14.5)	15.2 (9.5–20.9)	17.3 (8.8–24.1)	4.2 (0.1–8.2)	2.1 (0.9–3.6)	2.1 (0.9–3.6)	4.5 (<0.1–10.9)	4.5 (<0.1–10.9)
Denmark	<0.1 (<0.1–<0.1)	<0.1 (<0.1–<0.1)	28.9 (22.4–35.0)	24.3 (18.3–30.0)	4.0 (2.1–7.2)	4.0 (2.1–7.2)	1.0 (0.4–1.6)	1.0 (0.4–1.6)	9.9 (6.0–14.4)	9.9 (6.2–14.3)	12.3 (5.1–18.9)	6.6 (0.9–12.1)	<0.1 (<0.1–<0.1)	<0.1 (<0.1–<0.1)	23.2 (13.0–34.4)	23.2 (13.0–34.4)
Finland	<0.1 (<0.1–<0.1)	<0.1 (<0.1–<0.1)	27.2 (20.9–33.7)	18.8 (13.4–24.5)	1.2 (0.2–2.9)	1.2 (0.2–2.9)	1.0 (0.4–1.7)	1.0 (0.4–1.7)	7.7 (4.4–11.4)	8.5 (5.0–12.0)	16.6 (8.6–23.1)	6.2 (0.8–11.9)	<0.1 (<0.1–0.3)	<0.1 (<0.1–0.3)	17.9 (3.8–36.4)	17.9 (3.8–36.4)
France	<0.1 (<0.1–<0.1)	<0.1 (<0.1–<0.1)	30.5 (22.4–38.3)	25.7 (18.6–32.9)	5.1 (2.8–8.5)	5.1 (2.8–8.5)	1.0 (0.4–1.7)	1.0 (0.4–1.7)	7.9 (4.3–12.0)	8.1 (4.5–11.9)	17.4 (9.1–24.4)	8.4 (2.5–13.9)	<0.1 (<0.1–0.1)	<0.1 (<0.1–0.1)	21.7 (14.9–28.4)	21.7 (14.9–28.4)
Germany	<0.1 (<0.1–<0.1)	<0.1 (<0.1–<0.1)	31.8 (25.0–38.7)	22.8 (16.5–29.3)	5.3 (3.0–8.8)	5.3 (3.0–8.8)	1.0 (0.4–1.6)	1.0 (0.4–1.6)	9.7 (5.8–13.9)	10.1 (6.0–14.3)	18.3 (10.3–25.4)	9.7 (3.1–15.5)	<0.1 (<0.1–0.2)	<0.1 (<0.1–0.2)	22.5 (14.3–31.1)	22.5 (14.3–31.1)
Greece	<0.1 (<0.1–0.1)	0.1 (<0.1–0.2)	39.7 (31.9–46.7)	31.5 (23.7–39.0)	6.9 (4.0–10.9)	6.9 (4.0–10.9)	1.0 (0.4–1.7)	1.0 (0.4–1.7)	10.3 (6.3–14.9)	13.1 (8.2–18.4)	14.7 (7.0–21.3)	3.1 (0.0–7.6)	0.1 (<0.1–0.3)	0.1 (<0.1–0.3)	15.7 (12.3–19.4)	15.7 (12.3–19.4)
Iceland	<0.1 (<0.1–<0.1)	<0.1 (<0.1–<0.1)	24.5 (19.3–30.2)	18.0 (13.4–22.8)	1.2 (0.2–3.0)	1.2 (0.2–3.0)	1.0 (0.4–1.7)	1.0 (0.4–1.7)	12.0 (7.8–16.9)	12.4 (8.1–16.9)	12.4 (5.5–18.9)	4.7 (0.0–9.8)	<0.1 (0.0–0.0)	<0.1 (0.0–0.0)	21.5 (5.1–38.7)	21.5 (5.1–38.7)
Ireland	<0.1 (<0.1–<0.1)	<0.1 (<0.1–<0.1)	26.5 (20.0–33.3)	25.0 (18.0–31.9)	2.7 (1.2–5.1)	2.7 (1.2–5.1)	1.0 (0.4–1.6)	1.0 (0.4–1.6)	7.8 (4.5–11.4)	7.7 (4.4–11.3)	16.6 (9.5–23.3)	9.8 (4.1–15.5)	<0.1 (0.0–0.0)	<0.1 (0.0–0.0)	25.7 (14.4–37.2)	25.7 (14.4–37.2)
Israel	<0.1 (<0.1–<0.1)	<0.1 (<0.1–0.1)	25.1 (19.4–30.7)	14.0 (9.9–18.8)	10.1 (6.1–15.3)	10.1 (6.1–15.3)	1.0 (0.4–1.7)	1.0 (0.4–1.7)	8.9 (5.1–12.7)	10.9 (6.4–15.6)	4.4 (0.0–9.3)	1.2 (0.0–3.8)	2.1 (0.8–3.9)	2.1 (0.8–3.9)	3.5 (<0.1–10.4)	3.5 (<0.1–10.4)
Italy	<0.1 (<0.1–0.1)	<0.1 (<0.1–0.1)	28.3 (21.7–34.9)	20.6 (14.9–26.6)	7.9 (4.7–12.2)	7.8 (4.6–12.1)	1.0 (0.4–1.7)	1.0 (0.4–1.7)	10.9 (6.7–15.3)	12.6 (7.7–17.6)	13.8 (6.5–20.4)	6.1 (1.8–10.5)	0.1 (<0.1–0.2)	0.1 (<0.1–0.2)	17.4 (13.1–21.6)	17.2 (13.0–21.4)

	Household air pollution		Smoking		Ambient particulate matter		Handwashing		Secondhand smoke		Alcohol use		High temperature		Low temperature	
	**M**	**F**	**M**	**F**	**M**	**F**	**M**	**F**	**M**	**F**	**M**	**F**	**M**	**F**	**M**	**F**
Luxembourg	<0.1 (<0.1–<0.1)	<0.1 (<0.1–<0.1)	29.3 (21.9–36.3)	23.4 (17.1–29.9)	4.2 (2.2–7.3)	4.2 (2.2–7.3)	1.0 (0.4–1.6)	1.0 (0.4–1.6)	10.2 (6.3–14.4)	10.9 (6.6–15.3)	18.4 (10.2–25.0)	6.3 (0.8–11.3)	<0.1 (<0.1–0.1)	<0.1 (<0.1–0.1)	23.1 (13.3–33.6)	23.1 (13.3–33.6)
Malta	<0.1 (<0.1–<0.1)	<0.1 (<0.1–0.1)	28.3 (22.2–34.1)	23.7 (18.2–29.4)	6.1 (3.4–9.9)	6.1 (3.4–9.9)	1.0 (0.4–1.7)	1.0 (0.4–1.7)	10.4 (6.5–14.8)	12.1 (7.6–16.8)	13.0 (5.9–19.9)	3.2 (0.0–7.5)	0.3 (<0.1–0.9)	0.3 (<0.1–0.9)	3.6 (<0.1–15.0)	3.6 (<0.1–15.0)
Monaco	<0.1 (<0.1–<0.1)	<0.1 (<0.1–<0.1)	30.4 (22.2–37.7)	23.8 (16.0–31.5)	5.3 (2.5–9.2)	5.3 (2.5–9.2)	1.0 (0.4–1.6)	1.0 (0.4–1.6)	10.2 (6.2–14.4)	11.4 (7.1–16.2)	9.0 (0.0–22.5)	3.8 (0.0–12.6)	<0.1 (<0.1–0.1)	<0.1 (<0.1–0.1)	15.4 (11.9–19.0)	15.4 (11.9–19.0)
Netherlands	<0.1 (<0.1–<0.1)	<0.1 (<0.1–<0.1)	28.3 (21.7–35.4)	22.6 (16.5–28.9)	5.5 (3.1–9.0)	5.5 (3.1–9.0)	1.0 (0.4–1.6)	1.0 (0.4–1.6)	8.9 (5.3–13.2)	9.4 (5.6–13.6)	12.3 (5.3–19.0)	6.1 (0.9–11.2)	<0.1 (<0.1–0.1)	<0.1 (<0.1–0.1)	23.6 (13.4–33.5)	23.6 (13.4–33.5)
Norway	<0.1 (<0.1–<0.1)	<0.1 (<0.1–<0.1)	25.1 (19.2–31.2)	18.0 (12.7–23.5)	1.8 (0.6–3.9)	1.8 (0.6–3.9)	1.0 (0.4–1.6)	1.0 (0.4–1.6)	8.8 (5.3–13.0)	9.2 (5.4–13.0)	10.8 (3.8–16.8)	4.2 (0.0–9.2)	<0.1 (<0.1–0.1)	<0.1 (<0.1–0.1)	19.0 (5.7–35.8)	18.9 (5.8–35.9)
Portugal	<0.1 (<0.1–0.1)	0.1 (<0.1–0.2)	32.3 (24.7–39.6)	19.0 (12.6–25.7)	3.0 (1.4–5.5)	3.0 (1.4–5.5)	1.1 (0.4–1.8)	1.1 (0.4–1.8)	7.9 (4.4–11.6)	10.5 (6.0–15.2)	18.7 (10.5–25.6)	5.7 (1.6–9.8)	0.2 (<0.1–0.3)	0.2 (<0.1–0.3)	15.3 (11.7–19.2)	15.3 (11.7–19.2)
San Marino	<0.1 (<0.1–<0.1)	<0.1 (<0.1–<0.1)	27.4 (20.9–34.4)	20.6 (14.7–27.5)	4.1 (1.5–7.9)	4.1 (1.5–7.9)	1.0 (0.4–1.7)	1.0 (0.4–1.7)	11.2 (7.2–16.1)	12.4 (8.0–16.9)	14.9 (0.0–24.6)	7.0 (0.0–14.6)	0.1 (<0.1–0.2)	0.1 (<0.1–0.2)	15.7 (12.5–19.0)	15.7 (12.5–19.0)
Spain	0.1 (<0.1–0.2)	0.1 (<0.1–0.3)	36.2 (28.5–43.9)	30.0 (22.3–37.7)	4.0 (2.1–6.9)	4.0 (2.1–6.9)	1.0 (0.4–1.7)	1.0 (0.4–1.7)	10.1 (6.0–14.3)	11.8 (7.1–16.6)	15.9 (8.1–22.7)	6.3 (1.5–11.3)	0.2 (<0.1–0.5)	0.2 (<0.1–0.5)	17.3 (13.3–21.5)	17.3 (13.3–21.5)
Sweden	<0.1 (<0.1–<0.1)	<0.1 (<0.1–<0.1)	18.2 (13.6–23.2)	19.5 (14.5–24.5)	1.2 (0.3–3.0)	1.2 (0.3–3.0)	1.0 (0.4–1.7)	1.0 (0.4–1.7)	9.5 (6.0–13.4)	8.7 (5.6–12.6)	10.2 (3.4–16.7)	5.9 (0.7–11.2)	<0.1 (<0.1–0.1)	<0.1 (<0.1–0.1)	18.5 (5.5–32.9)	18.5 (5.3–33.0)
Switzerland	<0.1 (<0.1–<0.1)	<0.1 (<0.1–<0.1)	29.1 (22.4–35.8)	22.6 (16.3–28.9)	4.1 (2.2–7.0)	4.1 (2.2–7.0)	1.0 (0.4–1.6)	1.0 (0.4–1.6)	9.0 (5.2–13.0)	9.8 (5.9–14.1)	13.4 (5.6–20.0)	8.4 (2.3–14.0)	<0.1 (<0.1–<0.1)	<0.1 (<0.1–<0.1)	21.1 (11.3–32.9)	21.1 (11.3–32.9)
United Kingdom	<0.1 (<0.1–<0.1)	<0.1 (<0.1–<0.1)	29.0 (21.6–36.2)	25.4 (18.4–32.4)	4.2 (2.2–7.2)	4.2 (2.2–7.2)	1.0 (0.4–1.7)	1.0 (0.4–1.7)	8.7 (5.0–12.6)	9.6 (5.8–13.8)	14.8 (7.2–21.1)	7.7 (2.2–13.1)	<0.1 (<0.1–<0.1)	<0.1 (<0.1–<0.1)	24.8 (15.8–34.9)	24.8 (15.9–34.8)
**Latin America and Caribbean**	**3.8 (1.9–6.6)**	**6.7 (3.7–10.4)**	**15.0 (10.2–19.8)**	**7.2 (4.7–9.8)**	**8.6 (5.5–12.8)**	**8.5 (5.5–12.4)**	**6.8 (2.8–10.8)**	**7.4 (3.1–11.6)**	**5.8 (3.2–8.9)**	**6.1 (3.2–9.2)**	**12.1 (6.3–17.2)**	**3.0 (0.7–5.3)**	**1.4 (<0.1–4.3)**	**1.4 (<0.1–4.2)**	**3.3 (1.6–5.1)**	**3.1 (1.4–4.8)**
Andean Latin America	3.4 (1.4–6.3)	4.9 (2.2–8.7)	7.8 (4.4–11.7)	2.7 (1.3–4.4)	13.6 (8.4–20.8)	13.2 (8.3–19.8)	7.7 (3.2–12.3)	7.8 (3.2–12.4)	3.1 (1.1–5.5)	3.3 (1.2–6.1)	11.4 (4.7–16.9)	2.2 (0.0–5.4)	0.4 (<0.1–1.3)	0.5 (<0.1–1.4)	9.1 (5.6–12.8)	9.1 (5.7–12.8)
Bolivia (Plurinational State of)	6.3 (2.7–12.1)	8.5 (4.0–14.7)	7.8 (4.6–11.5)	2.2 (0.9–3.8)	12.8 (7.4–20.2)	12.3 (7.3–19.3)	8.4 (3.5–13.2)	8.4 (3.5–13.2)	2.8 (1.0–5.4)	3.2 (1.2–6.0)	9.1 (3.2–15.0)	1.6 (0.0–4.6)	1.5 (0.5–2.7)	1.5 (0.5–2.7)	9.1 (5.9–12.6)	9.1 (5.9–12.6)
Ecuador	1.0 (0.3–2.3)	1.4 (0.5–3.2)	9.9 (5.1–14.8)	3.0 (1.5–4.8)	10.2 (5.4–16.5)	10.2 (5.5–16.4)	6.9 (2.8–11.0)	6.9 (2.8–11.0)	2.8 (1.0–5.3)	3.1 (1.1–5.9)	10.1 (4.9–14.9)	1.5 (0.0–3.4)	0.3 (<0.1–1.2)	0.3 (<0.1–1.2)	5.8 (3.6–8.1)	5.8 (3.6–8.1)
Peru	3.5 (1.4–6.8)	4.8 (2.1–8.9)	7.0 (3.9–10.6)	2.8 (1.4–4.6)	15.1 (9.3–23.0)	14.8 (9.3–22.3)	7.9 (3.3–12.5)	7.9 (3.3–12.5)	3.2 (1.2–5.9)	3.3 (1.3–6.1)	12.5 (5.1–19.1)	2.6 (0.0–6.7)	0.2 (<0.1–0.9)	0.2 (<0.1–0.9)	10.4 (6.0–15.2)	10.4 (6.0–15.2)
Caribbean	14.3 (7.4–23.2)	25.2 (15.7–35.5)	14.2 (9.9–19.0)	4.8 (3.1–6.9)	7.6 (3.6–13.6)	6.4 (3.1–11.6)	14.3 (6.4–22.3)	16.1 (7.1–24.4)	5.6 (3.0–8.4)	5.4 (2.8–8.1)	11.8 (6.0–16.9)	2.4 (0.5–4.5)	0.9 (<0.1–2.9)	0.9 (<0.1–2.9)	<0.1 (<0.1–1.2)	<0.1 (<0.1–1.3)
Antigua and Barbuda	0.1 (<0.1–0.3)	0.2 (<0.1–0.4)	12.1 (8.3–16.2)	5.7 (3.5–8.8)	8.7 (2.7–18.4)	8.7 (2.7–18.4)	7.5 (3.1–11.9)	7.5 (3.1–11.9)	6.6 (3.9–9.7)	6.9 (4.0–10.2)	11.6 (5.1–17.0)	2.9 (0.1–5.9)	0.4 (<0.1–2.3)	0.4 (<0.1–2.3)	0.3 (<0.1–1.7)	0.3 (<0.1–1.7)

	Household air pollution		Smoking		Ambient particulate matter		Handwashing		Secondhand smoke		Alcohol use		High temperature		Low temperature	
	**M**	**F**	**M**	**F**	**M**	**F**	**M**	**F**	**M**	**F**	**M**	**F**	**M**	**F**	**M**	**F**
Bahamas	0.1 (<0.1–0.4)	0.2 (<0.1–0.5)	12.5 (8.5–16.9)	4.2 (2.5–6.6)	7.6 (1.7–16.4)	7.6 (1.7–16.4)	6.3 (2.6–10.2)	6.3 (2.6–10.2)	6.8 (4.0–9.8)	8.1 (4.8–11.6)	9.6 (0.6–17.5)	2.2 (0.0–6.0)	1.6 (<0.1–4.5)	1.6 (<0.1–4.5)	0.3 (<0.1–2.0)	0.3 (<0.1–2.0)
Barbados	<0.1 (<0.1–<0.1)	<0.1 (<0.1–<0.1)	16.8 (11.9–22.3)	5.7 (3.7–8.2)	10.8 (3.8–21.4)	10.8 (3.8–21.4)	7.1 (2.9–11.4)	7.1 (2.9–11.4)	5.6 (3.1–8.3)	5.6 (3.2–8.4)	16.6 (9.3–22.5)	2.3 (0.0–5.3)	1.2 (<0.1–3.9)	1.2 (<0.1–3.9)	0.4 (<0.1–2.0)	0.4 (<0.1–2.0)
Belize	2.9 (1.1–6.0)	4.0 (1.6–7.8)	13.6 (9.0–18.4)	3.0 (1.7–4.6)	10.6 (3.3–20.8)	10.4 (3.5–20.5)	9.5 (4.0–15.0)	9.5 (4.0–15.0)	5.9 (3.2–8.9)	8.0 (4.4–11.9)	12.6 (6.0–18.2)	2.2 (0.3–4.1)	1.7 (<0.1–4.2)	1.7 (<0.1–4.2)	0.1 (<0.1–1.3)	0.1 (<0.1–1.3)
Cuba	0.2 (0.1–0.6)	0.4 (0.1–0.9)	27.7 (20.9–34.4)	15.6 (10.7–21.2)	8.8 (3.7–16.9)	8.8 (3.7–16.9)	6.0 (2.5–9.6)	6.0 (2.5–9.6)	8.3 (4.8–12.1)	10.5 (6.4–14.9)	11.4 (5.0–16.8)	2.7 (0.1–5.6)	1.5 (<0.1–4.0)	1.5 (<0.1–4.0)	0.2 (<0.1–1.6)	0.2 (<0.1–1.6)
Dominica	1.0 (0.3–2.3)	1.4 (0.4–3.3)	14.2 (9.5–19.6)	4.5 (2.5–6.9)	9.4 (3.2–19.0)	9.4 (3.3–18.9)	8.3 (3.5–13.2)	8.3 (3.5–13.2)	5.1 (2.8–7.8)	6.0 (3.2–9.1)	13.2 (6.7–19.2)	3.7 (0.6–7.1)	1.1 (<0.1–3.5)	1.1 (<0.1–3.5)	0.3 (<0.1–3.2)	0.3 (<0.1–3.2)
Dominican Republic	1.9 (0.7–4.1)	2.6 (1.0–5.6)	12.0 (8.5–16.1)	6.1 (3.9–8.6)	8.9 (3.3–17.8)	8.8 (3.4–17.7)	16.0 (7.1–24.3)	16.0 (7.1–24.3)	6.8 (4.0–9.9)	6.3 (3.6–9.2)	10.5 (4.5–16.1)	4.2 (0.9–7.4)	0.6 (<0.1–2.2)	0.6 (<0.1–2.2)	<0.1 (<0.1–1.7)	<0.1 (<0.1–1.7)
Grenada	0.4 (0.1–0.9)	0.6 (0.2–1.3)	13.1 (8.5–17.9)	3.9 (2.1–6.1)	10.8 (3.4–21.5)	10.8 (3.5–21.4)	7.6 (3.2–12.2)	7.6 (3.2–12.2)	4.7 (2.4–7.3)	5.7 (2.8–9.0)	15.2 (8.1–21.1)	4.7 (1.4–8.2)	1.4 (<0.1–4.2)	1.4 (<0.1–4.2)	0.3 (<0.1–1.8)	0.3 (<0.1–1.8)
Guyana	1.1 (0.4–2.3)	1.6 (0.6–3.2)	17.1 (11.6–22.6)	2.9 (1.6–4.6)	10.1 (3.2–20.5)	10.0 (3.2–20.4)	14.1 (6.1–21.9)	14.1 (6.1–21.9)	5.0 (2.6–7.7)	7.7 (4.0–11.9)	14.8 (7.8–21.0)	0.7 (0.0–2.4)	0.9 (<0.1–2.8)	0.9 (<0.1–2.8)	<0.1 (<0.1–1.3)	<0.1 (<0.1–1.3)
Haiti	34.0 (20.8–48.1)	40.7 (27.5–53.2)	8.2 (4.9–11.8)	2.3 (1.2–3.8)	6.2 (2.6–12.0)	5.2 (2.2–10.0)	19.5 (8.7–29.2)	19.5 (8.7–29.2)	3.5 (1.6–5.8)	3.6 (1.7–6.0)	12.4 (6.5–17.7)	1.9 (0.4–3.6)	0.8 (<0.1–3.0)	0.8 (<0.1–3.0)	<0.1 (<0.1–1.3)	<0.1 (<0.1–1.3)
Jamaica	1.4 (0.4–3.1)	1.9 (0.6–4.4)	19.3 (14.0–24.5)	5.6 (3.4–8.1)	7.7 (3.9–13.2)	7.7 (4.0–13.0)	11.2 (4.8–17.4)	11.2 (4.8–17.4)	7.0 (4.0–10.3)	8.9 (5.1–12.7)	8.5 (2.9–13.8)	1.6 (0.0–4.1)	1.2 (<0.1–4.3)	1.2 (<0.1–4.3)	0.1 (<0.1–1.1)	0.1 (<0.1–1.1)
Saint Kitts and Nevis	0.2 (<0.1–0.7)	0.3 (0.1–1.0)	12.0 (7.5–16.7)	2.5 (1.3–4.2)	3.5 (1.3–6.7)	3.5 (1.4–6.7)	7.6 (3.2–12.1)	7.6 (3.2–12.1)	6.4 (3.5–9.8)	7.5 (4.3–11.2)	5.5 (0.0–17.0)	1.3 (0.0–5.8)	1.0 (<0.1–3.9)	1.0 (<0.1–3.9)	0.4 (<0.1–2.3)	0.4 (<0.1–2.3)
Saint Lucia	0.5 (0.2–1.1)	0.7 (0.2–1.6)	17.0 (11.7–22.7)	5.0 (2.8–7.6)	10.7 (3.7–21.1)	10.7 (3.8–21.0)	5.7 (2.3–9.2)	5.7 (2.3–9.2)	5.5 (2.9–8.4)	6.1 (3.3–9.1)	15.6 (8.4–21.8)	4.6 (0.5–8.9)	1.7 (<0.1–4.8)	1.7 (<0.1–4.8)	0.3 (<0.1–1.9)	0.3 (<0.1–1.9)
Saint Vincent and the Grenadines	0.6 (0.2–1.4)	0.9 (0.3–2.0)	15.8 (10.3–21.6)	3.5 (1.9–5.4)	10.6 (3.3–21.3)	10.6 (3.4–21.1)	9.1 (3.8–14.3)	9.1 (3.8–14.3)	5.1 (2.7–7.9)	6.7 (3.5–10.0)	14.3 (7.5–19.9)	4.3 (1.0–7.6)	1.3 (<0.1–4.0)	1.3 (<0.1–4.0)	0.4 (<0.1–2.0)	0.4 (<0.1–2.0)
Suriname	1.6 (0.5–3.7)	2.3 (0.8–4.9)	24.9 (18.3–31.8)	7.2 (4.3–10.8)	10.7 (4.1–20.6)	10.6 (4.2–20.4)	9.3 (3.9–14.9)	9.3 (3.9–14.9)	7.2 (4.0–10.7)	11.6 (6.7–16.9)	11.4 (5.0–17.0)	2.7 (0.0–5.5)	1.0 (<0.1–3.0)	1.0 (<0.1–3.0)	0.3 (<0.1–1.6)	0.3 (<0.1–1.6)
Trinidad and Tobago	<0.1 (<0.1–<0.1)	<0.1 (<0.1–<0.1)	19.4 (12.9–26.1)	8.4 (5.0–12.4)	11.0 (3.1–22.8)	11.0 (3.1–22.8)	6.8 (2.8–10.9)	6.8 (2.8–10.9)	5.9 (3.1–9.2)	8.6 (4.6–13.1)	11.5 (5.2–16.7)	3.2 (0.0–6.7)	0.4 (<0.1–2.6)	0.4 (<0.1–2.6)	0.2 (<0.1–1.2)	0.2 (<0.1–1.2)
Central Latin America	4.7 (2.2–8.1)	6.9 (3.6–11.2)	13.6 (8.1–19.1)	4.6 (2.5–6.9)	10.7 (6.9–15.7)	10.5 (6.9–15.3)	5.9 (2.4–9.4)	6.0 (2.5–9.7)	3.4 (1.3–6.3)	4.5 (1.8–8.0)	12.0 (6.4–16.8)	2.3 (0.4–4.3)	1.2 (0.2–2.9)	1.2 (0.1–3.2)	4.8 (2.6–7.1)	4.1 (2.2–6.2)
Colombia	1.8 (0.7–4.0)	2.5 (0.9–5.2)	11.7 (7.3–16.3)	5.4 (2.9–8.1)	11.2 (6.4–17.6)	11.1 (6.5–17.4)	6.1 (2.5–9.9)	6.1 (2.5–9.9)	4.8 (2.3–7.9)	5.6 (2.7–9.1)	11.9 (5.9–16.9)	1.9 (0.0–3.9)	1.0 (<0.1–3.4)	1.0 (<0.1–3.4)	3.4 (1.8–5.1)	3.4 (1.8–5.1)
Costa Rica	0.8 (0.3–2.0)	1.2 (0.4–2.7)	17.1 (11.9–22.5)	8.0 (4.9–11.6)	8.8 (5.2–13.8)	8.8 (5.2–13.7)	6.8 (2.8–10.9)	6.8 (2.8–10.9)	6.7 (3.8–9.9)	8.5 (4.8–12.5)	10.7 (4.6–15.8)	1.8 (0.0–4.4)	0.3 (<0.1–2.2)	0.3 (<0.1–2.2)	0.6 (<0.1–2.7)	0.6 (<0.1–2.7)

	Household air pollution		Smoking		Ambient particulate matter		Handwashing		Secondhand smoke		Alcohol use		High temperature		Low temperature	
	**M**	**F**	**M**	**F**	**M**	**F**	**M**	**F**	**M**	**F**	**M**	**F**	**M**	**F**	**M**	**F**
El Salvador	3.1 (1.2–5.9)	4.3 (1.9–7.6)	14.4 (9.4–19.7)	3.7 (2.3–5.5)	11.2 (5.9–18.1)	11.0 (6.0–17.7)	9.0 (3.8–14.4)	9.0 (3.8–14.4)	3.9 (2.0–6.2)	4.8 (2.5–7.3)	8.7 (3.7–13.0)	1.4 (0.0–3.0)	1.4 (<0.1–6.7)	1.4 (<0.1–6.7)	<0.1 (<0.1–0.6)	<0.1 (<0.1–0.6)
Guatemala	14.5 (6.9–24.6)	18.6 (9.8–30.0)	11.5 (6.5–16.6)	2.5 (1.3–4.0)	11.9 (7.3–17.7)	11.0 (6.8–16.2)	7.6 (3.2–12.1)	7.6 (3.2–12.1)	3.1 (1.2–5.5)	4.1 (1.6–7.4)	7.8 (3.6–12.1)	0.9 (0.0–2.1)	1.7 (0.5–3.5)	1.7 (0.5–3.5)	1.5 (<0.1–3.2)	1.5 (<0.1–3.2)
Honduras	18.1 (9.7–29.1)	22.9 (13.1–34.0)	17.8 (12.4–23.4)	3.3 (1.9–5.3)	9.5 (5.9–14.3)	8.5 (5.3–12.8)	5.4 (2.2–8.6)	5.4 (2.2–8.6)	5.4 (2.8–8.3)	8.0 (4.3–12.0)	8.6 (4.4–12.2)	0.7 (0.1–1.4)	0.7 (<0.1–2.6)	0.7 (<0.1–2.6)	<0.1 (<0.1–2.4)	<0.1 (<0.1–2.4)
Mexico	2.5 (1.1–4.7)	4.0 (1.9–7.1)	14.1 (7.8–20.4)	4.8 (2.2–7.9)	10.3 (6.3–15.5)	10.1 (6.3–14.9)	5.0 (2.0–8.1)	5.1 (2.1–8.2)	2.7 (0.8–5.6)	3.6 (1.0–7.5)	13.5 (7.3–19.0)	3.0 (0.5–5.8)	1.2 (0.4–1.9)	1.1 (0.3–2.0)	7.7 (4.9–10.8)	7.4 (4.6–10.4)
Nicaragua	14.3 (6.6–24.4)	18.0 (9.4–29.1)	12.1 (7.7–16.8)	3.6 (2.0–5.5)	9.1 (5.3–14.6)	8.5 (4.9–13.5)	8.1 (3.4–12.8)	8.1 (3.4–12.8)	5.6 (2.6–8.8)	6.5 (3.2–10.4)	10.4 (5.4–14.5)	1.1 (0.2–2.1)	0.8 (<0.1–4.9)	0.8 (<0.1–4.9)	<0.1 (<0.1–1.1)	<0.1 (<0.1–1.1)
Panama	1.3 (0.4–2.9)	1.9 (0.6–3.9)	9.5 (6.4–12.9)	3.0 (1.8–4.6)	6.5 (3.3–10.9)	6.5 (3.3–10.9)	6.1 (2.5–9.8)	6.1 (2.5–9.8)	5.0 (2.9–7.5)	5.6 (3.1–8.3)	14.4 (7.8–19.6)	3.4 (0.9–5.8)	0.6 (<0.1–2.3)	0.6 (<0.1–2.3)	<0.1 (<0.1–1.0)	<0.1 (<0.1–1.0)
Venezuela (Bolivarian Republic of)	0.1 (<0.1–0.2)	0.1 (<0.1–0.2)	16.2 (10.1–22.4)	6.6 (3.8–10.0)	11.1 (5.9–18.6)	11.1 (5.9–18.6)	5.7 (2.4–9.2)	5.7 (2.4–9.2)	5.1 (2.2–8.4)	6.3 (2.9–10.3)	13.3 (7.3–18.8)	2.7 (0.5–5.3)	1.2 (<0.1–9.4)	1.2 (<0.1–9.4)	0.1 (<0.1–1.7)	0.1 (<0.1–1.7)
Tropical Latin America	1.6 (0.6–3.4)	2.5 (0.9–4.8)	18.3 (13.6–23.5)	11.5 (8.0–15.4)	5.7 (3.2–9.2)	5.6 (3.3–9.1)	6.1 (2.5–9.9)	6.2 (2.5–10.0)	8.6 (5.1–12.4)	8.6 (5.1–12.3)	12.5 (6.5–17.8)	4.2 (1.4–6.9)	1.9 (<0.1–6.8)	1.8 (<0.1–7.2)	1.0 (<0.1–3.0)	1.0 (<0.1–2.9)
Brazil	1.5 (0.5–3.3)	2.3 (0.9–4.6)	18.3 (13.5–23.6)	11.6 (8.1–15.5)	5.7 (3.2–9.2)	5.6 (3.3–9.1)	6.1 (2.5–9.8)	6.2 (2.5–10.0)	8.6 (5.1–12.4)	8.6 (5.1–12.3)	12.5 (6.4–17.7)	4.2 (1.4–7.0)	1.8 (<0.1–6.8)	1.8 (<0.1–7.2)	1.0 (<0.1–2.9)	1.0 (<0.1–2.9)
Paraguay	6.4 (2.4–12.9)	8.8 (3.8–16.1)	18.6 (13.0–24.6)	5.5 (3.2–8.4)	6.3 (3.5–10.6)	6.1 (3.6–10.3)	7.9 (3.3–12.7)	7.9 (3.3–12.7)	8.5 (4.8–12.3)	9.3 (5.3–13.5)	15.7 (8.3–21.9)	2.3 (0.0–5.5)	5.2 (2.9–7.6)	5.2 (2.9–7.6)	0.9 (<0.1–5.0)	0.9 (<0.1–5.0)
**North Africa and Middle East**	**4.9 (2.8–7.5)**	**6.6 (4.1–9.8)**	**24.0 (19.1–28.9)**	**2.9 (1.9–4.0)**	**21.2 (14.9–28.3)**	**20.4 (14.4–27.4)**	**7.0 (3.0–11.1)**	**7.4 (3.2–11.7)**	**9.1 (5.7–12.7)**	**16.9 (11.0–22.9)**	**1.2 (0.4–2.0)**	**0.2 (0.0–0.4)**	**6.1 (3.7–8.6)**	**6.4 (4.0–8.8)**	**6.5 (2.4–10.7)**	**6.2 (2.0–10.4)**
Afghanistan	33.4 (20.6–45.2)	37.4 (25.1–48.6)	13.9 (9.9–18.4)	1.9 (1.1–3.1)	12.3 (5.9–20.6)	10.1 (4.6–17.8)	15.7 (6.8–23.8)	15.7 (6.8–23.8)	8.1 (4.8–11.7)	12.3 (7.4–17.5)	0.1 (0.0–0.5)	<0.1 (0.0–0.1)	3.1 (1.9–4.2)	3.1 (1.9–4.2)	13.7 (9.0–19.6)	13.7 (9.0–19.6)
Algeria	<0.1 (<0.1–0.1)	0.1 (<0.1–0.1)	22.8 (17.6–28.5)	1.7 (0.9–2.8)	16.6 (9.1–25.5)	16.6 (9.1–25.5)	4.9 (2.0–7.9)	4.9 (2.0–7.9)	11.1 (7.0–15.4)	18.8 (12.4–25.4)	2.1 (0.5–3.8)	0.2 (0.0–0.5)	9.2 (6.2–12.2)	9.2 (6.2–12.2)	4.3 (<0.1–10.1)	4.3 (<0.1–10.1)
Bahrain	0.1 (<0.1–0.2)	0.1 (<0.1–0.2)	21.6 (16.2–27.3)	3.1 (1.8–5.0)	26.5 (17.9–36.4)	26.5 (17.9–36.4)	4.6 (1.9–7.4)	4.6 (1.9–7.4)	9.6 (6.0–13.6)	12.5 (8.1–17.2)	3.0 (0.9–5.1)	0.3 (0.0–0.7)	11.6 (5.8–16.6)	11.6 (5.8–16.6)	2.0 (<0.1–10.1)	2.0 (<0.1–10.1)
Egypt	<0.1 (<0.1–<0.1)	<0.1 (<0.1–0.1)	25.5 (19.9–31.2)	0.6 (0.3–1.0)	28.9 (19.3–40.0)	28.9 (19.3–40.0)	3.8 (1.5–6.3)	3.8 (1.5–6.3)	8.7 (5.2–12.6)	20.0 (12.8–26.7)	0.4 (0.0–1.2)	<0.1 (0.0–0.1)	7.7 (4.8–10.9)	7.7 (4.8–10.9)	2.7 (<0.1–9.1)	2.7 (<0.1–9.1)
Iran (Islamic Republic of)	<0.1 (<0.1–0.1)	0.1 (<0.1–0.2)	20.2 (15.3–25.0)	3.0 (1.7–4.4)	19.1 (12.5–26.9)	19.1 (12.5–26.8)	4.3 (1.7–7.1)	4.3 (1.7–7.1)	8.2 (4.9–11.6)	15.5 (9.6–21.3)	2.1 (1.1–3.2)	0.3 (0.1–0.5)	3.5 (2.3–4.7)	3.6 (2.4–4.8)	12.8 (9.6–16.0)	12.6 (9.3–15.7)
Iraq	<0.1 (<0.1–0.1)	0.1 (<0.1–0.2)	25.0 (19.7–30.6)	1.6 (0.9–2.5)	22.7 (14.6–32.4)	22.7 (14.6–32.4)	2.7 (1.1–4.5)	2.7 (1.1–4.5)	10.6 (6.6–15.1)	17.7 (11.5–24.1)	1.0 (0.0–2.5)	0.1 (0.0–0.3)	10.8 (7.5–13.8)	10.8 (7.5–13.8)	4.7 (<0.1–10.6)	4.7 (<0.1–10.6)
Jordan	<0.1 (<0.1–<0.1)	<0.1 (<0.1–<0.1)	32.3 (26.2–38.7)	6.7 (4.3–9.6)	15.7 (9.8–23.4)	15.7 (9.8–23.4)	4.2 (1.7–6.9)	4.2 (1.7–6.9)	8.3 (5.0–11.9)	18.3 (11.5–25.0)	1.4 (0.0–3.2)	0.1 (0.0–0.4)	3.2 (1.7–4.9)	3.2 (1.7–4.9)	6.0 (<0.1–12.1)	6.0 (<0.1–12.1)

	Household air pollution		Smoking		Ambient particulate matter		Handwashing		Secondhand smoke		Alcohol use		High temperature		Low temperature	
	**M**	**F**	**M**	**F**	**M**	**F**	**M**	**F**	**M**	**F**	**M**	**F**	**M**	**F**	**M**	**F**
Kuwait	<0.1 (<0.1–<0.1)	<0.1 (<0.1–<0.1)	31.4 (25.4–37.5)	3.6 (2.2–5.5)	27.1 (18.5–36.9)	27.1 (18.5–36.9)	3.7 (1.5–6.0)	3.7 (1.5–6.0)	9.5 (5.9–13.4)	20.4 (13.6–27.4)	0.1 (0.0–0.5)	<0.1 (0.0–0.1)	13.1 (7.6–18.0)	13.1 (7.6–18.0)	3.1 (<0.1–15.6)	3.1 (<0.1–15.6)
Lebanon	<0.1 (<0.1–0.1)	0.1 (<0.1–0.1)	25.6 (19.9–31.2)	13.5 (9.5–18.5)	14.9 (9.0–22.6)	14.9 (9.0–22.6)	5.0 (2.1–8.0)	5.0 (2.1–8.0)	13.8 (9.0–19.2)	18.6 (12.2–25.0)	2.4 (0.6–4.5)	0.2 (0.0–0.7)	0.1 (<0.1–0.2)	0.1 (<0.1–0.2)	14.0 (10.4–17.5)	14.0 (10.4–17.5)
Libya	<0.1 (<0.1–0.1)	0.1 (<0.1–0.1)	29.8 (23.7–36.0)	0.9 (0.5–1.5)	19.0 (11.0–29.3)	19.0 (11.0–29.3)	4.5 (1.9–7.4)	4.5 (1.9–7.4)	10.9 (6.9–15.2)	20.6 (13.9–27.4)	1.2 (0.1–1.9)	0.2 (<0.1–0.3)	8.2 (4.7–12.0)	8.2 (4.7–12.0)	2.8 (<0.1–9.8)	2.8 (<0.1–9.8)
Morocco	0.9 (0.3–1.9)	1.3 (0.5–2.7)	20.6 (15.4–26.6)	1.2 (0.7–1.9)	17.4 (10.8–25.1)	17.3 (10.7–24.9)	7.6 (3.2–12.2)	7.6 (3.2–12.2)	7.8 (4.5–11.2)	12.6 (7.7–17.6)	1.5 (0.1–2.9)	<0.1 (0.0–<0.1)	2.5 (1.6–3.4)	2.5 (1.6–3.4)	8.2 (4.8–11.8)	8.2 (4.8–11.8)
Oman	0.1 (<0.1–0.2)	0.1 (<0.1–0.3)	12.9 (9.3–17.0)	1.1 (0.6–1.7)	21.4 (12.7–31.3)	21.4 (12.7–31.3)	5.0 (2.0–8.2)	5.0 (2.0–8.2)	7.7 (4.7–11.2)	10.8 (7.0–15.2)	0.6 (0.0–1.6)	<0.1 (0.0–0.2)	6.6 (<0.1–22.3)	6.6 (<0.1–22.3)	1.3 (<0.1–13.3)	1.3 (<0.1–13.3)
Palestine	0.2 (0.1–0.5)	0.3 (0.1–0.6)	20.6 (15.4–26.0)	1.9 (1.1–3.0)	15.9 (9.5–24.7)	15.9 (9.5–24.7)	7.1 (3.0–11.4)	7.1 (3.0–11.4)	10.5 (6.6–14.7)	21.1 (13.8–28.4)	4.1 (2.3–6.0)	0.7 (0.3–1.2)	2.2 (0.9–4.0)	2.2 (0.9–4.0)	3.9 (<0.1–10.5)	3.9 (<0.1–10.5)
Qatar	<0.1 (<0.1–<0.1)	<0.1 (<0.1–<0.1)	18.5 (14.2–23.6)	1.4 (0.8–2.2)	31.1 (22.0–41.1)	31.1 (22.0–41.1)	3.8 (1.5–6.3)	3.8 (1.5–6.3)	9.3 (5.7–13.0)	17.3 (11.3–23.3)	1.2 (0.0–2.8)	0.1 (0.0–0.4)	10.6 (3.7–19.5)	10.6 (3.7–19.5)	1.8 (<0.1–15.2)	1.8 (<0.1–15.2)
Saudi Arabia	<0.1 (<0.1–0.1)	0.1 (<0.1–0.2)	27.2 (20.7–34.0)	1.7 (0.9–2.8)	27.0 (18.4–37.3)	27.0 (18.4–37.3)	4.2 (1.7–6.7)	4.2 (1.7–6.7)	8.9 (5.7–12.6)	18.7 (12.8–25.0)	0.5 (0.0–1.5)	0.1 (0.0–0.3)	9.7 (5.9–14.1)	9.7 (5.9–14.1)	2.4 (<0.1–8.8)	2.4 (<0.1–8.8)
Sudan	12.3 (6.2–21.1)	15.7 (8.6–24.8)	19.4 (14.3–25.3)	1.9 (1.1–3.0)	20.6 (11.9–31.4)	19.3 (11.3–29.6)	17.7 (7.9–26.7)	17.7 (7.9–26.7)	12.1 (8.1–16.7)	15.7 (10.9–21.2)	<0.1 (0.0–0.2)	<0.1 (0.0–<0.1)	8.3 (3.6–15.7)	8.3 (3.6–15.7)	1.5 (<0.1–7.2)	1.5 (<0.1–7.2)
Syrian Arab Republic	<0.1 (<0.1–0.1)	<0.1 (<0.1–0.1)	23.1 (17.8–29.1)	3.4 (1.8–5.5)	15.8 (9.6–23.6)	15.8 (9.6–23.6)	4.9 (2.0–7.9)	4.9 (2.0–7.9)	10.4 (6.4–14.5)	17.1 (11.0–23.3)	1.5 (0.5–2.5)	0.2 (<0.1–0.3)	4.4 (2.5–6.3)	4.4 (2.5–6.3)	7.7 (2.5–12.6)	7.7 (2.5–12.6)
Tunisia	<0.1 (<0.1–0.1)	0.1 (<0.1–0.1)	33.2 (26.8–39.5)	2.1 (1.2–3.2)	15.5 (8.9–24.1)	15.5 (8.9–24.1)	5.7 (2.4–9.2)	5.7 (2.4–9.2)	9.0 (5.5–12.8)	18.5 (11.9–24.8)	6.2 (3.2–9.2)	1.5 (0.3–2.8)	4.5 (2.3–6.7)	4.5 (2.3–6.7)	5.1 (<0.1–11.3)	5.1 (<0.1–11.3)
Turkey	0.1 (<0.1–0.2)	0.1 (<0.1–0.2)	33.8 (27.6–40.5)	14.4 (10.0–19.1)	13.5 (8.4–20.4)	13.5 (8.4–20.3)	5.8 (2.4–9.3)	5.8 (2.4–9.3)	7.4 (4.5–10.7)	15.3 (9.5–21.2)	3.6 (1.3–5.8)	1.0 (0.0–2.3)	0.4 (0.1–0.7)	0.4 (0.1–0.7)	18.3 (13.7–23.4)	18.3 (13.7–23.4)
United Arab Emirates	<0.1 (<0.1–<0.1)	<0.1 (<0.1–<0.1)	25.8 (18.9–32.8)	4.6 (2.7–6.9)	21.1 (13.2–30.8)	21.1 (13.2–30.8)	3.8 (1.5–6.2)	3.8 (1.5–6.2)	10.0 (6.1–14.2)	16.8 (11.1–22.9)	2.0 (0.0–4.3)	0.3 (0.0–1.1)	8.4 (<0.1–25.0)	8.4 (<0.1–25.0)	1.9 (<0.1–18.2)	1.9 (<0.1–18.2)
Yemen	12.6 (6.2–22.1)	16.0 (8.6–25.1)	24.8 (19.1–30.8)	6.5 (4.2–9.4)	17.1 (9.2–27.3)	15.6 (8.1–25.0)	13.5 (5.8–20.7)	13.5 (5.8–20.7)	10.5 (6.6–14.7)	16.1 (10.4–22.0)	1.4 (0.4–2.5)	0.2 (<0.1–0.4)	5.5 (1.6–12.3)	5.5 (1.6–12.3)	0.9 (<0.1–5.1)	0.9 (<0.1–5.1)
**South Asia**	**16.5 (9.5–25.2)**	**20.9 (13.3–30.0)**	**15.6 (11.3–19.9)**	**1.6 (0.9–2.5)**	**24.4 (18.0–30.9)**	**23.2 (17.1–29.6)**	**11.9 (5.2–18.4)**	**12.3 (5.3–18.9)**	**6.4 (3.5–9.8)**	**11.5 (6.4–16.7)**	**5.7 (2.7–8.7)**	**0.3 (<0.1–0.6)**	**7.4 (4.2–11.8)**	**7.4 (4.1–11.3)**	**2.6 (<0.1–7.6)**	**2.4 (<0.1–7.3)**
Bangladesh	22.5 (13.0–34.3)	27.7 (17.5–39.2)	20.2 (13.4–26.6)	0.7 (0.3–1.3)	19.6 (13.7–25.9)	17.6 (11.7–23.6)	18.1 (8.1–27.1)	18.1 (8.1–27.1)	4.7 (2.3–7.6)	12.1 (6.2–18.8)	0.6 (0.0–1.6)	0.1 (0.0–0.4)	5.9 (2.7–9.8)	5.9 (2.7–9.8)	1.3 (<0.1–4.8)	1.3 (<0.1–4.8)
Bhutan	17.1 (8.8–28.5)	21.3 (12.6–32.5)	8.6 (5.7–11.7)	2.8 (1.6–4.5)	14.9 (9.9–21.0)	13.4 (8.7–19.3)	6.1 (2.5–9.8)	6.1 (2.5–9.8)	6.3 (3.5–9.3)	7.3 (4.1–11.1)	1.7 (0.0–4.4)	0.1 (0.0–0.4)	<0.1 (<0.1–0.1)	<0.1 (<0.1–0.1)	18.6 (11.8–26.9)	18.6 (11.8–26.9)
India	15.6 (8.9–23.9)	20.1 (12.8–28.6)	15.6 (11.3–20.0)	1.6 (0.9–2.5)	25.6 (19.1–32.1)	24.1 (17.8–30.4)	11.4 (4.9–17.5)	11.8 (5.1–18.2)	6.3 (3.4–9.7)	11.6 (6.6–16.8)	6.9 (3.3–10.7)	0.3 (<0.1–0.6)	7.3 (3.9–11.7)	7.5 (4.1–11.7)	2.1 (<0.1–6.8)	2.1 (<0.1–7.0)

	Household air pollution		Smoking		Ambient particulate matter		Handwashing		Secondhand smoke		Alcohol use		High temperature		Low temperature	
	M	F	M	F	M	F	M	F	M	F	M	F	M	F	M	F
Nepal	23.7 (13.9–35.6)	28.5 (17.8–39.5)	11.7 (6.5–17.1)	5.5 (2.8–8.5)	22.2 (14.4–30.6)	19.6 (12.0–27.9)	11.3 (4.9–17.8)	11.3 (4.9–17.8)	5.8 (2.6–9.9)	7.6 (3.4–12.8)	3.6 (0.0–8.3)	1.1 (0.0–3.0)	1.4 (0.7–2.2)	1.4 (0.7–2.2)	13.0 (7.6–19.0)	13.0 (7.6–19.0)
Pakistan	18.4 (10.6–28.1)	22.5 (13.7–32.3)	13.9 (10.2–18.0)	2.3 (1.3–3.5)	20.4 (14.1–27.5)	18.6 (12.6–25.6)	12.4 (5.2–19.2)	12.4 (5.3–19.2)	7.5 (4.0–11.2)	11.1 (6.1–16.2)	1.4 (0.3–2.4)	0.1 (<0.1–0.2)	9.2 (5.6–15.9)	9.2 (5.5–16.0)	4.9 (<0.1–12.8)	5.0 (<0.1–12.7)
**Southeast Asia, East Asia, and Oceania**	**9.4 (4.8–15.3)**	**13.5 (7.7–20.4)**	**28.3 (21.9–34.2)**	**3.1 (1.9–4.5)**	**12.7 (8.6–17.5)**	**11.6 (7.9–15.9)**	**6.1 (2.5–9.8)**	**6.4 (2.6–10.2)**	**6.3 (3.8–9.2)**	**16.2 (9.8–22.6)**	**10.2 (5.0–14.4)**	**1.4 (0.3–2.4)**	**1.6 (<0.1–7.5)**	**1.6 (<0.1–8.9)**	**3.7 (1.8–5.5)**	**3.3 (1.6–5.0)**
East Asia	5.7 (2.6–10.3)	8.5 (4.3–14.4)	34.0 (27.9–39.7)	2.8 (1.8–3.9)	19.4 (13.4–26.1)	18.9 (13.2–25.1)	3.8 (1.5–6.2)	3.8 (1.6–6.2)	8.0 (5.1–11.3)	21.6 (14.2–28.8)	11.9 (6.0–17.0)	0.9 (0.2–1.8)	0.7 (<0.1–1.3)	0.6 (<0.1–1.2)	12.1 (7.3–16.5)	12.8 (7.7–17.5)
China	5.2 (2.2–9.7)	7.3 (3.5–12.9)	34.2 (28.2–39.9)	2.5 (1.6–3.6)	20.0 (13.8–26.8)	19.5 (13.7–26.1)	4.0 (1.6–6.6)	4.0 (1.6–6.6)	8.1 (5.2–11.5)	21.9 (14.6–29.1)	12.2 (6.2–17.5)	1.0 (0.2–1.9)	0.7 (<0.1–1.3)	0.7 (<0.1–1.2)	12.1 (7.1–16.4)	12.6 (7.5–17.2)
Democratic People's Republic of Korea	20.3 (10.8–32.1)	25.7 (15.8–37.6)	27.8 (21.4–34.4)	2.9 (1.5–4.8)	16.1 (10.3–22.3)	14.6 (9.2–20.5)	2.3 (0.9–3.7)	2.3 (0.9–3.7)	7.3 (4.4–10.4)	20.1 (13.0–27.2)	7.7 (3.0–12.8)	0.4 (0.0–1.1)	0.1 (<0.1–0.7)	0.1 (<0.1–0.7)	19.3 (11.7–29.7)	19.3 (11.7–29.7)
Taiwan (Province of China)	0.4 (0.1–1.2)	0.6 (0.1–1.8)	35.6 (29.0–42.4)	8.0 (5.0–11.7)	12.1 (7.5–17.9)	12.1 (7.5–17.9)	1.8 (0.7–3.0)	1.8 (0.7–3.0)	6.7 (4.1–9.9)	17.3 (11.0–23.6)	10.4 (4.6–15.9)	0.6 (0.0–1.4)	0.9 (0.2–2.3)	0.9 (0.2–2.3)	3.5 (<0.1–6.8)	3.5 (<0.1–6.8)
Oceania	33.9 (21.3–46.6)	39.4 (27.0–50.6)	21.6 (14.3–28.7)	8.7 (5.0–12.6)	4.4 (1.4–10.7)	3.8 (1.2–9.4)	9.1 (4.0–14.4)	9.2 (4.0–14.8)	7.1 (3.4–11.4)	11.4 (5.8–17.6)	2.5 (0.0–6.1)	0.1 (0.0–0.7)	0.5 (<0.1–2.3)	0.5 (<0.1–2.3)	0.9 (<0.1–2.2)	0.9 (<0.1–2.2)
Fiji	4.5 (1.3–9.6)	6.2 (2.1–12.5)	20.6 (13.2–27.7)	8.1 (4.2–12.4)	5.4 (1.4–13.3)	5.4 (1.5–13.1)	2.9 (1.2–4.8)	2.9 (1.2–4.8)	5.6 (2.5–9.4)	8.7 (4.0–14.2)	6.9 (2.9–11.1)	0.4 (0.0–1.0)	0.5 (<0.1–2.4)	0.5 (<0.1–2.4)	<0.1 (<0.1–1.7)	<0.1 (<0.1–1.7)
Kiribati	25.0 (13.6–37.6)	30.4 (19.3–43.0)	26.1 (19.0–33.2)	14.2 (9.0–19.6)	3.7 (1.3–8.4)	3.2 (1.1–7.5)	6.5 (2.7–10.4)	6.5 (2.7–10.4)	9.3 (5.0–14.5)	13.9 (7.6–20.5)	2.3 (0.0–7.1)	<0.1 (0.0–0.3)	0.9 (<0.1–5.0)	0.9 (<0.1–5.0)	<0.1 (<0.1–0.1)	<0.1 (<0.1–0.1)
Marshall Islands	7.9 (3.2–14.9)	10.7 (5.1–18.6)	22.3 (15.8–28.6)	5.4 (3.1–8.5)	4.3 (1.6–9.2)	4.2 (1.6–8.7)	4.5 (1.8–7.3)	4.5 (1.8–7.3)	6.8 (3.5–10.5)	12.2 (6.7–18.0)	4.6 (0.0–10.9)	0.2 (0.0–1.2)	0.2 (<0.1–11.3)	0.2 (<0.1–11.3)	<0.1 (<0.1–0.1)	<0.1 (<0.1–0.1)
Micronesia (Federated States of)	7.0 (2.7–13.8)	9.4 (4.0–17.4)	23.1 (12.2–31.5)	12.5 (5.1–19.7)	5.1 (1.5–12.0)	4.9 (1.5–11.6)	3.9 (1.6–6.4)	3.9 (1.6–6.4)	6.0 (2.6–10.1)	9.6 (4.4–15.8)	6.7 (2.3–11.1)	0.1 (0.0–0.5)	0.2 (<0.1–11.3)	0.2 (<0.1–11.3)	<0.1 (<0.1–0.2)	<0.1 (<0.1–0.2)
Nauru	0.6 (0.1–1.5)	0.8 (0.2–2.1)	20.0 (13.2–26.4)	17.0 (10.7–23.5)	1.9 (0.4–5.1)	2.0 (0.6–5.2)	3.1 (1.2–5.1)	3.1 (1.2–5.1)	6.4 (2.8–10.9)	10.1 (4.5–16.5)	10.3 (3.9–16.2)	0.6 (0.0–2.3)	2.4 (<0.1–31.1)	2.4 (<0.1–31.1)	<0.1 (<0.1–0.3)	<0.1 (<0.1–0.3)
Palau	<0.1 (<0.1–<0.1)	<0.1 (<0.1–<0.1)	22.8 (15.9–29.8)	7.8 (4.4–11.8)	2.0 (<0.1–5.9)	2.0 (<0.1–5.9)	2.4 (0.9–3.9)	2.4 (0.9–3.9)	8.8 (4.7–13.1)	14.4 (8.1–20.7)	4.9 (0.0–12.4)	0.2 (0.0–1.2)	0.9 (<0.1–28.7)	0.9 (<0.1–28.7)	<0.1 (<0.1–0.4)	<0.1 (<0.1–0.4)
Papua New Guinea	38.8 (24.3–53.3)	44.3 (31.0–56.3)	20.0 (12.3–27.6)	8.6 (4.6–12.8)	4.6 (1.3–11.8)	3.8 (1.0–10.1)	12.2 (5.2–19.0)	12.2 (5.2–19.0)	7.2 (3.3–11.9)	11.2 (5.4–17.8)	1.9 (0.0–5.9)	0.1 (0.0–0.7)	0.4 (<0.1–1.9)	0.4 (<0.1–1.9)	1.6 (<0.1–3.7)	1.6 (<0.1–3.7)
Samoa	15.6 (7.3–27.1)	20.0 (10.8–31.7)	23.9 (18.2–30.2)	9.9 (6.6–13.7)	5.1 (1.4–12.0)	4.7 (1.3–11.2)	2.2 (0.9–3.6)	2.2 (0.9–3.6)	11.0 (6.6–15.7)	17.1 (10.6–23.4)	5.6 (0.2–11.4)	0.3 (0.0–1.5)	0.4 (<0.1–2.2)	0.4 (<0.1–2.2)	0.1 (<0.1–1.4)	0.1 (<0.1–1.4)
Solomon Islands	38.8 (24.7–53.6)	44.9 (31.0–57.2)	25.5 (17.9–32.7)	9.2 (5.4–13.3)	3.9 (1.2–9.1)	3.2 (0.9–7.3)	4.9 (2.0–7.9)	4.9 (2.0–7.9)	7.2 (3.6–11.1)	12.2 (6.5–18.4)	1.7 (0.0–5.2)	0.1 (0.0–0.6)	0.5 (<0.1–3.3)	0.5 (<0.1–3.3)	<0.1 (<0.1–0.5)	<0.1 (<0.1–0.5)
Tonga	7.1 (2.5–14.0)	9.6 (3.8–18.0)	22.7 (16.4–29.3)	8.4 (4.9–12.2)	5.2 (1.5–12.2)	5.1 (1.6–11.7)	3.3 (1.3–5.4)	3.3 (1.3–5.4)	8.2 (4.3–12.3)	13.9 (7.8–20.2)	2.4 (0.0–7.0)	0.1 (0.0–0.8)	1.2 (<0.1–3.0)	1.2 (<0.1–3.0)	0.2 (<0.1–2.6)	0.2 (<0.1–2.6)

	Household air pollution		Smoking		Ambient particulate matter		Handwashing		Secondhand smoke		Alcohol use		High temperature		Low temperature	
	**M**	**F**	**M**	**F**	**M**	**F**	**M**	**F**	**M**	**F**	**M**	**F**	**M**	**F**	**M**	**F**
Tuvalu	1.8 (0.4–4.3)	2.5 (0.8–5.5)	22.0 (15.1–29.1)	10.0 (5.7–14.9)	2.4 (0.8–5.6)	2.5 (1.0–5.6)	3.7 (1.5–6.1)	3.7 (1.5–6.1)	7.6 (3.7–12.1)	12.0 (6.1–18.4)	3.4 (0.0–8.0)	0.1 (0.0–0.8)	1.4 (<0.1–12.8)	1.4 (<0.1–12.8)	0.1 (<0.1–0.7)	0.1 (<0.1–0.7)
Vanuatu	29.4 (16.9–43.6)	35.6 (22.9–48.9)	20.1 (12.8–27.2)	2.9 (1.4–4.7)	4.8 (1.4–10.7)	4.2 (1.2–9.3)	10.0 (4.3–15.7)	10.0 (4.3–15.7)	4.6 (1.9–7.9)	9.1 (4.0–15.2)	4.7 (1.2–8.4)	0.1 (0.0–0.4)	0.4 (<0.1–2.1)	0.4 (<0.1–2.1)	<0.1 (<0.1–1.8)	<0.1 (<0.1–1.8)
Southeast Asia	10.1 (5.1–16.9)	14.1 (7.8–21.5)	26.0 (19.2–32.2)	2.9 (1.7–4.4)	10.0 (6.6–14.4)	9.4 (6.3–13.2)	7.0 (2.9–11.1)	7.1 (2.9–11.4)	5.5 (3.1–8.4)	14.5 (8.4–20.6)	9.7 (4.6–13.9)	1.6 (0.3–2.8)	2.0 (<0.1–10.6)	2.0 (<0.1–11.8)	0.1 (<0.1–1.3)	0.1 (<0.1–1.1)
Cambodia	27.8 (16.4–41.1)	33.8 (21.7–46.8)	23.4 (17.7–29.4)	2.7 (1.5–4.3)	8.0 (4.5–12.6)	6.9 (3.9–11.0)	8.6 (3.5–13.5)	8.6 (3.5–13.5)	7.8 (4.7–11.3)	17.9 (11.3–24.4)	12.3 (5.6–18.6)	2.2 (0.0–5.1)	2.7 (<0.1–21.8)	2.7 (<0.1–21.8)	<0.1 (<0.1–0.3)	<0.1 (<0.1–0.3)
Indonesia	6.3 (2.5–12.2)	9.2 (4.1–16.3)	28.5 (21.0–35.4)	2.1 (1.2–3.2)	9.5 (6.0–14.3)	9.0 (5.8–13.2)	7.3 (3.0–11.6)	7.6 (3.1–12.1)	4.6 (2.5–7.1)	17.1 (10.1–24.4)	0.3 (0.0–1.7)	<0.1 (0.0–0.1)	0.3 (<0.1–3.4)	0.4 (<0.1–3.6)	<0.1 (<0.1–0.9)	<0.1 (<0.1–0.9)
Lao People's Democratic Republic	30.0 (17.6–44.0)	36.2 (23.2–49.1)	21.5 (15.0–27.9)	2.6 (1.3–4.2)	7.3 (4.2–11.1)	6.4 (3.6–9.8)	10.2 (4.3–15.9)	10.2 (4.3–15.9)	6.3 (3.3–9.5)	14.4 (8.3–21.1)	10.4 (3.3–17.2)	4.3 (0.5–8.3)	1.8 (0.6–3.9)	1.8 (0.6–3.9)	<0.1 (<0.1–2.9)	<0.1 (<0.1–2.9)
Malaysia	0.1 (<0.1–0.2)	0.1 (<0.1–0.3)	30.4 (23.0–37.8)	2.1 (1.1–3.6)	8.3 (4.7–13.2)	8.3 (4.7–13.2)	2.0 (0.8–3.2)	2.0 (0.8–3.2)	5.7 (3.1–8.8)	15.3 (8.9–21.8)	2.8 (0.5–5.6)	0.2 (0.0–0.6)	0.8 (<0.1–3.9)	0.8 (<0.1–3.9)	<0.1 (<0.1–0.8)	<0.1 (<0.1–0.8)
Maldives	2.2 (0.7–5.1)	3.2 (1.1–6.9)	29.1 (22.2–35.8)	2.4 (1.2–4.0)	5.2 (2.9–8.7)	5.2 (3.0–8.4)	8.1 (3.5–13.0)	8.1 (3.5–13.0)	7.9 (4.4–11.8)	16.2 (9.6–22.9)	1.2 (0.0–5.2)	0.1 (0.0–0.5)	0.4 (<0.1–11.4)	0.4 (<0.1–11.4)	<0.1 (<0.1–0.1)	<0.1 (<0.1–0.1)
Mauritius	0.1 (<0.1–0.4)	0.2 (0.1–0.5)	23.6 (16.0–30.5)	3.4 (1.8–5.3)	7.3 (3.0–13.8)	7.3 (3.1–13.7)	1.1 (0.4–1.8)	1.1 (0.4–1.8)	5.7 (3.0–8.7)	13.8 (7.9–20.1)	6.8 (1.1–12.7)	0.3 (0.0–1.6)	0.7 (<0.1–3.5)	0.7 (<0.1–3.5)	<0.1 (<0.1–5.4)	<0.1 (<0.1–5.4)
Myanmar	20.8 (11.0–32.7)	26.4 (15.7–38.8)	19.3 (12.1–26.2)	2.1 (1.0–3.6)	11.6 (7.8–15.9)	10.4 (7.1–14.3)	11.1 (4.7–17.5)	11.1 (4.7–17.5)	4.2 (1.8–6.9)	10.0 (4.7–16.0)	7.4 (2.1–12.7)	0.2 (0.0–0.7)	2.9 (1.3–5.3)	2.9 (1.3–5.3)	1.9 (<0.1–4.3)	1.9 (<0.1–4.3)
Philippines	12.1 (5.7–20.5)	15.9 (8.5–24.8)	26.3 (20.0–32.5)	4.6 (2.7–6.8)	9.2 (6.0–13.1)	8.6 (5.8–12.0)	6.2 (2.6–9.9)	6.3 (2.6–10.0)	6.5 (3.7–9.9)	14.2 (8.3–20.4)	13.0 (6.1–18.7)	3.0 (0.6–5.4)	2.5 (<0.1–25.6)	2.4 (<0.1–24.7)	0.2 (<0.1–0.7)	0.2 (<0.1–0.7)
Seychelles	0.1 (<0.1–0.2)	0.1 (<0.1–0.2)	28.7 (21.6–36.1)	4.3 (2.4–7.0)	7.7 (3.3–14.3)	7.7 (3.3–14.3)	3.9 (1.6–6.3)	3.9 (1.6–6.3)	5.3 (2.9–8.1)	13.8 (8.1–19.8)	13.4 (5.9–20.5)	1.4 (0.0–3.3)	1.0 (<0.1–3.8)	1.0 (<0.1–3.8)	0.2 (<0.1–1.3)	0.2 (<0.1–1.3)
Sri Lanka	7.9 (2.9–15.9)	10.9 (4.4–20.9)	15.6 (8.9–22.5)	1.4 (0.8–2.3)	9.9 (5.8–15.5)	9.6 (5.7–14.8)	5.0 (2.0–8.1)	5.0 (2.0–8.1)	3.8 (1.5–6.8)	7.9 (3.3–13.5)	12.8 (6.9–18.5)	0.8 (0.1–1.6)	1.2 (<0.1–13.3)	1.2 (<0.1–13.3)	<0.1 (<0.1–0.8)	<0.1 (<0.1–0.8)
Thailand	2.7 (0.9–5.8)	3.9 (1.4–8.1)	25.7 (18.3–32.7)	2.2 (1.2–3.3)	13.8 (8.8–20.0)	13.6 (8.8–19.6)	7.5 (3.1–12.1)	7.5 (3.1–12.1)	4.8 (2.6–7.4)	11.7 (6.6–17.1)	15.4 (8.6–21.3)	1.8 (0.2–3.5)	2.9 (<0.1–10.6)	2.9 (<0.1–10.6)	<0.1 (<0.1–1.0)	<0.1 (<0.1–1.0)
Timor-Leste	26.9 (15.2–40.2)	32.1 (19.9–45.2)	20.8 (9.6–28.6)	1.5 (0.6–2.6)	6.0 (2.9–10.9)	5.4 (2.5–9.8)	6.6 (2.7–10.6)	6.6 (2.7–10.6)	4.7 (2.2–8.0)	11.9 (5.9–18.5)	6.3 (1.4–11.6)	0.4 (0.0–1.2)	0.5 (<0.1–2.1)	0.5 (<0.1–2.1)	<0.1 (<0.1–1.7)	<0.1 (<0.1–1.7)
Viet Nam	7.9 (3.2–15.0)	10.9 (5.0–19.3)	29.0 (22.3–35.8)	1.1 (0.6–1.9)	10.0 (6.4–14.6)	9.6 (6.3–13.8)	8.6 (3.6–13.6)	8.6 (3.6–13.6)	5.4 (3.0–8.0)	18.5 (11.4–25.6)	14.5 (6.6–21.9)	0.8 (0.0–2.1)	2.7 (0.9–5.9)	2.7 (0.9–5.9)	0.2 (<0.1–3.2)	0.2 (<0.1–3.2)
**Sub-Saharan Africa**	**30.6 (20.7–40.4)**	**36.7 (26.5–47.1)**	**11.8 (7.8–16.0)**	**1.8 (1.0–2.7)**	**11.2 (7.3–15.9)**	**9.5 (6.0–13.8)**	**21.3 (9.8–31.5)**	**21.5 (9.8–31.9)**	**3.9 (1.8–6.3)**	**5.8 (2.8–9.2)**	**7.3 (3.4–10.8)**	**2.0 (0.5–3.7)**	**2.9 (0.6–9.4)**	**3.1 (0.6–9.7)**	**1.8 (<0.1–3.5)**	**1.6 (<0.1–3.4)**
Central Sub-Saharan Africa	33.7 (21.9–45.6)	38.8 (26.9–49.7)	11.5 (7.2–15.9)	1.1 (0.6–1.7)	9.8 (5.1–16.8)	8.3 (4.0–14.2)	22.6 (10.4–33.2)	22.6 (10.4–33.2)	2.7 (1.2–4.8)	3.8 (1.6–6.5)	4.7 (0.8–8.6)	1.9 (0.0–4.3)	1.5 (0.3–3.4)	1.5 (0.4–3.4)	<0.1 (<0.1–2.4)	<0.1 (<0.1–2.4)

	**Household air pollution**		**Smoking**		**Ambient particulate matter**		**Handwashing**		**Secondhand smoke**		**Alcohol use**		**High temperature**		**Low temperature**	
	**M**	**F**	**M**	**F**	**M**	**F**	**M**	**F**	**M**	**F**	**M**	**F**	**M**	**F**	**M**	**F**
Angola	15.3 (8.0–25.2)	19.4 (11.1–29.4)	14.5 (10.4–19.2)	1.7 (1.0–2.7)	11.6 (6.1–19.0)	10.6 (5.6–17.2)	22.3 (10.2–32.8)	22.3 (10.2–32.8)	5.2 (2.9–8.0)	9.2 (5.2–13.6)	11.5 (5.7–16.5)	5.4 (1.7–8.9)	1.7 (0.5–3.8)	1.7 (0.5–3.8)	<0.1 (<0.1–2.9)	<0.1 (<0.1–2.9)
Central African Republic	47.3 (32.4–59.4)	51.1 (38.9–63.1)	11.6 (7.4–16.2)	1.1 (0.6–1.9)	7.7 (2.5–16.3)	5.9 (1.8–13.0)	21.6 (9.9–31.8)	21.6 (9.9–31.8)	3.5 (1.6–5.9)	4.9 (2.3–8.0)	3.4 (0.0–8.0)	1.2 (0.0–3.8)	3.8 (1.5–7.0)	3.8 (1.5–7.0)	<0.1 (<0.1–1.2)	<0.1 (<0.1–1.2)
Congo	12.2 (5.7–21.7)	16.3 (8.8–26.2)	14.4 (9.4–20.0)	1.1 (0.5–1.8)	16.4 (8.9–27.6)	15.3 (8.3–26.0)	21.0 (9.5–31.0)	21.0 (9.5–31.0)	3.9 (1.8–6.5)	5.9 (2.8–9.4)	7.2 (1.2–13.3)	3.5 (0.0–8.0)	0.9 (<0.1–3.0)	0.9 (<0.1–3.0)	<0.1 (<0.1–1.9)	<0.1 (<0.1–1.9)
Democratic Republic of the Congo	39.2 (25.1–52.5)	44.7 (31.1–56.1)	10.5 (6.0–15.2)	0.9 (0.5–1.5)	9.0 (4.1–16.1)	7.3 (3.1–13.3)	23.0 (10.6–33.7)	23.0 (10.6–33.7)	1.9 (0.5–3.8)	2.2 (0.6–4.6)	2.6 (0.0–6.8)	0.9 (0.0–3.2)	1.3 (0.1–3.1)	1.3 (0.1–3.1)	0.1 (<0.1–2.6)	0.1 (<0.1–2.6)
Equatorial Guinea	3.5 (1.3–7.2)	4.7 (1.9–9.2)	11.8 (7.2–16.8)	1.1 (0.5–1.9)	20.4 (10.1–33.6)	20.1 (10.1–33.1)	19.9 (9.0–29.7)	19.9 (9.0–29.7)	4.1 (1.7–7.2)	5.1 (2.1–8.7)	10.4 (4.6–16.5)	5.0 (1.0–9.4)	0.4 (<0.1–1.3)	0.4 (<0.1–1.3)	<0.1 (<0.1–2.4)	<0.1 (<0.1–2.4)
Gabon	1.2 (0.4–2.6)	1.8 (0.6–3.4)	14.1 (8.8–19.6)	1.9 (0.9–3.2)	17.8 (9.2–29.9)	17.7 (9.2–29.8)	16.9 (7.4–25.6)	16.9 (7.4–25.6)	4.1 (1.8–6.9)	6.0 (2.8–9.9)	12.7 (6.2–19.0)	6.3 (1.9–11.2)	0.7 (<0.1–2.6)	0.7 (<0.1–2.6)	<0.1 (<0.1–1.5)	<0.1 (<0.1–1.5)
Eastern Sub-Saharan Africa	40.0 (28.0–51.8)	44.9 (33.4–55.9)	9.9 (6.1–14.0)	1.8 (0.9–2.9)	6.8 (3.9–10.5)	5.6 (3.1–8.9)	22.4 (10.3–33.0)	22.5 (10.4–33.0)	3.3 (1.3–5.7)	4.6 (1.9–8.0)	5.8 (2.4–9.1)	1.9 (0.4–3.3)	2.1 (0.3–5.6)	2.1 (0.3–5.6)	1.4 (<0.1–3.9)	1.3 (<0.1–3.8)
Burundi	48.9 (34.4–60.6)	52.2 (40.3–63.9)	8.4 (4.9–12.4)	1.7 (0.7–3.1)	5.7 (2.0–11.7)	4.4 (1.4–10.0)	22.9 (10.6–33.7)	22.9 (10.6–33.7)	2.7 (1.0–5.0)	3.4 (1.2–6.2)	11.2 (5.1–17.3)	2.1 (0.5–3.9)	<0.1 (<0.1–0.3)	<0.1 (<0.1–0.3)	<0.1 (<0.1–6.1)	<0.1 (<0.1–6.1)
Comoros	30.0 (17.6–43.7)	36.3 (24.1–49.3)	14.2 (8.8–20.0)	1.1 (0.5–2.0)	6.1 (3.4–9.7)	5.2 (2.8–8.4)	21.0 (9.6–31.1)	21.0 (9.6–31.1)	5.5 (2.6–9.0)	8.3 (4.0–13.2)	0.7 (0.0–2.2)	0.3 (0.0–1.0)	0.7 (<0.1–2.9)	0.7 (<0.1–2.9)	0.2 (<0.1–2.9)	0.2 (<0.1–2.9)
Djibouti	6.8 (2.9–13.4)	9.2 (4.2–16.8)	18.6 (11.9–25.7)	2.3 (1.0–4.0)	18.5 (9.0–32.0)	17.5 (8.5–30.8)	21.6 (9.9–32.0)	21.6 (9.9–32.0)	4.9 (2.2–8.3)	9.5 (4.3–15.4)	0.3 (0.0–1.7)	0.1 (0.0–0.5)	5.9 (<0.1–30.0)	5.9 (<0.1–30.0)	0.2 (<0.1–5.2)	0.2 (<0.1–5.2)
Eritrea	30.1 (17.2–43.4)	35.5 (22.7–47.2)	12.8 (8.4–17.9)	0.5 (0.2–0.8)	12.5 (6.1–21.1)	10.6 (4.9–17.9)	22.6 (10.4–33.1)	22.6 (10.4–33.1)	4.8 (2.6–7.5)	5.3 (2.8–8.1)	1.6 (0.0–4.8)	0.4 (0.0–1.5)	4.5 (<0.1–22.1)	4.5 (<0.1–22.1)	0.1 (<0.1–2.5)	0.1 (<0.1–2.5)
Ethiopia	44.6 (30.9–55.8)	48.6 (36.7–59.8)	4.7 (2.7–6.9)	0.4 (0.2–0.8)	6.9 (3.3–11.7)	5.5 (2.6–9.9)	22.9 (10.6–33.6)	23.0 (10.6–33.6)	2.2 (0.7–4.1)	2.2 (0.8–4.1)	4.3 (1.0–8.3)	1.3 (0.0–3.3)	3.4 (0.6–11.1)	3.9 (0.7–13.2)	4.5 (3.0–6.4)	4.2 (2.7–6.1)
Kenya	25.9 (16.2–36.9)	31.8 (21.1–42.7)	10.9 (6.7–15.1)	1.5 (0.8–2.5)	7.4 (4.4–11.4)	6.5 (3.8–10.2)	21.9 (10.1–32.4)	22.0 (10.2–32.6)	3.3 (1.4–5.6)	4.7 (2.1–8.0)	6.8 (2.1–11.8)	1.2 (0.3–2.4)	2.6 (0.2–10.4)	2.6 (0.2–10.8)	3.4 (1.4–5.9)	3.3 (1.1–5.8)
Madagascar	40.9 (26.9–54.8)	46.5 (33.2–58.5)	10.9 (5.9–15.9)	1.1 (0.5–1.8)	5.0 (2.6–8.6)	4.1 (2.1–7.2)	22.4 (10.3–33.0)	22.4 (10.3–33.0)	2.8 (0.8–5.4)	4.4 (1.4–8.6)	2.1 (0.0–5.8)	0.5 (0.0–1.7)	0.8 (<0.1–2.4)	0.8 (<0.1–2.4)	1.0 (<0.1–3.8)	1.0 (<0.1–3.8)
Malawi	43.6 (29.8–56.5)	48.3 (34.9–59.5)	10.3 (5.9–15.3)	1.1 (0.4–1.9)	5.4 (2.5–9.6)	4.4 (2.0–8.0)	22.6 (10.4–33.3)	22.6 (10.4–33.3)	3.5 (1.5–6.0)	5.1 (2.1–8.8)	5.4 (1.9–9.4)	1.4 (0.0–3.4)	0.5 (<0.1–1.9)	0.5 (<0.1–1.9)	0.2 (<0.1–4.4)	0.2 (<0.1–4.4)
Mozambique	47.9 (33.4–59.5)	51.5 (39.4–62.3)	10.6 (6.2–15.6)	2.5 (1.0–4.3)	4.2 (1.8–7.7)	3.3 (1.4–6.3)	22.2 (10.2–32.7)	22.2 (10.2–32.7)	3.1 (1.0–5.8)	4.2 (1.4–7.8)	1.7 (0.0–5.5)	0.5 (0.0–2.3)	2.4 (1.0–4.4)	2.4 (1.0–4.4)	0.2 (<0.1–3.3)	0.2 (<0.1–3.3)
Rwanda	35.8 (22.3–49.7)	41.8 (28.6–54.8)	11.3 (7.2–15.7)	2.2 (1.2–3.5)	10.1 (4.7–17.4)	8.5 (3.8–15.7)	23.2 (10.7–34.0)	23.2 (10.7–34.0)	4.8 (2.3–7.6)	6.2 (3.2–9.7)	12.0 (5.1–18.8)	4.6 (0.8–9.0)	<0.1 (<0.1–0.1)	<0.1 (<0.1–0.1)	2.0 (<0.1–7.5)	2.0 (<0.1–7.5)
Somalia	58.8 (45.2–80.6)	61.1 (46.5–84.7)	11.3 (6.3–16.5)	1.0 (0.4–1.8)	2.0 (0.4–6.3)	1.5 (0.3–4.4)	22.7 (10.5–33.4)	22.7 (10.5–33.4)	3.5 (1.5–6.2)	4.9 (2.0–8.5)	0.0 (0.0–0.0)	0.0 (0.0–0.0)	2.3 (<0.1–15.8)	2.3 (<0.1–15.8)	0.1 (<0.1–1.3)	0.1 (<0.1–1.3)

	Household air pollution		Smoking		Ambient particulate matter		Handwashing		Secondhand smoke		Alcohol use		High temperature		Low temperature	
	M	F	M	F	M	F	M	F	M	F	M	F	M	F	M	F
South Sudan	41.0 (26.9–54.4)	46.1 (32.4–57.8)	11.4 (6.5–16.6)	1.0 (0.4–1.8)	8.9 (4.2–15.3)	7.3 (3.2–13.1)	22.9 (10.6–33.6)	22.9 (10.6–33.6)	3.8 (1.6–6.6)	5.0 (2.2–8.5)	0.3 (0.0–1.4)	0.1 (0.0–0.4)	5.9 (<0.1–23.1)	5.9 (<0.1–23.1)	<0.1 (<0.1–0.9)	<0.1 (<0.1–0.9)
Uganda	38.5 (25.1–51.6)	43.8 (30.4–55.8)	7.8 (4.4–11.6)	1.1 (0.5–2.0)	9.2 (4.8–14.8)	7.7 (3.9–12.6)	22.1 (10.1–32.5)	22.1 (10.1–32.5)	3.4 (1.4–6.0)	4.5 (1.9–7.7)	13.7 (7.1–19.6)	6.3 (2.5–10.2)	1.1 (0.2–2.9)	1.1 (0.2–2.9)	<0.1 (<0.1–2.3)	<0.1 (<0.1–2.3)
United Republic of Tanzania	37.7 (23.9–51.3)	43.6 (30.0–56.1)	15.3 (9.5–21.5)	6.1 (2.9–10.0)	7.2 (4.0–11.1)	6.0 (3.3–9.5)	22.5 (10.4–33.2)	22.5 (10.4–33.2)	4.7 (2.1–7.5)	7.5 (3.6–11.9)	9.6 (4.8–14.4)	3.3 (1.0–5.8)	0.9 (0.1–2.7)	0.9 (0.1–2.7)	0.1 (<0.1–3.5)	0.1 (<0.1–3.5)
Zambia	24.7 (14.0–37.9)	30.4 (18.9–43.2)	10.9 (5.8–16.1)	1.8 (0.7–3.2)	9.7 (5.6–15.2)	8.6 (5.0–13.5)	21.3 (9.7–31.5)	21.3 (9.7–31.5)	3.2 (1.0–6.3)	5.3 (1.7–10.2)	8.9 (3.2–14.3)	2.6 (0.6–4.9)	1.2 (0.3–3.5)	1.2 (0.3–3.5)	<0.1 (<0.1–5.0)	<0.1 (<0.1–5.0)
Southern Sub-Saharan Africa	8.7 (4.3–14.3)	10.4 (5.9–16.2)	19.1 (13.2–25.2)	4.2 (2.5–6.2)	12.9 (8.3–18.7)	12.8 (8.3–18.5)	16.5 (7.3–25.0)	16.3 (7.3–24.6)	4.6 (2.2–7.3)	8.8 (4.5–13.6)	12.6 (6.9–17.4)	3.1 (1.2–5.1)	1.1 (0.5–2.3)	1.1 (0.5–2.4)	6.7 (4.1–9.3)	6.8 (4.2–9.5)
Botswana	6.8 (2.6–13.2)	9.4 (4.2–16.9)	21.9 (15.0–28.9)	5.0 (2.9–7.4)	12.1 (7.2–18.8)	11.6 (7.0–17.8)	17.0 (7.5–25.6)	17.0 (7.5–25.6)	6.2 (3.0–9.8)	10.0 (5.0–15.4)	12.1 (6.3–17.7)	2.6 (0.8–4.7)	4.0 (1.5–7.7)	4.0 (1.5–7.7)	0.9 (<0.1–7.0)	0.9 (<0.1–7.0)
Eswatini	12.8 (5.8–22.5)	16.9 (8.8–27.6)	8.2 (4.6–12.2)	0.9 (0.5–1.5)	10.7 (6.3–16.3)	10.0 (6.0–15.0)	16.5 (7.4–25.0)	16.5 (7.4–25.0)	2.7 (1.1–4.9)	4.2 (1.7–7.6)	11.7 (6.5–16.6)	2.0 (0.7–3.5)	0.4 (<0.1–1.2)	0.4 (<0.1–1.2)	3.3 (<0.1–8.1)	3.3 (<0.1–8.1)
Lesotho	18.2 (9.3–29.3)	23.4 (13.3–34.9)	26.6 (20.3–33.1)	0.8 (0.5–1.2)	11.2 (6.7–16.8)	10.0 (6.1–15.4)	23.1 (10.7–33.9)	23.1 (10.7–33.9)	5.8 (3.2–8.6)	13.8 (8.2–19.6)	9.5 (3.1–15.0)	2.3 (0.0–5.0)	<0.1 (<0.1–<0.1)	<0.1 (<0.1–<0.1)	21.7 (15.2–28.4)	21.7 (15.2–28.4)
Namibia	9.6 (4.0–17.9)	12.9 (6.1–22.0)	11.4 (6.4–16.6)	3.3 (1.6–5.3)	11.5 (6.4–17.9)	10.9 (6.2–16.7)	14.3 (6.2–22.0)	14.3 (6.2–22.0)	3.8 (1.5–7.0)	5.7 (2.2–10.0)	12.8 (5.6–19.2)	6.1 (1.5–10.9)	3.2 (1.3–6.0)	3.2 (1.3–6.0)	1.3 (<0.1–6.0)	1.3 (<0.1–6.0)
South Africa	2.4 (0.9–4.5)	3.3 (1.4–6.0)	18.8 (12.0–25.6)	5.4 (3.2–8.2)	15.0 (9.5–21.7)	15.0 (9.7–21.5)	14.5 (6.4–22.3)	14.4 (6.4–22.3)	4.5 (2.0–7.4)	8.1 (4.0–13.0)	14.2 (8.0–19.4)	3.7 (1.6–6.0)	0.7 (0.3–2.0)	0.8 (0.3–2.0)	8.4 (6.3–10.8)	8.3 (6.2–10.7)
Zimbabwe	25.1 (14.0–37.9)	31.0 (19.5–43.8)	19.7 (14.0–25.7)	1.2 (0.7–2.0)	7.9 (4.6–12.4)	6.9 (3.9–10.6)	20.9 (9.6–31.0)	20.9 (9.6–31.0)	4.6 (2.4–7.1)	10.6 (5.9–15.8)	8.6 (3.2–14.3)	1.0 (0.0–2.5)	1.7 (0.7–3.8)	1.7 (0.7–3.8)	0.7 (<0.1–5.1)	0.7 (<0.1–5.1)
Western Sub-Saharan Africa	30.0 (20.2–40.6)	36.7 (26.2–47.4)	10.3 (7.0–14.0)	1.2 (0.7–1.9)	15.6 (10.0–22.7)	12.9 (8.1–19.2)	21.8 (10.0–32.1)	21.8 (10.0–32.1)	4.6 (2.4–7.0)	6.7 (3.5–10.1)	7.5 (3.8–10.7)	1.9 (0.4–3.6)	5.3 (0.4–19.2)	5.7 (0.6–21.2)	0.5 (<0.1–2.4)	0.6 (<0.1–2.8)
Benin	38.5 (25.0–52.3)	43.6 (30.6–55.0)	8.9 (5.7–12.7)	1.2 (0.6–2.0)	11.0 (5.4–18.3)	9.0 (4.2–15.4)	22.1 (10.1–32.6)	22.1 (10.1–32.6)	4.7 (2.4–7.2)	6.6 (3.5–10.2)	4.5 (1.3–8.1)	1.6 (0.0–3.9)	3.3 (<0.1–18.5)	3.3 (<0.1–18.5)	<0.1 (<0.1–1.2)	<0.1 (<0.1–1.2)
Burkina Faso	46.9 (31.7–59.5)	50.5 (38.1–63.9)	13.1 (7.9–18.7)	1.2 (0.6–2.1)	8.1 (2.8–16.2)	6.2 (2.1–13.4)	22.2 (10.2–32.7)	22.2 (10.2–32.7)	3.5 (1.5–6.1)	6.1 (2.7–10.4)	8.7 (3.2–14.6)	5.3 (0.4–10.6)	4.6 (<0.1–34.1)	4.6 (<0.1–34.1)	<0.1 (<0.1–3.1)	<0.1 (<0.1–3.1)
Cabo Verde	5.8 (2.6–10.6)	7.8 (4.0–13.5)	8.7 (5.5–12.4)	1.9 (0.9–3.1)	21.9 (13.5–31.7)	21.0 (13.2–30.6)	19.0 (8.5–28.3)	19.0 (8.5–28.3)	4.5 (2.0–7.1)	6.4 (3.1–10.2)	9.1 (4.8–13.2)	3.8 (1.1–6.8)	0.2 (<0.1–1.1)	0.2 (<0.1–1.1)	<0.1 (<0.1–6.3)	<0.1 (<0.1–6.3)
Cameroon	20.1 (11.2–31.6)	25.3 (15.3–36.8)	11.2 (7.4–15.4)	0.9 (0.4–1.6)	20.6 (13.1–29.3)	18.5 (11.6–26.3)	21.8 (10.0–32.1)	21.8 (10.0–32.1)	3.7 (1.7–6.0)	6.5 (3.1–10.2)	9.9 (5.1–14.3)	4.2 (1.3–7.2)	2.0 (0.1–7.1)	2.0 (0.1–7.1)	<0.1 (<0.1–1.6)	<0.1 (<0.1–1.6)
Chad	48.0 (33.3–61.1)	51.2 (39.2–65.3)	11.6 (7.2–16.6)	1.8 (0.9–2.9)	8.0 (2.9–17.2)	6.2 (2.2–13.9)	22.3 (10.2–32.8)	22.3 (10.2–32.8)	4.1 (1.8–6.7)	7.0 (3.3–11.6)	5.2 (0.6–9.8)	2.8 (0.0–6.6)	7.0 (0.4–22.9)	7.0 (0.4–22.9)	1.3 (<0.1–7.7)	1.3 (<0.1–7.7)
Côte d'Ivoire	30.1 (17.7–43.8)	36.0 (24.1–48.2)	18.5 (13.3–24.0)	2.0 (1.1–3.3)	15.2 (8.2–24.9)	13.0 (6.6–22.0)	20.8 (9.5–31.0)	20.8 (9.5–31.0)	6.7 (3.7–10.3)	10.4 (5.8–15.2)	10.3 (3.9–16.1)	1.9 (0.0–4.8)	1.6 (<0.1–5.7)	1.6 (<0.1–5.7)	0.2 (<0.1–1.2)	0.2 (<0.1–1.2)

	Household air pollution		Smoking		Ambient particulate matter		Handwashing		Secondhand smoke		Alcohol use		High temperature		Low temperature	
	**M**	**F**	**M**	**F**	**M**	**F**	**M**	**F**	**M**	**F**	**M**	**F**	**M**	**F**	**M**	**F**
Gambia	35.9 (22.6–49.4)	41.4 (28.2–52.8)	16.1 (11.0–22.2)	0.6 (0.3–1.0)	13.8 (7.2–22.3)	11.5 (5.8–19.5)	21.9 (10.1–32.3)	21.9 (10.1–32.3)	6.8 (3.6–10.4)	9.9 (5.3–14.8)	5.0 (1.7–8.4)	1.5 (0.0–3.6)	2.2 (<0.1–20.9)	2.2 (<0.1–20.9)	0.1 (<0.1–1.5)	0.1 (<0.1–1.5)
Ghana	14.8 (7.6–24.8)	19.3 (11.0–30.3)	6.1 (3.5–9.1)	1.7 (0.9–2.9)	20.1 (12.8–29.9)	18.6 (11.7–27.8)	22.3 (10.3–32.8)	22.3 (10.3–32.8)	3.4 (1.6–5.6)	4.5 (2.1–7.1)	7.7 (2.3–12.7)	1.8 (0.0–4.2)	2.9 (<0.1–18.5)	2.9 (<0.1–18.5)	<0.1 (<0.1–0.7)	<0.1 (<0.1–0.7)
Guinea	44.3 (29.5–56.5)	48.3 (35.1–59.4)	17.6 (11.9–24.1)	1.2 (0.6–1.9)	9.6 (4.1–17.2)	7.5 (3.2–14.5)	22.3 (10.3–32.8)	22.3 (10.3–32.8)	5.1 (2.6–7.9)	7.9 (4.2–12.0)	2.2 (0.4–4.2)	0.2 (0.0–0.9)	2.7 (0.7–7.1)	2.7 (0.7–7.1)	0.1 (<0.1–1.3)	0.1 (<0.1–1.3)
Guinea-Bissau	41.7 (27.4–54.6)	46.5 (33.2–57.7)	7.4 (4.8–10.5)	0.8 (0.4–1.3)	10.9 (4.8–19.0)	8.7 (3.8–15.7)	22.3 (10.3–32.8)	22.3 (10.3–32.8)	5.0 (2.6–8.0)	7.6 (4.0–11.6)	6.7 (2.5–10.8)	2.0 (0.0–4.3)	2.2 (<0.1–22.7)	2.2 (<0.1–22.7)	<0.1 (<0.1–1.3)	<0.1 (<0.1–1.3)
Liberia	39.1 (24.3–51.9)	44.3 (31.6–55.6)	9.4 (5.4–13.7)	1.4 (0.7–2.3)	11.5 (5.6–19.8)	9.3 (4.5–16.2)	23.3 (10.8–34.1)	23.3 (10.8–34.1)	3.3 (1.4–5.8)	4.7 (2.0–8.0)	8.4 (3.2–13.5)	4.1 (1.5–7.1)	0.9 (<0.1–3.3)	0.9 (<0.1–3.3)	0.1 (<0.1–2.0)	0.1 (<0.1–2.0)
Mali	47.4 (32.7–60.0)	50.8 (37.3–64.4)	12.4 (7.5–17.5)	0.6 (0.2–1.1)	8.6 (3.0–17.2)	6.7 (2.4–14.5)	21.9 (10.1–32.3)	21.9 (10.1–32.3)	3.9 (1.7–6.5)	6.2 (2.9–10.3)	1.1 (0.4–1.9)	1.2 (0.2–2.4)	7.3 (<0.1–26.7)	7.3 (<0.1–26.7)	1.3 (<0.1–13.3)	1.3 (<0.1–13.3)
Mauritania	15.6 (7.5–25.5)	19.9 (10.8–30.4)	18.7 (12.7–24.8)	4.8 (2.5–7.5)	22.4 (13.1–34.1)	20.5 (11.7–31.4)	19.6 (8.8–29.2)	19.6 (8.8–29.2)	4.1 (1.8–6.9)	6.4 (2.9–10.8)	<0.1 (0.0–<0.1)	0.0 (0.0–0.0)	8.0 (1.7–19.5)	8.0 (1.7–19.5)	1.9 (<0.1–13.0)	1.9 (<0.1–13.0)
Niger	51.6 (36.5–70.0)	54.7 (40.3–74.8)	9.8 (6.2–13.9)	0.8 (0.4–1.4)	7.2 (1.9–18.4)	5.6 (1.5–15.0)	21.4 (9.8–31.7)	21.4 (9.8–31.7)	4.4 (2.1–7.0)	6.7 (3.4–10.3)	0.3 (0.0–1.4)	0.1 (0.0–0.5)	8.4 (0.5–23.3)	8.4 (0.5–23.3)	1.6 (<0.1–11.5)	1.6 (<0.1–11.5)
Nigeria	25.7 (16.5–35.8)	30.6 (21.3–41.0)	7.3 (5.0–10.0)	0.8 (0.4–1.2)	18.4 (11.4–27.2)	16.1 (9.7–24.0)	21.9 (10.0–32.1)	21.9 (10.1–32.3)	4.6 (2.5–6.9)	5.8 (3.2–8.6)	9.6 (5.1–13.7)	1.3 (0.1–2.6)	8.8 (1.3–38.7)	9.1 (1.4–39.9)	1.0 (<0.1–2.8)	1.0 (<0.1–2.9)
Sao Tome and Principe	18.1 (9.3–29.0)	22.9 (13.1–34.0)	6.9 (4.4–9.8)	0.8 (0.4–1.4)	12.3 (6.7–19.5)	11.2 (6.0–17.8)	17.4 (7.7–26.1)	17.4 (7.7–26.1)	3.1 (1.5–5.2)	3.8 (1.9–6.0)	9.8 (5.0–14.5)	4.2 (1.1–7.5)	0.9 (<0.1–3.7)	0.9 (<0.1–3.7)	<0.1 (<0.1–1.9)	<0.1 (<0.1–1.9)
Senegal	33.0 (20.2–45.7)	38.1 (25.5–49.4)	14.3 (9.7–18.9)	0.9 (0.4–1.5)	14.7 (7.8–23.6)	12.3 (6.1–20.6)	18.8 (8.4–28.1)	18.8 (8.4–28.1)	8.2 (4.7–11.9)	11.0 (6.4–15.9)	1.4 (0.5–2.4)	0.4 (<0.1–0.9)	4.2 (<0.1–30.1)	4.2 (<0.1–30.1)	0.1 (<0.1–2.2)	0.1 (<0.1–2.2)
Sierra Leone	42.5 (27.4–54.9)	47.0 (33.7–58.2)	15.4 (9.5–21.8)	3.9 (1.8–6.6)	10.2 (4.4–18.2)	8.1 (3.6–15.0)	22.2 (10.2–32.6)	22.2 (10.2–32.6)	4.2 (1.6–7.7)	6.7 (2.7–12.0)	7.4 (3.0–11.6)	2.4 (0.2–5.0)	1.8 (<0.1–4.3)	1.8 (<0.1–4.3)	0.2 (<0.1–1.5)	0.2 (<0.1–1.5)
Togo	33.0 (20.5–47.0)	39.2 (26.9–51.3)	13.0 (9.1–17.3)	1.6 (0.9–2.4)	12.8 (6.9–20.2)	10.7 (5.7–17.2)	22.0 (10.1–32.5)	22.0 (10.1–32.5)	6.4 (3.8–9.3)	9.8 (5.9–14.0)	5.0 (1.7–8.6)	1.5 (0.0–3.4)	2.7 (<0.1–13.0)	2.7 (<0.1–13.0)	0.1 (<0.1–0.9)	0.1 (<0.1–0.9)

Appendix Table 10. Population-attributable fractions of lower respiratory infections due to evaluated risk factors among people aged 50 to 69 years in 2019, males (M) and females (F) (95% UI)

	Smoking		Household air pollution		Ambient particulate matter		Handwashing		Secondhand smoke		Alcohol use		High temperature		Low temperature	
	M	F	M	F	M	F	M	F	M	F	M	F	M	F	M	F
**Global**	**30.5 (24.1–36.9)**	**7.9 (5.5–10.5)**	**13.1 (8.0–19.0)**	**18.2 (12.5–24.5)**	**13.9 (9.8–18.4)**	**14.4 (10.4–18.9)**	**10.3 (4.6–15.8)**	**11.3 (5.0–17.2)**	**6.0 (3.5–8.7)**	**8.8 (5.1–12.8)**	**8.9 (4.5–12.5)**	**1.8 (0.5–3.1)**	**3.0 (1.3–6.4)**	**3.8 (1.7–7.7)**	**5.9 (3.4–8.4)**	**4.7 (2.2–7.3)**
**Central Europe, Eastern Europe, and Central Asia**	**45.7 (37.9–52.9)**	**16.8 (12.1–21.6)**	**0.6 (0.2–1.4)**	**1.3 (0.5–2.7)**	**7.7 (4.5–12.1)**	**8.6 (5.0–12.9)**	**2.3 (0.9–3.8)**	**2.4 (1.0–3.9)**	**7.0 (4.2–10.0)**	**12.6 (7.9–17.4)**	**16.2 (9.0–22.4)**	**3.9 (0.9–6.9)**	**0.1 (<0.1–0.4)**	**0.1 (<0.1–0.5)**	**18.1 (7.7–28.6)**	**18.3 (9.3–28.4)**
Central Asia	40.4 (32.7–47.7)	5.0 (3.1–7.2)	2.1 (0.8–4.2)	3.5 (1.6–6.6)	13.5 (7.5–21.1)	14.1 (7.8–22.4)	4.2 (1.7–6.9)	4.5 (1.8–7.2)	7.3 (4.4–10.4)	12.1 (7.5–16.7)	8.5 (3.7–13.0)	1.1 (0.0–2.6)	0.6 (<0.1–1.8)	0.7 (<0.1–1.8)	18.2 (12.8–25.0)	18.3 (13.0–24.7)
Armenia	49.9 (41.7–57.6)	5.5 (3.2–8.3)	0.3 (0.1–0.8)	0.5 (0.2–1.3)	16.8 (9.1–26.9)	16.8 (9.2–26.9)	3.8 (1.5–6.2)	3.8 (1.5–6.2)	8.8 (5.4–12.6)	17.8 (11.7–24.2)	4.9 (0.7–9.0)	2.7 (0.6–5.0)	<0.1 (<0.1–0.2)	<0.1 (<0.1–0.2)	18.4 (8.4–31.6)	18.4 (8.4–31.6)
Azerbaijan	48.9 (41.4–56.3)	2.6 (1.4–4.2)	0.9 (0.3–2.0)	1.3 (0.4–2.9)	12.9 (6.1–22.6)	12.9 (6.2–22.4)	3.5 (1.4–5.7)	3.5 (1.4–5.7)	9.8 (6.4–13.8)	18.9 (12.7–25.2)	12.0 (5.4–18.1)	2.7 (0.3–5.6)	0.5 (<0.1–1.1)	0.5 (<0.1–1.1)	15.7 (12.6–19.1)	15.7 (12.6–19.1)
Georgia	47.0 (39.1–54.5)	8.6 (5.7–12.2)	3.9 (1.2–8.5)	5.8 (2.1–12.1)	9.1 (5.3–14.5)	9.0 (5.3–13.7)	3.3 (1.3–5.4)	3.3 (1.3–5.4)	10.5 (6.5–15.0)	17.7 (11.8–23.9)	12.7 (6.2–18.3)	0.4 (0.0–1.7)	<0.1 (<0.1–0.2)	<0.1 (<0.1–0.2)	18.9 (11.2–29.0)	18.9 (11.2–29.0)
Kazakhstan	43.6 (35.2–51.6)	7.3 (4.2–11.0)	1.2 (0.3–2.8)	1.8 (0.6–4.2)	10.3 (5.4–16.8)	10.3 (5.4–16.6)	3.4 (1.4–5.7)	3.4 (1.4–5.7)	6.4 (3.7–9.4)	12.7 (7.5–18.0)	9.7 (4.0–15.3)	2.2 (0.0–5.1)	0.5 (<0.1–2.0)	0.5 (<0.1–2.0)	18.4 (11.3–27.6)	18.4 (11.3–27.6)
Kyrgyzstan	48.2 (40.2–55.8)	7.1 (4.8–10.0)	5.4 (2.2–10.4)	7.9 (3.6–14.5)	11.8 (6.3–19.4)	11.3 (6.2–18.4)	4.0 (1.6–6.5)	4.0 (1.6–6.5)	8.7 (5.6–12.3)	17.6 (11.7–23.7)	10.4 (5.1–15.2)	1.4 (0.1–2.8)	<0.1 (<0.1–<0.1)	<0.1 (<0.1–<0.1)	21.4 (8.4–37.4)	21.4 (8.4–37.4)
Mongolia	41.3 (32.7–49.4)	7.1 (4.2–10.4)	6.5 (2.6–12.6)	9.5 (4.3–16.9)	17.3 (10.9–24.5)	16.6 (10.7–23.5)	8.5 (3.6–13.6)	8.5 (3.6–13.6)	5.9 (3.3–8.8)	12.6 (7.2–18.3)	12.2 (6.1–18.2)	1.2 (0.0–3.3)	0.2 (<0.1–0.9)	0.2 (<0.1–0.9)	18.3 (3.6–34.9)	18.3 (3.6–34.9)
Tajikistan	34.1 (27.0–42.4)	4.0 (2.4–6.2)	7.5 (3.2–14.0)	10.7 (5.3–18.2)	16.9 (8.1–28.7)	15.9 (7.7–27.1)	7.1 (2.9–11.3)	7.1 (2.9–11.3)	8.4 (5.3–12.0)	10.3 (6.6–14.4)	4.9 (1.3–8.8)	0.3 (0.0–1.1)	0.3 (0.1–0.5)	0.3 (0.1–0.5)	21.4 (9.3–34.9)	21.4 (9.3–34.9)
Turkmenistan	38.0 (30.5–45.7)	7.7 (4.6–11.5)	<0.1 (<0.1–<0.1)	<0.1 (<0.1–0.1)	13.3 (6.3–23.0)	13.3 (6.3–23.0)	4.6 (1.9–7.4)	4.6 (1.9–7.4)	12.7 (8.4–17.7)	18.9 (12.7–25.6)	10.2 (4.3–15.4)	0.8 (0.0–2.6)	2.6 (0.9–4.3)	2.6 (0.9–4.3)	12.7 (10.0–15.4)	12.7 (10.0–15.4)
Uzbekistan	33.7 (26.1–41.0)	3.5 (2.0–5.5)	1.6 (0.6–3.5)	2.4 (0.9–5.0)	16.9 (8.4–28.0)	16.7 (8.4–27.5)	4.5 (1.8–7.3)	4.5 (1.8–7.3)	6.2 (3.4–9.3)	9.2 (5.4–13.2)	6.2 (2.0–10.4)	0.4 (0.0–1.4)	1.0 (<0.1–2.7)	1.0 (<0.1–2.7)	18.0 (13.9–22.0)	18.0 (13.9–22.0)
Central Europe	40.6 (33.1–47.9)	27.0 (20.2–34.2)	1.0 (0.3–2.5)	1.6 (0.5–3.7)	10.0 (6.1–15.0)	10.0 (6.1–14.9)	1.0 (0.4–1.7)	1.0 (0.4–1.7)	8.3 (4.9–12.0)	9.8 (5.9–13.7)	19.4 (11.4–26.0)	3.5 (0.6–6.7)	<0.1 (<0.1–0.1)	<0.1 (<0.1–0.1)	21.0 (14.7–28.6)	21.0 (14.7–28.6)
Albania	45.9 (38.2–53.5)	12.0 (7.6–17.3)	3.8 (1.2–8.2)	5.6 (2.0–11.7)	9.5 (5.8–14.4)	9.4 (5.8–14.0)	1.7 (0.7–2.9)	1.7 (0.7–2.9)	10.1 (6.4–14.7)	16.1 (10.5–21.8)	9.4 (4.0–15.1)	1.2 (0.0–2.9)	<0.1 (<0.1–0.1)	<0.1 (<0.1–0.1)	19.2 (14.6–24.1)	19.2 (14.6–24.1)

	Smoking		Household air pollution		Ambient particulate matter		Handwashing		Secondhand smoke		Alcohol use		High temperature		Low temperature	
	M	F	M	F	M	F	M	F	M	F	M	F	M	F	M	F
Bosnia and Herzegovina	49.2 (41.1–56.9)	32.6 (25.8–40.3)	3.8 (1.2–8.5)	5.6 (2.0–11.7)	14.6 (9.3–20.5)	14.3 (9.3–20.0)	1.3 (0.5–2.1)	1.3 (0.5–2.1)	11.3 (7.0–15.9)	14.4 (9.2–20.1)	14.1 (7.4–19.6)	1.3 (0.0–3.1)	<0.1 (<0.1–<0.1)	<0.1 (<0.1–<0.1)	22.3 (15.8–29.9)	22.3 (15.8–29.9)
Bulgaria	47.2 (39.1–55.0)	30.9 (23.7–38.1)	1.6 (0.4–4.2)	2.5 (0.7–5.8)	10.0 (6.2–14.9)	9.9 (6.2–14.7)	1.1 (0.4–1.8)	1.1 (0.4–1.8)	9.2 (5.7–13.4)	12.5 (7.7–17.3)	20.0 (12.3–26.8)	5.2 (1.4–9.1)	<0.1 (<0.1–<0.1)	<0.1 (<0.1–<0.1)	21.3 (14.8–27.9)	21.3 (14.8–27.9)
Croatia	40.7 (32.7–48.8)	32.7 (24.7–40.5)	0.4 (0.1–1.1)	0.6 (0.2–1.7)	9.4 (5.7–14.3)	9.4 (5.7–14.2)	1.0 (0.4–1.6)	1.0 (0.4–1.6)	13.1 (8.0–18.3)	13.1 (8.5–18.5)	16.2 (8.3–23.0)	3.7 (0.0–8.0)	<0.1 (<0.1–0.1)	<0.1 (<0.1–0.1)	20.6 (13.3–27.4)	20.6 (13.3–27.4)
Czechia	39.9 (32.7–47.4)	26.2 (19.2–33.2)	0.1 (<0.1–0.2)	0.1 (<0.1–0.4)	8.4 (5.0–13.0)	8.4 (5.0–13.0)	0.9 (0.3–1.4)	0.9 (0.3–1.4)	8.9 (5.5–12.8)	8.5 (5.2–11.9)	21.1 (12.7–28.5)	9.8 (4.3–15.2)	<0.1 (<0.1–0.2)	<0.1 (<0.1–0.2)	21.1 (13.6–30.0)	21.1 (13.6–30.0)
Hungary	40.6 (32.6–48.5)	33.0 (25.4–40.7)	1.3 (0.3–3.6)	2.0 (0.5–5.4)	8.4 (5.0–12.9)	8.4 (5.1–12.8)	0.9 (0.4–1.4)	0.9 (0.4–1.4)	10.7 (6.6–15.2)	11.7 (7.4–16.5)	19.6 (11.5–26.6)	3.2 (0.0–6.9)	<0.1 (<0.1–0.2)	<0.1 (<0.1–0.2)	21.4 (11.9–30.6)	21.4 (11.9–30.6)
Montenegro	49.0 (40.4–56.5)	40.6 (31.8–49.0)	2.7 (0.7–6.4)	4.0 (1.1–9.1)	10.9 (6.8–16.1)	10.8 (6.8–15.8)	1.2 (0.5–2.0)	1.2 (0.5–2.0)	13.1 (8.4–18.3)	13.4 (8.6–18.7)	16.6 (8.9–23.4)	3.2 (0.0–7.4)	<0.1 (<0.1–0.1)	<0.1 (<0.1–0.1)	20.4 (14.1–28.1)	20.4 (14.1–28.1)
North Macedonia	47.9 (40.3–55.5)	30.6 (23.0–38.3)	2.4 (0.7–5.6)	3.6 (1.2–7.8)	15.2 (9.7–21.6)	15.0 (9.7–21.2)	1.0 (0.4–1.7)	1.0 (0.4–1.7)	11.9 (7.4–16.4)	14.4 (9.2–19.8)	15.3 (7.8–21.7)	0.8 (0.0–3.0)	<0.1 (<0.1–0.1)	<0.1 (<0.1–0.1)	21.8 (15.6–28.1)	21.8 (15.6–28.1)
Poland	39.3 (32.0–46.9)	31.2 (23.6–38.7)	0.9 (0.2–2.4)	1.4 (0.4–3.4)	11.6 (7.2–17.1)	11.6 (7.3–17.0)	1.0 (0.4–1.6)	1.0 (0.4–1.6)	7.1 (4.1–10.6)	8.5 (5.1–12.0)	20.2 (11.8–26.9)	1.6 (0.0–4.4)	<0.1 (<0.1–0.2)	<0.1 (<0.1–0.2)	21.1 (13.2–30.1)	21.1 (13.2–30.0)
Romania	40.5 (32.3–48.3)	19.9 (13.1–26.3)	1.1 (0.3–2.7)	1.6 (0.4–4.1)	7.9 (4.7–12.1)	7.9 (4.8–12.1)	1.2 (0.5–2.0)	1.2 (0.5–2.0)	8.3 (4.9–12.3)	10.2 (6.0–14.7)	18.9 (10.7–25.7)	4.3 (0.6–8.4)	<0.1 (<0.1–0.1)	<0.1 (<0.1–0.1)	20.7 (14.2–28.2)	20.7 (14.2–28.2)
Serbia	45.3 (37.5–52.9)	34.9 (26.2–42.9)	1.9 (0.5–4.8)	2.9 (0.9–6.9)	13.0 (8.2–19.2)	12.9 (8.2–18.9)	1.0 (0.4–1.6)	1.0 (0.4–1.6)	10.3 (6.0–14.6)	10.8 (6.5–15.4)	16.4 (8.7–23.0)	2.5 (0.3–5.0)	<0.1 (<0.1–0.1)	<0.1 (<0.1–0.1)	21.9 (14.8–28.9)	21.9 (14.8–28.9)
Slovakia	34.1 (26.0–42.3)	19.2 (12.8–26.0)	0.1 (<0.1–0.2)	0.1 (<0.1–0.3)	9.4 (5.5–14.5)	9.4 (5.5–14.4)	0.9 (0.3–1.4)	0.9 (0.3–1.4)	8.5 (5.0–12.7)	8.8 (5.3–12.6)	19.6 (11.2–26.3)	2.5 (0.0–6.2)	<0.1 (<0.1–0.1)	<0.1 (<0.1–0.1)	20.2 (13.2–29.4)	20.2 (13.2–29.4)
Slovenia	38.5 (30.9–46.1)	30.5 (23.5–37.3)	0.5 (0.1–1.5)	0.8 (0.2–2.2)	8.6 (5.3–13.3)	8.6 (5.3–13.2)	0.8 (0.3–1.4)	0.8 (0.3–1.4)	11.2 (6.9–15.7)	10.1 (6.5–14.3)	10.8 (1.2–20.2)	1.7 (0.0–5.9)	<0.1 (<0.1–0.1)	<0.1 (<0.1–0.1)	22.1 (15.2–30.1)	22.1 (15.2–30.1)
Eastern Europe	48.6 (41.1–56.0)	15.6 (10.9–20.5)	0.2 (0.1–0.5)	0.3 (0.1–0.7)	5.6 (2.9–9.7)	5.5 (2.9–9.5)	2.4 (1.0–3.9)	2.3 (0.9–3.8)	6.4 (3.8–9.2)	14.3 (9.2–19.7)	16.6 (9.0–23.1)	5.3 (1.6–9.1)	<0.1 (<0.1–0.3)	<0.1 (<0.1–0.3)	16.9 (1.3–30.4)	16.8 (0.4–30.8)
Belarus	45.4 (36.7–53.1)	15.2 (10.5–20.6)	0.1 (<0.1–0.2)	0.1 (<0.1–0.3)	8.2 (4.7–13.0)	8.2 (4.7–13.0)	1.9 (0.8–3.1)	1.9 (0.8–3.1)	5.3 (3.1–7.7)	13.5 (8.7–18.6)	20.3 (11.6–27.5)	6.1 (1.5–10.9)	<0.1 (<0.1–0.2)	<0.1 (<0.1–0.2)	18.3 (9.6–30.8)	18.3 (9.6–30.8)
Estonia	44.3 (36.8–52.2)	24.7 (17.5–32.1)	0.4 (<0.1–1.2)	0.6 (0.1–1.9)	1.7 (0.5–3.5)	1.9 (0.7–3.7)	1.8 (0.7–3.0)	1.8 (0.7–3.0)	6.9 (4.0–9.9)	9.6 (5.9–13.5)	21.1 (11.9–28.1)	5.9 (0.7–10.9)	0.1 (<0.1–0.5)	0.1 (<0.1–0.5)	15.8 (3.4–32.6)	15.8 (3.4–32.6)
Latvia	43.1 (34.7–51.7)	18.5 (11.8–25.6)	0.4 (0.1–1.1)	0.6 (0.1–1.7)	5.6 (3.1–9.1)	5.6 (3.1–9.1)	1.9 (0.8–3.1)	1.9 (0.8–3.1)	5.2 (2.9–7.9)	10.9 (6.4–15.9)	19.7 (11.2–26.6)	4.1 (0.0–8.5)	<0.1 (<0.1–0.3)	<0.1 (<0.1–0.3)	17.2 (7.2–31.3)	17.2 (7.2–31.3)
Lithuania	39.1 (30.4–47.2)	15.4 (10.2–21.4)	0.2 (<0.1–0.5)	0.3 (<0.1–0.7)	4.5 (2.3–7.6)	4.5 (2.3–7.6)	1.9 (0.8–3.2)	1.9 (0.8–3.2)	5.4 (3.1–8.0)	9.6 (5.7–13.9)	19.3 (11.1–26.3)	7.2 (1.7–13.0)	<0.1 (<0.1–0.2)	<0.1 (<0.1–0.2)	19.1 (9.2–31.0)	19.1 (9.2–31.0)
Republic of Moldova	46.4 (38.2–54.1)	7.7 (5.1–11.0)	0.7 (0.2–1.6)	1.1 (0.4–2.4)	6.7 (3.3–11.9)	6.7 (3.4–11.9)	4.6 (1.9–7.4)	4.6 (1.9–7.4)	6.3 (4.0–9.0)	13.2 (8.7–18.1)	17.4 (9.3–24.0)	5.4 (1.1–9.8)	<0.1 (<0.1–0.1)	<0.1 (<0.1–0.1)	22.1 (13.3–30.8)	22.1 (13.3–30.8)

	Smoking		Household air pollution		Ambient particulate matter		Handwashing		Secondhand smoke		Alcohol use		High temperature		Low temperature	
	M	F	M	F	M	F	M	F	M	F	M	F	M	F	M	F
Russian Federation	49.6 (42.1–56.9)	16.3 (11.4–21.4)	0.1 (<0.1–0.4)	0.2 (0.1–0.6)	5.2 (2.6–9.0)	5.2 (2.6–9.0)	2.2 (0.9–3.5)	2.2 (0.9–3.6)	6.5 (3.8–9.4)	14.6 (9.5–20.0)	17.1 (9.5–23.6)	5.2 (1.4–9.2)	<0.1 (<0.1–0.4)	<0.1 (<0.1–0.4)	16.1 (<0.1–32.2)	16.1 (<0.1–32.3)
Ukraine	46.1 (37.6–53.9)	13.0 (8.3–18.3)	0.4 (0.1–0.9)	0.5 (0.2–1.3)	7.1 (3.7–12.3)	7.1 (3.7–12.3)	3.1 (1.2–5.0)	3.1 (1.2–5.0)	6.4 (3.8–9.4)	13.5 (8.7–18.8)	13.5 (6.4–20.4)	5.7 (1.7–10.1)	<0.1 (<0.1–0.2)	<0.1 (<0.1–0.2)	19.7 (13.3–26.3)	19.7 (13.3–26.2)
**High-income**	**35.0 (28.4–41.6)**	**24.8 (19.1–30.6)**	**0.1 (<0.1–0.1)**	**0.1 (<0.1–0.2)**	**5.2 (2.9–8.5)**	**4.6 (2.5–7.7)**	**1.4 (0.6–2.4)**	**1.4 (0.5–2.3)**	**7.1 (4.2–10.0)**	**7.2 (4.4–10.1)**	**13.6 (6.8–19.2)**	**6.6 (2.4–10.6)**	**0.5 (0.2–0.9)**	**0.6 (0.3–0.9)**	**17.2 (12.9–22.1)**	**17.0 (12.7–22.1)**
Australasia	27.2 (21.0–33.8)	22.0 (16.2–28.0)	<0.1 (<0.1–<0.1)	<0.1 (<0.1–0.1)	1.9 (0.4–4.2)	1.9 (0.4–4.2)	1.9 (0.8–3.2)	1.9 (0.8–3.2)	6.3 (3.6–9.4)	5.6 (3.1–8.3)	17.9 (10.2–24.4)	7.3 (1.8–12.3)	6.1 (4.1–8.1)	6.3 (4.2–8.5)	8.1 (5.2–11.2)	7.8 (4.8–10.7)
Australia	27.8 (21.7–34.4)	22.3 (16.4–28.2)	<0.1 (<0.1–<0.1)	<0.1 (<0.1–0.1)	1.9 (0.5–4.3)	1.9 (0.5–4.3)	1.9 (0.8–3.2)	1.9 (0.8–3.2)	6.3 (3.5–9.4)	5.7 (3.2–8.4)	18.3 (10.5–25.3)	7.0 (1.6–12.3)	7.3 (4.8–9.7)	7.3 (4.8–9.7)	5.9 (2.7–8.9)	5.9 (2.7–8.9)
New Zealand	23.7 (17.3–30.3)	20.5 (14.2–27.2)	<0.1 (<0.1–<0.1)	<0.1 (<0.1–0.1)	1.5 (0.2–3.5)	1.5 (0.3–3.5)	1.9 (0.8–3.2)	2.0 (0.8–3.2)	6.2 (3.4–9.3)	5.1 (2.8–7.6)	15.9 (8.1–22.0)	8.7 (2.9–13.8)	<0.1 (<0.1–<0.1)	<0.1 (<0.1–<0.1)	20.6 (13.5–28.0)	20.6 (13.4–27.8)
High-income Asia Pacific	39.0 (31.4–46.3)	12.3 (8.9–16.0)	<0.1 (<0.1–<0.1)	<0.1 (<0.1–0.1)	8.0 (4.6–12.9)	8.0 (4.5–12.8)	1.9 (0.7–3.1)	1.9 (0.7–3.1)	6.2 (3.6–9.0)	9.3 (5.6–13.2)	10.5 (4.3–16.5)	4.9 (1.3–8.6)	0.5 (<0.1–1.3)	0.5 (<0.1–1.4)	16.0 (12.7–19.5)	15.4 (12.2–18.7)
Brunei Darussalam	31.7 (24.9–39.1)	8.7 (5.3–13.9)	<0.1 (<0.1–0.1)	0.1 (<0.1–0.2)	2.6 (0.8–5.4)	2.6 (0.8–5.4)	2.0 (0.8–3.2)	2.0 (0.8–3.2)	5.7 (3.3–8.5)	7.8 (4.5–11.2)	0.2 (0.0–1.2)	0.1 (0.0–0.5)	0.9 (<0.1–5.8)	0.9 (<0.1–5.8)	<0.1 (<0.1–0.5)	<0.1 (<0.1–0.5)
Japan	39.6 (32.0–46.9)	14.4 (10.3–18.8)	<0.1 (<0.1–<0.1)	<0.1 (<0.1–0.1)	6.4 (3.4–10.4)	6.4 (3.4–10.4)	1.9 (0.7–3.1)	1.9 (0.7–3.1)	6.5 (3.7–9.6)	9.7 (5.8–13.8)	9.5 (3.3–16.1)	4.5 (0.7–8.6)	0.5 (<0.1–1.0)	0.5 (<0.1–1.0)	16.1 (12.9–19.5)	16.1 (13.0–19.6)
Republic of Korea	40.9 (33.1–48.3)	6.2 (3.9–9.0)	<0.1 (<0.1–<0.1)	<0.1 (<0.1–<0.1)	14.2 (8.6–21.1)	14.2 (8.6–21.1)	1.9 (0.7–3.1)	1.9 (0.7–3.1)	5.7 (3.3–8.5)	9.6 (5.7–13.8)	16.8 (9.3–23.6)	8.8 (3.7–13.8)	0.2 (<0.1–0.9)	0.2 (<0.1–0.9)	20.4 (14.7–25.9)	20.4 (14.7–25.9)
Singapore	25.5 (18.8–32.8)	7.7 (4.9–11.1)	<0.1 (<0.1–<0.1)	<0.1 (<0.1–0.1)	9.5 (5.0–16.2)	9.5 (5.0–16.2)	1.9 (0.8–3.1)	1.9 (0.8–3.1)	4.6 (2.4–7.1)	5.5 (3.0–8.2)	2.5 (0.0–5.9)	0.9 (0.0–2.5)	1.3 (<0.1–11.7)	1.3 (<0.1–11.7)	0.1 (<0.1–1.2)	0.1 (<0.1–1.2)
High-income North America	31.7 (25.5–38.0)	25.6 (20.1–31.2)	<0.1 (<0.1–<0.1)	<0.1 (<0.1–<0.1)	2.6 (1.1–4.9)	2.6 (1.2–4.9)	1.0 (0.4–1.6)	1.0 (0.4–1.6)	7.0 (4.2–10.0)	6.1 (3.9–8.7)	11.7 (6.0–17.0)	6.2 (2.1–9.9)	0.6 (0.3–1.0)	0.6 (0.3–1.0)	15.6 (11.7–19.7)	15.4 (11.7–19.5)
Canada	30.8 (24.2–37.7)	25.9 (19.9–32.3)	<0.1 (<0.1–<0.1)	<0.1 (<0.1–<0.1)	2.2 (0.9–4.4)	2.2 (0.9–4.4)	1.0 (0.4–1.7)	1.0 (0.4–1.7)	7.1 (4.4–10.6)	6.0 (3.7–8.6)	13.2 (6.6–19.1)	6.8 (2.1–11.6)	<0.1 (<0.1–0.3)	<0.1 (<0.1–0.3)	20.8 (0.8–39.3)	20.8 (0.8–39.3)
United States of America	31.8 (25.6–38.2)	25.6 (20.0–31.2)	<0.1 (<0.1–<0.1)	<0.1 (<0.1–<0.1)	2.6 (1.2–4.9)	2.6 (1.2–5.0)	1.0 (0.4–1.6)	1.0 (0.4–1.6)	7.0 (4.2–10.0)	6.1 (3.9–8.8)	11.7 (5.9–16.7)	6.1 (2.1–9.9)	0.6 (0.3–1.0)	0.7 (0.3–1.0)	15.2 (11.7–18.9)	15.1 (11.6–18.7)
Southern Latin America	34.7 (27.8–41.7)	29.5 (22.1–37.3)	0.3 (0.1–0.8)	0.5 (0.1–1.1)	6.9 (3.7–11.5)	6.8 (3.7–11.3)	2.4 (1.0–4.0)	2.5 (1.0–4.1)	8.1 (4.6–12.0)	8.3 (4.7–12.2)	14.8 (7.5–21.5)	6.3 (1.8–11.2)	0.8 (0.5–1.3)	0.9 (0.5–1.3)	16.2 (10.3–22.6)	16.1 (10.3–22.4)
Argentina	34.9 (27.8–41.9)	29.8 (22.4–37.4)	0.3 (0.1–0.8)	0.4 (0.1–1.1)	6.5 (3.4–11.1)	6.5 (3.4–11.0)	2.5 (1.0–4.1)	2.5 (1.0–4.1)	8.0 (4.6–12.0)	8.3 (4.7–12.3)	14.9 (7.4–21.7)	6.3 (1.7–11.4)	0.9 (0.5–1.4)	0.9 (0.5–1.4)	16.1 (10.5–22.4)	16.1 (10.5–22.4)
Chile	32.5 (23.5–40.7)	26.5 (18.1–34.8)	0.5 (0.1–1.3)	0.8 (0.2–2.1)	11.7 (7.1–18.0)	11.7 (7.1–17.9)	2.0 (0.8–3.4)	2.0 (0.8–3.4)	8.1 (4.2–12.8)	7.7 (3.8–12.0)	14.7 (7.2–21.3)	6.7 (2.1–11.6)	<0.1 (<0.1–<0.1)	<0.1 (<0.1–<0.1)	19.3 (10.4–29.4)	19.3 (10.4–29.4)
Uruguay	37.6 (30.3–45.1)	26.5 (19.7–33.5)	0.2 (0.1–0.6)	0.4 (0.1–0.9)	4.0 (1.7–7.6)	4.0 (1.8–7.6)	2.3 (0.9–3.7)	2.3 (0.9–3.7)	9.3 (5.4–13.7)	9.8 (5.8–14.1)	12.9 (6.2–19.3)	6.2 (2.2–10.5)	0.1 (<0.1–0.3)	0.1 (<0.1–0.3)	8.0 (4.5–11.6)	8.0 (4.5–11.6)

	Smoking		Household air pollution		Ambient particulate matter		Handwashing		Secondhand smoke		Alcohol use		High temperature		Low temperature	
	M	F	M	F	M	F	M	F	M	F	M	F	M	F	M	F
Western Europe	35.7 (28.7–42.6)	26.5 (20.1–33.1)	<0.1 (<0.1–<0.1)	<0.1 (<0.1–0.1)	4.9 (2.7–8.2)	4.9 (2.7–8.1)	1.0 (0.4–1.7)	1.0 (0.4–1.7)	7.3 (4.4–10.5)	7.1 (4.3–10.0)	17.5 (9.7–23.6)	8.4 (3.5–12.8)	0.1 (<0.1–0.2)	0.1 (<0.1–0.2)	21.1 (15.1–28.0)	21.5 (15.1–28.6)
Andorra	36.3 (28.0–44.7)	24.4 (16.7–33.0)	<0.1 (<0.1–<0.1)	<0.1 (<0.1–<0.1)	3.5 (1.7–6.3)	3.5 (1.7–6.3)	1.0 (0.4–1.6)	1.0 (0.4–1.6)	8.1 (4.8–11.9)	8.2 (4.9–11.8)	17.1 (9.1–23.6)	7.6 (2.2–12.5)	<0.1 (<0.1–<0.1)	<0.1 (<0.1–<0.1)	17.0 (1.9–35.8)	17.0 (1.9–35.8)
Austria	43.4 (35.4–50.8)	31.7 (24.9–39.0)	<0.1 (<0.1–0.1)	<0.1 (<0.1–0.1)	5.6 (3.1–9.1)	5.6 (3.1–9.1)	1.0 (0.4–1.6)	1.0 (0.4–1.6)	10.9 (6.9–15.0)	9.3 (5.9–13.1)	18.4 (10.4–25.1)	8.2 (2.8–13.3)	<0.1 (<0.1–0.1)	<0.1 (<0.1–0.1)	19.7 (10.6–31.5)	19.7 (10.6–31.5)
Belgium	38.0 (31.1–45.0)	30.2 (23.9–36.6)	<0.1 (<0.1–<0.1)	<0.1 (<0.1–<0.1)	5.9 (3.4–9.7)	5.9 (3.4–9.7)	1.0 (0.4–1.6)	1.0 (0.4–1.6)	7.8 (4.7–11.3)	7.4 (4.6–10.5)	18.2 (9.6–25.1)	10.4 (4.4–16.1)	<0.1 (<0.1–0.1)	<0.1 (<0.1–0.1)	23.4 (14.7–32.7)	23.4 (14.7–32.7)
Cyprus	46.6 (38.7–54.0)	23.3 (17.0–30.1)	<0.1 (<0.1–<0.1)	<0.1 (<0.1–0.1)	7.7 (4.5–12.5)	7.7 (4.5–12.5)	1.0 (0.4–1.6)	1.0 (0.4–1.6)	8.7 (5.4–12.4)	12.0 (7.4–17.0)	17.4 (9.3–24.2)	3.2 (0.1–6.8)	2.1 (0.9–3.6)	2.1 (0.9–3.6)	4.5 (<0.1–10.9)	4.5 (<0.1–10.9)
Denmark	37.1 (30.0–44.2)	31.3 (24.7–37.8)	<0.1 (<0.1–<0.1)	<0.1 (<0.1–<0.1)	4.0 (2.1–7.2)	4.0 (2.1–7.2)	1.0 (0.4–1.6)	1.0 (0.4–1.6)	7.4 (4.5–10.7)	6.5 (4.0–9.3)	15.2 (7.4–21.9)	9.4 (3.4–15.6)	<0.1 (<0.1–<0.1)	<0.1 (<0.1–<0.1)	23.2 (13.0–34.4)	23.2 (13.0–34.4)
Finland	32.3 (25.5–39.2)	23.1 (17.3–29.5)	<0.1 (<0.1–<0.1)	<0.1 (<0.1–<0.1)	1.2 (0.2–2.9)	1.2 (0.2–2.9)	1.0 (0.4–1.7)	1.0 (0.4–1.7)	5.2 (3.0–7.9)	4.9 (2.8–7.2)	15.0 (7.9–21.5)	5.0 (0.3–9.8)	<0.1 (<0.1–0.3)	<0.1 (<0.1–0.3)	17.9 (3.8–36.4)	17.9 (3.8–36.4)
France	35.2 (27.9–42.5)	24.1 (17.3–31.6)	<0.1 (<0.1–<0.1)	<0.1 (<0.1–<0.1)	5.1 (2.8–8.5)	5.1 (2.8–8.5)	1.0 (0.4–1.7)	1.0 (0.4–1.7)	5.2 (2.9–8.0)	5.1 (2.8–7.6)	19.5 (10.7–27.0)	10.2 (4.1–16.0)	<0.1 (<0.1–0.1)	<0.1 (<0.1–0.1)	21.7 (14.9–28.4)	21.7 (14.9–28.4)
Germany	36.7 (29.2–43.7)	26.4 (19.9–33.2)	<0.1 (<0.1–<0.1)	<0.1 (<0.1–<0.1)	5.3 (3.0–8.8)	5.3 (3.0–8.8)	1.0 (0.4–1.6)	1.0 (0.4–1.6)	7.0 (4.0–10.3)	6.5 (3.8–9.5)	21.7 (13.2–28.9)	14.0 (7.2–20.1)	<0.1 (<0.1–0.2)	<0.1 (<0.1–0.2)	22.5 (14.3–31.1)	22.5 (14.3–31.1)
Greece	47.8 (40.1–55.3)	36.1 (28.0–43.7)	<0.1 (<0.1–0.1)	0.1 (<0.1–0.2)	6.9 (4.0–10.9)	6.9 (4.0–10.9)	1.0 (0.4–1.7)	1.0 (0.4–1.7)	10.4 (6.3–15.0)	11.9 (7.5–16.5)	16.8 (9.4–23.4)	2.1 (0.0–5.4)	0.1 (<0.1–0.3)	0.1 (<0.1–0.3)	15.7 (12.3–19.4)	15.7 (12.3–19.4)
Iceland	31.1 (24.2–38.2)	24.7 (18.6–31.3)	<0.1 (<0.1–<0.1)	<0.1 (<0.1–<0.1)	1.2 (0.2–3.0)	1.2 (0.2–3.0)	1.0 (0.4–1.7)	1.0 (0.4–1.7)	9.7 (6.3–14.0)	8.6 (5.6–12.2)	13.4 (6.4–19.7)	5.4 (0.6–10.2)	<0.1 (0.0–0.0)	<0.1 (0.0–0.0)	21.5 (5.1–38.7)	21.5 (5.1–38.7)
Ireland	30.3 (23.6–36.9)	26.1 (19.3–33.1)	<0.1 (<0.1–<0.1)	<0.1 (<0.1–<0.1)	2.7 (1.2–5.1)	2.7 (1.2–5.1)	1.0 (0.4–1.6)	1.0 (0.4–1.6)	7.0 (3.9–10.4)	6.4 (3.7–9.3)	15.3 (8.2–21.6)	7.1 (2.1–12.1)	<0.1 (0.0–0.0)	<0.1 (0.0–0.0)	25.7 (14.4–37.2)	25.7 (14.4–37.2)
Israel	34.6 (27.9–41.6)	20.3 (14.7–26.4)	<0.1 (<0.1–<0.1)	<0.1 (<0.1–0.1)	10.1 (6.1–15.3)	10.1 (6.1–15.3)	1.0 (0.4–1.7)	1.0 (0.4–1.7)	7.2 (4.0–10.7)	6.6 (3.6–9.6)	3.9 (0.0–8.1)	0.9 (0.0–2.8)	2.1 (0.8–3.9)	2.1 (0.8–3.9)	3.5 (<0.1–10.4)	3.5 (<0.1–10.4)
Italy	34.1 (27.1–41.0)	23.6 (17.0–30.2)	<0.1 (<0.1–0.1)	<0.1 (<0.1–0.1)	7.8 (4.6–12.1)	7.8 (4.6–12.1)	1.0 (0.4–1.7)	1.0 (0.4–1.7)	10.0 (5.9–14.2)	9.3 (5.6–13.2)	16.8 (9.2–23.3)	6.7 (2.8–10.6)	0.1 (<0.1–0.2)	0.1 (<0.1–0.2)	17.3 (13.2–21.5)	17.3 (13.1–21.5)
Luxembourg	33.8 (26.1–41.2)	23.4 (16.8–30.5)	<0.1 (<0.1–<0.1)	<0.1 (<0.1–<0.1)	4.2 (2.2–7.3)	4.2 (2.2–7.3)	1.0 (0.4–1.6)	1.0 (0.4–1.6)	7.4 (4.4–10.8)	8.2 (5.0–12.0)	19.5 (10.9–26.5)	8.8 (3.1–14.4)	<0.1 (<0.1–0.1)	<0.1 (<0.1–0.1)	23.1 (13.3–33.6)	23.1 (13.3–33.6)
Malta	37.2 (29.8–44.6)	23.1 (17.1–29.1)	<0.1 (<0.1–<0.1)	<0.1 (<0.1–0.1)	6.1 (3.4–9.9)	6.1 (3.4–9.9)	1.0 (0.4–1.7)	1.0 (0.4–1.7)	8.1 (5.0–11.7)	8.1 (5.0–11.6)	14.1 (7.1–20.5)	4.1 (0.3–7.9)	0.3 (<0.1–0.9)	0.3 (<0.1–0.9)	3.6 (<0.1–15.0)	3.6 (<0.1–15.0)
Monaco	36.2 (27.6–44.4)	26.1 (17.1–35.9)	<0.1 (<0.1–<0.1)	<0.1 (<0.1–<0.1)	5.3 (2.5–9.2)	5.3 (2.5–9.2)	1.0 (0.4–1.6)	1.0 (0.4–1.6)	8.4 (4.9–11.9)	7.9 (4.8–11.4)	9.7 (0.0–23.5)	4.1 (0.0–12.4)	<0.1 (<0.1–0.1)	<0.1 (<0.1–0.1)	15.4 (11.9–19.0)	15.4 (11.9–19.0)
Netherlands	32.4 (25.2–39.5)	27.5 (20.5–34.9)	<0.1 (<0.1–<0.1)	<0.1 (<0.1–<0.1)	5.5 (3.1–9.0)	5.5 (3.1–9.0)	1.0 (0.4–1.6)	1.0 (0.4–1.6)	7.0 (4.0–10.6)	6.3 (3.6–9.6)	15.8 (8.0–22.6)	9.2 (3.6–14.9)	<0.1 (<0.1–0.1)	<0.1 (<0.1–0.1)	23.6 (13.4–33.5)	23.6 (13.4–33.5)

	Smoking		Household air pollution		Ambient particulate matter		Handwashing		Secondhand smoke		Alcohol use		High temperature		Low temperature	
	M	F	M	F	M	F	M	F	M	F	M	F	M	F	M	F
Norway	31.8 (25.2–38.9)	26.6 (19.5–34.0)	<0.1 (<0.1–<0.1)	<0.1 (<0.1–<0.1)	1.8 (0.6–3.9)	1.8 (0.6–3.9)	1.0 (0.4–1.6)	1.0 (0.4–1.6)	6.9 (3.9–10.2)	5.9 (3.4–8.6)	11.6 (5.0–17.7)	4.6 (0.1–9.2)	<0.1 (<0.1–0.1)	<0.1 (<0.1–0.1)	19.1 (5.8–35.9)	19.0 (5.9–35.9)
Portugal	35.4 (27.5–43.0)	14.9 (10.1–20.2)	<0.1 (<0.1–0.1)	0.1 (<0.1–0.2)	3.0 (1.4–5.5)	3.0 (1.4–5.5)	1.1 (0.4–1.8)	1.1 (0.4–1.8)	6.6 (3.7–9.7)	8.4 (4.8–12.1)	19.1 (11.0–25.9)	6.5 (2.6–10.3)	0.2 (<0.1–0.3)	0.2 (<0.1–0.3)	15.3 (11.7–19.2)	15.3 (11.7–19.2)
San Marino	32.9 (25.6–41.0)	24.0 (17.2–32.2)	<0.1 (<0.1–<0.1)	<0.1 (<0.1–<0.1)	4.1 (1.5–7.9)	4.1 (1.5–7.9)	1.0 (0.4–1.7)	1.0 (0.4–1.7)	9.0 (5.6–13.0)	8.9 (5.7–12.9)	15.9 (0.0–25.6)	7.6 (0.0–14.9)	0.1 (<0.1–0.2)	0.1 (<0.1–0.2)	15.7 (12.5–19.0)	15.7 (12.5–19.0)
Spain	42.1 (34.3–49.9)	29.9 (22.1–37.6)	0.1 (<0.1–0.2)	0.1 (<0.1–0.3)	4.0 (2.1–6.9)	4.0 (2.1–6.9)	1.0 (0.4–1.7)	1.0 (0.4–1.7)	8.5 (4.9–12.5)	8.7 (5.3–12.3)	16.3 (8.7–23.1)	5.7 (1.7–9.7)	0.2 (<0.1–0.5)	0.2 (<0.1–0.5)	17.3 (13.3–21.5)	17.3 (13.3–21.5)
Sweden	26.9 (21.0–32.9)	27.9 (21.6–34.4)	<0.1 (<0.1–<0.1)	<0.1 (<0.1–<0.1)	1.2 (0.3–3.0)	1.2 (0.3–3.0)	1.0 (0.4–1.7)	1.0 (0.4–1.7)	7.3 (4.5–10.6)	5.2 (3.2–7.7)	15.6 (7.9–22.4)	11.4 (4.5–17.3)	<0.1 (<0.1–0.1)	<0.1 (<0.1–0.1)	18.5 (5.4–32.9)	18.5 (5.3–33.0)
Switzerland	32.4 (25.6–39.2)	26.0 (19.5–32.4)	<0.1 (<0.1–<0.1)	<0.1 (<0.1–<0.1)	4.1 (2.2–7.0)	4.1 (2.2–7.0)	1.0 (0.4–1.6)	1.0 (0.4–1.6)	7.2 (4.1–10.9)	6.8 (4.0–10.0)	15.9 (7.9–22.6)	11.8 (5.0–18.0)	<0.1 (<0.1–<0.1)	<0.1 (<0.1–<0.1)	21.1 (11.3–32.9)	21.1 (11.3–32.9)
United Kingdom	32.2 (25.3–39.0)	27.0 (20.0–34.0)	<0.1 (<0.1–<0.1)	<0.1 (<0.1–<0.1)	4.1 (2.1–7.0)	4.1 (2.1–7.0)	1.0 (0.4–1.7)	1.0 (0.4–1.7)	7.2 (4.0–10.5)	6.9 (4.0–10.1)	14.2 (7.2–20.6)	5.8 (1.1–10.8)	<0.1 (<0.1–<0.1)	<0.1 (<0.1–<0.1)	24.8 (15.9–34.9)	24.8 (15.8–35.0)
**Latin America and Caribbean**	**23.3 (17.5–29.5)**	**13.5 (9.4–17.7)**	**3.0 (1.4–5.3)**	**4.9 (2.6–7.9)**	**8.3 (5.2–12.6)**	**8.2 (5.3–12.3)**	**6.6 (2.7–10.5)**	**6.8 (2.8–10.8)**	**5.9 (3.2–8.7)**	**5.9 (3.2–8.7)**	**9.9 (5.1–14.2)**	**1.8 (0.2–3.3)**	**1.4 (0.1–4.2)**	**1.4 (<0.1–4.2)**	**2.9 (1.2–4.8)**	**3.0 (1.3–4.8)**
Andean Latin America	14.5 (9.5–19.7)	3.8 (2.0–6.2)	3.5 (1.4–6.8)	5.3 (2.5–9.4)	13.7 (8.4–20.9)	13.3 (8.2–19.9)	7.8 (3.3–12.4)	7.8 (3.3–12.5)	2.7 (1.0–5.1)	2.5 (1.0–4.6)	9.0 (3.2–14.1)	1.1 (0.0–3.5)	0.5 (<0.1–1.3)	0.6 (<0.1–1.4)	9.3 (5.8–13.2)	9.2 (5.8–13.0)
Bolivia (Plurinational State of)	19.6 (13.5–25.7)	4.5 (1.7–7.9)	6.0 (2.5–11.7)	8.7 (4.1–15.1)	12.9 (7.4–20.6)	12.3 (7.2–19.3)	8.4 (3.5–13.2)	8.4 (3.5–13.2)	2.4 (0.8–4.6)	2.3 (0.8–4.3)	7.5 (1.8–13.0)	0.8 (0.0–2.9)	1.5 (0.5–2.7)	1.5 (0.5–2.7)	9.1 (5.9–12.6)	9.1 (5.9–12.6)
Ecuador	20.4 (13.4–26.9)	5.1 (2.8–7.8)	0.9 (0.3–2.1)	1.4 (0.5–3.2)	10.2 (5.4–16.6)	10.2 (5.5–16.4)	6.9 (2.8–11.0)	6.9 (2.8–11.0)	2.5 (0.9–4.8)	2.4 (0.8–4.5)	7.5 (3.5–11.7)	0.6 (0.0–1.8)	0.3 (<0.1–1.2)	0.3 (<0.1–1.2)	5.8 (3.6–8.1)	5.8 (3.6–8.1)
Peru	10.7 (6.5–15.4)	3.2 (1.6–5.2)	3.3 (1.3–6.5)	5.0 (2.2–8.9)	15.2 (9.3–23.1)	14.8 (9.3–22.2)	7.9 (3.3–12.5)	7.9 (3.3–12.5)	2.9 (1.0–5.3)	2.6 (1.0–4.8)	10.1 (3.3–16.3)	1.4 (0.0–4.1)	0.2 (<0.1–0.9)	0.2 (<0.1–0.9)	10.4 (6.0–15.2)	10.4 (6.0–15.2)
Caribbean	27.8 (21.0–34.9)	13.2 (9.0–18.2)	9.2 (4.5–15.6)	15.9 (9.5–23.9)	7.7 (3.7–14.0)	7.0 (3.5–12.5)	11.6 (5.1–18.1)	12.5 (5.5–19.4)	5.8 (3.2–8.5)	5.4 (3.0–8.0)	9.9 (4.9–14.1)	2.2 (0.5–3.9)	1.0 (<0.1–3.2)	1.0 (<0.1–3.2)	<0.1 (<0.1–1.3)	<0.1 (<0.1–1.3)
Antigua and Barbuda	19.7 (14.2–25.6)	8.0 (5.1–12.1)	0.1 (<0.1–0.3)	0.2 (<0.1–0.4)	8.7 (2.7–18.4)	8.7 (2.7–18.4)	7.5 (3.1–11.9)	7.5 (3.1–11.9)	5.9 (3.4–8.8)	5.2 (2.8–7.7)	9.9 (4.6–15.0)	2.4 (0.5–4.6)	0.4 (<0.1–2.3)	0.4 (<0.1–2.3)	0.3 (<0.1–1.7)	0.3 (<0.1–1.7)
Bahamas	18.6 (13.4–23.8)	5.7 (3.5–8.5)	0.1 (<0.1–0.4)	0.2 (<0.1–0.5)	7.6 (1.7–16.5)	7.6 (1.7–16.4)	6.3 (2.6–10.2)	6.3 (2.6–10.2)	6.2 (3.5–9.3)	6.3 (3.7–9.1)	8.1 (0.7–15.1)	1.9 (0.0–4.7)	1.6 (<0.1–4.5)	1.6 (<0.1–4.5)	0.3 (<0.1–2.0)	0.3 (<0.1–2.0)
Barbados	16.1 (11.7–21.2)	5.1 (3.2–7.6)	<0.1 (<0.1–<0.1)	<0.1 (<0.1–<0.1)	10.8 (3.8–21.4)	10.8 (3.8–21.4)	7.1 (2.9–11.4)	7.1 (2.9–11.4)	4.3 (2.3–6.5)	4.1 (2.2–6.2)	14.2 (8.1–19.4)	2.0 (0.0–4.2)	1.2 (<0.1–3.9)	1.2 (<0.1–3.9)	0.4 (<0.1–2.0)	0.4 (<0.1–2.0)
Belize	25.7 (18.5–32.7)	5.6 (3.4–8.3)	2.7 (1.0–5.7)	4.1 (1.7–7.9)	10.6 (3.3–20.9)	10.4 (3.6–20.5)	9.5 (4.0–15.0)	9.5 (4.0–15.0)	4.9 (2.6–7.5)	6.6 (3.6–9.9)	10.7 (5.5–15.3)	1.0 (0.1–2.1)	1.7 (<0.1–4.2)	1.7 (<0.1–4.2)	0.1 (<0.1–1.3)	0.1 (<0.1–1.3)
Cuba	39.6 (31.4–47.3)	24.4 (17.3–32.2)	0.2 (0.1–0.6)	0.4 (0.1–0.9)	8.8 (3.7–16.9)	8.8 (3.7–16.9)	6.0 (2.5–9.6)	6.0 (2.5–9.6)	7.8 (4.5–11.4)	8.1 (4.9–11.7)	9.4 (4.2–14.1)	2.1 (0.3–4.1)	1.5 (<0.1–4.0)	1.5 (<0.1–4.0)	0.2 (<0.1–1.6)	0.2 (<0.1–1.6)

	Smoking		Household air pollution		Ambient particulate matter		Handwashing		Secondhand smoke		Alcohol use		High temperature		Low temperature	
	M	F	M	F	M	F	M	F	M	F	M	F	M	F	M	F
Dominica	18.3 (12.8–24.4)	5.9 (3.5–9.0)	1.0 (0.3–2.3)	1.5 (0.4–3.4)	9.4 (3.2–19.0)	9.4 (3.3–18.9)	8.3 (3.5–13.2)	8.3 (3.5–13.2)	4.5 (2.3–7.0)	4.4 (2.3–6.8)	11.5 (6.0–16.9)	3.0 (0.9–5.6)	1.1 (<0.1–3.5)	1.1 (<0.1–3.5)	0.3 (<0.1–3.2)	0.3 (<0.1–3.2)
Dominican Republic	24.3 (18.6–30.6)	14.8 (9.8–20.9)	1.8 (0.6–4.0)	2.7 (1.0–5.6)	8.9 (3.3–17.9)	8.8 (3.4–17.7)	16.0 (7.1–24.3)	16.0 (7.1–24.3)	5.8 (3.3–8.5)	5.2 (2.9–7.6)	8.9 (3.8–14.0)	1.7 (0.1–3.6)	0.6 (<0.1–2.2)	0.6 (<0.1–2.2)	<0.1 (<0.1–1.7)	<0.1 (<0.1–1.7)
Grenada	21.1 (14.4–27.9)	6.3 (3.6–9.9)	0.4 (0.1–0.9)	0.6 (0.2–1.4)	10.8 (3.4–21.5)	10.8 (3.5–21.4)	7.6 (3.2–12.2)	7.6 (3.2–12.2)	4.2 (2.0–6.9)	4.3 (2.1–6.8)	13.2 (6.8–18.5)	3.9 (1.3–6.6)	1.4 (<0.1–4.2)	1.4 (<0.1–4.2)	0.3 (<0.1–1.8)	0.3 (<0.1–1.8)
Guyana	23.9 (16.7–30.8)	5.3 (2.9–8.0)	1.1 (0.4–2.3)	1.6 (0.6–3.3)	10.1 (3.2–20.5)	10.0 (3.2–20.3)	14.1 (6.1–21.9)	14.1 (6.1–21.9)	5.2 (2.7–7.8)	5.4 (2.7–8.2)	13.1 (6.8–19.1)	0.6 (0.0–1.9)	0.9 (<0.1–2.8)	0.9 (<0.1–2.8)	<0.1 (<0.1–1.3)	<0.1 (<0.1–1.3)
Haiti	15.5 (9.9–21.3)	4.1 (2.3–6.4)	33.4 (19.8–47.8)	41.2 (27.9–53.7)	6.3 (2.7–12.2)	5.1 (2.2–9.8)	19.5 (8.7–29.2)	19.5 (8.7–29.2)	3.0 (1.4–5.0)	2.8 (1.2–4.6)	11.0 (5.7–15.8)	2.4 (0.8–4.3)	0.8 (<0.1–3.0)	0.8 (<0.1–3.0)	<0.1 (<0.1–1.3)	<0.1 (<0.1–1.3)
Jamaica	29.3 (22.5–36.6)	9.4 (6.2–13.4)	1.3 (0.4–3.0)	2.0 (0.7–4.4)	7.7 (3.9–13.2)	7.7 (4.0–13.0)	11.2 (4.8–17.4)	11.2 (4.8–17.4)	6.0 (3.3–8.9)	7.0 (4.0–10.2)	7.3 (2.7–11.9)	1.5 (0.0–3.1)	1.2 (<0.1–4.3)	1.2 (<0.1–4.3)	0.1 (<0.1–1.1)	0.1 (<0.1–1.1)
Saint Kitts and Nevis	15.7 (11.1–21.1)	4.6 (2.7–7.4)	0.2 (<0.1–0.6)	0.4 (0.1–1.0)	3.5 (1.3–6.7)	3.5 (1.4–6.7)	7.6 (3.2–12.1)	7.6 (3.2–12.1)	5.7 (3.0–8.7)	5.6 (3.2–8.5)	4.6 (0.0–13.7)	1.0 (0.0–4.3)	1.0 (<0.1–3.9)	1.0 (<0.1–3.9)	0.4 (<0.1–2.3)	0.4 (<0.1–2.3)
Saint Lucia	25.0 (18.3–31.9)	6.7 (4.3–9.9)	0.5 (0.1–1.1)	0.7 (0.3–1.6)	10.7 (3.6–21.1)	10.7 (3.8–21.0)	5.7 (2.3–9.2)	5.7 (2.3–9.2)	4.7 (2.4–7.3)	4.4 (2.3–6.8)	13.5 (7.7–19.1)	3.8 (0.8–7.3)	1.7 (<0.1–4.8)	1.7 (<0.1–4.8)	0.3 (<0.1–1.9)	0.3 (<0.1–1.9)
Saint Vincent and the Grenadines	22.0 (15.1–29.0)	5.0 (2.8–7.7)	0.6 (0.2–1.4)	0.9 (0.3–2.0)	10.6 (3.3–21.3)	10.6 (3.4–21.1)	9.1 (3.8–14.3)	9.1 (3.8–14.3)	4.7 (2.4–7.3)	5.1 (2.7–7.9)	12.4 (6.4–17.6)	3.5 (1.2–5.8)	1.3 (<0.1–4.0)	1.3 (<0.1–4.0)	0.4 (<0.1–2.0)	0.4 (<0.1–2.0)
Suriname	35.3 (26.6–43.4)	11.7 (7.3–17.1)	1.5 (0.5–3.6)	2.3 (0.8–5.1)	10.7 (4.1–20.6)	10.6 (4.2–20.4)	9.3 (3.9–14.9)	9.3 (3.9–14.9)	7.0 (3.9–10.6)	9.6 (5.4–14.1)	9.9 (4.3–15.0)	2.3 (0.3–4.6)	1.0 (<0.1–3.0)	1.0 (<0.1–3.0)	0.3 (<0.1–1.6)	0.3 (<0.1–1.6)
Trinidad and Tobago	29.2 (21.4–37.1)	8.1 (4.9–12.2)	<0.1 (<0.1–<0.1)	<0.1 (<0.1–0.1)	11.0 (3.1–22.8)	11.0 (3.1–22.8)	6.8 (2.8–10.9)	6.8 (2.8–10.9)	5.5 (2.8–8.7)	7.3 (3.8–11.2)	10.5 (4.7–15.8)	3.3 (0.5–6.3)	0.4 (<0.1–2.6)	0.4 (<0.1–2.6)	0.2 (<0.1–1.2)	0.2 (<0.1–1.2)
Central Latin America	19.7 (13.5–26.1)	7.7 (4.6–11.0)	4.0 (1.8–6.9)	6.3 (3.4–10.2)	10.6 (6.8–15.7)	10.4 (6.8–15.3)	5.8 (2.4–9.3)	6.0 (2.5–9.6)	3.8 (1.5–6.6)	3.7 (1.5–6.8)	9.5 (4.8–13.5)	1.6 (0.2–3.1)	1.2 (0.1–3.2)	1.2 (0.1–3.1)	4.5 (2.3–6.7)	4.4 (2.3–6.6)
Colombia	19.7 (13.3–26.2)	10.6 (6.7–15.1)	1.7 (0.6–3.8)	2.6 (1.0–5.3)	11.2 (6.4–17.6)	11.1 (6.5–17.4)	6.1 (2.5–9.9)	6.1 (2.5–9.9)	5.0 (2.2–8.2)	4.3 (2.0–7.1)	9.0 (4.1–13.5)	0.8 (0.0–2.3)	1.0 (<0.1–3.4)	1.0 (<0.1–3.4)	3.4 (1.8–5.1)	3.4 (1.8–5.1)
Costa Rica	24.3 (18.4–30.6)	9.1 (5.7–12.9)	0.8 (0.2–1.9)	1.2 (0.4–2.8)	8.9 (5.2–13.8)	8.8 (5.2–13.7)	6.8 (2.8–10.9)	6.8 (2.8–10.9)	6.7 (3.8–10.1)	6.9 (3.8–10.3)	8.3 (3.3–12.8)	1.0 (0.0–2.6)	0.3 (<0.1–2.2)	0.3 (<0.1–2.2)	0.6 (<0.1–2.7)	0.6 (<0.1–2.7)
El Salvador	18.1 (12.8–23.5)	5.7 (3.5–8.7)	2.9 (1.2–5.7)	4.3 (2.0–7.6)	11.3 (5.9–18.3)	11.0 (6.0–17.6)	9.0 (3.8–14.4)	9.0 (3.8–14.4)	4.1 (2.1–6.6)	3.3 (1.7–5.3)	6.1 (2.3–9.5)	1.1 (0.0–2.5)	1.4 (<0.1–6.7)	1.4 (<0.1–6.7)	<0.1 (<0.1–0.6)	<0.1 (<0.1–0.6)
Guatemala	17.2 (11.0–23.6)	5.0 (2.7–7.9)	13.8 (6.3–24.3)	19.0 (10.1–30.7)	12.0 (7.5–17.9)	10.9 (6.7–16.0)	7.6 (3.2–12.1)	7.6 (3.2–12.1)	3.1 (1.1–5.5)	3.2 (1.2–5.8)	3.2 (0.6–6.1)	0.6 (0.0–1.5)	1.7 (0.5–3.5)	1.7 (0.5–3.5)	1.5 (<0.1–3.2)	1.5 (<0.1–3.2)
Honduras	23.5 (17.5–30.0)	6.9 (3.8–10.7)	17.3 (8.9–28.3)	23.3 (13.4–34.7)	9.6 (6.0–14.5)	8.5 (5.3–12.7)	5.4 (2.2–8.6)	5.4 (2.2–8.6)	6.0 (3.1–9.3)	6.6 (3.5–10.1)	6.7 (3.3–10.0)	0.7 (0.1–1.4)	0.7 (<0.1–2.6)	0.7 (<0.1–2.6)	<0.1 (<0.1–2.4)	<0.1 (<0.1–2.4)
Mexico	19.7 (12.9–26.8)	7.5 (4.2–11.0)	2.3 (0.9–4.4)	3.5 (1.7–6.3)	10.2 (6.3–15.5)	10.1 (6.3–15.0)	5.0 (2.0–8.0)	5.0 (2.0–8.1)	2.8 (0.8–5.7)	3.2 (0.9–6.6)	11.4 (6.1–15.9)	2.4 (0.4–4.4)	1.2 (0.4–2.1)	1.2 (0.3–2.0)	7.6 (4.7–10.5)	7.7 (4.8–10.7)

	Smoking		Household air pollution		Ambient particulate matter		Handwashing		Secondhand smoke		Alcohol use		High temperature		Low temperature	
	M	F	M	F	M	F	M	F	M	F	M	F	M	F	M	F
Nicaragua	19.9 (13.8–26.5)	5.3 (3.1–8.3)	13.4 (5.9–23.7)	18.5 (9.8–29.4)	9.2 (5.4–14.8)	8.4 (4.9–13.5)	8.1 (3.4–12.8)	8.1 (3.4–12.8)	5.6 (2.6–9.0)	5.5 (2.6–8.8)	7.0 (3.3–10.2)	0.7 (0.1–1.4)	0.8 (<0.1–4.9)	0.8 (<0.1–4.9)	<0.1 (<0.1–1.1)	<0.1 (<0.1–1.1)
Panama	16.5 (12.0–21.5)	4.8 (2.9–7.4)	1.3 (0.4–2.7)	1.9 (0.6–4.1)	6.5 (3.2–10.9)	6.5 (3.3–10.9)	6.1 (2.5–9.8)	6.1 (2.5–9.8)	4.5 (2.6–6.6)	4.2 (2.3–6.3)	11.5 (6.3–16.6)	2.4 (0.4–4.4)	0.6 (<0.1–2.3)	0.6 (<0.1–2.3)	<0.1 (<0.1–1.0)	<0.1 (<0.1–1.0)
Venezuela (Bolivarian Republic of)	21.6 (14.6–28.6)	10.6 (6.1–15.7)	0.1 (<0.1–0.2)	0.1 (<0.1–0.2)	11.1 (5.9–18.6)	11.1 (5.9–18.6)	5.7 (2.4–9.2)	5.7 (2.4–9.2)	5.5 (2.5–9.2)	5.5 (2.5–8.9)	10.4 (5.5–15.3)	1.9 (0.3–4.0)	1.2 (<0.1–9.4)	1.2 (<0.1–9.4)	0.1 (<0.1–1.7)	0.1 (<0.1–1.7)
Tropical Latin America	26.6 (20.4–33.2)	19.3 (14.0–25.0)	1.4 (0.5–3.1)	2.1 (0.8–4.1)	5.8 (3.3–9.4)	5.8 (3.4–9.3)	6.0 (2.4–9.6)	6.0 (2.4–9.7)	7.8 (4.6–11.4)	8.1 (4.7–11.6)	10.3 (5.3–14.8)	2.0 (0.4–3.7)	1.8 (<0.1–5.9)	1.8 (<0.1–6.2)	1.1 (<0.1–3.3)	1.1 (<0.1–3.2)
Brazil	26.6 (20.5–33.1)	19.4 (14.1–25.1)	1.3 (0.4–2.9)	2.0 (0.7–4.0)	5.8 (3.3–9.3)	5.8 (3.3–9.3)	6.0 (2.4–9.6)	6.0 (2.4–9.6)	7.8 (4.6–11.4)	8.1 (4.7–11.6)	10.2 (5.2–14.7)	2.0 (0.4–3.7)	1.7 (<0.1–5.9)	1.7 (<0.1–6.2)	1.1 (<0.1–3.3)	1.1 (<0.1–3.2)
Paraguay	30.3 (22.3–38.9)	11.3 (6.6–16.4)	6.1 (2.2–12.2)	9.0 (3.8–16.3)	6.3 (3.5–10.6)	6.1 (3.6–10.2)	7.9 (3.3–12.7)	7.9 (3.3–12.7)	7.1 (3.9–10.6)	8.7 (4.9–12.8)	14.9 (7.9–20.9)	2.1 (0.0–4.8)	5.2 (2.9–7.6)	5.2 (2.9–7.6)	0.9 (<0.1–5.0)	0.9 (<0.1–5.0)
**North Africa and Middle East**	**36.6 (29.7–43.6)**	**6.0 (4.2–8.0)**	**2.4 (1.2–3.8)**	**4.0 (2.5–5.8)**	**21.3 (14.6–29.1)**	**20.8 (14.4–28.1)**	**6.1 (2.5–9.8)**	**6.4 (2.7–10.1)**	**9.7 (6.1–13.6)**	**14.6 (9.3–19.9)**	**1.1 (0.3–1.8)**	**0.2 (0.0–0.4)**	**5.7 (3.5–8.0)**	**5.7 (3.6–8.0)**	**7.0 (3.2–11.2)**	**7.0 (3.3–10.9)**
Afghanistan	20.8 (15.4–27.1)	3.9 (2.3–6.0)	32.5 (19.2–44.6)	38.0 (26.3–49.2)	12.7 (6.2–20.9)	9.8 (4.3–17.4)	15.7 (6.8–23.8)	15.7 (6.8–23.8)	9.2 (5.4–13.4)	11.6 (6.8–16.6)	0.1 (0.0–0.3)	<0.1 (0.0–<0.1)	3.1 (1.9–4.2)	3.1 (1.9–4.2)	13.7 (9.0–19.6)	13.7 (9.0–19.6)
Algeria	35.9 (28.4–43.2)	3.1 (1.9–4.9)	<0.1 (<0.1–0.1)	0.1 (<0.1–0.1)	16.6 (9.1–25.5)	16.6 (9.1–25.5)	4.9 (2.0–7.9)	4.9 (2.0–7.9)	11.9 (7.4–17.0)	18.9 (12.2–25.6)	1.5 (0.4–2.8)	0.1 (0.0–0.4)	9.2 (6.2–12.2)	9.2 (6.2–12.2)	4.3 (<0.1–10.1)	4.3 (<0.1–10.1)
Bahrain	28.0 (21.5–35.3)	7.6 (4.5–11.2)	0.1 (<0.1–0.1)	0.1 (<0.1–0.2)	26.5 (17.9–36.4)	26.5 (17.9–36.4)	4.6 (1.9–7.4)	4.6 (1.9–7.4)	10.3 (6.4–14.7)	12.0 (7.7–17.0)	2.1 (0.5–3.8)	0.2 (0.0–0.6)	11.6 (5.8–16.6)	11.6 (5.8–16.6)	2.0 (<0.1–10.1)	2.0 (<0.1–10.1)
Egypt	41.6 (33.6–49.2)	1.6 (0.9–2.6)	<0.1 (<0.1–<0.1)	<0.1 (<0.1–0.1)	28.9 (19.3–40.0)	28.9 (19.3–40.0)	3.8 (1.5–6.3)	3.8 (1.5–6.3)	8.5 (5.2–12.0)	15.5 (9.8–21.3)	0.5 (0.0–1.3)	<0.1 (0.0–0.2)	7.7 (4.8–10.9)	7.7 (4.8–10.9)	2.7 (<0.1–9.1)	2.7 (<0.1–9.1)
Iran (Islamic Republic of)	32.8 (26.0–39.8)	7.5 (4.9–10.7)	<0.1 (<0.1–0.1)	0.1 (<0.1–0.1)	18.8 (12.3–26.5)	18.8 (12.3–26.5)	4.3 (1.7–7.0)	4.3 (1.7–7.0)	9.4 (5.7–13.5)	13.1 (8.3–18.6)	1.5 (0.8–2.3)	0.2 (0.1–0.4)	3.2 (2.1–4.3)	3.2 (2.2–4.3)	13.3 (10.1–16.4)	13.2 (10.1–16.4)
Iraq	40.6 (33.3–47.9)	9.0 (6.1–12.6)	<0.1 (<0.1–0.1)	0.1 (<0.1–0.2)	22.7 (14.6–32.4)	22.7 (14.6–32.3)	2.7 (1.1–4.5)	2.7 (1.1–4.5)	11.8 (7.4–16.6)	15.9 (10.2–21.8)	0.8 (0.0–1.9)	0.1 (0.0–0.3)	10.8 (7.5–13.8)	10.8 (7.5–13.8)	4.7 (<0.1–10.6)	4.7 (<0.1–10.6)
Jordan	44.7 (37.0–52.0)	12.5 (8.5–17.1)	<0.1 (<0.1–<0.1)	<0.1 (<0.1–<0.1)	15.7 (9.8–23.4)	15.7 (9.8–23.4)	4.2 (1.7–6.9)	4.2 (1.7–6.9)	11.2 (6.8–15.7)	16.0 (10.0–22.1)	1.1 (0.0–2.5)	0.1 (0.0–0.3)	3.2 (1.7–4.9)	3.2 (1.7–4.9)	6.0 (<0.1–12.1)	6.0 (<0.1–12.1)
Kuwait	33.7 (27.3–40.9)	5.7 (3.4–8.8)	<0.1 (<0.1–<0.1)	<0.1 (<0.1–<0.1)	27.1 (18.5–36.9)	27.1 (18.5–36.9)	3.7 (1.5–6.0)	3.7 (1.5–6.0)	11.2 (7.1–15.8)	18.0 (12.0–24.5)	0.1 (0.0–0.3)	<0.1 (0.0–<0.1)	13.1 (7.6–18.0)	13.1 (7.6–18.0)	3.1 (<0.1–15.6)	3.1 (<0.1–15.6)
Lebanon	46.7 (38.7–54.3)	33.6 (26.1–41.2)	<0.1 (<0.1–0.1)	0.1 (<0.1–0.1)	14.9 (9.0–22.6)	14.9 (9.0–22.6)	5.0 (2.1–8.0)	5.0 (2.1–8.0)	14.1 (9.1–19.4)	15.0 (9.7–20.8)	3.0 (1.3–4.8)	0.2 (0.0–0.6)	0.1 (<0.1–0.2)	0.1 (<0.1–0.2)	14.0 (10.4–17.5)	14.0 (10.4–17.5)
Libya	39.9 (32.8–46.8)	1.3 (0.7–2.2)	<0.1 (<0.1–0.1)	0.1 (<0.1–0.1)	19.0 (11.0–29.3)	19.0 (11.0–29.3)	4.5 (1.9–7.4)	4.5 (1.9–7.4)	13.6 (8.6–18.7)	19.7 (13.0–26.3)	0.8 (0.1–1.3)	0.1 (<0.1–0.2)	8.2 (4.7–12.0)	8.2 (4.7–12.0)	2.8 (<0.1–9.8)	2.8 (<0.1–9.8)
Morocco	27.7 (20.9–34.7)	2.0 (1.2–3.2)	0.9 (0.3–1.8)	1.3 (0.5–2.7)	17.4 (10.8–25.2)	17.3 (10.7–24.9)	7.6 (3.2–12.2)	7.6 (3.2–12.2)	8.5 (5.0–12.3)	11.2 (6.8–15.9)	0.8 (0.0–1.8)	<0.1 (0.0–<0.1)	2.5 (1.6–3.4)	2.5 (1.6–3.4)	8.2 (4.8–11.8)	8.2 (4.8–11.8)

	Smoking		Household air pollution		Ambient particulate matter		Handwashing		Secondhand smoke		Alcohol use		High temperature		Low temperature	
	M	F	M	F	M	F	M	F	M	F	M	F	M	F	M	F
Oman	21.5 (16.2–27.4)	2.7 (1.6–4.3)	0.1 (<0.1–0.2)	0.1 (<0.1–0.3)	21.4 (12.7–31.3)	21.4 (12.7–31.3)	5.0 (2.0–8.2)	5.0 (2.0–8.2)	8.5 (5.0–12.3)	9.8 (6.0–14.0)	0.4 (0.0–1.1)	<0.1 (0.0–0.2)	6.6 (<0.1–22.3)	6.6 (<0.1–22.3)	1.3 (<0.1–13.3)	1.3 (<0.1–13.3)
Palestine	43.3 (35.0–51.3)	4.9 (2.9–7.6)	0.2 (0.1–0.4)	0.3 (0.1–0.6)	15.9 (9.5–24.7)	15.9 (9.5–24.7)	7.1 (3.0–11.4)	7.1 (3.0–11.4)	11.5 (7.0–16.0)	18.0 (11.7–24.4)	3.1 (1.6–4.6)	0.6 (0.2–1.1)	2.2 (0.9–4.0)	2.2 (0.9–4.0)	3.9 (<0.1–10.5)	3.9 (<0.1–10.5)
Qatar	26.6 (20.2–33.3)	2.8 (1.6–4.7)	<0.1 (<0.1–<0.1)	<0.1 (<0.1–<0.1)	31.1 (22.0–41.1)	31.1 (22.0–41.1)	3.8 (1.5–6.3)	3.8 (1.5–6.3)	11.0 (6.9–15.6)	14.5 (9.2–20.0)	0.9 (0.0–2.0)	0.1 (0.0–0.3)	10.6 (3.7–19.5)	10.6 (3.7–19.5)	1.8 (<0.1–15.2)	1.8 (<0.1–15.2)
Saudi Arabia	28.5 (22.1–35.7)	3.4 (2.0–5.3)	<0.1 (<0.1–0.1)	0.1 (<0.1–0.2)	27.0 (18.4–37.3)	27.0 (18.4–37.3)	4.2 (1.7–6.7)	4.2 (1.7–6.7)	11.2 (7.1–15.8)	16.8 (11.3–22.7)	0.4 (0.0–1.3)	<0.1 (0.0–0.2)	9.7 (5.9–14.1)	9.7 (5.9–14.1)	2.4 (<0.1–8.8)	2.4 (<0.1–8.8)
Sudan	32.6 (24.9–40.3)	4.7 (2.9–7.2)	11.6 (5.6–20.4)	16.2 (9.1–25.7)	20.9 (12.0–31.6)	19.2 (11.2–29.1)	17.7 (7.9–26.7)	17.7 (7.9–26.7)	11.6 (7.7–16.2)	13.7 (9.0–18.7)	<0.1 (0.0–0.1)	<0.1 (0.0–<0.1)	8.3 (3.6–15.7)	8.3 (3.6–15.7)	1.5 (<0.1–7.2)	1.5 (<0.1–7.2)
Syrian Arab Republic	40.5 (32.3–48.0)	8.3 (5.1–12.5)	<0.1 (<0.1–<0.1)	<0.1 (<0.1–0.1)	15.8 (9.6–23.6)	15.8 (9.6–23.6)	4.9 (2.0–7.9)	4.9 (2.0–7.9)	10.2 (6.4–14.7)	14.1 (9.0–20.0)	1.4 (0.5–2.3)	0.1 (<0.1–0.2)	4.4 (2.5–6.3)	4.4 (2.5–6.3)	7.7 (2.5–12.6)	7.7 (2.5–12.6)
Tunisia	41.1 (33.2–48.7)	4.7 (2.8–7.2)	<0.1 (<0.1–0.1)	0.1 (<0.1–0.1)	15.5 (8.9–24.1)	15.5 (8.9–24.1)	5.7 (2.4–9.2)	5.7 (2.4–9.2)	11.0 (6.7–15.6)	16.0 (10.1–22.0)	2.6 (1.0–4.4)	0.4 (0.0–0.9)	4.5 (2.3–6.7)	4.5 (2.3–6.7)	5.1 (<0.1–11.3)	5.1 (<0.1–11.3)
Turkey	42.0 (34.1–49.2)	16.6 (12.1–21.7)	0.1 (<0.1–0.2)	0.1 (<0.1–0.2)	13.5 (8.4–20.4)	13.5 (8.4–20.3)	5.8 (2.4–9.3)	5.8 (2.4–9.3)	8.9 (5.4–12.9)	13.2 (8.2–18.6)	2.6 (1.0–4.1)	0.8 (0.1–1.7)	0.4 (0.1–0.7)	0.4 (0.1–0.7)	18.3 (13.7–23.4)	18.3 (13.7–23.4)
United Arab Emirates	24.6 (18.1–31.2)	7.0 (4.2–10.8)	<0.1 (<0.1–<0.1)	<0.1 (<0.1–<0.1)	21.1 (13.2–30.8)	21.1 (13.2–30.8)	3.8 (1.5–6.2)	3.8 (1.5–6.2)	13.2 (8.3–19.0)	16.5 (10.5–22.5)	1.6 (0.0–3.8)	0.2 (0.0–0.8)	8.4 (<0.1–25.0)	8.4 (<0.1–25.0)	1.9 (<0.1–18.2)	1.9 (<0.1–18.2)
Yemen	39.5 (32.3–46.8)	13.8 (9.6–18.5)	12.1 (5.8–21.8)	16.3 (8.8–25.7)	17.3 (9.2–27.6)	15.5 (7.9–24.7)	13.5 (5.8–20.7)	13.5 (5.8–20.7)	11.3 (7.0–15.8)	14.5 (9.1–19.8)	1.0 (0.3–1.8)	0.1 (<0.1–0.3)	5.5 (1.6–12.3)	5.5 (1.6–12.3)	0.9 (<0.1–5.1)	0.9 (<0.1–5.1)
**South Asia**	**27.7 (20.6–34.4)**	**5.1 (3.3–7.4)**	**15.7 (8.9–24.8)**	**20.6 (13.3–29.5)**	**24.9 (18.3–31.3)**	**23.0 (17.0–29.2)**	**12.2 (5.2–18.8)**	**12.2 (5.3–18.9)**	**5.9 (3.1–8.9)**	**9.4 (5.1–14.0)**	**5.4 (2.4–8.2)**	**0.4 (0.1–0.9)**	**7.2 (4.0–11.5)**	**7.1 (4.0–11.2)**	**2.4 (<0.1–7.1)**	**2.2 (<0.1–6.7)**
Bangladesh	33.7 (24.5–42.7)	2.5 (1.3–4.2)	21.5 (11.9–33.7)	28.4 (18.0–40.0)	20.0 (13.8–26.5)	17.3 (11.5–23.3)	18.1 (8.1–27.1)	18.1 (8.1–27.1)	5.1 (2.4–8.3)	9.5 (4.6–14.7)	0.7 (0.0–1.5)	0.1 (0.0–0.3)	5.9 (2.7–9.8)	5.9 (2.7–9.8)	1.3 (<0.1–4.8)	1.3 (<0.1–4.8)
Bhutan	16.3 (11.3–21.7)	6.1 (3.6–9.4)	16.3 (8.1–27.9)	21.6 (12.9–32.9)	15.2 (10.1–21.3)	13.3 (8.4–19.1)	6.1 (2.5–9.8)	6.1 (2.5–9.8)	5.3 (2.8–8.1)	4.9 (2.6–7.3)	1.9 (0.0–4.7)	0.1 (0.0–0.4)	<0.1 (<0.1–0.1)	<0.1 (<0.1–0.1)	18.6 (11.8–26.9)	18.6 (11.8–26.9)
India	27.0 (20.0–33.6)	4.9 (3.2–7.4)	15.0 (8.4–23.7)	19.9 (12.8–28.6)	25.7 (19.1–32.2)	23.6 (17.4–29.8)	11.7 (5.0–18.0)	11.9 (5.2–18.4)	5.9 (3.1–9.0)	9.4 (5.2–14.0)	6.2 (2.8–9.5)	0.5 (0.1–1.0)	7.2 (3.9–11.6)	7.2 (4.0–11.7)	2.1 (<0.1–6.7)	2.0 (<0.1–6.5)
Nepal	28.5 (19.2–37.7)	18.4 (11.4–25.8)	22.4 (12.6–35.1)	28.9 (18.4–40.2)	22.8 (15.0–31.2)	19.4 (12.0–27.6)	11.3 (4.9–17.8)	11.3 (4.9–17.8)	4.9 (2.2–8.4)	5.1 (2.2–8.5)	4.7 (0.2–9.9)	0.7 (0.0–1.9)	1.4 (0.7–2.2)	1.4 (0.7–2.2)	13.0 (7.6–19.0)	13.0 (7.6–19.0)
Pakistan	30.2 (23.2–37.2)	6.8 (4.0–10.2)	17.2 (9.3–26.9)	22.7 (13.9–32.4)	21.0 (14.3–28.5)	18.5 (12.6–25.4)	12.3 (5.2–19.1)	12.3 (5.2–19.2)	6.4 (3.4–10.0)	9.5 (5.1–14.1)	1.5 (0.5–2.6)	0.1 (<0.1–0.2)	9.2 (5.5–16.4)	9.2 (5.4–15.8)	4.8 (<0.1–12.6)	4.9 (<0.1–12.8)
**Southeast Asia, East Asia, and Oceania**	**41.1 (33.2–48.5)**	**6.1 (3.8–8.5)**	**7.9 (3.7–13.5)**	**11.4 (6.3–17.3)**	**13.5 (9.1–18.6)**	**12.6 (8.7–17.3)**	**5.8 (2.4–9.2)**	**5.8 (2.4–9.4)**	**7.3 (4.4–10.5)**	**14.4 (8.7–20.0)**	**9.9 (5.1–14.1)**	**1.2 (0.3–2.1)**	**1.4 (<0.1–5.8)**	**1.4 (<0.1–6.4)**	**4.6 (2.5–6.8)**	**4.4 (2.2–6.7)**
East Asia	47.3 (40.0–54.4)	5.5 (3.8–7.6)	5.2 (2.2–9.5)	8.1 (4.1–14.1)	19.6 (13.5–26.5)	19.0 (13.3–25.3)	3.8 (1.5–6.1)	3.8 (1.5–6.2)	9.1 (5.8–13.1)	18.6 (12.3–25.2)	12.3 (6.4–17.4)	1.2 (0.3–2.2)	0.7 (0.1–1.2)	0.6 (0.1–1.2)	12.2 (7.5–16.4)	12.8 (7.6–17.3)

	Smoking		Household air pollution		Ambient particulate matter		Handwashing		Secondhand smoke		Alcohol use		High temperature		Low temperature	
	**M**	**F**	**M**	**F**	**M**	**F**	**M**	**F**	**M**	**F**	**M**	**F**	**M**	**F**	**M**	**F**
China	47.8 (40.4–55.0)	5.5 (3.7–7.6)	4.9 (2.0–9.1)	7.4 (3.5–13.2)	20.4 (14.0–27.3)	19.7 (13.8–26.3)	4.0 (1.6–6.5)	4.0 (1.6–6.5)	9.3 (5.8–13.3)	18.9 (12.6–25.6)	12.7 (6.5–17.8)	1.3 (0.3–2.4)	0.7 (0.1–1.2)	0.6 (0.1–1.2)	12.6 (7.6–16.9)	12.9 (7.6–17.6)
Democratic People's Republic of Korea	45.9 (38.1–53.5)	6.6 (3.8–10.7)	19.5 (10.2–31.3)	26.2 (16.0–38.1)	16.4 (10.4–22.7)	14.4 (9.2–20.5)	2.3 (0.9–3.7)	2.3 (0.9–3.7)	7.7 (4.6–11.5)	17.4 (11.0–23.8)	9.2 (3.9–14.4)	0.6 (0.0–1.4)	0.1 (<0.1–0.7)	0.1 (<0.1–0.7)	19.3 (11.7–29.7)	19.3 (11.7–29.7)
Taiwan (Province of China)	41.8 (34.0–49.4)	4.8 (2.8–7.2)	0.4 (0.1–1.2)	0.7 (0.1–1.8)	12.1 (7.5–17.9)	12.1 (7.5–17.9)	1.8 (0.7–3.0)	1.8 (0.7–3.0)	8.4 (5.0–12.1)	14.0 (8.7–19.6)	10.3 (4.9–15.3)	0.7 (<0.1–1.6)	0.9 (0.2–2.3)	0.9 (0.2–2.3)	3.5 (<0.1–6.8)	3.5 (<0.1–6.8)
Oceania	30.4 (20.9–39.1)	15.7 (9.7–21.9)	32.0 (19.1–45.0)	38.6 (27.1–49.3)	4.6 (1.4–11.0)	3.8 (1.2–9.0)	9.2 (3.9–14.6)	9.3 (4.0–15.0)	7.4 (3.6–12.0)	10.6 (5.2–16.6)	1.9 (0.0–4.9)	0.1 (0.0–0.4)	0.5 (<0.1–2.2)	0.5 (<0.1–2.2)	1.0 (<0.1–2.4)	1.0 (<0.1–2.3)
Fiji	30.9 (20.7–40.7)	10.1 (5.4–15.7)	4.2 (1.2–9.4)	6.4 (2.2–13.1)	5.4 (1.4–13.3)	5.4 (1.5–13.1)	2.9 (1.2–4.8)	2.9 (1.2–4.8)	5.6 (2.6–9.7)	8.3 (3.8–13.5)	5.6 (2.2–9.1)	0.3 (0.0–0.7)	0.5 (<0.1–2.4)	0.5 (<0.1–2.4)	<0.1 (<0.1–1.7)	<0.1 (<0.1–1.7)
Kiribati	47.8 (39.3–55.9)	31.5 (21.7–40.7)	24.1 (12.5–37.1)	31.0 (19.8–43.3)	3.8 (1.3–8.6)	3.2 (1.1–7.3)	6.5 (2.7–10.4)	6.5 (2.7–10.4)	9.8 (5.1–14.7)	13.1 (7.2–19.4)	1.6 (0.0–5.1)	<0.1 (0.0–0.1)	0.9 (<0.1–5.0)	0.9 (<0.1–5.0)	<0.1 (<0.1–0.1)	<0.1 (<0.1–0.1)
Marshall Islands	28.4 (20.3–36.5)	8.5 (4.9–12.6)	7.5 (3.0–14.4)	10.9 (5.1–18.9)	4.4 (1.6–9.2)	4.2 (1.6–8.7)	4.5 (1.8–7.3)	4.5 (1.8–7.3)	7.3 (3.7–11.3)	11.2 (6.1–16.8)	3.4 (0.0–9.1)	0.1 (0.0–0.7)	0.2 (<0.1–11.3)	0.2 (<0.1–11.3)	<0.1 (<0.1–0.1)	<0.1 (<0.1–0.1)
Micronesia (Federated States of)	37.4 (26.6–47.3)	23.7 (14.6–33.7)	6.6 (2.4–13.4)	9.8 (4.1–18.1)	5.1 (1.5–12.2)	4.9 (1.5–11.4)	3.9 (1.6–6.4)	3.9 (1.6–6.4)	6.3 (2.7–10.8)	9.0 (4.1–14.8)	3.3 (0.7–6.1)	<0.1 (0.0–0.2)	0.2 (<0.1–11.3)	0.2 (<0.1–11.3)	<0.1 (<0.1–0.2)	<0.1 (<0.1–0.2)
Nauru	30.8 (21.2–40.0)	29.9 (20.1–39.9)	0.5 (0.1–1.5)	0.9 (0.2–2.2)	1.9 (0.4–5.1)	2.0 (0.6–5.2)	3.1 (1.2–5.1)	3.1 (1.2–5.1)	6.8 (3.0–11.5)	9.6 (4.3–15.6)	8.9 (3.0–14.4)	0.3 (0.0–1.2)	2.4 (<0.1–31.1)	2.4 (<0.1–31.1)	<0.1 (<0.1–0.3)	<0.1 (<0.1–0.3)
Palau	28.6 (20.3–37.4)	10.2 (5.7–15.7)	<0.1 (<0.1–<0.1)	<0.1 (<0.1–<0.1)	2.0 (<0.1–5.9)	2.0 (<0.1–5.9)	2.4 (0.9–3.9)	2.4 (0.9–3.9)	9.5 (5.0–14.5)	13.2 (7.4–19.5)	3.6 (0.0–9.7)	0.1 (0.0–0.6)	0.9 (<0.1–28.7)	0.9 (<0.1–28.7)	<0.1 (<0.1–0.4)	<0.1 (<0.1–0.4)
Papua New Guinea	27.7 (18.1–37.1)	16.7 (9.7–23.6)	37.9 (22.7–52.8)	44.8 (31.5–56.7)	4.7 (1.3–12.1)	3.7 (1.0–9.9)	12.2 (5.2–19.0)	12.2 (5.2–19.0)	7.5 (3.5–12.3)	10.5 (4.9–16.5)	1.4 (0.0–4.8)	0.1 (0.0–0.4)	0.4 (<0.1–1.9)	0.4 (<0.1–1.9)	1.6 (<0.1–3.7)	1.6 (<0.1–3.7)
Samoa	42.2 (34.1–49.8)	17.4 (12.0–23.8)	14.8 (6.6–26.4)	20.6 (11.2–32.3)	5.2 (1.4–12.2)	4.7 (1.3–10.9)	2.2 (0.9–3.6)	2.2 (0.9–3.6)	11.4 (6.5–16.4)	15.9 (9.8–22.3)	4.5 (0.0–9.5)	0.1 (0.0–0.8)	0.4 (<0.1–2.2)	0.4 (<0.1–2.2)	0.1 (<0.1–1.4)	0.1 (<0.1–1.4)
Solomon Islands	37.0 (28.0–46.2)	15.1 (9.6–21.5)	38.1 (23.2–53.1)	45.4 (31.8–57.7)	3.9 (1.2–9.2)	3.1 (0.9–7.2)	4.9 (2.0–7.9)	4.9 (2.0–7.9)	7.7 (3.8–12.0)	11.4 (6.0–17.4)	1.2 (0.0–4.2)	0.1 (0.0–0.3)	0.5 (<0.1–3.3)	0.5 (<0.1–3.3)	<0.1 (<0.1–0.5)	<0.1 (<0.1–0.5)
Tonga	37.4 (29.0–45.7)	10.2 (5.9–14.9)	6.7 (2.3–13.4)	9.9 (4.0–18.4)	5.3 (1.5–12.4)	5.1 (1.6–11.6)	3.3 (1.3–5.4)	3.3 (1.3–5.4)	8.5 (4.6–12.8)	12.7 (7.2–18.8)	1.8 (0.0–5.8)	0.1 (0.0–0.4)	1.2 (<0.1–3.0)	1.2 (<0.1–3.0)	0.2 (<0.1–2.6)	0.2 (<0.1–2.6)
Tuvalu	34.0 (23.7–43.2)	17.2 (10.5–25.4)	1.6 (0.4–4.1)	2.6 (0.8–5.6)	2.4 (0.8–5.6)	2.5 (1.0–5.6)	3.7 (1.5–6.1)	3.7 (1.5–6.1)	8.0 (3.9–12.8)	11.1 (5.7–17.3)	2.5 (0.0–6.3)	0.1 (0.0–0.4)	1.4 (<0.1–12.8)	1.4 (<0.1–12.8)	0.1 (<0.1–0.7)	0.1 (<0.1–0.7)
Vanuatu	24.9 (16.1–33.5)	3.0 (1.5–5.0)	28.4 (15.5–42.8)	36.3 (23.3–49.6)	4.9 (1.5–10.9)	4.1 (1.2–9.2)	10.0 (4.3–15.7)	10.0 (4.3–15.7)	5.0 (2.0–8.7)	8.0 (3.6–13.7)	2.5 (0.4–5.0)	<0.1 (0.0–0.2)	0.4 (<0.1–2.1)	0.4 (<0.1–2.1)	<0.1 (<0.1–1.8)	<0.1 (<0.1–1.8)
Southeast Asia	37.6 (29.2–45.7)	6.2 (3.6–8.9)	9.1 (4.3–15.7)	12.5 (7.0–19.2)	10.0 (6.5–14.4)	9.5 (6.3–13.5)	7.0 (2.9–11.1)	6.8 (2.8–11.0)	6.2 (3.4–9.3)	12.3 (7.1–17.7)	8.6 (4.2–12.4)	1.2 (0.2–2.2)	1.9 (<0.1–8.9)	1.9 (<0.1–9.3)	0.1 (<0.1–1.3)	<0.1 (<0.1–1.2)
Cambodia	45.5 (36.9–53.6)	6.9 (4.1–10.3)	26.8 (14.9–40.5)	34.3 (22.1–47.2)	8.1 (4.5–13.0)	6.8 (3.9–10.9)	8.6 (3.5–13.5)	8.6 (3.5–13.5)	5.5 (3.3–8.0)	15.7 (9.8–21.6)	12.7 (5.8–19.1)	1.6 (0.0–3.4)	2.7 (<0.1–21.8)	2.7 (<0.1–21.8)	<0.1 (<0.1–0.3)	<0.1 (<0.1–0.3)

	Smoking		Household air pollution		Ambient particulate matter		Handwashing		Secondhand smoke		Alcohol use		High temperature		Low temperature	
	M	F	M	F	M	F	M	F	M	F	M	F	M	F	M	F
Indonesia	40.1 (29.8–49.3)	3.9 (2.2–6.2)	5.9 (2.3–11.5)	9.2 (4.2–16.3)	9.5 (6.0–14.3)	9.0 (5.8–13.3)	7.3 (3.0–11.5)	7.4 (3.0–11.7)	5.5 (2.9–8.2)	14.0 (7.9–20.3)	0.3 (0.0–1.5)	<0.1 (0.0–0.1)	0.3 (<0.1–3.4)	0.3 (<0.1–3.6)	<0.1 (<0.1–0.9)	<0.1 (<0.1–0.9)
Lao People's Democratic Republic	39.2 (29.1–48.2)	6.2 (3.3–10.0)	28.9 (16.1–43.2)	36.9 (23.8–50.3)	7.5 (4.3–11.6)	6.3 (3.6–9.6)	10.2 (4.3–15.9)	10.2 (4.3–15.9)	5.6 (2.9–8.4)	13.3 (7.5–19.3)	8.0 (1.7–14.3)	2.0 (0.0–4.7)	1.8 (0.6–3.9)	1.8 (0.6–3.9)	<0.1 (<0.1–2.9)	<0.1 (<0.1–2.9)
Malaysia	35.1 (27.0–42.6)	3.4 (1.8–5.4)	0.1 (<0.1–0.2)	0.1 (<0.1–0.3)	8.3 (4.7–13.2)	8.3 (4.7–13.2)	2.0 (0.8–3.2)	2.0 (0.8–3.2)	7.2 (3.8–10.7)	13.4 (7.7–19.3)	3.2 (0.7–6.0)	0.3 (0.0–0.8)	0.8 (<0.1–3.9)	0.8 (<0.1–3.9)	<0.1 (<0.1–0.8)	<0.1 (<0.1–0.8)
Maldives	35.5 (27.8–43.1)	6.6 (3.7–10.0)	2.1 (0.7–5.0)	3.2 (1.2–7.0)	5.2 (2.9–8.8)	5.2 (3.0–8.4)	8.1 (3.5–13.0)	8.1 (3.5–13.0)	8.7 (5.0–13.2)	14.8 (8.8–21.3)	1.2 (0.0–4.9)	0.1 (0.0–0.4)	0.4 (<0.1–11.4)	0.4 (<0.1–11.4)	<0.1 (<0.1–0.1)	<0.1 (<0.1–0.1)
Mauritius	29.7 (21.2–38.4)	4.0 (2.2–6.5)	0.1 (<0.1–0.4)	0.2 (0.1–0.5)	7.3 (3.0–13.8)	7.3 (3.1–13.7)	1.1 (0.4–1.8)	1.1 (0.4–1.8)	6.4 (3.4–9.8)	12.4 (7.1–18.2)	8.1 (2.5–14.2)	0.4 (0.0–1.8)	0.7 (<0.1–3.5)	0.7 (<0.1–3.5)	<0.1 (<0.1–5.4)	<0.1 (<0.1–5.4)
Myanmar	30.1 (20.0–39.9)	10.4 (5.3–15.8)	20.0 (10.1–31.9)	26.8 (16.1–39.0)	11.8 (7.9–16.1)	10.4 (7.0–14.1)	11.1 (4.7–17.5)	11.1 (4.7–17.5)	4.7 (2.0–8.1)	7.6 (3.5–12.6)	6.8 (2.4–11.2)	0.3 (0.0–1.0)	2.9 (1.3–5.3)	2.9 (1.3–5.3)	1.9 (<0.1–4.3)	1.9 (<0.1–4.3)
Philippines	39.5 (31.3–47.4)	10.3 (6.6–14.5)	12.3 (5.6–21.3)	16.2 (8.6–25.3)	8.8 (5.7–12.5)	8.5 (5.6–12.0)	6.4 (2.6–10.1)	6.3 (2.6–10.0)	8.2 (4.4–11.9)	12.3 (7.1–17.7)	11.3 (5.2–16.7)	2.9 (0.8–5.1)	2.4 (<0.1–23.4)	2.4 (<0.1–24.0)	0.2 (<0.1–0.7)	0.2 (<0.1–0.7)
Seychelles	34.7 (27.4–42.4)	6.2 (3.6–9.5)	0.1 (<0.1–0.2)	0.1 (<0.1–0.2)	7.7 (3.3–14.3)	7.7 (3.3–14.3)	3.9 (1.6–6.3)	3.9 (1.6–6.3)	6.5 (3.5–9.7)	11.4 (6.4–16.4)	12.8 (5.7–19.4)	1.3 (0.0–2.9)	1.0 (<0.1–3.8)	1.0 (<0.1–3.8)	0.2 (<0.1–1.3)	0.2 (<0.1–1.3)
Sri Lanka	24.0 (14.7–33.3)	2.1 (1.2–3.5)	7.6 (2.7–15.6)	11.1 (4.6–21.3)	10.0 (5.8–15.6)	9.6 (5.7–14.8)	5.0 (2.0–8.1)	5.0 (2.0–8.1)	3.6 (1.5–6.4)	6.5 (2.7–11.3)	11.6 (6.2–16.7)	1.1 (0.3–2.1)	1.2 (<0.1–13.3)	1.2 (<0.1–13.3)	<0.1 (<0.1–0.8)	<0.1 (<0.1–0.8)
Thailand	34.2 (25.2–42.7)	4.0 (2.3–6.2)	2.7 (0.9–5.8)	4.0 (1.5–8.2)	13.8 (8.8–20.0)	13.6 (8.8–19.6)	7.5 (3.1–12.1)	7.5 (3.1–12.1)	4.7 (2.5–7.2)	9.6 (5.2–14.2)	14.5 (8.0–20.3)	1.6 (0.2–3.2)	2.9 (<0.1–10.6)	2.9 (<0.1–10.6)	<0.1 (<0.1–1.0)	<0.1 (<0.1–1.0)
Timor-Leste	38.5 (27.3–48.3)	4.3 (1.9–7.4)	25.3 (13.2–39.3)	32.9 (20.8–46.6)	6.2 (3.0–11.2)	5.3 (2.5–9.6)	6.6 (2.7–10.6)	6.6 (2.7–10.6)	4.9 (2.2–8.3)	9.6 (4.6–15.2)	6.7 (2.0–11.8)	0.4 (0.0–1.1)	0.5 (<0.1–2.1)	0.5 (<0.1–2.1)	<0.1 (<0.1–1.7)	<0.1 (<0.1–1.7)
Viet Nam	42.3 (33.8–50.0)	3.8 (2.1–5.8)	7.6 (3.0–14.6)	11.1 (5.2–19.5)	10.0 (6.4–14.7)	9.6 (6.3–13.8)	8.6 (3.6–13.6)	8.6 (3.6–13.6)	6.7 (3.9–9.9)	16.4 (9.8–22.8)	15.4 (7.7–22.6)	0.9 (0.0–2.1)	2.7 (0.9–5.9)	2.7 (0.9–5.9)	0.2 (<0.1–3.2)	0.2 (<0.1–3.2)
**Sub-Saharan Africa**	**18.1 (12.2–24.1)**	**3.5 (2.0–5.2)**	**30.8 (20.0–41.1)**	**36.9 (26.8–46.9)**	**11.4 (7.3–16.3)**	**9.7 (6.2–14.1)**	**21.5 (9.8–31.7)**	**21.6 (9.9–31.9)**	**3.3 (1.6–5.5)**	**4.3 (2.0–6.8)**	**7.5 (3.5–11.0)**	**2.2 (0.5–4.1)**	**3.0 (0.5–9.4)**	**3.2 (0.6–10.5)**	**1.6 (<0.1–3.4)**	**1.4 (<0.1–3.3)**
Central Sub-Saharan Africa	17.0 (10.6–23.4)	2.1 (1.1–3.4)	32.9 (20.6–44.9)	39.7 (27.7–50.5)	10.1 (5.2–17.1)	8.1 (3.9–14.1)	22.6 (10.4–33.2)	22.6 (10.5–33.3)	2.2 (0.9–3.9)	2.6 (1.1–4.5)	4.4 (0.4–8.4)	1.7 (0.0–4.0)	1.5 (0.3–3.5)	1.5 (0.3–3.4)	<0.1 (<0.1–2.4)	<0.1 (<0.1–2.4)
Angola	23.6 (17.0–30.4)	4.2 (2.2–6.9)	14.7 (7.3–24.7)	19.8 (11.5–30.0)	11.8 (6.2–19.1)	10.5 (5.5–17.2)	22.3 (10.2–32.8)	22.3 (10.2–32.8)	4.1 (2.2–6.3)	6.4 (3.4–9.6)	11.3 (5.5–16.7)	5.2 (1.6–8.8)	1.7 (0.5–3.8)	1.7 (0.5–3.8)	<0.1 (<0.1–2.9)	<0.1 (<0.1–2.9)
Central African Republic	16.2 (10.3–22.4)	2.1 (1.0–3.5)	46.8 (31.2–58.9)	51.4 (39.6–63.8)	7.9 (2.5–17.1)	5.7 (1.8–12.6)	21.6 (9.9–31.8)	21.6 (9.9–31.8)	2.9 (1.3–4.9)	3.6 (1.6–5.9)	3.2 (0.0–8.1)	1.2 (0.0–3.7)	3.8 (1.5–7.0)	3.8 (1.5–7.0)	<0.1 (<0.1–1.2)	<0.1 (<0.1–1.2)
Congo	17.3 (11.1–23.6)	2.1 (0.9–3.6)	11.8 (5.4–21.2)	16.5 (9.1–26.5)	16.5 (9.0–27.7)	15.3 (8.2–25.8)	21.0 (9.5–31.0)	21.0 (9.5–31.0)	3.3 (1.5–5.5)	4.3 (2.0–7.0)	6.4 (0.6–12.2)	3.0 (0.0–7.2)	0.9 (<0.1–3.0)	0.9 (<0.1–3.0)	<0.1 (<0.1–1.9)	<0.1 (<0.1–1.9)
Democratic Republic of the Congo	15.3 (8.6–22.2)	1.7 (0.8–3.0)	38.4 (23.9–52.2)	45.2 (32.1–56.6)	9.2 (4.4–16.5)	7.1 (3.0–13.1)	23.0 (10.6–33.7)	23.0 (10.6–33.7)	1.5 (0.4–3.2)	1.6 (0.5–3.2)	2.4 (0.0–6.7)	0.9 (0.0–3.0)	1.3 (0.1–3.1)	1.3 (0.1–3.1)	0.1 (<0.1–2.6)	0.1 (<0.1–2.6)

	Smoking		Household air pollution		Ambient particulate matter		Handwashing		Secondhand smoke		Alcohol use		High temperature		Low temperature	
	M	F	M	F	M	F	M	F	M	F	M	F	M	F	M	F
Equatorial Guinea	21.0 (13.3–29.0)	1.8 (0.8–3.2)	3.2 (1.2–6.7)	4.8 (1.9–9.3)	20.5 (10.1–33.9)	20.0 (10.1–33.1)	19.9 (9.0–29.7)	19.9 (9.0–29.7)	3.1 (1.2–5.6)	3.8 (1.6–6.6)	10.4 (4.3–16.6)	4.7 (1.1–9.2)	0.4 (<0.1–1.3)	0.4 (<0.1–1.3)	<0.1 (<0.1–2.4)	<0.1 (<0.1–2.4)
Gabon	18.0 (11.5–24.7)	2.3 (1.0–4.0)	1.2 (0.4–2.5)	1.8 (0.7–3.5)	17.8 (9.2–30.0)	17.7 (9.2–29.8)	16.9 (7.4–25.6)	16.9 (7.4–25.6)	3.5 (1.5–6.0)	4.5 (2.0–7.5)	12.2 (5.4–18.9)	5.9 (1.7–10.5)	0.7 (<0.1–2.6)	0.7 (<0.1–2.6)	<0.1 (<0.1–1.5)	<0.1 (<0.1–1.5)
Eastern Sub-Saharan Africa	17.1 (10.7–23.7)	3.9 (2.1–6.1)	39.1 (26.4–51.1)	45.5 (33.6–56.3)	7.1 (3.9–11.0)	5.6 (3.1–8.8)	22.5 (10.4–33.1)	22.5 (10.4–33.1)	2.6 (1.0–4.6)	3.6 (1.5–6.3)	7.1 (2.9–10.8)	2.3 (0.7–4.0)	2.0 (0.3–5.5)	1.9 (0.3–5.2)	1.6 (<0.1–4.1)	1.5 (<0.1–4.0)
Burundi	15.5 (9.1–22.8)	4.2 (1.7–7.4)	48.3 (32.9–60.1)	52.7 (40.8–64.6)	5.9 (2.0–12.2)	4.2 (1.3–9.5)	22.9 (10.6–33.7)	22.9 (10.6–33.7)	2.0 (0.7–3.7)	2.4 (0.9–4.5)	12.8 (6.2–19.3)	2.3 (0.6–4.2)	<0.1 (<0.1–0.3)	<0.1 (<0.1–0.3)	<0.1 (<0.1–6.1)	<0.1 (<0.1–6.1)
Comoros	18.2 (10.6–26.1)	2.9 (1.4–5.1)	29.2 (16.9–43.5)	36.9 (24.5–49.6)	6.2 (3.4–9.9)	5.1 (2.8–8.2)	21.0 (9.6–31.1)	21.0 (9.6–31.1)	5.4 (2.5–8.8)	6.5 (3.1–10.4)	0.8 (0.0–2.6)	0.3 (0.0–1.1)	0.7 (<0.1–2.9)	0.7 (<0.1–2.9)	0.2 (<0.1–2.9)	0.2 (<0.1–2.9)
Djibouti	29.8 (19.5–39.6)	3.9 (1.9–6.6)	6.5 (2.7–13.2)	9.3 (4.2–17.1)	18.6 (9.1–32.2)	17.5 (8.5–30.7)	21.6 (9.9–32.0)	21.6 (9.9–32.0)	4.5 (2.0–7.8)	8.7 (3.9–14.4)	0.3 (0.0–1.9)	0.1 (0.0–0.5)	5.9 (<0.1–30.0)	5.9 (<0.1–30.0)	0.2 (<0.1–5.2)	0.2 (<0.1–5.2)
Eritrea	16.0 (10.4–21.7)	0.6 (0.3–1.0)	29.3 (16.5–42.7)	35.9 (23.0–47.4)	12.8 (6.3–21.5)	10.4 (4.9–17.7)	22.6 (10.4–33.1)	22.6 (10.4–33.1)	4.1 (2.0–6.7)	4.0 (2.2–6.0)	2.0 (0.0–5.8)	0.4 (0.0–1.6)	4.5 (<0.1–22.1)	4.5 (<0.1–22.1)	0.1 (<0.1–2.5)	0.1 (<0.1–2.5)
Ethiopia	9.0 (5.3–12.8)	0.9 (0.4–1.6)	44.0 (29.7–55.6)	48.8 (37.2–59.8)	7.2 (3.3–12.4)	5.5 (2.6–10.0)	23.0 (10.6–33.7)	22.9 (10.7–33.7)	1.5 (0.5–2.9)	1.5 (0.5–2.9)	4.9 (1.0–9.4)	1.5 (0.0–3.8)	3.2 (0.6–10.3)	3.2 (0.6–10.2)	4.6 (3.0–6.6)	4.6 (3.0–6.6)
Kenya	18.8 (11.9–25.9)	2.7 (1.3–4.5)	26.5 (16.0–38.2)	33.2 (22.3–44.4)	7.5 (4.4–11.5)	6.5 (3.8–10.0)	22.1 (10.2–32.6)	22.2 (10.3–32.7)	2.6 (1.1–4.5)	3.9 (1.7–6.6)	8.2 (2.7–14.0)	2.3 (0.7–4.2)	2.5 (0.2–10.1)	2.2 (0.2–8.9)	3.6 (1.7–5.9)	3.6 (1.5–6.0)
Madagascar	16.7 (9.5–24.3)	2.2 (1.0–3.8)	39.8 (24.8–54.2)	47.2 (33.7–59.0)	5.2 (2.7–8.8)	4.0 (2.0–6.9)	22.4 (10.3–33.0)	22.4 (10.3–33.0)	2.7 (0.8–5.3)	3.5 (1.1–6.9)	2.7 (0.0–7.1)	0.6 (0.0–1.9)	0.8 (<0.1–2.4)	0.8 (<0.1–2.4)	1.0 (<0.1–3.8)	1.0 (<0.1–3.8)
Malawi	21.8 (13.5–30.5)	4.4 (2.0–7.4)	42.6 (27.7–56.0)	49.0 (35.8–59.8)	5.6 (2.6–10.0)	4.3 (1.9–7.9)	22.6 (10.4–33.3)	22.6 (10.4–33.3)	2.9 (1.2–5.1)	3.5 (1.4–6.1)	5.1 (1.7–9.0)	1.5 (0.0–3.7)	0.5 (<0.1–1.9)	0.5 (<0.1–1.9)	0.2 (<0.1–4.4)	0.2 (<0.1–4.4)
Mozambique	18.2 (10.7–26.3)	5.0 (2.4–8.2)	47.2 (31.8–59.3)	52.0 (40.4–62.6)	4.4 (1.9–8.0)	3.2 (1.3–6.1)	22.2 (10.2–32.7)	22.2 (10.2–32.7)	2.3 (0.8–4.4)	3.2 (1.1–6.2)	2.2 (0.0–6.5)	0.6 (0.0–2.4)	2.4 (1.0–4.4)	2.4 (1.0–4.4)	0.2 (<0.1–3.3)	0.2 (<0.1–3.3)
Rwanda	22.5 (14.8–30.7)	11.1 (6.7–16.8)	34.8 (21.1–49.2)	42.5 (29.4–55.2)	10.4 (4.9–18.0)	8.3 (3.7–15.3)	23.2 (10.7–34.0)	23.2 (10.7–34.0)	3.2 (1.6–5.1)	4.7 (2.4–7.4)	13.7 (6.0–20.8)	4.4 (0.9–8.5)	<0.1 (<0.1–0.1)	<0.1 (<0.1–0.1)	2.0 (<0.1–7.5)	2.0 (<0.1–7.5)
Somalia	19.1 (11.1–27.0)	2.9 (1.3–5.2)	58.4 (44.7–79.9)	61.4 (46.5–85.0)	2.1 (0.5–6.7)	1.4 (0.3–4.3)	22.7 (10.5–33.4)	22.7 (10.5–33.4)	2.9 (1.2–5.2)	4.0 (1.7–7.1)	0.0 (0.0–0.0)	0.0 (0.0–0.0)	2.3 (<0.1–15.8)	2.3 (<0.1–15.8)	0.1 (<0.1–1.3)	0.1 (<0.1–1.3)
South Sudan	18.9 (11.2–27.5)	2.8 (1.2–5.0)	40.0 (25.6–53.9)	46.8 (33.8–58.2)	9.3 (4.3–15.8)	7.1 (3.2–12.7)	22.9 (10.6–33.6)	22.9 (10.6–33.6)	3.0 (1.3–5.2)	4.3 (1.8–7.5)	0.3 (0.0–1.7)	0.1 (0.0–0.5)	5.9 (<0.1–23.1)	5.9 (<0.1–23.1)	<0.1 (<0.1–0.9)	<0.1 (<0.1–0.9)
Uganda	14.5 (8.0–21.0)	4.4 (2.0–7.3)	37.3 (23.0–51.3)	44.7 (31.4–56.3)	9.5 (5.0–15.1)	7.5 (3.7–12.3)	22.1 (10.1–32.5)	22.1 (10.1–32.5)	2.6 (1.0–4.7)	3.0 (1.2–5.4)	15.1 (8.2–21.4)	6.3 (2.5–10.4)	1.1 (0.2–2.9)	1.1 (0.2–2.9)	<0.1 (<0.1–2.3)	<0.1 (<0.1–2.3)
United Republic of Tanzania	25.6 (16.8–34.7)	8.6 (4.3–13.5)	36.6 (22.4–51.1)	44.3 (30.7–56.4)	7.4 (4.1–11.4)	5.9 (3.2–9.2)	22.5 (10.4–33.2)	22.5 (10.4–33.2)	3.6 (1.7–6.0)	6.2 (3.0–9.9)	13.6 (7.2–19.7)	4.8 (1.7–8.0)	0.9 (0.1–2.7)	0.9 (0.1–2.7)	0.1 (<0.1–3.5)	0.1 (<0.1–3.5)
Zambia	18.1 (10.2–26.1)	4.7 (2.0–7.9)	23.8 (12.7–36.8)	31.1 (19.5–43.4)	9.9 (5.7–15.7)	8.5 (4.9–13.4)	21.3 (9.7–31.5)	21.3 (9.7–31.5)	2.6 (0.8–5.1)	4.2 (1.3–8.2)	7.6 (2.2–12.7)	2.8 (0.5–5.4)	1.2 (0.3–3.5)	1.2 (0.3–3.5)	<0.1 (<0.1–5.0)	<0.1 (<0.1–5.0)

	Smoking		Household air pollution		Ambient particulate matter		Handwashing		Secondhand smoke		Alcohol use		High temperature		Low temperature	
	M	F	M	F	M	F	M	F	M	F	M	F	M	F	M	F
Southern Sub-Saharan Africa	27.2 (19.5–34.9)	7.5 (4.6–10.6)	9.4 (4.6–15.7)	12.1 (6.9–18.7)	12.5 (7.9–18.1)	12.1 (7.8–17.3)	16.8 (7.5–25.2)	16.6 (7.5–24.8)	4.5 (2.2–7.2)	7.6 (3.9–12.0)	11.2 (6.0–15.8)	3.0 (1.2–4.9)	1.2 (0.5–2.4)	1.1 (0.4–2.3)	6.5 (3.8–9.2)	6.7 (4.0–9.4)
Botswana	29.9 (22.5–37.2)	8.7 (5.2–12.5)	6.6 (2.5–13.1)	9.6 (4.3–17.5)	12.1 (7.2–18.9)	11.6 (7.0–17.6)	17.0 (7.5–25.6)	17.0 (7.5–25.6)	6.3 (3.0–10.3)	9.0 (4.5–14.2)	10.6 (5.1–15.8)	2.0 (0.5–3.6)	4.0 (1.5–7.7)	4.0 (1.5–7.7)	0.9 (<0.1–7.0)	0.9 (<0.1–7.0)
Eswatini	13.2 (8.0–18.8)	2.4 (1.2–4.0)	12.3 (5.5–22.2)	17.3 (9.1–27.7)	10.8 (6.3–16.4)	9.9 (6.0–14.8)	16.5 (7.4–25.0)	16.5 (7.4–25.0)	2.3 (0.9–4.2)	3.6 (1.5–6.3)	11.7 (6.5–16.7)	3.4 (1.2–5.9)	0.4 (<0.1–1.2)	0.4 (<0.1–1.2)	3.3 (<0.1–8.1)	3.3 (<0.1–8.1)
Lesotho	32.0 (24.3–39.5)	3.0 (1.7–5.0)	17.7 (8.8–29.2)	23.8 (13.9–35.1)	11.3 (6.7–17.1)	10.0 (5.9–15.3)	23.1 (10.7–33.9)	23.1 (10.7–33.9)	6.0 (3.3–9.0)	11.9 (7.0–17.0)	8.4 (2.4–14.1)	1.9 (0.0–4.2)	<0.1 (<0.1–<0.1)	<0.1 (<0.1–<0.1)	21.7 (15.2–28.4)	21.7 (15.2–28.4)
Namibia	16.0 (9.4–22.8)	10.4 (6.2–15.6)	9.2 (3.8–17.1)	13.2 (6.4–22.5)	11.5 (6.4–18.2)	10.9 (6.1–16.6)	14.3 (6.2–22.0)	14.3 (6.2–22.0)	3.5 (1.4–6.3)	4.7 (1.8–8.5)	11.6 (4.7–18.1)	3.7 (0.2–7.9)	3.2 (1.3–6.0)	3.2 (1.3–6.0)	1.3 (<0.1–6.0)	1.3 (<0.1–6.0)
South Africa	25.7 (17.7–33.9)	8.4 (5.0–11.9)	2.3 (0.9–4.6)	3.1 (1.3–5.6)	14.7 (9.2–21.4)	14.6 (9.3–21.1)	14.6 (6.4–22.2)	14.3 (6.3–22.1)	4.7 (2.2–7.8)	7.1 (3.4–11.6)	13.4 (7.7–18.4)	3.9 (1.7–6.3)	0.7 (0.3–1.9)	0.6 (0.3–1.6)	8.6 (6.4–11.0)	8.8 (6.6–11.2)
Zimbabwe	31.5 (23.5–39.7)	5.9 (3.5–9.0)	24.3 (13.1–37.7)	31.4 (20.2–44.4)	8.0 (4.7–12.6)	6.8 (3.9–10.6)	20.9 (9.6–31.0)	20.9 (9.6–31.0)	3.7 (1.9–5.7)	8.6 (4.7–12.9)	6.5 (1.6–12.2)	1.0 (0.0–2.6)	1.7 (0.7–3.8)	1.7 (0.7–3.8)	0.7 (<0.1–5.1)	0.7 (<0.1–5.1)
Western Sub-Saharan Africa	16.0 (11.0–21.2)	2.6 (1.6–4.0)	30.3 (19.6–41.3)	35.2 (25.4–45.7)	15.5 (9.9–22.8)	13.3 (8.4–19.6)	21.8 (10.0–32.1)	21.7 (10.0–32.0)	4.0 (2.1–6.3)	4.6 (2.4–7.0)	7.9 (3.8–11.4)	2.2 (0.4–4.2)	5.3 (0.4–18.7)	5.7 (0.5–22.2)	0.5 (<0.1–2.5)	0.5 (<0.1–2.7)
Benin	15.4 (10.3–21.3)	3.3 (1.8–5.4)	37.7 (23.4–51.9)	44.3 (31.5–55.4)	11.3 (5.6–18.8)	8.8 (4.1–15.1)	22.1 (10.1–32.6)	22.1 (10.1–32.6)	3.7 (1.8–5.9)	4.5 (2.3–7.2)	5.2 (1.6–9.2)	1.4 (0.0–3.6)	3.3 (<0.1–18.5)	3.3 (<0.1–18.5)	<0.1 (<0.1–1.2)	<0.1 (<0.1–1.2)
Burkina Faso	14.5 (8.6–20.6)	1.2 (0.6–2.2)	46.4 (30.9–59.2)	50.8 (38.4–64.4)	8.4 (2.9–16.6)	6.0 (2.0–12.9)	22.2 (10.2–32.7)	22.2 (10.2–32.7)	3.3 (1.4–5.8)	4.4 (1.9–7.8)	10.8 (4.2–17.6)	5.8 (0.4–11.8)	4.6 (<0.1–34.1)	4.6 (<0.1–34.1)	<0.1 (<0.1–3.1)	<0.1 (<0.1–3.1)
Cabo Verde	16.0 (10.8–21.4)	4.0 (2.0–6.4)	5.5 (2.4–10.4)	8.0 (4.1–13.7)	22.0 (13.6–31.9)	21.0 (13.2–30.6)	19.0 (8.5–28.3)	19.0 (8.5–28.3)	4.0 (1.9–6.5)	4.5 (2.1–7.2)	10.1 (5.1–15.0)	4.0 (1.0–7.3)	0.2 (<0.1–1.1)	0.2 (<0.1–1.1)	<0.1 (<0.1–6.3)	<0.1 (<0.1–6.3)
Cameroon	21.4 (15.1–28.4)	1.4 (0.7–2.5)	19.3 (10.4–30.8)	25.8 (15.8–37.7)	20.9 (13.2–29.7)	18.3 (11.5–26.1)	21.8 (10.0–32.1)	21.8 (10.0–32.1)	2.6 (1.3–4.2)	4.2 (2.1–6.8)	11.2 (5.8–16.5)	4.6 (1.5–8.2)	2.0 (0.1–7.1)	2.0 (0.1–7.1)	<0.1 (<0.1–1.6)	<0.1 (<0.1–1.6)
Chad	16.6 (10.3–23.3)	2.6 (1.2–4.5)	47.3 (32.6–60.6)	51.6 (39.7–65.9)	8.4 (2.9–17.8)	6.0 (2.1–13.8)	22.3 (10.2–32.8)	22.3 (10.2–32.8)	3.1 (1.3–5.2)	4.6 (2.1–7.7)	4.9 (0.6–9.4)	2.8 (0.0–7.0)	7.0 (0.4–22.9)	7.0 (0.4–22.9)	1.3 (<0.1–7.7)	1.3 (<0.1–7.7)
Côte d'Ivoire	21.6 (15.3–27.9)	4.1 (2.2–6.8)	29.4 (17.1–43.7)	36.6 (24.5–48.9)	15.5 (8.3–25.4)	12.7 (6.4–21.5)	20.8 (9.5–31.0)	20.8 (9.5–31.0)	6.4 (3.4–9.5)	7.8 (4.3–11.8)	8.9 (2.5–15.0)	2.7 (0.0–6.2)	1.6 (<0.1–5.7)	1.6 (<0.1–5.7)	0.2 (<0.1–1.2)	0.2 (<0.1–1.2)
Gambia	24.6 (17.4–31.7)	1.4 (0.7–2.5)	34.9 (21.2–48.8)	41.9 (28.7–53.4)	14.2 (7.3–23.0)	11.3 (5.6–19.1)	21.9 (10.1–32.3)	21.9 (10.1–32.3)	5.9 (3.1–8.9)	8.9 (4.7–13.5)	5.9 (2.1–10.1)	1.7 (0.0–4.0)	2.2 (<0.1–20.9)	2.2 (<0.1–20.9)	0.1 (<0.1–1.5)	0.1 (<0.1–1.5)
Ghana	12.7 (7.7–18.0)	2.1 (1.1–3.4)	14.2 (7.1–23.9)	19.7 (11.2–30.3)	20.3 (12.9–30.3)	18.4 (11.5–27.6)	22.3 (10.3–32.8)	22.3 (10.3–32.8)	2.5 (1.1–4.2)	2.5 (1.2–4.2)	9.4 (3.2–15.3)	2.1 (0.0–4.7)	2.9 (<0.1–18.5)	2.9 (<0.1–18.5)	<0.1 (<0.1–0.7)	<0.1 (<0.1–0.7)
Guinea	28.3 (19.7–36.9)	2.6 (1.3–4.6)	43.6 (28.5–56.2)	48.8 (36.2–60.4)	9.9 (4.2–17.7)	7.3 (3.1–14.2)	22.3 (10.3–32.8)	22.3 (10.3–32.8)	4.4 (2.0–6.9)	5.9 (2.9–9.0)	2.6 (0.4–5.1)	0.4 (0.0–1.3)	2.7 (0.7–7.1)	2.7 (0.7–7.1)	0.1 (<0.1–1.3)	0.1 (<0.1–1.3)
Guinea-Bissau	11.2 (7.4–15.5)	1.5 (0.8–2.4)	41.0 (25.8–54.2)	46.9 (33.9–58.0)	11.2 (5.0–19.4)	8.5 (3.7–15.5)	22.3 (10.3–32.8)	22.3 (10.3–32.8)	4.4 (2.2–6.9)	5.7 (2.9–8.7)	6.6 (2.4–10.8)	1.8 (0.0–4.2)	2.2 (<0.1–22.7)	2.2 (<0.1–22.7)	<0.1 (<0.1–1.3)	<0.1 (<0.1–1.3)

	Smoking		Household air pollution		Ambient particulate matter		Handwashing		Secondhand smoke		Alcohol use		High temperature		Low temperature	
	M	F	M	F	M	F	M	F	M	F	M	F	M	F	M	F
Liberia	17.9 (11.4–25.2)	2.9 (1.4–4.8)	38.4 (23.3–51.5)	44.9 (32.1–56.0)	11.8 (5.7–20.2)	9.0 (4.4–15.8)	23.3 (10.8–34.1)	23.3 (10.8–34.1)	2.4 (1.0–4.3)	3.4 (1.5–5.8)	7.3 (2.6–12.1)	3.0 (0.7–5.5)	0.9 (<0.1–3.3)	0.9 (<0.1–3.3)	0.1 (<0.1–2.0)	0.1 (<0.1–2.0)
Mali	18.0 (11.7–25.0)	3.4 (1.6–5.9)	46.8 (31.2–59.6)	51.3 (38.0–65.0)	9.0 (3.1–17.7)	6.5 (2.3–13.8)	21.9 (10.1–32.3)	21.9 (10.1–32.3)	3.3 (1.4–5.6)	4.4 (2.0–7.2)	1.9 (0.7–3.3)	1.4 (0.2–2.9)	7.3 (<0.1–26.7)	7.3 (<0.1–26.7)	1.3 (<0.1–13.3)	1.3 (<0.1–13.3)
Mauritania	21.4 (14.4–28.8)	5.3 (2.7–8.8)	14.9 (7.2–25.1)	20.4 (11.4–30.8)	22.6 (13.2–34.5)	20.3 (11.6–31.1)	19.6 (8.8–29.2)	19.6 (8.8–29.2)	3.5 (1.5–6.1)	4.7 (2.1–8.1)	<0.1 (0.0–<0.1)	<0.1 (0.0–0.0)	8.0 (1.7–19.5)	8.0 (1.7–19.5)	1.9 (<0.1–13.0)	1.9 (<0.1–13.0)
Niger	10.2 (6.4–14.6)	1.6 (0.7–2.7)	51.0 (35.2–69.0)	55.1 (40.4–75.8)	7.5 (2.1–19.0)	5.3 (1.5–14.5)	21.4 (9.8–31.7)	21.4 (9.8–31.7)	3.8 (1.9–6.3)	4.6 (2.4–7.1)	0.4 (0.0–1.7)	0.1 (0.0–0.6)	8.4 (0.5–23.3)	8.4 (0.5–23.3)	1.6 (<0.1–11.5)	1.6 (<0.1–11.5)
Nigeria	11.0 (7.6–14.9)	2.6 (1.4–4.3)	25.8 (16.3–36.4)	27.0 (18.6–37.0)	18.4 (11.3–27.5)	17.1 (10.4–25.1)	21.9 (10.0–32.1)	21.7 (10.0–32.0)	4.6 (2.5–7.2)	3.7 (2.0–5.5)	10.1 (5.4–14.6)	2.0 (0.2–4.4)	9.1 (1.3–40.2)	8.1 (0.9–37.2)	1.0 (<0.1–2.8)	0.8 (<0.1–2.3)
Sao Tome and Principe	15.0 (10.1–20.5)	2.3 (1.1–4.0)	17.3 (8.5–28.6)	23.3 (13.5–34.4)	12.5 (6.9–19.8)	11.0 (6.0–17.6)	17.4 (7.7–26.1)	17.4 (7.7–26.1)	2.1 (1.0–3.3)	2.3 (1.1–3.6)	11.0 (5.5–16.5)	4.4 (1.2–8.0)	0.9 (<0.1–3.7)	0.9 (<0.1–3.7)	<0.1 (<0.1–1.9)	<0.1 (<0.1–1.9)
Senegal	21.5 (15.5–28.0)	2.3 (1.1–4.1)	32.1 (18.7–45.3)	38.6 (26.3–50.1)	15.1 (7.9–24.1)	12.0 (5.9–20.1)	18.8 (8.4–28.1)	18.8 (8.4–28.1)	7.0 (3.9–10.4)	9.3 (5.3–13.5)	0.9 (<0.1–1.9)	0.2 (0.0–0.6)	4.2 (<0.1–30.1)	4.2 (<0.1–30.1)	0.1 (<0.1–2.2)	0.1 (<0.1–2.2)
Sierra Leone	25.7 (16.4–34.8)	6.4 (3.1–10.2)	41.7 (26.6–54.7)	47.5 (34.8–58.5)	10.5 (4.5–18.6)	7.9 (3.5–14.6)	22.2 (10.2–32.6)	22.2 (10.2–32.6)	3.6 (1.4–6.5)	5.7 (2.3–10.0)	8.4 (3.6–13.2)	3.1 (0.5–6.0)	1.8 (<0.1–4.3)	1.8 (<0.1–4.3)	0.2 (<0.1–1.5)	0.2 (<0.1–1.5)
Togo	24.1 (18.3–30.8)	5.2 (3.1–8.1)	32.2 (19.3–46.2)	39.8 (27.4–51.7)	13.0 (7.0–20.7)	10.5 (5.7–16.7)	22.0 (10.1–32.5)	22.0 (10.1–32.5)	5.3 (3.1–7.8)	6.1 (3.6–8.9)	5.6 (1.7–9.9)	1.5 (0.0–3.8)	2.7 (<0.1–13.0)	2.7 (<0.1–13.0)	0.1 (<0.1–0.9)	0.1 (<0.1–0.9)

Appendix table 11. Population-attributable fractions of lower respiratory infections due to evaluated risk factors among people aged 70 years and older in 2019, males (M) and females (F) (95% UI)

	Smoking		Household air pollution		Ambient particulate matter		Handwashing		Secondhand smoke		Alcohol use		High temperature		Low temperature	
	M	F	M	F	M	F	M	F	M	F	M	F	M	F	M	F
**Global**	**21.9 (16.8–27.3)**	**6.0 (4.0–8.0)**	**8.2 (4.9–12.3)**	**10.2 (6.8–14.2)**	**12.8 (8.9–17.2)**	**11.7 (8.2–15.8)**	**7.2 (3.1–11.2)**	**7.1 (3.1–10.9)**	**5.9 (3.5–8.7)**	**7.3 (4.3–10.5)**	**6.3 (2.8–9.4)**	**1.8 (0.5–3.2)**	**2.3 (1.1–4.8)**	**2.2 (1.1–4.9)**	**9.0 (6.5–11.7)**	**9.0 (6.6–11.8)**
**Central Europe, Eastern Europe, and Central Asia**	**28.1 (22.0–34.1)**	**5.8 (3.8–7.9)**	**0.8 (0.3–1.9)**	**1.2 (0.4–2.5)**	**8.9 (5.4–13.5)**	**8.7 (5.2–13.2)**	**1.8 (0.7–2.9)**	**1.8 (0.7–3.0)**	**6.7 (4.1–9.5)**	**8.5 (5.2–12.0)**	**11.6 (5.9–16.3)**	**1.6 (0.1–3.2)**	**<0.1 (<0.1–0.3)**	**<0.1 (<0.1–0.3)**	**19.3 (11.6–28.2)**	**19.1 (11.3–28.1)**
Central Asia	28.1 (22.0–34.4)	1.6 (0.9–2.3)	2.4 (0.9–4.6)	3.2 (1.4–6.2)	13.2 (7.6–20.2)	12.8 (7.4–19.6)	4.2 (1.7–6.8)	4.1 (1.6–6.6)	8.3 (5.1–11.7)	10.8 (6.8–14.9)	3.9 (1.2–6.7)	0.5 (0.0–1.4)	0.5 (<0.1–1.4)	0.5 (<0.1–1.4)	18.3 (12.2–26.2)	18.3 (12.2–25.9)
Armenia	34.9 (27.0–42.4)	0.8 (0.5–1.4)	0.3 (0.1–0.8)	0.5 (0.2–1.3)	16.8 (9.1–26.9)	16.8 (9.2–26.9)	3.8 (1.5–6.2)	3.8 (1.5–6.2)	10.1 (6.3–14.1)	14.3 (9.2–19.5)	1.5 (0.0–3.7)	0.5 (0.0–1.4)	<0.1 (<0.1–0.2)	<0.1 (<0.1–0.2)	18.4 (8.4–31.6)	18.4 (8.4–31.6)
Azerbaijan	39.4 (32.1–47.1)	0.6 (0.3–1.1)	0.9 (0.3–2.0)	1.3 (0.4–2.9)	12.9 (6.1–22.6)	12.9 (6.2–22.4)	3.5 (1.4–5.7)	3.5 (1.4–5.7)	10.3 (6.5–14.5)	15.4 (10.2–21.4)	7.5 (2.5–12.7)	1.1 (0.0–2.8)	0.5 (<0.1–1.1)	0.5 (<0.1–1.1)	15.7 (12.6–19.1)	15.7 (12.6–19.1)
Georgia	30.2 (23.6–36.9)	2.8 (1.7–4.3)	3.9 (1.2–8.5)	5.8 (2.1–12.1)	9.1 (5.3–14.5)	9.0 (5.3–13.7)	3.3 (1.3–5.4)	3.3 (1.3–5.4)	10.6 (6.7–14.9)	14.3 (9.3–19.4)	6.2 (2.1–10.2)	0.2 (0.0–0.8)	<0.1 (<0.1–0.2)	<0.1 (<0.1–0.2)	18.9 (11.2–29.0)	18.9 (11.2–29.0)
Kazakhstan	28.6 (21.8–35.4)	2.0 (1.1–3.2)	1.2 (0.3–2.8)	1.8 (0.6–4.2)	10.3 (5.4–16.8)	10.3 (5.4–16.6)	3.4 (1.4–5.7)	3.4 (1.4–5.7)	6.5 (3.7–9.9)	8.9 (5.2–12.9)	4.1 (0.7–8.1)	0.7 (0.0–2.2)	0.5 (<0.1–2.0)	0.5 (<0.1–2.0)	18.4 (11.3–27.6)	18.4 (11.3–27.6)
Kyrgyzstan	30.7 (24.3–37.4)	1.6 (0.9–2.4)	5.4 (2.2–10.4)	7.9 (3.6–14.5)	11.8 (6.3–19.4)	11.3 (6.2–18.4)	4.0 (1.6–6.5)	4.0 (1.6–6.5)	10.7 (6.8–14.9)	15.2 (9.9–20.4)	3.3 (1.0–6.0)	0.2 (0.0–0.7)	<0.1 (<0.1–<0.1)	<0.1 (<0.1–<0.1)	21.4 (8.4–37.4)	21.4 (8.4–37.4)
Mongolia	31.9 (24.2–39.2)	6.0 (3.3–9.2)	6.5 (2.6–12.6)	9.5 (4.3–16.9)	17.3 (10.9–24.5)	16.6 (10.7–23.5)	8.5 (3.6–13.6)	8.5 (3.6–13.6)	5.5 (3.0–8.4)	8.6 (4.9–12.7)	5.9 (1.9–10.2)	0.3 (0.0–1.2)	0.2 (<0.1–0.9)	0.2 (<0.1–0.9)	18.3 (3.6–34.9)	18.3 (3.6–34.9)
Tajikistan	21.9 (16.1–28.3)	1.0 (0.6–1.7)	7.5 (3.2–14.0)	10.7 (5.3–18.2)	16.9 (8.1–28.7)	15.9 (7.7–27.1)	7.1 (2.9–11.3)	7.1 (2.9–11.3)	8.9 (5.5–12.7)	7.3 (4.6–10.5)	1.7 (0.0–3.8)	0.1 (0.0–0.4)	0.3 (0.1–0.5)	0.3 (0.1–0.5)	21.4 (9.3–34.9)	21.4 (9.3–34.9)
Turkmenistan	20.7 (15.6–26.3)	1.1 (0.6–1.8)	<0.1 (<0.1–<0.1)	<0.1 (<0.1–0.1)	13.3 (6.3–23.0)	13.3 (6.3–23.0)	4.6 (1.9–7.4)	4.6 (1.9–7.4)	13.7 (9.0–18.8)	15.8 (10.4–21.4)	4.6 (1.2–8.4)	0.2 (0.0–0.9)	2.6 (0.9–4.3)	2.6 (0.9–4.3)	12.7 (10.0–15.4)	12.7 (10.0–15.4)
Uzbekistan	20.7 (14.7–27.3)	0.9 (0.5–1.7)	1.6 (0.6–3.5)	2.4 (0.9–5.0)	16.9 (8.4–28.0)	16.7 (8.4–27.5)	4.5 (1.8–7.3)	4.5 (1.8–7.3)	6.5 (3.5–9.8)	7.4 (4.3–10.9)	2.1 (0.1–4.3)	0.1 (0.0–0.6)	1.0 (<0.1–2.7)	1.0 (<0.1–2.7)	18.0 (13.9–22.0)	18.0 (13.9–22.0)
Central Europe	24.4 (18.6–30.2)	8.7 (5.8–11.7)	1.0 (0.3–2.4)	1.5 (0.4–3.3)	10.2 (6.3–15.1)	10.2 (6.2–15.0)	1.0 (0.4–1.7)	1.0 (0.4–1.7)	7.6 (4.6–10.7)	8.6 (5.2–12.2)	13.9 (7.6–19.3)	1.7 (<0.1–3.5)	<0.1 (<0.1–0.1)	<0.1 (<0.1–0.1)	21.1 (14.7–28.8)	21.1 (14.5–28.9)
Albania	39.5 (31.9–47.3)	8.6 (5.5–12.6)	3.8 (1.2–8.2)	5.6 (2.0–11.7)	9.5 (5.8–14.4)	9.4 (5.8–14.0)	1.7 (0.7–2.9)	1.7 (0.7–2.9)	11.9 (7.7–16.7)	14.9 (9.8–20.2)	5.5 (1.2–10.2)	0.4 (0.0–1.4)	<0.1 (<0.1–0.1)	<0.1 (<0.1–0.1)	19.2 (14.6–24.1)	19.2 (14.6–24.1)
Bosnia and Herzegovina	35.5 (28.3–42.7)	15.0 (10.9–19.8)	3.8 (1.2–8.5)	5.6 (2.0–11.7)	14.6 (9.3–20.5)	14.3 (9.3–20.0)	1.3 (0.5–2.1)	1.3 (0.5–2.1)	11.2 (7.0–15.7)	12.7 (8.2–17.8)	7.3 (2.7–11.7)	0.5 (0.0–1.8)	<0.1 (<0.1–<0.1)	<0.1 (<0.1–<0.1)	22.3 (15.8–29.9)	22.3 (15.8–29.9)
Bulgaria	27.1 (21.0–33.7)	6.3 (4.2–8.9)	1.6 (0.4–4.2)	2.5 (0.7–5.8)	10.0 (6.2–14.9)	9.9 (6.2–14.7)	1.1 (0.4–1.8)	1.1 (0.4–1.8)	6.9 (4.1–9.9)	9.7 (6.0–13.6)	15.1 (8.4–21.0)	2.2 (0.2–4.6)	<0.1 (<0.1–<0.1)	<0.1 (<0.1–<0.1)	21.3 (14.8–27.9)	21.3 (14.8–27.9)
Croatia	26.9 (20.2–33.4)	11.2 (7.6–15.7)	0.4 (0.1–1.1)	0.6 (0.2–1.7)	9.4 (5.7–14.3)	9.4 (5.7–14.2)	1.0 (0.4–1.6)	1.0 (0.4–1.6)	10.1 (6.1–14.5)	12.2 (7.7–17.0)	13.8 (6.1–20.7)	2.0 (0.0–5.0)	<0.1 (<0.1–0.1)	<0.1 (<0.1–0.1)	20.6 (13.3–27.4)	20.6 (13.3–27.4)

	Smoking		Household air pollution		Ambient particulate matter		Handwashing		Secondhand smoke		Alcohol use		High temperature		Low temperature	
	M	F	M	F	M	F	M	F	M	F	M	F	M	F	M	F
Czechia	25.0 (19.2–30.9)	11.2 (7.4–15.4)	0.1 (<0.1–0.2)	0.1 (<0.1–0.4)	8.4 (5.0–13.0)	8.4 (5.0–13.0)	0.9 (0.3–1.4)	0.9 (0.3–1.4)	9.2 (5.7–13.2)	6.3 (3.8–9.0)	16.9 (9.0–23.7)	4.9 (1.4–9.0)	<0.1 (<0.1–0.2)	<0.1 (<0.1–0.2)	21.1 (13.6–30.0)	21.1 (13.6–30.0)
Hungary	22.3 (16.5–28.6)	7.5 (4.9–10.5)	1.3 (0.3–3.6)	2.0 (0.5–5.4)	8.4 (5.0–12.9)	8.4 (5.1–12.8)	0.9 (0.4–1.4)	0.9 (0.4–1.4)	10.1 (6.3–14.3)	10.4 (6.5–14.7)	15.9 (8.1–22.5)	2.2 (0.1–4.8)	<0.1 (<0.1–0.2)	<0.1 (<0.1–0.2)	21.4 (11.9–30.6)	21.4 (11.9–30.6)
Montenegro	35.0 (27.7–42.4)	16.1 (11.0–22.3)	2.7 (0.7–6.4)	4.0 (1.1–9.1)	10.9 (6.8–16.1)	10.8 (6.8–15.8)	1.2 (0.5–2.0)	1.2 (0.5–2.0)	12.9 (8.2–17.8)	14.5 (9.3–19.9)	13.6 (6.7–20.1)	1.8 (0.0–4.4)	<0.1 (<0.1–0.1)	<0.1 (<0.1–0.1)	20.4 (14.1–28.1)	20.4 (14.1–28.1)
North Macedonia	30.7 (23.8–37.5)	13.6 (9.1–18.9)	2.4 (0.7–5.6)	3.6 (1.2–7.8)	15.2 (9.7–21.6)	15.0 (9.7–21.2)	1.0 (0.4–1.7)	1.0 (0.4–1.7)	11.9 (7.4–16.6)	12.7 (8.0–17.8)	11.1 (4.8–16.9)	0.6 (0.0–2.0)	<0.1 (<0.1–0.1)	<0.1 (<0.1–0.1)	21.8 (15.6–28.1)	21.8 (15.6–28.1)
Poland	23.9 (18.1–29.5)	9.0 (5.8–12.4)	0.9 (0.2–2.4)	1.4 (0.4–3.4)	11.5 (7.2–17.0)	11.5 (7.2–16.9)	1.0 (0.4–1.6)	1.0 (0.4–1.6)	6.0 (3.4–9.0)	8.1 (4.7–11.9)	13.8 (7.8–19.3)	0.4 (0.0–1.5)	<0.1 (<0.1–0.2)	<0.1 (<0.1–0.2)	21.1 (13.4–30.2)	21.1 (13.4–30.2)
Romania	21.5 (15.2–28.2)	6.4 (3.9–9.4)	1.1 (0.3–2.7)	1.6 (0.4–4.1)	7.9 (4.7–12.1)	7.9 (4.8–12.1)	1.2 (0.5–2.0)	1.2 (0.5–2.0)	7.8 (4.5–11.2)	9.0 (5.2–13.1)	13.6 (6.9–20.1)	3.0 (0.4–5.7)	<0.1 (<0.1–0.1)	<0.1 (<0.1–0.1)	20.7 (14.2–28.2)	20.7 (14.2–28.2)
Serbia	28.7 (21.9–35.4)	13.1 (9.0–17.8)	1.9 (0.5–4.8)	2.9 (0.9–6.9)	13.0 (8.2–19.2)	12.9 (8.2–18.9)	1.0 (0.4–1.6)	1.0 (0.4–1.6)	9.7 (5.9–14.0)	9.9 (6.0–14.2)	11.3 (5.2–17.0)	1.0 (0.0–2.3)	<0.1 (<0.1–0.1)	<0.1 (<0.1–0.1)	21.9 (14.8–28.9)	21.9 (14.8–28.9)
Slovakia	23.3 (16.7–29.9)	4.5 (2.7–6.8)	0.1 (<0.1–0.2)	0.1 (<0.1–0.3)	9.4 (5.5–14.5)	9.4 (5.5–14.4)	0.9 (0.3–1.4)	0.9 (0.3–1.4)	7.4 (4.3–10.8)	8.7 (5.3–12.4)	16.5 (9.4–22.8)	1.1 (0.0–3.6)	<0.1 (<0.1–0.1)	<0.1 (<0.1–0.1)	20.2 (13.2–29.4)	20.2 (13.2–29.4)
Slovenia	16.7 (12.5–21.5)	7.0 (4.8–9.7)	0.5 (0.1–1.5)	0.8 (0.2–2.2)	8.6 (5.3–13.3)	8.6 (5.3–13.2)	0.8 (0.3–1.4)	0.8 (0.3–1.4)	7.8 (5.0–11.3)	8.0 (5.0–11.5)	9.5 (0.8–19.2)	1.0 (0.0–3.9)	<0.1 (<0.1–0.1)	<0.1 (<0.1–0.1)	22.1 (15.2–30.1)	22.1 (15.2–30.1)
Eastern Europe	33.7 (26.9–40.4)	3.0 (2.0–4.3)	0.2 (<0.1–0.5)	0.3 (0.1–0.7)	5.6 (2.9–9.7)	5.7 (3.0–9.7)	2.3 (0.9–3.8)	2.3 (0.9–3.8)	4.8 (2.9–6.8)	7.8 (4.8–11.1)	10.1 (4.8–15.0)	1.8 (0.2–3.7)	<0.1 (<0.1–0.3)	<0.1 (<0.1–0.3)	16.8 (1.2–30.1)	16.8 (1.8–29.8)
Belarus	33.4 (26.2–40.9)	1.5 (0.9–2.3)	0.1 (<0.1–0.2)	0.1 (<0.1–0.3)	8.2 (4.7–13.0)	8.2 (4.7–13.0)	1.9 (0.8–3.1)	1.9 (0.8–3.1)	3.9 (2.3–5.8)	8.1 (4.9–11.6)	14.8 (7.7–21.3)	2.0 (0.3–4.0)	<0.1 (<0.1–0.2)	<0.1 (<0.1–0.2)	18.3 (9.6–30.8)	18.3 (9.6–30.8)
Estonia	25.4 (19.1–31.6)	7.2 (4.4–10.2)	0.4 (<0.1–1.2)	0.6 (0.1–1.9)	1.7 (0.5–3.5)	1.9 (0.7–3.7)	1.8 (0.7–3.0)	1.8 (0.7–3.0)	7.4 (4.6–10.7)	5.2 (3.0–7.8)	15.4 (8.0–22.0)	2.6 (0.0–5.7)	0.1 (<0.1–0.5)	0.1 (<0.1–0.5)	15.8 (3.4–32.6)	15.8 (3.4–32.6)
Latvia	25.0 (18.0–32.0)	4.0 (2.3–5.9)	0.4 (0.1–1.1)	0.6 (0.1–1.7)	5.6 (3.1–9.1)	5.6 (3.1–9.1)	1.9 (0.8–3.1)	1.9 (0.8–3.1)	5.3 (2.9–8.0)	7.0 (4.1–10.3)	12.8 (5.7–18.7)	1.5 (0.0–4.3)	<0.1 (<0.1–0.3)	<0.1 (<0.1–0.3)	17.2 (7.2–31.3)	17.2 (7.2–31.3)
Lithuania	25.7 (19.2–32.8)	2.5 (1.5–3.9)	0.2 (<0.1–0.5)	0.3 (<0.1–0.7)	4.5 (2.3–7.6)	4.5 (2.3–7.6)	1.9 (0.8–3.2)	1.9 (0.8–3.2)	4.5 (2.6–6.7)	5.9 (3.4–8.7)	15.4 (8.3–22.0)	2.6 (0.0–5.9)	<0.1 (<0.1–0.2)	<0.1 (<0.1–0.2)	19.1 (9.2–31.0)	19.1 (9.2–31.0)
Republic of Moldova	25.7 (19.9–31.8)	1.7 (1.0–2.6)	0.7 (0.2–1.6)	1.1 (0.4–2.4)	6.7 (3.3–11.9)	6.7 (3.4–11.9)	4.6 (1.9–7.4)	4.6 (1.9–7.4)	4.7 (3.0–6.7)	7.5 (4.8–10.6)	10.1 (3.6–16.3)	1.9 (0.0–4.8)	<0.1 (<0.1–0.1)	<0.1 (<0.1–0.1)	22.1 (13.3–30.8)	22.1 (13.3–30.8)
Russian Federation	35.0 (28.1–41.7)	3.4 (2.2–4.8)	0.1 (<0.1–0.4)	0.2 (<0.1–0.6)	5.2 (2.6–9.2)	5.3 (2.6–9.2)	2.1 (0.9–3.5)	2.1 (0.9–3.4)	4.8 (2.8–6.9)	7.8 (4.8–11.1)	10.4 (4.9–15.4)	2.0 (0.3–4.1)	<0.1 (<0.1–0.4)	<0.1 (<0.1–0.4)	15.9 (<0.1–31.8)	15.8 (<0.1–31.8)
Ukraine	30.6 (23.5–37.8)	1.7 (1.0–2.8)	0.4 (0.1–0.9)	0.5 (0.2–1.3)	7.1 (3.7–12.3)	7.1 (3.7–12.3)	3.1 (1.2–5.0)	3.1 (1.2–5.0)	5.0 (3.0–7.3)	8.3 (5.0–11.8)	6.6 (1.5–12.0)	0.9 (0.0–2.4)	<0.1 (<0.1–0.2)	<0.1 (<0.1–0.2)	19.7 (13.3–26.2)	19.7 (13.3–26.2)
**High-income**	**19.8 (15.1–24.7)**	**8.2 (5.8–10.9)**	**<0.1 (<0.1–0.1)**	**0.1 (<0.1–0.2)**	**5.5 (3.0–8.9)**	**5.3 (2.9–8.6)**	**1.5 (0.6–2.4)**	**1.4 (0.6–2.3)**	**4.4 (2.6–6.4)**	**4.6 (2.7–6.7)**	**9.1 (3.8–13.7)**	**3.9 (1.1–6.9)**	**0.4 (0.1–0.7)**	**0.4 (0.2–0.7)**	**17.9 (13.7–22.7)**	**18.2 (13.7–23.4)**

	Smoking		Household air pollution		Ambient particulate matter		Handwashing		Secondhand smoke		Alcohol use		High temperature		Low temperature	
	M	F	M	F	M	F	M	F	M	F	M	F	M	F	M	F
Australasia	8.0 (5.7–10.7)	7.3 (4.9–10.1)	<0.1 (<0.1–<0.1)	<0.1 (<0.1–0.1)	1.9 (0.4–4.1)	1.9 (0.4–4.1)	1.9 (0.8–3.2)	1.9 (0.8–3.2)	3.6 (2.0–5.5)	2.7 (1.5–4.1)	12.1 (5.1–18.2)	4.3 (0.4–8.8)	6.0 (4.0–7.9)	6.0 (3.9–7.9)	8.5 (5.4–11.4)	8.5 (5.5–11.4)
Australia	7.4 (5.3–10.0)	7.3 (4.9–10.2)	<0.1 (<0.1–<0.1)	<0.1 (<0.1–0.1)	1.9 (0.5–4.3)	1.9 (0.5–4.3)	1.9 (0.8–3.2)	1.9 (0.8–3.2)	3.7 (2.0–5.6)	2.8 (1.5–4.2)	12.4 (5.3–18.6)	4.3 (0.2–9.2)	7.3 (4.8–9.7)	7.3 (4.8–9.7)	5.9 (2.7–8.9)	5.9 (2.7–8.9)
New Zealand	10.7 (7.1–14.8)	7.4 (4.8–10.3)	<0.1 (<0.1–<0.1)	<0.1 (<0.1–<0.1)	1.5 (0.2–3.5)	1.5 (0.2–3.5)	1.9 (0.8–3.2)	1.9 (0.8–3.2)	3.5 (1.9–5.3)	2.3 (1.2–3.6)	10.8 (4.3–16.5)	4.4 (0.3–8.4)	<0.1 (<0.1–<0.1)	<0.1 (<0.1–<0.1)	20.6 (13.5–28.1)	20.6 (13.5–28.1)
High-income Asia Pacific	23.4 (17.8–29.1)	4.3 (2.8–6.0)	<0.1 (<0.1–<0.1)	<0.1 (<0.1–0.1)	7.1 (3.9–11.5)	7.4 (4.1–11.9)	1.9 (0.7–3.1)	1.9 (0.7–3.1)	4.2 (2.4–6.3)	6.1 (3.6–8.8)	6.0 (1.2–11.2)	2.6 (0.3–5.6)	0.4 (<0.1–1.0)	0.4 (<0.1–1.1)	16.3 (13.1–19.8)	16.3 (13.1–19.8)
Brunei Darussalam	24.2 (18.0–31.0)	7.0 (4.4–10.2)	<0.1 (<0.1–0.1)	0.1 (<0.1–0.2)	2.6 (0.8–5.4)	2.6 (0.8–5.4)	2.0 (0.8–3.2)	2.0 (0.8–3.2)	3.6 (2.1–5.4)	4.7 (2.7–7.0)	0.1 (0.0–0.7)	<0.1 (0.0–0.2)	0.9 (<0.1–5.8)	0.9 (<0.1–5.8)	<0.1 (<0.1–0.5)	<0.1 (<0.1–0.5)
Japan	23.0 (17.6–28.6)	4.1 (2.6–5.8)	<0.1 (<0.1–<0.1)	<0.1 (<0.1–0.1)	6.3 (3.4–10.4)	6.3 (3.3–10.4)	1.9 (0.7–3.1)	1.9 (0.7–3.1)	4.3 (2.5–6.5)	6.2 (3.6–9.0)	5.6 (0.7–10.8)	2.3 (0.0–5.4)	0.4 (<0.1–1.0)	0.5 (<0.1–1.0)	16.3 (13.1–19.7)	16.2 (13.1–19.7)
Republic of Korea	29.1 (22.1–35.8)	5.7 (3.5–8.7)	<0.1 (<0.1–<0.1)	<0.1 (<0.1–<0.1)	14.2 (8.6–21.1)	14.2 (8.6–21.1)	1.9 (0.7–3.1)	1.9 (0.7–3.1)	3.5 (2.0–5.2)	6.0 (3.4–8.6)	11.5 (5.3–17.5)	4.9 (1.5–9.3)	0.2 (<0.1–0.9)	0.2 (<0.1–0.9)	20.4 (14.7–25.9)	20.4 (14.7–25.9)
Singapore	14.4 (10.3–19.0)	3.3 (1.8–5.4)	<0.1 (<0.1–<0.1)	<0.1 (<0.1–0.1)	9.5 (5.0–16.2)	9.5 (5.0–16.2)	1.9 (0.8–3.1)	1.9 (0.8–3.1)	2.8 (1.6–4.3)	3.2 (1.7–4.8)	0.9 (0.0–2.8)	0.2 (0.0–0.9)	1.3 (<0.1–11.7)	1.3 (<0.1–11.7)	0.1 (<0.1–1.2)	0.1 (<0.1–1.2)
High-income North America	17.0 (12.8–21.4)	11.6 (8.4–15.0)	<0.1 (<0.1–0.1)	<0.1 (<0.1–<0.1)	2.5 (1.1–4.9)	2.5 (1.1–4.9)	1.0 (0.4–1.6)	1.0 (0.4–1.6)	4.3 (2.7–6.2)	3.4 (2.1–4.8)	7.7 (3.1–11.8)	4.0 (1.3–7.0)	0.5 (0.2–0.9)	0.5 (0.2–0.8)	16.3 (11.8–21.2)	16.4 (11.9–21.3)
Canada	16.1 (12.0–20.6)	10.5 (7.4–14.3)	<0.1 (<0.1–<0.1)	<0.1 (<0.1–<0.1)	2.2 (0.9–4.4)	2.2 (0.9–4.4)	1.0 (0.4–1.7)	1.0 (0.4–1.7)	4.3 (2.7–6.5)	3.3 (1.9–5.0)	8.1 (3.2–12.8)	3.8 (0.7–7.4)	<0.1 (<0.1–0.3)	<0.1 (<0.1–0.3)	20.8 (0.8–39.3)	20.8 (0.8–39.3)
United States of America	17.1 (12.9–21.6)	11.8 (8.5–15.2)	<0.1 (<0.1–<0.1)	<0.1 (<0.1–<0.1)	2.6 (1.1–4.9)	2.6 (1.1–4.9)	1.0 (0.4–1.6)	1.0 (0.4–1.6)	4.3 (2.6–6.3)	3.4 (2.1–4.8)	7.6 (3.2–11.9)	4.0 (1.3–7.2)	0.6 (0.3–1.0)	0.6 (0.3–0.9)	15.7 (12.1–19.7)	15.8 (12.1–19.7)
Southern Latin America	14.2 (10.1–18.8)	10.1 (7.0–13.4)	0.3 (0.1–0.8)	0.5 (0.2–1.1)	7.0 (3.8–11.7)	7.0 (3.8–11.6)	2.4 (1.0–4.0)	2.4 (1.0–4.0)	7.8 (4.5–11.5)	7.9 (4.4–11.5)	11.3 (4.7–17.6)	3.8 (0.7–7.2)	0.8 (0.5–1.2)	0.8 (0.5–1.2)	16.2 (10.3–22.7)	16.1 (10.2–22.6)
Argentina	15.2 (10.9–20.0)	11.1 (7.7–14.8)	0.3 (0.1–0.8)	0.4 (0.1–1.1)	6.5 (3.4–11.1)	6.5 (3.4–11.0)	2.5 (1.0–4.1)	2.5 (1.0–4.1)	7.7 (4.4–11.4)	7.8 (4.3–11.5)	11.5 (4.5–18.0)	3.7 (0.6–7.2)	0.9 (0.5–1.4)	0.9 (0.5–1.4)	16.1 (10.5–22.4)	16.1 (10.5–22.4)
Chile	6.7 (4.2–9.7)	4.7 (3.0–7.0)	0.5 (0.1–1.3)	0.8 (0.2–2.1)	11.7 (7.1–18.0)	11.7 (7.1–17.9)	2.0 (0.8–3.4)	2.0 (0.8–3.4)	8.2 (4.2–12.5)	7.7 (3.9–11.9)	10.4 (4.4–16.5)	4.1 (0.9–7.9)	<0.1 (<0.1–<0.1)	<0.1 (<0.1–<0.1)	19.3 (10.4–29.4)	19.3 (10.4–29.4)
Uruguay	13.6 (9.9–17.7)	5.5 (3.5–7.7)	0.2 (0.1–0.6)	0.4 (0.1–0.9)	4.0 (1.7–7.6)	4.0 (1.8–7.6)	2.3 (0.9–3.7)	2.3 (0.9–3.7)	9.0 (5.3–13.1)	9.2 (5.4–13.0)	10.1 (4.1–16.0)	4.3 (1.1–8.2)	0.1 (<0.1–0.3)	0.1 (<0.1–0.3)	8.0 (4.5–11.6)	8.0 (4.5–11.6)
Western Europe	18.9 (14.5–23.9)	8.9 (6.1–12.0)	<0.1 (<0.1–0.1)	<0.1 (<0.1–0.1)	4.9 (2.7–8.1)	4.9 (2.7–8.1)	1.0 (0.4–1.7)	1.0 (0.4–1.7)	4.0 (2.3–5.9)	3.6 (2.1–5.2)	12.6 (6.4–18.0)	4.8 (1.5–8.2)	0.1 (<0.1–0.2)	0.1 (<0.1–0.2)	21.2 (15.0–27.8)	21.3 (15.1–28.1)
Andorra	19.1 (13.4–24.8)	6.5 (3.7–10.2)	<0.1 (<0.1–<0.1)	<0.1 (<0.1–<0.1)	3.5 (1.7–6.3)	3.5 (1.7–6.3)	1.0 (0.4–1.6)	1.0 (0.4–1.6)	4.6 (2.6–6.7)	4.3 (2.5–6.3)	12.4 (5.2–18.4)	4.4 (0.5–8.5)	<0.1 (<0.1–<0.1)	<0.1 (<0.1–<0.1)	17.0 (1.9–35.8)	17.0 (1.9–35.8)
Austria	22.4 (17.1–28.1)	9.2 (6.3–12.6)	<0.1 (<0.1–0.1)	<0.1 (<0.1–0.1)	5.6 (3.1–9.1)	5.6 (3.1–9.1)	1.0 (0.4–1.6)	1.0 (0.4–1.6)	7.5 (4.7–10.6)	5.3 (3.3–7.7)	14.1 (7.0–20.5)	4.6 (0.6–9.0)	<0.1 (<0.1–0.1)	<0.1 (<0.1–0.1)	19.7 (10.6–31.5)	19.7 (10.6–31.5)

	Smoking		Household air pollution		Ambient particulate matter		Handwashing		Secondhand smoke		Alcohol use		High temperature		Low temperature	
	M	F	M	F	M	F	M	F	M	F	M	F	M	F	M	F
Belgium	22.6 (17.4–27.9)	9.5 (6.8–12.5)	<0.1 (<0.1–<0.1)	<0.1 (<0.1–<0.1)	5.9 (3.4–9.7)	5.9 (3.4–9.7)	1.0 (0.4–1.6)	1.0 (0.4–1.6)	3.5 (2.0–4.9)	4.6 (2.9–6.7)	13.1 (6.2–19.6)	7.1 (2.2–11.8)	<0.1 (<0.1–0.1)	<0.1 (<0.1–0.1)	23.4 (14.7–32.7)	23.4 (14.7–32.7)
Cyprus	28.6 (22.3–35.6)	7.0 (4.4–10.1)	<0.1 (<0.1–<0.1)	<0.1 (<0.1–0.1)	7.7 (4.5–12.5)	7.7 (4.5–12.5)	1.0 (0.4–1.6)	1.0 (0.4–1.6)	4.2 (2.5–6.3)	8.2 (4.9–11.5)	13.1 (5.7–19.4)	1.9 (0.0–4.3)	2.1 (0.9–3.6)	2.1 (0.9–3.6)	4.5 (<0.1–10.9)	4.5 (<0.1–10.9)
Denmark	28.3 (22.4–34.4)	18.2 (13.1–23.5)	<0.1 (<0.1–<0.1)	<0.1 (<0.1–<0.1)	4.0 (2.1–7.2)	4.0 (2.1–7.2)	1.0 (0.4–1.6)	1.0 (0.4–1.6)	5.2 (3.1–7.5)	3.3 (2.0–4.8)	11.4 (4.7–17.9)	6.3 (0.8–11.7)	<0.1 (<0.1–<0.1)	<0.1 (<0.1–<0.1)	23.2 (13.0–34.4)	23.2 (13.0–34.4)
Finland	15.1 (11.2–19.5)	6.2 (4.1–8.8)	<0.1 (<0.1–<0.1)	<0.1 (<0.1–<0.1)	1.2 (0.2–2.9)	1.2 (0.2–2.9)	1.0 (0.4–1.7)	1.0 (0.4–1.7)	3.2 (1.8–4.8)	2.5 (1.4–3.8)	7.4 (2.2–13.0)	2.2 (0.0–5.4)	<0.1 (<0.1–0.3)	<0.1 (<0.1–0.3)	17.9 (3.8–36.4)	17.9 (3.8–36.4)
France	16.3 (12.0–21.3)	6.5 (4.0–9.4)	<0.1 (<0.1–<0.1)	<0.1 (<0.1–<0.1)	5.1 (2.8–8.5)	5.1 (2.8–8.5)	1.0 (0.4–1.7)	1.0 (0.4–1.7)	2.6 (1.3–4.1)	2.2 (1.2–3.3)	15.7 (7.5–22.9)	6.5 (1.6–11.6)	<0.1 (<0.1–0.1)	<0.1 (<0.1–0.1)	21.7 (14.9–28.4)	21.7 (14.9–28.4)
Germany	19.5 (14.5–24.7)	9.1 (6.2–12.3)	<0.1 (<0.1–<0.1)	<0.1 (<0.1–<0.1)	5.3 (3.0–8.8)	5.3 (3.0–8.8)	1.0 (0.4–1.6)	1.0 (0.4–1.6)	3.0 (1.7–4.4)	2.4 (1.4–3.6)	17.4 (10.0–24.2)	9.3 (4.1–14.6)	<0.1 (<0.1–0.2)	<0.1 (<0.1–0.2)	22.5 (14.3–31.1)	22.5 (14.3–31.1)
Greece	29.2 (22.9–35.9)	9.5 (6.2–13.0)	<0.1 (<0.1–0.1)	0.1 (<0.1–0.2)	6.9 (4.0–10.9)	6.9 (4.0–10.9)	1.0 (0.4–1.7)	1.0 (0.4–1.7)	6.2 (3.6–9.0)	7.1 (4.3–10.2)	12.4 (6.0–18.6)	0.9 (0.0–2.8)	0.1 (<0.1–0.3)	0.1 (<0.1–0.3)	15.7 (12.3–19.4)	15.7 (12.3–19.4)
Iceland	17.8 (13.3–22.6)	9.8 (6.7–13.6)	<0.1 (<0.1–<0.1)	<0.1 (<0.1–<0.1)	1.2 (0.2–3.0)	1.2 (0.2–3.0)	1.0 (0.4–1.7)	1.0 (0.4–1.7)	5.5 (3.5–8.2)	4.4 (2.8–6.4)	8.8 (2.9–14.7)	2.9 (0.0–6.8)	<0.1 (0.0–0.0)	<0.1 (0.0–0.0)	21.5 (5.1–38.7)	21.5 (5.1–38.7)
Ireland	20.9 (15.7–26.4)	13.0 (8.8–17.6)	<0.1 (<0.1–0.1)	<0.1 (<0.1–<0.1)	2.7 (1.2–5.1)	2.7 (1.2–5.1)	1.0 (0.4–1.6)	1.0 (0.4–1.6)	4.2 (2.3–6.4)	3.7 (2.1–5.5)	11.8 (5.9–17.8)	4.5 (1.1–8.5)	<0.1 (0.0–0.0)	<0.1 (0.0–0.0)	25.7 (14.4–37.2)	25.7 (14.4–37.2)
Israel	18.8 (14.3–23.6)	7.8 (5.3–10.9)	<0.1 (<0.1–<0.1)	<0.1 (<0.1–0.1)	10.1 (6.1–15.3)	10.1 (6.1–15.3)	1.0 (0.4–1.7)	1.0 (0.4–1.7)	4.0 (2.2–5.9)	3.4 (1.9–5.1)	1.6 (0.0–4.4)	0.3 (0.0–1.3)	2.1 (0.8–3.9)	2.1 (0.8–3.9)	3.5 (<0.1–10.4)	3.5 (<0.1–10.4)
Italy	19.5 (14.6–24.5)	7.4 (4.8–10.5)	<0.1 (<0.1–0.1)	<0.1 (<0.1–0.1)	8.2 (4.9–12.5)	8.2 (4.9–12.5)	1.0 (0.4–1.7)	1.0 (0.4–1.7)	6.2 (3.7–9.0)	4.0 (2.4–5.9)	12.2 (6.2–18.0)	4.4 (1.7–7.6)	0.1 (<0.1–0.2)	0.1 (<0.1–0.2)	17.9 (13.2–22.5)	17.9 (13.2–22.5)
Luxembourg	20.6 (15.0–26.4)	10.3 (6.8–14.6)	<0.1 (<0.1–<0.1)	<0.1 (<0.1–<0.1)	4.2 (2.2–7.3)	4.2 (2.2–7.3)	1.0 (0.4–1.6)	1.0 (0.4–1.6)	3.8 (2.2–5.7)	5.5 (3.4–7.9)	15.8 (8.2–22.6)	5.1 (0.6–9.8)	<0.1 (<0.1–0.1)	<0.1 (<0.1–0.1)	23.1 (13.3–33.6)	23.1 (13.3–33.6)
Malta	17.0 (12.8–21.7)	6.8 (4.6–9.6)	<0.1 (<0.1–0.1)	<0.1 (<0.1–0.1)	6.1 (3.4–9.9)	6.1 (3.4–9.9)	1.0 (0.4–1.7)	1.0 (0.4–1.7)	4.8 (2.9–7.1)	4.5 (2.8–6.5)	9.1 (3.5–14.3)	2.7 (0.2–5.5)	0.3 (<0.1–0.9)	0.3 (<0.1–0.9)	3.6 (<0.1–15.0)	3.6 (<0.1–15.0)
Monaco	19.0 (13.1–24.8)	7.6 (4.4–11.5)	<0.1 (<0.1–<0.1)	<0.1 (<0.1–<0.1)	5.3 (2.5–9.2)	5.3 (2.5–9.2)	1.0 (0.4–1.6)	1.0 (0.4–1.6)	4.8 (2.9–7.0)	4.3 (2.6–6.3)	6.7 (0.0–18.0)	2.3 (0.0–7.7)	<0.1 (<0.1–0.1)	<0.1 (<0.1–0.1)	15.4 (11.9–19.0)	15.4 (11.9–19.0)
Netherlands	21.0 (15.5–26.6)	12.1 (7.8–16.7)	<0.1 (<0.1–<0.1)	<0.1 (<0.1–<0.1)	5.5 (3.1–9.0)	5.5 (3.1–9.0)	1.0 (0.4–1.6)	1.0 (0.4–1.6)	4.1 (2.4–6.1)	3.3 (1.9–4.9)	11.5 (4.6–17.3)	5.3 (0.8–9.8)	<0.1 (<0.1–0.1)	<0.1 (<0.1–0.1)	23.6 (13.4–33.5)	23.6 (13.4–33.5)
Norway	10.3 (7.4–13.6)	5.2 (3.2–7.9)	<0.1 (<0.1–<0.1)	<0.1 (<0.1–<0.1)	1.7 (0.6–3.8)	1.8 (0.6–3.8)	1.0 (0.4–1.6)	1.0 (0.4–1.6)	4.0 (2.3–5.9)	3.2 (1.8–4.6)	7.4 (1.7–13.0)	2.6 (0.0–6.2)	<0.1 (<0.1–0.1)	<0.1 (<0.1–0.1)	19.1 (5.7–36.0)	19.1 (5.8–36.0)
Portugal	14.3 (10.2–19.0)	1.7 (1.0–2.7)	<0.1 (<0.1–0.1)	0.1 (<0.1–0.2)	3.0 (1.4–5.5)	3.0 (1.4–5.5)	1.1 (0.4–1.8)	1.1 (0.4–1.8)	4.2 (2.3–6.3)	5.7 (3.1–8.5)	15.0 (7.8–21.1)	3.2 (1.1–5.7)	0.2 (<0.1–0.3)	0.2 (<0.1–0.3)	15.3 (11.7–19.2)	15.3 (11.7–19.2)
San Marino	17.4 (12.5–22.9)	6.7 (4.1–10.0)	<0.1 (<0.1–<0.1)	<0.1 (<0.1–<0.1)	4.1 (1.5–7.9)	4.1 (1.5–7.9)	1.0 (0.4–1.7)	1.0 (0.4–1.7)	5.2 (3.2–7.8)	4.6 (2.8–6.7)	11.7 (0.0–20.7)	4.5 (0.0–10.2)	0.1 (<0.1–0.2)	0.1 (<0.1–0.2)	15.7 (12.5–19.0)	15.7 (12.5–19.0)

	Smoking		Household air pollution		Ambient particulate matter		Handwashing		Secondhand smoke		Alcohol use		High temperature		Low temperature	
	M	F	M	F	M	F	M	F	M	F	M	F	M	F	M	F
Spain	20.8 (15.5–26.8)	3.0 (1.7–4.5)	0.1 (<0.1–0.2)	0.1 (<0.1–0.3)	4.0 (2.1–6.9)	4.0 (2.1–6.9)	1.0 (0.4–1.7)	1.0 (0.4–1.7)	4.8 (2.7–7.2)	4.6 (2.8–6.7)	10.3 (4.5–16.1)	2.9 (0.8–5.3)	0.2 (<0.1–0.5)	0.2 (<0.1–0.5)	17.3 (13.3–21.5)	17.3 (13.3–21.5)
Sweden	17.9 (13.4–22.8)	12.8 (9.1–16.7)	<0.1 (<0.1–<0.1)	<0.1 (<0.1–<0.1)	1.2 (0.3–3.0)	1.2 (0.3–3.0)	1.0 (0.4–1.7)	1.0 (0.4–1.7)	4.5 (2.7–6.5)	1.9 (1.2–2.8)	10.4 (4.2–16.4)	7.4 (2.2–12.7)	<0.1 (<0.1–0.1)	<0.1 (<0.1–0.1)	18.5 (5.2–33.0)	18.5 (5.2–33.1)
Switzerland	21.4 (16.1–27.3)	9.6 (6.3–13.3)	<0.1 (<0.1–<0.1)	<0.1 (<0.1–<0.1)	4.1 (2.2–7.0)	4.1 (2.2–7.0)	1.0 (0.4–1.6)	1.0 (0.4–1.6)	4.1 (2.3–6.1)	3.6 (2.1–5.4)	13.3 (5.8–20.0)	7.6 (1.9–13.1)	<0.1 (<0.1–<0.1)	<0.1 (<0.1–<0.1)	21.1 (11.3–32.9)	21.1 (11.3–32.9)
United Kingdom	17.1 (12.8–22.1)	12.3 (8.3–16.6)	<0.1 (<0.1–<0.1)	<0.1 (<0.1–<0.1)	4.1 (2.1–7.1)	4.1 (2.1–7.1)	1.0 (0.4–1.7)	1.0 (0.4–1.7)	4.1 (2.3–6.1)	3.6 (2.0–5.3)	9.4 (3.5–15.6)	2.9 (0.0–6.7)	<0.1 (<0.1–<0.1)	<0.1 (<0.1–<0.1)	24.9 (15.8–35.0)	24.9 (15.7–34.9)
**Latin America and Caribbean**	**14.8 (10.7–19.2)**	**6.1 (4.0–8.3)**	**2.9 (1.3–5.2)**	**3.8 (2.0–6.3)**	**8.6 (5.4–13.0)**	**8.3 (5.3–12.4)**	**6.6 (2.7–10.5)**	**6.6 (2.7–10.4)**	**4.8 (2.7–7.3)**	**5.0 (2.8–7.3)**	**4.2 (1.6–7.0)**	**0.7 (0.0–1.7)**	**1.4 (<0.1–4.1)**	**1.4 (<0.1–4.4)**	**3.1 (1.4–4.9)**	**3.0 (1.2–4.8)**
Andean Latin America	7.5 (4.9–10.4)	1.3 (0.6–2.1)	3.3 (1.3–6.4)	5.0 (2.3–9.0)	13.9 (8.6–21.1)	13.5 (8.5–20.2)	7.8 (3.2–12.4)	7.8 (3.2–12.4)	1.9 (0.7–3.5)	1.8 (0.7–3.4)	2.0 (0.0–4.8)	0.3 (0.0–1.4)	0.4 (<0.1–1.3)	0.5 (<0.1–1.3)	9.4 (5.8–13.3)	9.4 (5.8–13.3)
Bolivia (Plurinational State of)	10.7 (7.2–14.7)	1.4 (0.5–2.5)	6.0 (2.5–11.7)	8.7 (4.1–15.1)	12.9 (7.4–20.6)	12.3 (7.2–19.3)	8.4 (3.5–13.2)	8.4 (3.5–13.2)	1.5 (0.5–2.9)	1.5 (0.5–2.9)	1.9 (0.0–5.2)	0.3 (0.0–1.4)	1.5 (0.5–2.7)	1.5 (0.5–2.7)	9.1 (5.9–12.6)	9.1 (5.9–12.6)
Ecuador	10.9 (6.9–15.3)	2.0 (1.0–3.3)	0.9 (0.3–2.1)	1.4 (0.5–3.2)	10.2 (5.4–16.6)	10.2 (5.5–16.4)	6.9 (2.8–11.0)	6.9 (2.8–11.0)	1.8 (0.6–3.5)	1.7 (0.6–3.2)	1.7 (0.0–3.6)	0.2 (0.0–0.9)	0.3 (<0.1–1.2)	0.3 (<0.1–1.2)	5.8 (3.6–8.1)	5.8 (3.6–8.1)
Peru	5.8 (3.6–8.2)	1.1 (0.5–1.8)	3.3 (1.3–6.5)	5.0 (2.2–8.9)	15.2 (9.3–23.1)	14.8 (9.3–22.2)	7.9 (3.3–12.5)	7.9 (3.3–12.5)	2.0 (0.7–3.7)	1.9 (0.7–3.6)	2.1 (0.0–5.1)	0.4 (0.0–1.6)	0.2 (<0.1–0.9)	0.2 (<0.1–0.9)	10.4 (6.0–15.2)	10.4 (6.0–15.2)
Caribbean	18.9 (13.8–24.2)	7.0 (4.6–9.9)	5.0 (2.5–8.2)	6.6 (3.7–10.1)	7.9 (3.6–14.6)	7.6 (3.6–13.9)	9.2 (4.0–14.5)	9.5 (4.1–14.8)	5.3 (3.1–7.8)	5.2 (3.0–7.5)	4.6 (1.6–7.5)	0.7 (<0.1–1.5)	1.2 (<0.1–3.3)	1.2 (<0.1–3.3)	0.1 (<0.1–1.4)	0.1 (<0.1–1.4)
Antigua and Barbuda	10.2 (7.0–13.9)	3.3 (2.0–5.0)	0.1 (<0.1–0.3)	0.2 (<0.1–0.4)	8.7 (2.7–18.4)	8.7 (2.7–18.4)	7.5 (3.1–11.9)	7.5 (3.1–11.9)	4.8 (2.7–7.0)	4.0 (2.3–6.0)	4.7 (1.4–8.1)	0.8 (0.0–1.7)	0.4 (<0.1–2.3)	0.4 (<0.1–2.3)	0.3 (<0.1–1.7)	0.3 (<0.1–1.7)
Bahamas	9.7 (6.6–13.1)	2.8 (1.6–4.2)	0.1 (<0.1–0.4)	0.2 (<0.1–0.5)	7.6 (1.7–16.5)	7.6 (1.7–16.4)	6.3 (2.6–10.2)	6.3 (2.6–10.2)	5.0 (2.9–7.3)	4.8 (2.8–7.0)	3.7 (0.0–8.0)	0.6 (0.0–1.8)	1.6 (<0.1–4.5)	1.6 (<0.1–4.5)	0.3 (<0.1–2.0)	0.3 (<0.1–2.0)
Barbados	13.1 (9.1–17.5)	0.9 (0.5–1.3)	<0.1 (<0.1–<0.1)	<0.1 (<0.1–<0.1)	10.8 (3.8–21.4)	10.8 (3.8–21.4)	7.1 (2.9–11.4)	7.1 (2.9–11.4)	2.8 (1.5–4.2)	2.7 (1.5–4.0)	8.1 (4.1–12.1)	0.8 (0.0–2.1)	1.2 (<0.1–3.9)	1.2 (<0.1–3.9)	0.4 (<0.1–2.0)	0.4 (<0.1–2.0)
Belize	15.0 (10.1–20.1)	2.5 (1.4–3.9)	2.7 (1.0–5.7)	4.1 (1.7–7.9)	10.6 (3.3–20.9)	10.4 (3.6–20.5)	9.5 (4.0–15.0)	9.5 (4.0–15.0)	3.9 (2.1–6.0)	4.8 (2.6–7.2)	4.8 (2.0–7.8)	0.3 (<0.1–0.7)	1.7 (<0.1–4.2)	1.7 (<0.1–4.2)	0.1 (<0.1–1.3)	0.1 (<0.1–1.3)
Cuba	23.8 (17.7–30.4)	8.3 (5.2–12.4)	0.2 (0.1–0.6)	0.4 (0.1–0.9)	8.8 (3.7–16.9)	8.8 (3.7–16.9)	6.0 (2.5–9.6)	6.0 (2.5–9.6)	6.4 (3.9–9.3)	6.5 (3.8–9.5)	4.3 (1.2–7.5)	0.7 (0.0–1.6)	1.5 (<0.1–4.0)	1.5 (<0.1–4.0)	0.2 (<0.1–1.6)	0.2 (<0.1–1.6)
Dominica	9.3 (6.0–12.7)	2.3 (1.3–3.6)	1.0 (0.3–2.3)	1.5 (0.4–3.4)	9.4 (3.2–19.0)	9.4 (3.3–18.9)	8.3 (3.5–13.2)	8.3 (3.5–13.2)	3.7 (1.9–5.7)	3.5 (1.8–5.3)	5.9 (2.2–9.6)	1.1 (0.1–2.3)	1.1 (<0.1–3.5)	1.1 (<0.1–3.5)	0.3 (<0.1–3.2)	0.3 (<0.1–3.2)
Dominican Republic	21.7 (15.8–28.0)	14.4 (9.1–19.9)	1.8 (0.6–4.0)	2.7 (1.0–5.6)	8.9 (3.3–17.9)	8.8 (3.4–17.7)	16.0 (7.1–24.3)	16.0 (7.1–24.3)	5.1 (2.9–7.5)	4.3 (2.5–6.4)	3.9 (1.3–6.6)	0.5 (0.0–1.2)	0.6 (<0.1–2.2)	0.6 (<0.1–2.2)	<0.1 (<0.1–1.7)	<0.1 (<0.1–1.7)
Grenada	10.9 (7.1–15.2)	2.3 (1.3–3.6)	0.4 (0.1–0.9)	0.6 (0.2–1.4)	10.8 (3.4–21.5)	10.8 (3.5–21.4)	7.6 (3.2–12.2)	7.6 (3.2–12.2)	3.4 (1.6–5.5)	3.4 (1.6–5.5)	7.9 (3.6–12.1)	1.5 (0.3–2.8)	1.4 (<0.1–4.2)	1.4 (<0.1–4.2)	0.3 (<0.1–1.8)	0.3 (<0.1–1.8)

	Smoking		Household air pollution		Ambient particulate matter		Handwashing		Secondhand smoke		Alcohol use		High temperature		Low temperature	
	**M**	**F**	**M**	**F**	**M**	**F**	**M**	**F**	**M**	**F**	**M**	**F**	**M**	**F**	**M**	**F**
Guyana	9.7 (6.2–13.4)	2.6 (1.3–4.2)	1.1 (0.4–2.3)	1.6 (0.6–3.3)	10.1 (3.2–20.5)	10.0 (3.2–20.3)	14.1 (6.1–21.9)	14.1 (6.1–21.9)	4.5 (2.2–6.9)	5.0 (2.5–7.7)	7.3 (3.0–11.6)	0.2 (0.0–0.9)	0.9 (<0.1–2.8)	0.9 (<0.1–2.8)	<0.1 (<0.1–1.3)	<0.1 (<0.1–1.3)
Haiti	4.5 (2.5–6.9)	1.7 (0.9–2.8)	33.4 (19.8–47.8)	41.2 (27.9–53.7)	6.3 (2.7–12.2)	5.1 (2.2–9.8)	19.5 (8.7–29.2)	19.5 (8.7–29.2)	2.3 (1.0–3.8)	2.3 (1.1–3.8)	6.1 (2.6–9.9)	1.0 (0.2–2.0)	0.8 (<0.1–3.0)	0.8 (<0.1–3.0)	<0.1 (<0.1–1.3)	<0.1 (<0.1–1.3)
Jamaica	14.0 (9.9–18.5)	4.0 (2.4–6.2)	1.3 (0.4–3.0)	2.0 (0.7–4.4)	7.7 (3.9–13.2)	7.7 (4.0–13.0)	11.2 (4.8–17.4)	11.2 (4.8–17.4)	5.0 (2.8–7.5)	5.4 (3.1–7.9)	3.0 (0.4–5.8)	0.4 (0.0–1.2)	1.2 (<0.1–4.3)	1.2 (<0.1–4.3)	0.1 (<0.1–1.1)	0.1 (<0.1–1.1)
Saint Kitts and Nevis	8.3 (5.5–11.7)	2.3 (1.3–3.4)	0.2 (<0.1–0.6)	0.4 (0.1–1.0)	3.5 (1.3–6.7)	3.5 (1.4–6.7)	7.6 (3.2–12.1)	7.6 (3.2–12.1)	4.7 (2.6–7.2)	4.4 (2.4–6.7)	2.2 (0.0–8.1)	0.4 (0.0–1.6)	1.0 (<0.1–3.9)	1.0 (<0.1–3.9)	0.4 (<0.1–2.3)	0.4 (<0.1–2.3)
Saint Lucia	11.6 (8.0–15.8)	2.8 (1.6–4.4)	0.5 (0.1–1.1)	0.7 (0.3–1.6)	10.7 (3.6–21.1)	10.7 (3.8–21.0)	5.7 (2.3–9.2)	5.7 (2.3–9.2)	3.0 (1.6–4.7)	3.0 (1.6–4.6)	7.4 (3.6–11.7)	1.4 (0.1–3.0)	1.7 (<0.1–4.8)	1.7 (<0.1–4.8)	0.3 (<0.1–1.9)	0.3 (<0.1–1.9)
Saint Vincent and the Grenadines	10.1 (6.4–14.1)	2.5 (1.4–3.9)	0.6 (0.2–1.4)	0.9 (0.3–2.0)	10.6 (3.3–21.3)	10.6 (3.4–21.1)	9.1 (3.8–14.3)	9.1 (3.8–14.3)	3.8 (2.0–6.2)	4.0 (2.1–6.2)	6.5 (2.8–10.3)	1.2 (0.3–2.5)	1.3 (<0.1–4.0)	1.3 (<0.1–4.0)	0.4 (<0.1–2.0)	0.4 (<0.1–2.0)
Suriname	19.6 (13.8–25.9)	4.8 (2.7–7.2)	1.5 (0.5–3.6)	2.3 (0.8–5.1)	10.7 (4.1–20.6)	10.6 (4.2–20.4)	9.3 (3.9–14.9)	9.3 (3.9–14.9)	6.5 (3.5–9.7)	7.7 (4.4–11.4)	4.7 (1.5–8.3)	0.8 (0.0–1.8)	1.0 (<0.1–3.0)	1.0 (<0.1–3.0)	0.3 (<0.1–1.6)	0.3 (<0.1–1.6)
Trinidad and Tobago	13.9 (9.5–18.8)	3.1 (1.7–4.9)	<0.1 (<0.1–<0.1)	<0.1 (<0.1–0.1)	11.0 (3.1–22.8)	11.0 (3.1–22.8)	6.8 (2.8–10.9)	6.8 (2.8–10.9)	4.9 (2.5–7.6)	6.0 (3.1–9.2)	5.2 (1.5–8.8)	1.2 (0.0–2.8)	0.4 (<0.1–2.6)	0.4 (<0.1–2.6)	0.2 (<0.1–1.2)	0.2 (<0.1–1.2)
Central Latin America	13.6 (9.1–18.2)	4.0 (2.4–5.7)	4.6 (2.1–8.0)	5.9 (3.2–9.6)	10.7 (6.9–15.7)	10.4 (6.8–15.3)	6.0 (2.5–9.7)	6.0 (2.5–9.7)	3.4 (1.4–5.9)	3.1 (1.3–5.4)	3.9 (1.6–6.1)	0.6 (0.0–1.3)	1.3 (<0.1–3.3)	1.2 (<0.1–3.3)	3.8 (1.8–5.8)	3.9 (2.0–6.0)
Colombia	8.1 (5.3–11.3)	4.0 (2.3–6.2)	1.7 (0.6–3.8)	2.6 (1.0–5.3)	11.2 (6.4–17.6)	11.1 (6.5–17.4)	6.1 (2.5–9.9)	6.1 (2.5–9.9)	5.0 (2.4–8.2)	4.0 (1.9–6.6)	2.5 (0.4–4.8)	0.2 (0.0–0.8)	1.0 (<0.1–3.4)	1.0 (<0.1–3.4)	3.4 (1.8–5.1)	3.4 (1.8–5.1)
Costa Rica	13.5 (9.5–17.8)	4.9 (3.0–7.3)	0.8 (0.2–1.9)	1.2 (0.4–2.8)	8.9 (5.2–13.8)	8.8 (5.2–13.7)	6.8 (2.8–10.9)	6.8 (2.8–10.9)	5.3 (2.8–7.9)	5.0 (2.8–7.7)	3.2 (0.4–6.5)	0.3 (0.0–1.2)	0.3 (<0.1–2.2)	0.3 (<0.1–2.2)	0.6 (<0.1–2.7)	0.6 (<0.1–2.7)
El Salvador	7.1 (4.9–9.7)	2.3 (1.3–3.6)	2.9 (1.2–5.7)	4.3 (2.0–7.6)	11.3 (5.9–18.3)	11.0 (6.0–17.6)	9.0 (3.8–14.4)	9.0 (3.8–14.4)	3.3 (1.7–5.3)	2.4 (1.2–3.8)	1.9 (0.1–3.9)	0.3 (0.0–1.0)	1.4 (<0.1–6.7)	1.4 (<0.1–6.7)	<0.1 (<0.1–0.6)	<0.1 (<0.1–0.6)
Guatemala	13.2 (8.4–18.8)	2.6 (1.4–4.0)	13.8 (6.3–24.3)	19.0 (10.1–30.7)	12.0 (7.5–17.9)	10.9 (6.7–16.0)	7.6 (3.2–12.1)	7.6 (3.2–12.1)	2.6 (1.0–4.6)	2.3 (0.9–4.1)	0.9 (0.0–2.3)	0.2 (0.0–0.6)	1.7 (0.5–3.5)	1.7 (0.5–3.5)	1.5 (<0.1–3.2)	1.5 (<0.1–3.2)
Honduras	18.7 (12.9–24.7)	3.3 (1.8–5.5)	17.3 (8.9–28.3)	23.3 (13.4–34.7)	9.6 (6.0–14.5)	8.5 (5.3–12.7)	5.4 (2.2–8.6)	5.4 (2.2–8.6)	5.5 (2.9–8.4)	5.5 (2.9–8.3)	3.1 (1.1–5.1)	0.3 (0.0–0.7)	0.7 (<0.1–2.6)	0.7 (<0.1–2.6)	<0.1 (<0.1–2.4)	<0.1 (<0.1–2.4)
Mexico	16.2 (10.6–21.8)	4.6 (2.5–6.7)	2.6 (1.1–4.9)	3.4 (1.6–6.2)	10.1 (6.2–15.3)	10.0 (6.3–15.0)	5.0 (2.0–8.1)	5.0 (2.0–8.0)	2.4 (0.7–4.9)	2.3 (0.6–4.5)	5.8 (2.9–8.9)	1.0 (<0.1–2.0)	1.3 (0.4–2.2)	1.2 (0.4–2.1)	6.8 (4.1–9.7)	7.3 (4.4–10.2)
Nicaragua	14.9 (10.2–20.2)	1.2 (0.7–1.9)	13.4 (5.9–23.7)	18.5 (9.8–29.4)	9.2 (5.4–14.8)	8.4 (4.9–13.5)	8.1 (3.4–12.8)	8.1 (3.4–12.8)	4.5 (2.2–7.2)	4.6 (2.2–7.3)	3.8 (1.5–6.1)	0.4 (0.0–1.1)	0.8 (<0.1–4.9)	0.8 (<0.1–4.9)	<0.1 (<0.1–1.1)	<0.1 (<0.1–1.1)
Panama	10.6 (7.3–14.1)	4.1 (2.4–6.2)	1.3 (0.4–2.7)	1.9 (0.6–4.1)	6.5 (3.2–10.9)	6.5 (3.3–10.9)	6.1 (2.5–9.8)	6.1 (2.5–9.8)	3.9 (2.1–5.8)	3.2 (1.8–4.9)	5.2 (2.2–8.5)	0.8 (0.0–1.8)	0.6 (<0.1–2.3)	0.6 (<0.1–2.3)	<0.1 (<0.1–1.0)	<0.1 (<0.1–1.0)
Venezuela (Bolivarian Republic of)	14.0 (9.3–19.4)	5.2 (3.0–7.9)	0.1 (<0.1–0.2)	0.1 (<0.1–0.2)	11.1 (5.9–18.6)	11.1 (5.9–18.6)	5.7 (2.4–9.2)	5.7 (2.4–9.2)	5.4 (2.4–8.8)	5.3 (2.4–8.6)	4.5 (1.8–7.6)	0.7 (0.0–1.7)	1.2 (<0.1–9.4)	1.2 (<0.1–9.4)	0.1 (<0.1–1.7)	0.1 (<0.1–1.7)

	**Smoking**		**Household air pollution**		**Ambient particulate matter**		**Handwashing**		**Secondhand smoke**		**Alcohol use**		**High temperature**		**Low temperature**	
	**M**	**F**	**M**	**F**	**M**	**F**	**M**	**F**	**M**	**F**	**M**	**F**	**M**	**F**	**M**	**F**
Tropical Latin America	17.3 (12.6–22.2)	8.2 (5.6–11.3)	1.4 (0.5–3.2)	2.1 (0.8–4.0)	5.8 (3.3–9.4)	5.8 (3.4–9.3)	6.0 (2.4–9.7)	5.9 (2.4–9.6)	6.6 (3.9–9.9)	6.8 (4.0–9.8)	5.1 (1.9–8.2)	0.9 (0.0–2.0)	1.8 (<0.1–6.4)	1.8 (<0.1–6.2)	1.1 (<0.1–3.2)	1.2 (<0.1–3.4)
Brazil	17.1 (12.5–22.0)	8.2 (5.5–11.2)	1.4 (0.4–3.0)	2.0 (0.7–3.8)	5.8 (3.3–9.3)	5.8 (3.4–9.3)	6.0 (2.4–9.6)	5.9 (2.4–9.5)	6.6 (3.9–9.9)	6.8 (4.0–9.8)	5.1 (1.9–8.1)	0.9 (0.0–1.9)	1.8 (<0.1–6.4)	1.7 (<0.1–6.2)	1.1 (<0.1–3.2)	1.2 (<0.1–3.4)
Paraguay	26.0 (17.7–34.2)	13.8 (8.6–20.1)	6.1 (2.2–12.2)	9.0 (3.8–16.3)	6.3 (3.5–10.6)	6.1 (3.6–10.2)	7.9 (3.3–12.7)	7.9 (3.3–12.7)	6.3 (3.5–9.4)	7.0 (4.0–10.2)	9.2 (3.8–13.9)	1.1 (0.0–2.9)	5.2 (2.9–7.6)	5.2 (2.9–7.6)	0.9 (<0.1–5.0)	0.9 (<0.1–5.0)
**North Africa and Middle East**	**24.5 (19.0–30.1)**	**3.1 (2.1–4.2)**	**2.7 (1.5–4.3)**	**3.1 (1.9–4.6)**	**18.7 (12.7–25.7)**	**18.1 (12.0–24.9)**	**6.5 (2.7–10.4)**	**6.4 (2.7–10.2)**	**9.5 (6.0–13.3)**	**11.3 (7.0–15.5)**	**0.4 (0.1–0.7)**	**0.1 (0.0–0.3)**	**4.6 (3.0–6.3)**	**4.3 (2.8–5.9)**	**9.4 (6.0–12.9)**	**9.9 (6.6–13.4)**
Afghanistan	13.8 (9.9–17.9)	1.6 (0.9–2.6)	32.5 (19.2–44.6)	38.0 (26.3–49.2)	12.7 (6.2–20.9)	9.8 (4.3–17.4)	15.7 (6.8–23.8)	15.7 (6.8–23.8)	9.4 (5.6–13.7)	9.8 (5.8–14.0)	<0.1 (0.0–0.1)	<0.1 (0.0–<0.1)	3.1 (1.9–4.2)	3.1 (1.9–4.2)	13.7 (9.0–19.6)	13.7 (9.0–19.6)
Algeria	29.6 (22.8–36.3)	2.3 (1.4–3.7)	<0.1 (<0.1–0.1)	0.1 (<0.1–0.1)	16.6 (9.1–25.5)	16.6 (9.1–25.5)	4.9 (2.0–7.9)	4.9 (2.0–7.9)	12.1 (7.9–16.8)	15.3 (10.0–20.7)	0.3 (0.0–0.8)	0.1 (0.0–0.2)	9.2 (6.2–12.2)	9.2 (6.2–12.2)	4.3 (<0.1–10.1)	4.3 (<0.1–10.1)
Bahrain	25.6 (18.9–32.4)	5.5 (3.3–8.3)	0.1 (<0.1–0.1)	0.1 (<0.1–0.2)	26.5 (17.9–36.4)	26.5 (17.9–36.4)	4.6 (1.9–7.4)	4.6 (1.9–7.4)	9.9 (6.2–14.1)	10.1 (6.5–14.1)	0.6 (0.0–1.2)	0.1 (0.0–0.3)	11.6 (5.8–16.6)	11.6 (5.8–16.6)	2.0 (<0.1–10.1)	2.0 (<0.1–10.1)
Egypt	33.1 (25.7–40.4)	1.3 (0.7–2.1)	<0.1 (<0.1–<0.1)	<0.1 (<0.1–0.1)	28.9 (19.3–40.0)	28.9 (19.3–40.0)	3.8 (1.5–6.3)	3.8 (1.5–6.3)	7.9 (4.7–11.1)	11.2 (7.0–15.9)	0.1 (0.0–0.4)	<0.1 (0.0–0.1)	7.7 (4.8–10.9)	7.7 (4.8–10.9)	2.7 (<0.1–9.1)	2.7 (<0.1–9.1)
Iran (Islamic Republic of)	16.8 (12.2–22.1)	2.1 (1.3–3.2)	<0.1 (<0.1–0.1)	<0.1 (<0.1–0.1)	18.7 (12.2–26.5)	18.6 (12.1–26.2)	4.3 (1.7–7.0)	4.2 (1.7–6.9)	9.0 (5.3–12.8)	9.9 (6.1–13.9)	0.5 (0.2–0.8)	0.1 (0.1–0.3)	2.9 (1.9–4.0)	2.9 (1.8–3.9)	13.6 (10.6–16.8)	13.8 (10.7–17.0)
Iraq	33.0 (26.2–40.0)	5.4 (3.5–7.6)	<0.1 (<0.1–0.1)	0.1 (<0.1–0.2)	22.7 (14.6–32.4)	22.7 (14.6–32.3)	2.7 (1.1–4.5)	2.7 (1.1–4.5)	12.3 (7.4–17.3)	13.2 (8.4–18.2)	0.2 (0.0–0.6)	<0.1 (0.0–0.2)	10.8 (7.5–13.8)	10.8 (7.5–13.8)	4.7 (<0.1–10.6)	4.7 (<0.1–10.6)
Jordan	32.5 (25.5–39.7)	7.4 (4.8–10.5)	<0.1 (<0.1–<0.1)	<0.1 (<0.1–<0.1)	15.7 (9.8–23.4)	15.7 (9.8–23.4)	4.2 (1.7–6.9)	4.2 (1.7–6.9)	8.3 (5.0–11.7)	11.2 (6.9–16.0)	0.2 (0.0–0.7)	<0.1 (0.0–0.2)	3.2 (1.7–4.9)	3.2 (1.7–4.9)	6.0 (<0.1–12.1)	6.0 (<0.1–12.1)
Kuwait	23.9 (18.4–29.7)	3.6 (2.1–5.6)	<0.1 (<0.1–<0.1)	<0.1 (<0.1–<0.1)	27.1 (18.5–36.9)	27.1 (18.5–36.9)	3.7 (1.5–6.0)	3.7 (1.5–6.0)	10.7 (6.7–15.0)	14.1 (9.0–19.4)	<0.1 (0.0–0.1)	<0.1 (0.0–<0.1)	13.1 (7.6–18.0)	13.1 (7.6–18.0)	3.1 (<0.1–15.6)	3.1 (<0.1–15.6)
Lebanon	32.8 (26.3–40.1)	21.1 (15.2–27.3)	<0.1 (<0.1–0.1)	0.1 (<0.1–0.1)	14.9 (9.0–22.6)	14.9 (9.0–22.6)	5.0 (2.1–8.0)	5.0 (2.1–8.0)	13.5 (8.8–18.9)	12.7 (7.8–17.6)	1.1 (0.4–2.0)	0.2 (0.0–0.5)	0.1 (<0.1–0.2)	0.1 (<0.1–0.2)	14.0 (10.4–17.5)	14.0 (10.4–17.5)
Libya	23.4 (17.5–29.4)	0.8 (0.4–1.3)	<0.1 (<0.1–0.1)	0.1 (<0.1–0.1)	19.0 (11.0–29.3)	19.0 (11.0–29.3)	4.5 (1.9–7.4)	4.5 (1.9–7.4)	13.1 (8.5–18.1)	12.1 (7.9–16.7)	0.2 (<0.1–0.4)	0.1 (<0.1–0.2)	8.2 (4.7–12.0)	8.2 (4.7–12.0)	2.8 (<0.1–9.8)	2.8 (<0.1–9.8)
Morocco	15.5 (11.0–20.2)	0.9 (0.5–1.4)	0.9 (0.3–1.8)	1.3 (0.5–2.7)	17.4 (10.8–25.2)	17.3 (10.7–24.9)	7.6 (3.2–12.2)	7.6 (3.2–12.2)	7.9 (4.7–11.5)	8.6 (5.0–12.3)	0.1 (0.0–0.4)	<0.1 (0.0–<0.1)	2.5 (1.6–3.4)	2.5 (1.6–3.4)	8.2 (4.8–11.8)	8.2 (4.8–11.8)
Oman	13.7 (9.8–18.1)	2.2 (1.2–3.6)	0.1 (<0.1–0.2)	0.1 (<0.1–0.3)	21.4 (12.7–31.3)	21.4 (12.7–31.3)	5.0 (2.0–8.2)	5.0 (2.0–8.2)	8.7 (5.1–12.4)	8.1 (5.1–11.7)	0.1 (0.0–0.4)	<0.1 (0.0–0.1)	6.6 (<0.1–22.3)	6.6 (<0.1–22.3)	1.3 (<0.1–13.3)	1.3 (<0.1–13.3)
Palestine	31.7 (24.7–39.0)	3.6 (2.0–5.6)	0.2 (0.1–0.4)	0.3 (0.1–0.6)	15.9 (9.5–24.7)	15.9 (9.5–24.7)	7.1 (3.0–11.4)	7.1 (3.0–11.4)	8.3 (5.1–11.9)	11.9 (7.5–16.6)	1.2 (0.5–1.8)	0.4 (0.1–0.8)	2.2 (0.9–4.0)	2.2 (0.9–4.0)	3.9 (<0.1–10.5)	3.9 (<0.1–10.5)
Qatar	19.5 (14.5–25.0)	1.2 (0.6–2.1)	<0.1 (<0.1–<0.1)	<0.1 (<0.1–<0.1)	31.1 (22.0–41.1)	31.1 (22.0–41.1)	3.8 (1.5–6.3)	3.8 (1.5–6.3)	10.5 (6.7–15.1)	11.2 (7.1–15.5)	0.2 (0.0–0.7)	<0.1 (0.0–0.2)	10.6 (3.7–19.5)	10.6 (3.7–19.5)	1.8 (<0.1–15.2)	1.8 (<0.1–15.2)

	Smoking		Household air pollution		Ambient particulate matter		Handwashing		Secondhand smoke		Alcohol use		High temperature		Low temperature	
	**M**	**F**	**M**	**F**	**M**	**F**	**M**	**F**	**M**	**F**	**M**	**F**	**M**	**F**	**M**	**F**
Saudi Arabia	15.4 (11.1–20.3)	1.8 (1.0–3.0)	<0.1 (<0.1–0.1)	0.1 (<0.1–0.2)	27.0 (18.4–37.3)	27.0 (18.4–37.3)	4.2 (1.7–6.7)	4.2 (1.7–6.7)	10.7 (6.8–15.2)	12.2 (7.9–16.9)	0.1 (0.0–0.5)	<0.1 (0.0–0.1)	9.7 (5.9–14.1)	9.7 (5.9–14.1)	2.4 (<0.1–8.8)	2.4 (<0.1–8.8)
Sudan	21.2 (15.5–27.6)	2.0 (1.2–3.1)	11.6 (5.6–20.4)	16.2 (9.1–25.7)	20.9 (12.0–31.6)	19.2 (11.2–29.1)	17.7 (7.9–26.7)	17.7 (7.9–26.7)	11.1 (7.5–15.6)	10.7 (7.1–14.7)	<0.1 (0.0–<0.1)	<0.1 (0.0–<0.1)	8.3 (3.6–15.7)	8.3 (3.6–15.7)	1.5 (<0.1–7.2)	1.5 (<0.1–7.2)
Syrian Arab Republic	28.8 (21.8–36.2)	3.6 (2.1–5.8)	<0.1 (<0.1–<0.1)	<0.1 (<0.1–0.1)	15.8 (9.6–23.6)	15.8 (9.6–23.6)	4.9 (2.0–7.9)	4.9 (2.0–7.9)	9.0 (5.5–12.8)	9.5 (5.8–13.4)	0.6 (0.2–1.1)	<0.1 (0.0–0.1)	4.4 (2.5–6.3)	4.4 (2.5–6.3)	7.7 (2.5–12.6)	7.7 (2.5–12.6)
Tunisia	33.5 (26.2–40.7)	2.5 (1.4–3.8)	<0.1 (<0.1–0.1)	0.1 (<0.1–0.1)	15.5 (8.9–24.1)	15.5 (8.9–24.1)	5.7 (2.4–9.2)	5.7 (2.4–9.2)	10.5 (6.5–14.9)	12.3 (7.6–17.1)	0.8 (0.1–1.6)	0.1 (0.0–0.3)	4.5 (2.3–6.7)	4.5 (2.3–6.7)	5.1 (<0.1–11.3)	5.1 (<0.1–11.3)
Turkey	26.4 (20.4–32.6)	4.1 (2.7–5.9)	0.1 (<0.1–0.2)	0.1 (<0.1–0.2)	13.5 (8.4–20.4)	13.5 (8.4–20.3)	5.8 (2.4–9.3)	5.8 (2.4–9.3)	8.7 (5.4–12.6)	11.4 (7.1–15.9)	0.6 (0.1–1.3)	0.3 (0.0–0.7)	0.4 (0.1–0.7)	0.4 (0.1–0.7)	18.3 (13.7–23.4)	18.3 (13.7–23.4)
United Arab Emirates	18.3 (13.2–23.6)	3.6 (2.0–5.8)	<0.1 (<0.1–<0.1)	<0.1 (<0.1–<0.1)	21.1 (13.2–30.8)	21.1 (13.2–30.8)	3.8 (1.5–6.2)	3.8 (1.5–6.2)	13.2 (8.2–18.2)	13.2 (8.6–18.2)	0.5 (0.0–1.6)	0.1 (0.0–0.4)	8.4 (<0.1–25.0)	8.4 (<0.1–25.0)	1.9 (<0.1–18.2)	1.9 (<0.1–18.2)
Yemen	27.7 (21.8–34.1)	7.2 (4.7–10.4)	12.1 (5.8–21.8)	16.3 (8.8–25.7)	17.3 (9.2–27.6)	15.5 (7.9–24.7)	13.5 (5.8–20.7)	13.5 (5.8–20.7)	11.7 (7.4–16.4)	12.3 (7.9–16.9)	0.3 (<0.1–0.6)	0.1 (0.0–0.2)	5.5 (1.6–12.3)	5.5 (1.6–12.3)	0.9 (<0.1–5.1)	0.9 (<0.1–5.1)
**South Asia**	**22.5 (16.2–28.7)**	**5.2 (3.3–7.2)**	**15.3 (8.5–24.2)**	**19.9 (12.7–28.5)**	**24.7 (18.4–31.2)**	**22.2 (16.4–28.3)**	**12.1 (5.2–18.7)**	**11.8 (5.1–18.1)**	**6.8 (3.5–10.3)**	**8.5 (4.7–12.7)**	**3.1 (1.1–5.3)**	**0.2 (<0.1–0.4)**	**6.9 (3.8–11.3)**	**6.7 (3.6–11.2)**	**2.3 (<0.1–6.7)**	**2.2 (<0.1–6.4)**
Bangladesh	23.3 (15.2–31.3)	3.4 (1.9–5.2)	21.5 (11.9–33.7)	28.4 (18.0–40.0)	20.0 (13.8–26.5)	17.3 (11.5–23.3)	18.1 (8.1–27.1)	18.1 (8.1–27.1)	6.4 (3.1–10.3)	9.0 (4.4–14.2)	0.4 (0.0–1.0)	0.1 (0.0–0.2)	5.9 (2.7–9.8)	5.9 (2.7–9.8)	1.3 (<0.1–4.8)	1.3 (<0.1–4.8)
Bhutan	12.8 (8.6–17.5)	3.0 (1.8–4.6)	16.3 (8.1–27.9)	21.6 (12.9–32.9)	15.2 (10.1–21.3)	13.3 (8.4–19.1)	6.1 (2.5–9.8)	6.1 (2.5–9.8)	4.2 (2.3–6.4)	4.6 (2.5–7.1)	0.8 (0.0–2.3)	<0.1 (0.0–0.2)	<0.1 (<0.1–0.1)	<0.1 (<0.1–0.1)	18.6 (11.8–26.9)	18.6 (11.8–26.9)
India	22.0 (15.8–28.1)	5.2 (3.2–7.2)	14.4 (8.0–22.8)	19.0 (12.1–27.3)	25.5 (18.9–31.9)	22.7 (16.9–28.9)	11.5 (5.0–17.9)	11.4 (4.9–17.5)	6.9 (3.6–10.6)	8.6 (4.7–12.7)	3.6 (1.3–6.2)	0.2 (<0.1–0.4)	7.0 (3.8–11.6)	6.7 (3.5–11.4)	2.0 (<0.1–6.4)	2.0 (<0.1–6.2)
Nepal	26.0 (17.0–34.4)	17.1 (10.2–23.9)	22.4 (12.6–35.1)	28.9 (18.4–40.2)	22.8 (15.0–31.2)	19.4 (12.0–27.6)	11.3 (4.9–17.8)	11.3 (4.9–17.8)	5.6 (2.4–9.2)	4.8 (2.1–8.2)	2.3 (0.0–5.8)	0.3 (0.0–0.8)	1.4 (0.7–2.2)	1.4 (0.7–2.2)	13.0 (7.6–19.0)	13.0 (7.6–19.0)
Pakistan	26.8 (19.6–33.8)	4.1 (2.4–6.4)	17.4 (9.6–27.0)	22.9 (14.0–32.4)	20.8 (14.2–28.6)	18.4 (12.6–25.4)	12.3 (5.2–19.2)	12.3 (5.2–19.1)	6.1 (3.3–9.4)	8.7 (4.8–13.2)	0.6 (0.1–1.3)	<0.1 (0.0–0.1)	9.2 (5.6–15.5)	9.2 (5.6–15.6)	4.9 (<0.1–12.6)	5.0 (<0.1–12.6)
**Southeast Asia, East Asia, and Oceania**	**31.9 (24.7–39.0)**	**5.6 (3.4–7.9)**	**6.1 (2.8–10.9)**	**9.7 (5.2–15.4)**	**15.6 (10.6–21.3)**	**14.5 (9.9–19.6)**	**5.0 (2.1–8.0)**	**5.2 (2.1–8.2)**	**8.6 (5.2–12.2)**	**12.8 (7.9–17.7)**	**6.7 (3.1–10.0)**	**0.9 (0.2–1.7)**	**1.3 (0.2–4.3)**	**1.4 (0.1–5.1)**	**6.7 (4.2–9.2)**	**6.2 (3.8–8.9)**
East Asia	34.3 (27.0–41.2)	5.4 (3.5–7.5)	4.4 (1.9–8.2)	7.1 (3.5–12.3)	19.5 (13.5–26.5)	18.8 (12.9–25.2)	3.6 (1.5–5.9)	3.6 (1.5–6.0)	9.8 (6.2–13.9)	15.3 (10.0–20.9)	8.0 (3.9–12.0)	1.2 (0.3–2.2)	0.8 (0.2–1.5)	0.8 (0.2–1.5)	11.4 (7.4–15.0)	11.4 (7.6–15.0)
China	35.5 (28.1–42.4)	5.8 (3.7–8.1)	4.4 (1.8–8.3)	6.6 (3.1–11.8)	20.3 (14.0–27.3)	19.4 (13.5–26.1)	3.8 (1.5–6.3)	3.8 (1.5–6.3)	9.9 (6.2–14.0)	15.7 (10.2–21.3)	8.2 (4.1–12.5)	1.3 (0.3–2.4)	0.8 (0.2–1.5)	0.9 (0.2–1.6)	11.9 (7.7–15.6)	11.6 (7.8–15.2)
Democratic People's Republic of Korea	32.1 (24.6–40.1)	3.6 (1.9–5.8)	19.5 (10.2–31.3)	26.2 (16.0–38.1)	16.4 (10.4–22.7)	14.4 (9.2–20.5)	2.3 (0.9–3.7)	2.3 (0.9–3.7)	8.3 (4.9–11.9)	14.2 (9.0–19.9)	5.5 (1.8–9.3)	0.4 (0.0–0.9)	0.1 (<0.1–0.7)	0.1 (<0.1–0.7)	19.3 (11.7–29.7)	19.3 (11.7–29.7)
Taiwan (Province of China)	21.7 (15.5–27.9)	0.8 (0.4–1.2)	0.4 (0.1–1.2)	0.7 (0.1–1.8)	12.1 (7.5–17.9)	12.1 (7.5–17.9)	1.8 (0.7–3.0)	1.8 (0.7–3.0)	9.1 (5.6–13.1)	10.9 (6.7–15.8)	5.9 (2.1–9.7)	0.5 (<0.1–1.2)	0.9 (0.2–2.3)	0.9 (0.2–2.3)	3.5 (<0.1–6.8)	3.5 (<0.1–6.8)

	Smoking		Household air pollution		Ambient particulate matter		Handwashing		Secondhand smoke		Alcohol use		High temperature		Low temperature	
	M	F	M	F	M	F	M	F	M	F	M	F	M	F	M	F
Oceania	17.9 (11.6–24.4)	8.8 (5.2–12.8)	30.8 (18.3–43.0)	36.6 (25.4–46.6)	4.6 (1.5–11.1)	3.8 (1.3–9.2)	9.2 (4.0–14.5)	9.2 (3.9–14.7)	7.3 (3.6–11.6)	8.5 (4.2–13.4)	0.8 (0.0–2.5)	<0.1 (0.0–0.2)	0.5 (<0.1–2.2)	0.5 (<0.1–2.2)	1.0 (<0.1–2.4)	1.0 (<0.1–2.4)
Fiji	20.7 (13.6–28.3)	4.6 (2.4–7.4)	4.2 (1.2–9.4)	6.4 (2.2–13.1)	5.4 (1.4–13.3)	5.4 (1.5–13.1)	2.9 (1.2–4.8)	2.9 (1.2–4.8)	5.7 (2.5–9.6)	7.1 (3.3–11.7)	2.8 (0.7–5.2)	0.1 (0.0–0.3)	0.5 (<0.1–2.4)	0.5 (<0.1–2.4)	<0.1 (<0.1–1.7)	<0.1 (<0.1–1.7)
Kiribati	39.5 (31.2–47.1)	23.1 (15.0–30.9)	24.1 (12.5–37.1)	31.0 (19.8–43.3)	3.8 (1.3–8.6)	3.2 (1.1–7.3)	6.5 (2.7–10.4)	6.5 (2.7–10.4)	9.5 (4.9–14.7)	10.7 (5.7–16.3)	0.5 (0.0–2.3)	<0.1 (0.0–0.1)	0.9 (<0.1–5.0)	0.9 (<0.1–5.0)	<0.1 (<0.1–0.1)	<0.1 (<0.1–0.1)
Marshall Islands	15.5 (10.3–21.4)	4.8 (2.5–7.7)	7.5 (3.0–14.4)	10.9 (5.1–18.9)	4.4 (1.6–9.2)	4.2 (1.6–8.7)	4.5 (1.8–7.3)	4.5 (1.8–7.3)	7.0 (3.6–11.0)	8.6 (4.5–13.0)	1.3 (0.0–4.6)	<0.1 (0.0–0.3)	0.2 (<0.1–11.3)	0.2 (<0.1–11.3)	<0.1 (<0.1–0.1)	<0.1 (<0.1–0.1)
Micronesia (Federated States of)	24.3 (16.3–32.5)	9.7 (5.0–15.3)	6.6 (2.4–13.4)	9.8 (4.1–18.1)	5.1 (1.5–12.2)	4.9 (1.5–11.4)	3.9 (1.6–6.4)	3.9 (1.6–6.4)	6.1 (2.6–10.3)	6.9 (3.0–11.7)	1.4 (0.0–3.1)	<0.1 (0.0–0.1)	0.2 (<0.1–11.3)	0.2 (<0.1–11.3)	<0.1 (<0.1–0.2)	<0.1 (<0.1–0.2)
Nauru	20.0 (12.8–27.7)	10.7 (5.7–16.5)	0.5 (0.1–1.5)	0.9 (0.2–2.2)	1.9 (0.4–5.1)	2.0 (0.6–5.2)	3.1 (1.2–5.1)	3.1 (1.2–5.1)	6.6 (2.9–11.0)	7.6 (3.3–12.6)	3.6 (0.2–7.4)	0.1 (0.0–0.5)	2.4 (<0.1–31.1)	2.4 (<0.1–31.1)	<0.1 (<0.1–0.3)	<0.1 (<0.1–0.3)
Palau	16.3 (10.8–22.1)	4.2 (2.3–7.0)	<0.1 (<0.1–<0.1)	<0.1 (<0.1–<0.1)	2.0 (<0.1–5.9)	2.0 (<0.1–5.9)	2.4 (0.9–3.9)	2.4 (0.9–3.9)	9.3 (5.1–14.2)	10.6 (5.8–15.9)	1.3 (0.0–4.2)	<0.1 (0.0–0.2)	0.9 (<0.1–28.7)	0.9 (<0.1–28.7)	<0.1 (<0.1–0.4)	<0.1 (<0.1–0.4)
Papua New Guinea	13.8 (7.9–20.2)	9.5 (5.4–14.6)	37.9 (22.7–52.8)	44.8 (31.5–56.7)	4.7 (1.3–12.1)	3.7 (1.0–9.9)	12.2 (5.2–19.0)	12.2 (5.2–19.0)	7.3 (3.4–12.0)	8.4 (3.9–13.7)	0.5 (0.0–2.3)	<0.1 (0.0–0.1)	0.4 (<0.1–1.9)	0.4 (<0.1–1.9)	1.6 (<0.1–3.7)	1.6 (<0.1–3.7)
Samoa	29.2 (22.2–36.4)	8.7 (5.4–13.0)	14.8 (6.6–26.4)	20.6 (11.2–32.3)	5.2 (1.4–12.2)	4.7 (1.3–10.9)	2.2 (0.9–3.6)	2.2 (0.9–3.6)	11.1 (6.6–16.0)	12.7 (7.4–18.2)	1.5 (0.0–4.5)	<0.1 (0.0–0.3)	0.4 (<0.1–2.2)	0.4 (<0.1–2.2)	0.1 (<0.1–1.4)	0.1 (<0.1–1.4)
Solomon Islands	29.4 (21.2–38.1)	9.8 (5.9–14.7)	38.1 (23.2–53.1)	45.4 (31.8–57.7)	3.9 (1.2–9.2)	3.1 (0.9–7.2)	4.9 (2.0–7.9)	4.9 (2.0–7.9)	7.5 (3.7–11.7)	9.0 (4.5–14.0)	0.4 (0.0–2.1)	<0.1 (0.0–0.1)	0.5 (<0.1–3.3)	0.5 (<0.1–3.3)	<0.1 (<0.1–0.5)	<0.1 (<0.1–0.5)
Tonga	30.6 (22.2–39.1)	6.7 (3.6–10.6)	6.7 (2.3–13.4)	9.9 (4.0–18.4)	5.3 (1.5–12.4)	5.1 (1.6–11.6)	3.3 (1.3–5.4)	3.3 (1.3–5.4)	8.3 (4.3–12.7)	10.1 (5.5–15.1)	0.6 (0.0–2.4)	<0.1 (0.0–0.2)	1.2 (<0.1–3.0)	1.2 (<0.1–3.0)	0.2 (<0.1–2.6)	0.2 (<0.1–2.6)
Tuvalu	21.0 (14.3–27.9)	7.5 (4.0–11.8)	1.6 (0.4–4.1)	2.6 (0.8–5.6)	2.4 (0.8–5.6)	2.5 (1.0–5.6)	3.7 (1.5–6.1)	3.7 (1.5–6.1)	7.8 (3.8–12.3)	8.9 (4.5–13.7)	0.8 (0.0–2.7)	<0.1 (0.0–0.2)	1.4 (<0.1–12.8)	1.4 (<0.1–12.8)	0.1 (<0.1–0.7)	0.1 (<0.1–0.7)
Vanuatu	19.9 (12.4–27.4)	3.2 (1.5–5.3)	28.4 (15.5–42.8)	36.3 (23.3–49.6)	4.9 (1.5–10.9)	4.1 (1.2–9.2)	10.0 (4.3–15.7)	10.0 (4.3–15.7)	4.3 (1.8–7.4)	4.4 (1.9–7.4)	1.2 (<0.1–3.0)	<0.1 (0.0–0.1)	0.4 (<0.1–2.1)	0.4 (<0.1–2.1)	<0.1 (<0.1–1.8)	<0.1 (<0.1–1.8)
Southeast Asia	28.5 (20.6–36.2)	5.7 (3.1–8.6)	8.5 (4.0–14.5)	12.5 (6.9–19.4)	10.1 (6.6–14.4)	9.5 (6.4–13.5)	6.8 (2.8–11.0)	7.0 (2.9–11.1)	6.8 (3.7–10.1)	9.9 (5.7–14.7)	4.9 (1.9–7.8)	0.6 (0.0–1.3)	1.9 (<0.1–8.8)	2.0 (<0.1–10.3)	<0.1 (<0.1–1.2)	<0.1 (<0.1–1.2)
Cambodia	42.2 (33.1–51.0)	7.5 (4.5–11.2)	26.8 (14.9–40.5)	34.3 (22.1–47.2)	8.1 (4.5–13.0)	6.8 (3.9–10.9)	8.6 (3.5–13.5)	8.6 (3.5–13.5)	5.1 (3.0–7.4)	12.2 (7.4–16.9)	7.5 (2.4–12.5)	0.7 (0.0–1.7)	2.7 (<0.1–21.8)	2.7 (<0.1–21.8)	<0.1 (<0.1–0.3)	<0.1 (<0.1–0.3)
Indonesia	31.9 (22.0–41.7)	4.0 (2.2–6.2)	6.0 (2.4–11.7)	9.1 (4.1–16.1)	9.5 (5.9–14.2)	9.1 (5.8–13.3)	7.2 (3.0–11.5)	7.3 (3.0–11.6)	5.5 (2.9–8.3)	10.9 (6.1–16.0)	0.1 (0.0–0.7)	<0.1 (0.0–<0.1)	0.3 (<0.1–3.3)	0.3 (<0.1–3.3)	<0.1 (<0.1–0.9)	<0.1 (<0.1–0.9)
Lao People's Democratic Republic	32.9 (23.6–41.9)	4.7 (2.4–7.9)	28.9 (16.1–43.2)	36.9 (23.8–50.3)	7.5 (4.3–11.6)	6.3 (3.6–9.6)	10.2 (4.3–15.9)	10.2 (4.3–15.9)	6.6 (3.6–10.2)	11.8 (6.6–17.5)	3.6 (0.0–7.9)	0.8 (0.0–2.2)	1.8 (0.6–3.9)	1.8 (0.6–3.9)	<0.1 (<0.1–2.9)	<0.1 (<0.1–2.9)
Malaysia	26.1 (19.0–33.3)	3.0 (1.6–4.8)	0.1 (<0.1–0.2)	0.1 (<0.1–0.3)	8.3 (4.7–13.2)	8.3 (4.7–13.2)	2.0 (0.8–3.2)	2.0 (0.8–3.2)	7.0 (3.7–10.5)	10.8 (6.2–15.6)	0.9 (0.0–2.2)	0.1 (0.0–0.4)	0.8 (<0.1–3.9)	0.8 (<0.1–3.9)	<0.1 (<0.1–0.8)	<0.1 (<0.1–0.8)

	Smoking		Household air pollution		Ambient particulate matter		Handwashing		Secondhand smoke		Alcohol use		High temperature		Low temperature	
	M	F	M	F	M	F	M	F	M	F	M	F	M	F	M	F
Maldives	30.6 (21.7–39.1)	4.2 (2.1–7.2)	2.1 (0.7–5.0)	3.2 (1.2–7.0)	5.2 (2.9–8.8)	5.2 (3.0–8.4)	8.1 (3.5–13.0)	8.1 (3.5–13.0)	7.7 (4.3–11.5)	11.9 (6.9–17.4)	0.4 (0.0–2.1)	<0.1 (0.0–0.2)	0.4 (<0.1–11.4)	0.4 (<0.1–11.4)	<0.1 (<0.1–0.1)	<0.1 (<0.1–0.1)
Mauritius	23.7 (17.1–30.6)	2.1 (1.0–3.3)	0.1 (<0.1–0.4)	0.2 (0.1–0.5)	7.3 (3.0–13.8)	7.3 (3.1–13.7)	1.1 (0.4–1.8)	1.1 (0.4–1.8)	7.2 (3.9–10.9)	10.5 (5.8–15.5)	4.1 (0.0–8.5)	0.2 (0.0–1.1)	0.7 (<0.1–3.5)	0.7 (<0.1–3.5)	<0.1 (<0.1–5.4)	<0.1 (<0.1–5.4)
Myanmar	26.5 (17.6–35.3)	10.1 (4.8–16.0)	20.0 (10.1–31.9)	26.8 (16.1–39.0)	11.8 (7.9–16.1)	10.4 (7.0–14.1)	11.1 (4.7–17.5)	11.1 (4.7–17.5)	5.5 (2.3–9.0)	6.4 (2.9–10.7)	3.3 (0.6–6.2)	0.3 (0.0–0.9)	2.9 (1.3–5.3)	2.9 (1.3–5.3)	1.9 (<0.1–4.3)	1.9 (<0.1–4.3)
Philippines	27.7 (20.4–34.6)	9.5 (5.1–14.0)	13.2 (6.1–22.5)	16.8 (8.8–25.9)	8.4 (5.4–12.1)	8.2 (5.4–11.6)	6.5 (2.7–10.2)	6.3 (2.6–10.0)	9.7 (5.3–14.3)	10.0 (5.6–14.7)	5.2 (1.5–9.0)	1.0 (0.0–2.4)	2.4 (<0.1–21.9)	2.4 (<0.1–22.9)	0.2 (<0.1–0.8)	0.2 (<0.1–0.8)
Seychelles	30.0 (21.9–38.6)	3.5 (1.8–5.9)	0.1 (<0.1–0.2)	0.1 (<0.1–0.2)	7.7 (3.3–14.3)	7.7 (3.3–14.3)	3.9 (1.6–6.3)	3.9 (1.6–6.3)	7.2 (4.0–10.9)	8.7 (4.9–12.8)	7.3 (2.2–12.6)	0.6 (0.0–1.8)	1.0 (<0.1–3.8)	1.0 (<0.1–3.8)	0.2 (<0.1–1.3)	0.2 (<0.1–1.3)
Sri Lanka	17.1 (9.8–25.1)	1.7 (0.9–2.8)	7.6 (2.7–15.6)	11.1 (4.6–21.3)	10.0 (5.8–15.6)	9.6 (5.7–14.8)	5.0 (2.0–8.1)	5.0 (2.0–8.1)	3.7 (1.5–6.5)	5.3 (2.2–9.2)	6.9 (3.2–10.8)	0.9 (0.2–1.8)	1.2 (<0.1–13.3)	1.2 (<0.1–13.3)	<0.1 (<0.1–0.8)	<0.1 (<0.1–0.8)
Thailand	25.6 (17.5–33.7)	3.6 (1.9–5.5)	2.7 (0.9–5.8)	4.0 (1.5–8.2)	13.8 (8.8–20.0)	13.6 (8.8–19.6)	7.5 (3.1–12.1)	7.5 (3.1–12.1)	5.3 (2.8–8.0)	8.1 (4.4–12.3)	8.7 (3.9–13.6)	0.9 (0.0–1.9)	2.9 (<0.1–10.6)	2.9 (<0.1–10.6)	<0.1 (<0.1–1.0)	<0.1 (<0.1–1.0)
Timor-Leste	28.3 (19.3–37.5)	3.3 (1.5–5.8)	25.3 (13.2–39.3)	32.9 (20.8–46.6)	6.2 (3.0–11.2)	5.3 (2.5–9.6)	6.6 (2.7–10.6)	6.6 (2.7–10.6)	6.0 (2.7–9.6)	7.9 (3.8–12.7)	3.1 (0.4–6.2)	0.2 (0.0–0.7)	0.5 (<0.1–2.1)	0.5 (<0.1–2.1)	<0.1 (<0.1–1.7)	<0.1 (<0.1–1.7)
Viet Nam	32.2 (24.9–39.6)	2.6 (1.3–4.4)	7.6 (3.0–14.6)	11.1 (5.2–19.5)	10.0 (6.4–14.7)	9.6 (6.3–13.8)	8.6 (3.6–13.6)	8.6 (3.6–13.6)	7.8 (4.4–11.4)	12.2 (7.3–17.3)	10.7 (4.0–17.5)	0.3 (0.0–0.9)	2.7 (0.9–5.9)	2.7 (0.9–5.9)	0.2 (<0.1–3.2)	0.2 (<0.1–3.2)
**Sub-Saharan Africa**	**10.6 (6.7–14.4)**	**2.5 (1.4–3.7)**	**31.0 (20.2–41.4)**	**35.2 (25.4–44.7)**	**11.8 (7.6–16.8)**	**10.0 (6.5–14.1)**	**21.6 (9.9–31.9)**	**21.4 (9.9–31.5)**	**2.8 (1.4–4.5)**	**3.2 (1.5–5.1)**	**4.9 (1.9–7.7)**	**1.5 (0.3–2.8)**	**3.2 (0.5–10.5)**	**2.9 (0.5–9.7)**	**1.6 (<0.1–3.3)**	**1.7 (0.1–3.5)**
Central Sub-Saharan Africa	10.0 (5.8–14.3)	0.9 (0.5–1.5)	32.0 (19.8–44.2)	39.5 (27.4–50.3)	10.2 (5.4–17.4)	8.2 (4.0–14.2)	22.6 (10.4–33.2)	22.6 (10.5–33.1)	1.4 (0.6–2.5)	1.7 (0.7–3.0)	3.2 (<0.1–6.5)	1.2 (0.0–3.0)	1.4 (0.2–3.2)	1.4 (0.3–3.3)	<0.1 (<0.1–2.4)	<0.1 (<0.1–2.4)
Angola	15.8 (11.0–21.1)	1.9 (1.0–3.2)	14.7 (7.3–24.7)	19.8 (11.5–30.0)	11.8 (6.2–19.1)	10.5 (5.5–17.2)	22.3 (10.2–32.8)	22.3 (10.2–32.8)	2.7 (1.4–4.3)	4.3 (2.3–6.4)	8.7 (3.6–13.7)	3.9 (0.9–7.1)	1.7 (0.5–3.8)	1.7 (0.5–3.8)	<0.1 (<0.1–2.9)	<0.1 (<0.1–2.9)
Central African Republic	10.5 (6.5–15.2)	1.3 (0.6–2.2)	46.8 (31.2–58.9)	51.4 (39.6–63.8)	7.9 (2.5–17.1)	5.7 (1.8–12.6)	21.6 (9.9–31.8)	21.6 (9.9–31.8)	2.2 (1.0–3.8)	2.6 (1.1–4.3)	2.1 (0.0–6.3)	0.8 (0.0–2.8)	3.8 (1.5–7.0)	3.8 (1.5–7.0)	<0.1 (<0.1–1.2)	<0.1 (<0.1–1.2)
Congo	14.9 (9.6–20.8)	0.8 (0.4–1.4)	11.8 (5.4–21.2)	16.5 (9.1–26.5)	16.5 (9.0–27.7)	15.3 (8.2–25.8)	21.0 (9.5–31.0)	21.0 (9.5–31.0)	2.5 (1.1–4.0)	3.0 (1.4–4.9)	4.9 (0.0–10.3)	2.5 (0.0–6.1)	0.9 (<0.1–3.0)	0.9 (<0.1–3.0)	<0.1 (<0.1–1.9)	<0.1 (<0.1–1.9)
Democratic Republic of the Congo	8.1 (4.0–12.7)	0.7 (0.3–1.3)	38.4 (23.9–52.2)	45.2 (32.1–56.6)	9.2 (4.4–16.5)	7.1 (3.0–13.1)	23.0 (10.6–33.7)	23.0 (10.6–33.7)	0.9 (0.2–1.8)	1.1 (0.3–2.2)	1.5 (0.0–4.7)	0.6 (0.0–2.3)	1.3 (0.1–3.1)	1.3 (0.1–3.1)	0.1 (<0.1–2.6)	0.1 (<0.1–2.6)
Equatorial Guinea	12.0 (7.1–17.5)	1.0 (0.5–1.8)	3.2 (1.2–6.7)	4.8 (1.9–9.3)	20.5 (10.1–33.9)	20.0 (10.1–33.1)	19.9 (9.0–29.7)	19.9 (9.0–29.7)	2.3 (0.9–4.2)	2.9 (1.1–4.9)	7.7 (2.4–13.6)	3.4 (0.4–7.3)	0.4 (<0.1–1.3)	0.4 (<0.1–1.3)	<0.1 (<0.1–2.4)	<0.1 (<0.1–2.4)
Gabon	9.7 (5.7–14.3)	0.9 (0.4–1.7)	1.2 (0.4–2.5)	1.8 (0.7–3.5)	17.8 (9.2–30.0)	17.7 (9.2–29.8)	16.9 (7.4–25.6)	16.9 (7.4–25.6)	2.6 (1.1–4.4)	2.9 (1.3–4.9)	9.5 (3.4–15.7)	4.3 (0.9–8.7)	0.7 (<0.1–2.6)	0.7 (<0.1–2.6)	<0.1 (<0.1–1.5)	<0.1 (<0.1–1.5)
Eastern Sub-Saharan Africa	11.4 (6.9–16.1)	3.3 (1.7–4.9)	39.3 (26.2–51.3)	44.9 (32.8–55.7)	7.2 (4.0–11.2)	5.8 (3.2–9.2)	22.6 (10.4–33.2)	22.5 (10.4–33.1)	2.0 (0.8–3.5)	2.3 (1.0–3.9)	4.6 (1.7–7.5)	1.5 (0.4–2.7)	2.0 (0.4–5.3)	1.7 (0.4–4.6)	2.1 (<0.1–4.6)	1.7 (<0.1–4.3)

	Smoking		Household air pollution		Ambient particulate matter		Handwashing		Secondhand smoke		Alcohol use		High temperature		Low temperature	
	M	F	M	F	M	F	M	F	M	F	M	F	M	F	M	F
Burundi	9.5 (5.1–14.3)	1.5 (0.6–2.9)	48.3 (32.9–60.1)	52.7 (40.8–64.6)	5.9 (2.0–12.2)	4.2 (1.3–9.5)	22.9 (10.6–33.7)	22.9 (10.6–33.7)	1.3 (0.5–2.4)	1.2 (0.5–2.3)	8.4 (3.2–13.7)	1.3 (0.3–2.6)	<0.1 (<0.1–0.3)	<0.1 (<0.1–0.3)	<0.1 (<0.1–6.1)	<0.1 (<0.1–6.1)
Comoros	16.9 (9.2–24.6)	1.9 (0.9–3.2)	29.2 (16.9–43.5)	36.9 (24.5–49.6)	6.2 (3.4–9.9)	5.1 (2.8–8.2)	21.0 (9.6–31.1)	21.0 (9.6–31.1)	4.2 (1.9–6.9)	4.7 (2.2–7.6)	0.4 (0.0–1.4)	0.2 (0.0–0.6)	0.7 (<0.1–2.9)	0.7 (<0.1–2.9)	0.2 (<0.1–2.9)	0.2 (<0.1–2.9)
Djibouti	21.9 (13.4–30.3)	3.6 (1.6–6.2)	6.5 (2.7–13.2)	9.3 (4.2–17.1)	18.6 (9.1–32.2)	17.5 (8.5–30.7)	21.6 (9.9–32.0)	21.6 (9.9–32.0)	4.8 (2.1–8.3)	7.8 (3.5–13.2)	0.2 (0.0–1.1)	<0.1 (0.0–0.3)	5.9 (<0.1–30.0)	5.9 (<0.1–30.0)	0.2 (<0.1–5.2)	0.2 (<0.1–5.2)
Eritrea	7.8 (4.6–11.5)	0.3 (0.2–0.6)	29.3 (16.5–42.7)	35.9 (23.0–47.4)	12.8 (6.3–21.5)	10.4 (4.9–17.7)	22.6 (10.4–33.1)	22.6 (10.4–33.1)	3.4 (1.7–5.3)	2.6 (1.3–4.1)	1.0 (0.0–3.4)	0.2 (0.0–1.0)	4.5 (<0.1–22.1)	4.5 (<0.1–22.1)	0.1 (<0.1–2.5)	0.1 (<0.1–2.5)
Ethiopia	6.2 (3.5–9.2)	0.4 (0.1–0.8)	44.5 (29.9–56.3)	49.1 (37.1–60.0)	7.1 (3.2–12.3)	5.5 (2.5–10.1)	23.0 (10.7–33.8)	23.0 (10.7–33.7)	1.0 (0.3–1.9)	0.9 (0.3–1.7)	2.8 (0.0–6.1)	0.7 (0.0–2.2)	2.9 (0.6–9.0)	2.6 (0.6–7.9)	4.7 (3.0–6.7)	4.8 (3.1–6.9)
Kenya	12.8 (8.0–18.1)	2.4 (1.1–4.0)	26.4 (15.3–38.7)	33.5 (22.1–45.4)	7.7 (4.5–11.9)	6.7 (3.9–10.3)	22.2 (10.2–32.7)	22.2 (10.3–32.8)	2.4 (1.1–4.2)	2.5 (1.1–4.2)	5.1 (1.2–9.4)	1.2 (0.3–2.3)	2.1 (0.2–8.3)	1.8 (0.2–6.8)	3.8 (1.9–6.0)	3.7 (1.8–6.0)
Madagascar	7.5 (4.1–11.7)	1.0 (0.4–1.8)	39.8 (24.8–54.2)	47.2 (33.7–59.0)	5.2 (2.7–8.8)	4.0 (2.0–6.9)	22.4 (10.3–33.0)	22.4 (10.3–33.0)	2.0 (0.6–4.1)	2.3 (0.7–4.6)	1.3 (0.0–3.9)	0.3 (0.0–1.1)	0.8 (<0.1–2.4)	0.8 (<0.1–2.4)	1.0 (<0.1–3.8)	1.0 (<0.1–3.8)
Malawi	19.3 (11.8–27.7)	2.6 (1.1–4.3)	42.6 (27.7–56.0)	49.0 (35.8–59.8)	5.6 (2.6–10.0)	4.3 (1.9–7.9)	22.6 (10.4–33.3)	22.6 (10.4–33.3)	1.8 (0.7–3.1)	2.0 (0.8–3.6)	2.8 (0.5–5.9)	0.7 (0.0–2.1)	0.5 (<0.1–1.9)	0.5 (<0.1–1.9)	0.2 (<0.1–4.4)	0.2 (<0.1–4.4)
Mozambique	14.0 (8.2–20.4)	2.8 (1.3–4.8)	47.2 (31.8–59.3)	52.0 (40.4–62.6)	4.4 (1.9–8.0)	3.2 (1.3–6.1)	22.2 (10.2–32.7)	22.2 (10.2–32.7)	1.8 (0.6–3.4)	2.2 (0.7–4.4)	1.1 (0.0–4.0)	0.3 (0.0–1.2)	2.4 (1.0–4.4)	2.4 (1.0–4.4)	0.2 (<0.1–3.3)	0.2 (<0.1–3.3)
Rwanda	23.6 (15.0–32.5)	15.4 (9.3–22.1)	34.8 (21.1–49.2)	42.5 (29.4–55.2)	10.4 (4.9–18.0)	8.3 (3.7–15.3)	23.2 (10.7–34.0)	23.2 (10.7–34.0)	2.8 (1.4–4.5)	2.1 (1.0–3.4)	9.1 (3.1–15.1)	2.4 (0.3–5.1)	<0.1 (<0.1–0.1)	<0.1 (<0.1–0.1)	2.0 (<0.1–7.5)	2.0 (<0.1–7.5)
Somalia	14.1 (7.7–20.5)	2.5 (1.1–4.6)	58.4 (44.7–79.9)	61.4 (46.5–85.0)	2.1 (0.5–6.7)	1.4 (0.3–4.3)	22.7 (10.5–33.4)	22.7 (10.5–33.4)	2.9 (1.2–5.0)	2.9 (1.2–5.2)	0.0 (0.0–0.0)	0.0 (0.0–0.0)	2.3 (<0.1–15.8)	2.3 (<0.1–15.8)	0.1 (<0.1–1.3)	0.1 (<0.1–1.3)
South Sudan	12.8 (7.0–18.8)	2.3 (1.0–4.1)	40.0 (25.6–53.9)	46.8 (33.8–58.2)	9.3 (4.3–15.8)	7.1 (3.2–12.7)	22.9 (10.6–33.6)	22.9 (10.6–33.6)	3.1 (1.3–5.5)	3.6 (1.6–6.2)	0.1 (0.0–0.9)	<0.1 (0.0–0.2)	5.9 (<0.1–23.1)	5.9 (<0.1–23.1)	<0.1 (<0.1–0.9)	<0.1 (<0.1–0.9)
Uganda	9.7 (5.3–14.6)	3.9 (1.8–6.6)	37.3 (23.0–51.3)	44.7 (31.4–56.3)	9.5 (5.0–15.1)	7.5 (3.7–12.3)	22.1 (10.1–32.5)	22.1 (10.1–32.5)	2.1 (0.8–3.7)	1.8 (0.8–3.3)	10.8 (5.1–16.2)	3.7 (1.3–6.8)	1.1 (0.2–2.9)	1.1 (0.2–2.9)	<0.1 (<0.1–2.3)	<0.1 (<0.1–2.3)
United Republic of Tanzania	17.4 (10.5–24.2)	5.4 (2.6–8.8)	36.6 (22.4–51.1)	44.3 (30.7–56.4)	7.4 (4.1–11.4)	5.9 (3.2–9.2)	22.5 (10.4–33.2)	22.5 (10.4–33.2)	3.2 (1.5–5.2)	3.8 (1.8–6.2)	9.5 (4.5–14.5)	3.1 (1.0–5.9)	0.9 (0.1–2.7)	0.9 (0.1–2.7)	0.1 (<0.1–3.5)	0.1 (<0.1–3.5)
Zambia	15.3 (8.4–22.2)	9.5 (5.2–14.0)	23.8 (12.7–36.8)	31.1 (19.5–43.4)	9.9 (5.7–15.7)	8.5 (4.9–13.4)	21.3 (9.7–31.5)	21.3 (9.7–31.5)	2.2 (0.7–4.4)	2.9 (0.9–5.6)	3.4 (0.0–7.4)	2.3 (0.3–4.7)	1.2 (0.3–3.5)	1.2 (0.3–3.5)	<0.1 (<0.1–5.0)	<0.1 (<0.1–5.0)
Southern Sub-Saharan Africa	18.3 (12.4–24.2)	5.7 (3.5–8.1)	7.5 (3.6–12.6)	10.0 (5.6–15.3)	12.7 (8.0–18.6)	12.1 (7.7–17.6)	16.3 (7.2–24.5)	16.4 (7.4–24.7)	4.4 (2.0–7.0)	5.7 (2.7–9.1)	7.5 (3.6–10.9)	2.3 (0.9–4.0)	1.1 (0.5–2.4)	1.1 (0.5–2.3)	6.9 (4.4–9.5)	7.1 (4.6–9.7)
Botswana	25.1 (17.9–32.1)	7.4 (4.4–11.2)	6.6 (2.5–13.1)	9.6 (4.3–17.5)	12.1 (7.2–18.9)	11.6 (7.0–17.6)	17.0 (7.5–25.6)	17.0 (7.5–25.6)	6.7 (3.2–10.7)	8.2 (3.9–12.9)	7.3 (2.6–12.6)	0.8 (0.1–1.7)	4.0 (1.5–7.7)	4.0 (1.5–7.7)	0.9 (<0.1–7.0)	0.9 (<0.1–7.0)
Eswatini	10.0 (5.7–14.5)	6.1 (3.3–9.6)	12.3 (5.5–22.2)	17.3 (9.1–27.7)	10.8 (6.3–16.4)	9.9 (6.0–14.8)	16.5 (7.4–25.0)	16.5 (7.4–25.0)	2.2 (0.9–4.0)	2.5 (1.0–4.4)	7.6 (3.6–11.6)	2.0 (0.3–4.2)	0.4 (<0.1–1.2)	0.4 (<0.1–1.2)	3.3 (<0.1–8.1)	3.3 (<0.1–8.1)

	Smoking		Household air pollution		Ambient particulate matter		Handwashing		Secondhand smoke		Alcohol use		High temperature		Low temperature	
	M	F	M	F	M	F	M	F	M	F	M	F	M	F	M	F
Lesotho	25.4 (18.4–32.7)	5.9 (3.4–8.9)	17.7 (8.8–29.2)	23.8 (13.9–35.1)	11.3 (6.7–17.1)	10.0 (5.9–15.3)	23.1 (10.7–33.9)	23.1 (10.7–33.9)	6.0 (3.3–8.9)	8.0 (4.5–11.6)	3.9 (0.2–7.7)	0.7 (0.0–2.0)	<0.1 (<0.1–<0.1)	<0.1 (<0.1–<0.1)	21.7 (15.2–28.4)	21.7 (15.2–28.4)
Namibia	16.8 (10.1–24.1)	11.6 (6.4–17.1)	9.2 (3.8–17.1)	13.2 (6.4–22.5)	11.5 (6.4–18.2)	10.9 (6.1–16.6)	14.3 (6.2–22.0)	14.3 (6.2–22.0)	3.3 (1.3–6.0)	3.5 (1.3–6.4)	6.5 (1.6–11.5)	1.1 (0.0–3.5)	3.2 (1.3–6.0)	3.2 (1.3–6.0)	1.3 (<0.1–6.0)	1.3 (<0.1–6.0)
South Africa	15.5 (9.9–21.0)	5.1 (2.9–7.5)	2.5 (1.0–4.9)	4.0 (1.7–7.2)	14.1 (8.8–20.8)	13.6 (8.6–19.9)	14.9 (6.6–22.5)	15.1 (6.7–23.0)	4.5 (2.0–7.5)	5.6 (2.6–9.3)	9.1 (4.7–13.0)	3.0 (1.2–5.0)	0.7 (0.3–2.0)	0.8 (0.3–2.1)	8.7 (6.6–11.1)	8.6 (6.5–11.0)
Zimbabwe	27.4 (19.5–35.1)	6.9 (4.2–9.9)	24.3 (13.1–37.7)	31.4 (20.2–44.4)	8.0 (4.7–12.6)	6.8 (3.9–10.6)	20.9 (9.6–31.0)	20.9 (9.6–31.0)	3.5 (1.8–5.5)	5.6 (3.0–8.5)	2.4 (0.0–5.8)	0.4 (0.0–1.1)	1.7 (0.7–3.8)	1.7 (0.7–3.8)	0.7 (<0.1–5.1)	0.7 (<0.1–5.1)
Western Sub-Saharan Africa	8.4 (5.5–11.4)	1.3 (0.8–2.0)	28.6 (18.5–39.1)	33.2 (24.1–43.1)	15.9 (10.4–23.2)	13.7 (8.8–19.9)	21.7 (10.0–32.0)	21.6 (9.9–31.9)	3.6 (1.9–5.5)	3.7 (1.9–5.7)	5.1 (1.9–8.0)	1.4 (0.1–2.8)	5.1 (0.4–18.9)	5.0 (0.4–20.1)	0.5 (<0.1–2.5)	0.4 (<0.1–2.4)
Benin	12.2 (7.5–17.7)	1.3 (0.7–2.4)	37.7 (23.4–51.9)	44.3 (31.5–55.4)	11.3 (5.6–18.8)	8.8 (4.1–15.1)	22.1 (10.1–32.6)	22.1 (10.1–32.6)	2.7 (1.4–4.4)	3.2 (1.5–5.0)	2.5 (0.2–5.5)	0.6 (0.0–1.8)	3.3 (<0.1–18.5)	3.3 (<0.1–18.5)	<0.1 (<0.1–1.2)	<0.1 (<0.1–1.2)
Burkina Faso	5.7 (3.1–8.6)	0.5 (0.2–0.8)	46.4 (30.9–59.2)	50.8 (38.4–64.4)	8.4 (2.9–16.6)	6.0 (2.0–12.9)	22.2 (10.2–32.7)	22.2 (10.2–32.7)	2.9 (1.2–5.1)	3.8 (1.6–6.6)	7.0 (1.4–13.3)	3.5 (0.0–8.9)	4.6 (<0.1–34.1)	4.6 (<0.1–34.1)	<0.1 (<0.1–3.1)	<0.1 (<0.1–3.1)
Cabo Verde	5.7 (3.5–8.6)	1.3 (0.7–2.2)	5.5 (2.4–10.4)	8.0 (4.1–13.7)	22.0 (13.6–31.9)	21.0 (13.2–30.6)	19.0 (8.5–28.3)	19.0 (8.5–28.3)	3.3 (1.6–5.4)	3.5 (1.6–5.7)	5.5 (1.9–9.2)	1.9 (0.2–4.1)	0.2 (<0.1–1.1)	0.2 (<0.1–1.1)	<0.1 (<0.1–6.3)	<0.1 (<0.1–6.3)
Cameroon	9.4 (6.0–13.1)	1.1 (0.5–1.9)	19.3 (10.4–30.8)	25.8 (15.8–37.7)	20.9 (13.2–29.7)	18.3 (11.5–26.1)	21.8 (10.0–32.1)	21.8 (10.0–32.1)	1.9 (0.9–3.1)	2.9 (1.4–4.8)	7.3 (3.3–11.4)	2.6 (0.4–5.1)	2.0 (0.1–7.1)	2.0 (0.1–7.1)	<0.1 (<0.1–1.6)	<0.1 (<0.1–1.6)
Chad	9.8 (5.4–14.6)	2.5 (1.2–4.2)	47.3 (32.6–60.6)	51.6 (39.7–65.9)	8.4 (2.9–17.8)	6.0 (2.1–13.8)	22.3 (10.2–32.8)	22.3 (10.2–32.8)	2.6 (1.2–4.5)	3.8 (1.7–6.3)	2.4 (<0.1–5.4)	1.4 (0.0–3.7)	7.0 (0.4–22.9)	7.0 (0.4–22.9)	1.3 (<0.1–7.7)	1.3 (<0.1–7.7)
Côte d'Ivoire	10.6 (7.0–14.6)	4.1 (2.2–7.0)	29.4 (17.1–43.7)	36.6 (24.5–48.9)	15.5 (8.3–25.4)	12.7 (6.4–21.5)	20.8 (9.5–31.0)	20.8 (9.5–31.0)	6.0 (3.3–9.1)	6.9 (3.8–10.4)	4.8 (0.4–9.6)	1.4 (0.0–4.0)	1.6 (<0.1–5.7)	1.6 (<0.1–5.7)	0.2 (<0.1–1.2)	0.2 (<0.1–1.2)
Gambia	14.0 (9.3–19.4)	1.1 (0.5–1.9)	34.9 (21.2–48.8)	41.9 (28.7–53.4)	14.2 (7.3–23.0)	11.3 (5.6–19.1)	21.9 (10.1–32.3)	21.9 (10.1–32.3)	5.8 (3.0–9.0)	7.7 (4.1–11.8)	2.9 (0.3–5.9)	0.7 (0.0–2.1)	2.2 (<0.1–20.9)	2.2 (<0.1–20.9)	0.1 (<0.1–1.5)	0.1 (<0.1–1.5)
Ghana	7.5 (4.3–11.1)	1.6 (0.8–2.6)	14.2 (7.1–23.9)	19.7 (11.2–30.3)	20.3 (12.9–30.3)	18.4 (11.5–27.6)	22.3 (10.3–32.8)	22.3 (10.3–32.8)	1.6 (0.7–2.7)	1.9 (0.9–3.1)	6.8 (1.7–11.7)	1.2 (0.0–3.1)	2.9 (<0.1–18.5)	2.9 (<0.1–18.5)	<0.1 (<0.1–0.7)	<0.1 (<0.1–0.7)
Guinea	15.5 (9.8–21.6)	1.9 (1.0–3.3)	43.6 (28.5–56.2)	48.8 (36.2–60.4)	9.9 (4.2–17.7)	7.3 (3.1–14.2)	22.3 (10.3–32.8)	22.3 (10.3–32.8)	3.7 (1.9–6.0)	4.6 (2.3–7.2)	1.1 (0.0–2.7)	0.2 (0.0–0.8)	2.7 (0.7–7.1)	2.7 (0.7–7.1)	0.1 (<0.1–1.3)	0.1 (<0.1–1.3)
Guinea-Bissau	5.9 (3.6–8.4)	0.8 (0.4–1.3)	41.0 (25.8–54.2)	46.9 (33.9–58.0)	11.2 (5.0–19.4)	8.5 (3.7–15.5)	22.3 (10.3–32.8)	22.3 (10.3–32.8)	3.7 (1.9–5.9)	4.4 (2.2–6.8)	3.5 (0.8–6.7)	0.9 (0.0–2.2)	2.2 (<0.1–22.7)	2.2 (<0.1–22.7)	<0.1 (<0.1–1.3)	<0.1 (<0.1–1.3)
Liberia	7.6 (4.3–11.3)	1.3 (0.6–2.3)	38.4 (23.3–51.5)	44.9 (32.1–56.0)	11.8 (5.7–20.2)	9.0 (4.4–15.8)	23.3 (10.8–34.1)	23.3 (10.8–34.1)	2.1 (0.9–3.7)	2.8 (1.2–5.0)	3.3 (0.5–6.7)	1.2 (<0.1–2.8)	0.9 (<0.1–3.3)	0.9 (<0.1–3.3)	0.1 (<0.1–2.0)	0.1 (<0.1–2.0)
Mali	12.4 (7.4–17.8)	1.2 (0.5–2.2)	46.8 (31.2–59.6)	51.3 (38.0–65.0)	9.0 (3.1–17.7)	6.5 (2.3–13.8)	21.9 (10.1–32.3)	21.9 (10.1–32.3)	2.9 (1.3–4.8)	3.6 (1.6–6.0)	1.4 (0.3–2.6)	0.8 (0.0–2.0)	7.3 (<0.1–26.7)	7.3 (<0.1–26.7)	1.3 (<0.1–13.3)	1.3 (<0.1–13.3)
Mauritania	7.2 (3.7–11.1)	0.7 (0.3–1.4)	14.9 (7.2–25.1)	20.4 (11.4–30.8)	22.6 (13.2–34.5)	20.3 (11.6–31.1)	19.6 (8.8–29.2)	19.6 (8.8–29.2)	3.0 (1.3–5.1)	3.7 (1.6–6.2)	0.0 (0.0–0.0)	<0.1 (0.0–0.0)	8.0 (1.7–19.5)	8.0 (1.7–19.5)	1.9 (<0.1–13.0)	1.9 (<0.1–13.0)

	Smoking		Household air pollution		Ambient particulate matter		Handwashing		Secondhand smoke		Alcohol use		High temperature		Low temperature	
	M	F	M	F	M	F	M	F	M	F	M	F	M	F	M	F
Niger	5.6 (3.4–8.2)	0.5 (0.2–0.9)	51.0 (35.2–69.0)	55.1 (40.4–75.8)	7.5 (2.1–19.0)	5.3 (1.5–14.5)	21.4 (9.8–31.7)	21.4 (9.8–31.7)	2.7 (1.3–4.5)	3.7 (1.7–5.8)	0.2 (0.0–0.9)	0.1 (0.0–0.4)	8.4 (0.5–23.3)	8.4 (0.5–23.3)	1.6 (<0.1–11.5)	1.6 (<0.1–11.5)
Nigeria	6.1 (4.2–8.5)	1.0 (0.5–1.5)	20.8 (12.9–30.2)	23.5 (15.9–32.6)	19.3 (12.1–28.3)	17.6 (10.8–25.8)	21.7 (9.9–31.8)	21.6 (9.9–31.8)	4.2 (2.2–6.4)	2.8 (1.5–4.3)	6.9 (2.7–11.0)	1.5 (<0.1–3.3)	7.4 (1.0–34.7)	6.8 (0.7–34.3)	0.8 (<0.1–2.3)	0.7 (<0.1–2.0)
Sao Tome and Principe	6.6 (4.3–9.6)	1.3 (0.6–2.3)	17.3 (8.5–28.6)	23.3 (13.5–34.4)	12.5 (6.9–19.8)	11.0 (6.0–17.6)	17.4 (7.7–26.1)	17.4 (7.7–26.1)	1.2 (0.5–1.9)	1.4 (0.7–2.2)	6.7 (2.6–10.7)	2.3 (0.2–4.9)	0.9 (<0.1–3.7)	0.9 (<0.1–3.7)	<0.1 (<0.1–1.9)	<0.1 (<0.1–1.9)
Senegal	9.9 (6.7–14.0)	0.6 (0.3–1.0)	32.1 (18.7–45.3)	38.6 (26.3–50.1)	15.1 (7.9–24.1)	12.0 (5.9–20.1)	18.8 (8.4–28.1)	18.8 (8.4–28.1)	7.3 (4.1–10.7)	8.8 (5.0–12.9)	0.1 (0.0–0.4)	<0.1 (0.0–0.1)	4.2 (<0.1–30.1)	4.2 (<0.1–30.1)	0.1 (<0.1–2.2)	0.1 (<0.1–2.2)
Sierra Leone	12.7 (7.0–18.7)	1.8 (0.8–3.2)	41.7 (26.6–54.7)	47.5 (34.8–58.5)	10.5 (4.5–18.6)	7.9 (3.5–14.6)	22.2 (10.2–32.6)	22.2 (10.2–32.6)	4.0 (1.5–7.2)	5.6 (2.2–10.0)	4.8 (1.4–8.5)	1.4 (0.0–3.4)	1.8 (<0.1–4.3)	1.8 (<0.1–4.3)	0.2 (<0.1–1.5)	0.2 (<0.1–1.5)
Togo	17.6 (12.8–22.9)	3.3 (1.8–5.3)	32.2 (19.3–46.2)	39.8 (27.4–51.7)	13.0 (7.0–20.7)	10.5 (5.7–16.7)	22.0 (10.1–32.5)	22.0 (10.1–32.5)	3.6 (2.0–5.4)	3.9 (2.2–5.7)	2.9 (0.4–6.1)	0.7 (0.0–2.2)	2.7 (<0.1–13.0)	2.7 (<0.1–13.0)	0.1 (<0.1–0.9)	0.1 (<0.1–0.9)
